# Regio‐ and Stereocontrolled‐Synthesis of a Heterocycle Fragment Collection Using Palladium Catalyzed C‐H Arylation

**DOI:** 10.1002/chem.202503126

**Published:** 2025-11-10

**Authors:** Amalia‐Sofia Piticari, Daniele Antermite, Harry J. Linkhorn, Natalia A. Larionova, Matthew P. Webster, James A. Bull

**Affiliations:** ^1^ Department of Chemistry Imperial College London Molecular Sciences Research Hub Wood Lane London W12 0BZ UK; ^2^ AbbVie Inc. 1 North Waukegan Road North Chicago IL 60064 USA

**Keywords:** heterocycles, C‐H functionalization, fragment synthesis, piperidine, pyrrolidine

## Abstract

Saturated heterocycles are valuable fragments in drug discovery due to their polarity, 3D structure, and potential for versatile binding modes, yet their controlled functionalization remains challenging. Existing methods often require pre‐functionalized substrates and provide limited control over substitution patterns, restricting access to diverse exit vectors. Here, we report a campaign achieving systematic control over the position and orientation of exit vectors in heterocycles through the synthesis of a structurally diverse fragment collection using aminoquinoline‐directed C–H functionalization. The study prioritizes five‐ and six‐membered N‐heterocycles, as well as rings with sulfonyl and difluoromethylene units. Aminoquinoline directing groups installed at C(2), C(3), or C(4) enable β‐arylation, and are subsequently removed to reveal carboxylic acids, primary amides, primary alcohols, or nitriles. The resulting 44 fragments, now part of AbbVie's compound collection, combine structural novelty with favorable physicochemical properties. Finally, a simple script is provided for rapid analysis of fragment properties.

## Graphical abstract

44 novel heterocycle fragments are prepared through a C–H functionalization strategy. Systematic variation of exit vectors and polar functionality provides a set of fragments that sit in attractive fragment chemical space.

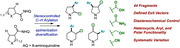

## 1 Introduction

**Figure 1:**
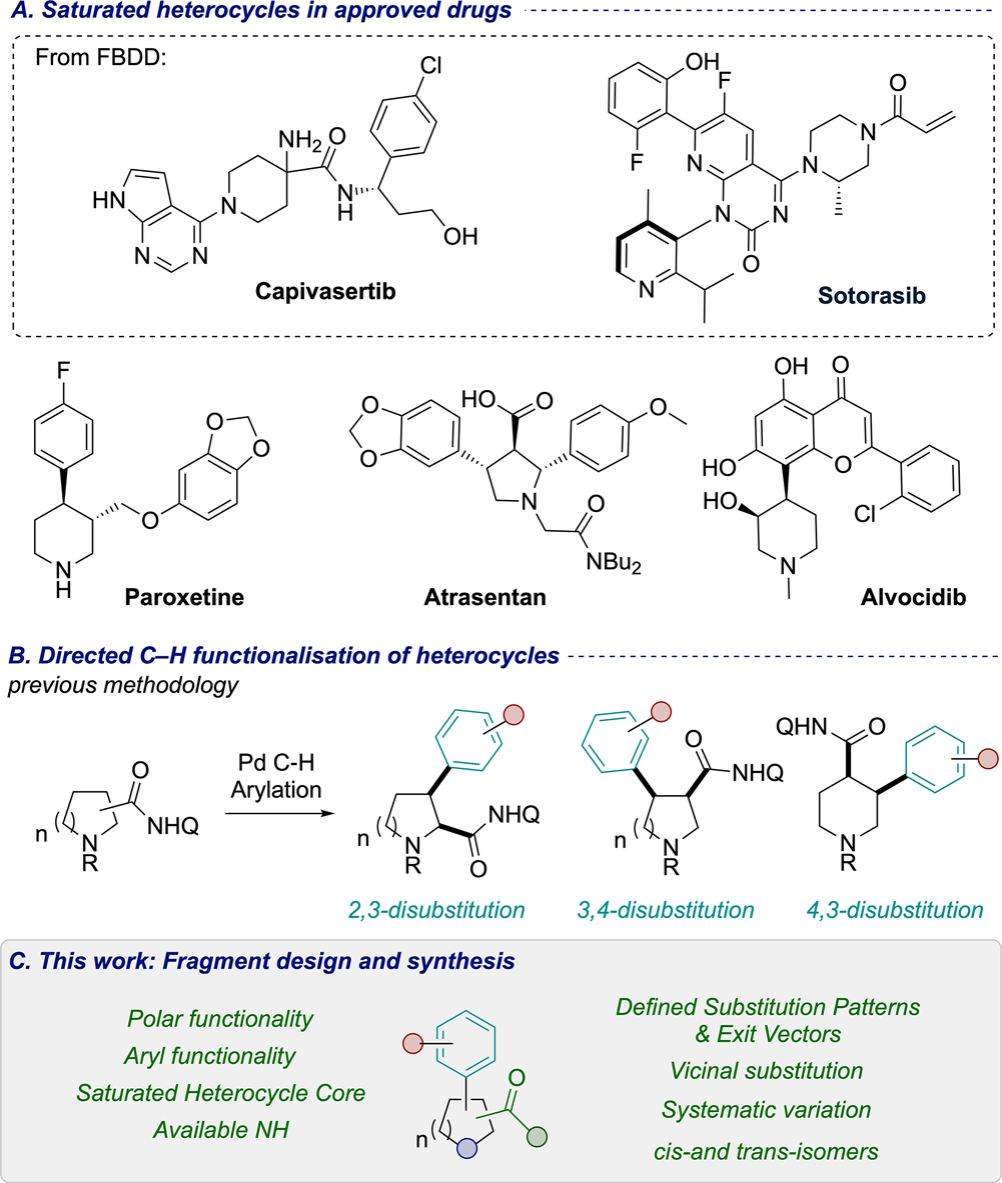
A) Selectedheterocycle containing drugs including those developed by fragment based drug discovery (FBDD). B) Previous methodologies for directed C‐H arylation of heterocycles. c) Our approach to arylated heterocyclic fragments using directed C–H arylation.

Fragment based drug discovery (FBDD) has become an established and powerful approach to the discovery of small molecule lead compounds.^[^ ^1^, ^2^ ^]^ The screening of low molecular weight fragments can both cover chemical space more effectively than screening larger compounds in traditional high throughput screens, and provide better quality leads through the elaboration of efficient binding fragment hits.^[^ ^3^, ^4^ ^]^ Eight current marketed drugs owe their development to this approach including Capivasertib and Sotorasib (Figure 1A).^[^ ^1^, ^5^ ^]^ Fragment screening has also been shown to unveil new binding modalities, including with “undruggable” targets.^[^ ^6^ ^]^ The curation of screening libraries to ensure valuable fragment‐like properties is therefore an important endeavor to ensure quality hits.^[^ ^7^ ^]^ Astex defined a Rule‐of‐3 (Ro3; molecular weight < 300, logP < 3, H‐bond donors or acceptors < 3) and later a Rule‐of‐2 (Ro2) for physicochemical property space to provide optimal fragment properties for screening by X‐Ray.^[^ ^8^, ^9^ ^]^ Additional parameters such as shape and the ability to grow fragments in different defined 3D directions are also of paramount importance.^[^ ^10^ ^]^ The concept of fragment oriented synthesis emerged in early 2010s,^[^ ^11^, ^12^ ^]^ and 3D fragments have since received significant attention,^[^ ^13^, ^14^ ^]^ to move away from predominantly flat fragment collections. Given the importance of saturated heterocycles in medicinal chemistry and in approved drugs,^[^ ^15^, ^16^, ^17^ ^]^ it is unsurprising that within the demands for new fragments, collections of substituted heterocycles have been particularly desirable. Saturated heterocycles provide valuable fragment structures with potential for 3D elaboration along defined exit vectors.^[^ ^18^, ^19^, ^20^, ^21^, ^22^ ^]^ Notable recent synthetic works from O'Brien,^[^ ^23^, ^24^, ^25^, ^26^, ^27^ ^]^ Marsden and Nelson,^[^ ^28^, ^29^, ^30^, ^31^, ^32^ ^]^ Spring,^[^ ^33^, ^34^, ^35^, ^36^ ^]^ and others^[^ ^37^, ^38^, ^39^, ^40^, ^41^ ^]^ have aimed to provide defined substitution patterns across heterocyclic rings with appropriate fragment properties. There remains considerable demand for new synthetic approaches to such designer heterocyclic fragments to access new chemical and IP space, with controlled access to different exit vectors across a collection and a need to provide the appropriate elements for binding.

Metal‐catalyzed C–H functionalization has enormous potential to aid diverse functionalization of intact heterocycles along C(sp^3^)–H bonds to prepare fragment compounds.^[^ ^42^, ^43^, ^44^, ^45^, ^46^, ^47^, ^48^, ^49^ ^]^ We previously reported methods to achieve C–H functionalization on pyrrolidines and piperidines with aminoquinoline (AQ) amide directing groups at C(2)‐,^[^ ^50^, ^51^ ^]^ C(3)‐,^[^ ^52^, ^53^, ^54^ ^]^ and C(4)‐positions using palladium catalysis (Figure 1B).^[^ ^55^, ^56^, ^57^, ^58^, ^59^, ^60^, ^61^, ^62^, ^63^, ^64^, ^65^, ^66^, ^67^, ^68^, ^69^ ^]^ We identified these approaches as potentially suitable to prepare fragment collections and enable diverse functionalization. However, our experience has been that the C–H arylation and the subsequent removal of directing groups on different heterocycles with varied ring size and heteroatom, as well as position of substitution, requires carefully tailored reaction conditions. Indeed, the removal of the directing groups is often a significant challenge for the preparation of screenable compounds.[^70^, ^71^, ^72^, ^73^, ^74^, ^75^ ^]^

Here, we report the design and full synthesis efforts to prepare a collection of heterocyclic fragments using

C–H functionalization methodologies to install arene substitutes vicinal to the directing group (Figure 1C). We demonstrate high levels of regiochemical and stereochemical diversity in the preparation of the fragments, with control from the C–H functionalization processes, and subsequent epimerization, starting from inexpensive and readily available heterocyclic carboxylic acids. Divergent synthetic elaboration is demonstrated in the removal of AQ amide directing group, to unveil useful polar functionality. Overall, we demonstrate the potential to populate 3D space, and to access different substitution patterns with control over a range of different exit vectors.

## 2 Results and Discussion

### 2.1 Fragment Design

In a collaboration between our group at Imperial and AbbVie, we designed a library of fragment compounds for use in screening programmes, targeting at least 50 mg of each final product. While numerous drug substructures feature aryl‐substituted saturated heterocycles (Figure 1A), both commercially available variants and established methodologies to access these sub‐structures are extremely limited. We predominantly targeted N‐heterocycles, aiming to maintain the polar NH functionality in the final fragment, and include aryl and polar functionalities to reflect binding elements often found in successful fragments,^[^ ^43^ ^]^ as well as to explore favorable physicochemical properties and attractive fragment space. To achieve this, we employed a suite of Pd‐catalyzed AQ ‐directed C–H functionalization methodologies developed in our group over the past 10 years,^[^ ^50^, ^51^, ^52^, ^53^, ^54^, ^55^ ^]^ to introduce aromatic substituents vicinal to the directing group, the latter serving to mask polar functionalities. Inexpensive carboxylic acid heterocycles allowed straightforward installation of the directing group via amide coupling. Fragments were prepared in racemic form for AbbVie's screening, though for chiral substrates, enantiopure starting materials are available and retain enantiomeric excess under C–H functionalization conditions, enabling facile synthesis of enantiopure derivatives for hit compounds.^[^ ^50^, ^51^, ^52^, ^53^, ^54^ ^]^

**Figure 2:**
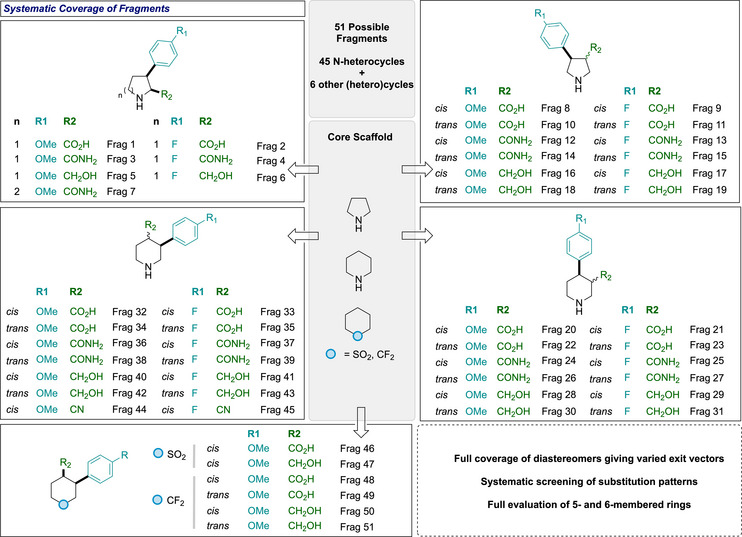
Systematic coverage of fragments installing aryl groups (4‐methoxyphenyl or 4‐fluorophenyl) and polar groups (carboxylic acid, primary amide, primary alcohol, or nitrile) with different exit vectors. Note FRAG31 and FRAG29 was not targeted for synthesis due to being paroxetine substructures and commercially available.

Conceptually, by varying the position of the directing group around the ring, the collection would give access to diverse substitution patterns, including 2,3‐, 3,4‐, and 4,3‐arrangements (Figure 2). 4‐Methoxyphenyl and 4‐fluorophenyl groups were used consistently as the aryl groups to provide medicinally relevant substituents. The previously developed arylation methods were optimized for *cis*‐selectivity, and would provide access to single diastereoisomeric products. We envisaged the stereochemical diversity of the collection could be further increased by a base‐mediated epimerization to afford the corresponding *trans*‐diastereomers to enable a broader range of exit vectors to be examined. Overall, we targeted a series of fragments, whereby we systematically explored a range of substitution patterns and relative stereochemistry, and optimized AQ cleavage to provide a practical guide for fragment synthesis.

### 2.2 Fragment Library Synthesis: C–H Arylation

**Scheme 1:**
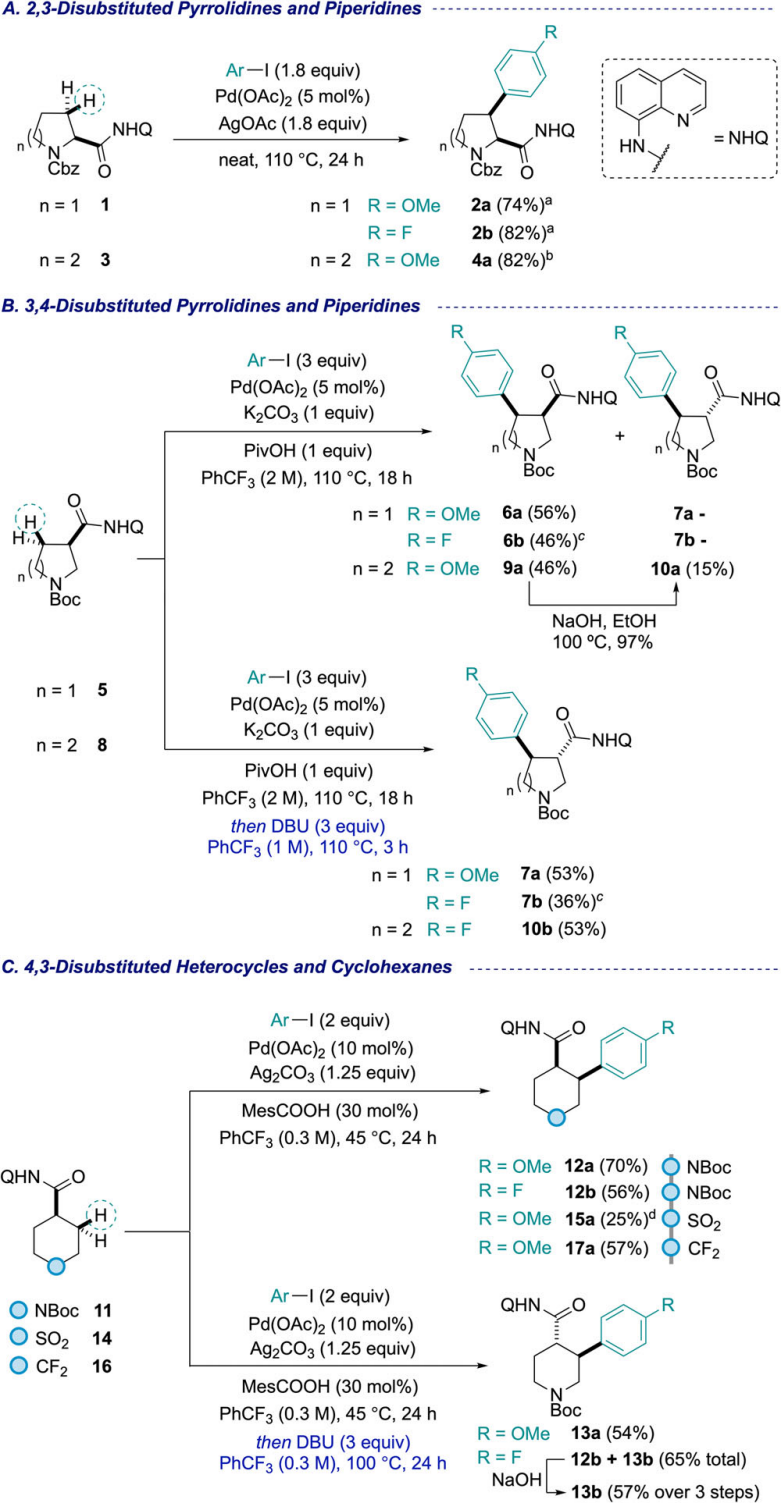
Arylation of saturated heterocycles to synthesize fragment precursors. Synthesis of A) 2,3‐disubstituted pyrrolidines and piperidines; B) 3,4‐disubstituted pyrrolidines and piperidines, and C) 4,3‐disubstituted heterocycles and cyclohexanes. ^a^Resubjection to reaction conditions required. ^b^Conditions: AgOAc (1 equiv), Pd(OAc)_2_, ArI (3 equiv), PhMe (3 M). ^c^Required 4 equiv ArI. ^d^Conditions: ArI (3 equiv), Ag_2_CO_3_ (2 equiv), 75 °C.

To begin, the AQ directing group was installed on a range of heterocyclic scaffolds via amide coupling (see Supporting Information for AQ amide preparation). Subsequent Pd‐catalyzed C–H arylation of these AQ amides was then carried out with a particular attention to improve scalability. First, *rac*‐N‐Cbz‐proline **1** with the AQ group installed at C(2) was subjected to C(3) arylation using 4‐iodoanisole (scheme 1). In this challenging series, Cbz‐protected pyrrolidines were previously found to be significantly more reactive than their Boc counterparts, and were therefore chosen for this study.^[^ ^50^, ^51^ ^]^ While performing the reaction on 0.2 mmol scale gave 64% isolated yield,^[^ ^50^, ^51^ ^]^ upon scaling the reaction to 5 mmol, run under solvent‐free conditions, incomplete conversion to the desired product **2a** was observed, resulting in a challenging separation of a mixture of **1** and **2a**. Resubjecting the crude mixture to the reaction conditions afforded **2a** in 74% yield. Investigations revealed that the order of reagent addition had a significant impact on conversion. The optimal sequence was found to be aryl iodide, AgOAc, Pd(OAc)_2_, and finally AQ substrate **1**. This effect was attributed to the high viscosity of substrate **1**, which hinders efficient stirring. The addition of co‐solvent, for example, toluene, to help with mixing proved less effective than optimizing the order of addition. When 4‐fluoro‐1‐iodobenzene was employed, the same incomplete conversion was observed. Applying the same procedure, including the order of addition, reaction conditions, and resubjection of the crude mixture, afforded the desired product **2b** in 82% yield. Extension to the 6‐membered ring, N‐Cbz piperidine AQ amide **3**, revealed higher reactivity compared to the piperidine system. On a 5 mmol scale, and under slightly modified conditions (addition of toluene as solvent and adjusted reagent equivalents), C(3)‐arylation with 4‐iodoanisole proceeded efficiently in a single operation, affording **4a** in 82% yield. These directed arylations gave exclusively the *cis*‐diastereomers.^[^ ^76^ ^]^

We next turned our attention to arylation reactions on substrates with a C(3) directing group, first applied on N‐Boc pyrrolidine **5** and targeting functionalization at C(4). On a 4 mmol scale, the arylation with 4‐iodoanisole proceeded with 56% yield and complete *cis‐*selectivity (**6a**). When more electron‐poor 4‐fluoroiodobenzene was employed, an additional equivalent of aryl iodide was required to achieve acceptable conversion, affording *cis‐*arylated pyrrolidine **6b**. The *trans* analogues **7a** and **7b** could also be accessed via a *cis*‐arylation and one‐pot epimerization sequence. The addition of DBU to the reaction at the end of the arylation gave epimerization in a one‐pot procedure, thereby providing access to the *trans* diastereomers in 53% and 36% yields respectively (**7a**/**7b**). Extension of this workflow to piperidine‐3‐carboxamide derivative **8** under identical conditions with 4‐iodoanisole furnished a separable mixture of *cis‐* and *trans*‐diastereomers in a combined 61% yield (d.r. 3:1, **9a**, **10a**). *cis*‐Arylated **9a**, could be further converted to **10a** by treatment with NaOH in almost quantitative yield. On larger scale, arylation with 1‐fluoro‐4‐iodobenzene gave an inseparable mixture of diastereoisomers. Therefore, the one‐pot arylation/epimerization protocol promoted by DBU was used to give *trans*‐4‐fluorophenyl piperidine **10b** as a single diastereomer in 53% yield.

The arylation of heterocycles and carbocycles with a C(4) directing group was achieved with good efficiency, delivering C(3)‐arylated *cis*‐products in up to 70% yield on a 4 mmol scale, providing gram quantities of products. Arylation of N‐Boc piperidine amide **11** with 4‐iodoanisole and 1‐fluoro‐4‐iodobenzene proceeded smoothly in 70% and 56% respectively (**12a**, **12b**). Notably, these reactions could be conducted reproducibly in round‐bottom flasks rather than sealed vials, facilitating set‐up. Access to the corresponding *trans*‐analogues was achieved via a *cis*‐arylation and epimerization sequence similar to the one employed on the 3,4‐disubstitution pattern described above. Thus, C(4)‐arylation with 4‐iodoanisole followed by treatment with DBU promoted > 90% epimerization to the *trans* isomer, affording **13a** in 54% overall yield. With 1‐fluoro‐4‐iodobenzene, epimerization with DBU was less effective (d.r. 7:3 *cis:trans*); however, subsequent exposure of the mixture to NaOH enabled rapid conversion of remaining *cis‐*product to *trans* **13b**, which was isolated in 57% overall yield over the 3 steps.

To expand beyond piperidine **11**, we next explored six‐membered rings **14** and **16** bearing sulfone (SO_2_) or difluoromethyl (CF_2_) groups, respectively. These selections were further facilitated by the commercial availability of the corresponding C(4) carboxylic acids, which serve as attachment point for AQ installation. Cyclic sulfone **14** proved more resistant to functionalization due to the coordinating nature of the sulfone moiety, however, increasing the temperature and the equivalents of the 4‐iodoanisole coupling partner provided access to **15a** in 25% yield. In contrast, difluorocyclohexane derivative **16** was arylated under standard conditions in 57% yield (**17a**). Epimerization of **17a** to the corresponding *trans*‐analogue was achieved in one step using NaOH in 84% yield (**18a** not shown), whilst retaining integrity of the AQ protecting group. Similarly, cleanly isolated C(3) *cis*‐arylated piperidines were seen to epimerize with NaOH in only 30 minutes to give the corresponding *trans*‐products with > 95% purity.

### 2.3 Fragment Library Synthesis: AQ Removal and Fragment Preparation

With the arylated heterocyclic precursors in hand, we proceeded with the divergent removal of the AQ group to access polar functional groups, along with subsequent deprotection steps. Notably, different conditions were required across the fragment series, and we examined a range of possibilities for interconversion.

**Scheme 2:**
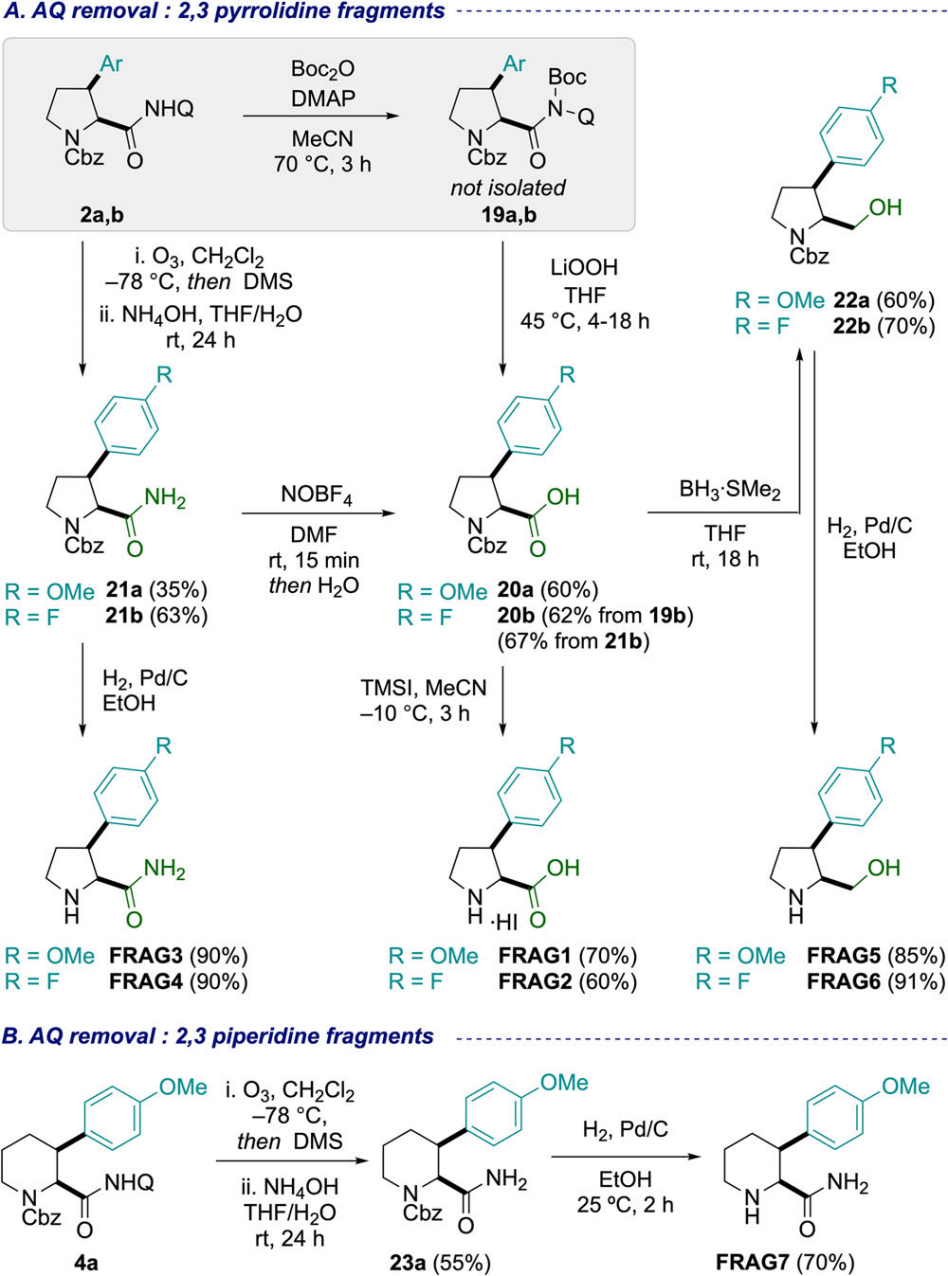
Aminoquinoline removal and diversification on 2,3‐disubstituted pyrrolidines and piperidines for the synthesis of fragments.

First, 2,3‐disubstituted pyrrolidines and piperidines were examined (Scheme 2). Simple NaOH‐mediated hydrolysis of **2a** led to mixtures of deprotected and epimerized products. Alternatively, activation of AQ amides **2a,b** with excess Boc_2_O in the presence of catalytic DMAP enabled LiOOH‐mediated hydrolysis to carboxylic acids **20a,b** in 60% and 62% yields, respectively, without epimerization. Cbz deprotection of these carboxylic acids by hydrogenolysis (H_2_, Pd/C) was unsuccessful, however, treatment with TMS iodide afforded the carboxylic acid fragments **FRAG1**, **FRAG2**, as the corresponding HI salts in > 60% yield. Ozonolysis of AQ amides **2a,b** followed by aqueous NH_4_OH workup furnished primary amides **21a,b** in 35% and 63% yield, respectively.^[^ ^72^ ^]^ Hydrogenolysis was successful on these substrates, affording **FRAG3, FRAG4** in 90% yield each. Additionally, primary amide **21b** could be converted to the corresponding carboxylic acid **20b** in 67% yield via NOBF_4_‐mediated nitrosylation, followed by hydrolysis.^[^ ^77^ ^]^ Protected pyrrolidine carboxylic acids **20a,b** also provided access to primary alcohol fragments. Acid **20a** was converted to alcohol **22a** via BH_3_·SMe_2_ reduction in 60% yield, followed by hydrogenolysis to give **FRAG5** in 85% yield. For **20b**, we also demonstrated the reduction‐hydrogenolysis in one‐pot, affording direct access to **FRAG6** in 63% yield over the two steps (not shown in figure). To further enable access to 2,3‐substituted piperidine fragments, **4a** was subjected to an ozonolysis–hydrogenolysis sequence analogous to **2a**, successfully affording the piperidine amide **FRAG7** (Scheme 2B).^[^ ^78^ ^]^

**Scheme 3:**
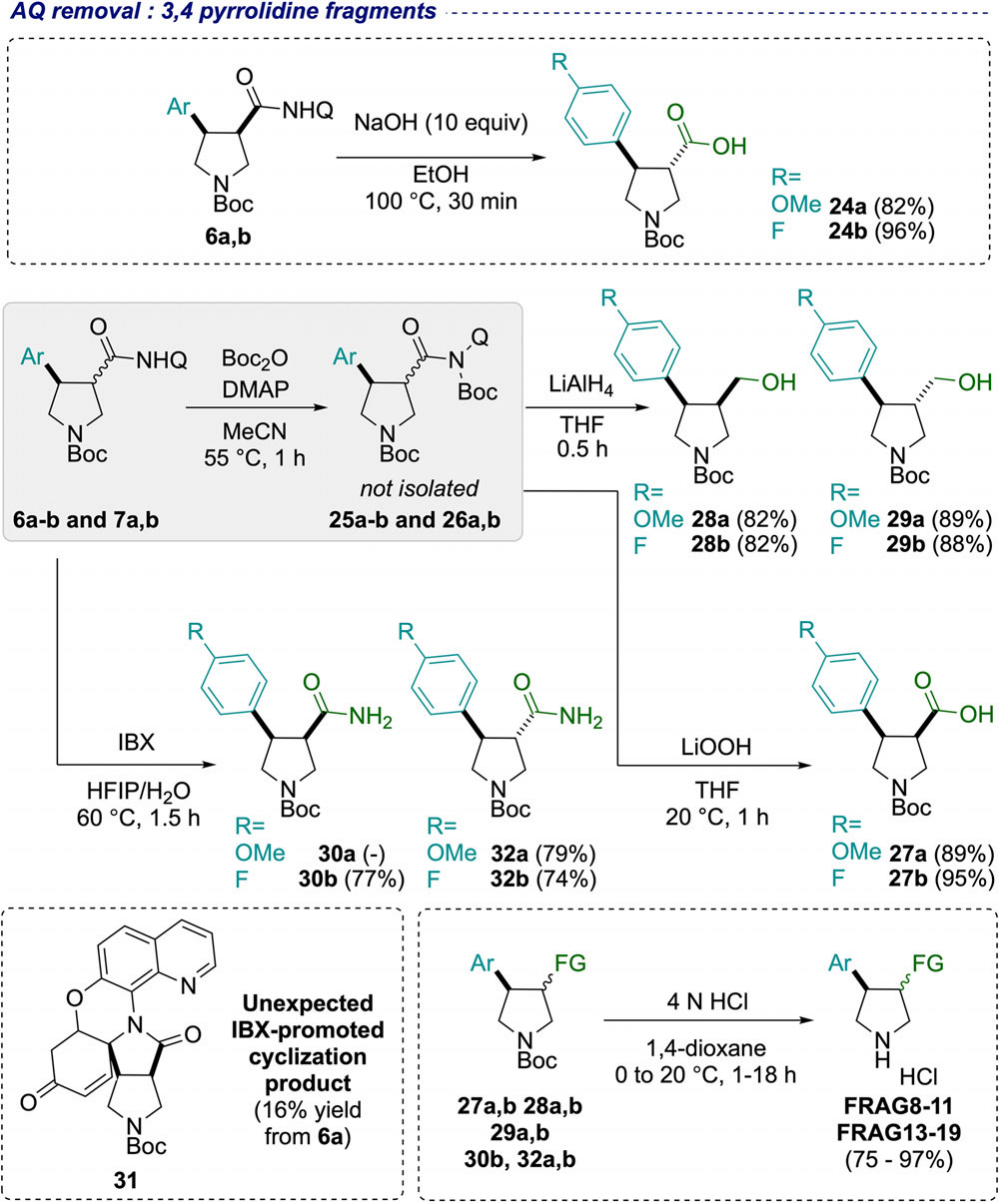
Aminoquinoline removal and diversification on 3,4‐disubstituted pyrrolidines for the synthesis of fragments.

AQ cleavage on the 3,4‐disubstituted pyrrolidine series began with an attempt to reveal the acid functionality on **6a** and **6b** via amide hydrolysis (Scheme 3). Treatment with NaOH resulted in rapid AQ amide hydrolysis and simultaneous epimerization to the thermodynamically preferred *trans*‐products **24a** and **24b**. These were obtained in 82% and 96% yield respectively, without the need for chromatographic purification. In order to access the *cis*‐diastereomers, the acid functionality was revealed via AQ amide activation followed by hydrolysis with LiOOH. This afforded 4‐methoxy‐ and 4‐fluorophenyl *cis‐*derivatives in excellent yields, after simple acid/base work‐up (**27a,b**). Boc amide activation also opened a more direct route to 3‐hydroxymethyl pyrrolidine derivatives by telescoped reduction with LiAlH_4_, which proceeded smoothly for both the *cis‐* and *trans*‐diastereoisomers (**28a,b** and **29a,b**). Next, we explored the synthesis of primary amide derivatives. A first attempt via ozonolysis as shown previously (*cf* Scheme 2), was unsuccessful due to overoxidation. Instead, we turned our attention to the chemically mediated oxidative cleavage of the AQ moiety using IBX in HFIP/H_2_O.^[^ ^71^ ^]^ Under these conditions, *cis‐* and *trans*‐4‐fluorophenyl pyrrolidines were successfully deprotected to the primary amide in 77% and 74% yields, respectively (**30b** and **32b**). Repeating the same protocol with *cis*‐4‐methoxyphenyl pyrrolidine **6a**, however, led to the formation of an unexpected spirocyclic compound **31** (see SI for proposed mechanism). On the other hand, *trans*‐4‐methoxyphenyl pyrrolidine **7a** successfully afforded the desired amide **32a** in 79% yield, presumably due to the *trans*‐configuration on the 5‐membered ring preventing cyclization. The final Boc deprotection was then conducted with 4 N HCl in 1,4‐dioxane giving the desired 3,4‐disubstituted pyrrolidine fragments as their HCl salts in 75–97% yield (acids **FRAG 8–11,** amides **FRAG 13–15,** and alcohols **FRAG16‐19**).

**Scheme 4:**
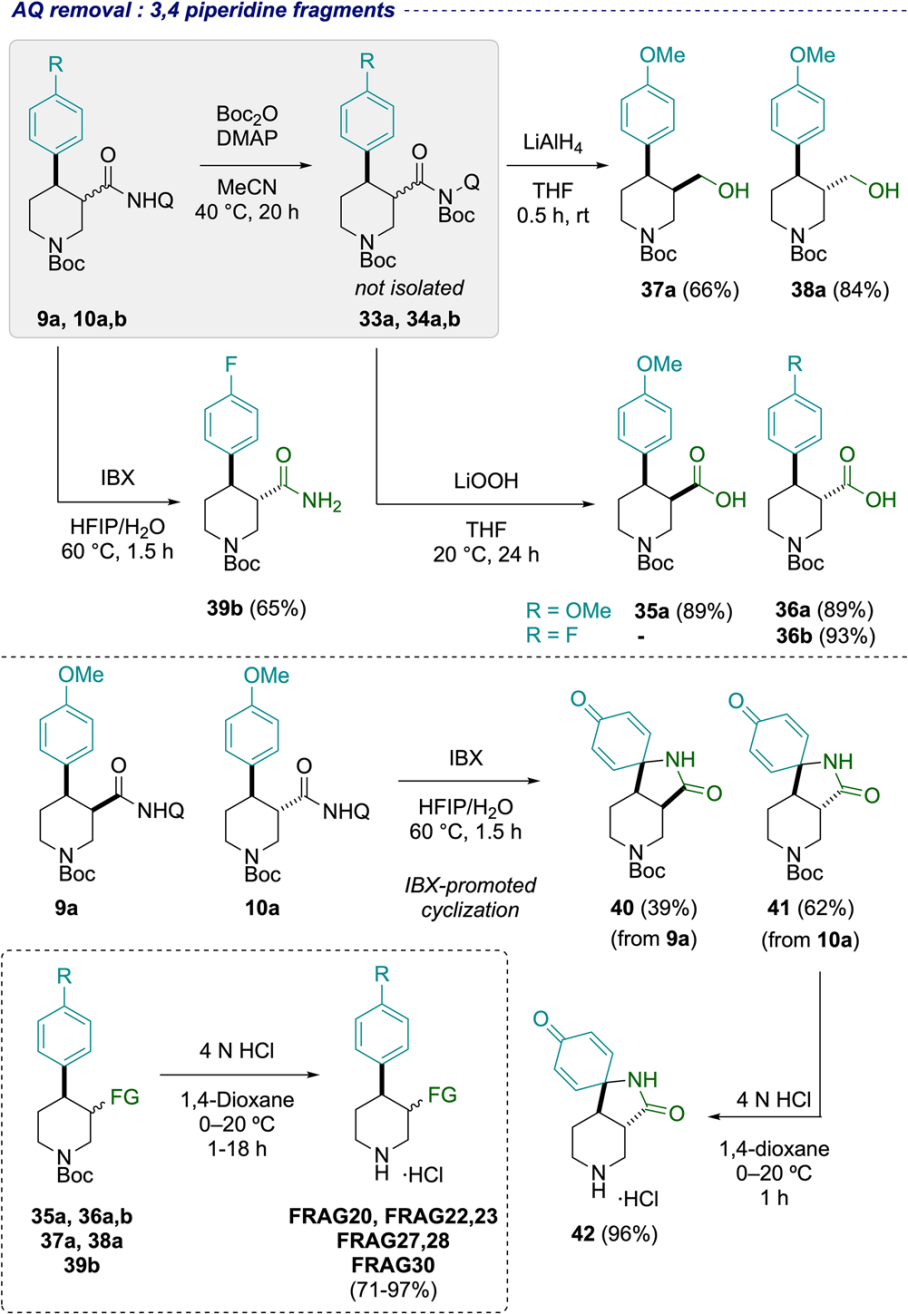
Aminoquinoline removal and diversification on 3,4‐disubstituted piperidines for the synthesis of fragments. Note FRAG31 was not targeted due to similarity with paroxetine and commercially available. See References 24a and 24b for synthesis of paroxetine derivatives by this approach.

Turning next to the 3,4‐disubstituted piperidine set, AQ cleavage was again attempted using NaOH in ethanol (Scheme 4). However, this failed to result in amide hydrolysis and epimerization was observed exclusively *(cf* Scheme 1).^[^ ^79^ ^]^ Alternatively, *trans‐*piperidine‐3‐carboxylic acids **36a** and **36b** were formed from *trans‐*carboxamides **10a** and **10b** using the telescoped Boc‐activation/hydrolysis protocol in 89% and 93% yield, respectively. On the other hand, *cis‐*configured piperidine amide **9a** proved to be poorly reactive under otherwise identical conditions in both Boc‐protection and hydrolysis steps, likely because of a higher steric congestion around the AQ amide compared to the *trans*‐isomer **10a** or the corresponding *cis*‐pyrrolidine **6a**. Nonetheless, using more forcing conditions, that is, higher temperature and reagent loadings in both steps, furnished *cis‐*4‐methoxyphenyl piperidine carboxylic acid **35a** in an excellent 89% overall yield (see the Supporting Information for full details). Similarly to the corresponding pyrrolidine series, a telescoped 2‐step protocol, involving amide activation followed by reduction with LiAlH_4_, provided straightforward access to 3‐hydroxymethyl piperidine derivatives *cis‐* **37a** and *trans‐* **38a** in 66% and 84% yield respectively. Attempts to access primary amide analogues via IBX‐mediated oxidative cleavage of **9a** and **10a**, however, were unsuccessful due to the electron‐rich nature of the aryl group. Instead, overoxidation of the 4‐methoxyphenyl substituent after AQ cleavage resulted in the formation of spirolactams **40** and **41** for both *cis*‐ *and trans*‐configured isomers.^[^ ^80^ ^]^ Retention of the relative stereochemistry was confirmed by Boc‐deprotection and analysis of coupling constants by ^1^H NMR for *trans*‐isomer **42**, consistent with an axial position of both piperidine protons at the ring junction. In contrast, the 4‐fluorophenyl substituent was found compatible with these oxidative conditions and primary amide **39b** was successfully isolated in 65% yield. Finally, Boc‐deprotection furnished the 3,4‐disubstituted piperidine fragments in 71–97% yield (acids **FRAG20, FRAG22, FRAG23**, amide **FRAG27,** and alcohols **FRAG28** and **FRAG30**).

**Scheme 5:**
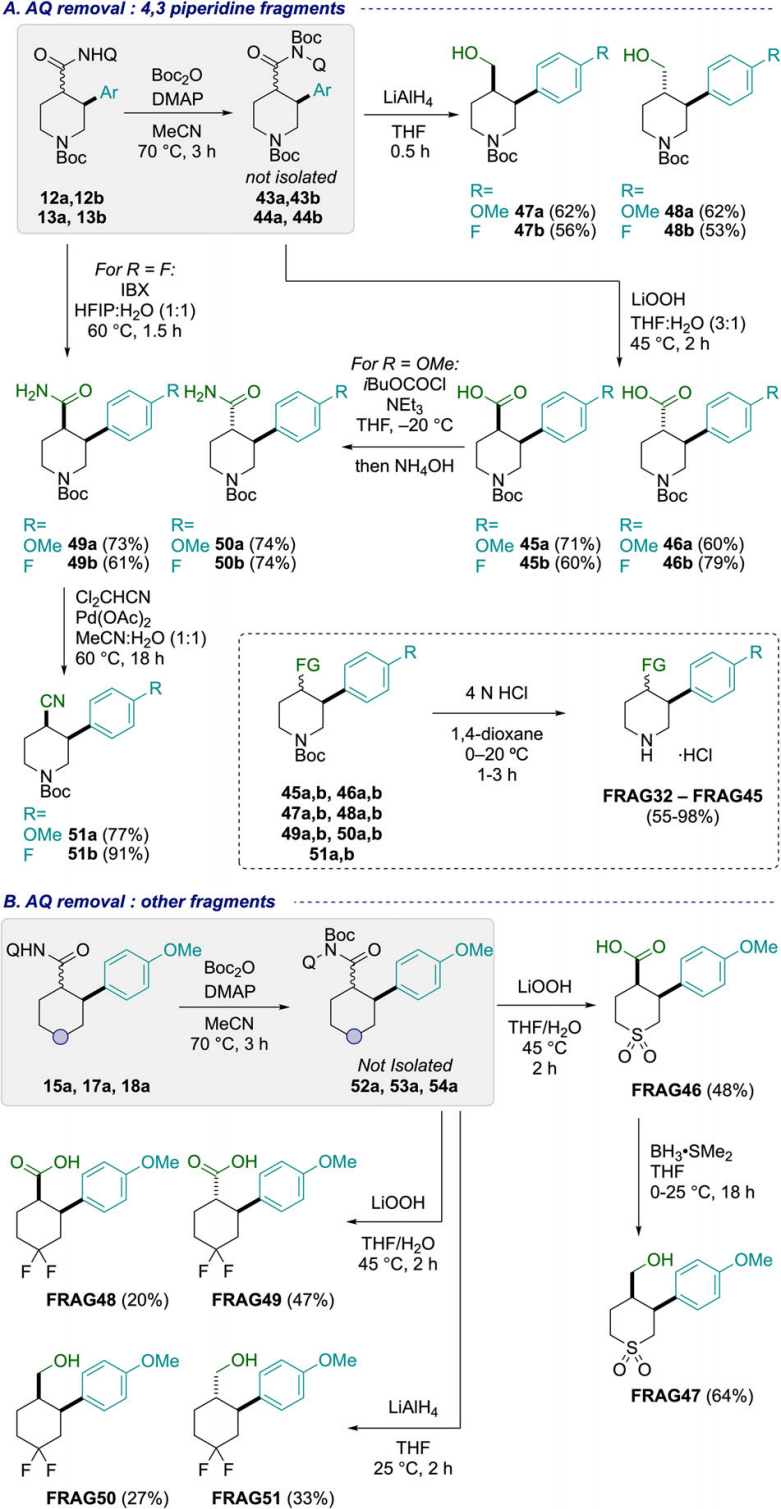
Aminoquinoline removal and diversification on 4,3‐disubstituted piperidine and other fragments.

Similar to other substitution patterns, diversification of the 4,3‐disubstituted piperidine series started from the Boc activated AQ amide intermediates **43a,b** and **44a,b** (Scheme 5A). Same‐pot LiOOH‐mediated cleavage provided access to each of the four carboxylic acid fragments **45a,b** and **46a,b** bearing methoxyphenyl and/or fluorophenyl groups in either *cis* or *trans* configurations in yields ranging from 60 to 79%. Moreover, LiAlH_4_ reduction of the Boc activated intermediates unveiled the corresponding alcohols **47a,b** and **48a,b**, with no epimerization observed on the *cis*‐substituted patterns.

Fluorophenyl piperidine amides were quickly accessed by IBX‐mediated oxidative cleavage of the AQ amides, affording **49b** and **50b** in good yields (61% and 74%, respectively). On the other hand, methoxyphenyl piperidine amides, which were prone to spirocyclization as observed in prior 3,4‐disubstituted systems, were subjected to an alternative approach. A telescoped one‐pot, two‐step procedure was employed from the available carboxylic acids **45a** and **46a**, involving carbonic anhydride formation with isobutyl chloroformate followed by direct ammonolysis with NH_4_OH,^[^ ^23^ ^]^ providing either the *cis*‐amide **49a** or the *trans*‐isomer **50a** in similarly high yields (>70%). Access to primary amides also enabled further diversification to nitrile substituents via Pd‐catalyzed dehydration with dichloroacetonitrile, (protocol adapted from Naka et al.^[^ ^81^ ^]^) affording nitriles **51a,b** in 77% and 91% yield. In our adaptation of this protocol, Pd(OAc)_2_ was successfully employed in place of Pd(TFA)_2_, ensuring compatibility with Boc‐protected substrates. The dehydration of C(3) amides **32a** and **39b** to nitriles was also successful attempted as a proof of concept to further expand the fragment collection (not shown, see SI for details). With all key substituents installed, final Boc deprotection using 4 N HCl in 1,4‐dioxane furnished the complete 4,3‐piperidine fragment set as their HCl salts (acids **FRAG32‐35,** amides **FRAG36‐39,** alcohols **FRAG40‐43**, and nitriles **FRAG44‐45**; see SI for further information).

Similar workflows were applied to other heterocyclic scaffolds starting with the Boc activation of the AQ amides (**15a, 17a, 18a**) to unmask polar functionalities and directly revealed fragment structures (Scheme 5B). LiOOH‐promoted hydrolysis of sulfone intermediate **52a** (from **15a**), provided carboxylic acid sulfone **FRAG46** in 48% yield. Selective carboxylic acid reduction of **FRAG46** to the alcohol **FRAG47** occurred in 64% yield, preserving the sulfone functionality. Difluorocyclohexyl fragments, **FRAG48** and **FRAG49**, were also generated from LiOOH hydrolysis of the Boc‐activated intermediates (**53a** and **54a**), though these were more resistant to hydrolysis, requiring double the equivalents of LiOOH in comparison to the piperidine examples. Direct LiAlH_4_ reduction of **53a** and **54a** afforded alcohol fragments **FRAG50** and **FRAG51** in moderate yields (27% and 33%).

**Scheme 6:**
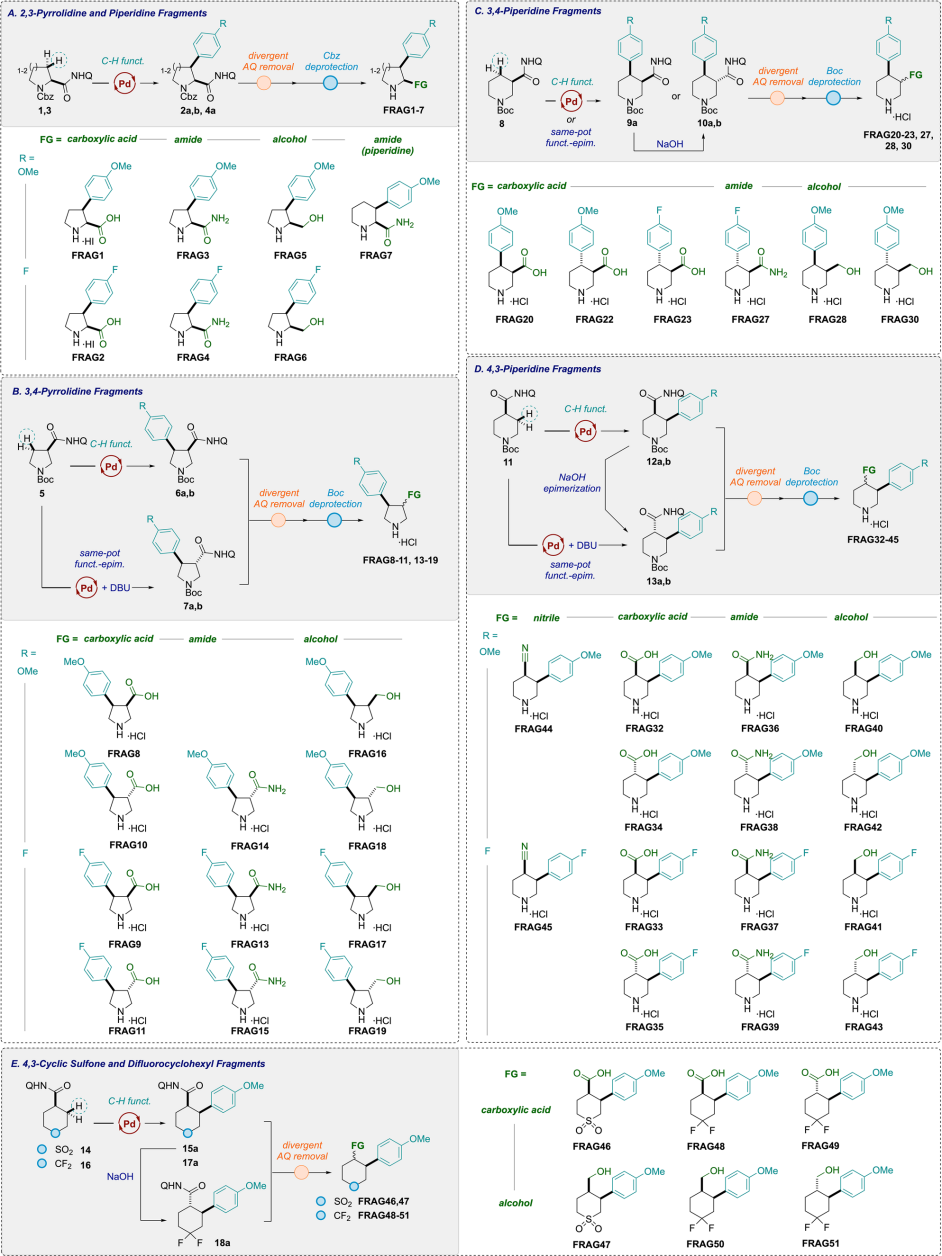
Summary of all 38 N‐heterocycle fragments synthesized and 6 cyclic sulfone and difluorocyclohexyl fragments.

In summary, from the 51 initially considered fragments in Figure 2, 44 were successfully prepared (see Scheme 6 for full summary). Of those not prepared, two fragments (**FRAG29** and **FRAG31**) were not attempted due to commercial availability, which results from being a sub‐structure of paroxetine. The nonavailability of the other fragments itself displays the challenge in covering this chemical space. In the same series, *cis*‐substituted fluoroarene derivatives (**FRAG21,25**) were not prioritized due to challenging chromatographic separation of a mix of *cis*/*trans*‐arylation products, which were instead readily fully converted to the *trans*‐analogues by epimerization. Attempts to deprotect the AQ group to form primary amides (**FRAG12,24,26**) under oxidative conditions in the presence of 4‐methoxypheny groups led to unexpected oxidative cyclization processes to form unusual novel spirocycles (3 compounds) when the PMP group was in sufficiently close proximity.

### 2.4 Analysis of the Fragment Library

**Figure 3:**
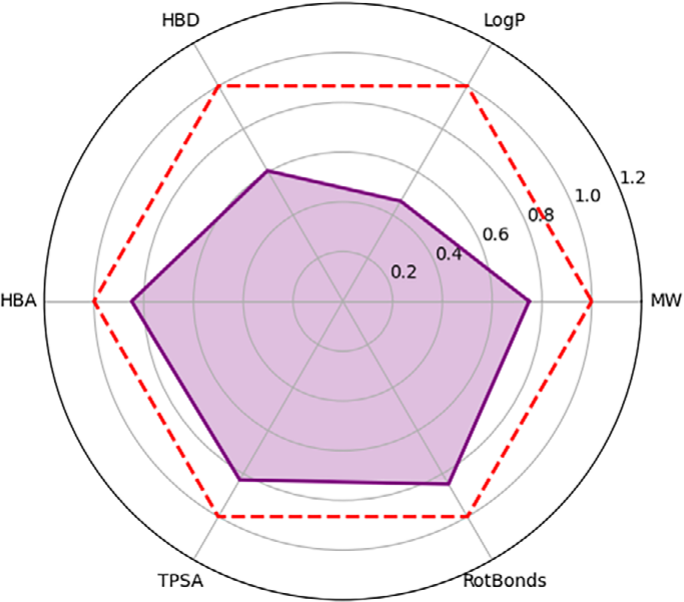
Radar plot showing normalized average physicochemical properties of the fragment library (purple) relative to Rule‐of‐3 (Ro3) thresholds (red dashed line). Properties are scaled to Ro3 limits to facilitate direct comparison across parameters.

With a set of 44 fragments prepared we sought to evaluate the overall physicochemical profile of the resulting fragment library. To do this, we developed a simple analysis tool built with RDKit, freely available on GitHub^[^ ^82^ ^]^ which systematically computes molecular properties and generates suggestive library statistics. Given that SMILES representations typically encode absolute stereochemistry, the tool automatically generates the corresponding enantiomer by inverting both defined stereocenters, thereby ensuring that the properties calculated are representative of racemates. For each compound, key fragment‐relevant properties are computed: molecular weight (MW), lipophilicity (LogP), hydrogen bond donors (HBD), hydrogen bond acceptors (HBA), topological polar surface area (TPSA), and rotatable bonds. These are then averaged across the whole library and normalized relative to Ro3 limits. As shown in the radar plot (Figure 3), the average profile of the library (purple) is positioned well within the Ro3‐defined fragment space (red dashed line), supporting the suitability of the series for FBDD efforts.

To investigate the structural novelty of our synthesized fragments, we compared these against 2 major commercial fragment collections, ZINC (containing over 230 million compounds) and the Enamine Fragment Collection (over 200 thousand compounds). From these, 400 fragments from ZINC and 33713 fragments from Enamine contained the same central hetero‐ or carbocyclic motifs as our synthesized fragments (pyrrolidine, piperidine, six‐membered cyclic sulfones, or *gem*‐difluorocyclohexane) and were extracted. Morgan fingerprints were computed for all synthesized and extracted fragments using RDKit,^[^ ^83^ ^]^ and the corresponding 2048‐bit fingerprints embedded in two dimensions using UMAP (Uniform Manifold Approximation and Projection),^[^ ^84^ ^]^ a nonlinear dimensionality reduction method known to efficiently capture both local and global relationships in chemical space.^[^ ^85^ ^]^

**Figure 4:**
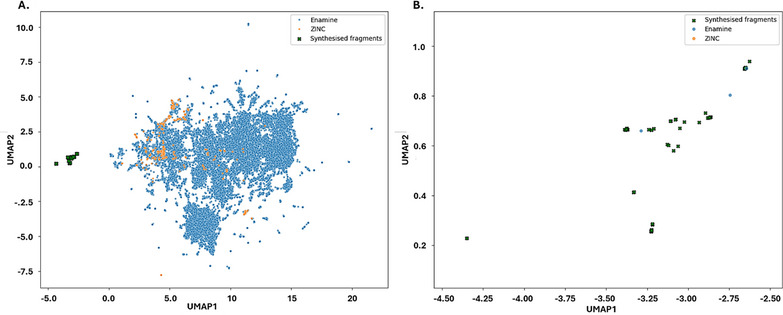
UMAP embedding of synthesized versus commercial fragments. a) Morgan fingerprints of synthesized fragments and extracted ZINC and Enamine fragments containing the same hetero‐ or carbocyclic central motifs were embedded in two dimensions using UMAP. Synthesized fragments (highlighted with crosses) occupy a region largely distinct from commercial fragments from both Enamine and ZINC. b) Zoom‐in on the embedding region occupied by the synthesized fragments, highlighting the internal diversity and spatial distribution within this newly explored chemical space.

As shown in Figure 4A, the synthesized fragments (green crosses) cluster together, reflecting the expected local structural relationships. Commercial fragments also describe 3–4 broad groups, consistent with the 4 scaffold types extracted, with Enamine displaying greater diversity than ZINC and a more cohesive charting of chemical space.

Most strikingly, our synthesized fragments occupy regions largely separate from the commercial collections, accessing chemical space so far untapped. Since Morgan fingerprints encode substructural patterns, this separation indicates that commercial fragments rarely share comparable designs—particularly in the arrangement of substituents and the nature of exit vectors ‐ underscoring a structural space unmet in existing libraries.

A zoomed‐in view of the embedded synthesized region (Figure 4B) further reinforces this novelty: only a few outlier Enamine compounds overlap with our collection, while the bulk of commercial space remains distant. Together, these findings suggest that elaboration of our designs would access previously unexplored regions of chemical space, underscoring not only the effectiveness of the C–H functionalization methodology but also the success of our synthetic campaign strategy in charting new opportunities for fragment‐based discovery.

## 3 Conclusion

Here we report a successful campaign for the preparation of heterocyclic fragments through a directed C–H functionalization strategy. Through variation in the position of the directing group (C2, C3, C4), with corresponding arylation at C3, C4, and C3 respectively, a set of distinct exit vectors was systematically installed across the scaffolds. C–H arylation proceeded with excellent regio and stereocontrol to prepare single *cis*‐diastereoisomer products, while controlled epimerization accessed the *trans*‐isomers. The directing groups were converted to polar functionality in the form of carboxylic acids, primary amides, primary alcohols, and nitriles, demonstrating the synthetic tractability of the approach. Moreover, the divergent synthetic routes and, in some cases, multiple derivatization pathways provide a guide to practitioners to remove the AQ group and enable rapid exploration of related fragment space. A total of 44 fragments, predominantly pyrrolidines and piperidines, but also sulfone and difluoromethylene containing rings were successfully prepared in 50 mg quantities or above and conforming to Ro3 thresholds. We report and use a simple script for the rapid analysis of fragment properties from SMILES, whilst a comparison of synthesized fragments with a subsection of ZINC database and Enamine library demonstrate that our fragments occupy regions of chemical space largely separate from the commercial collections.

## Supporting Information

The authors have cited additional references within the Supporting Information.^[^ ^86^, ^87^ ^]^

## Conflict of Interest

The authors declare no conflict of interest.

## Supporting information



Supporting Information

## Data Availability

The data that support the findings of this study are available in the supplementary material of this article.
