# Supplementary material for: Regio‐ and Stereocontrolled‐Synthesis of a Heterocycle Fragment Collection Using Palladium Catalyzed C‐H Arylation
Source: Chemistry. 2025 Nov 10;31(71):e03126. doi: 10.1002/chem.202503126 (PMC12734658; doi:10.1002/chem.202503126)
Supplement: Supplementary file 1 — Supporting Information [file CHEM-31-e03126-s001.pdf]

SUPPORTING INFORMATION

Regio- and Stereocontrolled Synthesis of a Heterocycle Fragment Collection using Palladium Catalyzed C–H Arylation

Amalia-Sofia Piticari,[1] Daniele Antermite,[1] Harry J. Linkhorn,[1] Natalia Larionova,[1] Matthew Webster,[2] and James A. Bull[1]*

[1] Department of Chemistry, Imperial College London, Molecular Sciences Research Hub, London W12 0BZ, U.K.

[2] AbbVie Inc. 1 North Waukegan Road, North Chicago, IL 60064, USA.

Email: j.bull@imperial.ac.uk

Contents

General Experimental 3

Additional compounds to SI 5

Proposed Mechanism for IBX Spirocyclization 6

Section 1: Synthesis and Characterisation of Aminoquinoline (AQ) Amides 7

- **C2 Aminoquinoline Amides** 7
- **C3 Aminoquinoline Amides** 8
- **C4 Aminoquinoline Amides** 10

Section 2: 2,3-Disubstituted Fragments 13

- **Synthesis overview** 13
- **C2-Directed CH Arylations** 16
- **2,3-Disubstituted Fragment Synthesis** 19

Section 3: 3,4-Disubstituted Fragments 30

- **Synthesis overview** 30
- **C3-Directed CH Arylations** 37
- **3,4-Disubstituted Fragment Synthesis** 44

Section 4: 4,3-Disubstituted Fragments 77

- **Synthesis overview** 77
- **C4-Directed CH Arylations** 84
- **4,3-Disubstitued Fragment Synthesis** 91

Section 5: Medicinal Chemistry Analysis 118

References 121

^{1}H, ^{13}C and ^{19}F NMR Spectra 122

General Experimental

All non-aqueous reactions were carried out under an inert atmosphere (argon) with flame-dried glassware using standard techniques, unless otherwise specified. Anhydrous solvents were obtained by filtration through drying columns (MeCN) or used as supplied (α,α,α-trifluorotoluene – Sigma product number: 547948 and THF – Fisher product number: 11924899). Reactions in sealed tubes were run using Biotage microwave vials (2–5 mL or 10–20 mL recommended volumes). Aluminum caps equipped with molded butyl/PTFE septa were used for reactions in α,α,α-trifluorotoluene. Simple butyl septa were used for reactions in MeCN or alcoholic solvents.

Chromatographic purification was performed either manually using 230–400 mesh silica, with the indicated solvent system according to standard techniques or using an automated flash chromatography Biotage® Selekt system with Biotage® Sfaar® HC cartridges (50-100 g) under the indicated conditions. Analytical thin-layer chromatography (TLC) was performed on precoated, glass-backed silica gel plates. Visualization of the developed chromatogram was performed by UV absorbance (254 nm) and/or stained with a potassium permanganate solution or ninhydrin solution in ethanol.

IR spectra were recorded as solids or neat liquids on an Agilent Cary 630 FTIR spectrometer and are reported in wavenumbers (cm^{-1}) to the nearest integer.

Nuclear magnetic resonance spectra were recorded on 400 or 500 MHz spectrometers. The frequency used to record the NMR spectra is given in each assignment and spectrum (^{1}H NMR at 400 or 500 MHz; ^{13}C NMR at 101 MHz or 126 MHz; ^{19}F NMR at 376 or 471 MHz). Chemical shifts for ^{1}H NMR spectra were recorded in parts per million from tetramethylsilane with the residual protonated solvent resonance as the internal standard ($CDCl_3$: δ 7.27 ppm, DMSO-d_6: δ 2.50 ppm, CD_3OD: δ 3.31 ppm). Data was reported as follows: chemical shift (multiplicity [s = singlet, d = doublet, t = triplet, m = multiplet and br = broad], coupling constant, integration and assignment). *J* values are reported in Hz. All multiplet signals were quoted over a chemical shift range. ^{13}C NMR spectra were recorded with complete proton decoupling. Chemical shifts were reported in parts per million from tetramethylsilane with the solvent resonance as the internal standard ($^{13}CDCl_3$: δ 77.0 ppm, $(^{13}CD_3)_2SO$: δ 39.5 ppm, $^{13}CD_3OD$: δ 49.0 ppm). Assignments of ^{1}H and ^{13}C spectra, as well as *cis*- or *trans*-configuration, were based upon the analysis of δ and *J* values, as well as DEPT, COSY, HMBC, HSQC and NOESY experiments where appropriate. ^{19}F NMR spectra were recorded with proton decoupling unless otherwise stated. ^{19}F NMR spectra are indirectly referenced to $CFCl_3$, automatically via direct measurement of the absolute frequency of the deuterium lock signal by the spectrometer hardware.

NMR spectra were conducted at 273 K unless otherwise mentioned.

Most *tert*-butyloxycarbonyl (Boc) containing compounds appeared as a mixture of rotamers in the NMR spectra at rt. In some cases, NMR experiments at 373 K were carried out in order to assign the signals.

The high resolution mass spectrometry (HRMS) analyses were performed using electrospray ion source (ESI) or atmospheric pressure chemical ionization (APCI) using an atmospheric solids analysis probe (ASAP). ESI was performed using a Waters LCT Premier equipped with an ESI source operated either in positive or negative ion mode. The software used was MassLynx 4.1, this software does not account for the electron and all the calibrations/references are calculated accordingly, *i.e.* $[M+H]^+$ is detected and the mass is calibrated to output [M+H]. APCI was performed using an Orbitrap XL or Xevo G2S using an ASAP to insert samples into the APCI source. The sample was introduced at ambient temperature and the temperature increased until the sample vaporized.

All melting points were determined in open glass capillaries and are uncorrected.

Reagents: Commercial reagents were used as supplied or purified by standard techniques where necessary. $Pd(OAc)_2$ was purchased from Fluorochem Ltd and used as supplied (purity >98%). 8-Aminoquinoline was purchased from TCI Chemicals and used as supplied. Ag_2CO_3 and K_2CO_3 (powder, 325 mesh) were purchased from Sigma-Aldrich, with K_2CO_3 flame-dried as part of the reaction set-up. 2-Iodoxybenzoic acid (IBX) was purchased as a mixture of the title compound (30 wt.% or 45 wt.%) and isophthalic acid and benzoic acid stabilisers (commercially known as stabilised IBX, SIBX), with the mixture used as supplied without any further purification.

Compound experimental procedures are organised in order of synthesis grouping similar transformation together.

Additional compounds to SI

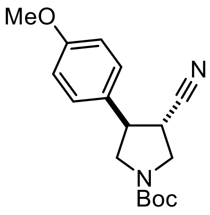

S1

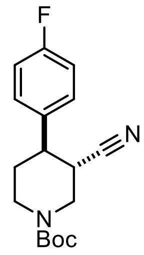

S2

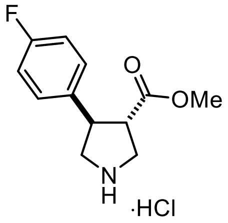

S3

Proposed Mechanism for IBX Spirocyclization

Proposed mechanism for the formation of **31** from **6a** based of similar reported IBX mediated cyclizations.[34,40]

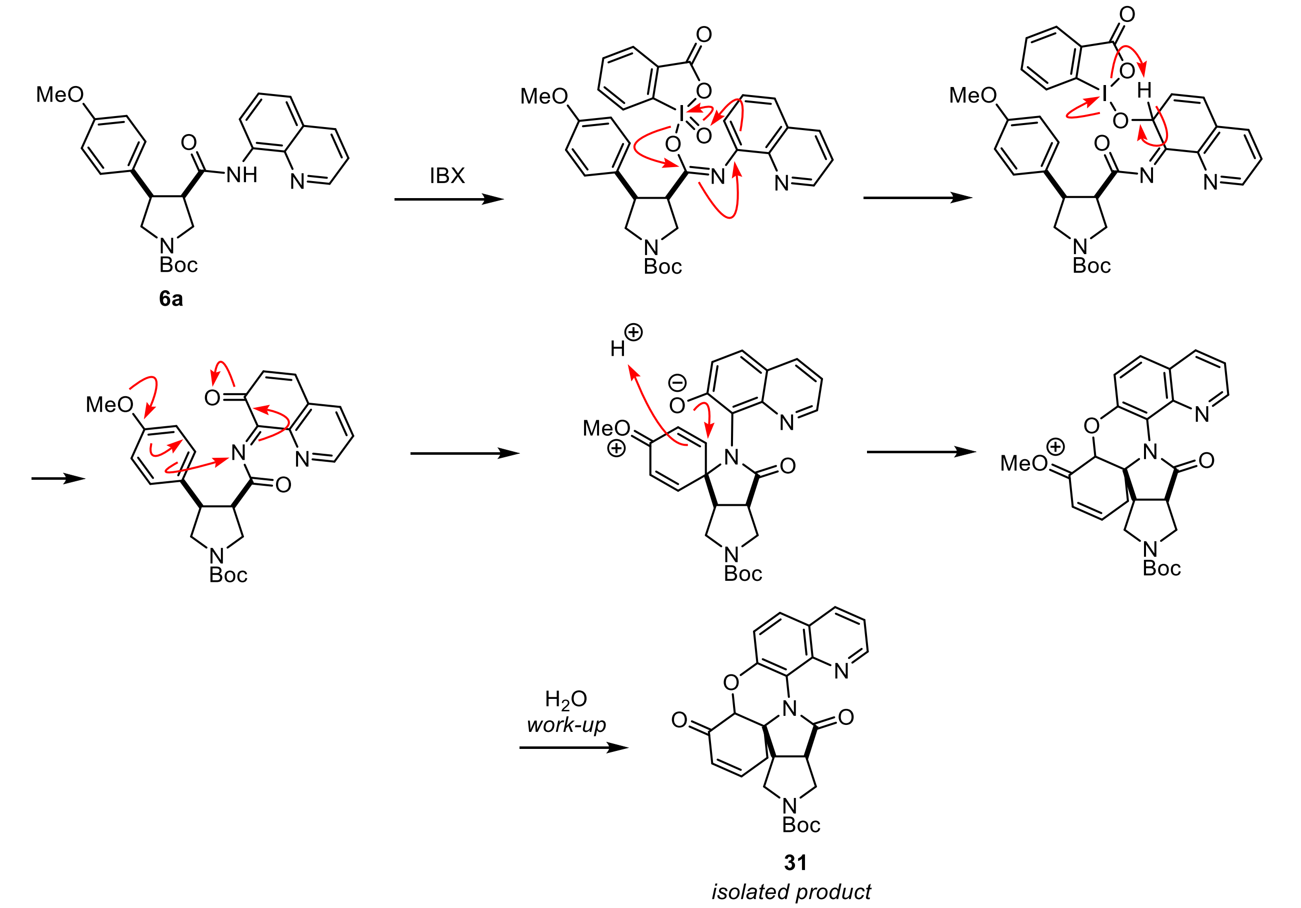

Proposed mechanism for the formation of **40** from **9a** based of similar reported IBX mediated cyclizations.[34] It is assumed **41** is formed via an analogous mechanism.

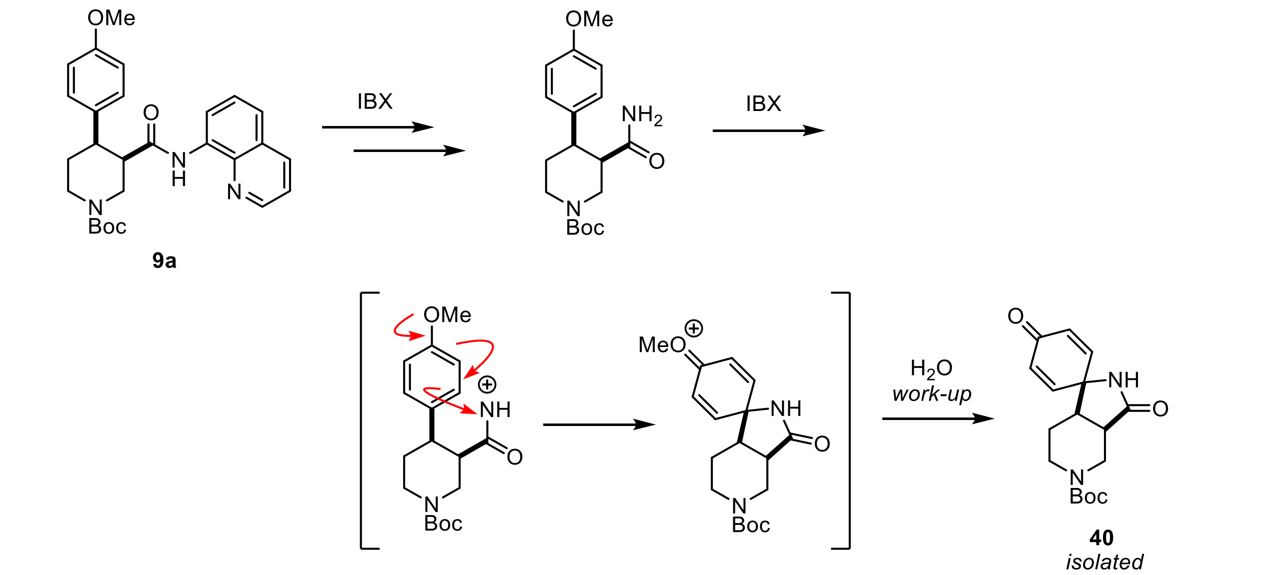

It is also noted treatment of **12a** with IBX lead to an analogous cyclisation (not isolated) thus **49a** was accessed via **45a** instead of directly from **12a**.

Section 1: Synthesis and Characterisation of Aminoquinoline (AQ) Amides

C2 Aminoquinoline Amides

Benzyl 2-(quinolin-8-ylcarbamoyl)pyrrolidine-1-carboxylate (1)

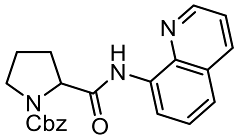

Prepared according to literature procedure at 40 mmol scale.[23a] HOBt hydrate (9.20 g, 60.0 mmol, 1.50 equiv) was added to a solution of 1-[(benzyloxy)carbonyl]pyrrolidine-2-carboxylic acid (rac-*N*-Cbz proline, 10.0 g, 40.0 mmol, 1.00 equiv), 8-aminoquinoline (6.30 g, 44.0 mmol, 1.10 equiv) and EDC·HCl (9.2 g, 48.0 mmol, 1.20 equiv) in CH_2Cl_2 (80 mL, 0.5 M) and the resulting solution was stirred at rt for 15 h. The solvent was removed under reduced pressure, a solution of sat. aq. $NaHCO_3$ (100 mL) was added, and the aqueous layer was extracted with EtOAc (3 × 250 mL). A solution of sat. aq. NH_4Cl (20 mL) was added after the first EtOAc extraction, to aid the separation of aqueous and organic layers. The combined organic extracts were dried with Na_2SO_4 and filtered. The solvent was removed under reduced pressure, and purification by flash column chromatography (30–60% Et_2O/hexane) afforded amide **1** as a viscous pale yellow oil (11.1 g, 75%).

^{1}H NMR (400 MHz, $CDCl_3$, mixture of rotamers) δ 10.50 and 10.33 (br s, 1 H, NH), 8.75 (d, *J* = 12.5 Hz, 2 H, 2 × HC_{Ar}), 8.17 (d, *J* = 8.2 Hz, 1 H, HC_{Ar}), 7.54 (d, *J* = 5.7 Hz, 2 H, 2 × HC_{Ar}), 7.44 (dt, *J* = 10.0, 5.0 Hz, 2 H, 2 × HC_{Ar}), 7.39–7.29 (m, 1 H, HC_{Ar}), 7.16 (d, *J* = 7.4 Hz, 1 H, HC_{Ar}), 6.93 (dd, *J* = 17.0, 7.1 Hz, 2 H, 2 × HC_{Ar}), 5.35–5.01 (m, 2 H, OCH_2), 4.76–4.48 (m, 1 H, CH(C=O)), 3.91–3.52 (m, 2 H, NCH_2), 2.42–2.15 (m, 2 H, $NCHC\mathit{H_2}$), 2.15–1.90 (m, 2 H, $NCH_2C\mathit{H_2}$).

^{13}C NMR (101 MHz, $CDCl_3$, mixture of rotamers) δ 171.0 (C=O amide), 155.0 (C=O carbamate), 148.2 (C_{Ar}), 138.5 (C_{Ar} quat), 136.4 (C_{Ar} and C_{Ar} quat), 133.8 (C_{Ar} quat), 128.5 (2 × C_{Ar}), 127.9 (C_{Ar}), 127.7 (C_{Ar}), 127.6 (C_{Ar} quat), 127.3 (2 × C_{Ar}), 121.8 (C_{Ar}), 121.5 (C_{Ar}), 116.6 (C_{Ar}), 67.2 (OCH_2), 62.0 (*C*H(C=O)), 47.5 and 47.0 (NCH_2), 31.3 and 29.9 ($NCH\mathit{C}H_2$), 24.5 and 23.8 ($NCH_2\mathit{C}H_2$).

Characterisation information consistent with data previously reported in literature.[23a]

Benzyl 2-(quinolin-8-ylcarbamoyl)piperidine-1-carboxylate (3)

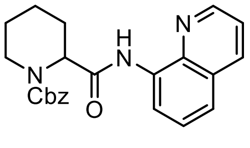

Prepared according to literature procedure at 20 mmol scale. [23b] HOBt hydrate (4.05 g, 30.0 mmol) was added to a solution of 1-[(Benzyloxy)carbonyl]piperidine-2-carboxylic acid (5.27 g, 20.0 mmol), 8-aminoquinoline (3.32 g, 23.0 mmol) and EDC·HCl (3.73 g, 24.0 mmol) in CH_2Cl_2 (40 mL, 0.5 M) and the resulting solution was stirred at rt for 15 h. The solvent was removed under reduced pressure, a solution of sat. aq. $NaHCO_3$ (100 mL) was added, and the aqueous layer was extracted with EtOAc (3 × 250 mL). A solution of sat. aq. NH_4Cl (20 mL) was added after the first EtOAc extraction, to aid the separation of aqueous and organic layers. The combined organic extracts were dried with Na_2SO_4 and filtered. The solvent was removed under reduced

pressure, and purification by flash column chromatography (10–20% EtOAc/hexane) afforded amide **3** as a viscous pale yellow oil (6.3 g, 80%).

^{1}H NMR (400 MHz, $CDCl_3$) δ 9.95 (s, 1 H, NH), 8.87–8.73 (m, 2 H, 2 × HC_{Ar}), 8.16 (dd, J = 8.2, 1.7 Hz, 1 H, HC_{Ar}), 7.57–7.49 (m, 2 H, 2 × HC_{Ar}), 7.46 (dd, J = 8.3, 4.2 Hz, 1 H, HC_{Ar}), 7.41–7.36 (m, 4 H, 4 × HC_{Ar}), 7.36–7.30 (m, 1 H, HC_{Ar}), 5.17 (s, 2 H, OCH_2), 4.32 (br s, 2 H, NCH_2), 2.96 (br s, 2 H, CH(C=O) and NCHC*H*H), 2.65 (ddt, J = 11.5, 7.6, 3.8 Hz, 1 H, NCHCH*H*), 2.05 (br s, 2 H, NCH_2CH_2), 1.94–1.79 (m, 2 H, $N(CH_2)_2CH_2$).

Characterisation information consistent with data previously reported in literature.[23b]

C3 Aminoquinoline Amides

tert-Butyl 3-(quinolin-8-ylcarbamoyl)pyrrolidine-1-carboxylate (5)

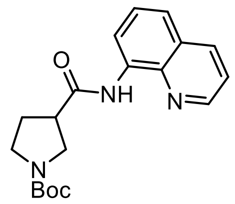

*Synthesis of amide **5** using EDC·HCl and HOBt:*

HOBt hydrate (2.56 g, 16.7 mmol) was added to a solution of *N*-Boc-pyrrolidine-3-carboxylic acid 1 (3.00 g, 14.0 mmol) and 8-aminoquinoline (2.41 g, 16.8 mmol) in dry CH_2Cl_2 (55 mL) at 25 °C, and was stirred for 5 min. EDC·HCl (3.20 g, 16.8 mmol) was then added and the solution was stirred for 5 days. The reaction was diluted with CH_2Cl_2 (75 mL), then a solution of sat. aq. $NaHCO_3$ (75 mL) was added and the aqueous layer was extracted with CH_2Cl_2 (3 × 150 mL). The combined organic extracts were dried over Na_2SO_4 and filtered. The solvent was removed under reduced pressure. The crude material was purified by flash column chromatography (15% to 50% EtOAc/pentane), the product containing fractions were combined and the solvent was removed under reduced pressure. Et_2O (20 mL) and pentane (20 mL) were added and the solvent was removed under reduced pressure to afford amide **5** as a white solid (4.30 g, 91%).

*Synthesis of amide **5** using EDC·HCl and DMAP:*

EDC·HCl (3.60 g, 18.7 mmol) was added to a solution of *N*-Boc-pyrrolidine-3-carboxylic acid 1 (4.00 g, 18.7 mmol) and 8-aminoquinoline (2.20 g, 15.6 mmol) in anhydrous CH_2Cl_2 (80 mL) at 0 °C. DMAP (70.1 mg, 0.6 mmol) was then added and the solution was stirred at 25 °C for 24 h. The reaction was diluted with CH_2Cl_2 (80 mL), then a solution of sat. aq. $NaHCO_3$ (80 mL) was added and the aqueous layer was extracted with CH_2Cl_2 (3 × 100 mL). The combined organic extracts were dried over Na_2SO_4 and filtered. The solvent was removed under reduced pressure. The crude material was purified by flash column chromatography (50% to 70% Et_2O/pentane) to afford amide **5** as a pale yellow solid (4.74 g, 89%).

Characterisation data from the two protocols was identical.

R_f 0.23 (25% EtOAc/pentane).

mp = 110–113 °C (from Et_2O/pentane).

IR (film)/cm^{-1} 3343 (NH), 2972, 1738, 1693 (C=O carbamate), 1672 (C=O amide), 1536, 1487, 1398, 1362, 1223, 1164, 1125, 1082, 986, 874, 821, 782, 686.

^{1}H NMR (400 MHz, DMSO-d_6, 373 K) δ 10.02 (br s, 1 H, NH), 8.91 (dd, *J* = 4.2, 1.7 Hz, 1 H, HC_{Ar}), 8.58 (dd, *J* = 7.7, 1.3 Hz, 1 H, HC_{Ar}), 8.36 (dd, *J* = 8.3, 1.7 Hz, 1 H, HC_{Ar}), 7.65 (dd, *J* = 8.2, 1.3 Hz, 1 H, HC_{Ar}), 7.60 (dd, *J* = 8.3, 4.2 Hz, 1 H, HC_{Ar}), 7.56 (t, *J* = 7.9 Hz, 1 H, HC_{Ar}), 3.63 (dd, *J* = 10.4, 7.5 Hz, 1 H, NC*H*HCHCO), 3.55–3.44 (m, 3 H, NCH*H*CHCO, CHCO, NC*H*HCH$_2$), 3.34 (ddd, *J* = 10.5, 7.5, 7.5 Hz, 1 H, NCH*H*CH$_2$), 2.27–2.19 (m, 1 H, NCH$_2$C*H*H), 2.18–2.09 (m, 1 H, NCH$_2$CH*H*), 1.43 (s, 9 H, C(CH$_3$)$_3$).

^{13}C NMR (101 MHz, DMSO-d_6, 373 K) δ 171.0 (C=O amide), 153.2 (C=O carbamate), 148.4 (C_{Ar}), 138.0 (C_{Ar} quat), 136.0 (C_{Ar}), 133.9 (C_{Ar} quat), 127.5 (C_{Ar} quat), 126.4 (C_{Ar}), 121.6 (C_{Ar}), 121.5 (C_{Ar}), 116.5 (C_{Ar}), 78.0 (*C*(CH$_3$)$_3$), 48.1 (N*C*H$_2$CHCO), 44.9 (N*C*H$_2$CH$_2$), 44.1 (*C*HCO), 28.5 (NCH$_2$*C*H$_2$), 27.8 (C(*C*H$_3$)$_3$).

HRMS (ESI$^+$) *m/z* Calculated for $C_{19}H_{24}N_3O_3$ [M+H] 342.1818; Found 342.1829.

Characterisation information consistent with data previously reported in literature. [24a]

tert-Butyl 3-(quinolin-8-ylcarbamoyl)piperidine-1-carboxylate (8)

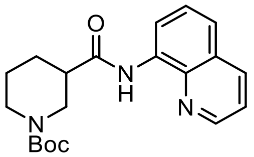

DIPEA (3.0 mL, 17.4 mmol) was added to a solution of *N*-Boc-piperidine-3-carboxylic acid (2.00 g, 8.70 mmol) and HATU (3.97 g, 10.4 mmol) in CH_2Cl_2 (40 mL) at 0 °C, and was stirred for 5 min. 8-Aminoquinoline (1.50 g, 10.4 mmol) was then added and the solution was stirred for 24 h at 25 °C. The reaction was diluted with CH_2Cl_2 (40 mL), then a solution of sat. aq. $NaHCO_3$ (40 mL) was added and the aqueous layer was extracted with CH_2Cl_2 (3 × 50 mL). The combined organic extracts were dried over Na_2SO_4 and filtered. The solvent was removed under reduced pressure. The crude material was purified by flash column chromatography (10% to 20% EtOAc/pentane), the product containing fractions were combined and the solvent was removed under reduced pressure. Et_2O (20 mL) and pentane (20 mL) were added and the solvent was removed under reduced pressure to afford amide **8** as a pale brown solid (2.73 g, 87%).

R_f 0.14 (15% EtOAc/pentane).

mp = 124–128 °C (from Et_2O/pentane).

IR (film)/cm^{-1} 3350 (NH), 2936, 1682 (C=O), 1525, 1486, 1423, 1389, 1366, 1327, 1257, 1241, 1163, 1147, 826, 792, 760.

^{1}H NMR (400 MHz, DMSO-d_6, 373 K) δ 10.00 (br s, 1 H, NH), 8.91 (dd, *J* = 4.2, 1.7 Hz, 1 H, HC_{Ar}), 8.60 (dd, *J* = 7.6, 1.4 Hz, 1 H, HC_{Ar}), 8.36 (dd, *J* = 8.3, 1.7 Hz, 1 H, HC_{Ar}), 7.64 (dd, *J* = 8.3, 1.4 Hz, 1 H, HC_{Ar}), 7.60 (dd, *J* = 8.3, 4.2 Hz, 1 H, HC_{Ar}), 7.56 (t, *J* = 7.9 Hz, 1 H, HC_{Ar}), 4.15–4.06 (m, 1 H, NC*H*HCHCO), 3.88–3.80 (m, 1 H, NC*H*HCH$_2$), 3.11 (dd, *J* = 13.1, 10.1 Hz, 1 H, NCH*H*CHCO), 2.94–2.86 (m, 1 H, NCH*H*CH$_2$), 2.84–2.75 (m, 1 H, CHCO), 2.12–2.04 (m, 1 H, N(CH$_2$)$_2$C*H*H), 1.81–1.70 (m, 2 H, N(CH$_2$)$_2$CH*H*, NCH$_2$C*H*H), 1.53–1.46 (m, 1 H, NCH$_2$CH*H*), 1.42 (s, 9 H, C(CH$_3$)$_3$).

^{13}C NMR (101 MHz, DMSO-d_6, 373 K) δ 171.2 (C=O amide), 153.5 (C=O carbamate), 148.2 (C_{Ar}), 137.8 (C_{Ar} quat), 135.9 (C_{Ar}), 133.9 (C_{Ar} quat), 127.3 (C_{Ar} quat), 126.3 (C_{Ar}), 121.4 (C_{Ar}), 121.2 (C_{Ar}), 116.1 (C_{Ar}), 78.3 (*C*(CH$_3$)$_3$), 45.7 (N*C*H$_2$CHCO), 43.3 (N*C*H$_2$CH$_2$), 42.7 (*C*HCO), 27.6 (C(*C*H$_3$)$_3$), 26.9 (N(CH$_2$)$_2$*C*H$_2$), 23.3 (NCH$_2$*C*H$_2$).

HRMS (ESI$^+$) *m/z* Calculated for $C_{20}H_{26}N_3O_3$ [M+H] 356.1974; Found 356.1986.

Characterisation information consistent with data previously reported in literature. [24a,b]

C4 Aminoquinoline Amides

***tert*-Butyl 4-(quinolin-8-ylcarbamoyl)piperidine-1-carboxylate (11)**

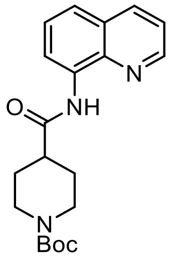

4-DMAP (508 mg, 4.00 mmol, 0.2 equiv) and EDC·HCl (4.20 g, 2.20 mmol, 1.1 equiv) were added to a solution of *N*-Boc-piperidine-4-carboxylic acid (4.60 mg, 20.0 mmol, 1.0 equiv) and 8-aminoquinoline (3.17 g, 22.0 mmol, 1.1 equiv) in CH_2Cl_2 (70 mL). After stirring at rt for 24 h, the reaction was diluted with CH_2Cl_2 (50 mL), then sat. aq. $NaHCO_3$ (50 mL) was added and the aqueous layer extracted with CH_2Cl_2 (3 x 50 mL). The combined organic extracts were dried over Na_2SO_4, filtered, and the solvent removed under reduced pressure. Purification of the residue by flash column chromatography (20% to 60% EtOAc/hexane) afforded amide **11** as a white solid (5.54 g, 16.5 mmol, 82%).

R_f 0.38 (35% EtOAc/hexane).

mp = 120–123 °C (from EtOAc).

IR (film)/cm^{-1} 3366 (NH br), 2929, 2983, 1692 (C=O), 1681 (C=O), 1528, 1409, 1297, 1163, 940.

^{1}H NMR (400 MHz, $CDCl_3$) δ 9.94 (br s, 1 H, NH), 8.86–8.72 (m, 2 H, 2 × HC_{Ar}), 8.16 (dd, *J* = 8.2, 1.7 Hz, 1 H, HC_{Ar}), 7.58–7.40 (m, 3 H, 3 × HC_{Ar}), 4.23 (br s, 2 H, 2 × NC*H*HCH$_2$), 2.89–2.82 (m, 2 H, 2 × NCH*H*CH$_2$), 2.70–2.54 (m, 1 H, CH(C=O)), 2.12–1.95 (m, 2 H, 2 × NCH$_2$C*H*H), 1.94–1.76 (m, 2 H, 2 × NCH$_2$CH*H*), 1.48 (s, 9 H, C(CH$_3$)$_3$).

^{13}C NMR (101 MHz, $CDCl_3$) δ 172.9 (C=O amide), 154.7 (C=O carbamate), 148.1 (C_{Ar}), 138.3 (C_{Ar} quat), 136.3 (C_{Ar}), 134.2 (C_{Ar} quat), 127.9 (C_{Ar} quat), 127.3 (C_{Ar}), 121.6 (C_{Ar}), 121.5 (C_{Ar}), 116.4 (C_{Ar}), 79.6 (*C*(CH$_3$)$_3$), 44.6 (2 × N*C*H$_2$CH$_2$), 43.2 (*C*H(C=O)), 28.6 (2 × NCH$_2$*C*H$_2$), 28.4 (C(*C*H$_3$)$_3$).

HRMS (ESI) *m/z* Calculated for $C_{20}H_{26}N_3O_3$ [M+H] 356.1976; Found 356.1974.

Characterisation information consistent with data previously reported in literature.[25]

N-(Quinolin-8-yl)tetrahydro-2H-thiopyran-4-carboxamide 1,1-dioxide (14)

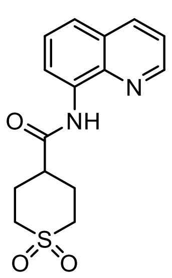

4-DMAP (508 mg, 4.00 mmol, 0.2 equiv) and EDC·HCl (4.20 g, 22.00 mmol, 1.1 equiv) were added to a solution of tetrahydro-2*H*-thiopyran-4-carboxylic acid 1,1-dioxide (3.56 g, 20.00 mmol, 1.0 equiv) and 8-aminoquinoline (3.20 g, 22.00 mmol, 1.1 equiv) in CH_2Cl_2 (40 mL). After stirring at rt for 24 h, the reaction was diluted with CH_2Cl_2 (20 mL), then sat. aq. $NaHCO_3$ (40 mL) was added and the aqueous layer extracted with CH_2Cl_2 (3 x 30 mL). The combined organic extracts were dried over Na_2SO_4, filtered, and the solvent removed under reduced pressure. Purification of the residue by flash column chromatography (40% to 60% acetone/pentane) afforded amide **14** as a dark orange amorphous solid (3.89 g, 64%).

R_f = 0.41 (40% acetone/pentane).

mp = 181–183 °C (from acetone/pentane).

IR (film)/cm^{-1} 3336, 3080, 2855, 1674 (C=O), 1480, 1323, 1282 (S=O), 1122 (S=O), 916, 857, 824, 794.

^{1}H NMR (400 MHz, $CDCl_3$) δ 9.98 (br s, 1 H, NH), 8.81 (dd, *J* = 4.2, 1.6 Hz, 1 H, HC_{Ar}), 8.73 (t, *J* = 4.5 Hz, 1 H, HC_{Ar}), 8.19 (dd, *J* = 8.3, 1.7 Hz, 1 H, HC_{Ar}), 7.55 (d, *J* = 4.5 Hz, 2 H, 2 × HC_{Ar}), 7.49 (dd, *J* = 8.2, 4.2 Hz, 1 H, HC_{Ar}), 3.40 (ddd, *J* = 12.0, 7.7, 4.1 Hz, 2 H, 2 × SC*H*HCH$_2$), 3.09 (ddd, *J* = 13.8, 9.5, 4.0 Hz, 2 H, 2 × SCH*H*CH$_2$), 2.84 (tt, *J* = 8.1, 3.8 Hz, 1 H, CH(C=O)), 2.73–2.25 (m, 4 H, 2 × SCH$_2$C*H$_2$*).

^{13}C NMR (101 MHz, $CDCl_3$) δ 170.9 (C=O amide), 148.3 (C_{Ar}), 138.3 (C_{Ar} quat), 136.5 (C_{Ar}), 133.8 (C_{Ar} quat), 127.9 (C_{Ar} quat), 127.3 (C_{Ar}), 122.1 (C_{Ar}), 121.8 (C_{Ar}), 116.7 (C_{Ar}), 49.8 (*C*H(C=O)), 41.8 (2 × S*C*H$_2$CH$_2$), 27.3 (2 × SCH$_2$*C*H$_2$).

HRMS (ESI) *m/z* Calculated for $C_{15}H_{17}N_2O_3S$ [M+H] 304.3640; Found 304.3632.

4,4-Difluoro-N-(quinolin-8-yl)cyclohexane-1-carboxamide (16)

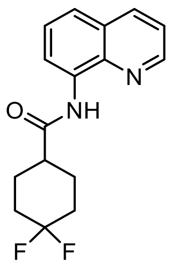

4-DMAP (508 mg, 4.00 mmol, 0.2 equiv) and EDC·HCl (4.20 g, 22.00 mmol, 1.1 equiv) were added to a solution of 4,4-difluorocyclohexane-1-carboxylic acid (3.30 g, 20.00 mmol, 1.0 equiv) and 8-aminoquinoline (3.17 g, 22.00 mmol, 1.1 equiv) in CH_2Cl_2 (50 mL). After stirring at rt for 24 h, the reaction was diluted with CH_2Cl_2 (30 mL), then sat. aq. $NaHCO_3$ (20 mL) was added and the aqueous layer extracted with CH_2Cl_2 (3 x 50 mL). The combined organic extracts were dried over Na_2SO_4, filtered, and the solvent removed under reduced

pressure. Purification of the residue by flash column chromatography (hexane/CH_2Cl_2/Et_2O 9:9:1) afforded amide **16** as a white solid (5.52 g, 19 mmol, 95%).

R_f = 0.31 (hexane/CH_2Cl_2/Et_2O 9:9:1).

mp = 142–145 °C (from hexane/CH_2Cl_2/Et_2O).

IR (film)/cm^{-1} 3348 (NH), 2938, 2871, 1682 (C=O), 1524, 1483, 1377, 1257, 1107, 963, 826.

^{1}H NMR (400 MHz, $CDCl_3$) δ 9.94 (s, 1 H, NH), 8.81 (dd, *J* = 4.2, 1.7 Hz, 1 H, HC_{Ar}), 8.77 (dd, *J* = 6.9, 2.1 Hz, 1 H, HC_{Ar}), 8.18 (dd, *J* = 8.3, 1.7 Hz, 1 H, HC_{Ar}), 7.57–7.50 (m, 2 H, 2 × HC_{Ar}), 7.47 (dd, *J* = 8.3, 4.2 Hz, 1 H, HC_{Ar}), 2.59 (dddd, *J* = 12.2, 10.6, 4.7, 2.7 Hz, 1 H, HC(C=O)), 2.33–2.21 (m, 2 H, 2 × CF_2C*H*H), 2.21–2.11 (m, 2 H, 2 × CF_2CH_2C*H*H), 2.11–1.99 (m, 2 H, 2 × CF_2CH*H*), 1.96–1.78 (m, 2 H, 2 × CF_2CH_2CH*H*).

^{13}C NMR (101 MHz, $CDCl_3$) δ 172.8 (C=O amide), 148.4 (C_{Ar}), 138.5 (C_{Ar} quat), 136.6 (C_{Ar}), 134.4 (C_{Ar} quat), 128.08 (C_{Ar} quat), 127.6 (C_{Ar}), 122.8 (t, J_{CF} = 241.2 Hz, CF_2), 121.8 (C_{Ar}), 121.8 (C_{Ar}), 116.7 (C_{Ar}), 44.3 (*C*H(C=O)), 33.0 (t, J_{CF} = 24.5 Hz, 2 × CF_2*C*H_2), 26.1 (t, J_{CF} = 9.0 Hz, 2 × CF_2CH_2*C*H_2).

^{19}F{^{1}H} NMR (377 MHz, $CDCl_3$) δ –92.90 (d, J_{FF} = 237.5 Hz), –100.55 (d, J_{FF} = 240.5 Hz).

HRMS (ESI) *m/z* Calculated for $C_{16}H_{17}N_2OF_2$ [M+H] 291.1309; Found 291.1317.

Section 2: 2,3-Disubstituted Fragments

Synthesis overview

2,3-Disubstituted Pyrrolidine Fragment Set (FRAG1-6)

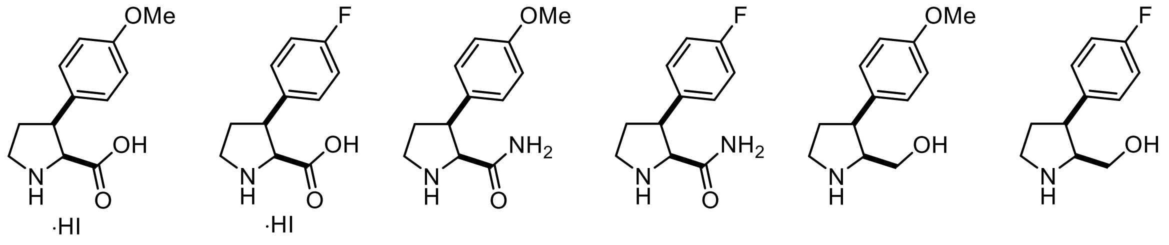

FRAG1 **FRAG2** **FRAG3** **FRAG4** **FRAG5** **FRAG6**

2,3-Disubstituted Piperidine Fragment Set (FRAG 7)

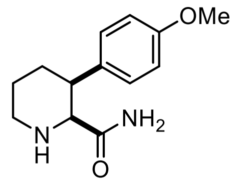

FRAG7

2,3-Disubstituted Fragments Synthetic Routes

CH arylation of aminoquinoline amides was performed as specified giving key intermediates **2a,b and 4a**.

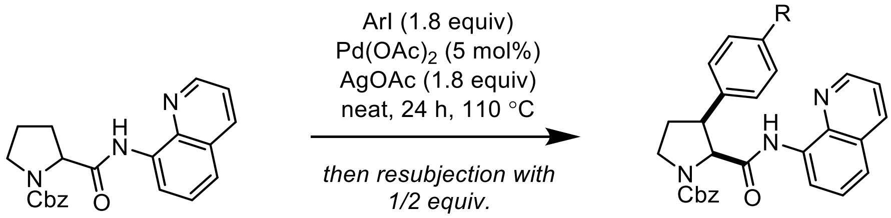

1

R = OMe, **2a** (74%)
R = F, **2b** (82%)

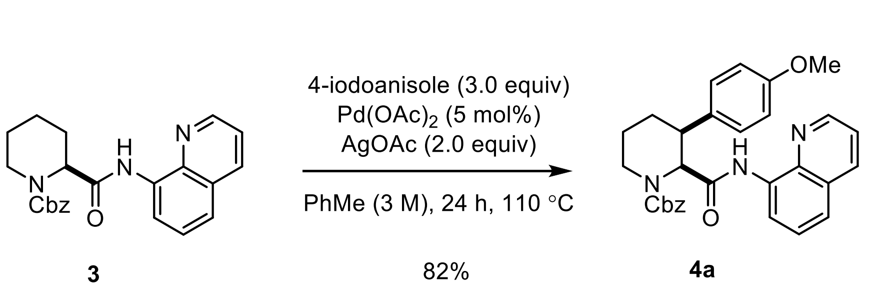

3 82% **4a**

The synthetic route used to access each fragment from the corresponding arylated aminoquinoline amide is given below.

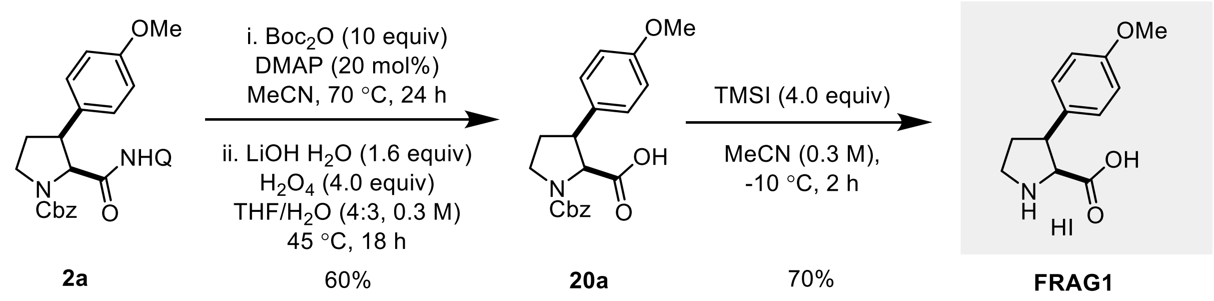

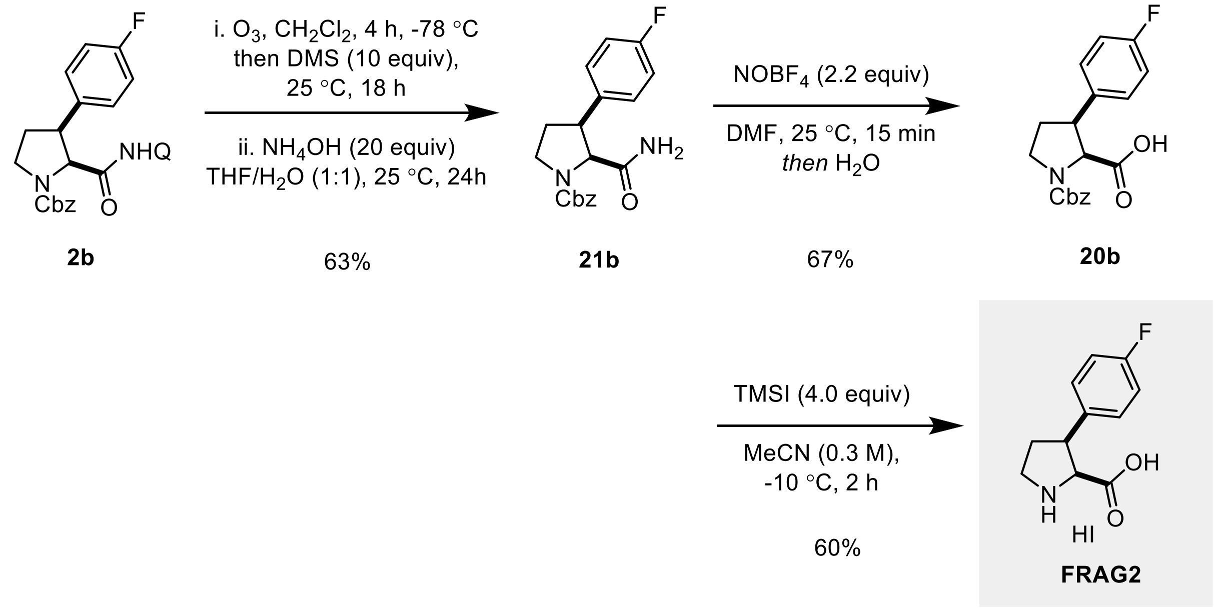

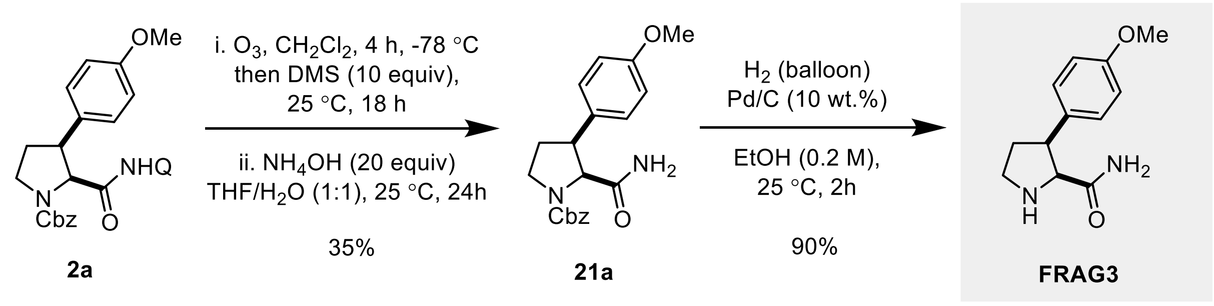

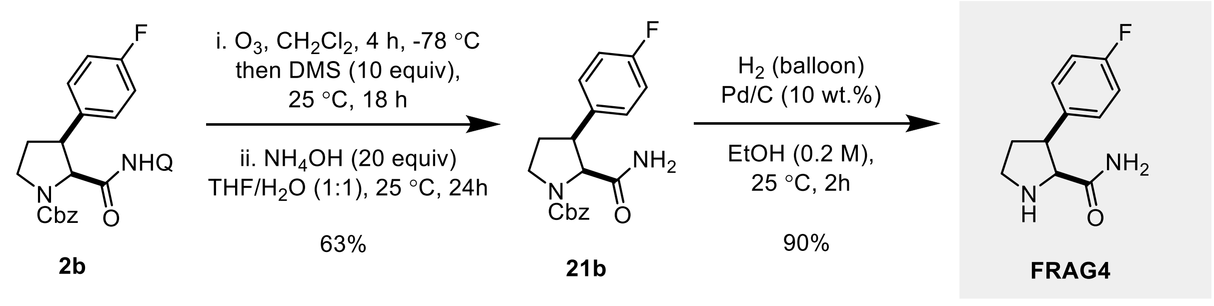

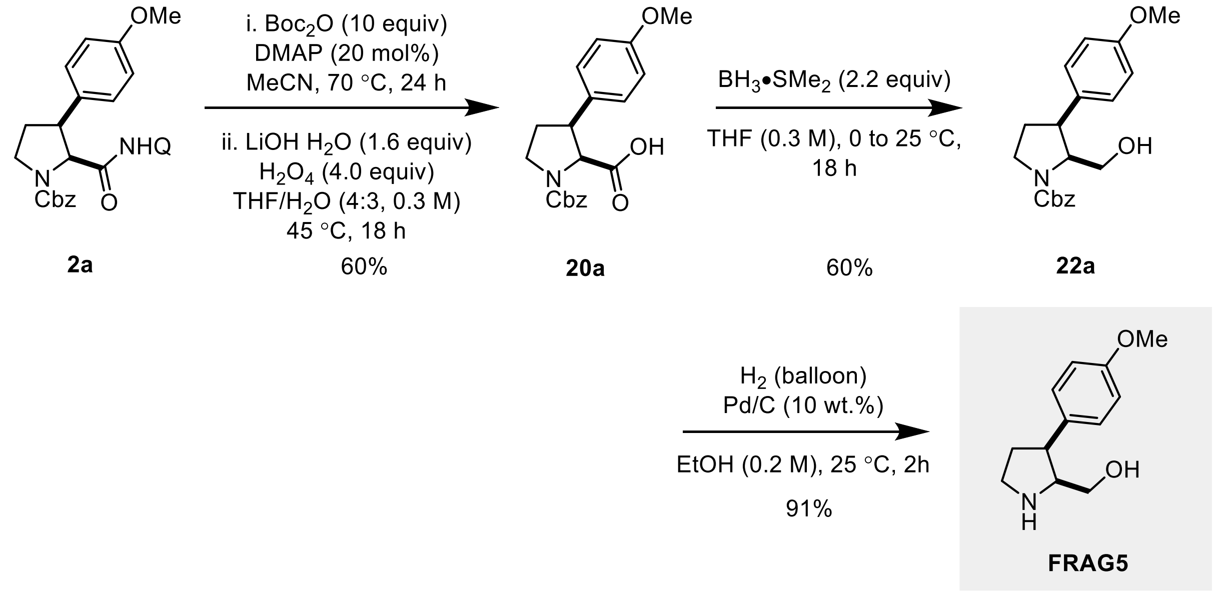
OMe
NHQ
N
Cbz
O
2a
i. Boc2O (10 equiv)
DMAP (20 mol%)
MeCN, 70 °C, 24 h
ii. LiOH H2O (1.6 equiv)
H2O4 (4.0 equiv)
THF/H2O (4:3, 0.3 M)
45 °C, 18 h
60%
OH
20a
BH3•SMe2 (2.2 equiv)
THF (0.3 M), 0 to 25 °C,
18 h
60%
22a
H2 (balloon)
Pd/C (10 wt.%)
EtOH (0.2 M), 25 °C, 2h
91%
N
H
FRAG5

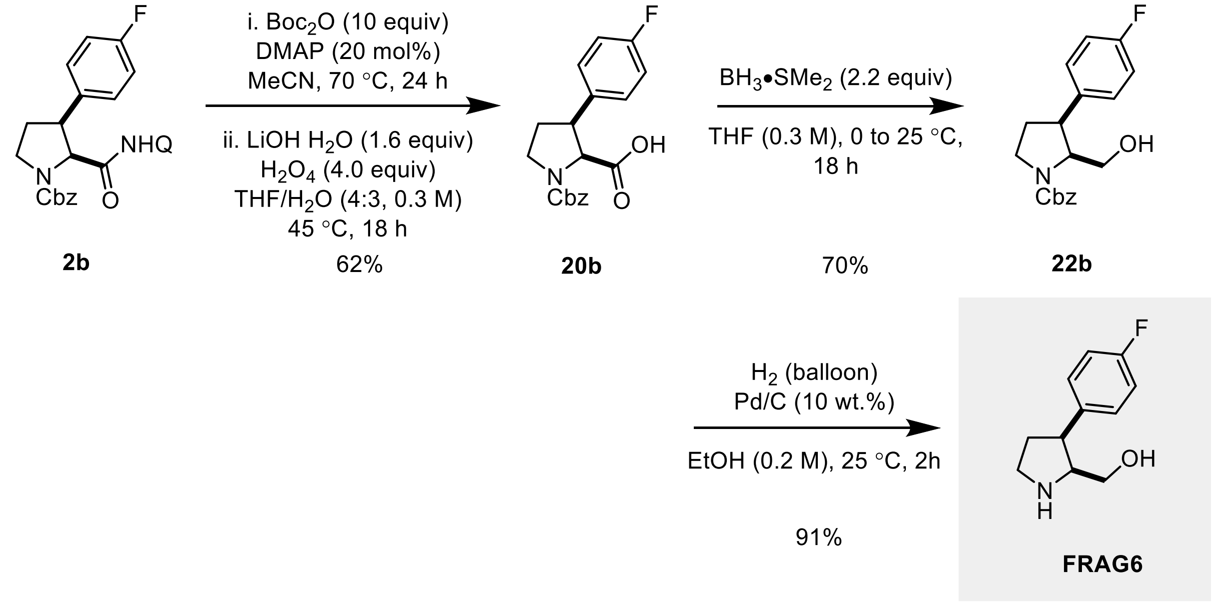
F
NHQ
N
Cbz
O
2b
i. Boc2O (10 equiv)
DMAP (20 mol%)
MeCN, 70 °C, 24 h
ii. LiOH H2O (1.6 equiv)
H2O4 (4.0 equiv)
THF/H2O (4:3, 0.3 M)
45 °C, 18 h
62%
OH
20b
BH3•SMe2 (2.2 equiv)
THF (0.3 M), 0 to 25 °C,
18 h
70%
22b
H2 (balloon)
Pd/C (10 wt.%)
EtOH (0.2 M), 25 °C, 2h
91%
N
H
FRAG6

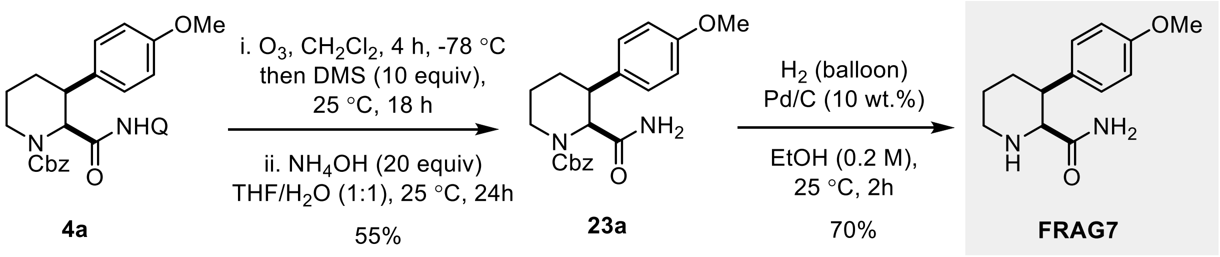
OMe
NHQ
N
Cbz
O
4a
i. O3, CH2Cl2, 4 h, -78 °C
then DMS (10 equiv),
25 °C, 18 h
ii. NH4OH (20 equiv)
THF/H2O (1:1), 25 °C, 24h
55%
NH2
23a
H2 (balloon)
Pd/C (10 wt.%)
EtOH (0.2 M),
25 °C, 2h
70%
N
H
FRAG7

C2-Directed CH Arylations

cis-(±)-Benzyl 3-(4-methoxyphenyl)-2-(quinolin-8-ylcarbamoyl)pyrrolidine-1-carboxylate (2a)

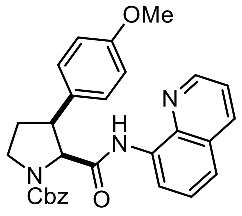

Prepared by adapting a procedure by Bull,[23a] a flame-dried microwave vial (25 mL) was sequentially charged with 4-iodoanisole (1.30 g, 9.00 mmol, 1.8 equiv), AgOAc (1.50 g, 6.00 mmol, 1.2 equiv), $Pd(OAc)_2$ (56 mg, 0.20 mmol, 0.05 equiv), and tert-butyl 2-[(quinolin-8-yl)carbamoyl]pyrrolidine-1-carboxylate (1.88 g, 5.00 mmol, 1.0 equiv) in this order. The vial was sealed, purged with argon, and heated at 110 °C for 24 h. After cooling to rt, EtOAc (50 mL) was added, and the mixture was filtered through Celite, eluting with further EtOAc (2 × 50 mL). The solvent was removed under reduced pressure to give a crude 1:1 mixture of starting material and product (by ^{1}H NMR). The crude mixture was resubjected to the reaction conditions in a round-bottom flask, using 4-iodoanisole (0.75 g, 4.50 mmol, 0.9 equiv), AgOAc (0.75 g, 4.50 mmol, 0.6 equiv), and $Pd(OAc)_2$ (28 mg, 0.13 mmol, 0.03 equiv). After cooling to rt, the mixture was filtered through Celite (EtOAc, 2 × 50 mL) and concentrated under reduced pressure. The same reaction was reconducted a second time parallel, and the crude residues were combined. Purification by flash column chromatography (20–50% EtOAc/hexane) afforded **2a** as an off-white solid (3.50 g, 7.40 mmol, 74%).

R_f 0.33 (35% EtOAc/Hexane);

mp = 166–167 °C;

IR (film)/cm^{-1} 3340 (NH br), 3034, 2952, 2892,1692 (C=O), 1612, 1528, 1489, 1410, 1357, 1325, 1248, 1161, 1035, 982, 825, 795;

^{1}H NMR (400 MHz, $CDCl_3$, mixture of rotamers) δ 9.52 (br s, 1 H, NH), 8.65 (s, 1 H, HC_{Ar}), 8.53 (d, *J* = 6.2 Hz, 1 H, HC_{Ar}), 8.11 (t, *J* = 10.5 Hz, 1 H, HC_{Ar}), 7.51–7.36 (m, 5 H, 5 × HC_{Ar}), 7.19 (t, *J* = 9.1 Hz, 3 H, 3 × HC_{Ar}), 6.97 (t, *J* = 7.3 Hz, 2 H, 2 × HC_{Ar}), 6.62 (t, *J* = 9.9 Hz, 2 H, 2 × HC_{Ar}), 5.29–5.02 (m, 2 H, CH_2Ph), 4.72 (d, *J* = 8.4 Hz, 1 H, CH(C=O)), 4.13–4.03 (m, 1 H, NC*H*H), 3.82–3.59 (m, 2 H, NCH*H* and CHAr), 3.51 (br s, 3 H, OCH_3) 2.83–2.68 (m, 1 H, NCH_2C*H*H), 2.28– 2.21 (m, 1 H, NCH_2CH*H*);

^{13}C NMR (101 MHz, $CDCl_3$, mixture of rotamers) δ 168.7 (C=O amide), 158.7 (C_{Ar} quat), 154.5 (C=O carbamate), 148.0 (C_{Ar}), 138.3 (C_{Ar} quat), 136.8 (C_{Ar} quat), 135.9 (C_{Ar}), 133.8 (C_{Ar} quat), 129.0 (2 × C_{Ar} and 2 × C_{Ar} quat), 128.4 (C_{Ar}), 128.0 (2 × C_{Ar}), 127.6 (2 × C_{Ar}), 127.2 (C_{Ar}), 121.4 (C_{Ar}), 116.3 (C_{Ar}), 113.8 (3 × C_{Ar}), 67.1 (CH_2Ph), 66.4 (*C*H(C=O)), 54.9 (OCH_3), 48.1 and 47.3 (CHAr), 46.6 and 46.3 (NCH_2), 28.7 and 28.1 (NCH_2*C*H_2);

HRMS (ESI) *m/z* Calculated for $C_{29}H_{28}N_3O_4$ [M+H] 482.2080; Found 482.2084.

Characterisation information consistent with data previously reported in literature.[23a]

***cis*-(±)-Benzyl 3-(4-fluorophenyl)-2-(quinolin-8-ylcarbamoyl)pyrrolidine-1-carboxylate (2b)**

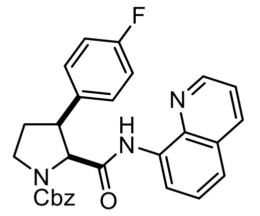

Prepared by adapting a procedure by Bull,[23a] a flame-dried microwave vial (25 mL) was sequentially charged with 1-fluoro-4-iodobenzene (1.11 mL, 9.00 mmol, 1.8 equiv), AgOAc (1.50 g, 6.00 mmol, 1.2 equiv), $Pd(OAc)_2$ (56 mg, 0.20 mmol, 0.05 equiv), and tert-butyl 2-[(quinolin-8-yl)carbamoyl]pyrrolidine-1-carboxylate (1.88 g, 5.00 mmol, 1.0 equiv) in this order. The vial was sealed, purged with argon, and heated at 110 °C for 24 h. After cooling to rt, EtOAc (50 mL) was added and the mixture was filtered through Celite, eluting with further EtOAc (2 × 50 mL). The solvent was removed under reduced pressure to give a crude 1:1 mixture of starting material and product (by ^{1}H NMR). The crude mixture was resubjected to the reaction conditions in a round-bottom flask, using 1-fluoro-4-iodobenzene (0.55 mL, 4.50 mmol, 0.9 equiv), AgOAc (0.75 g, 4.50 mmol, 0.6 equiv), and $Pd(OAc)_2$ (28 mg, 0.13 mmol, 0.03 equiv). After cooling to rt, the mixture was filtered through Celite (EtOAc, 2 × 50 mL) and concentrated under reduced pressure. The same reaction was conducted a second time in parallel, and the crude residues were combined. Purification by flash column chromatography (20–50% EtOAc/hexane) afforded **2b** as a pale yellow solid (3.84 g, 8.80 mmol, 82%).

R_f 0.27 (25% EtOAc/hexane);

mp = 148–150 °C;

IR (film)/cm^{-1} 3335 (NH br), 2952, 2881, 1690 (C=O), 1604, 1523, 1484, 1407, 1355, 1322, 1222, 1160, 1125, 1101, 990, 826, 791, 735;

^{1}H NMR (400 MHz, $CDCl_3$, mixture of rotamers) δ 9.58 (d, *J* = 6.8 Hz, 1 H, NH), 8.65 (d, *J* = 5.3 Hz, 1 H, HC_{Ar}), 8.60–8.47 (m, 1 H, HC_{Ar}), 8.14 (t, *J* = 8.2 Hz, 1 H, HC_{Ar}), 7.51–7.34 (m, 5 H, 5 × HC_{Ar}), 7.30–7.24 (m, 3 H, 3 × HC_{Ar}), 7.17 (d, *J* = 7.5 Hz, 1 H, HC_{Ar}), 6.92 (t, *J* = 7.5 Hz, 1 H, HC_{Ar}), 6.80 (d, *J* = 8.7 Hz, 2 H, 2 × HC_{Ar}), 5.27–5.09 (m, 2 H, CH_2Ph), 4.75 (d, *J* = 8.4 Hz, 1 H, CH(C=O)), 4.13–4.08 (m, 1 H, NC*H*H), 3.88–3.61 (m, 2 H, NCH*H* and CHAr), 2.77 (q, *J* = 11.7 Hz, 1 H, NCH_2C*H*H), 2.32–2.22 (m, 1 H, NCH_2CH*H*);

^{13}C NMR (101 MHz, $CDCl_3$, mixture of rotamers) δ 168.4 (C=O amide), 162.0 (d, J_{CF} = 243.7 Hz, FC_{Ar} quat), 154.5 (C=O carbamate), 148.1 (C_{Ar}), 138.3 (C_{Ar} quat), 136.1 (C_{Ar}), 133.6 (C_{Ar} quat), 132.3 (C_{Ar} quat), 129.6 (d, J_{CF} = 3.0 Hz, F-*p*-C_{Ar} quat), 129.5 (d, J_{CF} = 8.1 Hz, 2 × F-*m*-C_{Ar}), 128.5 (2 × C_{Ar}), 128.0 (C_{Ar} and C_{Ar} quat), 127.7 (2 × C_{Ar}), 127.1 (C_{Ar}), 121.7 (C_{Ar}), 121.5 (C_{Ar}), 116.34 (C_{Ar}), 115.2 (d, J_{CF} = 20.8 Hz, 2 × F-*o*-C_{Ar}), 67.3 (CH_2Ph), 66.3 (*C*H(C=O)), 48.1 and 46.6 (CHAr), 47.2 and 46.3 (NCH_2), 28.8 and 28.1 (NCH_2*C*H_2);

HRMS (ESI) *m/z* Calculated for $C_{28}H_{25}N_3O_3F$ [M+H] 470.1880; Found 470.1863.

Characterisation information consistent with data previously reported in literature.[23a]

cis-(±)-Benzyl 3-(4-methoxyphenyl)-2-(quinolin-8-ylcarbamoyl)piperidine-1-carboxylate (4a)

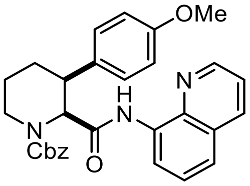

Prepared by adapting a procedure of Bull,[23b] a flame-dried microwave vial (25 mL) was sequentially charged with benzyl 2-(quinolin-8-ylcarbamoyl)piperidine-1-carboxylate (1.56 g, 4.00 mmol, 1.0 equiv), AgOAc (1.34 g, 8.00 mmol, 2.0 equiv), 4-iodoanisole (1.73 g, 12.00 mmol, 3.0 equiv), $Pd(OAc)_2$ (44.8 mg, 0.20 mmol, 0.05 equiv), and PhMe (1.34 mL). The vial was sealed, purged with argon, and heated at 110 °C for 24 h. After cooling to rt, the mixture was diluted with EtOAc (50 mL), filtered through Celite (eluting with EtOAc, 2 × 50 mL), and concentrated under reduced pressure. Purification by flash column chromatography (15–30% EtOAc/hexane) afforded **4a** as a white crystalline solid (1.62 g, 3.28 mmol, 82%);

Rf 0.17 (20% EtOAc/hexane);

mp 158–159 °C;

IR (film)/cm^{-1} 3330 (br, NH), 3034, 2941, 2859, 1684 (C=O), 1521, 1416, 1386, 1323, 1252, 1170, 1036, 970, 827, 790, 731;

^{1}H NMR (400 MHz, $CDCl_3$, mixture of rotamers) δ 9.40 (br s, 1 H, NH), 8.67–8.50 (m, 2 H, 2 × HC_{Ar}), 8.08 (t, *J* = 9.7 Hz, 1 H, HC_{Ar}), 7.51–7.33 (m, 6 H, 6 × HC_{Ar}), 7.32–7.29 (m, 3 H, 3 × HC_{Ar}), 7.17 (dd, *J* = 6.4, 5.5 Hz, 1 H, HC_{Ar}), 6.71 (d, *J* = 8.1 Hz, 2 H, 2 × HC_{Ar}), 5.27–5.07 (m, 3 H, CH_2Ph and CH(C=O)), 4.34–4.18 (m, 1 H, NC*H*H), 3.79–3.63 (m, 1 H, NCH*H*), 3.57 (s, 3 H, OCH_3), 3.25–3.09 (m, 1 H, CHAr), 2.63–2.46 (m, 1 H, C*H*HCHAr), 2.00–1.89 (m, 2 H, CH*H*CHAr and NCH_2C*H*H), 1.73 (td, *J* = 12.5, 11.8, 5.9 Hz, 1 H, NCH_2CH*H*);

^{13}C NMR (101 MHz, $CDCl_3$, mixture of rotamers) δ 168.9 (C=O amide), 158.5 (C_{Ar} quat), 147.9 (C_{Ar}), 136.6 (C_{Ar} quat), 135.8 (C_{Ar} quat), 132.9 (C_{Ar} quat), 128.9 (2 × C_{Ar} and 2 × C_{Ar} quat), 128.5 (C_{Ar}), 127.9 (2 × C_{Ar}), 127.7 (2 × C_{Ar}), 127.0 (C_{Ar}), 121.4 (C_{Ar}), 121.3 (C_{Ar}), 116.3 (C_{Ar}), 114.0 (3 × C_{Ar}), 67.6 (CH_2Ph), 60.7 (*C*H(C=O)), 55.0 (OCH_3), 43.9 (CHAr), 41.4 (NCH_2), 25.6 (NCH_2CH_2), 24.2(*C*H_2CHAr); The carbamate C=O resonance was not detected due to exchange broadening arising from rapid rotameric interconversion about the N–C(O) bond;

HRMS (ESI) *m/z* Calculated for $C_{30}H_{30}N_3O_4$ [M+H] 496.2236; Found 496.2221.

Characterisation information consistent with data previously reported in literature.[23b]

2,3-Disubstituted Fragment Synthesis

cis-(±)-1-((Benzyloxy)carbonyl)-3-(4-methoxyphenyl)pyrrolidine-2-carboxylic acid (20a)

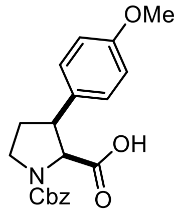

A microwave vial was charged with amide **2a** (483 mg, 1.00 mmol, 1.0 equiv), followed by di-*tert*-butyl dicarbonate (Boc_2O, 1.39 g, 5.00 mmol, 5.0 equiv) and 4-(dimethylamino)pyridine (DMAP, 25.3 mg, 0.20 mmol, 0.2 equiv). The reaction vial was sealed and purged with argon, then MeCN (2 mL) was added by syringe. The reaction tube was then placed in an oil bath and gradually heated to 70 °C, with stirring at this temperature for 24 h. The reaction mixture was then allowed to cool to rt and sat. sol. NH_4Cl (10 mL) and CH_2Cl_2 (10 mL) were added. The phases were separated, and the aqueous layer was extracted with CH_2Cl_2 (3 × 5 mL). The combined organic extracts were dried over Na_2SO_4, filtered and concentrated under reduced pressure. The resulting crude reaction mixture was filtered through a pad of silica (eluting with 30% EtOAc/hexane) to afford the corresponding Boc-protected amide as a pink crystalline solid (468 mg), which was carried to the next step without further purification.

A solution of H_2O_2 (30 wt.% in H_2O, 0.3 mL, 3.10 mmol, 4.0 equiv) in THF (0.8 mL) was added to a solution of $LiOH{\cdot}H_2O$ (54.6 mg, 1.30 mmol, 1.6 equiv) in H_2O (1.2 mL) at 0 °C. The resulting mixture was added dropwise to a solution of Boc protected amide obtained in the previous step (449 mg, 0.80 mmol, 1.0 equiv) in THF (2 mL), at 0 °C. The reaction was then stirred at 45 °C for 24 h. Sat. aq. $Na_2S_2O_3$ (5 mL) and EtOAc (10 mL) were added and the reaction mixture acidified to pH ~ 2 with aq. 1 M HCl. The phases were separated, and the aqueous layer was extracted with EtOAc (3 × 10 mL). The combined organic extracts were dried over Na_2SO_4, filtered and the solvent removed under reduced pressure. Sat. aq. $NaHCO_3$ (approx. 25 mL) was added to the residue and the resulting mixture was washed with EtOAc (2 x 10 mL), discarding the organic layer. The aqueous layer was then acidified to pH ~ 2 with aq. 1 M HCl and extracted with EtOAc (3 x 15 mL). The organics were dried over Na_2SO_4, filtered and the solvent removed under reduced pressure to afford carboxylic acid **20a** as a pale yellow solid (212 mg, 0.60 mmol, 60%);

R_f 0.20 (20% EtOAc/Hexane);

mp = 110–115 °C; IR (film/cm^{-1}) 2989 (OH br), 1725 (C=O), 1700 (C=O), 1645, 1613, 1518, 1417, 1364, 1247, 1201, 1180, 1117, 1028, 834, 747, 695;

^{1}H-NMR (400 MHz, $CDCl_3$, mixture of rotamers) δ 7.45–7.26 (m, 5 H, 5 × HC_{Ar}), 7.19–7.13 (m, 2 H, 2 × HC_{Ar}), 6.84 (d, *J* = 8.8 Hz, 2 H, 2 × HC_{Ar}), 5.30–5.04 (m, 2 H, CH_2Ph), 4.61 and 4.56 (d, *J* = 8.6 Hz, 1 H, CH(C=O)), 3.93–3.67 (m, 1 H, CHAr), 3.78 (s, 3 H, OCH_3), 3.75–3.61 (m, 1 H, NC*H*H), 3.55 (dt, *J* = 11.2, 8.1 Hz, 1 H, NCH*H*), 2.55 (br q, *J* = 11.6 Hz, 1 H, NCH_2C*H*H), 2.17 (dq, *J* = 11.6, 5.4 Hz, 1 H, NCH_2CH*H*);

^{13}C NMR (101 MHz, $CDCl_3$, mixture of rotamers) δ 175.4 and 174.9 (COOH), 159.0 (C_{Ar} quat), 155.0 and 154.2 (C=O carbamate), 136.5 and 136.4 (C_{Ar} quat), 128.9 (C_{Ar} quat), 128.5 (2 × C_{Ar}), 128.1 (2 × C_{Ar}), 128.0 (C_{Ar}), 127.6 (2 × C_{Ar}), 113.9 (2 × C_{Ar}), 67.3 (CH_2Ph), 63.9 and 63.6 (*C*H(C=O)), 55.2 (OCH_3), 47.4 (NCH_2), 46.5 and 46.0 (CHAr), 28.6 and 27.7 (NCH_2*C*H_2);

HRMS (ESI) *m/z* Calculated for $C_{20}H_{22}NO_5$ [M+H] 356.1498; Found 356.1496.

cis-(±)-1-((Benzyloxy)carbonyl)-3-(4-fluorophenyl)pyrrolidine-2-carboxylic acid (20b)

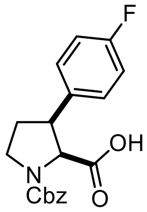

*Synthesis of **20b** using LiOOH:*

A microwave vial was charged with *cis*-arylated amide **2b** (471 mg, 1.00 mmol, 1.0 equiv), followed by di-tert-butyl dicarbonate (1.39 g, 5.00 mmol, 5.0 equiv) and 4-DMAP (25.3 mg, 0.20 mmol, 0.2 equiv). The reaction vial was sealed and purged with argon, then MeCN (2 mL) was added by syringe. The reaction tube was then placed in an oil bath and gradually heated to 70 ºC, with stirring at this temperature for 24 h. The reaction mixture was then allowed to cool to rt and sat. sol. NH_4Cl (10 mL) and CH_2Cl_2 (10 mL) were added. The phases were separated, and the aqueous layer was extracted with CH_2Cl_2 (3 × 5 mL). The combined organic extracts were dried over Na_2SO_4, filtered and concentrated under reduced pressure. The resulting crude material was filtered through a pad of silica (eluting with 30% EtOAc/hexane) to afford the corresponding Boc-protected amide as a pale orange solid (480 mg), which was carried to the next step without further purification. A solution of H_2O_2 (30 wt.% in H_2O, 0.3 mL, 3.10 mmol, 4.0 equiv) in THF (0.8 mL) was added to a solution of $LiOH{\cdot}H_2O$ (54.6 mg, 1.30 mmol, 1.6 equiv) in H_2O (1.2 mL) at 0 °C under argon. The resulting mixture was added dropwise to a solution of Boc protected amide obtained in the previous step (440 mg, 0.80 mmol, 1.0 equiv) in THF (2 mL), at 0 °C under argon. The reaction was then stirred at 45 °C for 24 h. Sat. aq. $Na_2S_2O_3$ (5 mL) and EtOAc (10 mL) were added and the reaction mixture was acidified to pH ~ 2 with aq. 1 M HCl. The phases were separated and the aqueous layer was extracted with EtOAc (3 × 10 mL). The combined organic extracts were dried over Na_2SO_4, filtered and the solvent was removed under reduced pressure. Sat. aq. $NaHCO_3$ (approx. 25 mL) was added to the residue and the resulting mixture was washed with EtOAc (2 x 10 mL), discarding the organic layer. The aqueous layer was then acidified to pH ~ 2 with aq. 1 M HCl and extracted with EtOAc (3 x 15 mL). The combined organic extracts were dried over Na_2SO_4, filtered and the solvent removed under reduced pressure to afford carboxylic acid **20b** as a pale yellow solid (212 mg, 0.62 mmol, 62%).

*Synthesis of **20b** using $NOBF_4$:*

Adapting the literature conditions reported by Kung, [31] $NOBF_4$ (51 mg, 0.44 mmol, 2.2 equiv) was added to a solution of *cis*-arylated amide **21b** (42 mg, 0.20 mmol, 1.0 equiv) in DMF (2 mL) at rt, inducing a colour change from pale yellow to dark green. After stirring at rt for 15 min, the reaction was quenched by dropwise addition of H_2O (1 mL). The reaction mixture was diluted with H_2O (10 mL) and extracted with EtOAc (2 x 10 mL). The combined organics were alternately washed with H_2O (10 mL) and brine (10 mL) twice, then dried over Na_2SO_4 and concentrated under reduced pressure. The residue was purified by flash column chromatography (10 to 30% acetone/pentane) to afford carboxylic acid **20b** as a white powder (46 mg, 0.13 mmol, 67%).

Characterisation data from the two protocols was identical.

R_f 0.18 (20% EtOAc/hexane);

mp = 89–91 °C;

IR (film/cm^{-1}) 2975 (OH br), 1723 (C=O acid), 1698 (C=O carbamate), 1619, 1516, 1413, 1360, 1245, 1204, 1178, 1112, 1026, 835, 748;

^{1}H NMR (400 MHz, $CDCl_3$) δ 8.39 (br s, 1 H, COOH), 7.44–7.25 (m, 5 H, 5 × HC_{Ar}), 7.20 (tdd, *J* = 6.6, 5.1, 2.1 Hz, 2 H, 2 × HC_{Ar}), 6.99 (td, *J* = 8.6, 4.2 Hz, 2 H, 2 × HC_{Ar}), 5.24–5.01 (m, 2 H, CH_2Ph), 4.59 (d, *J* = 8.7 Hz, 1 H, CH(C=O)), 3.90 (dd, *J* = 10.4, 8.7 Hz, 1 H, CHAr), 3.70 (tdd, *J* = 13.0, 8.5, 6.1 Hz, 1 H, NC*H*HCH$_2$), 3.63–3.46 (m, 1 H, NCH*H*CH$_2$), 2.64–2.41 (m, 1 H, NCH$_2$C*H*H), 2.17 (dq, *J* = 12.6, 6.4 Hz, 1 H, NCH$_2$CH*H*);

^{13}C NMR (101 MHz, $CDCl_3$, mixture or rotamers) δ 175.5 and 175.1 (COOH), 162.2 (d, J_{CF} = 246.8 Hz, FC_{Ar} quat), 155.0 and 154.2 (C=O carbamate), 136.4 and 136.3 (C_{Ar} quat), 131.7 and 131.6 (d, J_{CF} = 3.3 Hz, F-*p*-C_{Ar} quat), 129.5 and 129.4 (J_{CF} = 7.8 Hz, 2 × F-*m*-C_{Ar}), 128.5 (2 × C_{Ar}), 128.0 (C_{Ar}), 127.6 (2 × C_{Ar}), 115.4 (d, J_{CF} = 20.0 Hz, 2 × F-*o*-C_{Ar}), 67.4 and 67.3 (CH_2Ph), 63.8 and 63.5 (*C*H(C=O)), 47.3 (NCH_2), 46.3 and 46.0 (CHAr), 28.5 and 27.5 (*C*H$_2$CHAr);

$^{19}F\{^{1}H\}$ NMR (376 MHz, $CDCl_3$) δ –114.58 (s);

HRMS (ESI) *m/z* Calculated for $C_{19}H_{19}FNO_4$ [M+H] 344.1298; Found 344.1295.

cis-(±)-Benzyl 2-carbamoyl-3-(4-methoxyphenyl)pyrrolidine-1-carboxylate (21a)

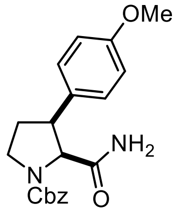

Adapting the literature conditions reported by Maulide, [28b] a solution of the *cis*-arylated pyrrolidine **2a** (720 mg, 1.50 mmol, 1.0 equiv) in anhydrous CH_2Cl_2 (15 mL) was cooled to –78 °C and a stream of ozone was passed through until a dark green coloration occurred (approx. 4 h). Upon passing a stream of oxygen, decolorisation to pale yellow occurred and the reaction was immediately quenched by addition of dimethylsulfide (DMS, 0.4 mL, 15.00 mmol, 10.0 equiv). The mixture was stirred at rt for 18 h, concentrated under reduced pressure and the residue dissolved in NH_4OH (aq. 28%)/THF (8 mL, 1:1 v/v). After stirring at rt for 20 h, the reaction mixture was diluted with CH_2Cl_2 (15 mL) and washed with sat. aq. NH_4Cl (10 mL). The organic layer was separated and the aqueous layer was extracted with CH_2Cl_2 (3 × 15 mL). The combined organic layers were dried over Na_2SO_4 and the solvent was removed under reduced pressure. The crude material was purified by flash column chromatography (10% to 40% EtOAc/hexane) afforded amide **21a** as a yellow solid (190 mg, 0.50 mmol, 35%).

R_f 0.21 (40% EtOAc/Hexane);

IR (film/cm^{-1}) 3345 (br NH), 3210 (br NH), 3033, 2958, 2838, 1698 (C=O), 1611, 1518, 1415, 1363, 1250, 1179, 1130, 838, 735;

^{1}H NMR (400 MHz, $CDCl_3$, mixture of rotamers) δ 7.40–7.34 (m, 5 H, 5 × HC_{Ar}), 7.21 (d, *J* = 8.1 Hz, 2 H, 2 × HC_{Ar}), 6.92–6.82 (m, 2 H, 2 × HC_{Ar}), 5.30–5.11 (m, 2 H, CH_2Ph and N*H*H amide), 4.99 (br s, 1 H, NH*H* amide), 4.44–4.30 (m, 1 H, CH(C=O)), 3.92 (br s, 1 H, NC*H*H), 3.79 (s, 3 H, OCH_3), 3.63–3.54 (m, 2 H, CHAr and NCH*H*), 2.65 (br s, 1 H, NCH$_2$C*H*H), 2.13 (dt, *J* = 12.4, 6.3 Hz, 1 H, NCH$_2$CH*H*);

^{13}C NMR (101 MHz, $CDCl_3$, mixture of rotamers) δ 172.2 (C=O amide), 129.1 (2 × C_{Ar}), 128.5 (2 × C_{Ar}), 128.0 (C_{Ar}), 127.9 (2 × C_{Ar}), 113.9 (2 × C_{Ar}), 67.2 (CH_2Ph), 64.7 (*C*H(C=O)), 55.2 (OCH_3); Not detected: C=O carbamate, 3 × C_{Ar} quat, CHAr and 2 × CH_2 due to exchange broadening arising from rapid rotameric interconversion about the N–C(O) bond;

HRMS (ESI) *m/z* Calculated for $C_{20}H_{23}N_2O_4$ [M+H] 355.1658; Found 355.1655.

cis-(±)-Benzyl 2-carbamoyl-3-(4-fluorophenyl)pyrrolidine-1-carboxylate (21b)

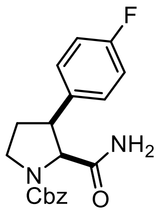

Adapting the literature conditions reported by Maulide,[28b] a solution of the *cis*-arylated pyrrolidine **2b** (705 mg, 1.50 mmol, 1.0 equiv) in anhydrous CH_2Cl_2 (15 mL) was cooled to –78 °C and a stream of ozone was passed through until a dark green coloration occurred (approx. 4 h). Upon passing a stream of oxygen, decolorisation to pale yellow occurred and the reaction was immediately quenched by addition of dimethylsulfide (DMS, 0.4 mL, 15.00 mmol, 10.0 equiv). The mixture was stirred at rt for 18 h, concentrated under reduced pressure and the residue dissolved in NH_4OH (aq. 28%)/THF (8 mL, 1:1 v/v). After stirring at rt for 20 h, the reaction mixture was diluted with CH_2Cl_2 (15 mL) and washed with sat. aq. NH_4Cl (10 mL). The organic layer was separated and the aqueous layer was extracted with CH_2Cl_2 (3 × 15 mL). The combined organic layers were dried over Na_2SO_4 and the solvent was removed under reduced pressure. The crude material was purified by flash column chromatography (10% to 40% EtOAc/hexane) afforded amide **21b** as a yellow solid (320 mg, 0.90 mmol, 63%).

R_f 0.29 (40% EtOAc/hexane);

mp = 105–110 °C;

IR (film)/cm^{-1} 3332 (br NH), 3194 (br NH), 2952, 2885, 1681 (C=O), 1608, 1513, 1416, 1360, 1226, 1126, 951, 832;

^{1}H NMR (400 MHz, $CDCl_3$, mixture of rotamers) δ 7.38–7.28 (m, 5 H, 5 × HC_{Ar}), 7.27–7.25 (m, 2 H, 2 × HC_{Ar}), 7.05 (dd, *J* = 8.5, 3.1 Hz, 2 H, 2 × HC_{Ar}), 5.57 (br s, 1 H, N*H*H amide), 5.23–5.11 (m, 3 H, NH*H* amide and CH_2Ph), 4.45 (br s, 1 H, CH(C=O)), 3.94 (br t, *J* = 9.7 Hz, 1 H, NC*H*H), 3.71–3.50 (m, 2 H, CHAr and NCH*H*), 2.74 (t, *J* = 13.3 Hz, 1 H, NCH_2C*H*H), 2.16 (dt, *J* = 11.9, 6.0 Hz, 1 H, NCH_2CH*H*);

^{13}C NMR (101 MHz, $CDCl_3$) δ 172.1 (C=O amide), 162.1 (d, J_{CF} = 245.8 Hz, FC_{Ar} quat), 155.0 (C=O carbamate), 136.4 (C_{Ar} quat), 132.4 (d, J_{CF} = 3.0 Hz, F-*p*-C_{Ar} quat), 129.6 (d, J_{CF} = 8.1 Hz, 2 × F-*m*-C_{Ar}), 128.5 (2 × C_{Ar}), 128.1 (C_{Ar}), 127.8 (2 × C_{Ar}), 115.4 (d, J_{CF} = 20.0 Hz, 2 × F-*o*-C_{Ar}); 67.2 (OCH_2), 64.4 (*C*H(C=O)), 46.5 (CHAr), 46.1 (NCH_2), 28.6 (NCH_2*C*H_2);

^{19}F{^{1}H} NMR (376 MHz, $CDCl_3$) δ –114.98 (s);

HRMS (ESI) *m/z* Calculated for $C_{19}H_{20}N_2O_3F$ [M+H] 343.1458; Found 343.1449.

cis-(±)-Benzyl 2-(hydroxymethyl)-3-(4-methoxyphenyl)pyrrolidine-1-carboxylate (22a)

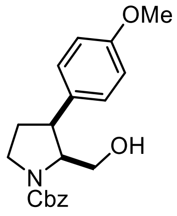

$BH_3 \cdot SMe_2$ (0.6 mL, 1.20 mmol, 2.2 equiv) was added dropwise to a solution of carboxylic acid **20a** (200 mg, 0.56 mmol, 1.0 equiv) in THF (1.7 mL) at 0 °C. The reaction mixture was stirred at 0 °C for 3 h, then at rt for 18 h. The mixture was then cooled again to 0 °C and quenched by dropwise addition of distilled H_2O (3 mL). After dilution with EtOAc (5 mL), the layers were separated and the organic layer washed with sat. aq. NaCl (10 mL), $NaHCO_3$ (10 mL), H_2O (5 mL) and again sat. aq. NaCl (5 mL). The organic layer was dried over Na_2SO_4 and concentrated under reduced pressure. The crude product was purified by column chromatography (10% to 35% EtOAc/hexane) to afford alcohol **22a** as a yellow oil (117 mg, 0.34 mmol, 60%).

R_f 0.59 (35% EtOAc/hexane);

IR (film)/cm^1 3324 (br, OH), 2937, 2837, 1610 (C=O), 1513, 1461, 1409, 1249, 1182, 1036, 835;

^{1}H NMR (400 MHz, $CDCl_3$) δ 7.42–7.36 (m, 5 H, 5 × HC_{Ar}), 7.21 (d, *J* = 8.1 Hz, 2 H, 2 × HC_{Ar}), 6.90 (d, *J* = 8.1 Hz, 2 H, 2 × HC_{Ar}), 5.30–5.11 (m, 2 H, CH_2Ph), 4.30 (t, *J* = 4.0 Hz, 1 H, CH_2OH), 3.82 (s, 3 H, OCH_3), 3.77–3.72 (m, 1 H, C*H*HOH), 3.60–3.50 (m, 2 H, NC*H*H and C*H*CH_2OH), 3.46–3.35 (m, 2 H, CHAr and NCH*H*), 3.27 (t, *J* = 5.5 Hz, 1 H, CH*H*OH), 2.47–2.32 (m, 1 H, NCH_2C*H*H), 2.16 (dt, *J* = 12.6, 6.5 Hz, 1 H, NCH_2CH*H*);

^{13}C NMR (101 MHz, $CDCl_3$) δ 136.5 (C_{Ar} quat), 128.9 (2 × C_{Ar} and C_{Ar} quat), 128.5 (3 × C_{Ar}), 128.0 (2 × C_{Ar}), 114.0 (2 × C_{Ar}), 67.3 (CH_2Ph), 64.0 (*C*HCH_2OH), 63.1 (CH_2OH), 55.3 (OCH_3), 46.0 (NCH_2), 45.4 (CHAr), 28.3 (NCH_2CH_2); Not detected: C=O carbamate and one C_{Ar} quat (Cbz phenyl) due to exchange broadening arising from rapid rotameric interconversion about the N–C(O) bond;

HRMS (ESI) *m/z* Calculated for $C_{20}H_{24}NO_4$ [M+H] 342.1705; Found 342.1699.

cis-(±)-Benzyl 3-(4-fluorophenyl)-2-(hydroxymethyl)pyrrolidine-1-carboxylate (22b)

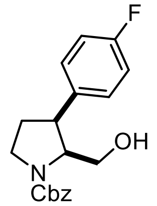

$BH_3 \cdot SMe_2$ (0.55 mL, 1.10 mmol, 2.2 equiv) was added dropwise to a solution of carboxylic acid **20b** (170 mg, 0.50 mmol, 1.0 equiv) in THF (1.6 mL) at 0 °C. The reaction mixture was stirred at 0 °C for 3 h, then at rt for 18 h. The mixture was then cooled again to 0 °C and quenched by dropwise addition of distilled H_2O (3 mL). After dilution with EtOAc (5 mL), the layers were separated and the organic layer washed with sat. aq. NaCl (10 mL), $NaHCO_3$ (10 mL), H_2O (5 mL) and again with sat. aq. NaCl (5 mL). The organic layer was dried over Na_2SO_4 and concentrated under reduced pressure. The crude product was purified by column chromatography (10% to 35% EtOAc/Hexane) to afford alcohol **22b** as a pale yellow oil (113 mg, 0.35 mmol, 70%).

R_f 0.66 (35% EtOAc/Hexane);

IR (film)/cm1 3302 (br, OH), 2959, 2835, 1603 (C=O), 1513, 1461, 1409, 1226, 1163, 1059, 839, 805;

^{1}H NMR (400 MHz, DMSO-d_6) δ 7.42–7.25 (m, 7 H, 7 × HC_{Ar}), 7.12–7.04 (m, 2 H, 2 × HC_{Ar}), 5.19–5.03 (m, 2 H, CH_2Ph), 4.08–3.97 (m, 2 H, CH_2OH), 3.60 (ddd, *J* = 10.5, 9.1, 1.5 Hz, 1 H, C*H*CH_2OH), 3.57–3.43 (m, 2 H, CHAr and NC*H*H), 3.10 (ddd, *J* = 11.3, 5.2, 3.1 Hz, 1 H, NCH*H*), 2.52–2.49 (m, 1 H NCH_2C*H*H), 2.09–1.99 (m, 1 H, NCH_2CH*H*);

^{13}C NMR (101 MHz, DMSO-d_6) δ 160.6 (d, J_{CF} = 246.9 Hz, FC_{Ar} quat), 153.8 (C=O carbamate), 136.8 (C_{Ar} quat), 134.4 (d, J_{CF} = 3.0 Hz, F-*p*-C_{Ar} quat), 129.7 (d, J_{CF} = 8.1 Hz, 2 × F-*m*-C_{Ar}), 127.7 (2 × C_{Ar}), 127.0 (C_{Ar}), 126.8 (2 × C_{Ar}), 114.1 (d, J_{CF} = 20.4 Hz, 2 × F-*o*-C_{Ar}), 65.3 (CH_2Ph), 61.4 (*C*HCH_2OH), 59.1 (CH_2OH), 45.2 (NCH_2), 30.0 (CHAr), 27.5 (NCH_2*C*H_2);

^{19}F{^{1}H} NMR (376 MHz, $CDCl_3$) δ –117.05 (s);

HRMS (ESI) *m/z* Calculated for $C_{19}H_{21}FNO_3$ [M+H] 330.1505; Found 330.1501.

cis-(±)-2-Carboxy-3-(4-methoxyphenyl)pyrrolidin-1-ium iodide (FRAG1)

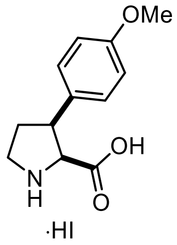

Adapting the literature conditions reported by Lyster,[41] TMSI (0.21 mL, 1.60 mmol, 4.0 equiv) was added dropwise over 15 min to a suspension of acid **20a** (137 mg, 0.40 mmol, 1.0 equiv) in MeCN (1.4 mL) at –10 °C. The reaction mixture was stirred at –10 °C for 30 min and at –5 °C for a further 1.5 h and then quenched by the addition of MeOH (0.2 mL). The volatiles were removed under reduced pressure. To remove the benzyl iodide by-product, the residue was dissolved in Et_2O (10 mL) and stirred for 10 min, followed by decanting the ethereal layer. This operation was repeated until benzyl iodide was removed (assessed by TLC; R_f of benzyl iodide: 0.90 in 30% EtOAc/hexane). Removal of residual Et_2O solvent under reduced pressure afforded **FRAG1** as an orange crystalline solid (94 mg, 0.28 mmol, 70%).

R_f 0.08 (10% MeOH/CH_2Cl_2);

mp not observed up to 260 °C;

IR (film)/cm1 3001 (br, OH), 2833, 1610 (C=O), 1513, 1394, 1327, 1249, 1182, 1029, 835;

^{1}H NMR (400 MHz, CD_3OD) δ 7.23 (d, *J* = 8.8 Hz, 2 H, 2 × HC_{Ar}), 6.89 (d, *J* = 8.8 Hz, 2 H, 2 × HC_{Ar}), 4.64 (d, *J* = 9.1 Hz, 1 H, CH(C=O)), 3.99 (td, *J* = 9.1, 7.8 Hz, 1 H, CHAr), 3.77 (s, 3 H, OCH_3), 3.73 (dt, *J* = 7.9, 4.0 Hz, 1 H, NC*H*HCH_2), 3.50 (ddd, *J* = 11.6, 9.1, 7.9 Hz, 1 H, NCH*H*CH_2), 2.54 (dtd, *J* = 13.3, 7.8, 4.0 Hz, 1 H, NCH_2C*H*H), 2.42–2.29 (m, 1 H, NCH_2CH*H*);

^{13}C NMR (101 MHz, CD_3OD) δ 168.5 (COOH), 159.4 (C_{Ar} quat), 129.0 (2 × C_{Ar}), 128.6 (C_{Ar} quat), 113.6 (2 × C_{Ar}), 63.6 (*C*H(C=O)), 54.4 (OCH_3), 45.3 (CHAr), 45.1 (NCH_2), 30.1 (NCH_2*C*H_2);

HRMS (ESI) *m/z* Calculated for $C_{12}H_{16}NO_3$ $[M-I]^+$ 222.1130; Found 222.1125.

cis-(±)-2-Carboxy-3-(4-fluorophenyl)pyrrolidin-1-ium iodide (FRAG2)

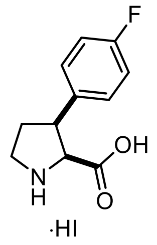

Adapting the literature conditions reported by Lyster,[41] TMSI (0.27 mL, 2.00 mmol, 4.0 equiv) was added dropwise over 15 min to a suspension of acid **20b** (172 mg, 0.50 mmol, 1.0 equiv) in MeCN (1.8 mL) at –10 °C. The reaction mixture was stirred at –10 °C for 30 min and at –5 °C for a further 1.5 h and then quenched by the addition of MeOH (0.4 mL). The volatiles were removed under reduced pressure. To remove the benzyl iodide by-product, the residue was dissolved in Et_2O (10 mL) and stirred for 10 min, followed by decanting the ethereal layer. This operation was repeated until benzyl iodide was removed (assessed by TLC; R_f of benzyl iodide: 0.90 in 30% EtOAc/hexane). Removal of residual Et_2O solvent under reduced pressure afforded **FRAG2** as a dark orange solid (110 mg, 0.30 mmol, 60%, containing 7 mg MeCN (6% by mass) – remaining MeCN could not be removed by available techniques).

R_f 0.05 (10% MeOH/CH_2Cl_2);

mp = 254–255 °C;

IR (film)/cm^{-1} 3377 (br, NH), 2822, 1722 (C=O), 1603, 1450, 1420, 1513, 1371, 1304, 1223, 1107, 839;

^{1}H NMR (400 MHz, CD_3OD) δ 7.44–7.23 (m, 2 H, 2 × HC_{Ar}), 7.21–7.01 (m, 2 H, 2 × HC_{Ar}), 4.65 (d, *J* = 9.2 Hz, 1 H, CH(C=O)), 4.03 (dt, *J* = 9.2, 8.6 Hz, 1 H, CHAr), 3.76 (ddd, *J* = 11.8, 8.2, 3.8 Hz, 1 H, NC*H*H), 3.48 (ddd, *J* = 11.8, 8.8, 7.4 Hz, 1 H, NCH*H*), 2.57 (dtd, *J* = 13.4, 7.8, 3.8 Hz, 1 H, NCH_2C*H*H), 2.38 (dtd, *J* = 13.4, 9.3, 8.6 Hz, 1 H, NCH_2CH*H*);

^{13}C NMR (101 MHz, CD_3OD) δ 168.3 (COOH), 160.8 (d, J_{CF} = 274.1 Hz, FC_{Ar} quat), 133.1 (d, J_{CF} = 3.1 Hz, F-*p*-C_{Ar} quat), 129.8 (d, J_{CF} = 8.1 Hz, 2 × F-*m*-C_{Ar}), 115.1 (d, J_{CF} = 20.8 Hz, 2 × F-*o*-C_{Ar}), 61.6 (*C*H(C=O)), 43.5 (CHAr), 43.3 (NCH_2), 26.9 (NCH_2*C*H_2); The quaternary carbon signals are weaker in intensity than expected, as the limited sample available for characterisation was partially insoluble/viscous, affecting spin-lattice relaxation;

^{19}F{^{1}H} NMR (376 MHz, $CDCl_3$) δ –117.71 (s);

HRMS (ESI) *m/z* Calculated for $C_{11}H_{13}FNO_2$ [M+H] 210.0930; Found 210.0925.

cis-(±)-3-(4-Methoxyphenyl)pyrrolidine-2-carboxamide (FRAG3)

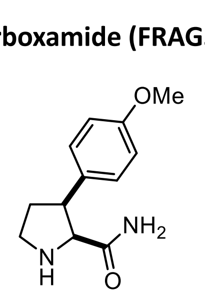

Pd/C (80 mg, 10 wt. %) was added to a 5 mL microwave vial containing a solution a solution of **21a** (147 mg, 0.4 mmol, 1.0 equiv) in EtOH (3 mL, 0.2 M). The vial was evacuated and backfilled with Ar three times, followed by evacuation and backfilling with H_2 (balloon). The resulting solution was stirred vigorously at rt for

2 h, then filtered through Celite, washing with EtOH (20 mL). The solvent was removed under reduced pressure to afford **FRAG3** as a white solid (80 mg, 0.36 mmol, 90%).

R_f 0.12 (10% MeOH/CH_2Cl_2);

mp = 151–154 °C;

IR (film)/cm^{-1} 3425 (br NH), 3325 (br NH), 3183, 2960, 2874, 1670 (C=O), 1513, 1245, 1036, 831;

^{1}H NMR (400 MHz, $CDCl_3$) δ 7.24–7.15 (m, 2 H, 2 × HC_{Ar}), 6.89–6.79 (m, 2 H, 2 × HC_{Ar}), 6.49 (br s, 1 H, N*H*H amide), 5.03 (br s, 1 H, NH*H* amide), 3.95 (d, *J* = 8.3 Hz, 1 H, CH(C=O)), 3.80 (s, 3 H, OCH_3), 3.62 (dt, *J* = 8.3, 7.7 Hz, 1 H, CHAr), 3.39 (ddd, *J* = 9.7, 7.7, 4.6 Hz, 1 H, NC*H*H), 3.16 (ddd, *J* = 9.7, 8.1, 7.5 Hz, 1 H, NCH*H*), 2.26 (dtd, *J* = 12.6, 7.7, 4.6 Hz, 1 H, NCH_2C*H*H), 2.08 (dtd, *J* = 12.6, 7.7, 6.6 Hz, 1 H, NCH_2CH*H*);

^{13}C NMR (101 MHz, $CDCl_3$) δ 175.3 (C=O amide), 158.3 (C_{Ar} quat), 132.9 (C_{Ar} quat), 129.2 (2 × C_{Ar}), 113.5 (2 × C_{Ar}), 66.1 (*C*H(C=O)), 55.2 (OCH_3), 47.2 (CHAr), 46.0 (NCH_2), 32.4 (NCH_2*C*H_2);

HRMS (ESI) *m/z* Calculated for $C_{12}H_{17}N_2O_2$ [M+H] 221.1290; Found 221.1286.

cis-(±)-3-(4-Fluorophenyl)pyrrolidine-2-carboxamide (FRAG4)

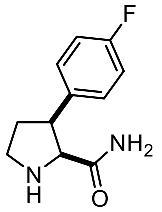

Pd/C (30 mg, 10 wt. %) was added to a 5 mL microwave vial containing a solution a solution of **21b** (110 mg, 0.3 mmol, 1.0 equiv) in EtOH (1.5 mL, 0.2 M). The vial was evacuated and backfilled with Ar three times, followed by evacuation and backfilling with H_2 (balloon). The resulting solution was stirred vigorously at rt for 2 h, then filtered through Celite, washing with EtOH (20 mL). The solvent was removed under reduced pressure and the crude residue was purified by flash column chromatography (0% to 10% MeOH/CH_2Cl_2) to afford **FRAG4** as a pale yellow solid (54 mg, 0.26 mmol, 90%).

R_f 0.10 (10% MeOH/CH_2Cl_2);

mp = 131–134 °C;

IR (film)/cm^{-1} 3345 (br NH), 3034, 2960, 1700 (C=O), 1607, 1513, 1416, 1357, 1226, 1130, 909, 834, 737, 730, 700;

^{1}H NMR (400 MHz, $CDCl_3$) δ 7.23 (dd, *J* = 8.5, 5.4 Hz, 2 H, 2 × HC_{Ar}), 6.98 (t, *J* = 8.5 Hz, 2 H, 2 × HC_{Ar}), 6.62 (br s, 1 H, N*H*H amide), 5.18 (br s, 1 H, NH*H* amide), 3.98 (d, *J* = 8.4 Hz, 1 H, CH(C=O)), 3.64 (ddd, *J* = 8.4, 7.5, 7.4 Hz, 1 H, CHAr), 3.39 (ddd, *J* = 9.7, 7.6, 4.6 Hz, 1 H, NC*H*H), 3.24–3.13 (m, 1 H, NCH*H*), 2.29 (dtd, *J* = 12.6, 7.9, 4.6 Hz, 1 H, NCH_2C*H*H), 2.07 (dq, *J* = 14.0, 7.3 Hz, 1 H, NCH_2CH*H*);

^{13}C NMR (101 MHz, $CDCl_3$) δ 175.0 (C=O amide), 161.7 (d, J_{CF} = 242.8 Hz, FC_{Ar} quat), 136.7 (d, J_{CF} = 3.1 Hz, F-*p*-C_{Ar} quat), 129.7 (d, J_{CF} = 8.1 Hz, 2 × F-*m*-C_{Ar}), 114.9 (d, J_{CF} = 20.8 Hz, 2 × F-*o*-C_{Ar}), 66.0 (*C*H(C=O)), 47.1 (CHAr), 45.9 (NCH_2), 32.4 (NCH_2*C*H_2);

^{19}F{^{1}H} NMR (376 MHz, $CDCl_3$) δ –116.55 (s);

HRMS (ESI) *m/z* Calculated for $C_{11}H_{14}N_2OF$ [M+H] 209.1090; Found 209.1087.

cis-(±)-3-((4-Methoxyphenyl)pyrrolidin-2-yl)methanol (FRAG5)

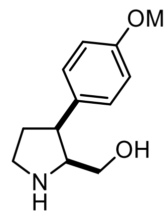

Pd/C (80 mg, 10 wt. %) was added to a 5 mL microwave vial containing a solution a solution of **22a** (117 mg, 0.30 mmol, 1.0 equiv) in EtOH (3 mL, 0.2 M). The vial was evacuated and backfilled with Ar three times, followed by evacuation and backfilling with H_2 (balloon). The resulting solution was stirred vigorously at rt for 2 h, then filtered through Celite, washing with EtOH (20 mL). The solvent was removed under reduced pressure to afford **FRAG5** as a white foam (64 mg, 0.31 mmol, 91%).

R_f 0.11 (10% MeOH/CH_2Cl_2);

mp = 75–80 °C;

IR (film)/cm^{-1} 3424 (br OH), 2937, 2837, 1513, 1409, 1249, 1182, 1036, 835;

^{1}H NMR (400 MHz, $CDCl_3$) δ 7.18–7.16 (m, 2 H, 2 × HC_{Ar}), 6.89–6.86 (m, 2 H, 2 × HC_{Ar}), 3.82 (s, 3 H, OCH_3), 3.51 (td, *J* = 7.8, 4.8 Hz, 1 H, C*H*HOH), 3.40 (dt, *J* = 10.0, 7.8 Hz, 1 H, CH*H*OH), 3.24 (ddd, *J* = 11.0, 7.8, 3.3 Hz, 1 H, C*H*CH_2OH), 3.18–3.10 (m, 2 H, NC*H*HCH_2 and CHAr), 3.04 (dd, *J* = 10.9, 8.5 Hz, 1 H, NCH*H*CH_2), 2.20–2.11 (m, 2 H, $NCH_2C\mathit{H_2}$);

^{13}C NMR (101 MHz, $CDCl_3$) δ 154.3 (C_{Ar} quat), 127.6 (C_{Ar} quat), 125.1 (2 × C_{Ar}), 109.8 (2 × C_{Ar}), 58.4 (CH_2OH), 57.8 (*C*HCH_2OH), 51.3 (OCH_3), 42.3 (NCH_2), 41.3 (CHAr), 26.6 ($NCH_2\mathit{C}H_2$);

HRMS (ESI) *m/z* Calculated for $C_{12}H_{18}NO_2$ [M+H] 208.2810; Found 208.2806.

cis-(±)-3-((4-Fluorophenyl)pyrrolidin-2-yl)methanol (FRAG6)

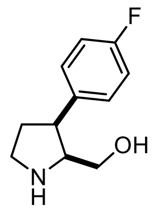

Pd/C (80 mg, 10 wt. %) was added to a 5 mL microwave vial containing a solution a solution of **22b** (113 mg, 0.40 mmol, 1.0 equiv) in EtOH (3 mL, 0.2 M). The vial was evacuated and backfilled with Ar three times, followed by evacuation and backfilling with H_2 (balloon). The resulting solution was stirred vigorously at rt for 2 h, then filtered through Celite, washing with EtOH (20 mL). The solvent was removed under reduced pressure to afford **FRAG6** as a dense dark yellow oil (67 mg, 0.34 mmol, 85%).

R_f 0.12 (10% MeOH/CH_2Cl_2);

IR (film)/cm^{-1} 3425 (OH br), 2969, 2834, 1513, 1409, 1226, 1163, 1059, 839;

^{1}H NMR (400 MHz, $CDCl_3$) δ 7.22–7.17 (m, 2 H, 2 × HC_{Ar}), 7.03–6.97 (m, 2 H, 2 × HC_{Ar}), 4.13 (br s, 1 H, NH), 3.52 (td, *J* = 7.9, 4.0 Hz, 1 H, C*H*HOH), 3.41 (dt, *J* = 9.7, 7.9 Hz, 1 H, CH*H*OH), 3.27 (ddd, *J* = 11.0, 7.9, 4.0 Hz, 1 H, C*H*CH_2OH), 3.19–3.08 (m, 2 H, NC*H*HCH_2 and CHAr), 3.02 (td, *J* = 11.1, 8.6 Hz, 1 H, NCH*H*CH_2), 2.25–2.10 (m, 2 H, NCH_2C*H_2*);

^{13}C NMR (101 MHz, $CDCl_3$) δ 161.8 (d, *J* = 266.8 Hz, FC_{Ar} quat), 134.2 (d, J_{CF} = 3.2 Hz, F-*p*-C_{Ar} quat), 129.6 (d, J_{CF} = 7.8 Hz, 2 × F-*m*-C_{Ar}), 115.3 (d, J_{CF} = 21.5 Hz, 2 × F-*o*-C_{Ar}), 62.6 (CH_2OH), 61.0 (*C*HCH_2OH), 45.8 (NCH_2), 44.9 (CHAr), 30.3 (NCH_2*C*H_2);

^{19}F{^{1}H} NMR (376 MHz, $CDCl_3$) δ –118.67 (s);

HRMS (ESI) *m/z* Calculated for $C_{11}H_{15}$NOF [M+H] 196.1138; Found 196.1140.

cis-(±)-Benzyl 2-carbamoyl-3-(4-methoxyphenyl)piperidine-1-carboxylate (23a)

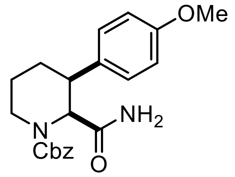

Adapting the literature conditions reported by Maulide,[28b] a solution of the *cis*-arylated piperidine **4a** (740 mg, 1.50 mmol, 1.0 equiv) in anhydrous CH_2Cl_2 (15 mL) was cooled to –78 °C and a stream of ozone was passed through until a dark green coloration occurred (approx. 4 h). Upon passing a stream of oxygen, decolorisation to pale yellow occurred and the reaction was immediately quenched by addition of dimethylsulfide (DMS, 0.4 mL, 15.00 mmol, 10.0 equiv). The mixture was stirred at rt for 18 h, concentrated under reduced pressure and the residue dissolved in NH_4OH (aq. 28%)/THF (8 mL, 1:1 v/v). After stirring at rt for 20 h, the reaction mixture was diluted with CH_2Cl_2 (15 mL) and washed with sat. aq. NH_4Cl (10 mL). The organic layer was separated and the aqueous layer was extracted with CH_2Cl_2 (3 × 15 mL). The combined organic layers were dried over Na_2SO_4 and the solvent was removed under reduced pressure. The crude material was purified by flash column chromatography (10% to 40% EtOAc/hexane) to afford amide **23a** as a yellow powder (293 mg, 0.80 mmol, 55%).

R_f 0.24 (40% EtOAc/hexane);

mp = 145–147 °C;

IR (film)/cm^{-1} 3358 (br NH), 3064, 2956, 2836, 1703 (C=O), 1613, 1513, 1416, 1361, 1249, 1185, 1129, 835, 734;

^{1}H NMR (400 MHz, $CDCl_3$, mixture of rotamers) δ 7.53–7.30 (m, 5 H, 5 × HC_{Ar}), 7.31–7.19 (m, 2 H, 2 × HC_{Ar}), 6.97–6.80 (m, 2 H, 2 × HC_{Ar}), 5.40–5.09 (m, 3 H, N*H*H amide and CH_2Ph), 5.09 (br s, 1 H, NH*H* amide), 4.86 (br t, *J* = 5.6, 1 H, CH(C=O)), 4.27–4.06 (m, 1 H, CHAr), 3.81 (s, 3 H, OCH_3), 3.49 (td, *J* = 13.1, 3.3 Hz, 1 H, NC*H*H), 3.02 (ddd, *J* = 13.1, 5.6, 3.5 Hz, 1 H, NCH*H*), 2.57–2.35 (m, 1 H, C*H*HCHAr), 2.05–1.78 (m, 2 H, CH*H*CHAr and NCH_2C*H*H), 1.63 (tt, *J* = 13.0, 4.3 Hz, 1 H, NCH_2CH*H*);

^{13}C NMR (101 MHz, $CDCl_3$, mixture of rotamers) δ 171.7 (C=O amide), 158.6 (C_{Ar} quat), 156.6 (C=O carbamate), 136.5 (C_{Ar} quat), 133.0 (C_{Ar} quat), 129.1 (2 × C_{Ar}), 128.6 (C_{Ar}), 128.5 (2 × C_{Ar}), 127.9 (2 × C_{Ar}), 113.9 (2 × C_{Ar}), 67.6 (CH_2Ph), 59.4 and 58.6 (*C*H(C=O)), 55.2 (OCH_3), 43.6 and 43.2 (CHAr), 41.1 (NCH_2), 25.5 and 25.2 (*C*H_2CHAr), 24.2 and 23.9 (NCH_2*C*H_2);

HRMS (ESI) *m/z* Calculated for $C_{21}H_{25}N_2O_4$ [M+H] 369.4410; Found 369.4403.

cis-(±)-3-(4-Methoxyphenyl)piperidine-2-carboxamide (FRAG7)

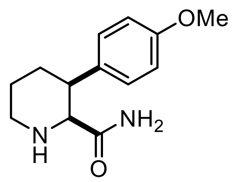

Pd/C (33 mg, 10 wt. %) was added to a 5 mL microwave vial containing a solution a solution of the desired *cis*-arylated piperidine **23a** (130 mg, 0.35 mmol, 1.0 equiv) in EtOH (1.7 mL, 0.2 M). The vial was evacuated and backfilled with Ar three times, followed by evacuation and backfilling with H_2 (balloon). The resulting solution was stirred vigorously at rt for 2 h, then filtered through Celite, washing with EtOH (20 mL). Concentration of the filtrate under reduced pressure afforded *cis*-arylated amide **FRAG7** as a white solid (60 mg, 0.25 mmol, 70%).

R_f 0.18 (15% MeOH/CH_2Cl_2);

mp = 136–138 °C;

IR (film)/cm^{-1} 3459 (br NH), 3317 (br NH), 3198, 1674 (C=O), 1513, 1249, 1182, 1036, 731;

^{1}H NMR (400 MHz, $CDCl_3$) δ 7.45 (d, *J* = 8.4 Hz, 2 H, 2 × HC_{Ar}), 6.91–6.78 (m, 2 H, 2 × HC_{Ar}), 5.86 (br s, 1 H, N*H*H amide), 5.15 (br s, 1 H, NH*H* amide), 3.80 (s, 3 H, OCH_3), 3.68 (d, *J* = 4.1 Hz, 1 H, CH(C=O)), 3.33–3.24 (m, 2 H, CHAr and NC*H*H), 2.85 (ddd, *J* = 12.8, 9.7, 3.6 Hz, 1 H, NCH*H*), 2.08–1.86 (m, 3 H, C*H*$_2$CHAr and NCH_2C*H*H), 1.53 (dt, *J* = 13.2, 4.5 Hz, 1 H, NCH_2CH*H*);

^{13}C NMR (101 MHz, $CDCl_3$) δ 175.3 (C=O amide), 158.1 (C_{Ar} quat), 134.3 (C_{Ar} quat), 130.1 (2 × C_{Ar}), 113.6 (2 × C_{Ar}), 63.4 (*C*H(C=O)), 55.2 (OCH_3), 45.6 (CHAr), 41.0 (NCH_2), 29.8 (*C*H_2CHAr), 22.2 (NCH_2*C*H_2);

HRMS (ESI) *m/z* Calculated for $C_{13}H_{19}N_2O_2$ [M+H] 235.1447; Found 235.1446.

Section 3: 3,4-Disubstituted Fragments

Synthesis overview

3,4-Disubstituted Pyrrolidine Set (FRAG8-11, FRAG13-19)

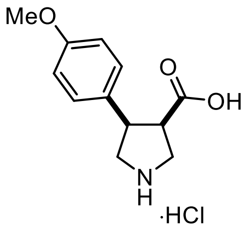

FRAG8

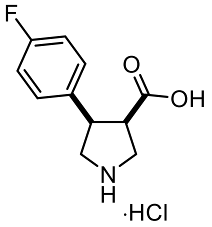

FRAG9

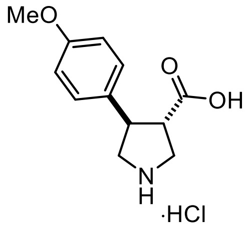

FRAG10

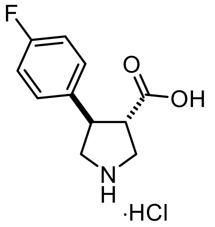

FRAG11

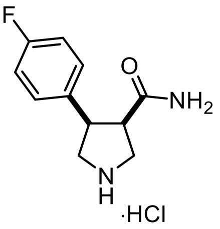

FRAG13

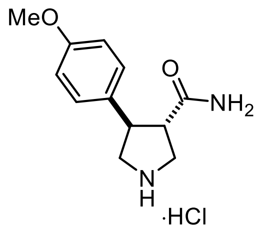

FRAG14

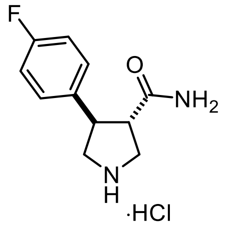

FRAG15

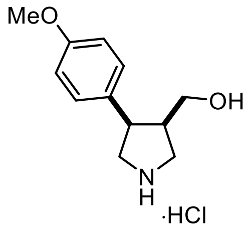

FRAG16

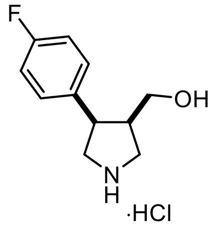

FRAG17

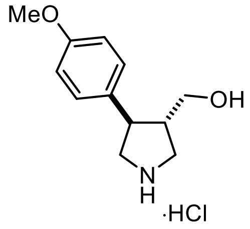

FRAG18

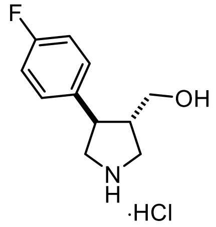

FRAG19

3,4-Disubstituted Piperidine Set (FRAG20, FRAG22-23, FRAG27-28, FRAG30)

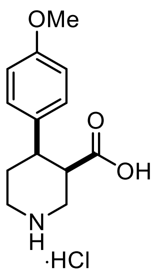

FRAG20

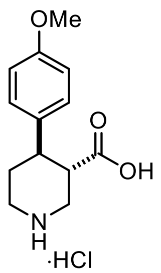

FRAG22

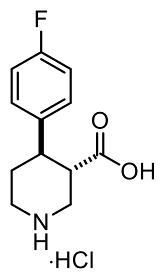

FRAG23

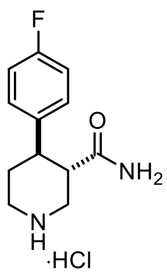

FRAG27

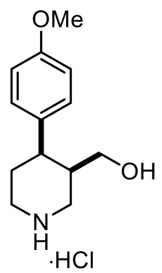

FRAG28

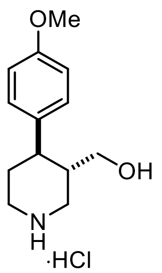

FRAG30

3,4-Disubstituted Fragments Synthetic Routes

CH arylation of aminoquinoline amides was performed as specified giving pyrrolidine intermediates **6a,b** and **7a,b** and piperidine **9a, 10a,b**.

cis-Arylation:

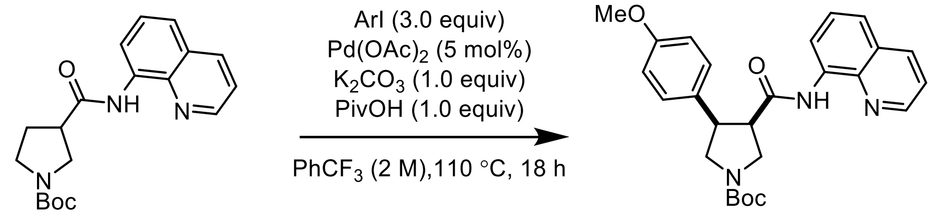

5

6a
56% (1.02 g) (4 mmol scale)

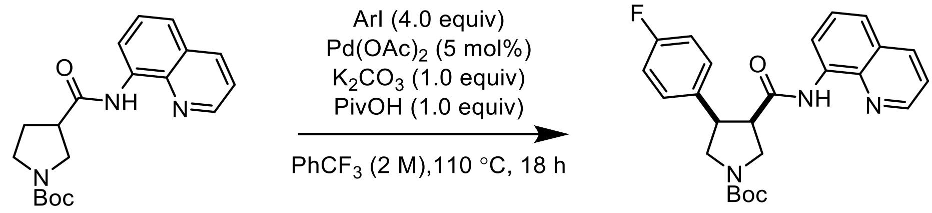

5

6b
46% (806 mg) (4 mmol scale)

trans-Arylation:

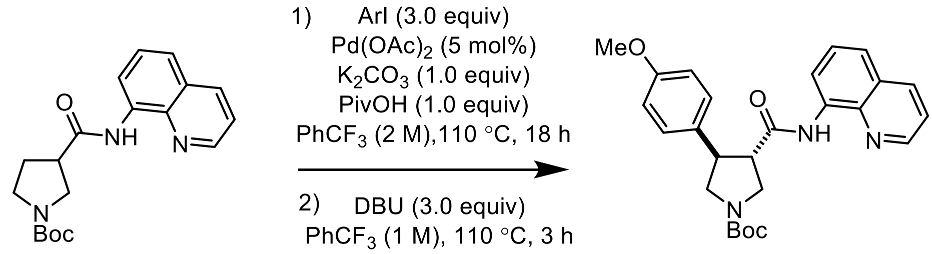

5

7a
53% (716 mg) (3 mmol scale)

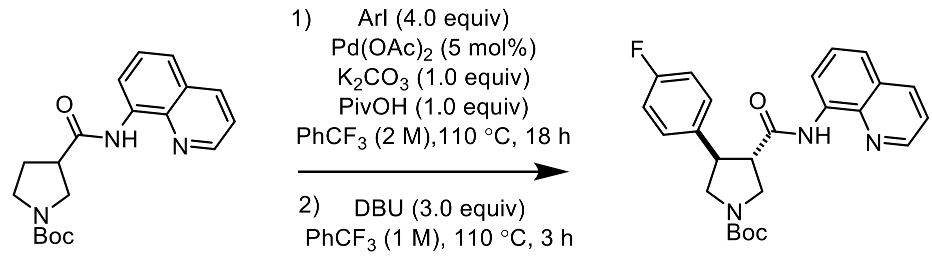

5

7b
36% (620 mg) (4 mmol scale)

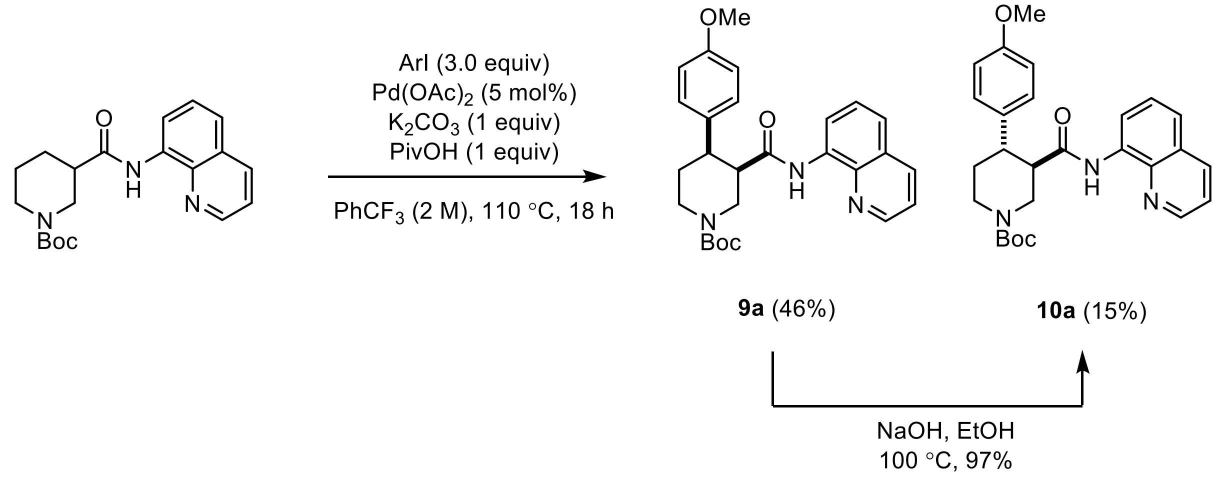

ArI (3.0 equiv)
Pd(OAc)2 (5 mol%)
K2CO3 (1 equiv)
PivOH (1 equiv)
PhCF3 (2 M), 110 °C, 18 h
OMe
O
N
H
N
Boc
9a (46%)
10a (15%)
NaOH, EtOH
100 °C, 97%

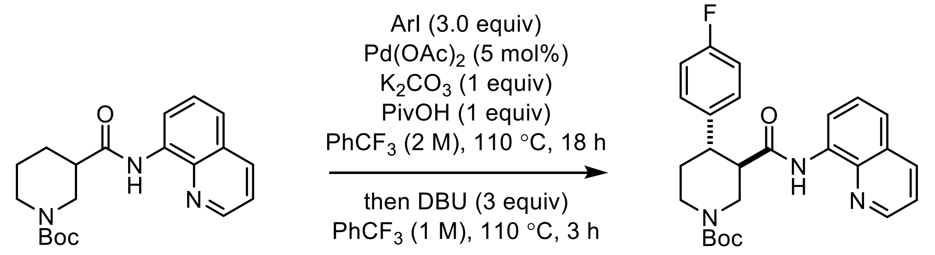

ArI (3.0 equiv)
Pd(OAc)2 (5 mol%)
K2CO3 (1 equiv)
PivOH (1 equiv)
PhCF3 (2 M), 110 °C, 18 h
then DBU (3 equiv)
PhCF3 (1 M), 110 °C, 3 h
F
O
N
H
N
Boc

10b (53%)

The synthetic route used to access each fragment from the corresponding arylated aminoquinoline amide is given below.

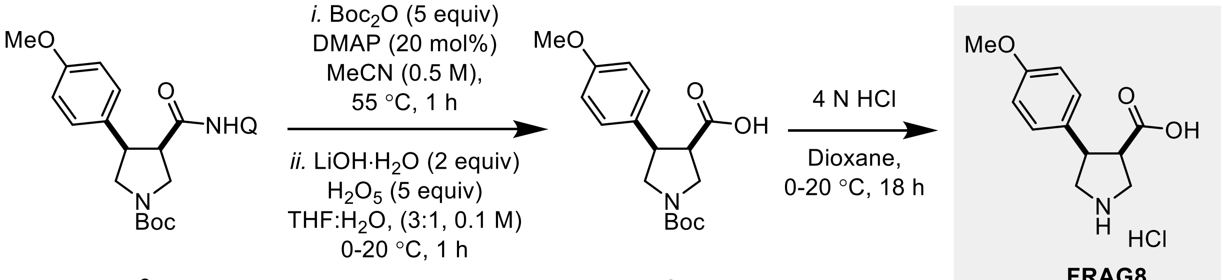

6a 89% **27a** 84% **FRAG8**

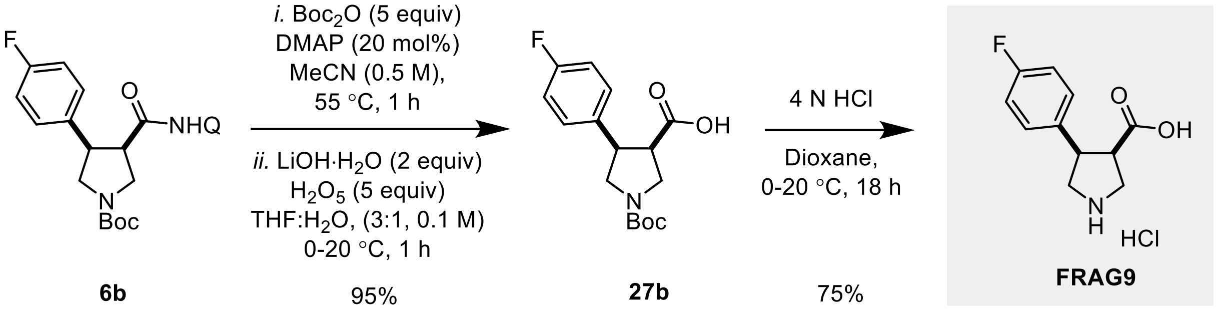

6b 95% **27b** 75% **FRAG9**

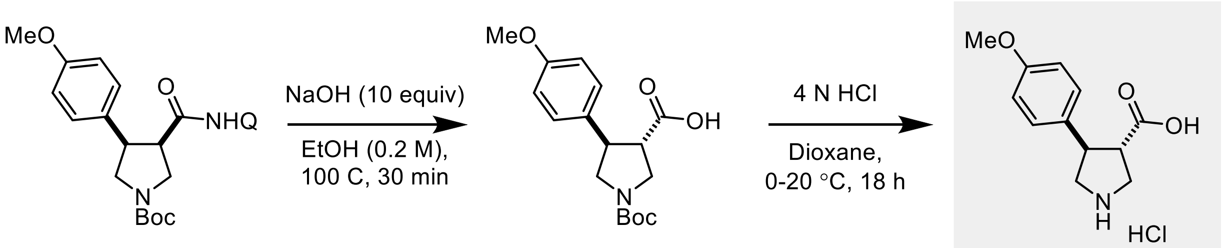

6a 82% **24a** 91% **FRAG10**

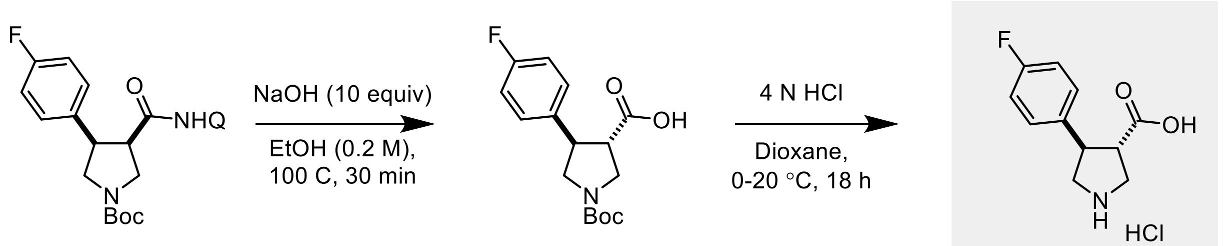

6b 96% **24b** 97% **FRAG11**

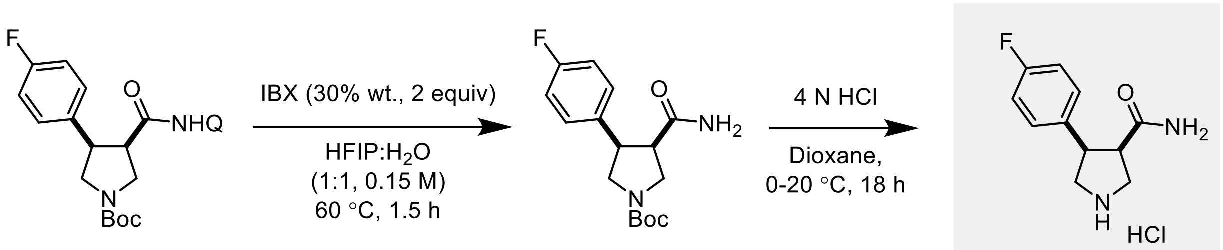

6b 77% **30b** 94% **FRAG13**

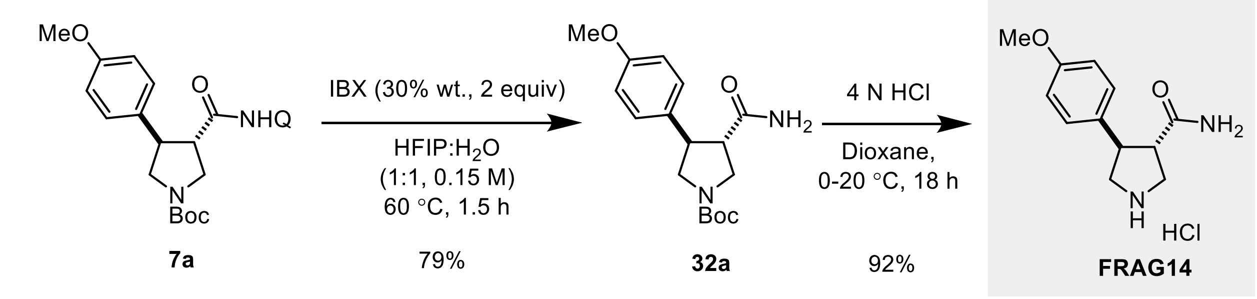
MeO
NHQ
N
Boc
7a
IBX (30% wt., 2 equiv)
HFIP:H2O
(1:1, 0.15 M)
60 °C, 1.5 h
79%
MeO
NH2
N
Boc
32a
4 N HCl
Dioxane,
0-20 °C, 18 h
92%
MeO
NH2
N
H
HCl
FRAG14

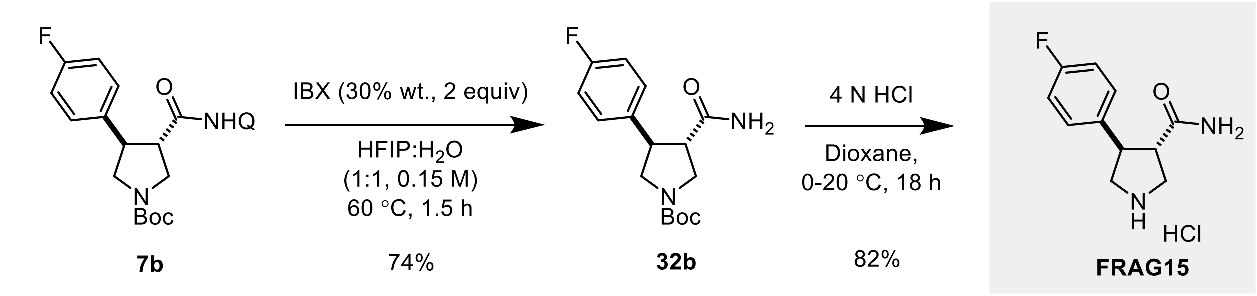
F
NHQ
N
Boc
7b
IBX (30% wt., 2 equiv)
HFIP:H2O
(1:1, 0.15 M)
60 °C, 1.5 h
74%
F
NH2
N
Boc
32b
4 N HCl
Dioxane,
0-20 °C, 18 h
82%
F
NH2
N
H
HCl
FRAG15

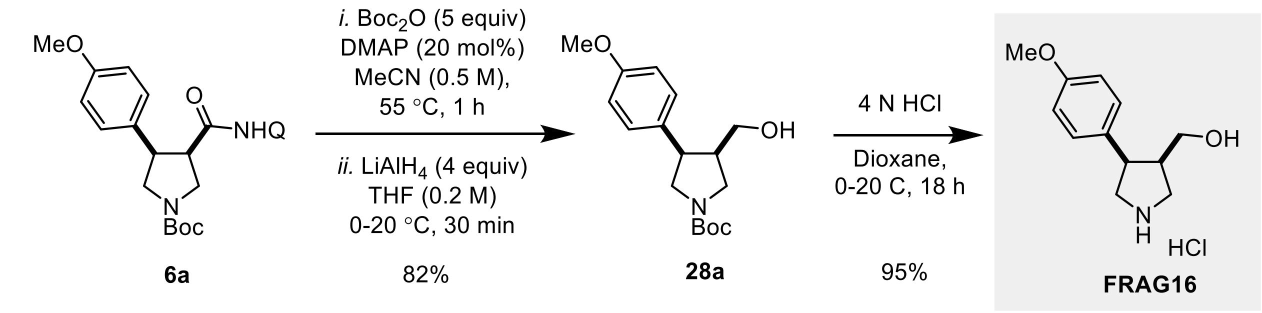
MeO
NHQ
N
Boc
6a
i. Boc2O (5 equiv)
DMAP (20 mol%)
MeCN (0.5 M),
55 °C, 1 h
ii. LiAlH4 (4 equiv)
THF (0.2 M)
0-20 °C, 30 min
82%
MeO
OH
N
Boc
28a
4 N HCl
Dioxane,
0-20 C, 18 h
95%
MeO
OH
N
H
HCl
FRAG16

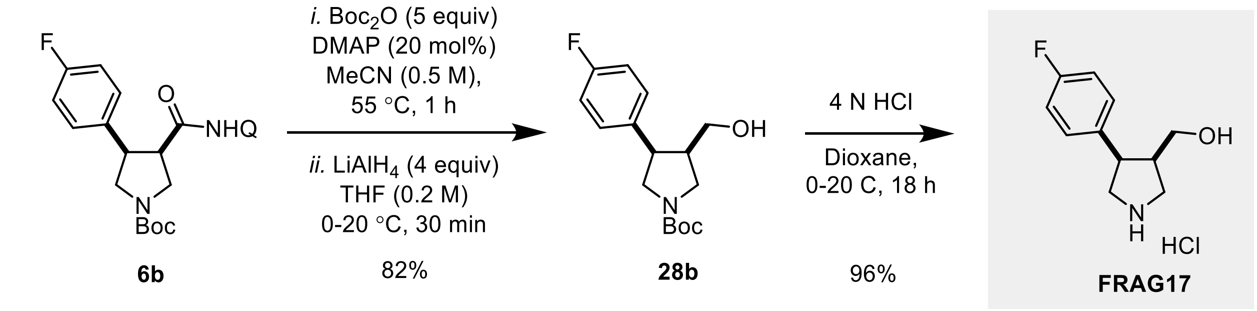
F
NHQ
N
Boc
6b
i. Boc2O (5 equiv)
DMAP (20 mol%)
MeCN (0.5 M),
55 °C, 1 h
ii. LiAlH4 (4 equiv)
THF (0.2 M)
0-20 °C, 30 min
82%
F
OH
N
Boc
28b
4 N HCl
Dioxane,
0-20 C, 18 h
96%
F
OH
N
H
HCl
FRAG17

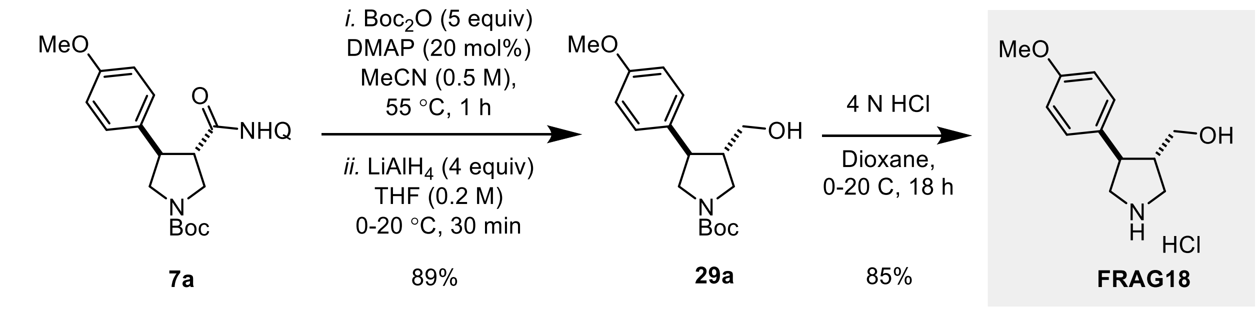
MeO
NHQ
N
Boc
7a
i. Boc2O (5 equiv)
DMAP (20 mol%)
MeCN (0.5 M),
55 °C, 1 h
ii. LiAlH4 (4 equiv)
THF (0.2 M)
0-20 °C, 30 min
89%
MeO
OH
N
Boc
29a
4 N HCl
Dioxane,
0-20 C, 18 h
85%
MeO
OH
N
H
HCl
FRAG18

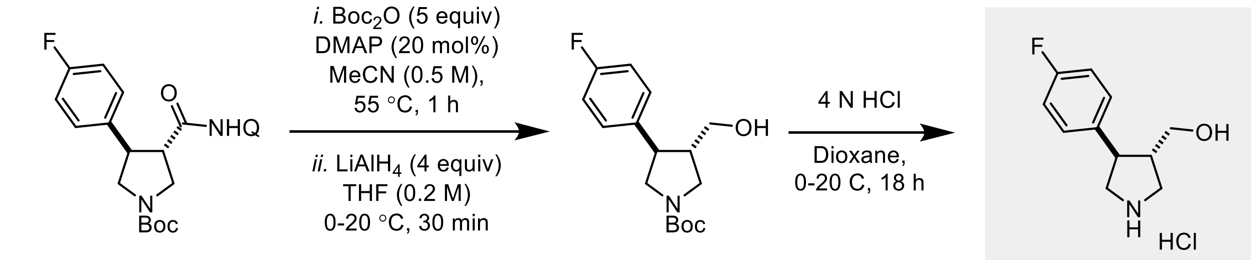

7b 88% **29b** 94% **FRAG19**

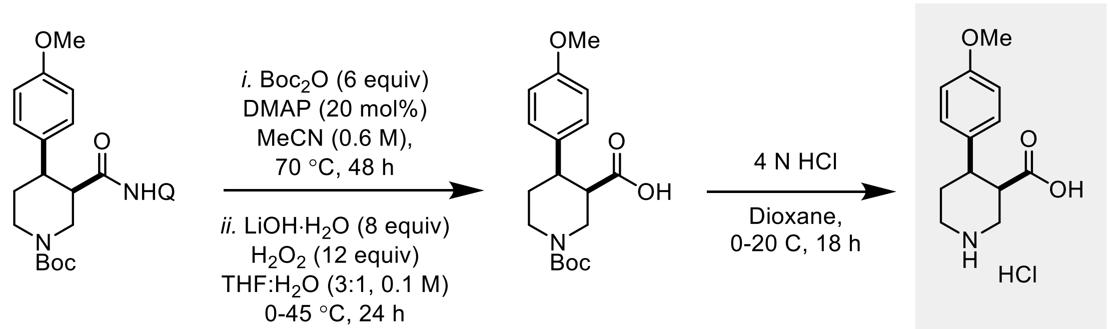

9a 89% **35a** 87% **FRAG20**

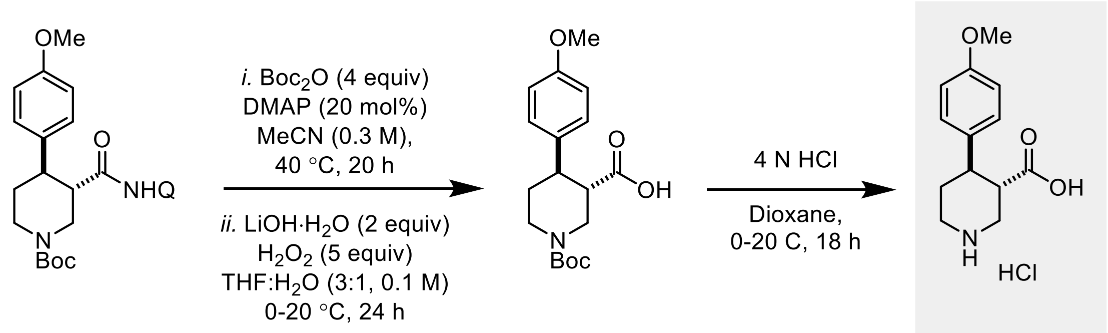

10a 89% **36a** 80% **FRAG22**

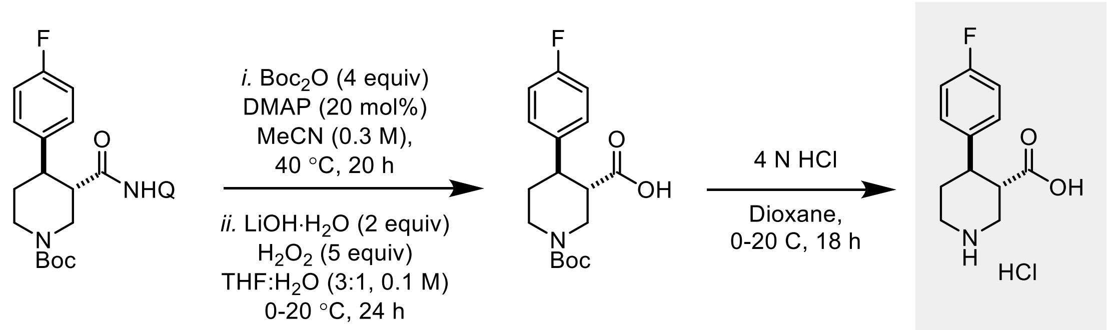

10b 93% **36b** 78% **FRAG23**

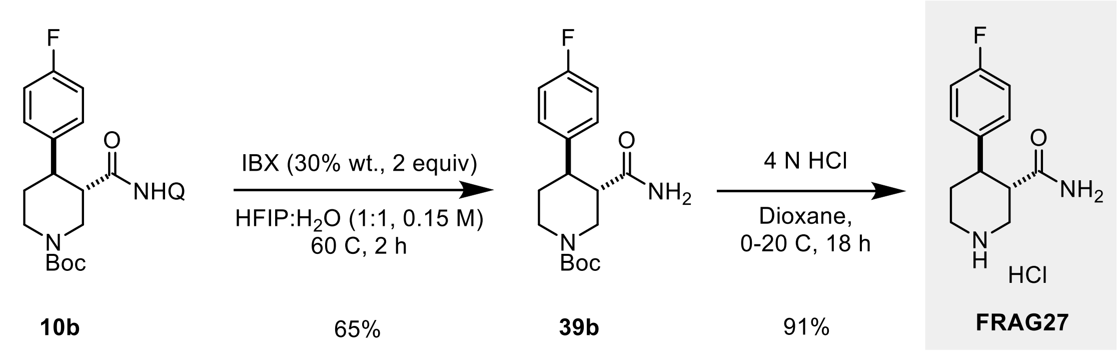
F
O
NHQ
N
Boc
IBX (30% wt., 2 equiv)
HFIP:H2O (1:1, 0.15 M)
60 C, 2 h
NH2
4 N HCl
Dioxane,
0-20 C, 18 h
H
HCl
10b
65%
39b
91%
FRAG27

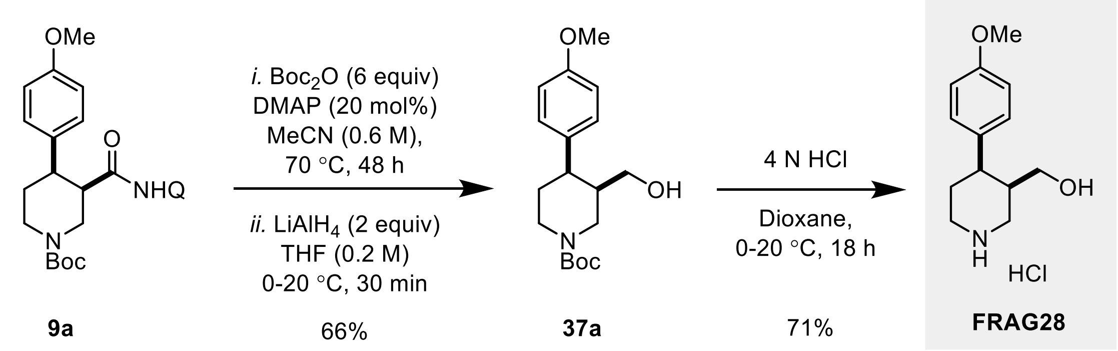
OMe
O
NHQ
N
Boc
i. Boc2O (6 equiv)
DMAP (20 mol%)
MeCN (0.6 M),
70 °C, 48 h
ii. LiAlH4 (2 equiv)
THF (0.2 M)
0-20 °C, 30 min
OH
4 N HCl
Dioxane,
0-20 °C, 18 h
H
HCl
9a
66%
37a
71%
FRAG28

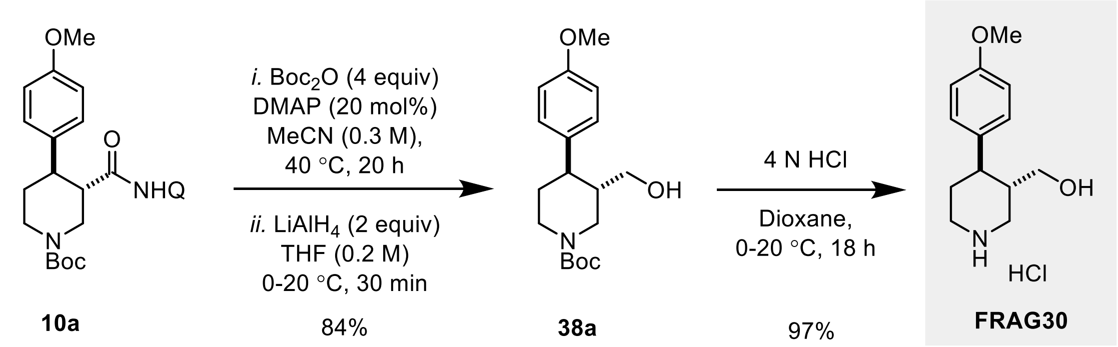
OMe
O
NHQ
N
Boc
i. Boc2O (4 equiv)
DMAP (20 mol%)
MeCN (0.3 M),
40 °C, 20 h
ii. LiAlH4 (2 equiv)
THF (0.2 M)
0-20 °C, 30 min
OH
4 N HCl
Dioxane,
0-20 °C, 18 h
H
HCl
10a
84%
38a
97%
FRAG30

C3-Directed CH Arylations

cis-(±)-tert-Butyl 3-(4-methoxyphenyl)-4-(quinolin-8-ylcarbamoyl)pyrrolidine-1-carboxylate (6a)

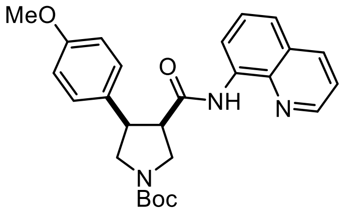

Adapting the literature conditions reported by Bull,[24a] A large microwave vial (10–20 mL recommended volume) was charged with K_2CO_3 (550 mg, 4.00 mmol), flame-dried, and allowed to cool under argon. Amide **5** (1.37 g, 4.00 mmol), 4-iodoanisole (2.81 g, 12.0 mmol), $Pd(OAc)_2$ (45.0 mg, 0.20 mmol) and PivOH (410 mg, 4.00 mmol) were added sequentially. The reaction vessel was sealed and purged with argon, then $PhCF_3$ (2.0 mL, 2.0 M) was added by syringe. The reaction tube was then placed in a preheated oil bath and stirred at 110 °C for 18 h. The reaction mixture was then allowed to cool to rt and EtOAc (20 mL) was added. The resulting mixture was filtered through a pad of silica, eluting with further EtOAc (2 × 50 mL). The solvent was removed under reduced pressure, and the crude material was purified by automated flash column chromatography (silica, 0–100% acetone/pentane). The product containing fractions were combined and the solvent was removed under reduced pressure. Et_2O (10 mL) and pentane (10 mL) were added and the solvent was removed under reduced pressure to afford the *cis*-arylated product **6a** as a pale yellow solid (1.02 g, 56%).

R_f 0.20 (20% acetone/pentane);

mp = 62–65 °C (from Et_2O/pentane);

IR (film)/cm^{-1} 3346 (NH br), 2976, 1681 (C=O), 1525, 1515, 1486, 1402, 1365, 1324, 1248, 1163, 1114, 1033, 912, 791, 758, 729;

^{1}H NMR (400 MHz, DMSO-d_6, 373 K) δ 9.69 (br s, 1 H, NH), 8.83 (dd, J = 4.2, 1.7 Hz, 1 H, HC_{Ar}), 8.35 (dd, J = 7.6, 1.4 Hz, 1 H, HC_{Ar}), 8.31 (dd, J = 8.3, 1.7 Hz, 1 H, HC_{Ar}), 7.60–7.55 (m, 2 H, HC_{Ar}), 7.47 (t, J = 7.9 Hz, 1 H, HC_{Ar}), 7.19–7.11 (m, 2 H, HC_{Ar}), 6.71–6.64 (m, 2 H, HC_{Ar}), 3.86–3.76 (m, 3 H, CHCO, CHAr, NC*H*HCHCO), 3.76–3.71 (m, 2 H, NCH_2CHAr), 3.67–3.60 (m, 1 H, NCH*H*CHCO), 3.55 (s, 3 H, OCH_3), 1.49 (s, 9 H, $C(CH_3)_3$);

^{13}C NMR (101 MHz, DMSO-d_6, 373 K) δ 169.4 (C=O amide), 157.7 (OC_{Ar} quat), 153.2 (C=O carbamate), 147.9 (C_{Ar}), 137.6 (C_{Ar} quat), 135.7 (C_{Ar}), 133.6 (C_{Ar} quat), 129.9 (C_{Ar} quat), 128.0 (2 × C_{Ar}), 127.2 (C_{Ar} quat), 126.1 (C_{Ar}), 121.2 (C_{Ar}), 121.1 (C_{Ar}), 116.0 (C_{Ar}), 113.3 (2 × C_{Ar}), 78.0 ($C(CH_3)_3$), 54.4 (OCH_3), 49.8 (NCH_2CHAr), 49.2 (*C*HCO), 47.4 (NCH_2CHCO), 44.8 (*C*HAr), 27.8 ($C(CH_3)_3$);

HRMS (ESI$^+$) *m/z* Calculated for $C_{26}H_{30}N_3O_4$ [M+H] 448.2236; Found 448.2238.

Characterisation information consistent with data previously reported in literature.[24a]

trans-(±)-tert-Butyl 3-(4-methoxyphenyl)-4-(quinolin-8-ylcarbamoyl)pyrrolidine-1-carboxylate (7a)

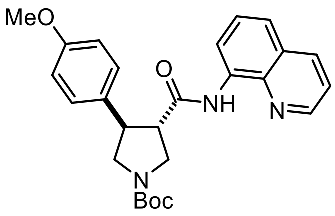

A large microwave vial (10–20 mL recommended volume) was charged with K_2CO_3 (420 mg, 3.00 mmol), flame-dried, and allowed to cool under argon. Amide **5** (1.02 g, 3.00 mmol), 4-iodoanisole (2.11 g, 9.00 mmol), $Pd(OAc)_2$ (33.8 mg, 0.15 mmol) and PivOH (310 mg, 3.00 mmol) were added sequentially. The reaction vessel was purged with argon, then $PhCF_3$ (1.5 mL, 2.0 M) was added by syringe. The reaction tube was placed in a preheated oil bath and stirred at 110 ºC for 18 h. The reaction mixture was then allowed to cool to rt and $PhCF_3$ (1.5 mL, 1.0 M) and DBU (1.3 mL, 9.00 mmol) were added by syringe. The reaction vessel was then stirred at 110 °C for additional 3 h. The reaction mixture was allowed to cool to rt and EtOAc (15 mL) was added. The resulting mixture was filtered through a pad of Celite, eluting with further EtOAc (2 × 40 mL). The solvent was removed under reduced pressure, and the crude material was purified by flash column chromatography (5% to 8% Et_2O/pentane) affording the *trans*-arylated pyrrolidine **7a** as a pale yellow solid (720 mg, 53%).

R_f 0.20 (20% acetone/hexane);

mp = 145–150 °C (from Et_2O/pentane);

IR (film)/cm^{-1} 3341 (NH br), 2973, 1684 (C=O), 1526, 1485, 1387, 1325, 1246, 1161, 1125, 1033, 881, 826, 791, 770;

^{1}H NMR (400 MHz, DMSO-d_6, 373 K) δ 9.81 (br s, 1 H, NH), 8.77 (dd, *J* = 4.2, 1.7 Hz, 1 H, HC_{Ar}), 8.55 (dd, *J* = 7.6, 1.4 Hz, 1 H, HC_{Ar}), 8.32 (dd, *J* = 8.3, 1.7 Hz, 1 H, HC_{Ar}), 7.61 (dd, *J* = 8.3, 1.4 Hz, 1 H, HC_{Ar}), 7.58–7.49 (m, 2 H, HC_{Ar}), 7.37–7.30 (m, 2 H, HC_{Ar}), 6.91–6.86 (m, 2 H, HC_{Ar}), 3.92–3.87 (m, 1 H, NC*H*HCHCO), 3.87–3.82 (m, 1 H, NC*H*HCHAr), 3.72 (s, 3 H, OCH_3), 3.71–3.57 (m, 3 H, NCH*H*CHCO, CHCO, CHAr), 3.38–3.26 (m, 1 H, NCH*H*CHAr), 1.46 (s, 9 H, $C(CH_3)_3$);

^{13}C NMR (101 MHz, DMSO-d_6, 373 K) δ 169.4 (C=O amide), 158.2 (OC_{Ar} quat), 152.9 (C=O carbamate), 148.0 (C_{Ar}), 137.7 (C_{Ar} quat), 135.8 (C_{Ar}), 133.7 (C_{Ar} quat), 130.9 (C_{Ar} quat), 128.0 (2 × C_{Ar}), 127.3 (C_{Ar} quat), 126.2 (C_{Ar}), 121.44 (C_{Ar}), 121.37 (C_{Ar}), 116.2 (C_{Ar}), 113.9 (2 × C_{Ar}), 78.2 ($C(CH_3)_3$), 54.7 (OCH_3), 52.6 (NCH_2CHAr), 51.0 (*C*HCO), 48.6 (NCH_2CHCO), 46.2 (CHAr), 27.8 ($C(CH_3)_3$);

HRMS (ESI$^+$) *m/z* Calculated for $C_{26}H_{30}N_3O_4$ [M+H] 448.2236; Found 448.2224.

cis-(±)-tert-Butyl 3-(4-fluorophenyl)-4-(quinolin-8-ylcarbamoyl)pyrrolidine-1-carboxylate (6b)

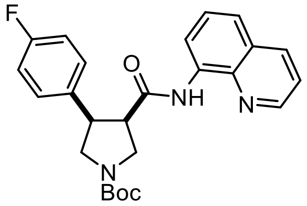

Adapting the literature conditions reported by Bull,[24a] a large microwave vial (10–20 mL recommended volume) was charged with K_2CO_3 (550 mg, 4.00 mmol), flame-dried, and allowed to cool under argon. Amide **5** (1.37 g, 4.00 mmol), 4-fluoroiodobenzene (3.55 g, 16.0 mmol), $Pd(OAc)_2$ (45.0 mg, 0.20 mmol) and PivOH (410 mg, 4.00 mmol) were added sequentially. The reaction vessel was sealed and purged with argon, then $PhCF_3$ (2.0 mL, 2.0 M) was added by syringe. The reaction tube was then placed in a preheated oil bath and stirred at 110 °C for 18 h. The reaction mixture was then allowed to cool to rt and EtOAc (20 mL) was added. The resulting mixture was filtered through a pad of silica, eluting with further EtOAc (2 × 50 mL). The solvent was removed under reduced pressure, and the crude material was purified by automated flash column chromatography (silica, 0–100% acetone/pentane). The product containing fractions were combined and the solvent was removed under reduced pressure. Et_2O (10 mL) and pentane (10 mL) were added and the solvent was removed under reduced pressure to afford the *cis*-arylated product **6b** as an off-white solid (810 mg, 46%).

The same procedure was repeated on a second batch of amide **5** (1.00 g, 3.00 mmol), using K_2CO_3 (415 mg, 3.00 mmol), 4-fluoroiodobenzene (2.67 g, 12.0 mmol), $Pd(OAc)_2$ (33.8 mg, 0.15 mmol), PivOH (307 mg, 3.00 mmol) and $PhCF_3$ (1.5 mL, 2.0 M). Purification by automated flash column chromatography (silica, 0–100% acetone/pentane) afforded the *cis*-arylated product **6b** as an off-white solid (573 mg, 44%).

R_f 0.12 (10% acetone/pentane);

mp = 94–97 °C (from Et_2O/pentane);

ν_{max} (film)/cm^{-1} 3334 (NH br), 2973, 1678 (C=O), 1526, 1486, 1391, 1324, 1226, 1164, 1119, 827, 791, 769;

^{1}H NMR (400 MHz, DMSO-d_6, 373 K) δ 9.73 (br s, 1 H, NH), 8.85 (dd, J = 4.2, 1.7 Hz, 1 H, HC_{Ar}), 8.33 (m, 1 H, HC_{Ar}), 8.31 (m, 1 H, HC_{Ar}), 7.61–7.55 (m, 2 H, HC_{Ar}), 7.47 (t, J = 8.0 Hz, 1 H, HC_{Ar}), 7.30–7.24 (m, 2 H, HC_{Ar}), 6.94–6.87 (m, 2 H, HC_{Ar}), 3.93–3.84 (m, 2 H, CHCO, CHAr), 3.84–3.71 (m, 3 H, NC*H*HCHCO, NCH_2CHAr), 3.66 (dd, J = 10.9, 6.7 Hz, 1 H, NCH*H*CHCO), 1.49 (s, 9 H, $C(CH_3)_3$);

^{13}C NMR (101 MHz, DMSO-d_6, 373 K) δ 169.3 (C=O amide), 160.4 (d, J_{CF} = 243.4 Hz, FC_{Ar} quat), 153.2 (C=O carbamate), 148.0 (C_{Ar}), 137.7 (C_{Ar} quat), 135.8 (C_{Ar}), 134.2 (d, J_{CF} = 3.3 Hz, F-*p*-C_{Ar} quat), 133.5 (C_{Ar} quat), 128.9 (d, J_{CF} = 8.1 Hz, 2 × F-*m*-C_{Ar}), 127.2 (C_{Ar} quat), 126.1 (C_{Ar}), 121.3 (C_{Ar}), 121.2 (C_{Ar}), 116.2 (C_{Ar}), 114.2 (d, J_{CF} = 21.0 Hz, 2 × F-*o*-C_{Ar}), 78.1 (*C*$(CH_3)_3$), 49.7 (N*C*H_2CHAr), 49.0 (*C*HCO), 47.4 (N*C*H_2CHCO), 44.8 (CHAr), 27.8 (C(*C*$H_3)_3$);

^{19}F NMR (471 MHz, DMSO-d_6, 373 K) δ –116.3 (tt, J_{HF} = 8.6, 5.8 Hz);

HRMS (ESI$^+$) *m/z* Calculated for $C_{25}H_{27}N_3O_3F$ [M+H] 436.2036; Found 436.2053.

Characterisation information consistent with data previously reported in literature.[24a]

trans-(±)-tert-Butyl 3-(4-fluorophenyl)-4-(quinolin-8-ylcarbamoyl)pyrrolidine-1-carboxylate (7b)

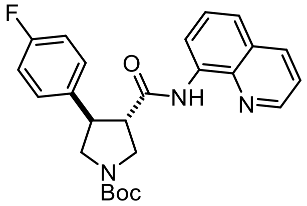

A large microwave vial (10–20 mL recommended volume) was charged with K_2CO_3 (550 mg, 4.00 mmol), flame-dried, and allowed to cool under argon. Amide **5** (1.37 g, 4.00 mmol), 4-fluoroiodobenzene (3.55 g, 16.0 mmol), $Pd(OAc)_2$ (45.0 mg, 0.20 mmol) and PivOH (410 mg, 4.00 mmol) were added sequentially. The reaction vessel was sealed and purged with argon, then $PhCF_3$ (2.0 mL, 2.0 M) was added by syringe. The reaction tube was placed in a preheated oil bath and stirred at 110 °C for 18 h. The reaction mixture was then allowed to cool to rt and $PhCF_3$ (2.0 mL, 1.0 M) and DBU (1.8 mL, 12.0 mmol) were added by syringe. The reaction vessel was then stirred at 110 °C for additional 3 h. The reaction mixture was allowed to cool to rt and EtOAc (20 mL) was added. The resulting mixture was filtered through a pad of Celite, eluting with further EtOAc (2 × 50 mL). The solvent was removed under reduced pressure, and the crude material was purified by flash column chromatography (5% Et_2O/CH_2Cl_2). The product containing fractions were combined and the solvent was removed under reduced pressure. Et_2O (10 mL) and pentane (10 mL) were added, and the solvent was removed under reduced pressure to afford the *trans*-arylated product **7b** as a pale yellow solid (620 mg, 36%).

R_f 0.23 (5% Et_2O/CH_2Cl_2).

mp = 70–74 °C (from Et_2O/pentane);

IR (film)/cm^{-1} 3332 (NH br), 2974, 1685 (C=O), 1528, 1486, 1387, 1327, 1159, 1126, 828, 790;

^{1}H NMR (500 MHz, DMSO-d_6, 373 K) δ 9.82 (br s, 1 H, NH), 8.78 (dd, J = 4.2, 1.7 Hz, 1 H, HC_{Ar}), 8.54 (dd, J = 7.7, 1.3 Hz, 1 H, HC_{Ar}), 8.32 (dd, J = 8.3, 1.7 Hz, 1 H, HC_{Ar}), 7.62 (dd, J = 8.3, 1.3 Hz, 1 H, HC_{Ar}), 7.57 (dd, J = 8.3, 4.2 Hz, 1 H, HC_{Ar}), 7.53 (t, J = 7.9 Hz, 1 H, HC_{Ar}), 7.49–7.44 (m, 2 H, HC_{Ar}), 7.14–7.08 (m, 2 H, HC_{Ar}), 3.93–3.86 (m, 2 H, NC*H*HCHAr, NC*H*HCHCO), 3.78–3.69 (m, 2 H, CHCO, CHAr), 3.65–3.58 (m, 1 H, NCH*H*CHCO), 3.38–3.32 (m, 1 H, NCH*H*CHAr), 1.46 (s, 9 H, $C(CH_3)_3$);

^{13}C NMR (126 MHz, DMSO-d_6, 373 K) δ 169.2 (C=O amide), 161.0 (d, J_{CF} = 243.5 Hz, FC_{Ar} quat), 152.9 (C=O carbamate), 148.0 (C_{Ar}), 137.7 (C_{Ar} quat), 135.8 (C_{Ar}), 135.1 (d, J_{CF} = 2.9 Hz, F-*p*-C_{Ar} quat), 133.6 (C_{Ar} quat), 129.0 (d, J_{CF} = 8.1 Hz, 2 × F-*m*-C_{Ar}), 127.3 (C_{Ar} quat), 126.2 (C_{Ar}), 121.5 (C_{Ar}), 121.4 (C_{Ar}), 116.3 (C_{Ar}), 114.8 (d, J_{CF} = 21.3 Hz, 2 × F-*o*-C_{Ar}), 78.2 (*C*$(CH_3)_3$), 52.4 (NCH_2CHAr), 50.9 (*C*HCO), 48.5 (NCH_2CHCO), 46.0 (CHAr), 27.7 (C$(CH_3)_3$);

^{19}F NMR (471 MHz, DMSO-d_6, 373 K) δ –115.9 (tt, J_{HF} = 9.7, 5.6 Hz);

HRMS (ESI$^+$) *m/z* Calculated for $C_{25}H_{27}N_3O_3F$ [M+H] 436.2036; Found 436.2030.

***cis*-(±)-*tert*-Butyl 4-(4-methoxyphenyl)-3-(quinolin-8-ylcarbamoyl)piperidine-1-carboxylate (9a)** and

***trans*-(±)-*tert*-Butyl 4-(4-methoxyphenyl)-3-(quinolin-8-ylcarbamoyl)piperidine-1-carboxylate (10a)**

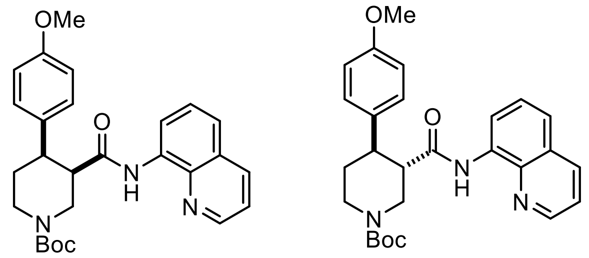

Adapting the literature conditions reported by Bull,[24a] a large microwave vial (10–20 mL recommended volume) was charged with K_2CO_3 (550 mg, 4.00 mmol), flame-dried, and allowed to cool under argon. Amide **8** (1.42 g, 4.00 mmol), 4-iodoanisole (2.81 g, 12 mmol), $Pd(OAc)_2$ (45.0 mg, 0.20 mmol) and PivOH (410 mg, 4.00 mmol) were added sequentially. The reaction vessel was sealed and purged with argon, then $PhCF_3$ (2.0 mL, 2.0 M) was added by syringe. The reaction tube was then placed in a preheated oil bath and stirred at 110 °C for 18 h. The reaction mixture was then allowed to cool to rt and EtOAc (20 mL) was added. The resulting mixture was filtered through a pad of silica, eluting with further EtOAc (2 × 50 mL). The solvent was removed under reduced pressure, and the crude material was purified by automated flash column chromatography (silica, 0–100% acetone/pentane). The product containing fractions were combined and the solvent was removed under reduced pressure. Et_2O (10 mL) and pentane (10 mL) were added and the solvent was removed under reduced pressure to afford the minor product *tert*-butyl *trans*-(±)-4-(4-methoxyphenyl)-3-(quinolin-8-ylcarbamoyl) piperidine-1-carboxylate **10a** as a white solid (286 mg, 15%) and the major product *tert*-butyl *cis*-(±)-4-(4-methoxyphenyl)-3-(quinolin-8-ylcarbamoyl)piperidine-1-carboxylate **9a** as a white solid (852 mg, 46%).

The same procedure was repeated on a second batch of amide **8** (1.22 g, 3.40 mmol), using K_2CO_3 (470 mg, 3.40 mmol), 4-iodoanisole (2.41 g, 10.3 mmol), $Pd(OAc)_2$ (38.3 mg, 0.17 mmol), PivOH (348 mg, 3.40 mmol) and $PhCF_3$ (1.7 mL, 2.0 M). Manual purification by flash column chromatography (5% to 15% acetone/pentane) afforded the minor product *tert*-butyl *trans*-(±)-4-(4-methoxyphenyl)-3-(quinolin-8-ylcarbamoyl) piperidine-1-carboxylate **10a** as a white solid (253 mg, 16%) and the major product *tert*-butyl *cis*-(±)-4-(4-methoxyphenyl)-3-(quinolin-8-ylcarbamoyl)piperidine-1-carboxylate **9a** as a white solid (821 mg, 52%).

Major *cis*-arylated Product (**9a**):

R_f 0.20 (15% acetone/pentane);

mp = 68–72 °C (from Et_2O/pentane);

IR (film)/cm^{-1} 3355 (NH), 2971, 1684 (C=O), 1522, 1485, 1424, 1324, 1246, 1162, 1120, 1035, 1004, 827, 792, 759;

^{1}H NMR (400 MHz, DMSO-d_6, 373 K) δ 9.71 (br s, 1 H, NH), 8.82 (dd, *J* = 4.2, 1.7 Hz, 1 H, HC_{Ar}), 8.47 (dd, *J* = 7.6, 1.4 Hz, 1 H, HC_{Ar}), 8.31 (dd, *J* = 8.3, 1.7 Hz, 1 H, HC_{Ar}), 7.58–7.54 (m, 2 H, HC_{Ar}), 7.48 (t, *J* = 7.9 Hz, 1 H, HC_{Ar}), 7.27–7.22 (m, 2 H, HC_{Ar}), 6.78–6.74 (m, 2 H, HC_{Ar}), 4.39 (ddd, *J* = 13.7, 2.5, 1.8 Hz, 1 H, NC*H*HCHCO), 4.24 (dddd, *J* = 13.1, 4.7, 2.8, 1.8 Hz, 1 H, NC*H*HCH_2), 3.63 (s, 3 H, OCH_3), 3.31 (dd, *J* = 13.7, 3.9 Hz, 1 H, NCH*H*CHCO), 3.26–3.21 (m, 1 H, CHCO), 3.12 (ddd, *J* = 12.3, 4.3, 4.3 Hz, 1 H, CHAr), 3.01–2.93 (m, 1 H, NCH*H*CH_2), 2.68 (dddd, *J* = 12.2, 12.2, 12.2, 4.7, 1 H, NCH_2C*H*H), 1.74–1.67 (m, 1 H, NCH_2CH*H*), 1.26 (s, 9 H, $C(CH_3)_3$);

^{13}C NMR (101 MHz, DMSO-d_6, 373 K) δ 170.1 (C=O amide), 157.3 (OC_{Ar} quat), 153.4 (C=O carbamate), 147.8 (C_{Ar}), 137.6 (C_{Ar} quat), 135.7 (C_{Ar}), 134.6 (C_{Ar} quat), 134.1 (C_{Ar} quat), 127.8 (2 × C_{Ar}), 127.2 (C_{Ar} quat), 126.2 (C_{Ar}), 121.2 (C_{Ar}), 120.7 (C_{Ar}), 115.6 (C_{Ar}), 113.2 (2 × C_{Ar}), 77.9 ($C(CH_3)_3$), 54.5 (OCH_3), 46.2 (NCH_2CHCO), 46.1 ($CHCO$), 43.1 (NCH_2CH_2), 41.6 (CHAr), 27.4 ($C(CH_3)_3$), 25.5 (NCH_2CH_2);

HRMS (ESI$^+$) *m/z* Calculated for $C_{27}H_{32}N_3O_4$ [M+H] 462.2393; Found 462.2386.

Minor *trans*-arylated Product (**10a**):

R_f 0.27 (15% acetone/pentane);

mp = 59–63 °C (from Et_2O/pentane);

IR (film)/cm^{-1} 3348 (NH), 2931, 1681 (C=O), 1525, 1514, 1486, 1424, 1366, 1284, 1233, 1158, 1126, 1034, 826, 791, 762;

^{1}H NMR (500 MHz, DMSO-d_6, 373 K) δ 9.69 (br s, 1 H, NH), 8.84 (dd, *J* = 4.2, 1.7 Hz, 1 H, HC_{Ar}), 8.41 (dd, *J* = 7.7, 1.3 Hz, 1 H, HC_{Ar}), 8.31 (dd, *J* = 8.3, 1.7 Hz, 1 H, HC_{Ar}), 7.59–7.54 (m, 2 H, HC_{Ar}), 7.47 (t, *J* = 7.9 Hz, 1 H, HC_{Ar}), 7.26–7.23 (m, 2 H, HC_{Ar}), 6.76–6.63 (m, 2 H, HC_{Ar}), 4.37–4.30 (m, 1 H, NC*H*HCHCO), 4.12 (dddd, *J* = 13.2, 4.4, 2.2, 2.2 Hz, 1 H, NC*H*HCH_2), 3.59 (s, 3 H, OCH_3), 3.07–3.01 (m, 3 H, NCH*H*CHCO, CHCO, CHAr), 2.97–2.90 (m, 1 H, NCH*H*CH_2), 1.81–1.76 (m, 1 H, NCH_2C*H*H), 1.71–1.60 (m, 1 H, NCH_2CH*H*), 1.48 (s, 9 H, $C(CH_3)_3$);

^{13}C NMR (126 MHz, DMSO-d_6, 373 K) δ 170.0 (C=O amide), 157.5 (OC_{Ar} quat), 153.4 (C=O carbamate), 148.0 (C_{Ar}), 137.6 (C_{Ar} quat), 135.7 (C_{Ar}), 135.0 (C_{Ar} quat), 133.6 (C_{Ar} quat), 127.7 (2 × C_{Ar}), 127.2 (C_{Ar} quat), 126.1 (C_{Ar}), 121.3 (C_{Ar}), 121.2 (C_{Ar}), 116.1 (C_{Ar}), 113.5 (2 × C_{Ar}), 78.5 ($C(CH_3)_3$), 54.5 (OCH_3), 49.8 ($CHCO$), 46.3 (NCH_2CHCO), 43.6 (CHAr), 43.5 (NCH_2CH_2), 32.4 (NCH_2CH_2), 27.7 ($C(CH_3)_3$);

HRMS (ESI$^+$) *m/z* Calculated for $C_{27}H_{32}N_3O_4$ [M+H] 462.2393; Found 462.2403.

Characterisation information consistent with data previously reported in literature.[24a]

trans-(±)-tert-Butyl 4-(4-methoxyphenyl)-3-(quinolin-8-ylcarbamoyl)piperidine-1-carboxylate (10a)

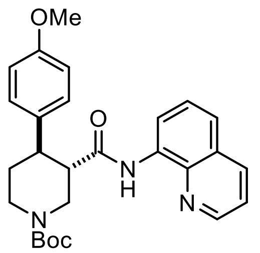

A flame-dried round-bottom-flask was charged with *cis*-arylated amide **9a** (224 mg, 0.49 mmol) and EtOH (2.5 mL, 0.2 M). NaOH (powder, 199 mg, 4.90 mmol) was then added and the resulting mixture was stirred at 100 °C for 30 min. The reaction crude was allowed to cool down to rt and diluted with EtOAc (5 mL) and H_2O (5 mL). The phases were separated, and the aqueous layer was extracted with EtOAc (3 × 10 mL). The combined organic extracts were dried over Na_2SO_4 and filtered. The solvent was removed under reduced pressure. Et_2O (5 mL) and pentane (5 mL) were added and the solvent was removed under reduced pressure to afford pure *trans*-3,4-disubstituted piperidine **10a** as a white solid (220 mg, 97%).

R_f 0.27 (15% acetone/pentane);

mp = 59–63 °C (from Et_2O/pentane);

IR (film)/cm^{-1} 3348 (NH), 2931, 1681 (C=O), 1525, 1514, 1486, 1424, 1366, 1284, 1233, 1158, 1126, 1034, 826, 791, 762;

^{1}H NMR (500 MHz, DMSO-d_6, 373 K) δ 9.69 (br s, 1 H, NH), 8.84 (dd, *J* = 4.2, 1.7 Hz, 1 H, HC_{Ar}), 8.41 (dd, *J* = 7.7, 1.3 Hz, 1 H, HC_{Ar}), 8.31 (dd, *J* = 8.3, 1.7 Hz, 1 H, HC_{Ar}), 7.59–7.54 (m, 2 H, HC_{Ar}), 7.47 (t, *J* = 7.9 Hz, 1 H, HC_{Ar}), 7.26–7.23 (m, 2 H, HC_{Ar}), 6.76–6.63 (m, 2 H, HC_{Ar}), 4.37–4.30 (m, 1 H, NC*H*HCHCO), 4.12 (dddd, *J* = 13.2, 4.4, 2.2, 2.2 Hz, 1 H, NC*H*HCH$_2$), 3.59 (s, 3 H, OCH_3), 3.07–3.01 (m, 3 H, NCH*H*CHCO, CHCO, CHAr), 2.97–2.90 (m, 1 H, NCH*H*CH$_2$), 1.81–1.76 (m, 1 H, NCH$_2$C*H*H), 1.71–1.60 (m, 1 H, NCH$_2$CH*H*), 1.48 (s, 9 H, $C(CH_3)_3$);

^{13}C NMR (126 MHz, DMSO-d_6, 373 K) δ 170.0 (C=O amide), 157.5 (OC_{Ar} quat), 153.4 (C=O carbamate), 148.0 (C_{Ar}), 137.6 (C_{Ar} quat), 135.7 (C_{Ar}), 135.0 (C_{Ar} quat), 133.6 (C_{Ar} quat), 127.7 (2 × C_{Ar}), 127.2 (C_{Ar} quat), 126.1 (C_{Ar}), 121.3 (C_{Ar}), 121.2 (C_{Ar}), 116.1 (C_{Ar}), 113.5 (2 × C_{Ar}), 78.5 (*C*(CH$_3$)$_3$), 54.5 (OCH_3), 49.8 (*C*HCO), 46.3 (N*C*H$_2$CHCO), 43.6 (CHAr), 43.5 (N*C*H$_2$CH$_2$), 32.4 (NCH$_2$*C*H$_2$), 27.7 (C(*C*H$_3$)$_3$);

HRMS (ESI$^+$) *m/z* Calculated for $C_{27}H_{32}N_3O_4$ [M+H] 462.2393; Found 462.2403.

Characterisation information consistent with data previously reported in literature.[24a]

trans-(±)-tert-Butyl 4-(4-fluorophenyl)-3-(quinolin-8-ylcarbamoyl)piperidine-1-carboxylate (10b)

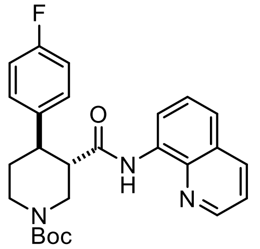

A large microwave vial (10–20 mL recommended volume) was charged with K_2CO_3 (550 mg, 4.00 mmol), flame-dried, and allowed to cool under argon. Amide **8** (1.42 g, 4.00 mmol), 4-fluoroiodobenzene (2.66 g, 12.0 mmol), $Pd(OAc)_2$ (45.0 mg, 0.20 mmol) and PivOH (410 mg, 4.00 mmol) were added sequentially. The reaction vessel purged with argon, then $PhCF_3$ (2.0 mL, 2.0 M) was added by syringe. The reaction tube was placed in a preheated oil bath and stirred at 110 °C for 18 h. The reaction mixture was then allowed to cool to rt and $PhCF_3$ (2.0 mL, 1.0 M) and DBU (1.8 mL, 12.0 mmol, 3 equiv) were added by syringe. The reaction vessel was then stirred at 110 °C for additional 18 h. The reaction mixture was allowed to cool to rt and EtOAc (20 mL) was added. The resulting mixture was filtered through a pad of Celite, eluting with further EtOAc (2 × 50 mL). The solvent was removed under reduced pressure, and the crude material was purified by flash column chromatography (55:40:0.5 to 50:40:10, hexane:Et_2O:CH_2Cl_2). The product containing fractions were combined and the solvent was removed under reduced pressure. Et_2O (10 mL) and pentane (10 mL) were added and the solvent was removed under reduced pressure to afford the *trans*-arylated product **10b** as a pale yellow solid (954 mg, 53%).

R_f 0.27 (50:40:10, hexane:Et_2O:CH_2Cl_2).

mp = 62–68 °C (from Et_2O/pentane);

IR (film)/cm^{-1} 3345 (NH), 2976, 1681 (C=O), 1525, 1486, 1424, 1389, 1366, 1325, 1227, 1158, 1124, 826, 791, 760;

^{1}H NMR (400 MHz, DMSO-*d*$_6$, 373 K) δ 9.71 (br s, 1 H, NH), 8.84 (dd, *J* = 4.2, 1.7 Hz, 1 H, HC$_{Ar}$), 8.39 (dd, *J* = 7.6, 1.3 Hz, 1 H, HC$_{Ar}$), 8.31 (dd, *J* = 8.3, 1.7 Hz, 1 H, HC$_{Ar}$), 7.62–7.52 (m, 2 H, HC$_{Ar}$), 7.47 (t, *J* = 8.0 Hz, 1 H, HC$_{Ar}$), 7.43–7.32 (m, 2 H, HC$_{Ar}$), 7.02–6.91 (m, 2 H, HC$_{Ar}$), 4.40–4.31 (m, 1 H, NC*H*HCHCO), 4.13 (dddd, *J* = 13.3, 4.4, 2.2, 2.2 Hz, 1 H, NC*H*HCH$_2$), 3.15–2.99 (m, 3 H, NCH*H*CHCO, CHCO, CHAr), 2.99–2.90 (m, 1 H, NCH*H*CH$_2$), 1.81 (dddd, *J* = 13.8, 3.2, 3.2, 3.2 Hz, 1 H, NCH$_2$C*H*H), 1.73–1.61 (m, 1 H, NCH$_2$CH*H*), 1.49 (s, 9 H, C(CH$_3$)$_3$);

^{13}C NMR (101 MHz, DMSO-*d*$_6$, 373 K) δ 169.9 (C=O amide), 160.4 (d, J_{CF} = 242.2 Hz, FC$_{Ar}$ quat), 153.4 (C=O carbamate), 148.0 (C$_{Ar}$), 139.0 (d, J_{CF} = 3.2 Hz, F-*p*-C$_{Ar}$ quat), 137.7 (C$_{Ar}$ quat), 135.7 (C$_{Ar}$), 133.5 (C$_{Ar}$ quat), 128.6 (d, J_{CF} = 8.0 Hz, 2 × F-*m*-C$_{Ar}$), 127.2 (C$_{Ar}$ quat), 126.1 (C$_{Ar}$), 121.4 (C$_{Ar}$), 121.3 (C$_{Ar}$), 116.3 (C$_{Ar}$), 114.3 (d, J_{CF} = 21.0 Hz, 2 × F-*o*-C$_{Ar}$), 78.6 (*C*(CH$_3$)$_3$), 49.6 (*C*HCO), 46.2 (N*C*H$_2$CHCO), 43.7 (CHAr), 43.4 (N*C*H$_2$CH$_2$), 32.2 (NCH$_2$*C*H$_2$), 27.7 (C(*C*H$_3$)$_3$);

^{19}F NMR (376 MHz, DMSO-*d*$_6$, 373 K) δ – 16.8 (br s);

HRMS (ESI$^+$) *m/z* Calculated for $C_{26}H_{29}N_3O_3F$ [M+H] 450.2193; Found 450.2216.

Characterisation information consistent with data previously reported in literature.[24a]

3,4-Disubstituted Fragment Synthesis

cis-(±)-1-(tert-Butoxycarbonyl)-4-(4-methoxyphenyl)pyrrolidine-3-carboxylic acid (27a)

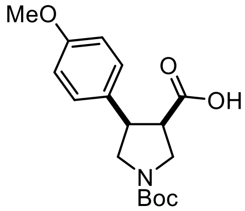

A flame-dried reaction tube was charged with *cis*-arylated amide **6a** (327 mg, 0.75 mmol), followed by Boc$_2$O (818 mg, 3.75 mmol) and DMAP (183 mg, 1.50 mmol). The reaction vessel was sealed and purged with argon, then anhydrous MeCN (1.5 mL, 0.5 M) was added by syringe. The reaction tube was placed in an oil bath and gradually warmed up to 55 °C. The reaction mixture was then stirred at 55 °C for 1 h. The reaction mixture was allowed to cool to rt and sat. aq. NH$_4$Cl (3 mL) and CH$_2$Cl$_2$ (3 mL) were added. The phases were separated, and the aqueous layer was extracted with CH$_2$Cl$_2$ (3 × 5 mL). The combined organic extracts were dried over Na$_2$SO$_4$ and filtered. The solvent was removed under reduced pressure to afford the crude *N*-Boc protected pyrrolidine derivative **25a**, which was used in the next step without further purification. A solution of H$_2$O$_2$ (30 wt.% in H$_2$O, 383 μL, 3.75 mmol) in THF (1.8 mL) was added to a solution of LiOH·H$_2$O (63.0 mg, 1.50 mmol) in H$_2$O (1.9 mL) at 0 °C under argon. The resulting mixture was added dropwise to a solution of crude **25a** in THF (3.8 mL) at 0 °C under argon. The reaction was then stirred at 20 °C for 1 h. Sat. aq. Na$_2$S$_2$O$_3$ (5 mL), EtOAc (5 mL) and 1 N NaOH (5 mL) were then added. The aqueous alkaline layer was collected, and the organic layer extracted with 1 N NaOH (3 × 15 mL). All the aqueous alkaline layers were combined, washed with EtOAc (40 mL), and acidified to pH ~ 1 with HCl, 37%. The acidic layer was extracted with EtOAc (5 × 50 mL). The combined organic extracts were dried over Na$_2$SO$_4$ and filtered. Solvent was removed under reduced pressure. Et$_2$O (5 mL) and pentane (5 mL) were added and the solvent was removed under reduced pressure to afford the pure *cis*-acid **27a** as a white solid (214 mg, 89% over 2 steps).

R$_f$ 0.22 (30% acetone/pentane).

mp = 137–141 °C (from Et$_2$O/pentane).

IR (film)/cm^{-1} 2971 (OH br), 1730 (C=O), 1629 (C=O), 1513, 1431, 1405, 1245, 1163, 1126, 1021, 869, 835, 775.

^{1}H NMR (400 MHz, $CDCl_3$, 298 K, observed as mixture of rotamers) δ 7.15–7.07 (m, 2 H, HC_{Ar}), 6.85–6.79 (m, 2 H, HC_{Ar}), 3.84–3.71 (m, 6 H, NC*H*$_2$CHAr, NC*H*HCHCO, OCH_3), 3.71–3.59 (m, 2 H, NCH*H*CHCO, CHAr), 3.42–3.29 (m, 1 H, CHCO), 1.50 and 1.49 (2 × s, 9 H, $C(CH_3)_3$).

^{13}C NMR (101 MHz, $CDCl_3$, 298 K, observed as mixture of rotamers) δ 176.1 and 175.8 (COOH), 158.7 (OC_{Ar} quat), 154.53 and 154.47 (C=O carbamate), 130.2 and 129.9 (C_{Ar} quat), 128.6 (2 × C_{Ar}), 113.9 (2 × C_{Ar}), 79.9 (*C*$(CH_3)_3$), 55.1 (OCH_3), 50.64 and 50.57 (N*C*H_2CHAr), 48.6 and 47.8 (*C*HCO), 46.9 and 46.7 (N*C*H_2CHCO), 45.4 and 44.5 (CHAr), 28.5 (C(*C*H_3)$_3$).

HRMS (ESI$^-$) *m/z* Calculated for $C_{17}H_{22}NO_5$ [M–H] 320.1498; Found 320.1500.

cis-(±)-1-(_tert_-Butoxycarbonyl)-4-(4-fluorophenyl)pyrrolidine-3-carboxylic acid (27b)

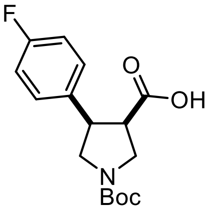

A flame-dried reaction tube was charged with *cis*-arylated amide **6b** (283 mg, 0.65 mmol), followed by Boc_2O (709 mg, 3.25 mmol) and DMAP (159 mg, 1.30 mmol). The reaction vessel was sealed and purged with argon, then anhydrous MeCN (1.3 mL, 0.5 M) was added by syringe. The reaction tube was placed in an oil bath and gradually warmed up to 55 °C. The reaction mixture was then stirred at 55 °C for 1 h. The reaction mixture was allowed to cool to rt and sat. aq. NH_4Cl (3 mL) and CH_2Cl_2 (3 mL) were added. The phases were separated, and the aqueous layer was extracted with CH_2Cl_2 (3 × 5 mL). The combined organic extracts were dried over Na_2SO_4 and filtered. The solvent was removed under reduced pressure to afford the crude *N*-Boc protected pyrrolidine derivative. A solution of H_2O_2 (30 wt.% in H_2O, 332 μL, 3.25 mmol) in THF (1.5 mL) was added to a solution of LiOH·H_2O (55.0 mg, 1.30 mmol) in H_2O (1.6 mL) at 0 °C under argon. The resulting mixture was added dropwise to a solution of crude *N*-Boc protected pyrrolidine in THF (3.4 mL) at 0 °C under argon. The reaction was then stirred at 20 °C for 1 h. Sat. aq. $Na_2S_2O_3$ (5 mL), EtOAc (5 mL) and 1 N NaOH (5 mL) were then added. The aqueous alkaline layer was collected, and the organic layer extracted with 1 N NaOH (3 × 15 mL). All the aqueous alkaline layers were combined, washed with EtOAc (40 mL), and acidified to pH ~ 1 with HCl, 37%. The acidic layer was extracted with EtOAc (5 × 50 mL). The combined organic extracts were dried over Na_2SO_4 and filtered. Solvent was removed under reduced pressure. Et_2O (5 mL) and pentane (5 mL) were added and the solvent was removed under reduced pressure to afford the pure *cis*-acid **27b** as a white solid (192 mg, 95% over 2 steps).

R_f 0.28 (20% acetone/pentane).

mp = 125–128 °C (from Et_2O/pentane).

IR (film)/cm^{-1} 2974 (OH br), 1730 (C=O), 1633 (C=O), 1510, 1428, 1204, 1129, 1156, 872.

^{1}H NMR (400 MHz, $CDCl_3$, 298 K, observed as a mixture of rotamers) δ 7.18–7.07 (m, 2 H, HC_{Ar}), 7.00–6.90 (m, 2 H, HC_{Ar}), 3.80–3.58 (m, 5 H, NC*H*$_2$CHAr, NC*H*$_2$CHCO, CHAr), 3.36 (m, 1 H, CHCO), 1.49 and 1.48 (2 × s, 9 H, $C(CH_3)_3$).

^{13}C NMR (101 MHz, $CDCl_3$, 298 K, observed as a mixture of rotamers) δ 175.9 and 175.7 (COOH), 161.9 (d, J_{CF} = 245.9 Hz, FC_{Ar} quat), 154.5 (C=O carbamate), 134.0 and 133.8 (F-*p*-C_{Ar} quat, observed as singlets), 129.1 (d, J_{CF} = 8.0 Hz, 2 × F-*m*-C_{Ar}), 115.4 (d, J_{CF} = 21.3 Hz, 2 × F-*o*-C_{Ar}), 80.2 (*C*(CH_3)$_3$), 50.62 and 50.55 (N*C*H_2CHAr), 48.4 and 47.6 (*C*HCO), 46.7 and 46.6 (N*C*H_2CHCO), 45.3 and 44.4 (CHAr), 28.4 (C(*C*H_3)$_3$).

^{19}F NMR (377 MHz, $CDCl_3$, 298 K, observed as a mixture of rotamers) δ –115.0 (m).

HRMS (ESI$^-$) *m/z* Calculated for $C_{16}H_{19}NO_4F$ [M–H] 308.1298; Found 308.1304.

trans-(±)-1-(tert-Butoxycarbonyl)-4-(4-methoxyphenyl)pyrrolidine-3-carboxylic acid (24a)

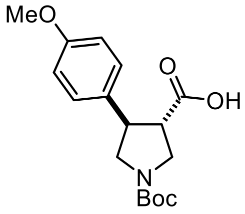

Prepared according to literature procedure,[24a] a round-bottom-flask was charged with *cis*-arylated amide **6a** (290 mg, 0.65 mmol) and EtOH (3.3 mL, 0.2 M). NaOH (powder, 263 mg, 6.50 mmol) was then added and the resulting mixture was stirred at 100 °C for 30 min. The reaction crude was allowed to cool down to rt and diluted with EtOAc (5 mL) and 1 N NaOH (5 mL). The aqueous alkaline layer was collected, and the organic layer extracted with 1 N NaOH (3 × 10 mL). All the aqueous alkaline layers were combined, washed with EtOAc (30 mL), and acidified to pH ~ 1 with HCl, 37%. The acidic layer was extracted with EtOAc (5 × 40 mL). The combined organic extracts were dried over Na_2SO_4 and filtered. Solvent was removed under reduced pressure. Et_2O (5 mL) and pentane (5 mL) were added and the solvent was removed under reduced pressure to afford the pure *trans*-acid **24a** as a pale orange solid (172 mg, 82%).

R_f 0.19 (80:20:0.5 acetone/pentane/TFA).

mp = 144–148 °C (from Et_2O/pentane).

IR (film)/cm^{-1} 3101 (OH br), 2979, 1727 (C=O), 1662 (C=O), 1517, 1435, 1408, 1369, 1253, 1165, 1136, 1030, 883, 817, 765, 666.

^{1}H NMR (400 MHz, $CDCl_3$, 298 K, observed as mixture of rotamers) δ 7.17 (d, *J* = 8.6 Hz, 2 H, HC_{Ar}), 6.86 (d, *J* = 8.5 Hz, 2 H, HC_{Ar}), 3.94–3.74 (m, 5 H, NC*H*HCHAr, NC*H*HCHCO, OCH_3), 3.66–3.53 (m, 2 H, NCH*H*CHCO, CHAr), 3.44–3.28 (m, 1 H, NCH*H*CHAr), 3.22–3.09 (m, 1 H, CHCO), 1.48 and 1.46 (2 × s, 9 H, C(CH_3)$_3$).

^{13}C NMR (101 MHz, $CDCl_3$, 298 K, observed as mixture of rotamers) δ 177.2 (COOH), 158.8 (OC_{Ar} quat), 154.1 (C=O carbamate), 130.8 and 130.7 (C_{Ar} quat), 128.3 (2 × C_{Ar}), 114.2 (2 × C_{Ar}), 80.0 (*C*(CH_3)$_3$), 55.2 (OCH_3), 52.9 and 52.2 (N*C*H_2CHAr), 50.4 and 49.4 (*C*HCO), 48.7 and 48.5 (N*C*H_2CHCO), 47.0 and 46.2 (CHAr), 28.4 (C(*C*H_3)$_3$).

HRMS (ESI$^+$) *m/z* Calculated for $C_{17}H_{24}NO_5$ [M+H] 322.1654; Found 322.1642.

Characterisation information consistent with data previously reported in literature.[24a]

trans-(±)-1-(tert-Butoxycarbonyl)-4-(4-fluorophenyl)pyrrolidine-3-carboxylic acid (24b)

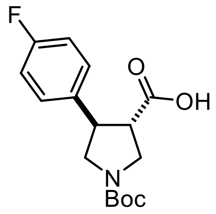

A round-bottom-flask was charged with *cis*-arylated amide **6b** (310 mg, 0.71 mmol) and EtOH (3.5 mL, 0.2 M). NaOH (powder, 288 mg, 7.10 mmol) was then added and the resulting mixture was stirred at 100 °C for 30 min. The reaction crude was allowed to cool down to rt and diluted with EtOAc (5 mL) and 1 N NaOH (5 mL). The aqueous alkaline layer was collected, and the organic layer extracted with 1 N NaOH (3 × 10 mL). All the aqueous alkaline layers were combined, washed with EtOAc (30 mL), and acidified to pH ~ 1 with HCl, 37%. The acidic layer was extracted with EtOAc (5 × 40 mL). The combined organic extracts were dried over Na_2SO_4 and filtered. Solvent was removed under reduced pressure. Et_2O (5 mL) and pentane (5 mL) were added and the solvent was removed under reduced pressure to afford the pure *trans*-acid **24b** as a pale yellow solid (210 mg, 96%).

R_f 0.12 (5% MeOH/CH_2Cl_2).

mp = decomposed at T >120 °C.

IR (film)/cm^{-1} 2978 (OH br), 1726 (C=O), 1655 (C=O), 1513, 1420, 1226, 1141, 835.

^{1}H NMR (500 MHz, DMSO-d_6, 373 K) δ 7.38–7.33 (m, 2 H, HC_{Ar}), 7.14–7.08 (m, 2 H, HC_{Ar}), 3.78 (dd, J = 10.5, 7.8 Hz, 1 H, NC*H*HCHAr), 3.74 (dd, J = 10.6, 8.2 Hz, 1 H, NC*H*HCHCO), 3.58 (ddd, J = 9.3, 9.3, 7.7 Hz, 1 H, CHAr), 3.48 (dd, J = 10.8, 8.5 Hz, 1 H, NCH*H*CHCO), 3.26 (dd, J = 10.6, 9.2 Hz, 1 H, NCH*H*CHAr), 3.19 (ddd, J = 9.6, 8.4, 8.4 Hz, 1 H, CHCO), 1.44 (s, 9 H, $C(CH_3)_3$).

^{13}C NMR (126 MHz, DMSO-d_6, 373 K) δ 172.4 (COOH), 160.8 (d, J_{CF} = 243.1 Hz, FC_{Ar} quat), 152.8 (C=O carbamate), 135.7 (d, J_{CF} = 3.1 Hz, F-*p*-C_{Ar} quat), 128.6 (d, J_{CF} = 8.1 Hz, 2 × F-*m*-C_{Ar}), 114.5 (d, J_{CF} = 21.3 Hz, 2 × F-*o*-C_{Ar}), 78.2 (*C*$(CH_3)_3$), 52.0 (N*C*H_2CHAr), 48.7 (*C*HCO), 48.0 (N*C*H_2CHCO), 45.6 (CHAr), 27.7 (C(*C*$H_3)_3$).

^{19}F NMR (471 MHz, DMSO-d_6, 373 K) δ –116.2 (tt, J_{HF} = 9.3, 5.5 Hz).

HRMS (FTMS – pAPCI) *m/z* Calculated for $C_{16}H_{19}NO_4F^-$ $[M–H]^-$ 308.1293; Found 308.1301.

cis-(±)-tert-Butyl 3-carbamoyl-4-(4-fluorophenyl)pyrrolidine-1-carboxylate (30b)

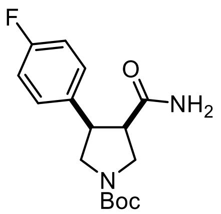

According to the procedure reported by Zhang and Chen,[28a] a round-bottom flask was charged with *cis*-arylated pyrrolidine **6b** (327 mg, 0.75 mmol) and IBX (30 wt.%, 1.40 g, 1.50 mmol) under air. H_2O (2.5 mL) and HFIP (2.5 mL) were then added, and the resulting solution (0.15 M) was stirred at 60 °C for 1.5 h. The reaction mixture was then allowed to cool to rt and sat. aq. $NaHCO_3$ (10 mL) and CH_2Cl_2 (10 ml) were added. The phases were separated, and the aqueous layer was extracted with CH_2Cl_2 (3 × 15 mL). The combined organic extracts

were dried over Na_2SO_4 and filtered. The solvent was removed under reduced pressure. The crude material was purified by flash column chromatography (35% acetone/pentane). The product containing fractions were combined and the solvent was removed under reduced pressure. Et_2O (5 mL) and pentane (5 mL) were added and the solvent was removed under reduced pressure to afford the primary amide **30b** as a pale yellow solid (179 mg, 77%).

R_f 0.33 (40% acetone/pentane).

mp = 73–76 °C (from Et_2O/pentane).

IR (film)/cm^{-1} 3317 (NH br), 3194 (NH br), 2974, 1659 (C=O), 1513, 1394, 1223, 1159, 1118, 835.

^{1}H NMR (400 MHz, $CDCl_3$, 298 K, observed as a mixture of rotamers) δ 7.25–7.14 (m, 2 H, HC_{Ar}), 7.03–6.94 (m, 2 H, HC_{Ar}), 5.53–5.32 (br m, 2 H, NH_2), 3.83–3.70 (m, 3 H, NC*H*$_2$CHAr, NC*H*HCHCO), 3.67–3.56 (m, 2 H, NCH*H*CHCO, CHAr), 3.21–3.09 (m, 1 H, CHCO), 1.48 (s, 9 H, $C(CH_3)_3$).

^{13}C NMR (101 MHz, $CDCl_3$, 298 K, observed as a mixture of rotamers) δ 172.2 and 173.0 (C=O amide), 161.7 (d, J_{CF} = 245.9 Hz, FC_{Ar} quat), 154.39 and 154.37 (C=O carbamate), 133.6 and 133.3 (F-*p*-C_{Ar} quat, observed as singlets), 129.4–129.2 (2 × F-*m*-C_{Ar}, observed as triplet), 115.4 (d, J_{CF} = 21.3 Hz, 2 × F-*o*-C_{Ar}), 79.8 (*C*$(CH_3)_3$), 50.0 (N*C*H_2CHAr), 49.5 and 48.7 (*C*HCO), 47.8 and 47.7 (N*C*H_2CHCO), 46.1 and 45.3 (CHAr), 28.5 (C(*C*$H_3)_3$).

^{19}F NMR (377 MHz, $CDCl_3$, 298 K) δ –115.0 (tt, J_{HF} = 8.5, 4.3 Hz).

HRMS (ESI$^+$) *m/z* Calculated for $C_{16}H_{21}N_2O_3FNa$ [M+Na] 331.1434; Found 331.1436.

trans-(±)-tert-Butyl 3-carbamoyl-4-(4-methoxyphenyl)pyrrolidine-1-carboxylate (32a)

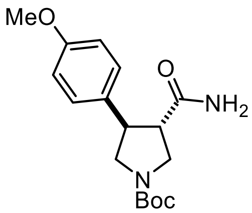

According to the procedure reported by Zhang and Chen,[28a] a round-bottom flask was charged with *trans*-arylated pyrrolidine **7a** (356 mg, 0.80 mmol) and IBX (30 wt.%, 1.50 g, 1.60 mmol) under air. H_2O (2.7 mL) and HFIP (2.7 mL) were then added, and the resulting solution (0.15 M) was stirred at 60 °C for 1.5 h. The reaction mixture was then allowed to cool to rt and sat. aq. $NaHCO_3$ (10 mL) and CH_2Cl_2 (10 ml) were added. The phases were separated, and the aqueous layer was extracted with CH_2Cl_2 (3 × 15 mL). The combined organic extracts were dried over Na_2SO_4 and filtered. The solvent was removed under reduced pressure. The crude material was purified by flash column chromatography (40% acetone/pentane). The product containing fractions were combined and the solvent was removed under reduced pressure. Et_2O (5 mL) and pentane (5 mL) were added and the solvent was removed under reduced pressure to afford the primary amide **32a** as a pale yellow solid (203 mg, 79%).

R_f 0.28 (40% acetone/pentane).

mp = 147–150 °C (from Et_2O/pentane).

IR (film)/cm^{-1} 3403 (NH br), 3168 (NH br), 2967, 1692 (C=O), 1655 (C=O), 1405, 1179, 1129, 828.

^{1}H NMR (400 MHz, $CDCl_3$, 298 K, observed as a mixture of rotamers) δ 7.20 (d, *J* = 8.3 Hz, 2 H, HC_{Ar}), 6.88 (d, *J* = 8.4 Hz, 2 H, HC_{Ar}), 5.60 (br s, 1 H, N*H*H), 5.33 and 5.20 (2 × br s, 1 H, NH*H*), 3.96–3.73 (m, 5 H, NC*H*HCHAr, NC*H*HCHCO, OCH_3), 3.72–3.60 (m, 1 H, NCH*H*CHCO), 3.59–3.30 (m, 2 H, CHAr, NCH*H*CHAr), 3.04–2.88 (m, 1 H, CHCO), 1.47 and 1.45 (2 × s, 9 H, $C(CH_3)_3$).

^{13}C NMR (101 MHz, $CDCl_3$, 298 K, observed as a mixture of rotamers) δ 173.1 (C=O amide), 159.0 and 158.9 (OC_{Ar} quat), 154.2 and 154.1 (C=O carbamate), 130.9 and 130.6 (C_{Ar} quat), 128.5 and 128.4 (2 × C_{Ar}), 114.5 and 114.4 (2 × C_{Ar}), 79.6 ($\mathit{C}(CH_3)_3$), 55.3 (OCH_3), 53.2 and 52.8 (N*C*H_2CHAr), 51.9 and 51.1 (*C*HCO), 48.7 and 48.6 (N*C*H_2CHCO), 47.6 and 47.1 (CHAr), 28.4 ($C(\mathit{C}H_3)_3$).

HRMS (ESI$^+$) *m/z* Calculated for $C_{17}H_{24}N_2O_4Na$ [M+Na] 343.1634; Found 343.1625.

trans-(±)-_tert_-Butyl 3-carbamoyl-4-(4-fluorophenyl)pyrrolidine-1-carboxylate (32b)

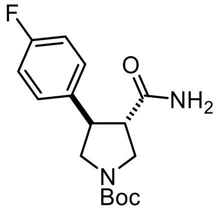

According to the procedure reported by Zhang and Chen,[28a] a round-bottom flask was charged with *trans*-arylated pyrrolidine **7b** (220 mg, 0.50 mmol) and IBX (30 wt.%, 940 mg, 1.00 mmol) under air. H_2O (1.7 mL) and HFIP (1.7 mL) were then added, and the resulting solution (0.15 M) was stirred at 60 °C for 1.5 h. The reaction mixture was then allowed to cool to rt and sat. aq. $NaHCO_3$ (10 mL) and CH_2Cl_2 (10 ml) were added. The phases were separated, and the aqueous layer was extracted with CH_2Cl_2 (3 × 15 mL). The combined organic extracts were dried over Na_2SO_4 and filtered. The solvent was removed under reduced pressure. The crude material was purified by flash column chromatography (35% acetone/pentane). The product containing fractions were combined and the solvent was removed under reduced pressure. Et_2O (5 mL) and pentane (5 mL) were added and the solvent was removed under reduced pressure to afford the primary amide **32b** as a white solid (114 mg, 74%).

R_f 0.35 (40% acetone/pentane);

mp = 162–165 °C (from Et_2O/pentane);

IR (film)/cm^{-1} 3388 (NH br), 3198 (NH br), 2967, 1662 (C=O), 1513, 1402, 1219, 1174, 1126, 831;

^{1}H NMR (400 MHz, $CDCl_3$, 298 K, observed as a mixture of rotamers) δ 7.28–7.22 (m, 2 H, HC_{Ar}), 7.07–7.00 (m, 2 H, HC_{Ar}), 5.59 (br s, 1 H, N*H*H), 3.59 and 5.24 (2 × br s, 1 H, NH*H*), 3.99–3.75 (m, 2 H, NC*H*HCHAr, NC*H*HCHCO), 3.71–3.49 (m, 2 H, NCH*H*CHCO, CHAr), 3.44–3.31 (m, 1 H, NCH*H*CHAr), 3.02–2.87 (m, 1 H, CHCO), 1.47 and 1.46 (2 × s, 9 H, $C(CH_3)_3$);

^{13}C NMR (101 MHz, $CDCl_3$, 298 K, observed as a mixture of rotamers) δ 172.8 (C=O amide), 162.1 (d, J_{CF} = 244.5 Hz, FC_{Ar} quat), 154.1 (C=O carbamate), 134.8 and 134.6 (F-*p*-C_{Ar} quat, observed as singlets), 129.0 (d, J_{CF} = 6.9 Hz, 2 × F-*m*-C_{Ar}), 115.94 and 115.85 (2 × d, J_{CF} = 21.6 Hz, 2 × F-*o*-C_{Ar}), 79.8 ($\mathit{C}(CH_3)_3$), 52.9 and 52.5 (N*C*H_2CHAr), 52.1 and 51.2 (*C*HCO), 49.0 and 48.9 (N*C*H_2CHCO), 47.4 and 46.9 (CHAr), 28.4 ($C(\mathit{C}H_3)_3$);

^{19}F NMR (377 MHz, $CDCl_3$, 298 K, observed as a mixture of rotamers) δ –114.2 and –114.7 (2 × m);

HRMS (ESI$^+$) *m/z* Calculated for $C_{18}H_{24}N_3O_3FNa$ [M+MeCN+Na] 372.1699; Found 372.1699.

cis-(±)-tert-Butyl 3-(hydroxymethyl)-4-(4-methoxyphenyl)pyrrolidine-1-carboxylate (28a)

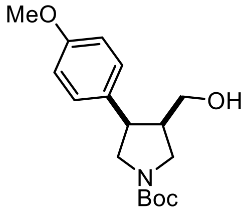

Prepared according to literature procedure,[24a] a flame-dried reaction tube was charged with *cis*-arylated amide **6a** (340 mg, 0.76 mmol), followed by Boc_2O (829 mg, 3.80 mmol) and DMAP (186 mg, 1.52 mmol). The reaction vessel was sealed and purged with argon, then anhydrous MeCN (1.5 mL, 0.5 M) was added by syringe. The reaction tube was placed in an oil bath and gradually warmed up to 55 ºC. The reaction mixture was then stirred at 55 °C for 1 h. The reaction mixture was allowed to cool to rt and sat. aq. NH_4Cl (3 mL) and CH_2Cl_2 (3 mL) were added. The phases were separated, and the aqueous layer was extracted with CH_2Cl_2 (3 × 5 mL). The combined organic extracts were dried over Na_2SO_4 and filtered. The solvent was removed under reduced pressure to afford the crude *N*-Boc protected pyrrolidine derivative. A solution of the crude *N*-Boc protected pyrrolidine derivative in dry THF (2.5 mL) was added dropwise to a suspension of $LiAlH_4$ (115 mg, 3.04 mmol) in dry THF (1.5 mL) at 0 °C under argon atmosphere. The mixture was then stirred at 20 °C for 30 min. The reaction mixture was quenched by slow addition of sat. aq. NH_4Cl (2 mL) at 0 °C and stirred at rt for 30 min. The resulting suspension was filtered through a pad of Celite, eluting with EtOAc (3 × 5 mL). The phases were separated, and the aqueous layer was extracted with EtOAc (3 × 5 mL). The combined organic extracts were dried over Na_2SO_4 and filtered. The solvent was removed under reduced pressure. The crude material was purified by flash column chromatography (20% acetone/pentane). The product containing fractions were combined and the solvent was removed under reduced pressure. Et_2O (5 mL) and pentane (5 mL) were added and the solvent was removed under reduced pressure to afford the *cis*-alcohol derivative **28a** as a colourless paste (192 mg, 82% over 2 steps).

R_f 0.28 (20% acetone/pentane);

IR (film)/cm^{-1} 3407 (OH br), 2935, 1683 (C=O), 1513, 1401, 1365, 1246, 1162, 1124, 1027, 876, 826, 772;

^{1}H NMR (400 MHz, DMSO-d_6, 373 K) δ 7.10–7.05 (m, 2 H, HC_{Ar}), 6.90–6.85 (m, 2 H, HC_{Ar}), 4.08 (t, *J* = 4.9 Hz, 1 H, OH), 3.75 (s, 3 H, OCH_3), 3.62 (dd, *J* = 10.7, 7.2 Hz, 1 H, NC*H*HCHAr), 3.51 (dd, *J* = 10.7, 5.8 Hz, 1 H, NCH*H*CHAr), 3.49–3.40 (m, 2 H, NC*H*HCHCH$_2$OH, CHAr), 3.25 (dd, *J* = 10.8, 6.6 Hz, 1 H, NCH*H*CHCH$_2$OH), 3.14 (dt, *J* = 10.2, 5.1 Hz, 1 H, C*H*HOH), 3.03–2.95 (m, 1 H, CH*H*OH), 2.57–2.47 (m, 1 H, C*H*CH$_2$OH), 1.46 (s, 9 H, $C(CH_3)_3$);

^{13}C NMR (101 MHz, DMSO-d_6, 373 K) δ 157.6 (OC_{Ar} quat), 153.3 (C=O carbamate), 131.2 (C_{Ar} quat), 128.1 (2 × C_{Ar}), 113.4 (2 × C_{Ar}), 77.8 (*C*(CH$_3$)$_3$), 59.6 (*C*H$_2$OH), 54.7 (OCH_3), 50.2 (N*C*H$_2$CHAr), 47.6 (N*C*H$_2$CHCH$_2$OH), 44.1 (*C*HCH$_2$OH), 43.2 (CHAr), 27.8 (C(*C*H$_3$)$_3$);

HRMS (ESI$^+$) *m/z* Calculated for $C_{17}H_{26}NO_4$ [M+H] 308.1862; Found 308.1857.

Characterisation information consistent with data previously reported in literature.[24a]

cis-(±)-tert-Butyl 3-(hydroxymethyl)-4-(4-fluorophenyl)pyrrolidine-1-carboxylate (28b)

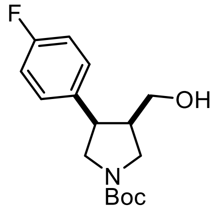

A flame-dried reaction tube was charged with *cis*-arylated amide **6b** (261 mg, 0.60 mmol), followed by Boc_2O (655 mg, 3.00 mmol) and DMAP (147 mg, 1.20 mmol). The reaction vessel was sealed and purged with argon, then anhydrous MeCN (1.2 mL, 0.5 M) was added by syringe. The reaction tube was placed in an oil bath and gradually warmed up to 55 °C. The reaction mixture was then stirred at 55 °C for 1 h. The reaction mixture was allowed to cool to rt and sat. aq. NH_4Cl (3 mL) and CH_2Cl_2 (3 mL) were added. The phases were separated, and the aqueous layer was extracted with CH_2Cl_2 (3 × 5 mL). The combined organic extracts were dried over Na_2SO_4 and filtered. The solvent was removed under reduced pressure to afford the crude *N*-Boc protected pyrrolidine derivative. A solution of the crude *N*-Boc protected pyrrolidine derivative in dry THF (2.0 mL) was added dropwise to a suspension of $LiAlH_4$ (91.0 mg, 2.40 mmol) in dry THF (1.0 mL) at 0 °C under argon atmosphere. The mixture was then stirred at 20 °C for 30 min. The reaction mixture was quenched by slow addition of sat. aq. NH_4Cl (2 mL) at 0 °C and stirred at rt for 30 min. The resulting suspension was filtered through a pad of Celite, eluting with EtOAc (3 × 5 mL). The phases were separated, and the aqueous layer was extracted with EtOAc (3 × 5 mL). The combined organic extracts were dried over Na_2SO_4 and filtered. The solvent was removed under reduced pressure. The crude material was purified by flash column chromatography (15% to 20% acetone/pentane). The product containing fractions were combined and the solvent was removed under reduced pressure. Et_2O (5 mL) and pentane (5 mL) were added and the solvent was removed under reduced pressure to afford the *cis*-alcohol derivative **28b** as a colourless paste (145 mg, 82% over 2 steps).

R_f 0.12 (15% acetone/pentane).

IR (film)/cm^{-1} 3407 (OH br), 2933, 1670 (C=O), 1510, 1394, 1226, 1156, 1126, 1047, 835;

^{1}H NMR (400 MHz, $CDCl_3$, 298 K, observed as a mixture of rotamers) δ 7.18–7.10 (m, 2 H, HC_{Ar}), 7.05–6.97 (m, 2 H, HC_{Ar}), 3.80–3.56 (m, 3 H, NCH_2CHAr, NC*H*HCHCH$_2$OH), 3.51 (ddd, *J* = 6.9, 6.9, 3.4 Hz, 1 H, CHAr), 3.38–3.21 (m, 3 H, C*H*$_2$OH, NCH*H*CHCH$_2$OH), 2.73–2.58 (m, 1 H, C*H*CH$_2$OH), 1.50 and 1.49 (2 × s, 9 H, $C(CH_3)_3$);

^{13}C NMR (101 MHz, $CDCl_3$, 298 K, observed as a mixture of rotamers) δ 161.7 (d, J_{CF} = 245.1 Hz, FC_{Ar} quat), 154.6 (C=O carbamate), 135.2 (F-*p*-C_{Ar} quat, observed as broad singlet), 129.3 and 129.2 (2 × d, J_{CF} = 7.4 Hz, 2 × F-*m*-C_{Ar}), 115.4 (d, J_{CF} = 21.1 Hz, 2 × F-*o*-C_{Ar}), 79.6 (*C*(CH$_3$)$_3$), 61.6 and 61.4 (CH_2OH), 51.5 and 50.9 (N*C*H$_2$CHAr), 47.6 and 47.2 (N*C*H$_2$CHCH$_2$OH), 45.2 and 44.5 (*C*HCH$_2$OH), 44.4 and 43.6 (CHAr), 28.5 (C(*C*H$_3$)$_3$);

^{19}F NMR (377 MHz, $CDCl_3$, 298 K, observed as a mixture of rotamers) δ –115.9 (m);

HRMS (ESI$^+$) *m/z* Calculated for $C_{16}H_{23}NO_3F$ [M+H] 296.1662; Found 296.1663.

trans-(±)-tert-Butyl 3-(hydroxymethyl)-4-(4-methoxyphenyl)pyrrolidine-1-carboxylate (29a)

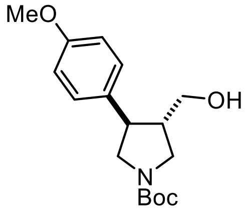

A flame-dried reaction tube was charged with *trans*-arylated amide **7a** (323 mg, 0.72 mmol), followed Boc_2O (786 mg, 3.60 mmol) and DMAP (176 mg, 1.44 mmol). The reaction vessel was sealed and purged with argon, then anhydrous MeCN (1.4 mL, 0.5 M) was added by syringe. The reaction tube was placed in an oil bath and gradually warmed up to 55 °C. The reaction mixture was then stirred at 55 °C for 1 h. The reaction mixture was allowed to cool to rt and sat. aq. NH_4Cl (3 mL) and CH_2Cl_2 (3 mL) were added. The phases were separated, and the aqueous layer was extracted with CH_2Cl_2 (3 × 5 mL). The combined organic extracts were dried over Na_2SO_4 and filtered. The solvent was removed under reduced pressure to afford the crude *N*-Boc protected pyrrolidine derivative. A solution of the crude *N*-Boc protected pyrrolidine derivative in dry THF (2.3 mL) was added dropwise to a suspension of $LiAlH_4$ (109 mg, 2.88 mmol) in dry THF (1.3 mL) at 0 °C under argon atmosphere. The mixture was then stirred at 20 °C for 30 min. The reaction mixture was quenched by slow addition of sat. aq. NH_4Cl (2 mL) at 0 °C and stirred at rt for 30 min. The resulting suspension was filtered through a pad of Celite, eluting with EtOAc (3 × 5 mL). The phases were separated, and the aqueous layer was extracted with EtOAc (3 × 5 mL). The combined organic extracts were dried over Na_2SO_4 and filtered. The solvent was removed under reduced pressure. The crude material was purified by flash column chromatography (15% to 20% acetone/pentane). The product containing fractions were combined and the solvent was removed under reduced pressure. Et_2O (5 mL) and pentane (5 mL) were added and the solvent was removed under reduced pressure to afford the *trans*-alcohol derivative **29a** as a colourless paste (198 mg, 89% over 2 steps).

R_f 0.28 (20% acetone/pentane);

IR (film)/cm^{-1} 3470 (OH br), 2930, 1685 (C=O), 1513, 1416, 1245, 1170, 1133, 1033, 826, 768;

^{1}H NMR (400 MHz, $CDCl_3$, 298 K, observed as a mixture of rotamers) δ 7.20–7.13 (m, 2 H, HC_{Ar}), 6.90–6.83 (m, 2 H, HC_{Ar}), 3.88–3.69 (m, 5 H, OCH_3, NC*H*HCHAr, NC*H*HCHCH$_2$OH), 3.69–3.61 (br m, 1 H, C*H*HOH), 3.57–3.45 (br m, 1 H, CH*H*OH), 3.40–3.20 (m, 2 H, NCH*H*CHCH$_2$OH, NCH*H*CHAr), 3.15–2.98 (m, 1 H, CHAr), 2.51–2.38 (br m, 1 H, C*H*CH$_2$OH), 1.87 (br s, 1 H, OH), 1.48 and 1.45 (2 × s, 9 H, $C(CH_3)_3$);

^{13}C NMR (101 MHz, $CDCl_3$, 298 K, observed as a mixture of rotamers) δ 158.6 (OC_{Ar} quat), 154.5 (C=O carbamate), 131.9 and 131.8 (C_{Ar} quat), 128.5 (2 × C_{Ar}), 114.1 (2 × C_{Ar}), 79.3 (*C*(CH$_3$)$_3$), 62.8 and 62.6 (CH_2OH), 55.2 (OCH_3), 53.6 and 52.9 (N*C*H$_2$CHAr), 49.3 and 48.7 (N*C*H$_2$CHCH$_2$OH), 48.3 and 47.4 (*C*HCH$_2$OH), 45.8 and 45.1 (CHAr), 28.5 (C(*C*H$_3$)$_3$);

HRMS (ESI$^+$) *m/z* Calculated for $C_{17}H_{26}NO_4$ [M+H] 308.1862; Found 308.1854.

trans-(±)-tert-Butyl 3-(hydroxymethyl)-4-(4-fluorophenyl)pyrrolidine-1-carboxylate (29b)

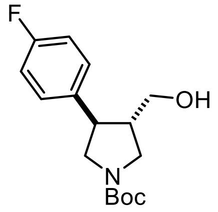

A flame-dried reaction tube was charged with *trans*-arylated amide **7b** (280 mg, 0.64 mmol), followed by Boc_2O (701 mg, 3.21 mmol) and DMAP (156 mg, 1.30 mmol). The reaction vessel was sealed and purged with argon, then anhydrous MeCN (1.3 mL, 0.5 M) was added by syringe. The reaction tube was placed in an oil bath and gradually warmed up to 55 °C. The reaction mixture was then stirred at 55 °C for 1 h. The reaction mixture was allowed to cool to rt and sat. aq. NH_4Cl (3 mL) and CH_2Cl_2 (3 mL) were added. The phases were separated, and the aqueous layer was extracted with CH_2Cl_2 (3 × 5 mL). The combined organic extracts were dried over Na_2SO_4 and filtered. The solvent was removed under reduced pressure to afford the crude *N*-Boc protected pyrrolidine derivative. A solution of the crude *N*-Boc protected pyrrolidine derivative in dry THF (2.1 mL) was added dropwise to a suspension of $LiAlH_4$ (97.2 mg, 2.56 mmol) in dry THF (1.1 mL) at 0 °C under argon atmosphere. The mixture was then stirred at 20 °C for 30 min. The reaction mixture was quenched by slow addition of sat. aq. NH_4Cl (2 mL) at 0 °C and stirred at rt for 30 min. The resulting suspension was filtered through a pad of Celite, eluting with EtOAc (3 × 5 mL). The phases were separated, and the aqueous layer was extracted with EtOAc (3 × 5 mL). The combined organic extracts were dried over Na_2SO_4 and filtered. The solvent was removed under reduced pressure. The crude material was purified by flash column chromatography (15% to 20% acetone/pentane). The product containing fractions were combined and the solvent was removed under reduced pressure. Et_2O (5 mL) and pentane (5 mL) were added and the solvent was removed under reduced pressure to afford the *trans*-alcohol derivative **29b** as a white solid (167 mg, 88% over 2 steps).

R_f 0.11 (15% acetone/pentane).

mp = 101–104 °C (from Et_2O/pentane);

IR (film)/cm^{-1} 3466 (OH br), 2978, 1677 (C=O), 1513, 1416, 1226, 1133, 1074, 880, 831, 768; ^{1}H NMR (400 MHz, $CDCl_3$, 298 K, observed as a mixture of rotamers) δ 7.25–7.18 (m, 2 H, HC_{Ar}), 7.07–6.99 (m, 2 H, HC_{Ar}), 3.92–3.72 (m, 2 H, NC*H*HCHAr, NC*H*HCHCH$_2$OH), 3.68 (dt, *J* = 10.7, 4.6 Hz, 1 H, C*H*HOH), 3.60–3.49 (br m, 1 H, CH*H*OH), 3.42–3.24 (m, 2 H, NCH*H*CHCH$_2$OH, NCH*H*CHAr), 3.22–2.05 (m, 1 H, CHAr), 2.52–2.40 (m, 1 H, C*H*CH$_2$OH), 1.49 and 1.47 (2 × s, 9 H, C(CH$_3$)$_3$), 1.33 (br m, 1 H, OH);

^{13}C NMR (101 MHz, $CDCl_3$, 298 K, observed as a mixture of rotamers) δ 161.9 (d, J_{CF} = 245.5 Hz, FC_{Ar} quat), 154.4 (C=O carbamate), 135.8 (F-*p*-C_{Ar} quat, observed as broad singlet), 129.0 (d, J_{CF} = 7.9 Hz, 2 × F-*m*-C_{Ar}), 115.6 (d, J_{CF} = 21.2 Hz, 2 × F-*o*-C_{Ar}), 79.5 (*C*(CH$_3$)$_3$), 62.6 and 62.4 (CH$_2$OH), 53.5 and 52.8 (N*C*H$_2$CHAr), 49.2 and 48.6 (N*C*H$_2$CHCH$_2$OH), 48.4 and 47.5 (*C*HCH$_2$OH), 45.8 and 45.1 (CHAr), 28.5 (C(*C*H$_3$)$_3$);

^{19}F NMR (377 MHz, $CDCl_3$, 298 K) δ –115.6 (tt, J_{HF} = 8.6, 5.5 Hz);

HRMS (ESI$^+$) *m/z* Calculated $C_{16}H_{23}NO_3F$ [M+H] 296.1662; Found 296.1673.

trans-(±)-tert-Butyl 3-cyano-4-(4-methoxyphenyl)pyrrolidine-1-carboxylate (S1)

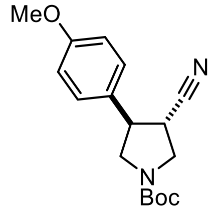

Adapting the procedure reported by Naka,[35] a flame-dried reaction tube was charged with *trans*-amide **32a** (50.0 mg, 0.16 mmol, 1 equiv), $Pd(OAc)_2$ (3.6 mg, 16.0 μmol, 10 mol%), dichloroacetonitrile (128 μL, 1.60 mmol, 10 equiv), dry MeCN (150 μL) and H_2O (150 μL). The reaction vessel was sealed and purged with argon. The reaction tube was then placed in a preheated oil bath and stirred at 60 °C for 18 h. The reaction mixture was allowed to cool to rt and MeOH (1 mL) was added. The resulting mixture was filtered through a pad of Celite®, eluting with further MeOH (2 × 3 mL). The solvent was removed under reduced pressure, and the crude material was purified by flash column chromatography (40% Et_2O/pentane) affording the *trans*-cyano derivative **S1** as a white solid (43.6 mg, 88%).

R_f 0.21 (40% Et2O/pentane).

mp = 125–127 °C (from Et_2O/pentane);

IR (film)/cm^{-1} 2974, 2244 (C≡N), 1689 (C=O), 1517, 1409, 1256, 1178, 1129, 1025, 880, 828, 768;

^{1}H NMR (400 MHz, $CDCl_3$, 298 K, observed as a mixture of rotamers) δ 7.25–7.19 (m, 2 H, HCAr), 6.95–6.89 (m, 2 H, HCAr), 4.04–3.84 (m, 2 H, NC*H*HCHAr, NCH*H*CHCN), 3.82 (s, 3 H, OCH_3), 3.64 (t, J = 9.9 Hz, 1 H, NC*H*HCHCN), 3.60–3.51 (m, 1 H, CHAr), 3.51–3.37 (m, 1 H, NCH*H*CHAr), 3.07 (q, J = 9.4 Hz, 1 H, CHCN), 1.50 and 1.48 (2 × s, 9 H, C(CH3)3);

^{13}C NMR (101 MHz, $CDCl_3$, 298 K, observed as a mixture of rotamers) δ 159.4 (OCAr quat), 153.8 (br, C=O carbamate), 128.4 (CAr quat), 128.2 (2 × CAr), 118.8 (C≡N), 114.5 (2 × CAr), 80.4 (br, $C(CH_3)_3$), 55.3 (OCH_3), 51.5 and 50.9 (N*C*H_2CHAr), 48.7 and 48.4 (N*C*H_2CHCN), 48.2 and 47.3 (CHAr), 36.2 and 35.3 (*C*HCN), 28.4 (C(*C*H_3)$_3$);

HRMS (FTMS + pAPCI) m/z Calculated for $C_{17}H_{22}N_2O_3Na^+$ $[M+Na]^+$ 325.1523; Found 325.1519.

cis-(±)-3-Carboxy-4-(4-methoxyphenyl)pyrrolidin-1-ium chloride (FRAG8)

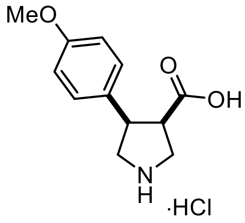

4 N HCl in 1,4-dioxane (1.1 mL, 4.50 mmol) was added to *N*-Boc protected pyrrolidine **27a** (145 mg, 0.45 mmol) at 0 °C. The resulting solution was warmed up to 20 ºC and the product began to crush out of solution. The reaction mixture was stirred at 20 °C for 1 h and then ice-cold Et_2O (2 mL) was added. The reaction mixture was filtered and the precipitate was washed with further ice-cold Et_2O (2 × 5 mL). The solid precipitate was dried under reduced pressure to afford the deprotected pyrrolidine product as the corresponding hydrochloride salt **FRAG8** as an off-white solid (97.4 mg, 84%).

mp = 214–218 °C (from dioxane/Et_2O);

IR (film)/cm^{-1} 2911 (NH, OH br), 1703 (C=O), 1517, 1387, 1252, 1200, 1170, 1029, 839, 779;

^1H NMR (400 MHz, CD_3OD, 298 K) δ 7.26–7.20 (m, 2 H, HC_{Ar}), 6.93–6.87 (m, 2 H, HC_{Ar}), 3.91 (ddd, *J* = 9.5, 9.5, 7.5 Hz, 1 H, CHAr), 3.77 (s, 3 H, OCH_3), 3.75–3.68 (m, 3 H, NC*H*HCHCO, NC*H₂*CHAr), 3.59 (dd, *J* = 12.2, 6.8 Hz, 1 H, NCH*H*CHCO), 3.49 (ddd, *J* = 7.6, 6.8, 3.4 Hz, 1 H, CHCO);

^{13}C NMR (101 MHz, CD_3OD, 298 K) δ 174.7 (COOH), 160.8 (OC_{Ar} quat), 130.0 (2 × C_{Ar}), 128.0 (C_{Ar} quat), 115.1 (2 × C_{Ar}), 55.7 (OCH_3), 49.4 (*C*HCO), 49.25 (N*C*H_2CHAr), 49.23 (N*C*H_2CHCO), 46.9 (CHAr);

HRMS (ESI^+) *m/z* Calculated for $C_{12}H_{16}NO_3$ [M–Cl] 222.1130; Found 222.1135.

trans-(±)-3-Carboxy-4-(4-methoxyphenyl)pyrrolidin-1-ium chloride (FRAG10)

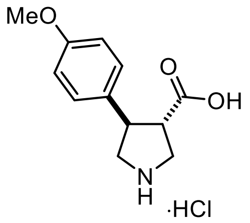

4 N HCl in 1,4-dioxane (1.0 mL, 4.00 mmol) was added to *N*-Boc protected pyrrolidine **24a** (130 mg, 0.40 mmol) at 0 °C. The resulting solution was warmed up to 20 ºC and the product began to crush out of solution. The reaction mixture was stirred at 20 °C for 1 h and then ice-cold Et_2O (2 mL) was added. The reaction mixture was filtered and the precipitate was washed with further ice-cold Et_2O (2 × 5 mL). The solid precipitate was dried under reduced pressure to afford the deprotected pyrrolidine product as the corresponding hydrochloride salt **FRAG10** as an off-white solid (93.7 mg, 91%).

mp = 209–213 °C (from dioxane/Et_2O);

IR (film)/cm^{-1} 2915 (NH, OH br), 1730 (C=O), 1513, 1383, 1256, 1163, 1029, 850, 678;

^1H NMR (400 MHz, CD_3OD, 298 K) δ 7.33–7.28 (m, 2 H, HC_{Ar}), 6.96–6.90 (m, 2 H, HC_{Ar}), 3.83–3.70 (m, 5 H, NC*H*HCHAr, NC*H*HCHCO, OCH_3), 3.70–3.59 (m, 2 H, NCH*H*CHCO, CHAr), 3.40–3.32 (m, 2 H, NCH*H*CHAr, CHCO);

^{13}C NMR (101 MHz, CD_3OD, 298 K) δ 173.9 (COOH), 160.9 (OC_{Ar} quat), 130.0 (C_{Ar} quat), 129.7 (2 × C_{Ar}), 115.4 (2 × C_{Ar}), 55.2 (OCH_3), 55.7 (N*C*H_2CHAr), 52.8 (*C*HCO), 50.7 (N*C*H_2CHCO), 48.5 (CHAr);

HRMS (ESI^+) *m/z* Calculated for $C_{12}H_{16}NO_3$ [M–Cl] 222.1130; Found 222.1126.

cis-(±)-3-Carboxy-4-(4-fluorophenyl)pyrrolidin-1-ium chloride (FRAG9)

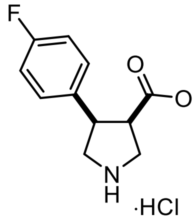

4 N HCl in 1,4-dioxane (1.0 mL, 4.00 mmol) was added to *N*-Boc protected pyrrolidine **27b** (124 mg, 0.40 mmol) at 0 °C. The resulting solution was warmed up to 20 °C and the product began to crush out of solution. The reaction mixture was stirred at 20 °C for 2 h and then ice-cold Et_2O (2 mL) was added. The reaction mixture was filtered and the precipitate was washed with further ice-cold Et_2O (2 × 5 mL). The solid precipitate was dried under reduced pressure to afford the deprotected pyrrolidine product as the corresponding hydrochloride salt **FRAG9** as a white solid (74.0 mg, 75%).

mp = 210–213 °C (from dioxane/Et_2O);

IR (film)/cm^{-1} 2807 (NH, OH br), 1730 (C=O), 1551, 1513, 1401, 1208, 835, 693;

^{1}H NMR (400 MHz, CD_3OD, 298 K) δ 7.37–7.30 (m, 2 H, HC_{Ar}), 7.13–7.05 (m, 2 H, HC_{Ar}), 3.96 (ddd, *J* = 9.5, 9.5, 7.3 Hz, 1 H, CHAr), 3.77–3.70 (m, 3 H, NC*H*HCHAr, NCH_2CHCO), 3.59 (dd, *J* = 12.0, 6.8 Hz, 1 H, NCH*H*CHAr), 3.52 (ddd, *J* = 7.1, 7.1, 3.0 Hz, 1 H, CHCO);

^{13}C NMR (101 MHz, CD_3OD, 298 K) δ 174.6 (COOH), 163.8 (d, J_{CF} = 245.4 Hz, FC_{Ar} quat), 132.3 (d, J_{CF} = 3.4 Hz, F-*p*-C_{Ar} quat), 130.9 (d, J_{CF} = 8.2 Hz, 2 × F-*m*-C_{Ar}), 116.4 (d, J_{CF} = 21.7 Hz, 2 × F-*o*-C_{Ar}), 49.4 (*C*HCO), 49.3 (N*C*H_2CHAr), 49.1 (N*C*H_2CHCO), 46.8 (CHAr);

^{19}F NMR (377 MHz, CD_3OD, 298 K) δ –116.6 (tt, J_{HF} = 8.7, 5.2 Hz);

HRMS (ESI$^+$) *m/z* Calculated for $C_{11}H_{13}NO_2F$ [M–Cl] 210.0930; Found 210.0936.

trans-(±)-3-Carboxy-4-(4-fluorophenyl)pyrrolidin-1-ium chloride (FRAG11)

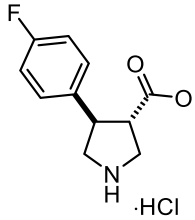

4 N HCl in 1,4-dioxane (1.0 mL, 4.00 mmol) was added to *N*-Boc protected pyrrolidine **24b** (124 mg, 0.40 mmol) at 0 °C. The reaction mixture was stirred at 20 °C for 2 h. Ice-cold Et_2O (3 mL) was then added and the product crushed out of solution. The reaction mixture was filtered, and the precipitate was washed with further ice-cold Et_2O (2 × 5 mL). The solid precipitate was found to be hygroscopic and turned quickly into a paste. This was solubilised in a 1:1 mixture of deionized H_2O and acetone. The resulting solution was concentrated under reduced pressure. Et_2O (3 mL) and pentane (3 mL) were added and the solvent was removed under reduced pressure to afford the carboxylic acid product as the corresponding hydrochloride salt **FRAG11** as a dark orange solid (95.6 mg, 97%).

mp = 71–74 °C (from Et_2O/pentane);

IR (film)/cm^{-1} 2896 (NH, OH br), 2747 (NH br), 1718 (C=O), 1513, 1398, 1223, 1163, 831;

^{1}H NMR (400 MHz, CD_3OD, 298 K) δ 7.45–7.38 (m, 2 H, HC_{Ar}), 7.16–7.08 (m, 2 H, HC_{Ar}), 3.83–3.62 (m, 4 H, CHAr, NC*H*HCHAr, NCH_2CHCO), 3.43–3.32 (m, 2 H, CHCO, NCH*H*CHAr);

^{13}C NMR (101 MHz, CD_3OD, 298 K) δ 173.6 (COOH), 163.8 (d, J_{CF} = 245.1 Hz, FC_{Ar} quat), 134.4 (d, J_{CF} = 2.9 Hz, F-*p*-C_{Ar} quat), 130.6 (d, J_{CF} = 8.2 Hz, 2 × F-*m*-C_{Ar}), 116.7 (d, J_{CF} = 21.8 Hz, 2 × F-*o*-C_{Ar}), 52.8 (N*C*H_2CHAr), 50.6 (*C*HCO), 48.9 (N*C*H_2CHCO), 48.2 (CHAr);

^{19}F NMR (377 MHz, CD_3OD, 298 K) δ –116.7 (tt, J_{HF} = 9.0, 5.4 Hz);

HRMS (ESI$^+$) *m/z* Calculated for $C_{11}H_{13}NO_2F$ [M–Cl] 210.0930; Found 210.0936.

Note: Hygroscopic solid.

Note: Avoid concentration from MeOH solution, as this could led to partial or complete esterification to the corresponding methyl ester, see compound S3.

trans-(±)-3-(4-Fluorophenyl)-4-(methoxycarbonyl)pyrrolidin-1-ium chloride (S3)

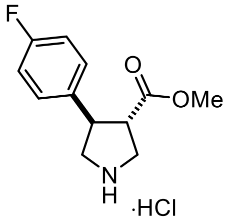

4 N HCl in 1,4-dioxane (1.0 mL, 4.00 mmol) was added to *N*-Boc protected pyrrolidine **24b** (124 mg, 0.40 mmol) at 0 °C. The reaction mixture was stirred at 20 °C for 18 h. Ice-cold Et_2O (3 mL) was then added and the product crushed out of solution. The reaction mixture was filtered and the precipitate was washed with further ice-cold Et_2O (2 × 5 mL). The solid precipitate was found to be hygroscopic and turned quickly into a paste. This was solubilised in MeOH and the resulting solution was concentrated under reduced pressure. The reaction was performed a second time on the crude mixture under the same conditions. Following an identical work-up procedure, Et_2O (3 mL) and pentane (3 mL) were added and the solvent was removed under reduced pressure to afford the methyl ester product as the corresponding hydrochloride salt **S3** as an off-white solid (86.3 mg, 83%).

mp = 152–155 °C (from Et_2O/pentane);

IR (film)/cm^{-1} 2848 (NH br), 2751 (NH br), 1718 (C=O), 1513, 1439, 1349, 1226, 1159, 1096, 816;

^{1}H NMR (400 MHz, CD_3OD, 298 K) δ 7.47–7.37 (m, 2 H, HC_{Ar}), 7.16–7.07 (m, 2 H, HC_{Ar}), 3.89–3.60 (m, 7 H, CHAr, NC*H*HCHAr, NCH_2CHCO, OCH_3), 3.46 (ddd, *J* = 9.0, 9.0, 9.0 Hz, 1 H, CHCO), 3.37 (dd, *J* = 10.1, 10.1 Hz, 1 H, NCH*H*CHAr);

^{13}C NMR (101 MHz, CD_3OD, 298 K) δ 172.6 (*C*OOCH_3), 163.8 (d, J_{CF} = 245.3 Hz, FC_{Ar} quat), 134.1 (d, J_{CF} = 2.7 Hz, F-*p*-C_{Ar} quat), 130.6 (d, J_{CF} = 8.4 Hz, 2 × F-*m*-C_{Ar}), 116.8 (d, J_{CF} = 21.7 Hz, 2 × F-*o*-C_{Ar}), 53.0 (OCH_3), 52.7 (N*C*H_2CHAr), 50.5 (*C*HCO), 48.8 (N*C*H_2CHCO), 48.3 (CHAr);

^{19}F NMR (377 MHz, CD_3OD, 298 K) δ –116.5 (tt, J_{HF} = 9.2, 5.1 Hz);

HRMS (ESI$^+$) *m/z* Calculated for $C_{12}H_{15}NO_2F$ [M–Cl] 224.1087; Found 224.1088.

trans-(±)-3-Carbamoyl-4-(4-methoxyphenyl)pyrrolidin-1-ium chloride (FRAG14)

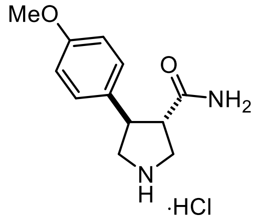

4 N HCl in 1,4-dioxane (1.0 mL, 4.00 mmol) was added to N-Boc protected pyrrolidine **32a** (128 mg, 0.40 mmol) at 0 °C. The resulting solution was warmed up to 20 ºC and the product began to crush out of solution. The reaction mixture was stirred at 20 °C for 3 h and then ice-cold Et_2O (2 mL) was added. The reaction mixture was filtered and the precipitate was washed with further ice-cold Et_2O (2 × 5 mL). The solid precipitate was dried under reduced pressure to afford the deprotected pyrrolidine product as the corresponding hydrochloride salt **FRAG14** as a white solid (94.4 mg, 92%).

mp = 275–278 °C (from dioxane/Et_2O);

IR (film)/cm^{-1} 3362 (CONH br), 3175 (CONH br), 2952 (NH br), 1689 (C=O), 1521, 1413, 1252, 1182, 1029, 828;

^{1}H NMR (400 MHz, DMSO-d_6, 298 K) δ 10.04–9.80 (br m, 1 H, N*H*H$^+$), 9.70–9.48 (br m, 1 H, NH*H*$^+$), 7.59 (s, 1 H, CON*H*H), 7.30–7.20 (m, 2 H, HC_{Ar}), 7.09 (s, 1 H, CONH*H*), 6.94–6.83 (m, 2 H, HC_{Ar}), 3.73 (s, 3 H, OCH_3), 3.65–3.56 (m, 3 H, NC*H*HCHAr, NC*H*HCHCO, CHAr), 3.26–3.02 (m, 3 H, NCH*H*CHAr, NCH*H*CHCO, CHCO);

^{13}C NMR (101 MHz, DMSO-d_6, 298 K) δ 171.3 ($CONH_2$), 158.5 (OC_{Ar} quat), 129.5 (C_{Ar} quat), 128.7 (2 × C_{Ar}), 114.0 (2 × C_{Ar}), 55.1 (OCH_3), 50.2 (N*C*H_2CHAr), 49.7 (*C*HCO), 47.7 (N*C*H_2CHCO), 46.4 (CHAr);

HRMS (ESI$^+$) *m/z* Calculated for $C_{12}H_{17}N_2O_2$ [M–Cl] 221.1290; Found 221.1288.

cis-(±)-3-Carbamoyl-4-(4-fluorophenyl)pyrrolidin-1-ium chloride (FRAG13)

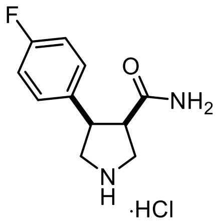

4 N HCl in 1,4-dioxane (1.0 mL, 4.00 mmol) was added to *N*-Boc protected pyrrolidine **30b** (123 mg, 0.40 mmol) at 0 °C. The resulting solution was warmed up to 20 ºC and the product began to crush out of solution. The reaction mixture was stirred at 20 °C for 2 h and then ice-cold Et_2O (2 mL) was added. The reaction mixture was filtered and the precipate was washed with further ice-cold Et_2O (2 × 5 mL). The solid precipitate was dried under reduced pressure to afford the deprotected pyrrolidine product as the corresponding hydrochloride salt **FRAG13** as an off-white solid (91.8 mg, 94%).

mp = 237–240 °C (from dioxane/Et_2O);

IR (film)/cm^{-1} 3373 (CONH br), 3194 (CONH br), 2937 (NH br), 1685 (C=O), 1513, 1394, 1223, 1159, 835;

^{1}H NMR (400 MHz, CD_3OD, 298 K) δ 7.39–7.32 (m, 2 H, HC_{Ar}), 7.12–7.02 (m, 2 H, HC_{Ar}), 3.92 (ddd, *J* = 11.8, 7.6, 7.6 Hz, 1 H, CHAr), 3.80 (dd, *J* = 11.4, 11.4 Hz, 1 H, NC*H*HCHCO), 3.72–3.64 (m, 2 H, NCH*H*CHCO, NC*H*HCHAr), 3.57 (dd, *J* = 12.0, 6.6 Hz, 1 H, NCH*H*CHAr), 3.43 (ddd, *J* = 6.9, 6.9, 2.3 Hz, 1 H, CHCO);

^{13}C NMR (101 MHz, CD_3OD, 298 K) δ 175.8 ($CONH_2$), 163.8 (d, J_{CF} = 245.3 Hz, FC_{Ar} quat), 132.1 (d, J_{CF} = 3.4 Hz, F-*p*-C_{Ar} quat), 131.1 (d, J_{CF} = 8.1 Hz, 2 × F-*m*-C_{Ar}), 116.4 (d, J_{CF} = 21.8 Hz, 2 × F-*o*-C_{Ar}), 49.7 (N*C*H_2CHAr), 49.3 (*C*HCO), 49.1 (N*C*H_2CHCO), 47.2 (CHAr);

^{19}F NMR (377 MHz, CD_3OD, 298 K) δ –116.7 (tt, J_{HF} = 9.0, 5.5 Hz);

HRMS (ESI^+) *m/z* Calculated for $C_{11}H_{14}N_2OF$ [M–Cl] 209.1090; Found 209.1087.

trans-(±)-3-Carbamoyl-4-(4-fluorophenyl)pyrrolidin-1-ium chloride (FRAG15)

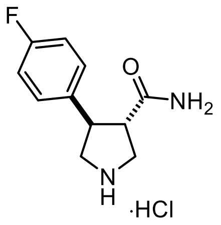

4 N HCl in 1,4-dioxane (0.8 mL, 3.20 mmol) was added to *N*-Boc protected pyrrolidine **32b** (100 mg, 0.32 mmol) at 0 °C. The resulting solution was warmed up to 20 ºC and the product began to crush out of solution. The reaction mixture was stirred at 20 °C for 3 h and then ice-cold Et_2O (2 mL) was added. The reaction mixture was filtered and the precipate was washed with further ice-cold Et_2O (2 × 5 mL). The solid precipitate was dried under reduced pressure to afford the deprotected pyrrolidine product as the corresponding hydrochloride salt **FRAG15** as a white solid (64.0 mg, 82%).

mp = 252–255 °C (from dioxane/Et_2O);

IR (film)/cm^{-1} 3362 (CONH br), 3183 (CONH br), 2907 (NH br), 2766 (NH br), 1681 (C=O), 1513, 1416, 1223, 831;

^{1}H NMR (400 MHz, CD_3OD, 298 K) δ 7.45–7.37 (m, 2 H, HC_{Ar}), 7.16–7.08 (m, 2 H, HC_{Ar}), 3.81–3.73 (m, 2 H, NC*H*HCHCO, NC*H*HCHAr), 3.69 (ddd, *J* = 10.2, 10.2, 7.9 Hz, 1 H, CHAr), 3.52 (dd, *J* = 11.8, 9.2 Hz, 1 H, NCH*H*CHCO), 3.39 (dd, *J* = 10.9, 10.9 Hz, 1 H, NCH*H*CHAr), 3.33–3.24 (m, 1 H, CHCO);

^{13}C NMR (101 MHz, CD_3OD, 298 K) δ 174.3 ($CONH_2$), 163.9 (d, J_{CF} = 245.5 Hz, FC_{Ar} quat), 133.8 (d, J_{CF} = 3.1 Hz, F-*p*-C_{Ar} quat), 130.7 (d, J_{CF} = 8.2 Hz, 2 × F-*m*-C_{Ar}), 116.8 (d, J_{CF} = 21.8 Hz, 2 × F-*o*-C_{Ar}), 52.3 (N*C*H_2CHAr), 51.6 (*C*HCO), 49.6 (N*C*H_2CHCO), 48.9 (CHAr);

^{19}F NMR (377 MHz, CD_3OD, 298 K) δ –116.5 (tt, J_{HF} = 8.5, 5.1 Hz);

HRMS (ESI^+) *m/z* Calculated for $C_{11}H_{14}N_2OF$ [M–Cl] 209.1090; Found 209.1092.

cis-(±)-3-(Hydroxymethyl)-4-(4-methoxyphenyl)pyrrolidin-1-ium chloride (FRAG16)

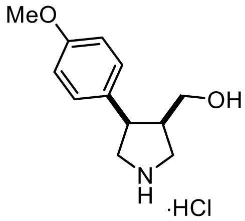

4 N HCl in 1,4-dioxane (1.2 mL, 4.90 mmol) was added to *N*-Boc protected pyrrolidine **28a** (150 mg, 0.49 mmol) at 0 °C. The reaction mixture was stirred at 20 °C for 18 h until the product completely crushed out of solution. Ice-cold Et_2O (3 mL) was then added. The reaction mixture was filtered, and the precipitate was washed with further ice-cold Et_2O (2 × 5 mL). The solid precipitate was dried under reduced pressure to afford the deprotected pyrrolidine product as the corresponding hydrochloride salt **FRAG16** as an off-white solid (113.1 mg, 95%).

mp = 138–142 °C (from dioxane/Et_2O);

IR (film)/cm^{-1} 3355 (OH br), 2967 (NH br), 2766, 1517, 1305, 1252, 1185, 1029, 828;

^{1}H NMR (400 MHz, CD_3OD, 298 K) δ 7.28–7.22 (m, 2 H, HC_{Ar}), 6.95–6.90 (m, 2 H, HC_{Ar}), 3.79 (s, 3 H, OCH_3), 3.76–3.57 (m, 3 H, NC*H*$_2$CHAr, CHAr), 3.56–3.50 (m, 2 H, NC*H*$_2$CHCH$_2$OH), 3.39 (dd, *J* = 10.6, 4.2 Hz, 1 H, C*H*HOH), 3.27 (dd, *J* = 10.6, 5.7 Hz, 1 H, CH*H*OH), 3.68–3.58 (m, 1 H, C*H*CH$_2$OH);

^{13}C NMR (101 MHz, CD_3OD, 298 K) δ 160.5 (OC$_{Ar}$ quat), 130.3 (2 × C$_{Ar}$), 128.9 (C$_{Ar}$ quat), 115.1 (2 × C$_{Ar}$), 61.3 (CH$_2$OH), 55.7 (OCH$_3$), 50.1 (N*C*H$_2$CHAr), 49.6 (N*C*H$_2$CHCH$_2$OH), 46.3 (CHAr), 45.0 (*C*HCH$_2$OH);

HRMS (ESI$^+$) *m/z* Calculated for $C_{12}H_{18}NO_2$ [M–Cl] 208.1338; Found 208.1342.

trans-(±)-3-(Hydroxymethyl)-4-(4-methoxyphenyl)pyrrolidin-1-ium chloride (FRAG18)

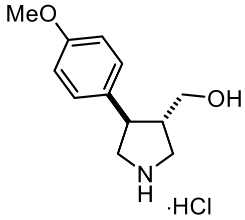

4 N HCl in 1,4-dioxane (1.0 mL, 4.00 mmol) was added to *N*-Boc protected pyrrolidine **29a** (123 mg, 0.40 mmol) at 0 °C. The resulting solution was warmed up to 20 ºC and the product began to crush out of solution. The reaction mixture was stirred at 20 °C for 3 h and then ice-cold Et_2O (2 mL) was added. The reaction mixture was filtered and the precipitate was washed with further ice-cold Et_2O (2 × 5 mL). The solid precipitate was dried under reduced pressure to afford the deprotected pyrrolidine product as the corresponding hydrochloride salt **FRAG18** as an off-white solid (83.3 mg, 85%).

mp = 193–195 °C (from dioxane/Et_2O);

IR (film)/cm^{-1} 3355 (OH br), 2952 (NH br), 1517, 1398, 1252, 1182, 1074, 1029, 813;

^{1}H NMR (400 MHz, CD_3OD, 298 K) δ 7.31–7.24 (m, 2 H, HC_{Ar}), 6.96–6.89 (m, 2 H, HC_{Ar}), 3.78 (s, 3 H, OCH_3), 3.70–3.58 (m, 3 H, NC*H*HCHAr, NC*H*HCHCH$_2$OH, C*H*HOH), 3.47 (dd, *J* = 11.2, 6.0 Hz, 1 H, CH*H*OH), 3.37–3.21 (m, 3 H, NCH*H*CHCH$_2$OH, NCH*H*CHAr, CHAr), 3.60–3.46 (m, 1 H, C*H*CH$_2$OH);

^{13}C NMR (101 MHz, CD_3OD, 298 K) δ 160.7 (OC_{Ar} quat), 130.6 (C_{Ar} quat), 129.8 (2 × C_{Ar}), 115.5 (2 × C_{Ar}), 60.7 (*C*H_2OH), 55.7 (OCH_3), 52.9 (N*C*H_2CHAr), 49.3 (N*C*H_2CHCH_2OH), 48.9 (*C*HCH_2OH), 45.8 (CHAr);

HRMS (ESI$^+$) *m/z* Calculated for $C_{12}H_{18}NO_2$ [M–Cl] 208.1338; Found 208.1335.

***cis*-(±)-3-(Hydroxymethyl)-4-(4-fluorophenyl)pyrrolidin-1-ium chloride (FRAG17)**

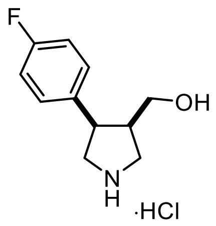

4 N HCl in 1,4-dioxane (0.9 mL, 3.70 mmol) was added to *N*-Boc protected pyrrolidine **28b** (110 mg, 0.37 mmol) at 0 °C. The reaction mixture was stirred at 20 °C for 18 h. Ice-cold Et_2O (3 mL) was then added and the product crushed out of solution. The reaction mixture was filtered and the precipitate was washed with further ice-cold Et_2O (2 × 5 mL). The solid precipitate was found to be hygroscopic and turned quickly into a paste. This was then solubilised in MeOH and the resulting solution was concentrated under reduced pressure to afford the deprotected pyrrolidine product as the corresponding hydrochloride salt **FRAG17** as a bright orange paste (81.9 mg, 96%).

IR (film)/cm^{-1} 3343 (OH br), 2904 (NH br), 2725 (NH br), 1603, 1513, 1402, 1223, 1163, 1045, 835;

^{1}H NMR (400 MHz, CD_3OD, 298 K) δ 7.41–7.34 (m, 2 H, HC_{Ar}), 7.15–7.06 (m, 2 H, HC_{Ar}), 3.84–3.59 (m, 3 H, NC*H*HCHAr, NC*H*HCHCH_2OH, CHAr), 3.59–3.52 (m, 2 H, NCH*H*CHCH_2OH, NCH*H*CHAr), 3.40 (dd, *J* = 10.6, 4.1 Hz, 1 H, C*H*HOH), 3.27 (dd, *J* = 10.7, 5.1 Hz, 1 H, CH*H*OH), 2.71–2.62 (m, 1 H, C*H*CH_2OH);

^{13}C NMR (101 MHz, CD_3OD, 298 K) δ 163.5 (d, J_{CF} = 244.7 Hz, FC_{Ar} quat), 133.2 (d, J_{CF} = 3.3 Hz, F-*p*-C_{Ar} quat), 131.2 (d, J_{CF} = 8.0 Hz, 2 × F-*m*-C_{Ar}), 116.4 (d, J_{CF} = 21.6 Hz, 2 × F-*o*-C_{Ar}), 61.2 (CH_2OH), 50.1 (N*C*H_2CHAr), 49.6 (N*C*H_2CHCH_2OH), 46.4 (CHAr), 44.9 (*C*HCH_2OH);

^{19}F NMR (377 MHz, CD_3OD, 298 K) δ –117.3 (tt, J_{HF} = 9.1, 5.5 Hz);

HRMS (ESI$^+$) *m/z* Calculated for $C_{11}H_{15}NOF$ [M–Cl] 196.1138; Found 196.1143.

***trans*-(±)-3-(Hydroxymethyl)-4-(4-fluorophenyl)pyrrolidin-1-ium chloride (FRAG19)**

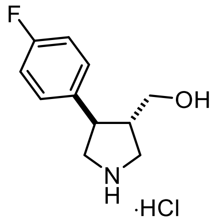

4 N HCl in 1,4-dioxane (1.2 mL, 4.00 mmol) was added to *N*-Boc protected pyrrolidine **29b** (118 mg, 0.40 mmol) at 0 °C. The reaction mixture was stirred at 20 °C for 18 h. Ice-cold Et_2O (3 mL) was then added and the product crushed out of solution. The reaction mixture was filtered and the precipitate was washed with further ice-cold Et_2O (2 × 5 mL). The solid precipitate was found to be hygroscopic and turned quickly into a paste. This was then solubilised in MeOH and the resulting solution was concentrated under reduced

pressure. Et_2O (3 mL) and pentane (3 mL) were added and the solvent was removed under reduced pressure to afford the deprotected pyrrolidine product as the corresponding hydrochloride salt **FRAG19** as a bright orange solid (87.1 mg, 94%).

mp = 120–123 °C (from Et_2O/pentane);

IR (film)/cm^{-1} 3347 (OH br), 2956 (NH br), 2740 (NH br), 1513, 1398, 1227, 1096, 826;

^{1}H NMR (400 MHz, CD_3OD, 298 K) δ 7.43–7.36 (m, 2 H, HC_{Ar}), 7.14–7.07 (m, 2 H, HC_{Ar}), 3.74–3.64 (m, 2 H, NC*H*HCHAr, NC*H*HCHCH$_2$OH), 3.62 (dd, *J* = 11.3, 3.5 Hz, 1 H, C*H*HOH), 3.47 (dd, *J* = 11.3, 5.8 Hz, 1 H, CH*H*OH), 3.40–3.28 (m, 3 H, NCH*H*CHCH$_2$OH, NCH*H*CHAr, CHAr), 2.62–2.51 (m, 1 H, C*H*CH$_2$OH);

^{13}C NMR (101 MHz, CD_3OD, 298 K) δ 163.7 (d, J_{CF} = 244.9 Hz, FC_{Ar} quat), 135.1 (d, J_{CF} = 3.1 Hz, F-*p*-C_{Ar} quat), 130.7 (d, J_{CF} = 8.1 Hz, 2 × F-*m*-C_{Ar}), 116.8 (d, J_{CF} = 21.7 Hz, 2 × F-*o*-C_{Ar}), 60.5 (CH_2OH), 52.9 (N*C*H$_2$CHAr), 49.2 (N*C*H$_2$CHCH$_2$OH), 49.0 (*C*HCH$_2$OH), 45.8 (CHAr);

^{19}F NMR (377 MHz, CD_3OD, 298 K) δ –117.0 (tt, J_{HF} = 9.0, 5.4 Hz);

HRMS (ESI$^+$) *m/z* Calculated for $C_{11}H_{15}NOF$ [M–Cl] 196.1138; Found 196.1145.

Note: Hygroscopic solid.

cis-(±)-(tert-Butoxycarbonyl)-4-(4-methoxyphenyl)piperidine-3-carboxylic acid (35a)

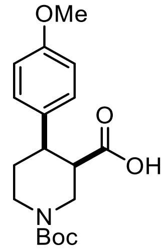

Prepared according to literature,[24a] a flame-dried round-bottom flask was charged with *cis*-arylated amide **9a** (346 mg, 0.75 mmol), followed by Boc_2O (982 mg, 4.50 mmol) and DMAP (18.3 mg, 0.15 mmol). The reaction vessel was sealed and purged with argon, then MeCN (1.25 mL, 0.6 M) was added by syringe. The reaction mixture was then placed in an oil bath and gradually warmed up to 70 °C. The reaction was then stirred at 70 °C for 48 h. The reaction mixture was allowed to cool to rt and sat. aq. NH_4Cl (3 mL) and CH_2Cl_2 (3 mL) were added. The phases were separated, and the aqueous layer was extracted with CH_2Cl_2 (3 × 5 mL). The combined organic extracts were dried over Na_2SO_4 and filtered. The solvent was removed under reduced pressure to afford the crude *N*-Boc protected piperidine derivative. A solution of H_2O_2 (30 wt.% in H_2O, 0.90 mL, 9.00 mmol) in THF (2.4 mL) was added to a solution of $LiOH·H_2O$ (252 mg, 6.00 mmol,) in H_2O (1.9 mL) at 0 °C under argon. The resulting mixture was added dropwise to a solution of crude *N*-Boc protected piperidine in THF (3.2 mL) at 0 °C under argon. The reaction was then stirred at 45 °C for 24 h. The reaction mixture was then allowed to cool down to rt and sat. aq. $Na_2S_2O_3$ (8 mL), EtOAc (5 mL) and 1 N NaOH (5 mL) were added. The aqueous alkaline layer was collected, and the organic layer extracted with 1 N NaOH (3 × 15 mL). All the aqueous alkaline layers were combined, washed with EtOAc (40 mL), and acidified to pH ~ 1 with HCl, 37%. The acidic layer was extracted with EtOAc (5 × 50 mL). The combined organic extracts were dried over Na_2SO_4 and filtered. Solvent was removed under reduced pressure. Et_2O (5 mL) and pentane (5 mL) were added and the solvent was removed under reduced pressure to afford the pure *cis*-acid **35a** as an off-white solid (223 mg, 89% over 2 steps).

R_f 0.17 (3% MeOH/CH_2Cl_2);

mp = 186–191 °C (from Et_2O/pentane);

ν_{max} (film)/cm^{-1} 2933 (OH br), 1706 (C=O), 1681 (C=O), 1514, 1465, 1418, 1366, 1244, 1178, 1118, 1040, 1006, 830, 769;

^{1}H NMR (400 MHz, $CDCl_3$, 298 K) δ 7.20–7.11 (m, 2 H, HC_{Ar}), 6.85–6.80 (m, 2 H, HC_{Ar}), 4.44 (br d, *J* = 13.6, 1 H, NC*H*HCHCO), 4.27 (br d, *J* = 12.5, 1 H, NC*H*HCH_2), 3.78 (s, 3 H, OCH_3), 3.18 (dd, *J* = 13.9, 3.8 Hz, 1 H, NCH*H*CHCO), 2.99–2.81 (m, 3 H, CHCO, CHAr, NCH*H*CH_2), 2.54 (dddd, *J* = 12.3, 12.3, 12.3, 4.5 Hz, 1 H, NCH_2C*H*H), 1.67 (dddd, *J* = 13.0, 3.3, 3.3, 3.3 Hz, 1 H, NCH_2CH*H*), 1.39 (s, 9 H, $C(CH_3)_3$);

^{13}C NMR (101 MHz, DMSO-*d*$_6$, 373 K) δ 176.6 (COOH), 158.3 (OC_{Ar} quat), 154.6 (C=O carbamate), 134.2 (C_{Ar} quat), 128.4 (2 × C_{Ar}), 113.8 (2 × C_{Ar}), 79.8 (*C*$(CH_3)_3$), 55.2 (OCH_3), 46.1 (N*C*H_2CHCO), 45.4 (*C*HCO), 43.9 (N*C*H_2CH_2), 42.3 (CHAr), 28.3 (C(*C*H_3)$_3$), 25.9 (NCH_2*C*H_2);

HRMS (ESI$^+$) *m/z* Calculated for $C_{18}H_{26}NO_5$ [M+H] 336.1811; Found 336.1819.

Characterisation information consistent with data previously reported in literature.[24a]

trans-(±)-1-(tert-Butoxycarbonyl)-4-(4-methoxyphenyl)piperidine-3-carboxylic acid (36a)

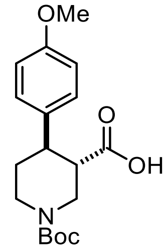

A flame-dried reaction tube was charged with *trans*-arylated amide **10a** (280 mg, 0.61 mmol), followed by Boc_2O (533 mg, 2.40 mmol) and DMAP (14.9 mg, 0.12 mmol). The reaction vessel was sealed and purged with argon, then MeCN (2.0 mL, 0.3 M) was added by syringe. The reaction tube was then placed in an oil bath and gradually warmed up to 40 °C. The reaction was then stirred at 40 °C for 20 h. The reaction mixture was allowed to cool to rt and sat. aq. NH_4Cl (3 mL) and CH_2Cl_2 (3 mL) were added. The phases were separated, and the aqueous layer was extracted with CH_2Cl_2 (3 × 5 mL). The combined organic extracts were dried over Na_2SO_4 and filtered. The solvent was removed under reduced pressure to afford the crude *N*-Boc protected piperidine derivative. A solution of H_2O_2 (30 wt.% in H_2O, 313 µL, 3.05 mmol) in THF (2.0 mL) was added to a solution of LiOH·H_2O (51.2 mg, 1.22 mmol) in H_2O (1.5 mL) at 0 °C under argon. The resulting mixture was added dropwise to a solution of crude *N*-Boc protected piperidine in THF (2.5 mL) at 0 °C under argon. The reaction was then stirred at 20 °C for 24 h. The reaction mixture was then allowed to cool down to rt and sat. aq. $Na_2S_2O_3$ (5 mL), EtOAc (5 mL) and 1 N NaOH (5 mL) were added. The aqueous alkaline layer was collected, and the organic layer extracted with 1 N NaOH (3 × 15 mL). All the aqueous alkaline layers were combined, washed with EtOAc (40 mL), and acidified to pH ~ 1 with HCl, 37%. The acidic layer was extracted with EtOAc (5 × 50 mL). The combined organic extracts were dried over Na_2SO_4 and filtered. Solvent was removed under reduced pressure. The crude material was purified by flash column chromatography (5% MeOH/CH_2Cl_2), the product containing fractions were combined and the solvent was removed under reduced pressure. Et_2O (5 mL) and pentane (5 mL) were added and the solvent was removed under reduced pressure to afford the pure *trans*-acid **36a** as a white solid (183 mg, 89% over 2 steps).

R_f 0.33 (5% MeOH/CH_2Cl_2).

mp = 57–62 °C;

IR (film)/cm^{-1} 2933 (OH br), 1692 (C=O), 1655 (C=O), 1513, 1428, 1286, 1238, 1156, 1126, 1033, 828, 764;

^{1}H NMR (400 MHz, $CDCl_3$, 298 K, observed as a mixture of rotamers) δ 7.09 (d, *J* = 8.3 Hz, 2 H, HC_{Ar}), 6.81 (d, *J* = 8.6 Hz, 2 H, HC_{Ar}), 4.37 (br s, 1 H, NC*H*HCHCO), 4.21 (br s, 1 H, NC*H*HCH$_2$), 3.78 (s, 3 H, OCH_3), 2.99–2.71 (m, 3 H, NCH*H*CHCO, NCH*H*CH$_2$, CHAr), 2.71–2.57 (m, 1 H, CHCO), 1.83–1.70 (m, 1 H, NCH$_2$C*H*H), 1.68–1.54 (m, 1 H, NCH$_2$CH*H*), 1.48 (s, 9 H, C(CH_3)$_3$);

^{13}C NMR (101 MHz, $CDCl_3$, 298 K, observed as a mixture of rotamers) δ 177.5 (br, COOH), 158.3 (OC_{Ar} quat), 154.5 (C=O carbamate), 134.6 (C_{Ar} quat), 128.2 (2 × C_{Ar}), 113.9 (2 × C_{Ar}), 80.2 (*C*(CH_3)$_3$), 55.1 (OCH_3), 48.8 (br, *C*HCO), 46.5 (br, N*C*H$_2$CHCO), 44.2 (CHAr), 43.7 (br, N*C*H$_2$CH$_2$), 32.8 (br, NCH$_2$*C*H$_2$), 28.4 (C(*C*H$_3$)$_3$);

HRMS (ESI$^+$) *m/z* Calculated for $C_{18}H_{26}NO_5$ [M+H] 336.1811; Found 336.1824.

trans-(±)-(_tert_-Butoxycarbonyl)-4-(4-fluorophenyl)piperidine-3-carboxylic acid (36b)

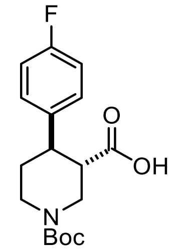

A flame-dried reaction tube was charged with *trans*-arylated amide **10b** (360 mg, 0.80 mmol), followed by Boc_2O (698 mg, 3.20 mmol) and DMAP (19.5 mg, 0.16 mmol). The reaction vessel was sealed and purged with argon, then anhydrous MeCN (2.7 mL, 0.3 M) was added by syringe. The reaction tube was placed in an oil bath and gradually warmed up to 40 °C. The reaction mixture was then stirred at 40 °C for 20 h. The reaction mixture was allowed to cool to rt and sat. aq. NH_4Cl (3 mL) and CH_2Cl_2 (3 mL) were added. The phases were separated, and the aqueous layer was extracted with CH_2Cl_2 (3 × 5 mL). The combined organic extracts were dried over Na_2SO_4 and filtered. The solvent was removed under reduced pressure to afford the crude *N*-Boc protected piperidine derivative. A solution of H_2O_2 (30 wt.% in H_2O, 410 μL, 4.00 mmol) in THF (2.5 mL) was added to a solution of LiOH·H_2O (67.1 mg, 1.60 mmol) in H_2O (2.0 mL) at 0 °C under argon. The resulting mixture was added dropwise to a solution of crude *N*-Boc protected piperidine in THF (3.5 mL) at 0 °C under argon. The reaction was then stirred at 20 °C for 24 h. Sat. aq. $Na_2S_2O_3$ (5 mL), EtOAc (5 mL) and 1 N NaOH (5 mL) were then added. The aqueous alkaline layer was collected, and the organic layer extracted with 1 N NaOH (3 × 15 mL). All the aqueous alkaline layers were combined, washed with EtOAc (40 mL), and acidified to pH ~ 1 with HCl, 37%. The acidic layer was extracted with EtOAc (5 × 50 mL). The combined organic extracts were dried over Na_2SO_4 and filtered. Solvent was removed under reduced pressure. Et_2O (5 mL) and pentane (5 mL) were added and the solvent was removed under reduced pressure to afford the pure *trans*-acid **36b** as a pale yellow solid (240 mg, 93% over 2 steps).

R_f 0.23 (20% acetone/pentane).

mp = 137–139 °C (from Et_2O/pentane);

IR (film)/cm^{-1} 2974 (OH br), 1692 (C=O), 1655 (C=O), 1510, 1424, 1282, 1226, 1156, 1126, 831, 775;

^{1}H NMR (400 MHz, $CDCl_3$, 298 K, observed as a mixture of rotamers) δ 7.17–7.09 (m, 2 H, HC_{Ar}), 7.00–6.92 (m, 2 H, HC_{Ar}), 4.39 (br s, 1 H, NC*H*HCHCO), 4.22 (br s, 1 H, NC*H*HCH_2), 2.97–2.71 (m, 3 H, NCH*H*CHCO, CHAr, NCH*H*CH_2), 2.71–2.59 (m, 1 H, CHCO), 1.78 (dddd, *J* = 13.4, 2.8, 2.8, 2.8 Hz, 1 H, NCH_2C*H*H), 1.60 (dddd, *J* = 13.6, 13.6, 13.6, 4.1 Hz, 1 H, NCH_2CH*H*), 1.48 (s, 9 H, $C(CH_3)_3$);

^{13}C NMR (101 MHz, $CDCl_3$, 298 K, observed as a mixture of rotamers) δ 177.1 (COOH), 161.7 (d, J_{CF} = 245.0 Hz, FC_{Ar} quat), 154.4 (C=O carbamate), 138.1 (d, J_{CF} = 2.6 Hz, F-*p*-C_{Ar} quat), 128.7 (d, J_{CF} = 7.8 Hz, 2 × F-*m*-C_{Ar}), 115.4 (d, J_{CF} = 21.2 Hz, 2 × F-*o*-C_{Ar}), 80.4 (*C*$(CH_3)_3$), 48.7 (br, *C*HCO), 46.4 (br, N*C*H_2CHCO), 44.4 (CHAr), 43.6 (br, N*C*H_2CH_2), 32.7 (br, NCH_2*C*H_2), 28.4 (C(*C*$H_3)_3$);

^{19}F NMR (377 MHz, $CDCl_3$, 298 K) δ –115.6 (br s);

HRMS (ESI$^+$) *m/z* Calculated for $C_{17}H_{23}NO_4F$ [M+H] 324.1611; Found 324.1625.

***trans*-(±)-*tert*-Butyl 3-carbamoyl-4-(4-fluorophenyl)piperidine-1-carboxylate (39b)**

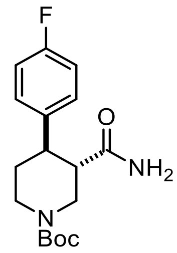

According to the procedure reported by Zhang and Chen,[28a] a round-bottom flask was charged with *trans*-arylated piperidine **10b** (360 mg, 0.80 mmol) and IBX (30 wt.%, 1.50 g, 1.60 mmol) under air. H_2O (2.7 mL) and HFIP (2.7 mL) were then added, and the resulting solution (0.15 M) was stirred at 60 °C for 1.5 h. The reaction mixture was then allowed to cool to rt and sat. aq. $NaHCO_3$ (10 mL) and CH_2Cl_2 (10 ml) were added. The phases were separated, and the aqueous layer was extracted with CH_2Cl_2 (3 × 15 mL). The combined organic extracts were dried over Na_2SO_4 and filtered. The solvent was removed under reduced pressure. The crude material was purified by flash column chromatography (30% acetone/pentane). The product containing fractions were combined and the solvent was removed under reduced pressure. Et_2O (5 mL) and pentane (5 mL) were added and the solvent was removed under reduced pressure to afford the primary amide **39b** as a white solid (211 mg, 65%).

R_f 0.30 (35% acetone/pentane);

mp = 180–183 °C (from Et_2O/pentane);

IR (film)/cm^{-1} 3422 (NH br), 3329 (NH br), 2978, 1685 (C=O), 1614 (C=O), 1510, 1428, 1364, 1219, 1159, 1129, 876, 828, 772;

^{1}H NMR (400 MHz, $CDCl_3$, 298 K, observed as a mixture of rotamers) δ 7.22–7.12 (m, 2 H, HC_{Ar}), 7.04–6.91 (m, 2 H, HC_{Ar}), 5.72–4.87 (br m, 2 H, NH_2), 4.57–4.95 (br m, 2 H, NC*H*HCHCO, NC*H*HCH_2), 3.16–2.71 (br m, 3 H, NCH*H*CHCO, CHAr, NCH*H*CH_2), 2.53–2.25 (br s, 1 H, CHCO), 1.86–1.75 (br m, 1 H, NCH_2C*H*H), 1.74–1.58 (br m, 1 H, NCH_2CH*H*), 1.49 (s, 9 H, $C(CH_3)_3$);

^{13}C NMR (101 MHz, $CDCl_3$, 298 K, observed as a mixture of rotamers) δ 174.0 (C=O amide), 161.7 (d, J_{CF} = 245.0 Hz, FC_{Ar} quat), 154.6 (C=O carbamate), 138.7 (d, J_{CF} = 3.3 Hz, F-*p*-C_{Ar} quat), 128.7 (d, J_{CF} = 7.8 Hz, 2 × F-*m*-C_{Ar}), 115.5 (d, J_{CF} = 21.1 Hz, 2 × F-*o*-C_{Ar}), 80.1 (*C*$(CH_3)_3$), 50.1 (br, *C*HCO), 46.5 (br, N*C*H_2CHCO), 44.4 (br, CHAr, N*C*H_2CH_2), 32.4 (br, NCH_2*C*H_2), 28.4 (C(*C*$H_3)_3$);

^{19}F NMR (377 MHz, $CDCl_3$, 298 K, observed as a mixture of rotamers) δ –115.9 (br s);

HRMS (ESI$^+$) *m/z* Calculated for $C_{17}H_{24}N_2O_3F$ [M+H] 323.1771; Found 323.1772.

cis-(±)-_tert_-Butyl 3-(hydroxymethyl)-4-(4-methoxyphenyl)piperidine-1-carboxylate (37a)

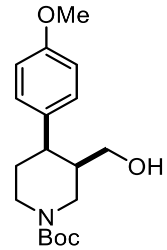

A flame-dried round-bottom flask was charged with *cis*-arylated amide **9a** (346 mg, 0.75 mmol), followed by Boc_2O (982 mg, 4.50 mmol) and DMAP (18.3 mg, 0.15 mmol). The reaction vessel was sealed and purged with argon, then MeCN (1.2 mL, 0.6 M) was added by syringe. The reaction mixture was placed in an oil bath and gradually warmed up to 70 °C. The reaction was then stirred at 70 °C for 48 h. The reaction mixture was allowed to cool to rt and sat. aq. NH_4Cl (3 mL) and CH_2Cl_2 (3 mL) were added. The phases were separated, and the aqueous layer was extracted with CH_2Cl_2 (3 × 5 mL). The combined organic extracts were dried over Na_2SO_4 and filtered. The solvent was removed under reduced pressure to afford the crude *N*-Boc protected piperidine derivative. A solution of the crude *N*-Boc protected derivative in dry THF (3.0 mL) was added dropwise to a suspension of $LiAlH_4$ (114 mg, 3.00 mmol) in dry THF (2.0 mL) at 0 °C under argon atmosphere. The mixture was then stirred at 20 °C for 30 min. The resulting suspension was filtered through a pad of Celite, eluting with EtOAc (3 × 5 mL). The phases were separated, and the aqueous layer was extracted with EtOAc (3 × 5 mL). The combined organic extracts were dried over Na_2SO_4 and filtered. The solvent was removed under reduced pressure. The crude material was purified by flash column chromatography (15% acetone/pentane). The product containing fractions were combined and the solvent was removed under reduced pressure. Et_2O (5 mL) and pentane (5 mL) were added and the solvent was removed under reduced pressure to afford the *cis*-alcohol **37a** as a white paste (160 mg, 66% over 2 steps).

R_f 0.24 (15% acetone/pentane);

IR (film)/cm^{-1} 3452 (OH br), 2930, 1659 (C=O), 1513, 1428, 1238, 1163, 1126, 1033, 999, 838,772;

^1H NMR (400 MHz, $CDCl_3$, 298 K, observed as a mixture of rotamers) δ 7.10–7.04 (m, 2 H, HC_{Ar}), 6.89–6.82 (m, 2 H, HC_{Ar}), 4.49–4.15 (br m, 2 H, NC*H*HCHCH$_2$OH, NC*H*HCH$_2$), 3.80 (s, 3 H, OCH_3), 3.59–3.28 (br m, 1 H, C*H*HOH), 3.15 (br s, 1 H, CH*H*OH), 3.03 (m, 1 H, CHAr), 2.92 (br s, 2 H, NCH*H*CHCH$_2$OH, NCH*H*CH$_2$), 2.13 (br s, 1 H, C*H*CH$_2$OH), 1.94 (dddd, *J* = 13.0, 13.0, 13.0, 4.7 Hz, 1 H, NCH$_2$CH*H*), 1.84 (br s, 0.4 H, OH), 1.71–1.60 (m, 1 H, NCH$_2$CH*H*), 1.50 and 1.48 (2 × s, 9 H, $C(CH_3)_3$);

^{13}C NMR (101 MHz, $CDCl_3$, 298 K, observed as a mixture of rotamers) δ 158.1 (OC_{Ar} quat), 134.6 (C_{Ar} quat), 127.9 (2 × C_{Ar}), 113.8 (2 × C_{Ar}), 80.1 (*C*(CH$_3$)$_3$), 57.8 (br, CH_2OH), 55.2 (OCH_3), 45.7 (br, N*C*H$_2$CHCH$_2$OH), 45.3 (br, N*C*H$_2$CH$_2$), 42.6 (CHAr), 42.1 (br, *C*HCH$_2$OH), 28.4 (C(*C*H$_3$)$_3$), 25.3 (NCH$_2$*C*H$_2$); the quaternary signal from the C=O (carbamate) was not observed;

HRMS (ESI$^+$) *m/z* Calculated for $C_{18}H_{28}NO_4$ [M+H] 322.2018; Found 322.2020.

trans-(±)-tert-Butyl 3-(hydroxymethyl)-4-(4-methoxyphenyl)piperidine-1-carboxylate (38a)

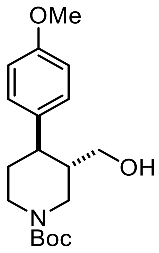

A flame-dried reaction tube was charged with *trans*-arylated amide **10a** (323 mg, 0.70 mmol), followed by Boc_2O (611 mg, 2.80 mmol) and DMAP (17.1 mg, 0.14 mmol). The reaction vessel was sealed and purged with argon, then MeCN (2.3 mL, 0.3 M) was added by syringe. The reaction tube was placed in an oil bath and gradually warmed up to 40 °C. The reaction mixture was then stirred at 40 °C for 24 h. The reaction mixture was allowed to cool to rt and sat. aq. NH_4Cl (3 mL) and CH_2Cl_2 (3 mL) were added. The phases were separated, and the aqueous layer was extracted with CH_2Cl_2 (3 × 5 mL). The combined organic extracts were dried over Na_2SO_4 and filtered. The solvent was removed under reduced pressure to afford the crude *N*-Boc protected piperidine derivative. A solution of the crude *N*-Boc protected derivative in dry THF (2.3 mL) was added dropwise to a suspension of $LiAlH_4$ (53.1 mg, 1.40 mmol) in dry THF (1.2 mL) at 0 °C under argon atmosphere. The mixture was then stirred at 20 °C for 30 min. The resulting suspension was filtered through a pad of Celite, eluting with EtOAc (3 × 5 mL). The phases were separated, and the aqueous layer was extracted with EtOAc (3 × 5 mL). The combined organic extracts were dried over Na_2SO_4 and filtered. The solvent was removed under reduced pressure. The crude material was purified by flash column chromatography (15% acetone/pentane). The product containing fractions were combined and the solvent was removed under reduced pressure. Et_2O (5 mL) and pentane (5 mL) were added and the solvent was removed under reduced pressure to afford the *trans*-alcohol **38a** as a colourless paste (188 mg, 84% over 2 steps).

R_f 0.19 (15% acetone/pentane);

IR (film)/cm^{-1} 3422 (OH br), 2930, 1662 (C=O), 1513, 1424, 1364, 1230, 1159, 1126, 1033, 826, 760;

^1H NMR (400 MHz, $CDCl_3$, 298 K, observed as a mixture of rotamers) δ 7.14–7.08 (m, 2 H, HC_{Ar}), 6.89–6.83 (m, 2 H, HC_{Ar}), 4.36 (br d, *J* = 13.4 Hz, 1 H, NC*H*HCHCH$_2$OH), 4.21 (br s, 1 H, NC*H*HCH$_2$), 3.80 (s, 3 H, OCH_3), 3.46 (dd, *J* = 11.0, 3.4 Hz, 1 H, C*H*HOH), 3.29 (dd, *J* = 11.0, 6.4 Hz, 1 H, CH*H*OH), 2.84–2.73

(m, 1 H, NCH*H*CH$_2$), 2.69 (dd, *J* = 13.3, 11.3 Hz, 1 H, NCH*H*CHCH$_2$OH), 2.48 (br ddd, *J* = 11.7, 11.7, 3.9 Hz, 1 H, CHAr), 1.88–1.73 (m, 2 H, C*H*CH$_2$OH, NCH$_2$C*H*H), 1.73–1.61 (m, 2 H, NCH$_2$CH*H*, OH), 1.50 (s, 9 H, C(CH$_3$)$_3$);

^{13}C NMR (101 MHz, $CDCl_3$, 298 K, observed as a mixture of rotamers) δ 158.2 (OC_{Ar} quat), 154.9 (C=O carbamate), 135.8 (C_{Ar} quat), 128.2 (2 × C_{Ar}), 114.0 (2 × C_{Ar}), 79.6 (*C*(CH$_3$)$_3$), 63.3 (CH$_2$OH), 55.2 (OCH_3), 47.2 (br, N*C*H$_2$CHCH$_2$OH), 44.6 (br, N*C*H$_2$CH$_2$), 44.1 (CHAr), 43.9 (*C*HCH$_2$OH), 34.1 (NCH$_2$*C*H$_2$), 28.4 (C(*C*H$_3$)$_3$);

HRMS (ESI$^+$) *m/z* Calculated for $C_{18}H_{28}NO_4$ [M+H] 322.2018; Found 322.2018.

trans-(±)-tert-Butyl 3-cyano-4-(4-fluorophenyl)piperidine-1-carboxylate (S2)

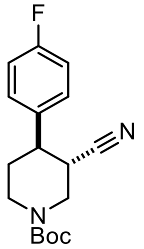

According to the procedure reported by Naka,[35] a flame-dried reaction tube was charged with *trans*-amide **39b** (50.0 mg, 0.16 mmol, 1 equiv), $Pd(TFA)_2$ (0.5 mg, 1.6 μmol, 1 mol%), dichloroacetonitrile (128 μL, 1.60 mmol, 10 equiv), dry MeCN (150 μL) and H_2O (150 μL). The reaction vessel was sealed and purged with argon. The reaction tube was then placed in a preheated oil bath and stirred at 60 ºC for 18 h. The reaction mixture was allowed to cool to rt and MeOH (1 mL) was added. The resulting mixture was filtered through a pad of Celite®, eluting with further MeOH (2 × 3 mL). The solvent was removed under reduced pressure, and the crude material was purified by flash column chromatography (30% Et_2O/pentane) affording the *trans*-cyano derivative **S2** as a white solid (19.8 mg, 41%).

R_f 0.32 (30% Et_2O/pentane).

mp = 136–139 °C (from Et_2O/pentane);

IR (film)/cm^{-1} 2986, 2240 (C≡N), 1681 (C=O), 1510, 1428, 1368, 1282, 1230, 1156, 835, 768;

^{1}H NMR (400 MHz, $CDCl_3$, 298 K, observed as a mixture of rotamers) δ 7.25–7.18 (m, 2 H, HCAr), 7.11–7.04 (m, 2 H, HCAr), 4.59 (br s, 1 H, NC*H*HCHCN), 4.28 (br s, 1 H, NC*H*HCH2), 2.98 (br s, 1 H, NC*H*HCHCN), 2.90–2.75 (m, 2 H, CHAr, NCH*H*CH2), 2.67 (td, J = 11.4, 4.0 Hz, 1 H, CHCN), 1.89 (dq, J = 13.8, 2.7 Hz, 1 H, NCH2C*H*H), 1.67 (qd, J = 13.4, 4.4 Hz, 1 H, NCH2CH*H*), 1.50 (s, 9 H, $C(CH_3)_3$);

^{13}C NMR (101 MHz, $CDCl_3$, 298 K, observed as a mixture of rotamers) δ 162.2 (d, J_{CF} = 246.6 Hz, FCAr quat), 153.9 (C=O carbamate), 136.6 (d, J_{CF} = 3.3 Hz, F-*p*-CAr quat), 128.7 (d, J_{CF} = 8.0 Hz, 2 ×F-m-CAr), 116.0 (d, J_{CF} = 21.4 Hz, 2 × F-*o*-CAr), 80.8 ($C(CH_3)_3$), 46.2 (br, NCH_2CHCN), 45.5 (CHAr), 43.6 (br, NCH_2CH_2), 35.1 (br, *C*HCN), 32.2 (br, NCH_2CH_2), 28.3 ($C(CH_3)_3$);

^{19}F NMR (377 MHz, $CDCl_3$, 298 K, observed as a mixture of rotamers) δ –114.4 (br m);

HRMS (ESI$^-$) m/z Calculated for $C_{17}H_{20}N_2O_2F$ [M–H] 303.1509; Found 303.1514.

cis-(±)-3-Carboxy-4-(4-methoxyphenyl)piperidin-1-ium chloride (FRAG20)

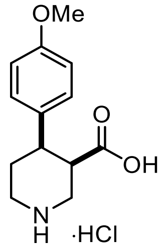

4 N HCl in 1,4-dioxane (1.1 mL, 4.50 mmol) was added to *N*-Boc protected piperidine **35a** (151 mg, 0.45 mmol) at 0 °C. The resulting solution was warmed up to 20 ºC and the product began to crush out of solution. The reaction mixture was stirred at 20 °C for 1 h and then ice-cold Et_2O (2 mL) was added. The reaction mixture was filtered and the precipate was washed with further ice-cold Et_2O (2 × 5 mL). The solid precipitate was dried under reduced pressure to afford the deprotected piperidine product as the corresponding hydrochloride salt **FRAG20** as a white solid (106 mg, 87%).

mp = 253–256 °C (from dioxane/Et_2O);

IR (film)/cm^{-1} 3064 (NH br), 2837 (OH br), 1685 (C=O), 1554, 1513, 1416, 1249, 1111, 1033, 876, 813, 705;

^{1}H NMR (400 MHz, CD_3OD, 298 K) δ 7.22–7.15 (m, 2 H, HC_{Ar}), 6.91–6.84 (m, 2 H, HC_{Ar}), 3.77 (s, 3 H, OCH_3), 3.64 (ddd, *J* = 13.0, 1.8, 1.8 Hz, 1 H, NC*H*HCHCO), 3.58–3.51 (ddd, *J* = 12.6, 4.1, 4.1 Hz, 1 H, NC*H*HCH_2), 3.35–3.26 (m, 2 H, CHAr, NCH*H*CHCO), 3.17 (ddd, *J* = 12.7, 12.7, 3.3 Hz, 1 H, NCH*H*CH_2), 3.13–3.09 (m, 1 H, CHCO), 2.54 (dddd, *J* = 14.2, 12.8, 12.8, 4.2 Hz, 1 H, NCH_2C*H*H), 1.98 (dddd, *J* = 14.4, 2.9, 2.9, 2.9 Hz, 1 H, NCH_2CH*H*);

^{13}C NMR (101 MHz, CD_3OD, 298 K) δ 176.1 (COOH), 160.3 (OC_{Ar} quat), 134.1 (C_{Ar} quat), 129.3 (2 × C_{Ar}), 114.9 (2 × C_{Ar}), 55.6 (OCH_3), 46.5 (N*C*H_2CHCO), 45.3 (N*C*H_2CH_2), 44.1 (*C*HCO), 41.0 (CHAr), 24.7 (NCH_2*C*H_2);

HRMS (ESI$^+$) *m/z* Calculated for $C_{13}H_{18}NO_3$ [M–Cl] 236.1287; Found 236.1284.

trans-(±)-3-Carboxy-4-(4-methoxyphenyl)piperidin-1-ium chloride (FRAG22)

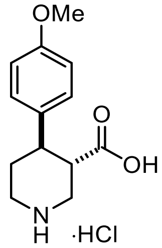

4 N HCl in 1,4-dioxane (1.0 mL, 4.00 mmol) was added to *N*-Boc protected piperidine **36a** (134 mg, 0.40 mmol) at 0 °C. The resulting solution was warmed up to 20 ºC and the product began to crush out of solution. The reaction mixture was stirred at 20 °C for 1 h and then ice-cold Et_2O (2 mL) was added. The reaction mixture was filtered and the precipate was washed with further ice-cold Et_2O (2 × 5 mL). The solid precipitate was dried under reduced pressure to afford the deprotected piperidine product as the corresponding hydrochloride salt **FRAG22** as a white solid (87.3 mg, 80%).

mp = 269–272 °C (from dioxane/Et_2O);

IR (film)/cm^{-1} 3101 (NH br), 2792 (OH br), 1722 (C=O), 1513, 1409, 1230, 1182, 1033, 828, 678;

^{1}H NMR (400 MHz, CD_3OD, 298 K) δ 7.22–7.17 (m, 2 H, HC_{Ar}), 6.90–6.84 (m, 2 H, HC_{Ar}), 3.76 (s, 3 H, OCH_3), 3.68–3.61 (m, 1 H, NC*H*HCHCO), 3.50 (dddd, *J* = 12.8, 3.3, 3.3, 1.6 Hz, 1 H, NC*H*HCH_2), 3.23–3.13 (m, 2 H, NCH*H*CHCO, NCH*H*CH_2), 3.08–2.97 (m, 2 H, CHAr, CHCO), 2.08–1.91 (m, 2 H, NCH_2CH_2);

^{13}C NMR (101 MHz, CD_3OD, 298 K) δ 173.7 (COOH), 160.4 (OC_{Ar} quat), 134.8 (C_{Ar} quat), 129.4 (2 × C_{Ar}), 115.0 (2 × C_{Ar}), 55.7 (OCH_3), 47.6 (*C*HCO), 46.8 (N*C*H_2CHCO), 45.4 (N*C*H_2CH_2), 43.8 (CHAr), 31.1 (NCH_2*C*H_2);

HRMS (ESI$^+$) *m/z* Calculated for $C_{13}H_{18}NO_3$ [M–Cl] 236.1287; Found 236.1296.

trans-(±)-3-Carboxy-4-(4-fluorophenyl)piperidin-1-ium chloride (FRAG23)

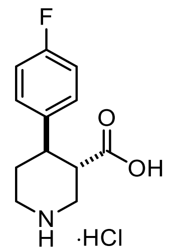

4 N HCl in 1,4-dioxane (1.0 mL, 4.00 mmol) was added to *N*-Boc protected piperidine **36b** (134 mg, 0.40 mmol) at 0 °C. The resulting solution was warmed up to 20 ºC and the product began to crush out of solution. The reaction mixture was stirred at 20 °C for 1 h and then ice-cold Et_2O (2 mL) was added. The reaction mixture was filtered and the precipate was washed with further ice-cold Et_2O (2 × 5 mL). The solid precipitate was dried under reduced pressure to afford the deprotected piperidine product as the corresponding hydrochloride salt **FRAG23** as a white solid (81.3 mg, 78%).

mp = 269–271 °C (from dioxane/Et_2O);

IR (film)/cm^{-1} 2926 (OH br), 2281, 1733 (C=O), 1510, 1394, 1215, 1185, 1111, 794;

^{1}H NMR (400 MHz, CD_3OD, 298 K) δ 7.34–7.27 (m, 2 H, HC_{Ar}), 7.09–7.01 (m, 2 H, HC_{Ar}), 3.66 (ddd, *J* = 12.2, 3.2, 1.4 Hz, 1 H, NC*H*HCHCO), 3.51 (dddd, *J* = 12.8, 3.3, 3.3, 1.5 Hz, 1 H, NC*H*HCH_2), 3.25–3.15 (m, 2 H, NCH*H*CHCO, NCH*H*CH_2), 3.15–3.03 (m, 2 H, CHAr, CHCO), 2.09–1.95 (m, 2 H, NCH_2CH_2);

^{13}C NMR (101 MHz, CD_3OD, 298 K) δ 173.5 (COOH), 163.4 (d, J_{CF} = 244.0 Hz, FC_{Ar} quat), 139.0 (d, J_{CF} = 3.1 Hz, F-*p*-C_{Ar} quat), 130.3 (d, J_{CF} = 7.9 Hz, 2 × F-*m*-C_{Ar}), 116.3 (d, J_{CF} = 21.5 Hz, 2 × F-*o*-C_{Ar}), 47.3 (*C*HCO), 46.7 (N*C*H_2CHCO), 45.3 (N*C*H_2CH_2), 43.8 (CHAr), 31.0 (NCH_2*C*H_2);

^{19}F NMR (377 MHz, CD_3OD, 298 K) δ –117.6 (tt, J_{HF} = 8.8, 5.3 Hz);

HRMS (ESI$^+$) *m/z* Calculated for $C_{12}H_{15}NO_2F$ [M–Cl] 224.1087; Found 224.1082.

trans-(±)-3-Carbamoyl-4-(4-fluorophenyl)piperidin-1-ium chloride (FRAG27)

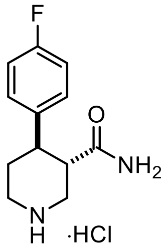

4 N HCl in 1,4-dioxane (1.0 mL, 4.00 mmol) was added to *N*-Boc protected piperidine **39b** (130 mg, 0.40 mmol) at 0 °C. The resulting solution was warmed up to 20 ºC and the product began to crush out of solution. The reaction mixture was stirred at 20 °C for 1 h and then ice-cold Et_2O (2 mL) was added. The reaction mixture was filtered and the precipitate was washed with further ice-cold Et_2O (2 × 5 mL). The solid precipitate was dried under reduced pressure to afford the deprotected piperidine product as the corresponding hydrochloride salt **FRAG27** as an off-white solid (93.8 mg, 91%).

mp > 300 °C (from dioxane/Et_2O);

ν_{max} (film)/cm^{-1} 3247 (CONH br), 3124 (CONH br), 3019 (NH br), 1670 (C=O), 1510, 1431, 1219, 828;

^{1}H NMR (400 MHz, DMSO-d_6, 298 K) δ 9.64–9.12 (br m, 1 H, NH_2^+), 7.46 (s, 1 H, CON*H*H), 7.29–7.18 (m, 2 H, HC_{Ar}), 7.18–7.06 (m, 2 H, HC_{Ar}), 6.82 (s, 1 H, CONH*H*), 3.46–3.33 (m, 1 H, NC*H*HCHCO), 3.29 (br d, *J* = 12.9 Hz, 1 H, NC*H*HCH$_2$), 3.08–2.86 (m, 4 H, NCH*H*CHCO, NCH*H*CH$_2$, CHAr, CHCO), 2.02–1.87 (br m, 1 H, NCH$_2$C*H*H), 1.87–1.76 (br m, 1 H, NCH$_2$CH*H*);

^{13}C NMR (101 MHz, DMSO-d_6, 298 K) δ 171.5 (CONH$_2$), 161.0 (d, J_{CF} = 242.2 Hz, FC$_{Ar}$ quat), 138.7 (F-*p*-C$_{Ar}$ quat, observed as singlet), 129.2 (d, J_{CF} = 8.0 Hz, 2 × F-*m*-C$_{Ar}$), 115.0 (d, J_{CF} = 21.0 Hz, 2 × F-*o*-C$_{Ar}$), 45.3 (*C*HCO), 45.1 (br, N*C*H$_2$CHCO), 43.1 (N*C*H$_2$CH$_2$), 41.6 (CHAr), 29.4 (NCH$_2$*C*H$_2$);

^{19}F NMR (377 MHz, DMSO-d_6, 298 K) δ –116.3 (br s);

HRMS (ESI$^+$) *m/z* Calculated for $C_{12}H_{16}N_2OF$ [M–Cl] 223.1247; Found 223.1240.

cis-(±)-3-(Hydroxymethyl)-4-(4-methoxyphenyl)piperidin-1-ium chloride (FRAG28)

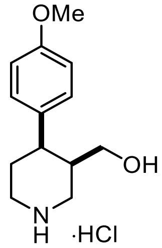

4 N HCl in 1,4-dioxane (1.0 mL, 4.00 mmol) was added to *N*-Boc protected piperidine **37a** (131 mg, 0.41 mmol) at 0 °C. The resulting solution was warmed up to 20 ºC and the product began to crush out of solution. The reaction mixture was stirred at 20 °C for 2 h and then ice-cold Et_2O (2 mL) was added. The reaction mixture was filtered and the precipate was washed with further ice-cold Et_2O (2 × 5 mL). The solid precipitate was dried under reduced pressure to afford the deprotected piperidine product as the corresponding hydrochloride salt **FRAG28** as a bright orange solid (74.8 mg, 71%).

mp = 174–177 °C (from dioxane/Et_2O);

ν_{max} (film)/cm^{-1} 3377 (OH br), 2945 (NH br), 2803 (NH br), 1595, 1513, 1457, 1271, 1245, 1178, 1021, 980, 828;

^{1}H NMR (400 MHz, CD_3OD, 298 K) δ 7.24–7.17 (m, 2 H, HC_{Ar}), 6.94–6.88 (m, 2 H, HC_{Ar}), 3.78 (s, 3 H, OCH_3), 3.68 (ddd, *J* = 13.1, 1.9, 1.9 Hz, 1 H, NC*H*HCHCH$_2$OH), 3.59 (dd, *J* = 10.8, 5.8 Hz, 1 H, C*H*HOH), 3.56–3.50 (m, 1 H, NC*H*HCH$_2$), 3.47 (dd, *J* = 10.8, 4.2 Hz, 1 H, CH*H*OH), 3.37–3.30 (m, 1 H, NCH*H*CHCH$_2$OH), 3.26–3.11 (m, 2 H, NCH*H*CH$_2$, CHAr), 2.49 (dddd, *J* = 13.2, 13.2, 13.2, 4.3 Hz, 1 H NCH$_2$C*H*H), 2.20–2.12 (m, 1 H, C*H*CH$_2$OH), 1.93 (dddd, *J* = 14.1, 3.4, 3.4, 3.4 Hz, 1 H, NCH$_2$CH*H*);

^{13}C NMR (101 MHz, CD_3OD, 298 K) δ 160.0 (OC_{Ar} quat), 135.0 (C_{Ar} quat), 129.4 (2 × C_{Ar}), 115.0 (2 × C_{Ar}), 60.9 (CH_2OH), 55.7 (OCH_3), 48.0 (N*C*H$_2$CHCH$_2$OH), 45.7 (N*C*H$_2$CH$_2$), 41.8 (CHAr), 39.7 (*C*HCH$_2$OH), 25.1 (NCH$_2$*C*H$_2$);

HRMS (ESI$^+$) *m/z* Calculated for $C_{13}H_{20}NO_2$ [M–Cl] 222.1494; Found 222.1496.

trans-(±)-3-(Hydroxymethyl)-4-(4-methoxyphenyl)piperidin-1-ium chloride (FRAG30)

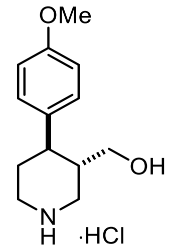

4 N HCl in 1,4-dioxane (1.0 mL, 4.00 mmol) was added to *N*-Boc protected piperidine **38a** (130 mg, 0.40 mmol) at 0 °C. The reaction mixture was stirred at 20 °C for 18 h. Ice-cold Et_2O (3 mL) was then added and the product crushed out of solution. The reaction mixture was filtered and the precipate was washed with further ice-cold Et_2O (2 × 5 mL). The solid precipitate was found to be hygroscopic and turned quickly into a paste. This was then solubilised in MeOH and the resulting solution was concentrated under reduced pressure. Et_2O (3 mL) and pentane (3 mL) were added and the solvent was removed under reduced pressure to afford the deprotected piperidine product as the corresponding hydrochloride salt **FRAG30** as a bright orange solid (99.8 mg, 97%).

mp = 56–60 °C (from Et_2O/pentane);

IR (film)/cm^{-1} 3343 (OH br), 2930 (NH br), 2796 (NH br), 1610, 1513, 1461, 1241, 1178, 1029, 828;

^{1}H NMR (400 MHz, CD_3OD, 298 K) δ 7.21–7.14 (m, 2 H, HC_{Ar}), 6.92–6.86 (m, 2 H, HC_{Ar}), 3.77 (s, 3 H, OCH_3), 3.61 (ddd, *J* = 12.7, 4.1, 1.5 Hz, 1 H, NC*H*HCHCH$_2$OH), 3.47 (dddd, *J* = 12.0, 3.1, 3.1, 1.4 Hz, 1 H, NC*H*HCH$_2$), 3.36 (dd, *J* = 11.1, 3.2 Hz, 1 H, C*H*HOH), 3.20 (dd, *J* = 11.1, 7.1 Hz, 1 H, CH*H*OH), 3.08 (ddd, *J* = 12.9, 9.8, 7.8 Hz, 1 H, NCH*H*CH$_2$), 2.96 (dd, *J* = 12.4, 12.4 Hz, 1 H, NCH*H*CHCH$_2$OH), 2.67 (ddd, *J* = 11.4, 9.3, 6.8 Hz, 1 H, CHAr), 2.20–2.08 (m, 1 H, C*H*CH$_2$OH), 2.01–1.93 (m, 2 H, NCH$_2$C*H$_2$*);

^{13}C NMR (101 MHz, CD_3OD, 298 K) δ 160.2 (OC_{Ar} quat), 135.5 (C_{Ar} quat), 129.4 (2 × C_{Ar}), 115.2 (2 × C_{Ar}), 62.2 (CH_2OH), 55.7 (OCH_3), 48.2 (N*C*H$_2$CHCH$_2$OH), 45.8 (N*C*H$_2$CH$_2$), 42.8 (CHAr), 42.7 (*C*HCH$_2$OH), 32.0 (NCH$_2$*C*H$_2$);

HRMS (ESI$^+$) *m/z* Calculated for $C_{13}H_{20}NO_2$ [M–Cl] 222.1494; Found 222.1503.

<u>Note:</u> Hygroscopic solid.

tert-Butyl cis-(±)-6,16-dioxo-3,3a,7,7a,16,16a-hexahydro-6H-pyrido[2,3-a]pyrrolo[3',4':3,4]pyrrolo[2,1-l]phenoxazine-2(1H)-carboxylate (31)

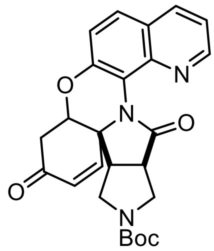

A reaction tube was charged with *cis*-arylated pyrrolidine **6a** (89.4 mg, 0.20 mmol) and IBX (45 wt. %, 249 mg, 0.40 mmol) under air. H_2O (700 μL) and HFIP (700 μL) were then added, the vial was sealed, and the solution was stirred at 60 °C for 1.5 h. The reaction mixture was then allowed to cool to rt and sat. aq. $NaHCO_3$ (3 mL) was added. The aqueous layer was extracted with CH_2Cl_2 (3 × 5 mL). The combined organic extracts were dried over Na_2SO_4 and filtered. The solvent was removed under reduced pressure. The crude material was purified by flash column chromatography (40% acetone/pentane). The product containing fractions were combined and the solvent was removed under reduced pressure. Et_2O (2 mL) and pentane (2 mL) were added, and the solvent was removed under reduced pressure to afford product **31** as a white solid (15.7 mg, 16%).

R_f 0.36 (40% acetone/pentane);

mp = 154–158 °C (from Et_2O/pentane);

IR (film)/cm^{-1} 3358, 2974, 1685 (C=O), 1618, 1394, 1226, 1163, 1122, 1029, 831, 790;

^{1}H NMR (400 MHz, DMSO-d_6, 373 K) δ 8.84 (dd, *J* = 4.2, 1.6 Hz, 1 H, $HC_{Ar}N$), 8.22 (dd, *J* = 8.2, 1.6 Hz, 1 H, HC_{Ar}), 7.75 (d, *J* = 9.0 Hz, 1 H, HC_{Ar}), 7.38 (dd, *J* = 8.2, 4.2 Hz, 1 H, HC_{Ar}), 7.19 (d, *J* = 8.9 Hz, 1 H, HC_{Ar}), 6.57 (dd, *J* = 10.4, 2.7 Hz, 1 H, C*H*=CHCO), 5.99 (dd, *J* = 10.4, 1.2 Hz, 1 H, CH=C*H*CO), 4.97–4.93 (m, 1 H, OCH), 3.84 (dd, *J* = 11.4, 2.9 Hz, 1 H, NC*H*HCHCO$_{amide}$), 3.76–3.66 (m, 2 H, NCH*H*CHCO$_{amide}$, NC*H*HCH), 3.61 (dd, *J* = 11.8, 8.1 Hz, 1 H, NCH*H*CH), 3.50 (ddd, *J* = 8.3, 8.3, 5.8 Hz, 1 H, NCH_2CH), 3.45–3.34 (m, 2 H, CHCO$_{amide}$, C*H*HCO$_{ketone}$), 2.93–2.82 (m, 1 H, CH*H*CO$_{ketone}$), 1.48 (s, 9 H, $C(CH_3)_3$);

^{13}C NMR (101 MHz, DMSO-d_6, 373 K) δ 192.9 (C=O ketone), 171.6 (C=O amide), 152.7 (C=O carbamate), 149.3 (NC_{Ar}), 147.9 (OC_{Ar} quat), 145.0 (*C*H=CHCO), 143.0 (C_{Ar} quat), 134.9 (C_{Ar}), 131.8 (CH=*C*HCO), 126.4 (C_{Ar}), 122.9 (C_{Ar} quat), 118.9 (C_{Ar}), 117.0 (C_{Ar}), 116.1 (C_{Ar} quat), 78.6 ($C(CH_3)_3$), 77.9 (OCH), 58.0 ($NCCH_2$=CH_2), 47.7 (NCH_2CHCO_{amide}), 46.2 (NCH_2CH), 44.5 ($CHCO_{amide}$), 41.2 (NCH_2CH), 39.5 (CH_2CO_{ketone}), 27.6 ($C(CH_3)_3$);

HRMS (ESI$^+$) *m/z* Calculated for $C_{25}H_{26}N_3O_5$ [M+H] 448.1867; Found 448.1878.

tert-Butyl _cis_-(±)-3',4-dioxo-2',3',3a',6',7',7a'-hexahydrospiro[cyclohexane-1,1'-pyrrolo[3,4-c]pyridine]-2,5-diene-5'(4'H)-carboxylate (40)

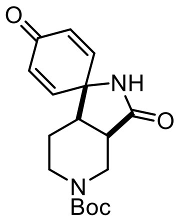

A reaction tube was charged with *cis*-arylated piperidine **9a** (46.2 mg, 0.10 mmol) and IBX (30 wt.%, 187 mg, 0.20 mmol) under air. H_2O (350 μL) and HFIP (250 μL) were then added, the vial was sealed, and the solution (0.15 M) was stirred at 60 °C for 1.5 h. The reaction mixture was then allowed to cool to rt and sat. aq. $NaHCO_3$ (3 mL) was added. The aqueous layer was extracted with CH_2Cl_2 (3 × 5 mL). The combined organic extracts were dried over Na_2SO_4 and filtered. The solvent was removed under reduced pressure. The crude material was purified by flash column chromatography (40% acetone/pentane). The product containing fractions were combined and the solvent was removed under reduced pressure. Et_2O (2 mL) and pentane (2 mL) were added and the solvent was removed under reduced pressure to afford the product **40** as a pale yellow paste (12.5 mg, 39%).

R_f 0.26 (40% acetone/pentane);

IR (film)/cm^{-1} 3250 (NH br), 2974, 1666 (C=O), 1625 (C=O), 1405, 1245, 1159, 1118, 865, 746;

^{1}H NMR (400 MHz, $CDCl_3$, 298 K, observed as a mixture of rotamers) δ 6.92 (dd, *J* = 10.1, 3.0 Hz, 1 H, (C*H*=CH)CO(CH=CH)), 6.87 (dd, *J* = 10.3, 3.1 Hz, 1 H, (CH=CH)CO(CH=C*H*)), 6.37 (dd, *J* = 10.3, 1.9 Hz, 1 H, (CH=CH)CO(C*H*=CH)), 6.30 (br s, 1 H, NH), 6.25 (dd, *J* = 10.0, 2.0 Hz, 1 H, (CH=C*H*)CO(CH=CH)), 4.16 (br s, 1 H, NC*H*HCHCO), 3.85 (br s, 1 H, NC*H*HCH$_2$), 3.49–3.28 (br m, 1 H, NCH*H*CHCO), 2.97–2.73 (br m, 2 H, NCH*H*CH$_2$, CHCO), 2.66–2.55 (m, 1 H, NCH$_2$CH$_2$C*H*), 1.81–1.66 (br m, 2 H, NCH$_2$C*H*$_2$), 1.46 (s, 9 H, C(CH$_3$)$_3$);

^{13}C NMR (101 MHz, $CDCl_3$, 298 K, observed as a mixture of rotamers) δ 184.0 (C=O ketone), 175.6 (C=O amide), 154.6 (C=O carbamate), 148.8 ((*C*H=CH)CO(CH=CH)), 146.7 ((CH=CH)CO(CH=*C*H)), 131.5 ((CH=CH)CO(*C*H=CH)), 128.6 ((CH=*C*H)CO(CH=CH)), 80.2 (*C*(CH$_3$)$_3$), 58.5 (CNH quat), 44.0 (NCH$_2$CH$_2$*C*H), 41.4 (br, N*C*H$_2$CH$_2$), 40.5 (br, N*C*H$_2$CHCO), 40.3 (*C*HCO), 28.3 (C(*C*H$_3$)$_3$), 24.5 (NCH$_2$*C*H$_2$);

HRMS (ESI$^-$) *m/z* Calculated for $C_{17}H_{21}N_2O_4$ [M–H] 317.1501; Found 317.1504.

tert-Butyl _trans_-(±)-3',4-dioxo-2',3',3a',6',7',7a'-hexahydrospiro[cyclohexane-1,1'-pyrrolo[3,4-c]pyridine]-2,5-diene-5'(4'H)-carboxylate (41)

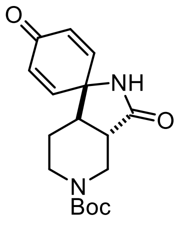

A round bottom flask was charged with *trans*-arylated piperidine **10a** (360 mg, 0.78 mmol) and IBX (30 wt.%, 1.46 g, 1.56 mmol) under air. H_2O (2.6 mL) and HFIP (2.6 mL) were then added, and the resulting solution (0.15 M) was stirred at 60 °C for 1.5 h. The reaction mixture was then allowed to cool to rt and sat. aq. $NaHCO_3$

(3 mL) was added. The aqueous layer was extracted with CH_2Cl_2 (3 × 5 mL). The combined organic extracts were dried over Na_2SO_4 and filtered. The solvent was removed under reduced pressure. The crude material was purified by flash column chromatography (30% to 40% acetone/pentane). The product containing fractions were combined and the solvent was removed under reduced pressure. Et_2O (5 mL) and pentane (5 mL) were added and the solvent was removed under reduced pressure to afford the product **41** as an off-white solid (153 mg, 62%).

R_f 0.23 (30% acetone/pentane);

mp = 200–204 °C (from Et_2O/pentane);

IR (film)/cm^{-1} 3191 (NH br), 2974, 1700 (C=O), 1670 (C=O), 1629, 1413, 1238, 1129, 865, 768;

^{1}H NMR (400 MHz, $CDCl_3$, 298 K, observed as a mixture of rotamers) δ 6.86 (dd, *J* = 10.1, 3.1 Hz, 1 H, (C*H*=CH)CO(CH=CH)), 6.69 (dd, *J* = 9.9, 3.0 Hz, 1 H, (CH=CH)CO(CH=C*H*)), 6.41–6.29 (m, 3 H, CO(C*H*=CH)$_2$, NH), 4.62 (br s, 1 H, NC*H*HCHCO), 4.35 (br s, 1 H, NC*H*HCH$_2$), 2.71 (br s, 1 H, NCH*H*CHCO), 2.64–2.51 (br m, 1 H, NCH*H*CH$_2$), 2.33 (ddd, *J* = 14.5, 10.9, 4.0 Hz, 1 H, CHCO), 2.13 (ddd, *J* = 12.9, 12.9, 3.2 Hz, 1 H, NCH$_2$CH$_2$C*H*), 1.62–1.50 (br m, 1 H, NCH$_2$C*H*H), 1.45 (s, 9 H, C(CH$_3$)$_3$), 1.36–1.26 (br m, 1 H, NCH$_2$CH*H*);

^{13}C NMR (101 MHz, $CDCl_3$, 298 K, observed as a mixture of rotamers) δ 184.4 (C=O ketone), 174.7 (C=O amide), 154.6 (C=O carbamate), 148.4 ((*C*H=CH)CO(CH=CH)), 145.0 ((CH=CH)CO(CH=*C*H)), 131.4 ((CH=CH)CO(*C*H=CH)), 130.8 ((CH=*C*H)CO(CH=CH)), 80.5 (*C*(CH$_3$)$_3$), 59.4 (CNH quat), 52.0 (NCH$_2$CH$_2$CH), 44.9 (br, N*C*H$_2$CHCO), 43.5 (*C*HCO), 43.2 (br, N*C*H$_2$CH$_2$), 28.3 (C(*C*H$_3$)$_3$), 25.1 (NCH$_2$*C*H$_2$);

HRMS (ESI$^-$) *m/z* Calculated for $C_{17}H_{21}N_2O_4$ [M–H] 317.1501; Found 317.1508.

trans-(±)-3',4-Dioxo-2',3',3a',4',5',6',7',7a'-octahydrospiro[cyclohexane-1,1'-pyrrolo[3,4-c]pyridine]-2,5-dien-5'-ium chloride (42)

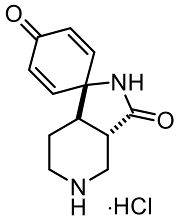

4 N HCl in 1,4-dioxane (0.9 mL, 3.60 mmol) was added to *N*-Boc protected derivative **41** (120 mg, 0.38 mmol) at 0 °C. The resulting solution was warmed up to 20 ºC and the product began to crush out of solution. The reaction mixture was stirred at 20 °C for 1 h and then ice-cold Et_2O (2 mL) was added. The reaction mixture was filtered, and the precipitate was washed with further ice-cold Et_2O (2 × 5 mL). The solid precipitate was found to be hygroscopic and turned quickly into a paste. This was then solubilised in MeOH, and the resulting solution was concentrated under reduced pressure. Et_2O (3 mL) and pentane (3 mL) were added and the solvent was removed under reduced pressure to afford to afford the deprotected product as the corresponding hydrochloride salt **42** as a pale yellow solid (92.8 mg, 96%).

mp = 270–274 °C (from Et_2O/pentane);

IR (film)/cm^{-1} 2769 (br NH), 1666 (C=O), 1625 (C=O), 1394, 1290, 1059, 861;

^{1}H NMR (400 MHz, CD_3OD, 298 K) δ 7.17 (dd, *J* = 10.3, 2.7 Hz, 1 H, (C*H*=CH)CO(CH=CH)), 6.88 (dd, *J* = 10.2, 2.8 Hz, 1 H, (CH=CH)CO(CH=C*H*)), 6.37 (d, *J* = 10.2 Hz, 2 H, CO(C*H*=CH)$_2$), 3.78 (dd, *J* = 11.6, 3.2 Hz, 1 H,

NC*H*HCHCO), 3.52 (br dd, *J* = 12.7, 3.6 Hz, 1 H, NC*H*HCH$_2$), 3.16 (dd, *J* = 11.9, 11.9 Hz, 1 H, NCH*H*CHCO), 3.00 (ddd, *J* = 12.7, 12.7, 3.5 Hz, 1 H, NCH*H*CH$_2$), 2.89 (ddd, *J* = 12.9, 12.9, 3.5 Hz, 1 H, CHCO), 2.56 (ddd, *J* = 12.8, 12.8, 3.0 Hz, 1 H, NCH$_2$CH$_2$C*H*), 1.81 (br d, *J* = 13.1 Hz, 1 H, NCH$_2$C*H*H), 1.66 (dddd, *J* = 13.2, 13.2, 13.2, 4.0 Hz, 1 H, NCH$_2$CH*H*);

^{13}C NMR (101 MHz, CD$_3$OD, 298 K) δ 186.4 (C=O ketone), 175.0 (C=O amide), 150.0 ((*C*H=CH)CO(CH=CH)), 147.2 ((CH=CH)CO(CH=*C*H)), 131.7 ((CH=CH)CO(*C*H=CH)), 131.5 ((CH=*C*H)CO(CH=CH)), 60.6 (CNH quat), 49.0 (NCH$_2$CH$_2$*C*H), 45.9 (N*C*H$_2$CHCO), 45.3 (N*C*H$_2$CH$_2$), 42.2 (*C*HCO), 23.7 (NCH$_2$*C*H$_2$);

HRMS (ESI$^+$) *m/z* Calculated for $C_{12}H_{15}N_2O_2$ [M–Cl] 219.1134; Found 219.1141.

<u>*Note:*</u> *Hygroscopic solid.*

Section 4: 4,3-Disubstituted Fragments

Synthesis overview

4,3-Disubstituted Piperidine Set (FRAG32-45)

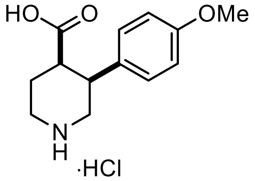

FRAG32

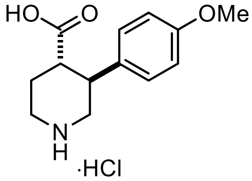

FRAG34

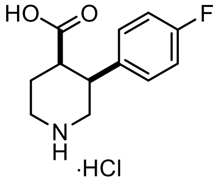

FRAG33

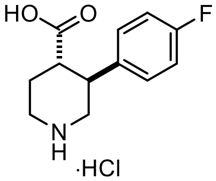

FRAG35

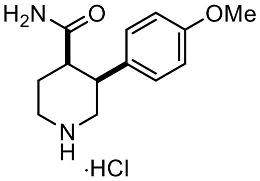

FRAG36

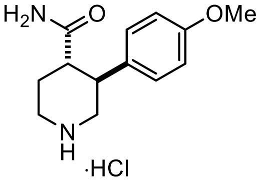

FRAG38

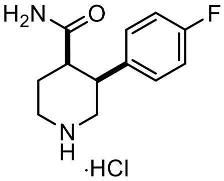

FRAG37

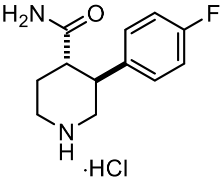

FRAG39

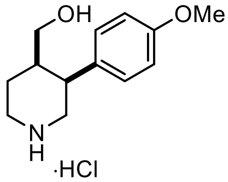

FRAG40

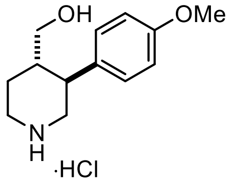

FRAG42

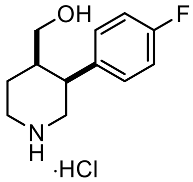

FRAG41

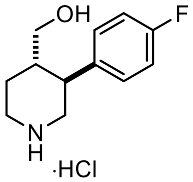

FRAG43

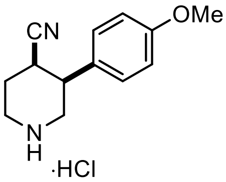

FRAG44

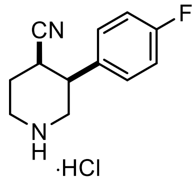

FRAG45

4,3-Disubstituted Heterocycle Set (FRAG46-51)

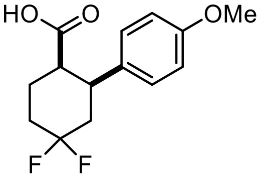

FRAG48

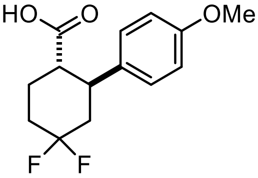

FRAG49

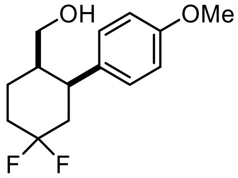

FRAG50

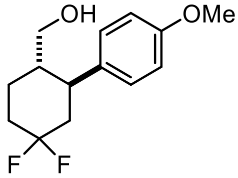

FRAG51

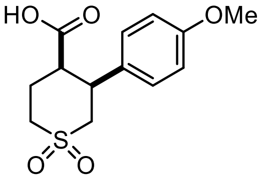

FRAG46

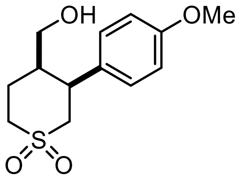

FRAG47

4,3-Disubstituted Fragments Synthetic Routes

CH arylation of aminoquinoline amides was performed as specified giving key intermediates **12a,b** and **13a,b**.

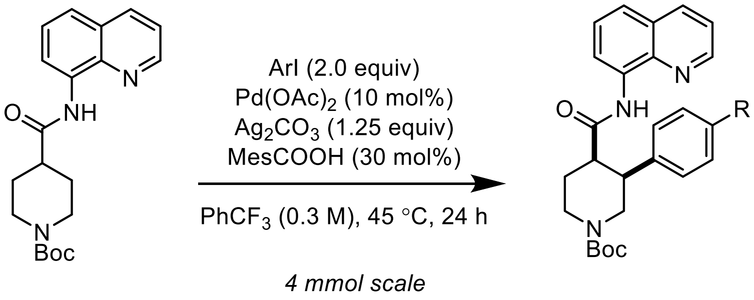

11

R = OMe, **12a**, 70% (1.35 g)
R = F, **12b**, 56% (0.98 g)

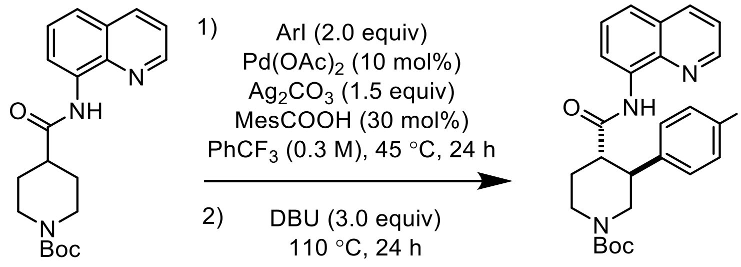

11 *4 mmol scale*

R = OMe, **13a**, 54% (0.92 g)
R = F, **12b + 13b**, (65% total)

NaOH

13b (57% over 3 steps)

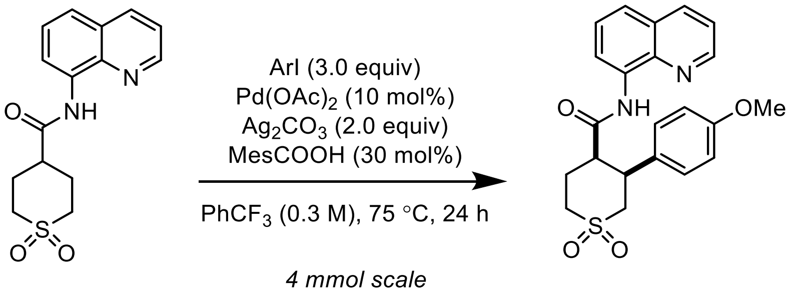

14

15a, 25% (0.41 g)

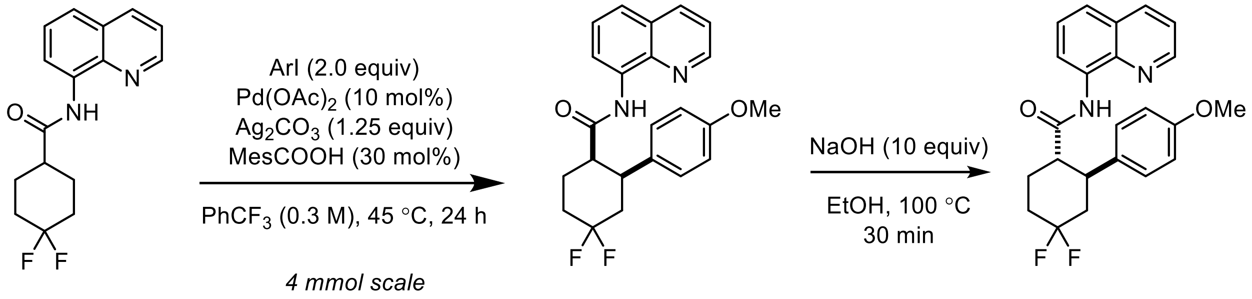

16 **17a**, 57% (0.96 g) **18a**, 84% (0.36 g)

The synthetic route used to access each piperidine fragment (**FRAG32-45**) from the corresponding arylated aminoquinoline amide is given below.

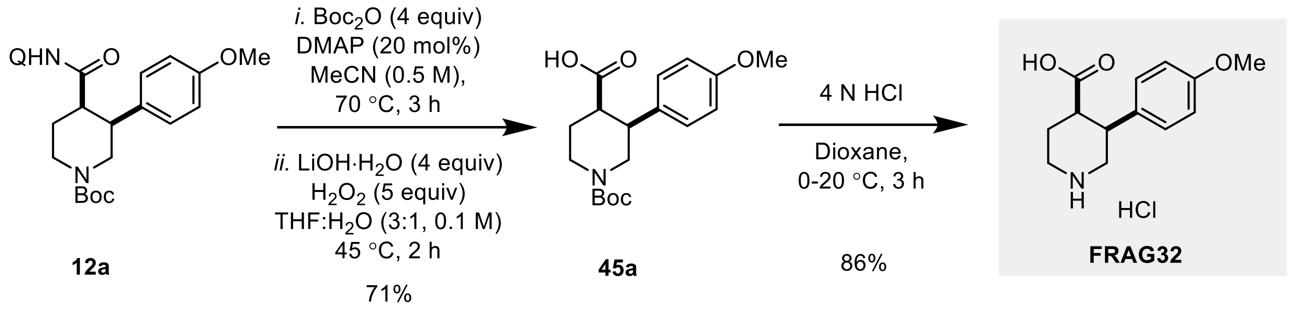

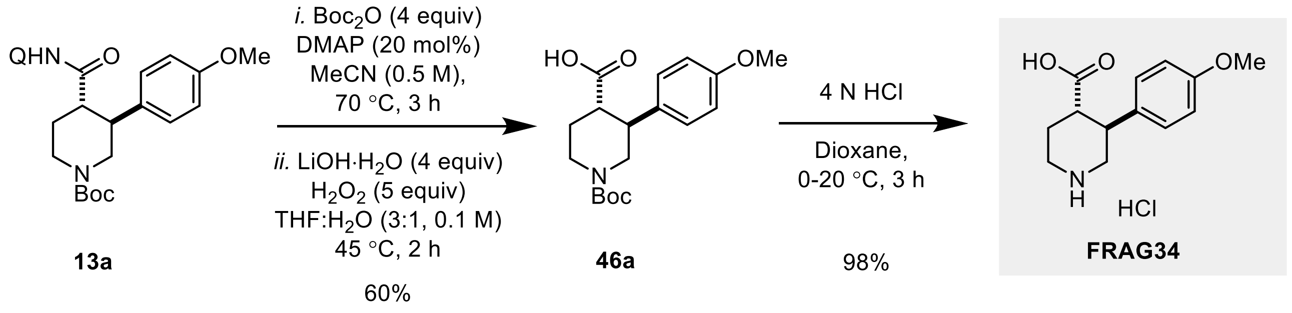

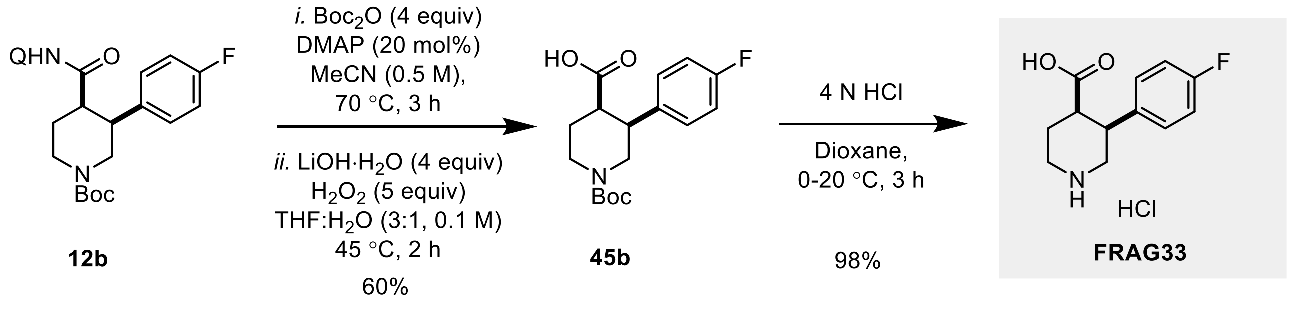

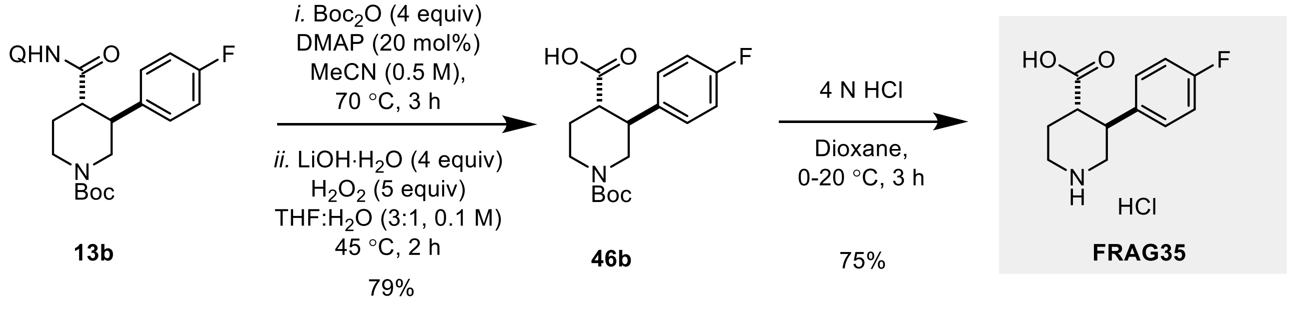

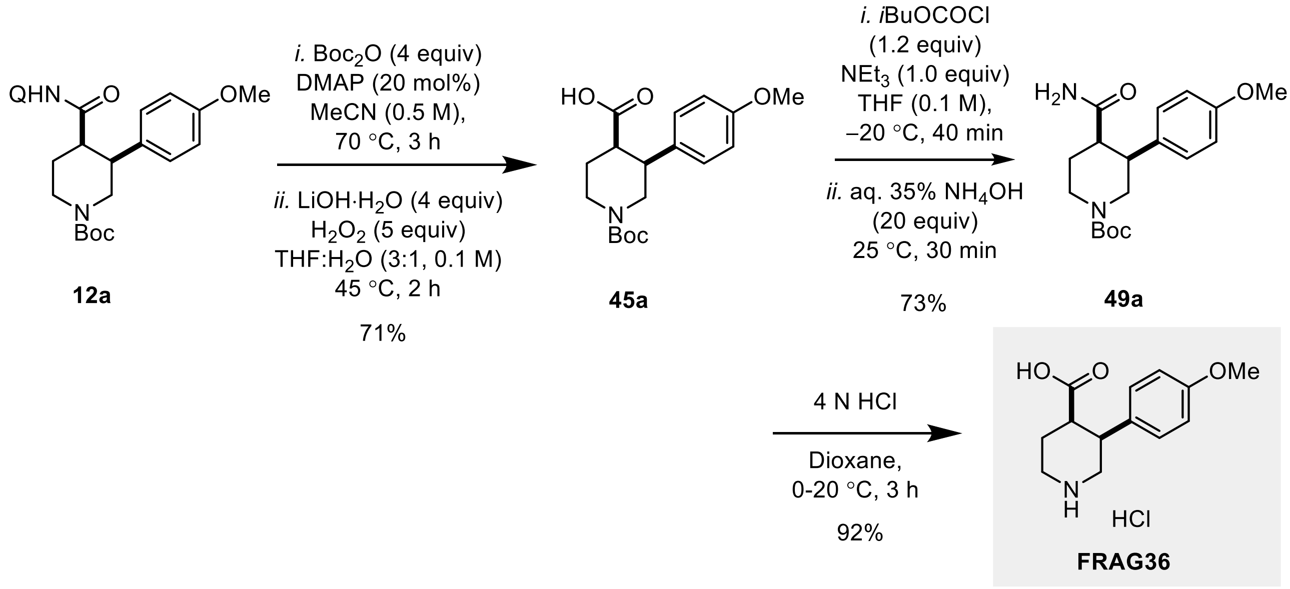
QHN
O
OMe
N
Boc
12a
i. Boc$_2$O (4 equiv)
DMAP (20 mol%)
MeCN (0.5 M),
70 °C, 3 h
ii. LiOH·H$_2$O (4 equiv)
H$_2$O$_2$ (5 equiv)
THF:H$_2$O (3:1, 0.1 M)
45 °C, 2 h
71%
HO
45a
i. *i*BuOCOCl
(1.2 equiv)
NEt$_3$ (1.0 equiv)
THF (0.1 M),
–20 °C, 40 min
ii. aq. 35% NH$_4$OH
(20 equiv)
25 °C, 30 min
73%
H$_2$N
49a
4 N HCl
Dioxane,
0-20 °C, 3 h
92%
H
HCl
FRAG36

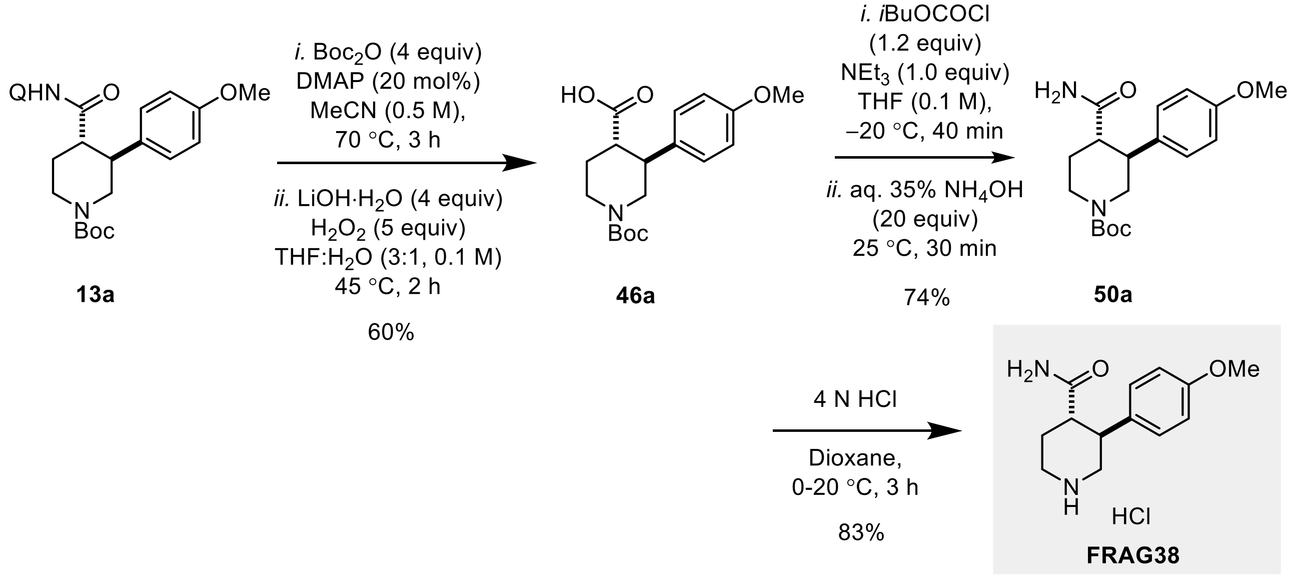
QHN
O
OMe
N
Boc
13a
i. Boc$_2$O (4 equiv)
DMAP (20 mol%)
MeCN (0.5 M),
70 °C, 3 h
ii. LiOH·H$_2$O (4 equiv)
H$_2$O$_2$ (5 equiv)
THF:H$_2$O (3:1, 0.1 M)
45 °C, 2 h
60%
HO
46a
i. *i*BuOCOCl
(1.2 equiv)
NEt$_3$ (1.0 equiv)
THF (0.1 M),
–20 °C, 40 min
ii. aq. 35% NH$_4$OH
(20 equiv)
25 °C, 30 min
74%
H$_2$N
50a
4 N HCl
Dioxane,
0-20 °C, 3 h
83%
H
HCl
FRAG38

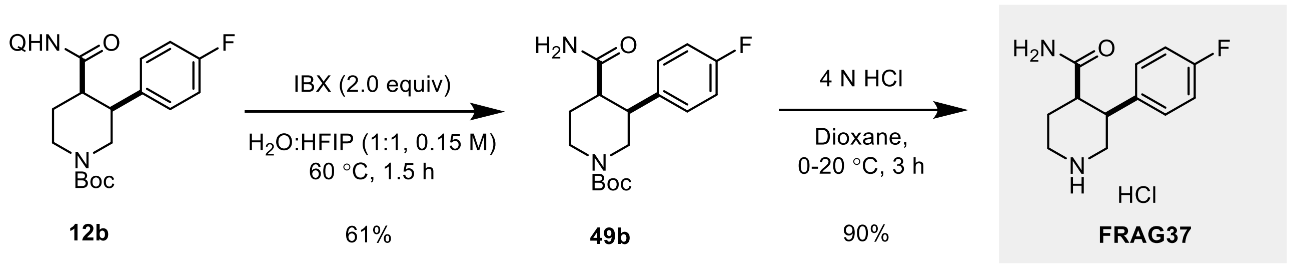
QHN
O
F
N
Boc
12b
IBX (2.0 equiv)
H$_2$O:HFIP (1:1, 0.15 M)
60 °C, 1.5 h
61%
H$_2$N
49b
4 N HCl
Dioxane,
0-20 °C, 3 h
90%
H
HCl
FRAG37

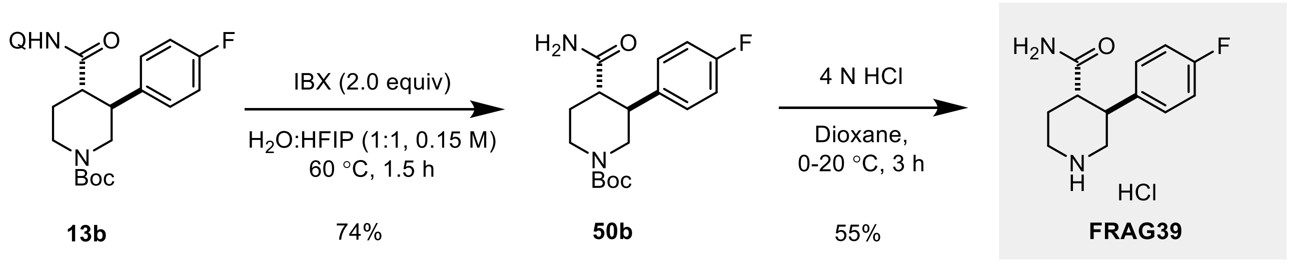
QHN
O
F
N
Boc
13b
IBX (2.0 equiv)
H$_2$O:HFIP (1:1, 0.15 M)
60 °C, 1.5 h
74%
H$_2$N
50b
4 N HCl
Dioxane,
0-20 °C, 3 h
55%
H
HCl
FRAG39

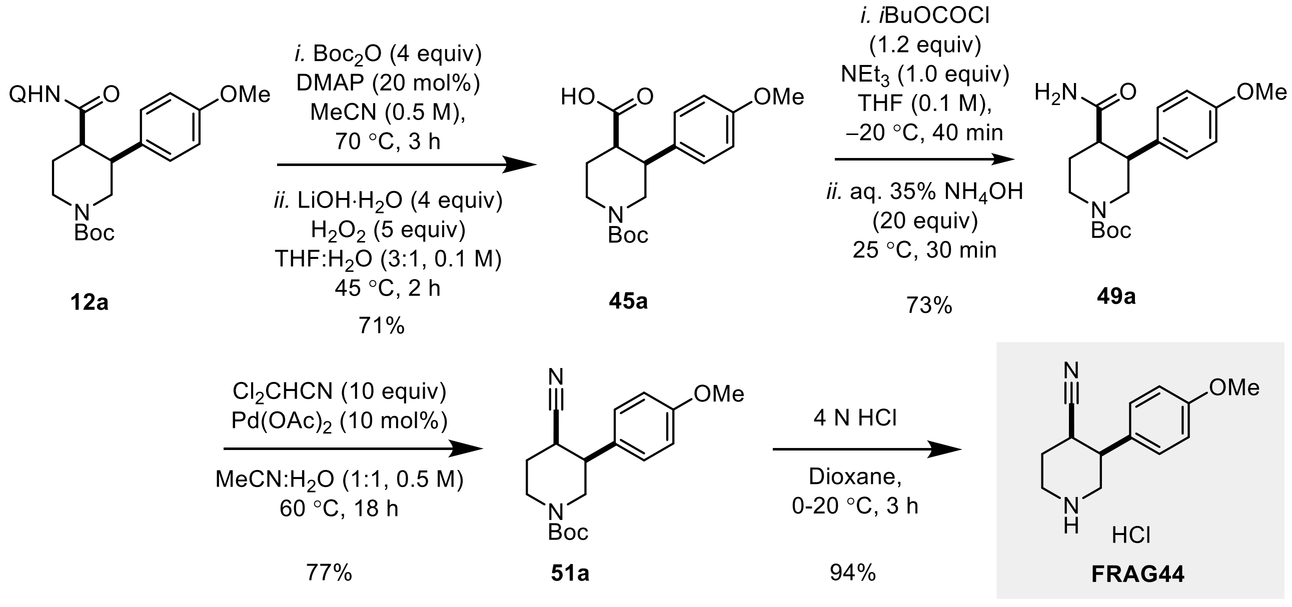
QHN
O
OMe
N
Boc
12a
i. Boc2O (4 equiv)
DMAP (20 mol%)
MeCN (0.5 M),
70 °C, 3 h
ii. LiOH·H2O (4 equiv)
H2O2 (5 equiv)
THF:H2O (3:1, 0.1 M)
45 °C, 2 h
71%
HO
45a
i. iBuOCOCl
(1.2 equiv)
NEt3 (1.0 equiv)
THF (0.1 M),
–20 °C, 40 min
ii. aq. 35% NH4OH
(20 equiv)
25 °C, 30 min
73%
H2N
49a
Cl2CHCN (10 equiv)
Pd(OAc)2 (10 mol%)
MeCN:H2O (1:1, 0.5 M)
60 °C, 18 h
77%
51a
4 N HCl
Dioxane,
0-20 °C, 3 h
94%
H
HCl
FRAG44

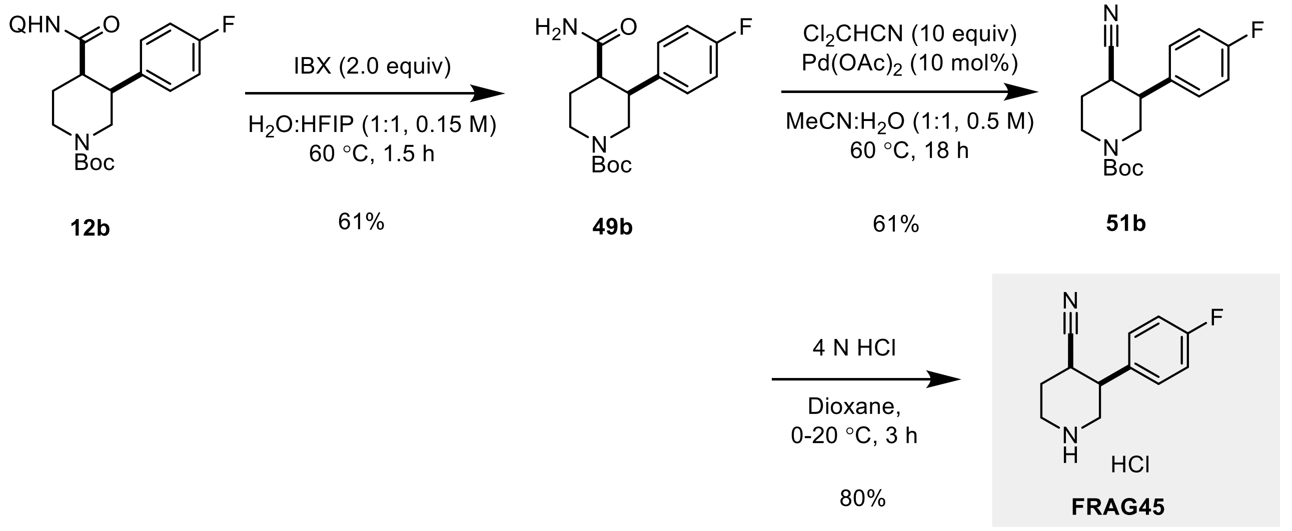
QHN
O
F
N
Boc
12b
IBX (2.0 equiv)
H2O:HFIP (1:1, 0.15 M)
60 °C, 1.5 h
61%
H2N
49b
Cl2CHCN (10 equiv)
Pd(OAc)2 (10 mol%)
MeCN:H2O (1:1, 0.5 M)
60 °C, 18 h
61%
51b
4 N HCl
Dioxane,
0-20 °C, 3 h
80%
H
HCl
FRAG45

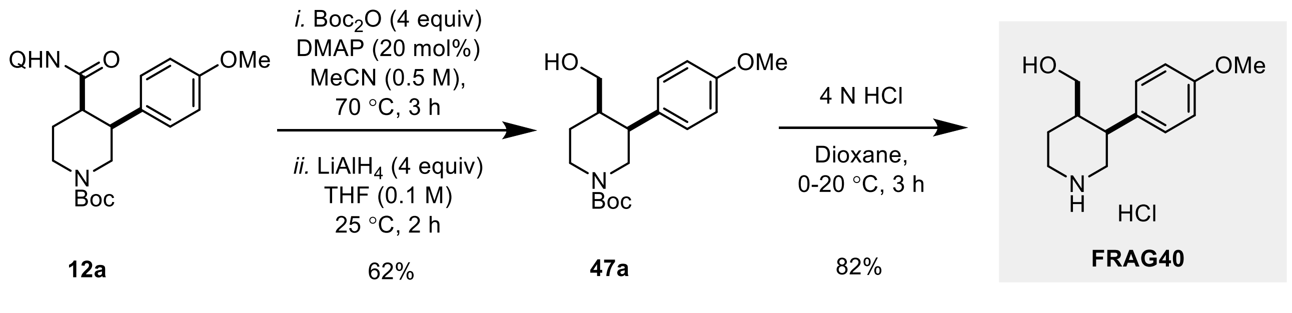
QHN
O
OMe
N
Boc
12a
i. Boc2O (4 equiv)
DMAP (20 mol%)
MeCN (0.5 M),
70 °C, 3 h
ii. LiAlH4 (4 equiv)
THF (0.1 M)
25 °C, 2 h
62%
HO
47a
4 N HCl
Dioxane,
0-20 °C, 3 h
82%
H
HCl
FRAG40

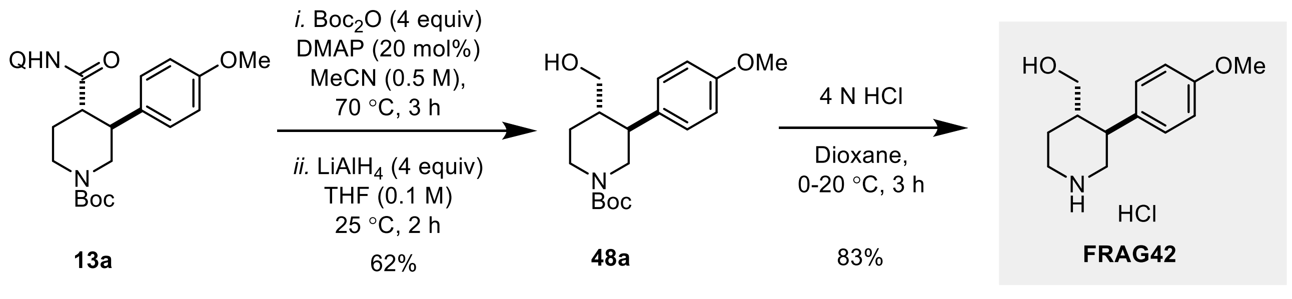
QHN
O
OMe
N
Boc
13a
i. Boc2O (4 equiv)
DMAP (20 mol%)
MeCN (0.5 M),
70 °C, 3 h
ii. LiAlH4 (4 equiv)
THF (0.1 M)
25 °C, 2 h
62%
HO
48a
4 N HCl
Dioxane,
0-20 °C, 3 h
83%
H
HCl
FRAG42

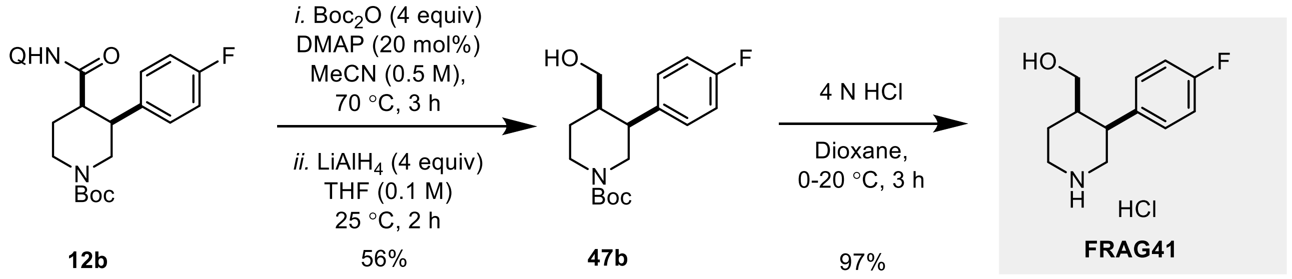
QHN
O
F
N
Boc
12b
i. Boc2O (4 equiv)
DMAP (20 mol%)
MeCN (0.5 M),
70 °C, 3 h
ii. LiAlH4 (4 equiv)
THF (0.1 M)
25 °C, 2 h
56%
HO
47b
4 N HCl
Dioxane,
0-20 °C, 3 h
97%
H
HCl
FRAG41

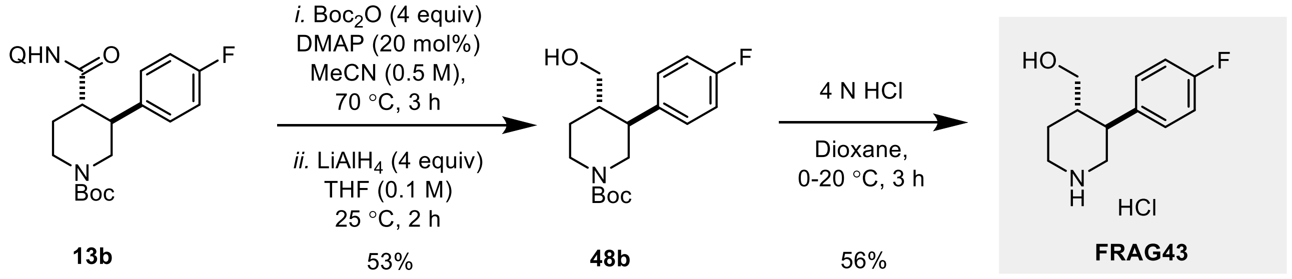
QHN
O
F
N
Boc
13b
i. Boc2O (4 equiv)
DMAP (20 mol%)
MeCN (0.5 M),
70 °C, 3 h
ii. LiAlH4 (4 equiv)
THF (0.1 M)
25 °C, 2 h
53%
HO
48b
4 N HCl
Dioxane,
0-20 °C, 3 h
56%
H
HCl
FRAG43

The synthetic route used to access each difluorocyclohexane and sulfone fragments (**FRAG46-51**) from the corresponding arylated aminoquinoline amide is given below.

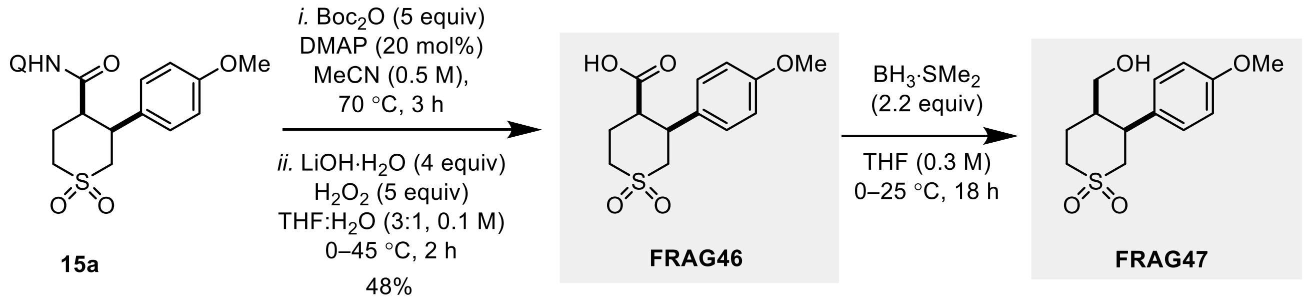

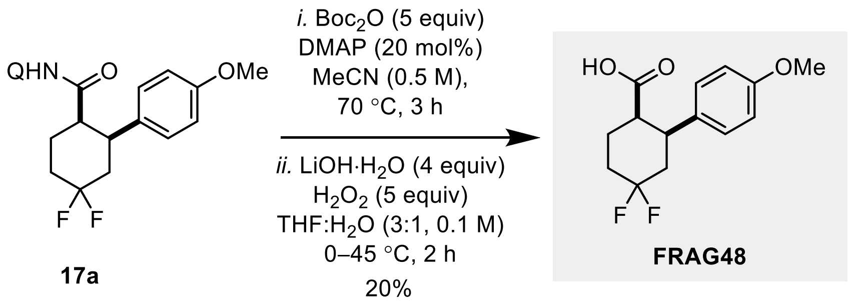

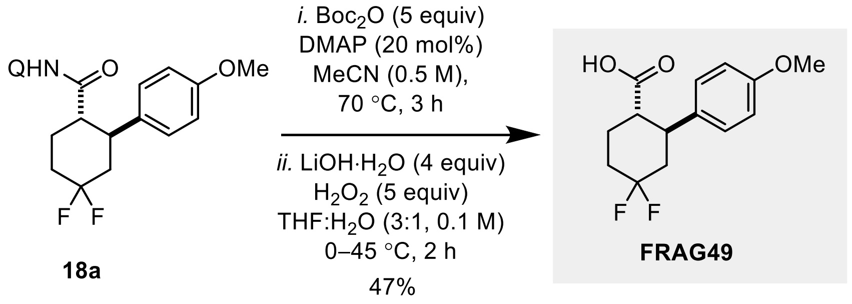

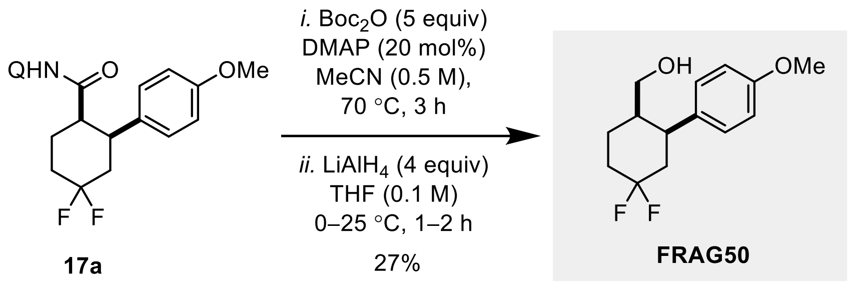

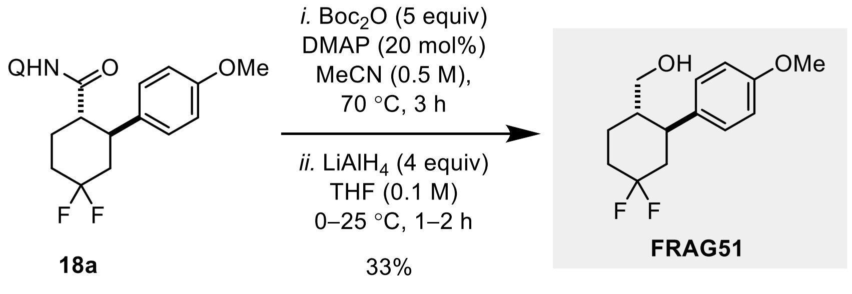

C4-Directed CH Arylations

tert-Butyl-cis-(±)-3-(4-methoxyphenyl)-4-(quinolin-8-ylcarbamoyl)piperidine-1-carboxylate (12a)

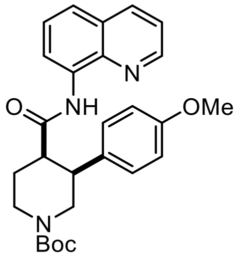

According to the procedure reported by Bull,[25] a flask was charged with aminoquinoyl amide **11** (1.42 g, 4.00 mmol, 1.0 equiv), 4-iodoanisole (1.87 g, 8.00 mmol, 2.0 equiv), Ag_2CO_3 (1.38 g, 5.00 mmol, 1.25equiv), 2,4,6-trimethylbenzoic acid (MesCOOH, 197 mg, 1.2 mmol, 0.3 equiv) and $Pd(OAc)_2$ (90 mg, 0.4 mmol, 0.1 equiv) in this order. The reaction vessel was covered with a suba seal and purged with argon, then $PhCF_3$ (13.4 mL, 0.3 M) was added by syringe. The flask was placed in a preheated oil bath and stirred at 45 ºC for 18 h under an atmosphere of argon. The mixture was then allowed to cool to rt and EtOAc (20 mL) was added before filtering through a pad of Celite, eluting with further EtOAc (2 × 50 mL). The solvent was removed under reduced pressure and the residue purified by flash column chromatography (2%-20% acetone/pentane). Et_2O (5 mL) and pentane (10 mL) were added and the solvent was removed under reduced pressure to afford *cis* arylated piperidine **12a** as a white solid (1.34 g, 2.91 mmol, 70%).

R_f 0.34 (20% acetone/pentane);

mp = 135–137 °C (from Et_2O/pentane);

IR (film)/cm^{-1} 3348 (NH br), 2933, 2974, 1681 (C=O), 1528, 1483, 1423, 1245 (C–O), 1163 (C–O), 910, 828, 731;

^{1}H NMR (500 MHz, DMSO-d_6, 373 K) δ 9.62 (br s, 1 H, NH), 8.80 (dd, *J* = 4.2, 1.7 Hz, 1 H, HC_{Ar}), 8.46 (dd, *J* = 7.7, 1.4 Hz, 1 H, HC_{Ar}), 8.31 (dd, *J* = 7.7, 1.5 Hz, 1 H, HC_{Ar}), 7.58 (dd, *J* = 8.3, 1.3 Hz, 1 H, HC_{Ar}), 7.55 (dd, *J* = 8.3, 4.2 Hz, 1 H, HC_{Ar}), 7.50 (t, *J* = 7.9 Hz, 1 H, HC_{Ar}), 7.22–7.17 (m, 2 H, 2 × HC_{Ar}), 6.72–6.66 (m, 2 H, 2 × HC_{Ar}), 4.00 (dd, *J* = 13.0, 8.1 Hz, 1 H, NC*H*HCHAr), 3.82 (ddd, *J* = 12.9, 8.6, 3.8 Hz, 1 H, NC*H*HCH$_2$), 3.73 (dd, *J* = 13.0, 4.1 Hz, 1 H, NCH*H*CHAr), 3.58 (s, 3 H, OCH_3), 3.54 (ddd, *J* = 12.7, 7.0, 4.7 Hz, 1 H, NCH*H*CH$_2$), 3.31 (ddd, *J* = 6.3, 4.7, 4.5 Hz, 1 H, CH(C=O)), 3.23 (ddd, *J* = 8.7, 6.7, 4.1 Hz, 1 H, CHAr), 2.05 (ddt, *J* = 13.1, 6.5, 3.8 Hz, 1 H, NCH$_2$C*H*H), 1.90 (ddt, *J* = 13.3, 8.6, 4.6 Hz, 1 H, NCH$_2$CH*H*), 1.43 (s, 9 H, $C(CH_3)_3$);

^{13}C NMR (126 MHz, DMSO-d_6, 373 K) δ 171.4 (C=O amide), 157.6 (C_{Ar} quat), 153.7 (C=O carbamate), 147.9 (C_{Ar}), 137.6 (C_{Ar} quat), 135.7 (C_{Ar}), 133.7 (C_{Ar} quat), 132.6 (C_{Ar} quat), 128.3 (2 × C_{Ar}), 127.2 (C_{Ar} quat), 126.2 (C_{Ar}), 121.3 (C_{Ar}), 121.0 (C_{Ar}), 115.9 (C_{Ar}), 113.2 (2 × C_{Ar}), 78.2 (*C*(CH$_3$)$_3$), 54.4 (OCH_3), 45.43 (N*C*H$_2$CHAr), 45.36 (*C*H(C=O)), 41.4 (CHAr), 40.5 (N*C*H$_2$CH$_2$), 27.7 (C(*C*H$_3$)$_3$), 25.6 (NCH$_2$*C*H$_2$);

HRMS (ESI) *m/z* Calculated for $C_{27}H_{32}N_3O_4$ [M+H] 462.2393; Found 462.2388.

Characterisation information consistent with data previously reported in literature.[25]

tert-Butyl-cis-(±)-3-(4-fluorophenyl)-4-(quinolin-8-ylcarbamoyl)piperidine-1-carboxylate (12b)

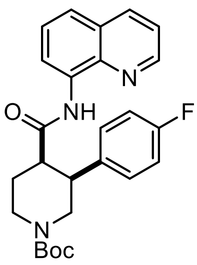

According to the procedure reported by Bull,[25] a flask was charged with aminoquinoyl amide **11** (1.42 g, 4.00 mmol, 1.0 equiv), 1-fluoro-4-iodobenzene (1.78 g, 8.00 mmol, 2.0 equiv), Ag_2CO_3 (1.38 g, 5.00 mmol, 1.25 equiv), MesCOOH (197 mg, 1.2 mmol, 0.3 equiv) and $Pd(OAc)_2$ (90 mg, 0.4 mmol, 0.1 equiv) in this order. The reaction vessel was covered with a suba seal and purged with argon, then $PhCF_3$ (13.4 mL, 0.3 M) was added by syringe. The flask was placed in a preheated oil bath and stirred at 45 ºC for 18 h under an atmosphere of argon. The mixture was then allowed to cool to rt and EtOAc (20 mL) was added before filtering through a pad of Celite, eluting with further EtOAc (2 × 50 mL). The solvent was removed under reduced pressure and the residue purified by sequential flash column chromatography (5%-8% Et_2O/CH_2Cl_2, followed by 20% acetone/pentane). Et_2O (5 mL) and pentane (10 mL) were added and the solvent was removed under reduced pressure to afford *cis* arylated piperidine **12b** as a yellow foamy solid (980 mg, 2.18 mmol, 56%).

R_f 0.47 (10% Et_2O/CH_2Cl_2);

mp = 68–70 °C (from Et_2O/pentane);

IR (film)/cm^{-1} 3342 (NH br), 3001, 2972, 2924, 1675 (C=O), 1521 (C=O), 1481, 1420, 1365, 1226, 1158, 1111, 906, 826, 728;

^{1}H NMR (400 MHz, DMSO-*d₆*, 373 K) δ 9.64 (br s, 1 H, NH), 8.81 (dd, *J* = 4.2, 1.7 Hz, 1 H, HC_{Ar}), 8.42 (dd, *J* = 7.6, 1.3 Hz, 1 H, HC_{Ar}), 8.31 (ddd, *J* = 8.3, 1.7, 0.4 Hz, 1 H, HC_{Ar}), 7.61–7.53 (m, 2 H, 2 × HC_{Ar}), 7.49 (t, *J* = 7.9 Hz, 1 H, HC_{Ar}), 7.34–7.27 (m, 2 H, 2 × HC_{Ar}), 6.96–6.89 (m, 2 H, 2 × HC_{Ar}), 4.01 (dd, *J* = 13.0, 8.0 Hz, 1 H, NC*H*HCHAr), 3.81 (ddd, *J* = 12.7, 8.5, 3.9 Hz, 1 H, NC*H*HCH₂), 3.73 (dd, *J* = 13.0, 4.1 Hz, 1 H, NCH*H*CHAr), 3.53 (ddd, *J* = 13.1, 6.7, 4.3 Hz, 1 H, NCH*H*CH_2), 3.35 (dt, *J* = 6.6, 4.8 Hz, 1 H, CH(C=O)), 3.29 (dt, *J* = 8.6, 4.5 Hz, 1 H, CHAr), 2.05 (dtd, *J* = 13.5, 6.6, 3.8 Hz, 1 H, NCH_2C*H*H), 1.90 (ddt, *J* = 13.3, 8.8, 4.5 Hz, 1 H, NCH_2CH*H*), 1.42 (s, 9 H, $C(CH_3)_3$);

^{13}C NMR (126 MHz, DMSO-*d₆*, 373 K) δ 171.2 (C=O amide), 160.6 (d, J_{CF} = 246.7 Hz, FC_{Ar} quat), 153.7 (C=O carbamate), 148.0 (C_{Ar}), 137.7 (C_{Ar} quat), 136.7 (d, J_{CF} = 3.4 Hz, F-*p*-C_{Ar} quat), 135.8 (C_{Ar}), 133.6 (C_{Ar} quat), 129.1 (d, J_{CF} = 8.1 Hz, 2 × F-*m*-C_{Ar}), 127.2 (C_{Ar} quat), 126.2 (C_{Ar}), 121.3 (C_{Ar}), 121.1 (C_{Ar}), 116.0 (C_{Ar}), 114.1 (d, J_{CF} = 21.0 Hz, 2 × F-*o*-C_{Ar}), 78.3 (*C*(CH_3)₃), 45.3 (NCH_2CHAr), 45.1 (*C*H(C=O)), 41.3 (*C*HAr), 40.5 (NCH_2CH_2), 27.6 (C(*C*H₃)₃), 25.5 (NCH_2CH_2);

^{19}F{^{1}H} NMR (471 MHz, DMSO-*d₆*, 373 K) d –116.69 (s);

HRMS (ESI) *m/z* Calculated for $C_{26}H_{29}FN_3O_3$ [M+H] 450.2193; Found 450.2191.

Characterisation information consistent with data previously reported in literature.[25]

tert-Butyl-trans-(±)-3-(4-methoxyphenyl)-4-(quinolin-8-ylcarbamoyl)piperidine-1-carboxylate (13a)

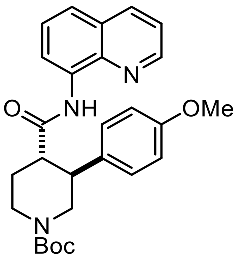

According to the procedure reported by Bull,[25] a flame-dried microwave vial (25 mL) was charged sequentially with amide **11** (710 mg, 2.00 mmol, 1.0 equiv), 4-iodoanisole (936 mg, 4.00 mmol, 2.0 equiv), Ag_2CO_3 (690 mg, 2.50 mmol, 1.25 equiv), MesCOOH (99 mg, 0.60 mmol, 0.3 equiv) and $Pd(OAc)_2$ (45 mg, 0.20 mmol, 0.1 equiv) in this order. The reaction vessel was sealed and purged with argon, then $PhCF_3$ (6.7 mL, 0.3 M) was added by syringe. The reaction tube was placed in a preheated oil bath and stirred at 45 °C for 24 h. The reaction mixture was then allowed to cool to rt and 1,8-diazabicyclo[5.4.0]undec-7-ene (DBU, 0.9 mL, 6.00 mmol, 3.0 equiv) was added by syringe. The reaction vessel was then stirred at 110 °C for additional 24 h. The reaction was allowed to cool to rt and EtOAc (20 mL) was added. The resulting mixture was filtered through a pad of Celite, eluting with further EtOAc (2 × 50 mL). The solvent was removed under reduced pressure, and the crude material was purified by flash column chromatography (5%-20% acetone/pentane) to afford a mixture of mono-*trans* arylated piperidine **13a** and di-*cis-cis* arylated piperidine. A second purification by column chromatography (10% Et_2O/CH_2Cl_2) was conducted and the fractions containing the *trans*-product were combined and concentrated under reduced pressure. Et_2O (10 mL) and pentane (10 mL) were added and the solvent was removed under reduced pressure to afford the *trans*-arylated piperidine **13a** as a white powder (498 mg, 1.08 mmol, 54%).

R_f 0.37 (10% Et_2O/CH_2Cl_2);

mp = 99–101 °C (from Et_2O/pentane);

IR (film)/cm^{-1} 3347 (NH br), 3045, 2931, 2858, 2837, 1663 (C=O), 1526, 1485, 1422, 1366, 1245, 1157, 926, 732;

^{1}H NMR (400 MHz, DMSO-d_6, 373 K) δ 9.71 (br s, 1 H, NH), 8.86 (dd, J = 4.2, 1.7 Hz, 1 H, HC_{Ar}), 8.43 (dd, J = 7.7, 1.4 Hz, 1 H, HC_{Ar}), 8.34–8.28 (m, 1 H, HC_{Ar}), 7.57 (ddd, J = 8.3, 2.8, 1.5 Hz, 2 H, HC_{Ar}), 7.46 (dd, J = 7.9, 7.8 Hz, 1 H, HC_{Ar}), 7.33–7.23 (m, 2 H, HC_{Ar}), 6.83–6.74 (m, 2 H, HC_{Ar}), 4.13 (ddd, J = 11.0, 4.3, 2.2 Hz, 1 H, NC*H*HCH$_2$), 4.00 (dd, J = 10.1, 8.8 Hz, 1 H, NC*H*HCHAr), 3.62 (s, 3 H, OCH_3), 3.22 (td, J = 11.4, 3.7 Hz, 1 H, CH(C=O)), 3.02–2.99 (m, 1 H, NCH*H*CH$_2$), 2.95–2.85 (m, 2 H, NCH*H*CHAr and CHAr), 2.04 (dq, J = 13.2, 3.2 Hz, 1 H, NCH$_2$C*H*H), 1.81–1.64 (m, 1 H, NCH$_2$CH*H*), 1.46 (s, 9 H, C(C*H*$_3$)$_3$);

^{13}C NMR (101 MHz, DMSO-d_6, 373 K) δ 171.6 (C=O amide), 157.8 (C_{Ar} quat), 153.5 (C=O carbamate), 148.0 (C_{Ar}), 137.6 (C_{Ar} quat), 135.8 (C_{Ar}), 133.8 (C_{Ar} quat), 132.7 (C_{Ar} quat), 128.0 (2 x C_{Ar}), 127.2 (C_{Ar} quat), 126.2 (C_{Ar}), 121.4 (C_{Ar}), 121.1 (C_{Ar}), 115.8 (C_{Ar}), 113.6 (2 × C_{Ar}), 78.4 (*C*(CH$_3$)$_3$), 54.5 (OCH_3), 49.3 (N*C*H$_2$CHAr), 49.1 (*C*H(C=O)), 43.6 (CHAr), 42.6 (N*C*H$_2$CH$_2$), 28.8 (NCH$_2$*C*H$_2$) 27.7 (C(*C*H$_3$)$_3$);

HRMS (ESI) *m/z* Calculated for $C_{27}H_{32}N_3O_4$ [M+H] 462.2393; Found 462.2396.

Characterisation information consistent with data previously reported in literature.[25]

tert-Butyl-trans-(±)-3-(4-fluorophenyl)-4-(quinolin-8-ylcarbamoyl)piperidine-1-carboxylate (13b)

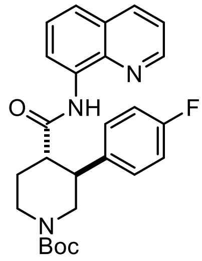

A flame-dried microwave vial (25 mL) was charged sequentially with aminoquinoyl amide **11** (1.42 g, 4.00 mmol, 1.0 equiv), 4-fluoroiodobenzene (1.77 g, 8.00 mmol, 2.0 equiv), Ag_2CO_3 (1.38 g, 5.00 mmol, 1.25 equiv), MesCOOH (197 mg, 1.20 mmol, 0.3 equiv) and $Pd(OAc)_2$ (90 mg, 0.40 mmol, 0.1 equiv) in this order. The reaction vessel was sealed and purged with argon, then $PhCF_3$ (13.4 mL) was added by syringe. The reaction tube was placed in a preheated oil bath and stirred at 45 °C for 24 h. The reaction mixture was then allowed to cool to rt and 1,8-diazabicyclo[5.4.0]undec-7-ene (DBU, 1.8 mL, 12.00 mmol, 3.0 equiv) was added by syringe. The reaction was stirred at 110 °C for additional 24 h before cooling to rt and diluting with EtOAc (20 mL). The resulting mixture was filtered through a pad of Celite, eluting with further EtOAc (2 × 50 mL), and the solvent removed under reduced pressure. The residue was purified by flash column chromatography (5% to 10% Et_2O/CH_2Cl_2) to afford a mixture of mono-*trans* and mono-*cis* arylated piperidines (1.16 g, combined yield 65%, d.r. 7:3 *cis*:*trans*) which was subjected to NaOH-mediated epimerisation to promote full conversion of the remaining mono-*cis* isomer to mono-*trans*.

NaOH (480 mg, 12.00 mmol, 10.0 equiv) was added to the mixture of *cis* and *trans*-arylated piperidines from above. The reaction vessel was sealed and purged with argon, then EtOH (18 mL) was added by syringe. The reaction tube was gradually heated to 100 °C in an oil bath, followed by stirring at 100 °C for 30 min. The mixture was then allowed to cool down to rt and the solvent evaporated. EtOAc (10 mL) and distilled water (10 mL) were added and the organic phase separated and washed with brine (2 × 5 mL). The organic phase was dried over Na_2SO_4, filtered and concentrated under reduced pressure to afford *trans*-arylated piperidine **13b** as an orange solid which did not require further purification (1.03 g, 2.28 mmol, 57% from **11**).

R_f 0.33 (20% acetone/pentane);

mp = 137–139 °C (from acetone/pentane);

IR (film)/cm^{-1} 3342 (NH br), 3041, 2968, 2920, 2853, 1683 (C=O), 1525, 1482, 1420, 1387, 1325, 1234, 1157, 828, 791, 731;

^{1}H NMR (400 MHz, DMSO-d_6, 373 K) δ 9.72 (br s, 1 H, NH), 8.86 (dd, J = 4.2, 1.7 Hz, 1 H, HC_{Ar}), 8.41 (dd, J = 7.7, 1.4 Hz, 1 H, HC_{Ar}), 8.31 (ddd, J = 8.3, 1.7, 0.4 Hz, 1 H, HC_{Ar}), 7.59–7.57 (m, 1 H, HC_{Ar}), 7.57–7.55 (m, 1 H, HC_{Ar}), 7.46 (dd, J = 8.2, 7.7 Hz, 1 H, HC_{Ar}), 7.43–7.36 (m, 2 H, HC_{Ar}), 7.05–6.98 (m, 2 H, HC_{Ar}), 4.14 (ddt, J = 13.3, 4.4, 2.3 Hz, 1 H, NC*H*HCHAr), 4.06–3.98 (m, 1 H, NC*H*HCH$_2$), 3.32–3.22 (m, 1 H, NCH*H*CHAr), 3.03–2.99 (m, 1 H, NCH*H*CH$_2$), 2.96–2.93 (m, 2 H, CH(C=O) and CHAr), 2.10–2.01 (m, 1 H, NCH$_2$C*H*H), 1.74 (tdd, J = 12.9, 11.6, 4.5 Hz, 1 H, NCH$_2$CH*H*), 1.46 (s, 9 H, C(C*H$_3$*)$_3$);

^{13}C NMR (101 MHz, DMSO-d_6, 373 K) δ 171.4 (C=O amide), 160.6 (d, J_{CF} = 246.7 Hz, FC_{Ar} quat), 153.5 (C=O carbamate), 148.1 (C_{Ar}), 137.7 (C_{Ar}), 136.8 (d, J_{CF} = 3.4 Hz, F-*p*-C_{Ar} quat), 135.8 (C_{Ar} quat), 133.7 (C_{Ar} quat), 128.9 (d, J_{CF} = 8.1 Hz, 2 × F-*m*-C_{Ar}), 127.2 (C_{Ar} quat), 126.2 (C_{Ar}), 121.4 (C_{Ar}), 121.2 (C_{Ar}), 116.0 (C_{Ar}), 114.5 (d, J_{CF} = 21.0 Hz, 2 × F-*o*-C_{Ar}), 78.5 (*C*(CH$_3$)$_3$), 49.0 (*C*H(C=O)), 48.9 (N*C*H$_2$CHAr), 43.7 (CHAr), 42.5 (N*C*H$_2$CH$_2$), 28.7 (NCH$_2$*C*H$_2$), 27.7 (C(*C*H$_3$)$_3$);

^{19}F NMR (376 MHz, DMSO-d_6, 373 K) δ -116.2;

HRMS (ESI) *m/z* Calculated for $C_{26}H_{29}N_3O_3F$ [M+H] 450.2193; Found 450.220.

cis-(±)-3-(4-Methoxyphenyl)-N-(quinolin-8-yl)tetrahydro-2H-thiopyran-4-carboxamide 1,1-dioxide (15a)

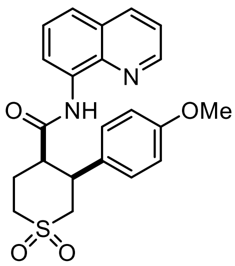

A flask was charged with aminoquinoyl amide **14** (1.21 g, 4.00 mmol, 1.0 equiv), 4-iodoanisole (2.80 g, 12.0 mmol, 3.0 equiv), Ag_2CO_3 (2.20 g, 8.00 mmol, 2.0 equiv), MesCOOH (197 mg, 1.20 mmol, 0.3 equiv) and $Pd(OAc)_2$ (90 mg, 0.40 mmol, 0.1 equiv) in this order. The reaction vessel was covered with a suba seal and purged with argon, then $PhCF_3$ (13.34 mL, 0.3 M) was added by syringe. The flask was placed in a preheated oil bath and stirred at 75 °C for 24 h. The mixture was then allowed to cool to rt and diluted with EtOAc (10 mL) before filtering through a pad of Celite, eluting with further EtOAc (2 × 15 mL). The solvent was removed under reduced pressure and the crude material purified by flash column chromatography (40-60% acetone/pentane). Et_2O (5 mL), followed by pentane (5 mL) were added to the isolated product, with the resulting suspension concentrated under reduced pressure. This procedure was repeated three times to eliminate residual solvent and afford **15a** as a pale pink crystalline solid (409 mg, 1.00 mmol, 25%).

R_f 0.31 (40% acetone/pentane);

mp = 118–120 °C (from Et_2O/pentane);

IR (film)/cm^{-1} 3332 (br NH), 3049, 2930, 2833, 1670 (C=O), 1521, 1379, 1252 (S=O), 1118 (S=O), 1029, 910, 828, 731;

^{1}H NMR (400 MHz, $CDCl_3$) δ 9.32 (br s, 1 H, NH), 8.67–8.60 (m, 2 H, 2 × HC_{Ar}), 8.12 (dd, *J* = 8.3, 1.7 Hz, 1 H, HC_{Ar}), 7.55–7.49 (m, 2 H, 2 × HC_{Ar}), 7.41 (dd, *J* = 8.3, 4.3 Hz, 1 H, HC_{Ar}), 7.20–7.11 (m, 2 H, 2 × HC_{Ar}), 6.74–6.66 (m, 2 H, 2 × HC_{Ar}), 4.44 (t, *J* = 13.4 Hz, 1 H, SC*H*HCHAr), 4.05 (td, *J* = 13.7, 4.1 Hz, 1 H, SC*H*HCH$_2$), 3.81 (dt, *J* = 13.4, 3.6 Hz, 1 H, CHAr), 3.58 (s, 3 H, OCH_3), 3.09 (dt, *J* = 13.5, 3.3 Hz, 1 H, SCH*H*CHAr), 3.07–2.99 (m, 2 H, CH(C=O) and SCH*H*CH$_2$), 2.64 (tt, *J* = 13.4, 4.1 Hz, 1 H, SCH$_2$C*H*H), 2.53 (dq, *J* = 14.8, 3.6 Hz, 1 H, SCH$_2$CH*H*);

^{13}C NMR (101 MHz, $CDCl_3$) δ 170.9 (C=O amide), 158.9 (C_{Ar} quat), 148.0 (C_{Ar}), 138.1 (C_{Ar} quat), 136.2 (C_{Ar}), 133.6 (C_{Ar}), 131.7 (C_{Ar} quat), 128.2 (2 × C_{Ar}), 127.8 (C_{Ar} quat), 127.1 (C_{Ar}), 122.1 (C_{Ar}), 121.6 (C_{Ar}), 116.6 (C_{Ar} quat), 114.3 (2 × C_{Ar}), 55.0 (OCH_3), 51.8 (S*C*H$_2$CHAr), 47.0 (S*C*H$_2$CH$_2$), 46.0 (*C*H(C=O)), 43.5 (CHAr), 27.3 (SCH$_2$*C*H$_2$);

HRMS (ESI) *m/z* Calculated for $C_{22}H_{23}N_2O_4S$ [M+H] 411.1379; Found 411.1382.

cis-(±)-4,4-Difluoro-2-(4-methoxyphenyl)-N-(quinolin-8-yl)cyclohexane-1-carboxamide (17a)

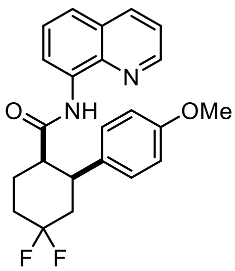

A flask was charged with aminoquinoyl amide **16** (1.16 g, 4.00 mmol, 1.0 equiv), 4-iodoanisole (1.87 g, 8.00 mmol, 2.0 equiv), Ag_2CO_3 (1.38 g, 5.00 mmol, 1.25 equiv), MesCOOH (197 mg, 1.20 mmol, 0.3 equiv) and $Pd(OAc)_2$ (90 mg, 0.40 mmol, 0.1 equiv) in this order. The reaction vessel was covered with a suba seal and purged with argon, then $PhCF_3$ (13.4 mL) was added by syringe. The flask was placed in a preheated oil bath and stirred at 45 °C for 24 h. The mixture was then allowed to cool to rt and diluted with EtOAc (5 mL) before filtering through a pad of Celite, eluting with further EtOAc (2 × 10 mL). The solvent was removed under reduced pressure and the crude material was purified by flash column chromatography (20% EtOAc/pentane) to afford **17a** as an off-white solid (906 mg, 2.29 mmol, 57%).

R_f 0.26 (20% EtOAc/hexane);

mp = 141–142 °C (from acetone/pentane);

IR (film)/cm^{-1} 3345 (NH), 2933, 1676 (C=O), 1522, 1483, 1374, 1245, 1105, 966, 827;

^{1}H NMR (400 MHz, $CDCl_3$) δ 9.22 (s, 1 H, NH), 8.66–8.58 (m, 2 H, 2 × HC_{Ar}), 8.08 (dd, *J* = 8.3, 1.7 Hz, 1 H, HC_{Ar}), 7.52–7.41 (m, 2 H, 2 × HC_{Ar}), 7.37 (dd, *J* = 8.3, 4.2 Hz, 1 H, HC_{Ar}), 7.23–7.15 (m, 2 H, 2 × HC_{Ar}), 6.73–6.65 (m, 2 H, 2 × HC_{Ar}), 3.56 (s, 3 H, OCH_3), 3.38–3.28 (m, 1 H, CHAr), 3.16–2.92 (m, 2 H, CH(C=O) and $CF_2C\mathit{H}HCHAr$), 2.70 (dtt, *J* = 33.2, 13.0, 4.2 Hz, 1 H, $CF_2C\mathit{H}HCH_2$), 2.30-2.07 (m, 4 H, $CF_2CH\mathit{H}CH_2$, $CF_2CH_2C\mathit{H}_2$, $CF_2CH\mathit{H}CH$);

^{13}C NMR (101 MHz, $CDCl_3$) δ 172.1 (C=O amide), 158.6 (C_{Ar} quat), 148.0 (C_{Ar}), 138.3 (C_{Ar} quat), 136.2 (C_{Ar}), 134.2 (C_{Ar} quat), 133.5 (C_{Ar} quat), 128.5 (2 × C_{Ar}), 127.9 (C_{Ar} quat), 127.3 (C_{Ar}), 124.5 (dd, J_{CF} = 243.0, 238.1 Hz, CF_2), 121.61 (C_{Ar}), 121.57 (C_{Ar}), 116.4 (C_{Ar}), 114.1 (2 × C_{Ar}), 55.1 (OCH_3), 47.2 (*C*H(C=O)), 42.0 (t, J_{CF} = 10.0 Hz, CHAr), 35.0 (t, J_{CF} = 24.0 Hz, $CF_2\mathit{C}H_2CHAr$), 30.0 (t, J_{CF} = 24.1 Hz, $CF_2\mathit{C}H_2CH_2$), 26.3 (t, J_{CF} = 10.6 Hz, $CF_2CH_2\mathit{C}H_2$);

^{19}F{^{1}H} NMR (377 MHz, $CDCl_3$) δ –88.29 (d, J_{FF} = 235.9 Hz), –100.76 (d, J_{FF} = 236.0 Hz);

HRMS (ESI) m/z Calculated for $C_{23}H_{23}N_2O_2F_2$ [M+H] 397.1728; Found 397.1720.

trans-(±)-4,4-Difluoro-2-(4-methoxyphenyl)-N-(quinolin-8-yl)cyclohexane-1-carboxamide (18a)

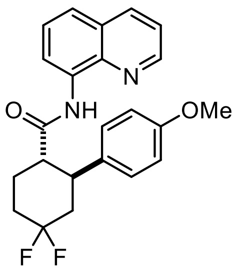

A reaction tube was charged with *cis*-arylated **17a** (435 mg, 1.50 mmol, 1.0 equiv) and NaOH (600 mg, 15.00 mmol, 10.0 equiv). The reaction vessel was sealed and purged with argon, then EtOH (7.5 mL) was added by

syringe. The reaction tube was gradually heated to 100 °C in an oil bath, followed by stirring at 100 °C for 30 min. The mixture was then allowed to cool down to rt and the solvent evaporated. EtOAc (10 mL) and distilled water (10 mL) were added and the organic phase separated and washed with brine (2 × 5 mL). The organic phase was dried over Na_2SO_4, filtered and concentrated under reduced pressure to afford the *trans*-arylated piperidine **18a** as a yellow solid which did not require further purification (360 mg, 0.84 mmol, 84%).

R_f 0.45 (20% acetone/pentane);

mp = 153–154 °C (from acetone/pentane);

IR (film)/cm^{-1} 3347 (NH br), 2937, 1685 (C=O), 1521, 1372, 1252, 1163, 1096, 969, 828;

^{1}H NMR (400 MHz, $CDCl_3$) δ 9.49 (s, 1 H, NH), 8.74 (dd, *J* = 4.2, 1.7 Hz, 1 H, HC_{Ar}), 8.60 (dd, *J* = 5.3, 3.7 Hz, 1 H, HC_{Ar}), 8.11 (dd, *J* = 8.3, 1.7 Hz, 1 H, HC_{Ar}), 7.48–7.37 (m, 3 H, 3 × HC_{Ar}), 7.24–7.13 (m, 2 H, 2 × HC_{Ar}), 6.81–6.67 (m, 2 H, 2 × HC_{Ar}), 3.60 (s, 3 H, OCH_3), 3.34 (ddd, *J* = 13.1, 11.3, 4.0 Hz, 1 H, CHAr), 2.69 (ddd, *J* = 10.6, 7.8, 5.9 Hz, 1 H, CH(C=O)), 2.45–2.31 (m, 2 H, $F_2CC\mathit{H}HCHAr$ and $F_2CC\mathit{H}HCH_2$), 2.28–2.17 (m, 2 H, $F_2CCH\mathit{H}CHAr$ and $F_2CCH\mathit{H}CH_2$), 2.17–1.86 (m, 2 H, $F_2CCH_2C\mathit{H_2}$);

^{13}C NMR (101 MHz, $CDCl_3$) δ 171.7 (C=O amide), 158.4 (C_{Ar} quat), 147.9 (C_{Ar}), 138.1 (C_{Ar} quat), 136.2 (C_{Ar}), 134.0 (C_{Ar} quat), 133.6 (C_{Ar} quat), 128.1 (2 × C_{Ar}), 127.7 (C_{Ar} quat), 127.2 (C_{Ar}), 122.6 (dd, J_{CF} = 243.8, 240.8 Hz, CF_2), 121.5 (C_{Ar}), 116.4 (C_{Ar}), 114.1 (2 × C_{Ar}), 55.0 (OCH_3), 52.3 (*C*H(C=O)), 42.7 (d, J_{CF} = 10.0 Hz, CHAr), 40.8 (t, J_{CF} = 24.0 Hz, $F_2C\mathit{C}H_2CHAr$), 32.9 (t, J_{CF} = 24.1 Hz, $F_2C\mathit{C}H_2CH_2$), 26.7 (d, J_{CF} = 10.6 Hz, $F_2CCH_2\mathit{C}H_2$);

^{19}F{^{1}H} NMR (376 MHz, $CDCl_3$) δ –90.49 (d, J_{FF} = 238.6 Hz), –101.24 (d, J_{FF} = 238.7 Hz);

HRMS (ESI) *m/z* Calculated for $C_{23}H_{23}N_2O_2F_2$ [M+H] 397.1728; Found 397.1721.

4,3-Disubstitued Fragment Synthesis

cis-(±)-1-(*tert*-Butoxycarbonyl)-3-(4-methoxyphenyl)piperidine-4-carboxylic acid (45a)

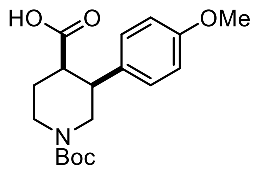

According to the procedure reported by Bull,[25] a flame-dried microwave vial (25 mL) was charged with *cis*-arylated piperidine **12a** (460 mg, 1.00 mmol, 1.00 equiv), di-*tert*-butyl dicarbonate (Boc_2O, 1.10 g, 5.00 mmol, 5.0 equiv) and 4-(dimethylamino)pyridine (DMAP, 25 mg, 0.20 mmol, 0.2 equiv). The reaction vessel was sealed and purged with argon, then MeCN (2 mL, 0.5 M) was added by syringe. The vial was placed in an oil bath and gradually warmed up to 70 °C, then stirred for 3 h at 70 °C. The mixture was allowed to cool down and sat. aq. NH_4Cl (10 mL) and CH_2Cl_2 (15 mL) were added. The phases were separated, and the aqueous layer was extracted with CH_2Cl_2 (3 × 10 mL). The combined organic extracts were dried over Na_2SO_4 and filtered and the solvent was removed under reduced pressure to afford the crude *N*-Boc protected amide derivative, which was carried forward to the hydrolysis step without further purification. A solution of H_2O_2 (30 wt.% in H_2O, 0.40 mL, 5.0 equiv) in THF (1.1 mL, 0.1 M) was added to a solution of $LiOH{\cdot}H_2O$ (168 mg, 4 mmol, 4 equiv) in H_2O (1.6 mL, 0.1 M) at 0 °C under argon. The resulting mixture was added dropwise to a solution of the crude *N*-Boc protected amide in THF (2 mL, 0.5 M) at 0 °C under argon. The reaction was then stirred at 45 °C for 2 h. The reaction mixture was then allowed to cool down to rt and sat. aq. $Na_2S_2O_3$ (10 mL), EtOAc (10 mL) and 1 M NaOH (5 mL) were added. The aqueous alkaline layer was collected, and the organic layer extracted with 1 M NaOH (3 × 15 mL). All the aqueous alkaline layers were combined and acidified to pH ~ 1 with 1 M HCl, then extracted with EtOAc (3 × 50 mL). The combined organic extracts were dried over Na_2SO_4, filtered, and the solvent evaporated under reduced pressure to afford the desired acid **45a** as a white solid (255 mg, 0.71 mmol, 71% over 2 steps).

R_f 0.27 (25% acetone/pentane);

mp = 140–142 °C (from EtOAc);

IR (film)/cm^{-1} 3206 (OH br), 2971, 2933, 1733 (C=O), 1651 (C=O), 1513, 1431, 1260, 1152, 1118, 1025, 820, 757;

^{1}H NMR (500 MHz, DMSO-*d*$_6$, 373 K) δ 7.18 (dd, J = 8.9, 0.6 Hz, 2 H, 2 × HCAr), 6.82 (d, J = 8.8 Hz, 2 H, 2 × HCAr), 3.93 (ddd, J = 13.1, 7.2, 0.8 Hz, 1 H, NC*H*HCHAr), 3.74 (s, 3 H, OCH_3), 3.66 (ddd, J = 13.2, 7.6, 4.0 Hz, 1 H, NC*H*HCH_2), 3.58 (dd, J = 13.1, 4.1 Hz, 1 H, NCH*H*CHAr), 3.37 (ddd, J = 13.2, 7.6, 4.2 Hz, 1 H, NCH*H*CH_2), 3.09 (dt, J = 7.2, 4.4 Hz, 1 H, CHAr), 2.90 (dt, J = 7.5, 4.8 Hz, 1 H, CH(C=O)), 1.87 (dtd, J = 13.9, 7.5, 4.0 Hz, 1 H, NCH_2C*H*H), 1.77 (ddt, J = 13.9, 7.6, 4.4 Hz, 1 H, NCH_2CH*H*), 1.39 (s, 9 H, $C(CH_3)_3$);

^{13}C NMR (126 MHz, DMSO-*d*$_6$, 373 K) δ 173.6 (C=O acid), 157.6 (C_{Ar} quat), 153.6 (C=O carbamate), 132.8 (C_{Ar} quat), 128.5 (2 × C_{Ar}), 113.1 (2 × C_{Ar}), 78.2 (*C*$(CH_3)_3$), 54.6 (OCH_3), 45.8 (N*C*H_2CHAr), 43.5 (*C*H(C=O)), 40.6 (N*C*H_2CH_2), 40.1 (CHAr), 27.6 (C(*C*$H_3)_3$), 24.7 (NCH_2*C*H_2);

HRMS (ESI) m/z Calculated for $C_{18}H_{26}NO_5$ [M+H] 336.1811; Found 336.1822.

Characterisation information consistent with data previously reported in literature.[25]

trans-(±)-1-(_tert_-Butoxycarbonyl)-3-(4-methoxyphenyl)piperidine-4-carboxylic acid (46a)

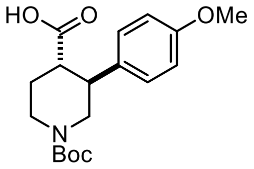

A flame-dried microwave vial (25 mL) was charged with *trans*-arylated piperidine **13a** (369 mg, 0.80 mmol, 1.00 equiv), di-*tert*-butyl dicarbonate (Boc_2O, 0.88 g, 4.00 mmol, 5.0 equiv) and 4-(dimethylamino)pyridine (DMAP, 20 mg, 0.16 mmol, 0.2 equiv). The reaction vessel was sealed and purged with argon, then MeCN (1.6 mL, 0.5 M) was added by syringe. The vial was placed in an oil bath and gradually warmed up to 70 °C, then stirred for 3 h at 70 °C. The mixture was allowed to cool down and sat. aq. NH_4Cl (10 mL) and CH_2Cl_2 (15 mL) were added. The phases were separated, and the aqueous layer was extracted with CH_2Cl_2 (3 × 10 mL). The combined organic extracts were dried over Na_2SO_4 and filtered and the solvent was removed under reduced pressure to afford the crude *N*-Boc protected amide derivative, which was carried forward to the hydrolysis step without further purification. A solution of H_2O_2 (30 wt.% in H_2O, 0.32 mL, 5.0 equiv) in THF (0.9 mL, 0.1 M) was added to a solution of LiOH·H_2O (134 mg, 3.2 mmol, 4 equiv) in H_2O (1.3 mL, 0.1 M) at 0 °C under argon. The resulting mixture was added dropwise to a solution of the crude *N*-Boc protected amide in THF (1.6 mL, 0.5 M) at 0 °C under argon. The reaction was then stirred at 45 °C for 2 h. The reaction mixture was then allowed to cool down to rt and sat. aq. $Na_2S_2O_3$ (10 mL), EtOAc (10 mL) and 1 M NaOH (5 mL) were added. The aqueous alkaline layer was collected, and the organic layer extracted with 1 M NaOH (3 × 15 mL). All the aqueous alkaline layers were combined and acidified to pH ~ 1 with 1 M HCl, then extracted with EtOAc (3 × 50 mL). The combined organic extracts were dried over Na_2SO_4, filtered, and the solvent evaporated under reduced pressure to afford the desired acid **46a** as a white solid (161 mg, 0.48 mmol, 60% over 2 steps).

R_f 0.25 (20% acetone/pentane);

mp = 173–174 °C (from Et_2O/pentane);

IR (film)/cm^{1} 2999, 2972, 2928, 2859, 1730 (C=O), 1689 (C=O), 1512, 1423, 1293, 1244, 1158, 910, 828, 732;

^{1}H NMR (400 MHz, DMSO-*d*$_6$, 373 K) δ 7.17 (d, *J* = 8.7 Hz, 2 H, 2 × HC_{Ar}), 6.86 (d, *J* = 8.8 Hz, 2 H, 2 × HC_{Ar}), 4.05 (ddd, *J* = 13.3, 4.4, 2.6 Hz, 1 H, NC*H*HCH$_2$), 3.91 (dt, *J* = 10.8, 1.6 Hz, 1 H, NC*H*HCHAr), 3.75 (s, 3 H, OCH_3), 2.87 (td, J = 13.0, 2.9 Hz, 1 H, NCH*H*CH$_2$), 2.84–2.70 (m, 3 H, NCH*H*CHAr, CH(C=O) and CHAr), 1.93 (dq, J = 13.3, 3.0 Hz, 1 H, NCH$_2$C*H*H), 1.62–1.51 (m, 1 H, NCH$_2$CH*H*), 1.43 (s, 9 H, C(CH_3)$_3$);

^{13}C NMR (101 MHz, DMSO*d*$_6$, 373 K) δ 174.0 (C=O acid), 157.8 (C_{Ar} quat), 153.4 (C=O carbamate), 132.8 (C_{Ar} quat), 128.0 (2 × C_{Ar}), 113.5 (2 × C_{Ar}), 78.4 (*C*(CH_3)$_3$), 54.6 (OCH_3), 49.0 (N*C*H$_2$CHAr), 46.8 (*C*H(C=O)), 43.2 (N*C*H$_2$CH$_2$), 42.4 (*C*HAr), 28.2 (NCH$_2$*C*H$_2$), 27.6 (C(*C*H$_3$)$_3$);

HRMS (ESI) *m/z* Calculated for $C_{18}H_{26}NO_5$ [M+H] 336.1811; Found 336.1820.

cis-(±)-1-(tert-Butoxycarbonyl)-3-(4-fluorophenyl)piperidine-4-carboxylic acid (45b)

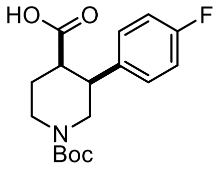

A flame-dried microwave vial (25 mL) was charged with *trans*-arylated piperidine **12b** (0.43 g, 0.96 mmol, 1.00 equiv), di*tert*-butyl dicarbonate (Boc_2O, 1.04 g, 4.80 mmol, 5.0 equiv) and 4-(dimethylamino)pyridine (DMAP, 20 mg, 0.19 mmol, 0.2 equiv). The reaction vessel was sealed and purged with argon, then MeCN (1.6 mL, 0.5 M) was added by syringe. The vial was placed in an oil bath and gradually warmed up to 70 °C, then stirred for 3 h at 70 °C. The mixture was allowed to cool down and sat. aq. NH_4Cl (10 mL) and CH_2Cl_2 (15 mL) were added. The phases were separated, and the aqueous layer was extracted with CH_2Cl_2 (3 × 10 mL). The combined organic extracts were dried over Na_2SO_4 and filtered and the solvent was removed under reduced pressure to afford the crude *N*-Boc protected amide derivative, which was carried forward to the hydrolysis step without further purification. A solution of H_2O_2 (30 wt.% in H_2O, 0.32 mL, 5.0 equiv) in THF (0.9 mL, 0.1 M) was added to a solution of $LiOH \cdot H_2O$ (157 mg, 3.8 mmol, 4 equiv) in H_2O (1.3 mL, 0.1 M) at 0 °C under argon. The resulting mixture was added dropwise to a solution of the crude *N*-Boc protected amide in THF (1.6 mL, 0.5 M) at 0 °C under argon. The reaction was then stirred at 45 °C for 2 h. The reaction mixture was then allowed to cool down to rt and sat. aq. $Na_2S_2O_3$ (10 mL), EtOAc (10 mL) and 1 M NaOH (5 mL) were added. The aqueous alkaline layer was collected, and the organic layer extracted with 1 M NaOH (3 × 15 mL). All the aqueous alkaline layers were combined and acidified to pH ~ 1 with 1 M HCl, then extracted with EtOAc (3 × 50 mL). The combined organic extracts were dried over Na_2SO_4, filtered, and the solvent evaporated under reduced pressure to afford the desired acid **45b** as a white solid (186 mg, 60% over 2 steps).

R_f 0.15 (20% acetone/pentane);

mp = 161–164 °C (from Et_2O/pentane);

IR (film)/cm^{-1} 2973 (OH br), 1729 (C=O), 1692 (C=O), 1647, 1510, 1429, 1366, 1301, 1228, 1160, 1112, 1008, 908, 825, 769;

^{1}H NMR (400 MHz, DMSO-d_6, 373 K) δ 7.33–7.26 (m, 2 H, HC_{Ar}), 7.10–7.00 (m, 2 H, HC_{Ar}), 3.94 (ddd, *J* = 13.2, 7.1, 0.8 Hz, 1 H, NC*H*HCH), 3.67 (ddd, *J* = 13.5, 7.4, 4.1 Hz, 1 H, NC*H*HCH_2), 3.59 (dd, *J* = 13.1, 4.1 Hz, 1 H, NCH*H*CH), 3.37 (ddd, *J* = 13.3, 7.6, 4.2 Hz, 1 H, NCH*H*CH_2), 3.17 (dt, *J* = 7.1, 4.4 Hz, 1 H, CHAr), 2.95 (dt, *J* = 7.6, 4.8 Hz, 1 H, CH(C=O)), 1.89 (dtd, *J* = 14.0, 7.6, 4.1 Hz, 1 H, NCH_2C*H*H), 1.79 (ddt, *J* = 14.0, 7.5, 4.5 Hz, 1 H, NCH_2CH*H*), 1.38 (s, 9 H, C(CH_3)$_3$).

^{13}C NMR (101 MHz, DMSO-d_6, 373 K) δ 173.4 (COOH), 160.5 (d, *J* = 242.7 Hz, FC_{Ar} quat), 153.6 (C=O carbamate), 137.0 (d, *J* = 3.1 Hz, F-*p*-C_{Ar} quat), 129.3 (d, *J* = 7.8 Hz, 2 × F-*m*-C_{Ar}), 114.0 (d, *J* = 21.1 Hz, 2 × F-*o*-C_{Ar}), 78.3 (*C*(CH_3)$_3$), 45.7 (N*C*H_2CH), 43.3 (*C*HCOOH), 40.6 (N*C*H_2CH_2), 40.1 (*C*HAr), 27.6 (C(*C*H_3)$_3$), 24.6 (NCH_2*C*H_2).

^{19}F NMR (376 MHz, DMSO-d_6, 373 K) δ -116.85 (dd, *J* = 9.6, 5.0 Hz).

HRMS (ESI$^+$) *m/z* Calculated for $C_{17}H_{21}NO_4F$ [M-H] 322.1455; Found 322.1460.

trans-(±)-1-(tert-Butoxycarbonyl)-3-(4-fluorophenyl)piperidine-4-carboxylic acid (46b)

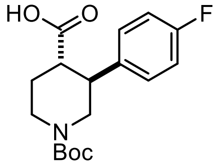

A flame-dried microwave vial (25 mL) was charged with *trans*-arylated piperidine **13b** (396 mg, 1.00 mmol, 1.00 equiv), di*tert*-butyl dicarbonate (Boc_2O, 1.10 g, 5.00 mmol, 5.0 equiv) and 4-(dimethylamino)pyridine (DMAP, 25 mg, 0.20 mmol, 0.2 equiv). The reaction vessel was sealed and purged with argon, then MeCN (2 mL, 0.5 M) was added by syringe. The vial was placed in an oil bath and gradually warmed up to 70 °C, then stirred for 3 h at 70 °C. The mixture was allowed to cool down and sat. aq. NH_4Cl (10 mL) and CH_2Cl_2 (15 mL) were added. The phases were separated, and the aqueous layer was extracted with CH_2Cl_2 (3 × 10 mL). The combined organic extracts were dried over Na_2SO_4 and filtered and the solvent was removed under reduced pressure to afford the crude *N*-Boc protected amide derivative, which was carried forward to the hydrolysis step without further purification. A solution of H_2O_2 (30 wt.% in H_2O, 0.40 mL, 5.0 equiv) in THF (1.1 mL, 0.1 M) was added to a solution of $LiOH{\cdot}H_2O$ (168 mg, 4 mmol, 4 equiv) in H_2O (1.6 mL, 0.1 M) at 0 °C under argon. The resulting mixture was added dropwise to a solution of the crude *N*-Boc protected amide in THF (2 mL, 0.5 M) at 0 °C under argon. The reaction was then stirred at 45 °C for 2 h. The reaction mixture was then allowed to cool down to rt and sat. aq. $Na_2S_2O_3$ (10 mL), EtOAc (10 mL) and 1 M NaOH (5 mL) were added. The aqueous alkaline layer was collected, and the organic layer extracted with 1 M NaOH (3 × 15 mL). All the aqueous alkaline layers were combined and acidified to pH ~ 1 with 1 M HCl, then extracted with EtOAc (3 × 50 mL). The combined organic extracts were dried over Na_2SO_4, filtered, and the solvent evaporated under reduced pressure to afford the desired acid **46b** as a white solid (257 mg, 0.79 mmol, 79% over 2 steps).

R_f 0.31 (20% acetone/pentane);

mp = 148–150 °C (from Et_2O/pentane);

IR (film)/cm^{-1} 2982, 1685 (C=O), 1513, 1424, 1297, 1230, 1163, 910, 835, 731;

^1H NMR (400 MHz, DMSO-d_6, 373 K) δ 7.34–7.22 (m, 2 H, 2 × HC_{Ar}), 7.09 (t, *J* = 9.0 Hz, 2 H, 2 × HC_{Ar}), 4.05 (dddd, *J* = 13.3, 4.4, 2.7, 1.7 Hz, 1 H, NC*H*HCH$_2$), 3.92 (dq, *J* = 9.3, 1.9 Hz, 1 H, NC*H*HCHAr), 2.90 (td, *J* = 13.0, 3.0 Hz, 1 H, NCH*H*CH$_2$), 2.84–2.71 (m, 3 H, NCH*H*CHAr, CH(C=O), CHAr), 1.95 (dq, J = 13.2, 2.9 Hz, 1 H, NCH_2C*H*H), 1.64–1.51 (m, 1 H, NCH_2CH*H*), 1.43 (s, 9 H, $C(CH_3)_3$);

^{13}C NMR (101 MHz, DMSO-d_6, 373 K) δ 173.9 (C=O acid), 160.7 (d, J_{CF} = 241.7 Hz, FC_{Ar} quat), 153.4 (C=O carbamate), 136.9 (d, J_{CF} = 3.2 Hz, F-*p*-C_{Ar} quat), 128.8 (d, J_{CF} = 8.9 Hz, 2 × F-*m*-C_{Ar}), 114.4 (d, J_{CF} = 22.6 Hz, 2 × F-*o*-C_{Ar}), 78.5 (*C*$(CH_3)_3$), 48.7 (N*C*H_2CHAr), 46.7 (*C*H(C=O)), 43.3 (CHAr), 42.4 (N*C*H_2CH_2), 28.1 (NCH_2*C*H_2), 27.6 (C(*C*$H_3)_3$);

$^{19}F\{^1H\}$ NMR (376 MHz, DMSO-d_6, 373 K) δ –116.25 (s);

HRMS (ESI) *m/z* Calculated for $C_{17}H_{21}NO_4F$ [M+H] 322.1455; Found 322.1462.

tert-Butyl-cis-(±)-4-carbamoyl-3-(4-methoxyphenyl)piperidine-1-carboxylate (49a)

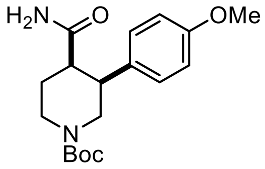

According to the procedure reported by Bull,[25] by adapting a procedure reported by O'Brien,[15a] a flame-dried microwave vial (25 mL) was charged with *cis*-arylated piperidine carboxylic acid **45a** (260 mg, 0.78 mmol, 1.0 equiv), freshly distilled Et_3N (0.98 mL, 0.78 mmol, 1.0 equiv) and THF (7.8 mL). The reaction vessel was sealed and purged with argon, then cooled to –20 °C before isobutyl chloroformate (127 mg, 0.93 mmol, 1.2 equiv) was added dropwise. The resulting solution was stirred at –20 °C for 40 min, then 35% aq. NH_4OH (7.8 mL) was added and the mixture allowed to warm to rt over 30 min. The solvent was evaporated under reduced pressure and the residue dissolved in EtOAc (15 mL), washed with sat. aq. $NaHCO_3$ (2 × 10 mL), then brine (15 mL) and dried over Na_2SO_4. The organic layer was evaporated under reduced pressure to give the crude product, which was purified by flash column chromatography (78:20:2 pentane/acetone/Et_3N to 63:35:2 pentane/acetone/Et_3N). The product containing fractions were combined and concentrated under reduced pressure. To remove any residual solvent, Et_2O (10 mL) and pentane (10 mL) were added and the mixture was evaporated again to afford amide **49a** as a white solid (192 mg, 0.57 mmol, 73% over 2 steps).

R_f 0.19 (20% acetone/pentane);

mp = 177–179 °C (from Et_2O/pentane);

IR (film)/cm^{1} 3459 (NH), 3326 (NH br), 2999, 2972, 2929, 1663 (C=O), 1610 (C=O), 1511, 1463, 1425, 1247, 1163, 1119, 1032, 909, 830, 732;

^{1}H NMR (500 MHz, DMSO-d_6, 373 K) δ 7.18 (d, *J* = 8.7 Hz, 2 H, 2 × HC_{Ar}), 6.81 (d, *J* = 8.7 Hz, 2 H, 2 × HC_{Ar}), 6.49 (br s, 2 H, NH_2), 3.99 (dd, *J* = 12.8, 8.6 Hz, 1 H, NC*H*HCHAr), 3.74 (s, 3 H, OCH_3), 3.64 (ddd, *J* = 12.9, 8.9, 3.8 Hz, 1 H, NC*H*HCH_2), 3.59 (dd, *J* = 12.7, 4.3 Hz, 1 H, NCH*H*CHAr), 3.46 (ddd, *J* = 12.9, 6.1, 4.3 Hz, 1 H, NCH*H*CH_2), 2.99 (dt, *J* = 8.7, 4.4 Hz, 1 H, CHAr), 2.76 (dt, *J* = 6.2, 4.8 Hz, 1 H, CH(C=O)), 1.82 (dtd, *J* = 13.7, 6.2, 3.8 Hz, 1 H, NCH_2C*H*H), 1.71 (ddt, *J* = 13.6, 9.1, 4.6 Hz, 1 H, NCH_2CH*H*), 1.40 (s, 9 H, C(CH_3)$_3$);

^{13}C NMR (126 MHz, DMSO-d_6, 373 K) δ 174.4 (C=O amide), 157.5 (C_{Ar} quat), 153.7 (C=O carbamate), 133.2 (C_{Ar} quat), 128.5 (2 × C_{Ar}), 113.1 (2 × C_{Ar}), 78.0 (*C*(CH_3)$_3$), 54.6 (OCH_3), 45.5 (NCH_2CHAr), 43.1 (CH(C=O)), 41.2 (CHAr), 40.3 (NCH_2CH_2), 27.7 (C(CH_3)$_3$), 26.0 (NCH_2*C*H_2);

HRMS (ESI) *m/z* Calculated for $C_{18}H_{27}N_2O_4$ [M+H] 335.1971; Found 335.1977.

Characterisation information consistent with data previously reported in literature.[25]

tert-Butyl-trans-(±)-4-carbamoyl-3-(4-methoxyphenyl)piperidine-1-carboxylate (50a)

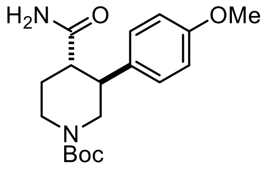

Adapting a procedure reported by O'Brien, [15a] a flame-dried microwave vial (25 mL) was charged with *trans*-arylated piperidine carboxylic acid **46a** (228 mg, 0.70 mmol, 1.0 equiv), freshly distilled Et_3N (0.91 mL, 0.70

mmol, 1.0 equiv) and THF (7.0 mL). The reaction vessel was sealed and purged with argon, then cooled to –20 °C before isobutyl chloroformate (112 mg, 0.84 mmol, 1.2 equiv) was added dropwise. The resulting solution was stirred at –20 °C for 40 min, then 35% aq. NH_4OH (7.0 mL) was added and the mixture allowed to warm to rt over 30 min. The solvent was evaporated under reduced pressure and the residue dissolved in EtOAc (15 mL), washed with sat. aq. $NaHCO_3$ (2 × 10 mL), then brine (15 mL) and dried over Na_2SO_4. The organic layer was evaporated under reduced pressure to give the crude product, which was purified by flash column chromatography (78:20:2 pentane/acetone/Et_3N to 63:35:2 pentane/acetone/Et_3N). The product containing fractions were combined and concentrated under reduced pressure. To remove any residual solvent, Et_2O (10 mL) and pentane (10 mL) were added and the mixture was evaporated again to afford amide **50a** as a white solid (175 mg, 0.51 mmol, 74% over 2 steps).

R_f 0.15 (20% acetone/pentane);

mp = 177–179 °C (from Et_2O/pentane);

IR (film)/cm^1 3325 (NH br), 3191, 2974, 2855, 1662 (C=O), 1513, 1416, 1238, 1163, 1036, 910, 828, 731;

^{1}H NMR (400 MHz, DMSO-d_6, 373 K) δ 7.16 (dd, J = 8.2, 1.5 Hz, 2 H, 2 × HC_{Ar}), 6.91–6.80 (m, 2 H, 2 × HC_{Ar}), 4.06 (dt, J = 13.1, 3.7 Hz, 1 H, NC*H*HCH$_2$), 3.99–3.87 (m, 1 H, NC*H*HCHAr), 3.74 (s, 3 H. OCH_3), 2.90–2.69 (m, 3 H, NCH*H*CH$_2$, NCH*H*CHAr and CHAr), 2.62 (td, J = 11.1, 10.7, 3.8 Hz, 1 H, CH(C=O)), 1.82 (dd, J = 13.1, 3.3 Hz, 1 H, NCH$_2$C*H*H), 1.57 (qd, J = 12.6, 4.6 Hz, 1 H, NCH$_2$CH*H*), 1.43 (s, 9 H, C(C*H$_3$*)$_3$);

^{13}C NMR (101 MHz, DMSO-d_6, 373 K) δ 174.3 (C=O amide), 157.7 (C_{Ar} quat), 153.4 (C=O carbamate), 133.1 (C_{Ar} quat), 128.0 (2 × C_{Ar}), 113.4 (2 × C_{Ar}), 78.2 (*C*(CH$_3$)$_3$), 54.6 (OCH_3), 49.3 (N*C*H$_2$CHAr), 47.4 (*C*H(C=O)), 42.8 (CHAr), 42.7 (N*C*H$_2$CH$_2$), 28.7 (NCH$_2$*C*H$_2$), 27.6 (C(*C*H$_3$)$_3$);

HRMS (ESI) *m/z* Calculated for $C_{18}H_{27}N_2O_4$ [M+H] 335.1971; Found 335.1979.

tert-Butyl-cis-(±)-4-carbamoyl-3-(4-fluorophenyl)piperidine-1-carboxylate (49b)

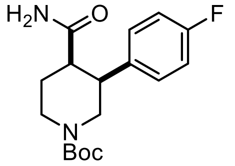

Adapting a previously reported protocol by Chen,[28a] a flask was charged with *trans*-arylated piperidine **12b** (1.30 g, 2.89 mmol, 1.0 equiv) and stabilised 2-iodoxybenzoic acid (IBX, 30 wt.%, 1.62 g, 5.78 mmol, 2.0 equiv) under air. H_2O (3.0 mL, 0.3 M) and 1,1,1,3,3,3-hexafluoro-2-propanol (HFIP, 3.0 mL, 0.3 M) were added, and the resulting solution (6.0 mL, 0.15 M) was stirred at 60 °C for 1.5 h. The reaction mixture was then allowed to cool to rt and sat. aq. $NaHCO_3$ (10 mL) and CH_2Cl_2 (10 ml) were added. The phases were separated, and the aqueous layer was extracted with CH_2Cl_2 (3 × 15 mL). The combined organic extracts were dried over Na_2SO_4, filtered and concentrated under reduced pressure. The crude material was purified by flash column chromatography (78:20:2 pentane:acetone:Et_3N). Et_2O (5 mL) and pentane (5 mL) were added, and the mixture was evaporated again to afford the desired free amide **49b** as a white solid (570 mg, 1.76 mmol, 61%).

R_f = 0.10 (20% acetone/pentane).

mp = 139–142 °C (from Et_2O/pentane);

IR (film)/cm^{-1} 3329 (NH br), 3190 (NH br), 2973, 1659 (C=O), 1509, 1423, 1365, 1282, 1226, 12159, 1007, 905, 835, 755;

^{1}H NMR (400 MHz, DMSO-d_6, 373 K) δ 7.34–7.25 (m, 2 H, HC_{Ar}), 7.09–6.98 (m, 2 H, HC_{Ar}), 4.02 (dd, *J* = 12.8, 8.4 Hz, 1 H, NC*H*HCHAr), 3.71–3.56 (m, 2 H, NC*H*HCH$_2$ and NCH*H*CHAr), 3.51–3.41 (m, 1 H, NHCH*H*CH$_2$), 3.07 (dt, *J* = 8.7, 4.5 Hz, 1 H, CHAr), 2.80 (dt, *J* = 6.3, 4.8 Hz, 1 H, CH(C=O)), 1.83 (dtd, *J* = 13.8, 6.3, 3.9 Hz, 1 H, NCH$_2$C*H*H), 1.78–1.67 (m, 1 H, NCH$_2$CH*H*), 1.39 (s, 9 H, C(CH$_3$)$_3$);

^{13}C NMR (101 MHz, DMSO-d_6, 373 K) δ 174.3 (C=O amide), 160.5 (d, J_{CF} = 242.5 Hz, FC_{Ar} quat), 153.7 (C=O carbamate), 137.2 (d, J_{CF} = 3.3 Hz, F-*p*-C_{Ar} quat), 129.3 (d, J_{CF} = 7.8 Hz, 2 × F-*m*-C_{Ar}), 113.9 (d, J_{CF} = 20.8 Hz, 2 × F-*o*-C_{Ar}), 78.1 (*C*(CH$_3$)$_3$), 45.3 (N*C*H$_2$CH), 43.0 (*C*HCO), 41.1 (CHAr), 40.3 (N*C*H$_2$CH$_2$), 27.7 (C(*C*H$_3$)$_3$), 25.9 (NCH$_2$*C*H$_2$);

^{19}F NMR (376 MHz, DMSO-d_6, 373 K) δ -117.00–-117.12 (m);

HRMS (ESI) m/z Calculated for $C_{17}H_{24}N_2O_3F$ [M+H] 323.1771; Found 323.1784.

tert-Butyl-_trans_-(±)-4-carbamoyl-3-(4-fluorophenyl)piperidine-1-carboxylate (50b)

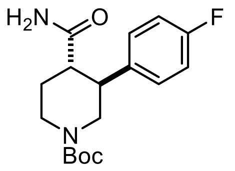

Adapting a previously reported protocol by Chen,[28a] a flask was charged with *trans*-arylated piperidine **13b** (400 mg, 0.90 mmol, 1.0 equiv) and stabilised 2-iodoxybenzoic acid (IBX, 30 wt.%, 513 mg, 1.80 mmol, 2.0 equiv) under air. H_2O (3.0 mL, 0.3 M) and 1,1,1,3,3,3-hexafluoro-2-propanol (HFIP, 3.0 mL, 0.3 M) were added, and the resulting solution (6.0 mL, 0.15 M) was stirred at 60 °C for 1.5 h. The reaction mixture was then allowed to cool to rt and sat. aq. $NaHCO_3$ (10 mL) and CH_2Cl_2 (10 ml) were added. The phases were separated, and the aqueous layer was extracted with CH_2Cl_2 (3 × 15 mL). The combined organic extracts were dried over Na_2SO_4, filtered and concentrated under reduced pressure. The crude material was purified by flash column chromatography (78:20:2 pentane:acetone:Et_3N). Et_2O (5 mL) and pentane (5 mL) were added, and the mixture was evaporated again to afford the desired free amide **50b** as a pale orange solid (195 mg, 0.61 mmol, 68%).

R_f = 0.12 (40% acetone/pentane)

mp = 204–206 °C (from Et_2O/pentane);

IR (film)/cm^{-1} 3398, 3326 (NH br), 2974, 2924, 1661 (C=O), 1510, 1418, 1366, 1229, 1161, 909, 830, 732;

^{1}H NMR (400 MHz, DMSO-d_6, 373 K) δ 7.27 (dd, *J* = 8.7, 5.5 Hz, 2 H, 2 × HC_{Ar}), 7.06 (t, *J* = 8.9 Hz, 2 H, 2 × HC_{Ar}), 4.12–4.02 (m, 1 H, NC*H*HCH$_2$), 3.93 (ddd, *J* = 12.7, 3.8, 1.5 Hz, 1 H, NC*H*HCHAr), 2.88–2.80 (m, 2 H, NHCH*H*CH$_2$ and NCH*H*CHAr), 2.81–2.73 (m, 1 H, CHAr), 2.64 (td, *J* = 11.2, 3.9 Hz, 1 H, CH(C=O)), 1.83 (ddt, *J* = 13.2, 3.9, 2.8 Hz, 1 H, NCH$_2$C*H*H), 1.57 (tdd, *J* = 13.0, 11.7, 4.5 Hz, 1 H, NCH$_2$CH*H*), 1.43 (s, 9 H, C(CH$_3$)$_3$);

^{13}C NMR (101 MHz, DMSO-d_6, 373 K) δ 174.3 (C=O amide), 160.7 (d, J_{CF} = 243.6 Hz, FC_{Ar} quat), 153.5 (C=O carbamate), 137.2 (d, J_{CF} = 3.2 Hz, F-*p*-C_{Ar} quat), 129.0 (d, J_{CF} = 8.2 Hz, 2 × F-*m*-C_{Ar}), 114.3 (d, J_{CF} = 22.6 Hz, 2 × F-*o*-C_{Ar}), 78.5 (*C*(CH$_3$)$_3$), 49.1 (N*C*H$_2$CHAr), 47.4 (*C*H(C=O)), 43.0 (CHAr), 42.7 (N*C*H$_2$CH$_2$), 28.7 (NCH$_2$*C*H$_2$), 27.7 (C(*C*H$_3$)$_3$);

$^{19}F\{^1H\}$ NMR (376 MHz, DMSO-d_6, 373 K) δ –116.58 (s);

HRMS (ESI) m/z Calculated for $C_{17}H_{24}N_2O_3F$ [M+H] 323.1771; Found 323.1764.

tert-Butyl-cis-(±)-4-carbamoyl-3-(4-methoxyphenyl)piperidine-1-carboxylate (51a)

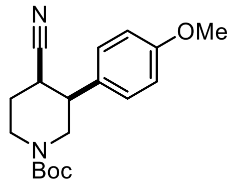

Prepared as previously reported [25] by adapting the procedure reported by Naka,[35] a flame-dried reaction tube was charged with the corresponding *cis* amide **49a** (172 mg, 0.51 mmol, 1.00 equiv), $Pd(OAc)_2$ (11.2 mg, 0.05 mmol, 0.1 equiv), dichloroacetonitrile (0.41 mL, 5.10 mmol, 10.0 equiv), dry MeCN (0.5 mL, 1 M) and H_2O (0.5 mL, 1 M). The reaction vessel was sealed and purged with argon. The reaction tube was then placed in a preheated oil bath and stirred at 60 ºC for 18 h. The reaction mixture was allowed to cool to rt and MeOH (2 mL) was added. The resulting mixture was filtered through a pad of Celite, eluting with further MeOH (2 × 5 mL). The solvent was removed under reduced pressure, and the crude material was purified by flash column chromatography (40% Et_2O/pentane) to afford *cis* arylated nitrile **51a** as a pale yellow solid (125 mg, 0.39 mmol, 77%).

R_f 0.21 (40% Et_2O/pentane);

mp = 125–127 °C (from Et_2O/pentane);

IR (film)/cm^{-1} 2974, 1692 (C=O), 1513, 1416, 1252, 1163, 1036, 835;

^1H NMR (500 MHz, DMSO-d_6, 373 K) δ 7.29–7.25 (m, 2 H, 2 × HC_{Ar}), 6.93 (d, *J* = 8.7 Hz, 2 H, 2 × HC_{Ar}), 4.05 (ddd, *J* = 13.5, 2.5, 1.4 Hz, 1 H, NC*H*HCHAr), 4.02–3.94 (m, 1 H, NC*H*HCH$_2$), 3.78 (s, 3 H, OCH_3), 3.45 (q, *J* = 4.1 Hz, 1 H, CHCN), 3.26 (dd, *J* = 13.5, 11.3 Hz, 1 H, NCH*H*CHAr), 3.06 (ddd, *J* = 13.8, 11.8, 3.3 Hz, 1 H, NCH*H*CH$_2$), 2.97 (d, *J* = 4.1 Hz, 1 H, CHAr), 2.01–1.92 (m, 1 H, NCH$_2$C*H*H), 1.92–1.84 (m, 1 H, NCH$_2$CH*H*), 1.43 (s, 9 H, C(CH$_3$)$_3$);

^{13}C NMR (126 MHz, DMSO-d_6, 373 K) δ 158.2 (C_{Ar} quat), 153.4 (C=O carbamate), 131.1 (C_{Ar} quat), 127.8 (2 × C_{Ar}), 119.1 (nitrile CN), 113.7 (2 × C_{Ar}), 78.8 (*C*(CH$_3$)$_3$), 54.7 (OCH_3), 44.5 (N*C*H$_2$CHAr), 40.1 (CHAr), 39.7 (N*C*H$_2$CH$_2$), 32.9 (*C*HCN), 27.6 (C(*C*H$_3$)$_3$), 27.3 (NCH$_2$*C*H$_2$);

HRMS (ESI) *m/z* Calculated for $C_{18}H_{25}N_2O_3$ [M+H] 317.1865; Found 317.1850.

Characterisation information consistent with data previously reported in literature.[25]

tert-Butyl-cis-(±)-4-cyano-3-(4-fluorophenyl)piperidine-1-carboxylate (51b)

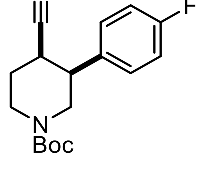

Adapting the procedure reported by Naka,[35] a flame-dried reaction tube was charged with the corresponding *cis* amide **49b** (220 mg, 0.68 mmol, 1.00 equiv), $Pd(OAc)_2$ (15.7 mg, 0.07 mmol, 0.1 equiv), dichloroacetonitrile

(0.41 mL, 5.10 mmol, 10.0 equiv), dry MeCN (0.7 mL, 1 M) and H_2O (0.7 mL, 1 M). The reaction vessel was sealed and purged with argon. The reaction tube was then placed in a preheated oil bath and stirred at 60 ºC for 18 h. The reaction mixture was allowed to cool to rt and MeOH (2 mL) was added. The resulting mixture was filtered through a pad of Celite, eluting with further MeOH (2 × 5 mL). The solvent was removed under reduced pressure, and the crude material was purified by flash column chromatography (20-30% acetone/pentane) to afford *cis* arylated nitrile **51b** as a pale yellow paste (190 mg, 0.62 mmol, 91%).

R_f 0.05 (10% acetone/pentane);

IR (film)/cm^{-1} 2972, 2233 (C=N), 1690 (C=O), 1511, 1418, 1365, 1281, 1160, 1005, 961, 835;

^{1}H NMR (500 MHz, DMSO-*d*$_6$, 373 K) δ 7.44–7.35 (m, 2 H, 2 × HC_{Ar}), 7.21–7.12 (m, 2 H, 2 × HC_{Ar}), 4.06 (ddt, *J* = 13.5, 3.9, 1.4 Hz, 1 H, NC*H*HCHAr), 4.03–3.94 (m, 1 H, NC*H*HCH_2), 3.53–3.46 (m, 1 H, CHAr), 3.30 (dd, *J* = 13.5, 11.2 Hz, 1 H, NCH*H*CHAr), 3.12–3.01 (m, 2 H, NHCH*H*CH_2 and CHCN), 1.97 (dq, *J* = 13.8, 3.4 Hz, 1 H, NCH_2C*H*H), 1.90 (ddt, *J* = 13.8, 11.7, 4.3 Hz, 1 H, NCH_2CH*H*), 1.43 (s, 9 H, $C(CH_3)_3$);

^{13}C NMR (126 MHz, DMSO-*d*$_6$, 373 K) δ 161.0 (d, *J* = 244.1 Hz, FC_{Ar} quat), 153.4 (C=O carbamate), 135.2 (d, *J* = 3.2 Hz, F-*p*-C_{Ar} quat), 128.7 (d, *J* = 8.1 Hz, 2 × F-*m*-C_{Ar}), 118.9 (nitrile CN), 114.6 (d, *J* = 21.3 Hz, 2 × F-*o*-C_{Ar}), 78.8 (*C*$(CH_3)_3$), 44.3 (N*C*H_2CHAr), 40.1 (*C*HCN), 39.7 (N*C*H_2CH_2), 32.7 (CHAr), 27.6 (C(*C*$H_3)_3$), 27.2 (NCH_2*C*H_2);

^{19}F{^{1}H} NMR (471 MHz, DMSO-*d*$_6$, 373 K) δ –115.69 (s);

HRMS (ESI) m/z Calculated for $C_{17}H_{25}N_3O_2F$ [M+NH_4] 322.1931; Found 322.1936.

***tert*-Butyl-*cis*-(±)-4-(hydroxymethyl)-3-(4-methoxyphenyl)piperidine-1-carboxylate (47a)**

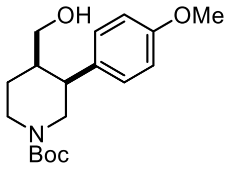

According to the procedure reported by Bull,[25] a flame-dried microwave vial (25 mL) was charged with *cis*-arylated piperidine **12a** (324 mg, 0.70 mmol, 1.0 equiv), di-*tert*-butyl dicarbonate (Boc_2O, 763 mg, 3.50 mmol, 5.0 equiv) and 4-(dimethylamino)pyridine (DMAP, 18 mg, 0.14 mmol, 0.2 equiv). The reaction vessel was sealed and purged with argon, then MeCN (1.4 mL, 0.5 M) was added by syringe. The vial was placed in an oil bath and gradually warmed up to 70 ºC, then stirred for 3 h at 70 °C. The mixture was allowed to cool down and sat. aq. NH_4Cl (10 mL) and CH_2Cl_2 (15 mL) were added. The phases were separated, and the aqueous layer was extracted with CH_2Cl_2 (3 × 10 mL). The combined organic extracts were dried over Na_2SO_4 and filtered. The solvent was removed under reduced pressure to afford the crude *N*-Boc protected amide derivative **43a** which was carried forward to the reductive cleavage step without further purification.

A solution of the crude *N*-Boc-protected amide **43a** in dry THF (3.5 mL, 0.2 M) was added dropwise to a suspension of $LiAlH_4$ (110 mg, 2.80 mmol, 4.0 equiv) in dry THF (1.4 mL, 0.5 M) at 0 °C under argon atmosphere. The mixture was then stirred at 25 °C for 1 h or until TLC showed reaction completion. The reaction mixture was then quenched by slow addition of sat. aq. NH_4Cl (2-3 mL) at 0 °C and stirred at rt for 30 min. The suspension was filtered through a pad of Celite, eluting with EtOAc (3 × 5 mL). The phases were separated and the aqueous layer extracted with EtOAc (3 × 10 mL). The combined organic extracts were dried over Na_2SO_4 and filtered. The solvent was removed under reduced pressure and the crude residue purified by flash column chromatography under the specified conditions. The product containing fractions were

combined and concentrated. Et_2O (10 mL) and pentane (10 mL) were added and the solvent was removed under reduced pressure to afford the desired primary alcohol **47a** as a pale yellow oil (138 mg, 0.43 mmol, 62% over 2 steps).

R_f 0.25 (20% acetone/pentane);

IR (film)/cm^{-1} 3442 (OH br), 2974, 2930, 1689 (C=O), 1511, 1430, 1366, 1249, 1161, 1036, 829;

^{1}H NMR (500 MHz, DMSO-d_6, 373 K) δ 7.16 (dd, *J* = 8.9, 0.5 Hz, 2 H, HC_{Ar}), 6.83 (d, *J* = 8.8 Hz, 2 H, HC_{Ar}), 3.97 (t, *J* = 5.0 Hz, 1 H, OH), 3.82–3.75 (m, 2 H, NC*H*HCHAr and NC*H*HCH$_2$), 3.75 (s, 3 H, OCH_3), 3.40 (dd, *J* = 13.2, 4.2 Hz, 1 H, NCH*H*CH$_2$), 3.23–3.16 (m, 2 H, C*H*HOH and NCH*H*CHAr), 3.11 (ddd, *J* = 10.7, 6.1, 4.9 Hz, 1 H, CH*H*OH), 2.99–2.94 (m, 1 H, CHAr, *superimposed onto H_2O peak*), 2.01–1.94 (m, 1 H, C*H*CH$_2$OH), 1.62–1.53 (m, 2 H, NCH$_2$C*H$_2$*), 1.40 (s, 9 H, C(C*H$_3$*)$_3$);

^{13}C NMR (126 MHz, DMSO-d_6, 373 K) δ 157.4 (C_{Ar} quat), 153.7 (C=O carbamate), 133.3 (C_{Ar} quat), 128.8 (2 × C_{Ar}), 113.1 (2 × C_{Ar}), 78.0 (*C*(CH$_3$)$_3$), 60.8 (CH$_2$OH), 54.6 (OCH_3), 46.4 (N*C*H$_2$CHAr), 41.3 (N*C*H$_2$CH$_2$), 40.6 (*C*HAr), 40.0 (*C*HCH$_2$OH), 27.7 (C(*C*H$_3$)$_3$), 23.9 (NCH$_2$*C*H$_2$);

HRMS (ESI) *m/z* Calculated for $C_{18}H_{28}NO_4$ [M+H] 322.2018; Found 322.2023.

Characterisation information consistent with data previously reported in literature.[25]

tert-Butyl-trans-(±)-4-(hydroxymethyl)-3-(4-methoxyphenyl)piperidine-1-carboxylate (48a)

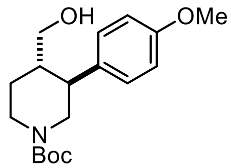

A flame-dried microwave vial (25 mL) was charged with *trans*-arylated piperidine **13a** (462 mg, 1.00 mmol, 1.0 equiv), di-*tert*-butyl dicarbonate (Boc_2O, 1.10 g, 5.00 mmol, 5.0 equiv) and 4-(dimethylamino)pyridine (DMAP, 25 mg, 0.20 mmol, 0.2 equiv). The reaction vessel was sealed and purged with argon, then MeCN (2.0 mL, 0.5 M) was added by syringe. The vial was placed in an oil bath and gradually warmed up to 70 ºC, then stirred for 3 h at 70 °C. The mixture was allowed to cool down and sat. aq. NH_4Cl (10 mL) and CH_2Cl_2 (15 mL) were added. The phases were separated, and the aqueous layer was extracted with CH_2Cl_2 (3 × 10 mL). The combined organic extracts were dried over Na_2SO_4 and filtered. The solvent was removed under reduced pressure to afford the crude *N*-Boc protected amide derivative **44a** which was carried forward to the reductive cleavage step without further purification.

A solution of the crude *N*-Boc-protected amide **44a** in dry THF (5.0 mL, 0.2 M) was added dropwise to a suspension of $LiAlH_4$ (156 mg, 4.00 mmol, 4.0 equiv) in dry THF (2.0 mL, 0.5 M) at 0 °C under argon atmosphere. The mixture was then stirred at 25 °C for 1 h or until TLC showed reaction completion. The reaction mixture was then quenched by slow addition of sat. aq. NH_4Cl (2-3 mL) at 0 °C and stirred at rt for 30 min. The suspension was filtered through a pad of Celite, eluting with EtOAc (3 × 5 mL). The phases were separated and the aqueous layer extracted with EtOAc (3 × 10 mL). The combined organic extracts were dried over Na_2SO_4 and filtered. The solvent was removed under reduced pressure and the crude residue purified by flash column chromatography under the specified conditions. The product containing fractions were combined and concentrated. Et_2O (10 mL) and pentane (10 mL) were added and the solvent was removed

under reduced pressure to afford the desired primary alcohol **48a** as a white solid (198 mg, 0.62 mmol, 62% over 2 steps).

R_f 0.34 (20% acetone/pentane);

mp = 55–57 °C (from Et_2O/pentane);

IR (film)/cm^{-1} 3443 (OH br), 2997, 2971, 2908, 1668 (C=O), 1511, 1422, 1364, 1245, 1164, 1131, 1035, 828, 732;

^{1}H NMR (400 MHz, DMSO-*d*$_6$, 373 K) δ 7.17–7.11 (m, 2 H, HC_{Ar}), 6.90–6.85 (m, 2 H, HC_{Ar}), 4.05 (ddt, *J* = 13.1, 4.5, 2.3 Hz, 1 H, NC*H*HCH_2), 3.87 (ddd, J = 12.9, 4.4, 1.8 Hz, 1 H, NC*H*HCHAr), 3.75 (s, 3 H, OCH_3), 3.22 (dt, *J* = 10.9, 3.2 Hz, 1 H, C*H*HOH), 3.07–3.05 (m, 1 H, CH*H*OH), 2.83 (td, *J* = 12.9, 2.9 Hz, 1 H, NCH*H*CH_2), 2.76 (dd, *J* = 13.0, 11.6 Hz, 1 H, NCH*H*CHAr), 2.39 (td, *J* = 11.3, 4.4 Hz, 1 H, CHAr), 1.92–1.84 (m, 1 H, NCH_2C*H*H), 1.79 (dtq, *J* = 10.9, 7.2, 3.5 Hz, 1 H, C*H*CH_2OH), 1.41 (s, 9 H, $C(CH_3)_3$), 1.38–1.25 (m, 1 H, NCH_2CH*H*);

^{13}C NMR (101 MHz, DMSO-*d*$_6$, 373 K) δ 157.7 (C_{Ar} quat), 153.6 (C=O carbamate), 133.6 (C_{Ar} quat), 128.1 (2 × C_{Ar}), 113.7 (2 × C_{Ar}), 78.2 (*C*$(CH_3)_3$), 62.6 (CH_2OH), 54.8 (OCH_3), 49.9 (N*C*H_2CHAr), 43.34 (N*C*H_2CH_2), 43.27 (*C*HAr), 42.6 (*C*HCH_2OH), 28.0 (NCH_2*C*H_2), 27.8 (C(*C*$H_3)_3$);

HRMS (ESI) *m/z* Calculated for $C_{18}H_{28}NO_4$ [M+H] 322.2018; Found 322.2025.

tert-Butyl-_cis_-(±)-3-(4-fluorophenyl)-4-(hydroxymethyl)piperidine-1-carboxylate (47b)

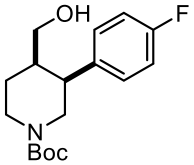

A flame-dried microwave vial (25 mL) was charged with *trans*-arylated piperidine **13b** (252 mg, 0.56 mmol, 1.0 equiv), di-*tert*-butyl dicarbonate (Boc_2O, 610 mg, 2.80 mmol, 5.0 equiv) and 4-(dimethylamino)pyridine (DMAP, 14 mg, 0.11 mmol, 0.2 equiv). The reaction vessel was sealed and purged with argon, then MeCN (1.2 mL, 0.5 M) was added by syringe. The vial was placed in an oil bath and gradually warmed up to 70 ºC, then stirred for 3 h at 70 °C. The mixture was allowed to cool down and sat. aq. NH_4Cl (10 mL) and CH_2Cl_2 (15 mL) were added. The phases were separated, and the aqueous layer was extracted with CH_2Cl_2 (3 × 10 mL). The combined organic extracts were dried over Na_2SO_4 and filtered. The solvent was removed under reduced pressure to afford the crude *N*-Boc protected amide derivative **43b** which was carried forward to the reductive cleavage step without further purification.

A solution of the crude *N*-Boc-protected amide **43b** in dry THF (2.8 mL, 0.2 M) was added dropwise to a suspension of $LiAlH_4$ (88 mg, 2.24 mmol, 4.0 equiv) in dry THF (1.2 mL, 0.5 M) at 0 °C under argon atmosphere. The mixture was then stirred at 25 °C for 1 h or until TLC showed reaction completion. The reaction mixture was then quenched by slow addition of sat. aq. NH_4Cl (2-3 mL) at 0 °C and stirred at rt for 30 min. The suspension was filtered through a pad of Celite, eluting with EtOAc (3 × 5 mL). The phases were separated and the aqueous layer extracted with EtOAc (3 × 10 mL). The combined organic extracts were dried over Na_2SO_4 and filtered. The solvent was removed under reduced pressure and the crude residue purified by flash column chromatography under the specified conditions. The product containing fractions were combined and concentrated. Et_2O (10 mL) and pentane (10 mL) were added and the solvent was removed under reduced pressure to afford the desired primary alcohol **47b** as a pale yellow oil (97 mg, 0.31 mmol, 56% over 2 steps).

R_f 0.25 (20% acetone/pentane);

IR (film)/cm^{-1} 3437 (OH br), 2920, 2850, 1689 (C=O), 1683, 1667, 1507, 1427, 1364, 1242, 1158, 1114, 1034, 925, 834, 767;

^{1}H NMR (500 MHz, DMSO-d_6, 373 K) δ 7.31–7.26 (m, 2 H, HC_{Ar}), 7.09–7.00 (m, 2 H, HC_{Ar}), 3.87–3.76 (m, 2 H, NC*H*HCHAr and NC*H*HCH$_2$), 3.40 (dd, *J* = 13.2, 4.1 Hz, 1 H, NCH*H*CHAr), 3.22–3.15 (m, 2 H, C*H*HOH and NCH*H*CH$_2$), 3.10–3.06 (m, 1 H, CH*H*OH), 3.04 (q, *J* = 4.9 Hz, 1 H, CHAr), 2.01 (dddd, *J* = 11.3, 7.8, 7.1, 5.6 Hz, 1 H, C*H*CH$_2$OH), 1.62–1.50 (m, 2 H, NCH$_2$C*H*$_2$), 1.39 (s, 9 H, C(CH$_3$)$_3$);

^{13}C NMR (126 MHz, DMSO-d_6, 373 K) δ 160.4 (d, *J* = 242.2 Hz, FC_{Ar} quat), 153.7 (C=O carbamate), 137.3 (d, *J* = 3.2 Hz, F-*p*-C_{Ar} quat), 129.6 (d, *J* = 7.6 Hz, 2 × F-*m*-C_{Ar}), 113.8 (d, *J* = 20.7 Hz, 2 × F-*o*-C_{Ar}), 78.1 (*C*(CH$_3$)$_3$), 60.9 (*C*H$_2$OH), 46.3 (N*C*H$_2$CHAr), 41.4 (N*C*H$_2$CH$_2$), 40.5 (*C*HCH$_2$OH), 39.9 (CHAr), 27.6 (C(*C*H$_3$)$_3$), 23.8 (NCH$_2$*C*H$_2$);

^{19}F NMR (471 MHz, DMSO-d_6, 373 K) δ -117.11–-117.30 (m);

HRMS (ESI) m/z Calculated for $C_{17}H_{25}NO_3F$ [M+H] 310.1818; Found 310.1824.

tert-Butyl-_trans_-(±)-3-(4-fluorophenyl)-4-(hydroxymethyl)piperidine-1-carboxylate (48b)

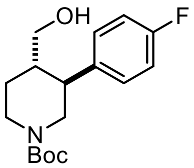

A flame-dried microwave vial (25 mL) was charged with *trans*-arylated piperidine **13b** (450 mg, 1.00 mmol, 1.0 equiv), di-*tert*-butyl dicarbonate (Boc$_2$O, 1.10 g, 5.00 mmol, 5.0 equiv) and 4-(dimethylamino)pyridine (DMAP, 25 mg, 0.20 mmol, 0.2 equiv). The reaction vessel was sealed and purged with argon, then MeCN (2.0 mL, 0.5 M) was added by syringe. The vial was placed in an oil bath and gradually warmed up to 70 ºC, then stirred for 3 h at 70 °C. The mixture was allowed to cool down and sat. aq. NH_4Cl (10 mL) and CH_2Cl_2 (15 mL) were added. The phases were separated, and the aqueous layer was extracted with CH_2Cl_2 (3 × 10 mL). The combined organic extracts were dried over Na_2SO_4 and filtered. The solvent was removed under reduced pressure to afford the crude *N*-Boc protected amide derivative **44b** which was carried forward to the reductive cleavage step without further purification.

A solution of the crude *N*-Boc-protected amide **44b** in dry THF (5.0 mL, 0.2 M) was added dropwise to a suspension of $LiAlH_4$ (156 mg, 4.00 mmol, 4.0 equiv) in dry THF (2.0 mL, 0.5 M) at 0 °C under argon atmosphere. The mixture was then stirred at 25 °C for 1 h or until TLC showed reaction completion. The reaction mixture was then quenched by slow addition of sat. aq. NH_4Cl (2-3 mL) at 0 °C and stirred at rt for 30 min. The suspension was filtered through a pad of Celite, eluting with EtOAc (3 × 5 mL). The phases were separated and the aqueous layer extracted with EtOAc (3 × 10 mL). The combined organic extracts were dried over Na_2SO_4 and filtered. The solvent was removed under reduced pressure and the crude residue purified by flash column chromatography under the specified conditions. The product containing fractions were combined and concentrated. Et_2O (10 mL) and pentane (10 mL) were added and the solvent was removed under reduced pressure to afford the desired primary alcohol **48b** as a pale yellow oil (163 mg, 0.53 mmol, 53% over 2 steps).

R_f 0.28 (20% acetone/pentane);

mp = 65–67 °C (from Et_2O/pentane);

IR (film)/cm^{-1} 3430 (OH br), 3001, 2974, 2920, 1669 (C=O), 1509, 1425, 1365, 1225, 1160, 1131, 908, 829, 731;

^{1}H NMR (400 MHz, DMSO-d_6, 373 K) δ 7.24–7.20 (m, 2 H, 2 × HC_{Ar}), 7.07–7.01 (m, 2 H, 2 × HC_{Ar}), 4.05–3.96 (m, 1 H, NC*H*HCH$_2$), 3.82 (ddd, *J* = 13.0, 4.4, 1.9 Hz, 1 H, NC*H*HCHAr), 3.20–3.16 (m, 1 H, C*H*HOH), 3.00 (dd, *J* = 10.7, 6.7 Hz, 1 H, CH*H*OH), 2.81 (td, *J* = 12.8, 2.5 Hz, 1 H, NCH*H*CH$_2$), 2.74 (dd, *J* = 13.0, 11.5 Hz, 1 H, NCH*H*CHAr), 2.41 (td, *J* = 11.0, 4.3 Hz, 1 H, CHAr), 1.86–1.73 (m, 2 H, NCH$_2$C*H*H and C*H*CH$_2$OH), 1.36 (s, 9 H, $C(CH_3)_3$), 1.33–1.24 (m, 1 H, NCH$_2$CH*H*);

^{13}C NMR (101 MHz, DMSO-d_6, 373 K) δ 161.2 (d, *J* = 241.8 Hz, FC_{Ar} quat), 154.4 (C=O carbamate), 138.1 (d, *J* = 2.6 Hz, F-*p*-C_{Ar} quat), 129.5 (d, *J* = 7.6 Hz, 2 × F-*m*-C_{Ar}), 115.1 (d, *J* = 21.2 Hz, 2 × F-*o*-C_{Ar}), 79.2 (*C*(CH$_3$)$_3$), 63.1 (*C*H$_2$OH), 50.2 (N*C*H$_2$CHAr), 43.9 (CHAr), 43.8 (N*C*H$_2$CH$_2$), 42.9 (*C*HCH$_2$OH), 28.3 (NCH$_2$*C*H$_2$), 28.2 (C(*C*H$_3$)$_3$);

^{19}F{^{1}H} NMR (471 MHz, DMSO-d_6, 373 K) δ –116.50 (s);

HRMS (ESI) m/z Calculated for $C_{17}H_{25}NO_3F$ [M+H] 310.1818; Found 310.1809.

cis-(±)-4-Carboxy-3-(4-methoxyphenyl)piperidin-1-ium chloride (FRAG32)

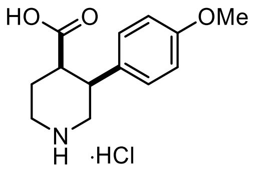

4 N HCl in 1,4-dioxane (1.2 mL, 4.80 mmol, 10.0 equiv) was added to *N*-Boc protected piperidine **45a** (151 mg, 0.45 mmol) at 0 °C. The resulting solution was warmed up to 20 °C and the product began to crush out of solution. The reaction mixture was stirred at 20 °C for 3 h and then ice-cold Et_2O (2 mL) was added. The reaction mixture was filtered, and the precipitate was washed with further ice-cold Et_2O (2 × 5 mL). The solid precipitate was dried under reduced pressure to afford the deprotected piperidine product as the corresponding hydrochloride salt **FRAG32** as a pale yellow solid (105 mg, 0.39 mmol, 86%).

mp = 256–258 °C (from dioxane/Et_2O);

IR (film)/cm^{-1} 3012 (OH br), 2937, 2825, 1707 (C=O), 1517, 1420, 1252, 1156, 1122, 1029, 824, 705;

^{1}H NMR (400 MHz, CD_3OD) δ 7.20 (d, *J* = 8.7 Hz, 2 H, 2 × HC_{Ar}), 6.90 (d, *J* = 8.8 Hz, 2 H, 2 × HC_{Ar}), 4.01 (t, *J* = 12.5 Hz, 1 H, NC*H*HCHAr), 3.77 (s, 3 H, OCH_3), 3.47 (td, *J* = 12.3, 4.9 Hz, 1 H, NC*H*HCH$_2$), 3.42–3.32 (m, 2 H, NCH*H*CHAr, NCH*H*CH$_2$), 3.30–3.26 (m, 1 H, CHAr), 3.09 (q, *J* = 4.2 Hz, 1 H, CH(C=O)), 2.32–2.15 (m, 2 H, NCH$_2$C*H$_2$*);

^{13}C NMR (101 MHz, CD_3OD) δ 176.2 (C=O acid), 160.5 (C_{Ar} quat), 131.8 (C_{Ar} quat), 129.4 (2 × C_{Ar}), 115.1 (2 × C_{Ar}), 55.7 (OCH_3), 44.6 (N*C*H$_2$CHAr), 43.4 (*C*H(C=O)), 40.9 (N*C*H$_2$CH$_2$), 40.8 (CHAr), 26.2 (NCH$_2$*C*H$_2$);

HRMS (ESI) *m/z* Calculated for $C_{13}H_{18}NO_3$ [M–Cl] 236.1287; Found 236.1290.

Characterisation information consistent with data previously reported in literature.[25]

trans-(±)-4-Carboxy-3-(4-methoxyphenyl)piperidin-1-ium chloride (FRAG34)

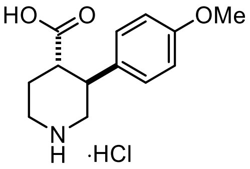

4 N HCl in 1,4-dioxane (0.8 mL, 3.20 mmol, 10.0 equiv) was added to *N*-Boc protected piperidine **46a** (110 mg, 0.33 mmol) at 0 °C. The resulting solution was warmed up to 20 ºC and the product began to crush out of solution. The reaction mixture was stirred at 20 °C for 3 h and then ice-cold Et_2O (2 mL) was added. The reaction mixture was filtered, and the precipitate was washed with further ice-cold Et_2O (2 × 5 mL). The solid precipitate was dried under reduced pressure to afford the deprotected piperidine product as the corresponding hydrochloride salt **FRAG34** as a pale yellow solid (86 mg, 0.32 mmol, 98%).

mp = 278–280 °C (from dioxane/Et_2O);

IR (film)/cm^{-1} 2961 (OH), 2934, 2781, 1718 (C=O), 1513, 1249, 1182, 1125, 1026, 821;

^{1}H NMR (400 MHz, CD_3OD) δ 7.24 (d, *J* = 8.7 Hz, 2 H, 2 × HC_{Ar}), 6.89 (d, *J* = 8.7 Hz, 2 H, 2 × HC_{Ar}), 3.77 (s, 3 H, OCH_3), 3.52 (ddd, *J* = 12.0, 4.7, 2.6 Hz, 1 H, NC*H*HCH_2), 3.35 (ddd, *J* = 6.3, 3.6, 2.2 Hz, 1 H, NC*H*HCHAr), 3.23–3.08 (m, 3 H, NCH*H*CHAr, NCH*H*CH_2 and CHAr), 2.95 (td, *J* = 11.8, 3.6 Hz, 1 H, CH(C=O)), 2.27 (dq, *J* = 14.4, 3.1 Hz, 1 H, NCH_2C*H*H), 2.09–1.94 (m, 1 H, NCH_2CH*H*);

^{13}C NMR (101 MHz, CD_3OD) δ 176.1 (C=O acid), 160.8 (C_{Ar} quat), 131.7 (C_{Ar} quat), 130.0 (2 × C_{Ar}), 115.3 (2 × C_{Ar}), 55.7 (OCH_3), 48.6 (N*C*H_2CHAr), 47.1 (*C*H(C=O)), 44.2 (N*C*H_2CH_2), 43.0 (CHAr), 27.6 (NCH_2*C*H_2);

HRMS (ESI) *m/z* Calculated for $C_{13}H_{18}NO_3$ [M-Cl] 236.1287; Found 236.1294.

cis-(±)-4-Carboxy-3-(4-fluorophenyl)piperidin-1-ium chloride (FRAG33)

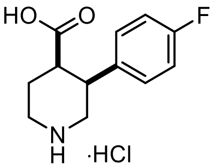

4 N HCl in 1,4-dioxane (0.8 mL, 3.10 mmol, 10.0 equiv) was added to *N*-Boc protected piperidine **45b** (100 mg, 0.31 mmol) at 0 °C. The resulting solution was warmed up to 20 ºC and the product began to crush out of solution. The reaction mixture was stirred at 20 °C for 3 h and then ice-cold Et_2O (2 mL) was added. The reaction mixture was filtered, and the precipitate was washed with further ice-cold Et_2O (2 × 5 mL). The solid precipitate was dried under reduced pressure to afford the deprotected piperidine product as the corresponding hydrochloride salt **FRAG33** as a grey solid (79 mg, 0.30 mmol, 98%).

mp= 250–253 °C (from dioxane/Et_2O).

IR (film)/cm^{-1} 2923 (OH br), 2852 (NH br), 1713 (C=O), 1549, 1508, 1415, 1303, 1157, 1041, 978, 835;

^{1}H NMR (400 MHz, CD_3OD, 298 K) δ 7.31–7.24 (m, 2 H, HC_{Ar}), 7.11–7.00 (m, 2 H, HC_{Ar}), 4.00 (t, *J* = 12.4 Hz, 1 H, NC*H*HCH), 3.52–3.28 (m, 4 H, NCH*H*CH, NC*H*$_2$$CH_2$ and C*H*Ar), 3.10 (q, *J* = 4.1 Hz, 1 H, CH(C=O)), 2.21 (dt, *J* = 7.6, 3.4 Hz, 2 H, NCH_2C*H*$_2$).

^{13}C NMR (101 MHz, CD_3OD, 298 K) δ 175.9 (COOH), 164.8 (FC_{Ar} quat), 136.0 (F-*p*-C_{Ar} quat), 130.3 (d, *J* = 8.1 Hz, 2 × F-*m*-C_{Ar}), 116.4 (d, *J* = 21.6 Hz, 2 × F-*o*-C_{Ar}), 44.4 (N*C*H_2CH), 43.4 (*C*HCOOH), 41.0 (CHAr), 40.7 (N*C*H_2CH_2), 26.3 (NCH_2*C*H_2);

^{19}F NMR (376 MHz, CD_3OD, 298 K) δ -117.04 (d, *J* = 8.3 Hz).

HRMS (ESI) *m/z* Calculated for $C_{13}H_{20}NO_2$ [M–Cl] 224.1087; Found 224.1086.

trans-(±)-4-Carboxy-3-(4-fluorophenyl)piperidin-1-ium chloride (FRAG35)

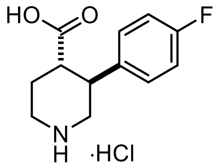

4 N HCl in 1,4-dioxane (0.9 mL, 3.60 mmol, 10.0 equiv) was added to *N*-Boc protected piperidine **46b** (114 mg, 0.35 mmol) at 0 °C. The resulting solution was warmed up to 20 ºC and the product began to crush out of solution. The reaction mixture was stirred at 20 °C for 3 h and then ice-cold Et_2O (2 mL) was added. The reaction mixture was filtered, and the precipitate was washed with further ice-cold Et_2O (2 × 5 mL). The solid precipitate was dried under reduced pressure to afford the deprotected piperidine product as the corresponding hydrochloride salt **FRAG35** as a pale yellow solid (68 mg, 0.26 mmol, 75%).

mp = 276–278 °C (from dioxane/Et_2O);

IR (film)/cm^{-1} 3399 (br OH), 2949, 2806, 1720 (C=O), 1511, 1418, 1224, 1184, 1160, 1122, 833;

^{1}H NMR (400 MHz, CD_3OD) δ 7.40–7.32 (m, 2 H, 2 × HC_{Ar}), 7.12–7.03 (m, 2 H, 2 × HC_{Ar}), 3.58–3.49 (m, 1 H, NC*H*HCH_2), 3.38 (ddd, *J* = 12.0, 3.4, 1.3 Hz, 1 H, NC*H*HCHAr), 3.27–3.12 (m, 3 H, NCH*H*CH_2, NCH*H*CHAr and CHAr), 3.05–2.94 (m, 1 H, CH(C=O)), 2.29 (dq, *J* = 14.4, 2.9 Hz, 1 H, NCH_2C*H*H), 2.09–1.95 (m, 1 H, NCH_2CH*H*);

^{13}C NMR (101 MHz, CD_3OD) δ 175.8 (C=O acid), 163.7 (d, J_{CF} = 245 Hz, FC_{Ar} quat), 136.0 (d, J_{CF} = 3.2 Hz, F-*p*-C_{Ar} quat), 130.9 (d, J_{CF} = 8.3 Hz, 2 × F-*m*-C_{Ar}), 116.6 (d, J_{CF} = 21.7 Hz, 2 × F-*o*-C_{Ar}), 49.1 (N*C*H_2CHAr), 47.0 (*C*H(C=O)), 44.2 (N*C*H_2CH_2), 43.0 (CHAr), 27.5 (NCH_2*C*H_2);

^{19}F{^{1}H} NMR (376 MHz, CD_3OD) δ –116.62 (s);

HRMS (ESI) *m/z* Calculated for $C_{12}H_{15}NO_2F$ [M-Cl] 224.1087; Found 224.1086.

cis-(±)-4-Carbamoyl-3-(4-methoxyphenyl)piperidin-1-ium chloride (FRAG36)

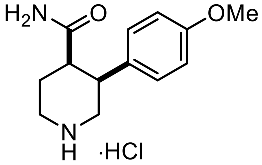

4 N HCl in 1,4-dioxane (0.8 mL, 3.20 mmol, 10.0 equiv) was added to *N*-Boc protected piperidine **49a** (100 mg, 0.30 mmol) at 0 °C. The resulting solution was warmed up to 20 ºC and the product began to crush out of solution. The reaction mixture was stirred at 20 °C for 3 h and then ice-cold Et_2O (2 mL) was added. The reaction mixture was filtered, and the precipitate was washed with further ice-cold Et_2O (2 × 5 mL). The solid

precipitate was dried under reduced pressure to afford the deprotected piperidine product as the corresponding hydrochloride salt **FRAG36** as a pale yellow solid (74 mg, 0.27 mmol, 92%).

mp = 271–272 °C (from dioxane/Et_2O);

IR (film)/cm^{-1} 3421 (NH br), 2995, 2957, 2833, 2533, 2363, 2115, 1641 (C=O), 1513, 1451, 1248, 1183, 1027, 831;

^{1}H NMR (400 MHz, CD_3OD) δ 7.22–7.14 (m, 2 H, 2 × HC_{Ar}), 6.91–6.86 (m, 2 H, 2 × HC_{Ar}), 4.17 (t, *J* = 12.3 Hz, 1 H, NC*H*HCHAr), 3.77 (s, 3 H, OCH_3), 3.76–3.65 (m, 1 H, NC*H*HCH$_2$), 3.31–3.20 (m, 3 H, NCH*H*CHAr, NCH*H*CH$_2$ and CHAr), 2.92 (dt, *J* = 6.8, 3.2 Hz, 1 H, CH(C=O)), 2.27–2.08 (m, 2 H, NCH$_2$C*H$_2$*);

^{13}C NMR (101 MHz, CD_3OD) δ 178.1 (C=O amide), 160.6 (C_{Ar} quat), 132.0 (C_{Ar} quat), 129.6 (2 × C_{Ar}), 115.1 (2 × C_{Ar}), 55.7 (OCH_3), 44.7 (N*C*H$_2$CHAr), 43.0 (*C*H(C=O)), 41.6 (CHAr), 41.0 (N*C*H$_2$CH$_2$), 27.1 (NCH$_2$*C*H$_2$);

HRMS (ESI) *m/z* Calculated for $C_{13}H_{19}N_2O_2$ [M–Cl] 235.1447; Found 235.1454.

Characterisation information consistent with data previously reported in literature.[25]

trans-(±)-4-Carbamoyl-3-(4-methoxyphenyl)piperidin-1-ium chloride (FRAG38)

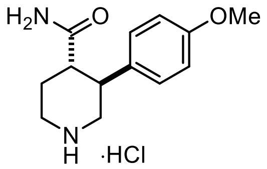

4 N HCl in 1,4-dioxane (0.9 mL, 3.60 mmol, 10.0 equiv) was added to *N*-Boc protected piperidine **50a** (116 mg, 0.35 mmol) at 0 °C. The resulting solution was warmed up to 20 ºC and the product began to crush out of solution. The reaction mixture was stirred at 20 °C for 3 h and then ice-cold Et_2O (2 mL) was added. The reaction mixture was filtered, and the precipitate was washed with further ice-cold Et_2O (2 × 5 mL). The solid precipitate was dried under reduced pressure to afford the deprotected piperidine product as the corresponding hydrochloride salt **FRAG38** as a pale yellow solid (78 mg, 0.29 mmol, 83%).

mp not observed up to 280 °C (from dioxane/Et_2O);

IR (film)/cm^{-1} 3355 (br NH), 3183 (br NH), 2952, 2803, 1670 (C=O), 1513, 1446, 1252, 1185, 835;

^{1}H NMR (400 MHz, CD_3OD) δ 7.31–7.19 (m, 2 H, 2 × HC_{Ar}), 6.96–6.85 (m, 2 H, 2 × HC_{Ar}), 3.78 (s, 3 H, OCH_3), 3.53 (ddt, *J* = 12.9, 4.3, 2.1 Hz, 1 H, NC*H*HCH$_2$), 3.37 (dd, *J* = 8.0, 1.4 Hz, 1 H, NC*H*HCHAr), 3.24–3.11 (m, 3 H, CHAr, NCH*H*CHAr and NCH*H*CH$_2$), 2.87 (ddt, *J* = 11.6, 8.4, 3.5 Hz, 1 H, CH(C=O)), 2.21–2.12 (m, 1 H, NCH$_2$C*H*H), 2.11–1.98 (m, 1 H, NCH$_2$CH*H*);

^{13}C NMR (101 MHz, CD_3OD) δ 177.4 (C=O amide), 160.7 (C_{Ar} quat), 131.6 (C_{Ar} quat), 130.0 (2 × C_{Ar}), 115.3 (2 × C_{Ar}), 55.7 (OCH_3), 49.4 (N*C*H$_2$CHAr), 47.7 (*C*H(C=O)), 44.3 (N*C*H$_2$CH$_2$), 42.8 (CHAr), 27.7 (NCH$_2$*C*H$_2$);

HRMS (ESI) *m/z* Calculated for $C_{13}H_{19}N_2O_2$ [M-Cl] 235.1447; Found 235.1449.

cis-(±)-4-Carbamoyl-3-(4-fluorophenyl)piperidin-1-ium chloride (FRAG37)

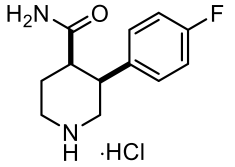

4 N HCl in 1,4-dioxane (0.9 mL, 3.70 mmol, 10.0 equiv) was added to *N*-Boc protected piperidine **49b** (120 mg, 0.37 mmol) at 0 °C. The resulting solution was warmed up to 20 ºC and the product began to crush out of solution. The reaction mixture was stirred at 20 °C for 3 h and then ice-cold Et_2O (2 mL) was added. The reaction mixture was filtered, and the precipitate was washed with further ice-cold Et_2O (2 × 5 mL). The solid precipitate was dried under reduced pressure to afford the deprotected piperidine product as the corresponding hydrochloride salt **FRAG37** as a pale yellow solid (87 mg, 90%).

mp > 250 °C (from dioxane/Et_2O);

IR (film)/cm^{-1} 3374 (CONH br), 3315 (CONH br), 2956 (NH br), 2799 (NH br), 1657(C=O), 1510, 1445, 1327, 1226, 1111, 1017, 833, 810;

^{1}H NMR (400 MHz, CD_3OD, 298 K) δ 7.33–7.23 (m, 2 H, HC_{Ar}), 7.11–7.00 (m, 2 H, HC_{Ar}), 4.17 (t, *J* = 13.3 Hz, 1 H, NC*H*HCHAr), 3.66 (td, *J* = 13.0, 3.8 Hz, 1 H, NC*H*HCH$_2$), 3.35–3.25 (m, 3 H, NCH*H*CHAr, NCH*H*CH$_2$ and CHAr), 2.92 (br s, 1H, CH(C=O)), 2.29–2.06 (m, 2 H, NCH$_2$C*H$_2$*);

^{13}C NMR (101 MHz, CD_3OD, 298 K) δ 177.9 (C=O amide), 136.1 (F-*p*-C_{Ar} quat), 130.5 (d, *J* = 8.0 Hz, 2 × F-*m*-C_{Ar}), 116.4 (d, *J* = 21.6 Hz, 2 × F-*o*-C_{Ar}), 44.4 (N*C*H$_2$CH), 42.9 (*C*H(C=O)), 41.51 (CHAr), 41.0 (N*C*H$_2$CH$_2$), 27.1 (NCH$_2$*C*H$_2$), FC_{Ar} quat not observed;

^{19}F NMR (377 MHz, CD_3OD, 298K) δ -117.03 (s);

HRMS (ESI) *m/z* Calculated for $C_{12}H_{16}N_2OF$ [M–Cl] 223.1247; Found 223.1254.

trans-(±)-4-Carbamoyl-3-(4-fluorophenyl)piperidin-1-ium chloride (FRAG39)

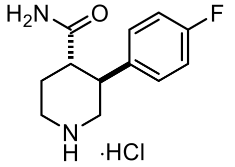

4 N HCl in 1,4-dioxane (1.2 mL, 4.80 mmol, 10.0 equiv) was added to *N*-Boc protected piperidine **50b** (145 mg, 0.45 mmol) at 0 °C. The resulting solution was warmed up to 20 ºC and the product began to crush out of solution. The reaction mixture was stirred at 20 °C for 3 h and then ice-cold Et_2O (2 mL) was added. The reaction mixture was filtered, and the precipitate was washed with further ice-cold Et_2O (2 × 5 mL). The solid precipitate was dried under reduced pressure to afford the deprotected piperidine product as the corresponding hydrochloride salt **FRAG39** as a pale yellow solid (65 mg, 0.25 mmol, 55%).

mp not observed up to 280 °C (from dioxane/Et_2O);

IR (film)/cm^{-1} 3336 (br NH), 3178 (br NH), 2953, 2764, 2124, 1664 (C=O), 1605, 1510, 1422, 1224, 835, 538;

^{1}H NMR (400 MHz, CD_3OD) δ 7.43–7.27 (m, 2 H, 2 × HC_{Ar}), 7.12–7.01 (m, 2 H, 2 × HC_{Ar}), 3.53 (dq, *J* = 12.7, 2.0 Hz, 1 H, NC*H*HCH$_2$), 3.38 (dq, *J* = 8.2, 1.8 Hz, 1 H, NC*H*HCHAr), 3.27–3.13 (m, 3 H, NCH*H*CH$_2$, NCH*H*CHAr and

CHAr), 2.88 (td, J = 11.1, 4.0 Hz, 1 H, CH(C=O)), 2.16 (dtd, J = 14.5, 3.6, 2.3 Hz, 1 H, NCH_2C*H*H), 2.03 (dddd, J = 14.5, 13.6, 11.9, 4.2 Hz, 1 H, NCH_2CH*H*);

^{13}C NMR (101 MHz, CD_3OD) δ 177.0 (C=O amide), 163.8 (d, J_{CF} = 243.5 Hz, FC_{Ar} quat), 135.8 (d, J_{CF} = 3.2 Hz, F-*p*-C_{Ar} quat), 131.0 (d, J_{CF} = 8.2 Hz, 2 × F-*m*-C_{Ar}), 116.6 (d, J_{CF} = 22.0 Hz, 2 × F-*o*-C_{Ar}), 49.1 (N*C*H_2CHAr), 47.6 (*C*H(C=O)), 44.3 (N*C*H_2CH_2), 42.8 (CHAr), 27.7 (NCH_2*C*H_2);

^{19}F{^{1}H} NMR (376 MHz, CD_3OD) δ –116.68 (s);

HRMS (ESI) *m/z* Calculated for $C_{12}H_{16}N_2OF$ [M-Cl] 223.1247; Found 223.1242.

Note: Hygroscopic solid.

cis-(±)-4-Cyano-3-(4-methoxyphenyl)piperidin-1-ium chloride (FRAG44)

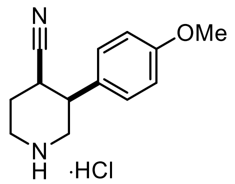

4 N HCl in 1,4-dioxane (0.9 mL, 3.60 mmol, 10.0 equiv) was added to *N*-Boc protected piperidine **51a** (110 mg, 0.35 mmol) at 0 °C. The resulting solution was warmed up to 20 ºC and the product began to crush out of solution. The reaction mixture was stirred at 20 °C for 3 h and then ice-cold Et_2O (2 mL) was added. The reaction mixture was filtered, and the precipitate was washed with further ice-cold Et_2O (2 × 5 mL). The solid precipitate was dried under reduced pressure to afford the deprotected piperidine product as the corresponding hydrochloride salt **FRAG44** as a pale yellow solid (97 mg, 0.33 mmol, 94%).

mp not observed up to 280 °C (from dioxane/Et_2O);

IR (film)/cm^{-1} 3124, 2962, 2919, 2704, 2598, 1514, 1440, 1251 (CN), 1183, 1119, 1031, 817, 787;

^{1}H NMR (400 MHz, CD_3OD) δ 7.29 (d, J = 8.3 Hz, 2 H, 2 × HC_{Ar}), 6.96 (d, J = 8.4 Hz, 2 H, 2 × HC_{Ar}), 3.80 (s, 3 H, OCH_3), 3.61–3.49 (m, 4 H, NC*H_2*CHAr, CHCN and NC*H*HCH_2), 3.39 (dt, J = 11.9, 4.2 Hz, 1 H, CHAr), 3.29–3.17 (m, 1 H, NCH*H*CH_2), 2.36–2.26 (m, 2 H, NCH_2C*H_2*);

^{13}C NMR (101 MHz, CD_3OD) δ 161.1 (C_{Ar} quat), 130.4 (C_{Ar} quat), 129.4 (2 × C_{Ar}), 119.6 (CN), 115.4 (2 × C_{Ar}), 55.8 (OCH_3), 45.3 (N*C*H_2CHAr), 41.5 (N*C*H_2CH_2), 40.4 (CHAr), 34.2 (CHCN), 26.9 (NCH_2*C*H_2);

HRMS (ESI) *m/z* Calculated for $C_{15}H_{20}N_3O$ [M+CH_3CN–Cl] 258.1606; Found 258.1613.

Characterisation information consistent with data previously reported in literature.[25]

cis-(±)-4-Cyano-3-(4-fluorophenyl)piperidin-1-ium chloride (FRAG45)

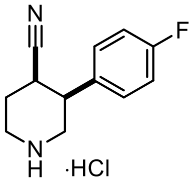

4 N HCl in 1,4-dioxane (0.9 mL, 3.60 mmol, 10.0 equiv) was added to *N*-Boc protected piperidine **51b** (110 mg, 0.35 mmol) at 0 °C. The resulting solution was warmed up to 20 °C and the product began to crush out of solution. The reaction mixture was stirred at 20 °C for 3 h and then ice-cold Et_2O (2 mL) was added. The reaction mixture was filtered, and the precipitate was washed with further ice-cold Et_2O (2 × 5 mL). The solid precipitate was dried under reduced pressure to afford the deprotected piperidine product as the corresponding hydrochloride salt **FRAG45** as a pale yellow solid (70 mg, 0.28 mmol, 80%).

mp = 250–252 °C (from dioxane/Et_2O);

IR (film)/cm^{-1} 2918 (NH br), 2698 (NH br), 2234 (C=N), 1704, 1509, 1432, 1356, 1221, 1112, 1011, 968, 831;

^1H NMR (400 MHz, CD_3OD) δ 7.43–7.36 (m, 2 H, 2 × HC_{Ar}), 7.20–7.09 (m, 2 H, 2 × HC_{Ar}), 3.66–3.48 (m, 4 H, NCH_2CHAr *and* NCH_2CH_2), 3.40 (s, 1 H, CHAr), 3.23 (ddd, *J* = 13.5, 12.3, 4.7 Hz, 1 H, CHCN), 2.36–2.21 (m, 2 H, NCH_2CH_2);

^{13}C NMR (101 MHz, CD_3OD) δ 162.1 (d, J_{CF} = 243.5 Hz, FC_{Ar} quat), 134.8 (d, J_{CF} = 3.2 Hz, F-*p*-C_{Ar} quat), 131.3 (CN), 130.4 (d, J_{CF} = 8.4 Hz, 2 × F-*m*-CAr), 116.9 (d, J_{CF} = 21.7 Hz, 2 × F-*o*-CAr), 45.1 (NCH_2CHAr), 41.5 (CHAr), 40.5 (NCH_2CH_2), 34.0 (*C*HCN), 26.9 (NCH_2CH_2);

$^{19}F\{^1H\}$ NMR (376 MHz, CD_3OD) δ –115.64 (s);

HRMS (ESI) *m/z* Calculated for $C_{12}H_{14}N_2F$ [M–Cl] 205.1141; Found 205.1148.

cis-(±)-4-(Hydroxymethyl)-3-(4-methoxyphenyl)piperidin-1-ium chloride (FRAG40)

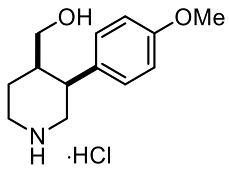

4 N HCl in 1,4-dioxane (0.9 mL, 3.60 mmol, 10.0 equiv) was added to *N*-Boc protected piperidine **47a** (115 mg, 0.36 mmol) at 0 °C. The resulting solution was warmed up to 20 °C and the product began to crush out of solution. The reaction mixture was stirred at 20 °C for 3 h and then ice-cold Et_2O (2 mL) was added. The reaction mixture was filtered, and the precipitate was washed with further ice-cold Et_2O (2 × 5 mL). The solid precipitate was dried under reduced pressure to afford the deprotected piperidine product as the corresponding hydrochloride salt **FRAG40** as a pale yellow solid (75 mg, 0.30 mmol, 82%).

mp= 268–269 °C (from dioxane/Et_2O);

IR (film)/cm^{-1} 3370 (OH br), 2958, 2835, 2490, 2126, 1515, 1249, 1183, 1033, 988;

^{1}H NMR (400 MHz, CD_3OD) δ 7.19–7.11 (m, 2 H, 2 × HC_{Ar}), 6.96–6.89 (m, 2 H, 2 × HC_{Ar}), 3.78 (s, 3 H, OCH_3), 3.70–3.60 (m, 2 H, CH_2OH), 3.42–3.32 (m, 5 H, NCH_2CHAr, NC$H_2$$CH_2$ and CHAr), 2.25 (dq, *J* = 14.7, 2.8 Hz, 1 H, NCH_2C*H*H), 2.14 (m, 1 H, C*H*CH_2OH), 2.11–2.01 (m, 1 H, NCH_2CH*H*);

^{13}C NMR (101 MHz, CD_3OD) δ 160.3 (C_{Ar} quat), 132.4 (C_{Ar} quat), 129.4 (2 × C_{Ar}), 115.2 (2 × C_{Ar}), 60.2 (CH_2OH), 55.7 (OCH_3), 45.1 (N*C*H_2CHAr), 41.7 (CHAr), 41.3 (N*C*$H_2$$CH_2$), 39.7 (*C*H$CH_2$OH), 25.2 ($NCH_2$*C*$H_2$);

HRMS (ESI) *m/z* Calculated for $C_{13}H_{20}NO_2$ [M–Cl] 222.1494; Found 222.1503.

Characterisation information consistent with data previously reported in literature.[25]

trans-(±)-4-(Hydroxymethyl)-3-(4-methoxyphenyl)piperidin-1-ium chloride (FRAG42)

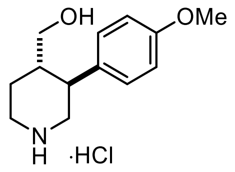

4 N HCl in 1,4-dioxane (1.1 mL, 4.40 mmol, 10.0 equiv) was added to *N*-Boc protected piperidine **48a** (140 mg, 0.43 mmol) at 0 °C. The resulting solution was warmed up to 20 °C and the product began to crush out of solution. The reaction mixture was stirred at 20 °C for 3 h and then ice-cold Et_2O (2 mL) was added. The reaction mixture was filtered, and the precipitate was washed with further ice-cold Et_2O (2 × 5 mL). The solid precipitate was dried under reduced pressure to afford the deprotected piperidine product as the corresponding hydrochloride salt **FRAG42** as a pale yellow solid (90 mg, 0.36 mmol, 83%).

mp = 218–220 °C (from Et_2O/dioxane);

IR (film)/cm^{-1} 3370 (br OH), 2952, 2803, 1513, 1252, 1178, 1029, 828;

^{1}H NMR (400 MHz, CD_3OD) δ 7.22 (d, *J* = 8.7 Hz, 2 H, 2 × HC_{Ar}), 6.92 (d, *J* = 8.7 Hz, 2 H, 2 × HC_{Ar}), 3.78 (s, 3 H, OCH_3), 3.52 (ddt, *J* = 12.7, 4.2, 2.0 Hz, 1 H, NC*H*HCH_2), 3.36 (dd, *J* = 10.9, 3.0 Hz, 1 H, C*H*HOH), 3.30–3.25 (m, 1 H, NC*H*HCHAr), 3.22–3.06 (m, 3 H, CH*H*OH, NCH*H*CH_2 and NCH*H*CHAr), 2.81 (td, *J* = 12.0, 4.2 Hz, 1 H, CHAr), 2.18 (dq, *J* = 14.5, 3.1 Hz, 1 H, NCH_2C*H*H), 2.07–1.93 (m, 1 H, C*H*CH_2OH), 1.75 (tdd, *J* = 14.1, 11.8, 4.3 Hz, 1 H, NCH_2CH*H*);

^{13}C NMR (101 MHz, CD_3OD) δ 160.7 (C_{Ar} quat), 132.2 (C_{Ar} quat), 129.9 (2 × C_{Ar}), 115.4 (2 × C_{Ar}), 63.8 (CH_2OH), 55.7 (OCH_3), 50.3 (N*C*H_2CHAr), 45.3 (N*C*$H_2$$CH_2$), 42.8 (CHAr), 42.6 (*C*HCH_2OH), 27.0 (NCH_2*C*H_2);

HRMS (ESI) *m/z* Calculated for $C_{13}H_{20}NO_2$ [M-Cl] 222.1494; Found 222.1493.

cis-(±)-4-(Hydroxymethyl)-3-(4-fluorophenyl)piperidin-1-ium chloride (FRAG41)

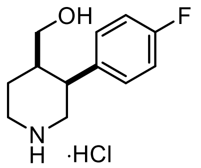

4 N HCl in 1,4-dioxane (0.8 mL, 3.20 mmol, 10.0 equiv) was added to *N*-Boc protected piperidine **47b** (95 mg, 0.30 mmol) at 0 °C. The resulting solution was warmed up to 20 °C and the product began to crush out of

solution. The reaction mixture was stirred at 20 °C for 3 h and then ice-cold Et_2O (2 mL) was added. The reaction mixture was filtered, and the precipitate was washed with further ice-cold Et_2O (2 × 5 mL). The solid precipitate was dried under reduced pressure to afford the deprotected piperidine product as the corresponding hydrochloride salt **FRAG41** as a pale yellow solid (67 mg, 0.29 mmol, 97%).

mp = 200–203 °C (from dioxane/Et_2O);

IR (film)/cm^{-1} 3351 (OH br), 2927 (NH br), 2793, 1688, 1600, 1510, 1444,1363, 1225, 1161, 1020, 940, 821, 753;

^{1}H NMR (400 MHz, CD_3OD) δ 7.32–7.21 (m, 2 H, 2 × HC_{Ar}), 7.14–7.03 (m, 2 H, 2 × HC_{Ar}), 3.73–3.56 (m, 2 H, NC*H*HCH and C*H*HOH), 3.42–3.25 (m, 5 H, NCH_2CH_2, NCH*H*CH, CHAr, CH*H*OH), 2.24 (dq, *J* = 14.6, 2.8 Hz, 1 H, NCH_2C*H*H), 2.16–2.03 (m, 2 H, NCH_2CH*H* and C*H*CH_2OH);

^{13}C NMR (101 MHz, CD_3OD) δ 164.6 (d, *J* = 242.8 Hz, FC_{Ar} quat), 136.6 (d, *J* = 3.2 Hz, F-*p*-C_{Ar} quat), 130.2 (d, *J* = 8.1 Hz, 2 × F-*m*-C_{Ar}), 116.5 (d, *J* = 21.4 Hz, 2 × F-*o*-C_{Ar}), 60.3 (*C*H_2OH), 44.9 (N*C*H_2CH), 41.7 (N*C*H_2CH_2), 41.3 (CHAr), 39.5 (*C*HCH_2OH), 25.8 (NCH_2*C*H_2);

^{19}F NMR (376 MHz, CD_3OD) δ –117.45;

HRMS (ESI) *m/z* Calculated for $C_{12}H_{17}NOF$ [M–Cl] 210.1294; Found 210.1297.

trans-(±)-4-(Hydroxymethyl)-3-(4-fluorophenyl)piperidin-1-ium chloride (FRAG43)

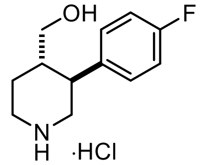

4 N HCl in 1,4-dioxane (1.3 mL, 5.20 mmol, 10.0 equiv) was added to *N*-Boc protected piperidine **48b** (155 mg, 0.50 mmol) at 0 °C. The resulting solution was warmed up to 20 °C and the product began to crush out of solution. The reaction mixture was stirred at 20 °C for 30 min and then ice-cold Et_2O (2 mL) was added. The reaction mixture was filtered, and the precipitate was washed with further ice-cold Et_2O (2 × 5 mL). The solid precipitate was dried under reduced pressure to afford the deprotected piperidine product as the corresponding hydrochloride salt **FRAG43** as a pale yellow solid (69 mg, 0.28 mmol, 56%).

mp = 214–216 °C (from Et_2O/dioxane);

IR (film)/cm^{-1} 3366 (br OH), 2984, 2796, 1510, 1224, 1161, 1093, 1068, 1032, 833;

^{1}H NMR (400 MHz, CD_3OD) δ 7.42–7.34 (m, 2 H, 2 × HC_{Ar}), 7.19–7.11 (m, 2 H, 2 × HC_{Ar}), 3.56 (ddt, *J* = 12.6, 4.1, 2.0 Hz, 1 H, NC*H*HCH_2), 3.41–3.33 (m, 2 H, C*H*HOH, NC*H*HCHAr), 3.26–3.13 (m, 3 H, NCH*H*CH_2, CH*H*OH and NCH*H*CHAr), 2.99–2.88 (m, 1 H, CHAr), 2.21 (dq, *J* = 14.5, 3.1 Hz, 1 H, NCH_2C*H*H), 2.05 (tdd, *J* = 11.7, 6.4, 3.2 Hz, 1 H, C*H*CH_2OH), 1.90–1.76 (m, 1 H, NCH_2CH*H*);

^{13}C NMR (101 MHz, CD_3OD) δ 164.6 (d, J_{CF} = 244.5 Hz, FC_{Ar} quat), 136.4 (d, J_{CF} = 3.2 Hz, F-*p*-C_{Ar} quat), 130.8 (d, J_{CF} = 8.0 Hz, 2 × F-*m*-C_{Ar}), 116.7 (d, J_{CF} = 21.4 Hz, 2 × F-*o*-C_{Ar}), 63.6 (CH_2OH), 50.0 (N*C*H_2CHAr), 45.2 (N*C*H_2CH_2), 42.7 (CHAr), 42.6 (*C*HCH_2OH), 26.9 (NCH_2*C*H_2);

^{19}F{^{1}H} NMR (376 MHz, CD_3OD) δ –116.85 (s);

HRMS (ESI) *m/z* Calculated for $C_{12}H_{17}NOF$ [M-Cl] 210.1294; Found 210.1303.

cis-(±)-3-(4-Methoxyphenyl)tetrahydro-2H-thiopyran-4-carboxylic acid 1,1-dioxide (FRAG46)

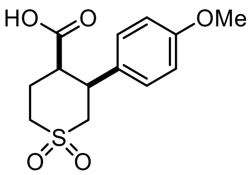

A flame-dried microwave vial (25 mL) was charged with *cis*-arylated **15a** (0.37 g, 0.90 mmol, 1.0 equiv), di-*tert*-butyl dicarbonate (Boc_2O, 981 mg, 4.50 mmol, 5.0 equiv) and 4-(dimethylamino)pyridine (DMAP, 22 mg, 0.18 mmol, 0.2 equiv). The reaction vessel was sealed and purged with argon, then MeCN (1.8 mL, 0.5 M) was added by syringe. The vial was placed in an oil bath and gradually warmed up to 70 °C, then stirred for 3 h at 70 °C. The mixture was allowed to cool down and sat. aq. NH_4Cl (10 mL) and CH_2Cl_2 (15 mL) were added. The phases were separated, and the aqueous layer was extracted with CH_2Cl_2 (3 × 10 mL). The combined organic extracts were dried over Na_2SO_4 and filtered and the solvent was removed under reduced pressure to afford the crude *N*-Boc protected amide derivative, which was carried forward to the hydrolysis step without further purification. A solution of H_2O_2 (30 wt.% in H_2O, 0.36 mL, 4.0 equiv) in THF (0.9 mL, 0.1 M) was added to a solution of $LiOH·H_2O$ (61 mg, 1.44 mmol, 1.6 equiv) in H_2O (1.5 mL, 0.1 M) at 0 °C under argon. The resulting mixture was added dropwise to a solution of the crude *N*-Boc protected amide in THF (1.8 mL, 0.5 M) at 0 °C under argon. The reaction was then stirred at 45 °C for 2 h. The reaction mixture was then allowed to cool down to rt and sat. aq. $Na_2S_2O_3$ (10 mL), EtOAc (10 mL) and 1 M NaOH (5 mL) were added. The aqueous alkaline layer was collected, and the organic layer extracted with 1 M NaOH (3 × 15 mL). All the aqueous alkaline layers were combined and acidified to pH ~ 1 with 1 M HCl, then extracted with EtOAc (3 × 50 mL). The combined organic extracts were dried over Na_2SO_4, filtered, and the solvent evaporated under reduced pressure to afford the desired acid **FRAG46** as a pale orange solid (221 mg, 0.43 mmol, 48% over 2 steps, 35% recovery of starting material **15a**).

R_f 0.27 (40% acetone/pentane);

mp = 255–257 °C (from Et_2O/pentane);

IR (film)/cm^{-1} 3220 (br OH), 2914, 2847, 1707 (C=O), 1513, 1416, 1252 (S=O), 1177 (S=O), 1111, 1013, 820;

^{1}H NMR (400 MHz, CD_3CN) δ 9.10 (br s, 1 H, COOH), 7.21–7.12 (m, 2 H, 2 × HC_{Ar}), 6.92–6.82 (m, 2 H, 2 × HC_{Ar}), 4.09 (t, *J* = 13.6 Hz, 1 H, SC*H*HCHAr), 3.76 (s, 3 H, OCH_3), 3.56 (dt, *J* = 13.4, 3.6 Hz, 1 H, CH(C=O)), 3.32 (ddd, *J* = 14.2, 13.1, 4.5 Hz, 1 H, SC*H*HCH_2), 3.04 (dt, *J* = 13.8, 3.6 Hz, 1 H, SCH*H*CHAr), 3.02–2.89 (m, 2 H, CHAr and SCH*H*CH_2), 2.48–2.28 (m, 2 H, $O_2SCH_2CH_2$);

^{13}C NMR (101 MHz, CD_3CN) δ 174.4 (C=O acid), 159.9 (C_{Ar} quat), 133.2 (C_{Ar} quat), 129.4 (2 × C_{Ar}), 114.8 (2 × C_{Ar}), 55.8 (OCH_3), 52.1 (S*C*H_2CHAr), 47.6 (S*C*H_2CH_2), 44.1 (CHAr), 42.5 (*C*H(C=O)), 27.0 (CH_2CH_2);

HRMS (ESI) *m/z* Calculated for $C_{13}H_{15}O_5S$ [M+H] 283.0640; Found 283.0649.

cis-(±)-4-(Hydroxymethyl)-3-(4-methoxyphenyl)tetrahydro-2H-thiopyran 1,1-dioxide (FRAG47)

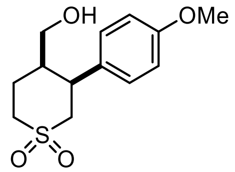

$BH_3{\cdot}SMe_2$ (70 µL, 0.77 mmol, 2.2 equiv) was added to a solution of *cis*-carboxylic acid **FRAG46** (95 mg, 0.35 mmol, 1.0 equiv) in THF (1.8 mL, 0.2 M) at 0 °C. The reaction mixture was stirred at 0 °C for 3 h, then at rt for 18 h. The mixture was then cooled again to 0 °C and quenched by dropwise addition of H_2O (3 mL). After dilution with EtOAc (10 mL), the layers were separated and the organic layer washed with sat. aq. NaCl (10 mL), $NaHCO_3$ (10 mL), H_2O (5 mL) and again sat. aq. NaCl (5 mL). The organic layer was dried over Na_2SO_4 and concentrated under reduced pressure. The crude product was purified by flash column chromatography (10-35% EtOAc/hexane) to afford the desired primary alcohol **FRAG47** as a white powder (61 mg, 0.23 mmol, 64%).

R_f 0.67 (100% EtOAc);

mp = 136–138 °C (from Et_2O/pentane);

IR (film)/cm^{-1} 3504 (br OH), 2937, 1513, 1305, 1245 (S=O), 1118 (S=O), 1029, 835;

^{1}H NMR (400 MHz, $CDCl_3$) δ 7.15–7.06 (m, 2 H, 2 × HC_{Ar}), 6.89 (d, *J* = 8.7 Hz, 2 H, 2 × HC_{Ar}), 3.82 (dd, *J* = 12.8, 12.7 Hz, 1 H, SC*H*HCHAr), 3.81 (s, 3 H, OCH_3), 3.73–3.65 (m, 2 H, C*H*HOH and CHAr), 3.62 (dt, *J* = 10.4, 3.4 Hz, 1 H, CH*H*OH), 3.50 (ddd, *J* = 14.1, 11.2, 6.7 Hz, 1 H, SC*H*HCH$_2$), 3.12–3.04 (m, 1 H, SCH*H*CHAr), 2.99 (dq, *J* = 14.1, 3.7 Hz, 1 H, SCH*H*CH$_2$), 2.48–2.40 (m, 2 H, SCH$_2$C*H$_2$*), 2.04 (dp, *J* = 7.8, 3.9 Hz, 1 H, C*H*CH$_2$OH), 1.40 (t, *J* = 3.7 Hz, 1 H, OH);

^{13}C NMR (101 MHz, $CDCl_3$) δ 158.7 (C_{Ar} quat), 132.2 (C_{Ar} quat), 128.2 (2 × C_{Ar}), 114.2 (2 × C_{Ar}), 61.6 (*C*H$_2$OH), 55.3 (OCH_3), 52.4 (S*C*H$_2$CHAr), 47.7 (S*C*H$_2$CH$_2$), 42.7 (CHAr), 39.0 (*C*HCH$_2$OH), 26.9 (SCH$_2$*C*H$_2$);

HRMS (ESI) *m/z* Calculated for $C_{15}H_{21}NO_4NaS$ [M+CH_3CN+Na] 334.1089; Found 334.1096.

cis-(±)-4,4-Difluoro-2-(4-methoxyphenyl)cyclohexane-1-carboxylic acid (FRAG48)

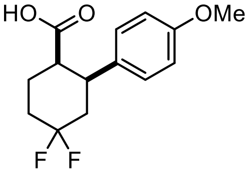

A flame-dried microwave vial (25 mL) was charged with *cis*-arylated **17a** (0.40 g, 1.00 mmol, 1.0 equiv), di-*tert*-butyl dicarbonate (Boc$_2$O, 1.10 g, 5.00 mmol 5.0 equiv) and 4-(dimethylamino)pyridine (DMAP, 25 mg, 0.20 mmol, 0.2 equiv). The reaction vessel was sealed and purged with argon, then MeCN (2.0 mL, 0.5 M) was added by syringe. The vial was placed in an oil bath and gradually warmed up to 70 ºC, then stirred for 3 h at 70 °C. The mixture was allowed to cool down and sat. aq. NH_4Cl (10 mL) and CH_2Cl_2 (15 mL) were added. The phases were separated, and the aqueous layer was extracted with CH_2Cl_2 (3 × 10 mL). The combined organic extracts were dried over Na_2SO_4 and filtered and the solvent was removed under reduced pressure to afford the crude *N*-Boc protected amide derivative, which was carried forward to the hydrolysis step without further purification. A solution of H_2O_2 (30 wt.% in H_2O, 0.40 mL, 4.0 equiv) in THF (1.1 mL, 0.1 M)

was added to a solution of $LiOH \cdot H_2O$ (68 mg, 1.60 mmol, 1.6 equiv) in H_2O (1.6 mL, 0.1 M) at 0 °C under argon. The resulting mixture was added dropwise to a solution of the crude *N*-Boc protected amide in THF (2.0 mL, 0.5 M) at 0 °C under argon. The reaction was then stirred at 45 °C for 2 h. The reaction mixture was then allowed to cool down to rt and sat. aq. $Na_2S_2O_3$ (10 mL), EtOAc (10 mL) and 1 M NaOH (5 mL) were added. The aqueous alkaline layer was collected, and the organic layer extracted with 1 M NaOH (3 × 15 mL). All the aqueous alkaline layers were combined and acidified to pH ~ 1 with 1 M HCl, then extracted with EtOAc (3 × 50 mL). The combined organic extracts were dried over Na_2SO_4, filtered, and the solvent evaporated under reduced pressure to afford the desired acid **FRAG48** as a yellow solid (54 mg, 0.20 mmol, 20% over 2 steps).

R_f 0.30 (15% acetone/pentane);

mp = 127–129 °C (from Et_2O/pentane);

IR (film)/cm^{-1} 2945 (br OH), 1707 (C=O), 1513, 1453, 1245, 1103, 1036, 969;

^{1}H NMR (400 MHz, $CDCl_3$) δ 7.17–7.09 (m, 2 H, 2 × HC_{Ar}), 6.89–6.80 (m, 2 H, 2 × HC_{Ar}), 3.80 (s, 3 H, OCH_3), 3.18 (dt, *J* = 13.7, 4.7 Hz, 1 H, CHAr), 2.98 (d, *J* = 4.6 Hz, 1 H, CH(C=O)), 2.81 (dtd, *J* = 33.9, 13.5, 3.9 Hz, 1 H, F_2CC*H*HCHAr), 2.26–2.15 (m, 2 H, F_2CC*H*HCH_2 and F_2CCH*H*CHAr), 2.23–1.98 (ddt, *J* = 17.9, 6.9, 3.2 Hz, 3 H, F_2CCH*H*CH_2 and F_2CCH_2C*H_2*);

^{13}C NMR (101 MHz, $CDCl_3$) δ 178.7 (C=O acid), 158.6 (C_{Ar} quat), 133.2 (C_{Ar} quat), 128.2 (2 × C_{Ar}), 122.1 (t, J_{CF} = 241.5 Hz, CF_2), 114.1 (2 × C_{Ar}), 55.2 (OCH_3), 48.4 (*C*H(C=O)), 41.9 (d, J_{CF} = 10.1 Hz, CHAr), 40.9 (t, J_{CF} = 24.3 Hz, F_2C*C*H_2CHAr), 32.7 (t, J_{CF} = 25.2 Hz, F_2C*C*H_2CH_2), 26.2 (d, J_{CF} = 10.1 Hz, F_2CCH_2*C*H_2);

^{19}F{^{1}H} NMR (376 MHz, $CDCl_3$) δ –88.66 (d, J_{FF} = 238.0 Hz), –100.84 (d, J_{FF} = 237.8 Hz);

HRMS (ESI) *m/z* Calculated for $C_{14}H_{15}O_3F_2$ [M+H] 269.0989; Found 269.0994.

trans-(±)-4,4-Difluoro-2-(4-methoxyphenyl)cyclohexane-1-carboxylic acid (FRAG49)

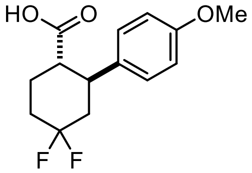

A flame-dried microwave vial (25 mL) was charged with *trans*-arylated **18a** (160 mg, 0.40 mmol, 1.0 equiv), di-*tert*-butyl dicarbonate (Boc_2O, 0.44 g, 2.00 mmol, 5.0 equiv) and 4-(dimethylamino)pyridine (DMAP, 10 mg, 0.08 mmol, 0.2 equiv). The reaction vessel was sealed and purged with argon, then MeCN (0.8 mL, 0.5 M) was added by syringe. The vial was placed in an oil bath and gradually warmed up to 70 °C, then stirred for 3 h at 70 °C. The mixture was allowed to cool down and sat. aq. NH_4Cl (10 mL) and CH_2Cl_2 (15 mL) were added. The phases were separated, and the aqueous layer was extracted with CH_2Cl_2 (3 × 10 mL). The combined organic extracts were dried over Na_2SO_4 and filtered and the solvent was removed under reduced pressure to afford the crude *N*-Boc protected amide derivative, which was carried forward to the hydrolysis step without further purification. A solution of H_2O_2 (30 wt.% in H_2O, 0.16 mL, 4.0 equiv) in THF (0.4 mL, 0.1 M) was added to a solution of $LiOH \cdot H_2O$ (27 mg, 0.64 mmol, 1.6 equiv) in H_2O (0.7 mL, 0.1 M) at 0 °C under argon. The resulting mixture was added dropwise to a solution of the crude *N*-Boc protected amide in THF (0.8 mL, 0.5 M) at 0 °C under argon. The reaction was then stirred at 45 °C for 2 h. The reaction mixture was then allowed to cool down to rt and sat. aq. $Na_2S_2O_3$ (10 mL), EtOAc (10 mL) and 1 M NaOH (5 mL) were added. The aqueous alkaline layer was collected, and the organic layer extracted with 1 M NaOH (3 × 15 mL). All the

aqueous alkaline layers were combined and acidified to pH ~ 1 with 1 M HCl, then extracted with EtOAc (3 × 50 mL). The combined organic extracts were dried over Na_2SO_4, filtered, and the solvent evaporated under reduced pressure to afford the desired acid **FRAG49** as a yellow solid (51 mg, 0.19 mmol, 47% over 2 steps).

R_f 0.37 (20% acetone/pentane);

mp = 134–136 °C (from Et_2O/pentane);

IR (film)/cm^{-1} 2967 (br OH), 1707 (C=O), 1513, 1461, 1252, 1174, 1036, 962, 835;

^{1}H NMR (400 MHz, $CDCl_3$) δ 7.16–7.06 (m, 2 H, 2 × HC_{Ar}), 6.89–6.78 (m, 2 H, 2 × HC_{Ar}), 3.79 (s, 3 H, OCH_3), 3.19–3.03 (td, *J* = 12.5, 3.6 Hz, 1 H, CHAr), 2.57 (td, *J* = 12.0, 3.0 Hz, 1 H, CH(C=O)), 2.34–2.20 (m, 2 H, F_2CC*H*HCHAr and F_2CC*H*HCH_2), 2.18–2.08 (m, 1 H, F_2CCH_2C*H*H), 1.99–1.73 (m, 3 H, F_2CCH*H*CH_2, F_2CCH*H*CHAr and F_2CCH_2CH*H*);

^{13}C NMR (101 MHz, $CDCl_3$) δ 178.7 (C=O acid), 158.6 (C_{Ar} quat), 133.2 (C_{Ar} quat), 128.2 (2 × C_{Ar}), 122.1 (t, J_{CF} = 241.5 Hz, CF_2), 114.1 (2 × C_{Ar}), 55.2 (OCH_3), 48.4 (*C*H(C=O)), 41.9 (d, J_{CF} = 10.6 Hz, CHAr), 40.9 (t, J_{CF} = 23.5 Hz, F_2C*C*H_2CHAr), 32.7 (t, J_{CF} = 24.9 Hz, F_2C*C*H_2CH_2), 26.2 (d, J_{CF} = 10.2 Hz, F_2CCH_2*C*H_2);

^{19}F{^{1}H} NMR (376 MHz, $CDCl_3$) δ –90.97 (d, J_{FF} = 239.2 Hz), –101.44 (J_{FF} = 237.5 Hz);

HRMS (APCI) *m/z* Calculated for $C_{14}H_{15}O_2F_2$ [M+H] 269.0995; Found 269.0996.

cis-(±)-4,4-Difluoro-2-(4-methoxyphenyl)cyclohexyl)methanol (FRAG50)

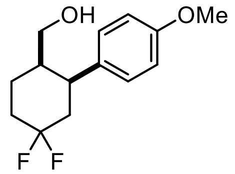

A flame-dried microwave vial (25 mL) was charged with the corresponding *cis*-arylated **17a** (396 mg, 1.00 mmol, 1.0 equiv), di-*tert*-butyl dicarbonate (Boc_2O, 1.10 g, 5.00 mmol, 5.0 equiv) and 4-(dimethylamino)pyridine (DMAP, 25 mg, 0.20 mmol, 0.2 equiv). The reaction vessel was sealed and purged with argon, then MeCN (2.0 mL, 0.5 M) was added by syringe. The vial was placed in an oil bath and gradually warmed up to 70 °C, then stirred for 3 h at 70 °C. The mixture was allowed to cool down and sat. aq. NH_4Cl (10 mL) and CH_2Cl_2 (15 mL) were added. The phases were separated, and the aqueous layer was extracted with CH_2Cl_2 (3 × 10 mL). The combined organic extracts were dried over Na_2SO_4 and filtered. The solvent was removed under reduced pressure to afford the crude *N*-Boc protected amide derivative which was carried forward to the reductive cleavage step without further purification.

A solution of the crude *N*-Boc-protected amide in dry THF (5 mL, 0.2 M) was added dropwise to a suspension of $LiAlH_4$ (156 mg, 4.00 mmol, 4.0 equiv) in dry THF (2 mL, 0.5 M) at 0 °C under argon atmosphere. The mixture was then stirred at 25 °C for 1 h. The reaction mixture was then quenched by slow addition of sat. aq. NH_4Cl (3 mL) at 0 °C and stirred at rt for 30 min. The suspension was filtered through a pad of Celite, eluting with EtOAc (3 × 5 mL). The phases were separated and the aqueous layer extracted with EtOAc (3 × 10 mL). The combined organic extracts were dried over Na_2SO_4 and filtered. The solvent was removed under reduced pressure and the crude residue purified by flash column chromatography (20–40% EtOAc/hexane) to afford the desired alcohol **FRAG50** as a yellow oil (69 mg, 0.27 mmol, 27% over 2 steps).

R_f 0.30 (30% EtOAc/hexane);

IR (film)/cm1 3370 (br OH), 2937, 1513, 1461, 1372, 1245 (S=O), 1103, 1005, 969;

^{1}H NMR (400 MHz, $CDCl_3$) δ 7.16–7.06 (m, 2 H, 2 × HC_{Ar}), 6.94–6.82 (m, 2 H, 2 × HC_{Ar}), 3.81 (s, 3 H, OCH_3), 3.54 (ddd, *J* = 10.8, 8.7, 6.3 Hz, 1 H, C*H*HOH), 3.46 (dt, *J* = 10.8, 4.1 Hz, 1 H, CH*H*OH), 3.25 (dd, *J* = 11.6, 5.6 Hz, 1 H, CHAr), 2.30–2.20 (m, 1 H, F_2CC*H*HCHAr), 2.20–2.10 (m, 3 H, C*H*CH_2OH, F_2CCH*H*CHAr and F_2CCH_2C*H*H), 2.07–1.99 (m, 1 H, F_2CC*H*HCH_2), 1.92–1.88 (m, 2 H, F_2CC*H*HCH_2 and F_2CCH_2CH*H*), 0.93 (dd, *J* = 6.5, 4.0 Hz, 1 H, OH);

^{13}C NMR (101 MHz, $CDCl_3$) δ 158.3 (C_{Ar} quat), 133.7 (C_{Ar} quat), 128.0 (2 × C_{Ar}), 124.0 (t, J_{CF} = 246.0 Hz, CF_2), 114.0 (2 × C_{Ar}), 59.6 (CH_2OH), 55.3 (OCH_3), 40.9 (*C*HCH_2OH), 40.4 (d, J_{CF} = 9.5 Hz, CHAr), 34.2 (dd, J_{CF} = 25.2, 22.8 Hz, F_2C*C*H_2CHAr), 29.1 (dd, J_{CF} = 25.2, 22.8 Hz, F_2C*C*H_2CH_2), 24.0 (d, J_{CF} = 9.5 Hz, F_2CCH_2*C*H_2);

^{19}F NMR (376 MHz, $CDCl_3$, 298 K) δ –87.13 - –87.79 (m), –99.75- –100.62 (m);

HRMS (APCI) *m/z* Calculated for $C_{14}H_{18}O_2F_2^+$ $[M+H]^+$ 256.1269; Found 256.1261.

trans-(±)-4,4-Difluoro-2-(4-methoxyphenyl)cyclohexyl)methanol (FRAG51)

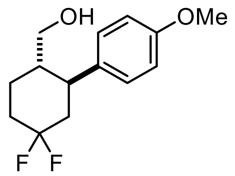

A flame-dried microwave vial (25 mL) was charged with the corresponding *trans*-arylated **18a** (300 mg, 0.75 mmol, 1.0 equiv), di-*tert*-butyl dicarbonate (Boc_2O, 817 mg, 375 mmol, 5.0 equiv) and 4-(dimethylamino)pyridine (DMAP, 19 mg, 0.15 mmol, 0.2 equiv). The reaction vessel was sealed and purged with argon, then MeCN (2.0 mL, 0.5 M) was added by syringe. The vial was placed in an oil bath and gradually warmed up to 70 ºC, then stirred for 3 h at 70 °C. The mixture was allowed to cool down and sat. aq. NH_4Cl (10 mL) and CH_2Cl_2 (15 mL) were added. The phases were separated, and the aqueous layer was extracted with CH_2Cl_2 (3 × 10 mL). The combined organic extracts were dried over Na_2SO_4 and filtered. The solvent was removed under reduced pressure to afford the crude *N*-Boc protected amide derivative which was carried forward to the reductive cleavage step without further purification.

A solution of the crude *N*-Boc-protected amide in dry THF (3.8 mL, 0.2 M) was added dropwise to a suspension of $LiAlH_4$ (117 mg, 3.00 mmol, 4.0 equiv) in dry THF (1.5 mL, 0.5 M) at 0 °C under argon atmosphere. The mixture was then stirred at 25 °C for 1 h. The reaction mixture was then quenched by slow addition of sat. aq. NH_4Cl (3 mL) at 0 °C and stirred at rt for 30 min. The suspension was filtered through a pad of Celite, eluting with EtOAc (3 × 5 mL). The phases were separated and the aqueous layer extracted with EtOAc (3 × 10 mL). The combined organic extracts were dried over Na_2SO_4 and filtered. The solvent was removed under reduced pressure and the crude residue purified by flash column chromatography (20–40% EtOAc/hexane) to afford the desired alcohol **FRAG51** as an orange oil (63 mg, 0.25 mmol, 33% over 2 steps).

R_f 0.35 (30% EtOAc/Hexane);

IR (film)/cm^{-1} 3360 (OH), 2937, 1513, 1446, 1372, 1238, 1074, 962;

^{1}H NMR (400 MHz, $CDCl_3$) δ 7.17–7.06 (m, 2 H, 2 × HC_{Ar}), 6.93–6.80 (m, 2 H, 2 × HC_{Ar}), 3.80 (s, 3 H, OCH_3), 3.43 (dd, *J* = 10.8, 3.7 Hz, 1 H, C*H*HOH), 3.24 (dd, *J* = 10.8, 6.4 Hz, 1 H, CH*H*OH), 2.68 (ddd, *J* = 13.0, 11.2, 4.0 Hz, 1 H, CHAr), 2.31–2.14 (m, 2 H, F_2CC*H*HCHAr and F_2CC*H*HCH_2), 2.12–2.01 (m, 1 H, F_2CCH_2C*H*H), 1.98–1.75 (m, 2 H, F_2CCH*H*CHAr and F_2CCH*H*CH_2), 1.76–1.66 (m, 1 H, C*H*CH_2OH), 1.59 (ddd, *J* = 13.6, 12.3, 3.9 Hz, 1 H, F_2CCH_2CH*H*), 1.21 (s, 1 H, OH);

^{13}C NMR (101 MHz, $CDCl_3$) δ 158.5 (C_{Ar} quat), 134.5 (C_{Ar} quat), 128.2 (2 × C_{Ar}),123.2 (dd, J_{CF} = 243.0, 239.1 Hz, CF_2), 114.2 (2 × C_{Ar}), 65.0 (CH_2OH), 55.2 (OCH_3), 43.9 ($\mathit{C}HCH_2OH$), 42.4 (d, J_{CF} = 10.6 Hz, CHAr), 41.8 (dd, J_{CF} = 25.4, 22.2 Hz, $F_2C\mathit{C}H_2CHAr$), 33.4 (dd, J_{CF} = 25.4, 22.2 Hz, $F_2C\mathit{C}H_2CH_2$), 25.8 (d, J_{CF} = 10.6 Hz, $F_2CCH_2\mathit{C}H_2$);

^{19}F NMR (376 MHz, $CDCl_3$) δ –90.09 - –95.75 (m), –101.19 - –102.06 (m);

HRMS (APCI) *m/z* Calculated for $C_{14}H_{18}O_2F_2^+$ $[M+H]^+$ 256.1269; Found 256.1269.

Section 5: Medicinal Chemistry Analysis

All analyses were performed in **Python 3.10** using: RDKit, NumPy, pandas, matplotlib, seaborn, and umap-learn packages.

Data Preparation

- An initial set of 44 fragment SMILES (containing absolute stereochemical information) was parsed
- To reflect the fact that the actual experimental materials were racemic mixtures, the stereochemistry was inverted: for each chiral atom annotated with CIP tags (R/S), the chiral tag was switched (R → S, S → R). Both the original and inverted enantiomer SMILES were retained, doubling the fragment set (to a total of 88 SMILES) to give the **Synthesised Fragments Collection**.

Physicochemical Property Calculations

For each molecule, the following descriptors were calculated in RDKit:

- Molecular weight (MW)
- logP (octanol-water partition coefficient, Crippen)
- Number of hydrogen bond donors (HBD)
- Number of hydrogen bond acceptors (HBA)
- Topological polar surface area (TPSA)
- Number of rotatable bonds (RotBonds)

For each descriptor, we calculated the average value across all fragments. The averages were normalised by dividing by the Rule of 3 (Ro3) thresholds (i.e. avg / limit) to allow comparison across descriptors of different scales. These normalised values were visualised on a radar (spider) plot, displaying how close the fragment set lies to the Ro3 boundaries.

Comparison with Reference Libraries

Two commercial fragment libraries were used for benchmarking:

- **ZINC20 fragments** (https://zinc.docking.org/)
- **Enamine Fragment Collection** (version 13 April 2023, ~259,380 in-stock fragments; https://enamine.net/compound-collections/fragment-collection)

To ensure a meaningful comparison with our synthetic series, commercial sets were filtered using SMARTS patterns (inserted into the user-friendly on-line interface) representing heterocyclic and saturated ring motifs closely related to our targeted scaffolds:

- piperidine (N1CCCC1)
- bicyclic amine (C1CNCCC1)
- cyclohexane sulfone (O=S1(CCCCC1)=O)
- difluoro-cyclohexane (FC1(F)CCCCC1).

Molecules matching any of these patterns were retained, yielding reference libraries enriched in scaffolds structurally analogous to those explored in our synthesis.

Dimensionality Reduction and Embedding

Fingerprint vectors from the three datasets (Enamine, ZINC, and synthesised fragments) were concatenated and embedded into two dimensions using **UMAP (Uniform Manifold Approximation and Projection; n_components = 2, random_state = 42)**. All other hyperparameters (**n_neighbors=15, min_dist=0.1, metric='euclidean'**) were left at their defaults. The resulting 2D embedding was visualised with **Seaborn (v0.12.2)**.

Code

Code and inputs available on GitHub:

https://github.com/SofiaPiticari/Fragment-analysis-pipeline-drug-discovery-

SMILES input for synthesised fragments

NC([C@H]1NCC[C@H]1C2=CC=C(OC)C=C2)=O

NC([C@H]1NCC[C@H]1C2=CC=C(F)C=C2)=O

OC([C@H]1NCC[C@H]1C2=CC=C(OC)C=C2)=O

OC([C@H]1NCC[C@H]1C2=CC=C(F)C=C2)=O

OC[C@H]1NCC[C@H]1C2=CC=C(OC)C=C2

OC[C@H]1NCC[C@H]1C2=CC=C(F)C=C2

NC([C@H]1NCCC[C@H]1C2=CC=C(OC)C=C2)=O

O=C(N)[C@@H]1CNC[C@H]1C2=CC=C(OC)C=C2

O=C(O)[C@H]1CNC[C@H]1C2=CC=C(OC)C=C2

O=C(O)[C@@H]1CNC[C@H]1C2=CC=C(OC)C=C2

OC[C@H]1CNC[C@H]1C2=CC=C(OC)C=C2

OC[C@@H]1CNC[C@H]1C2=CC=C(OC)C=C2

O=C(N)[C@H]1CNC[C@H]1C2=CC=C(F)C=C2

O=C(N)[C@@H]1CNC[C@H]1C2=CC=C(F)C=C2

O=C(O)[C@H]1CNC[C@H]1C2=CC=C(F)C=C2

O=C(O)[C@@H]1CNC[C@H]1C2=CC=C(F)C=C2

OC[C@H]1CNC[C@H]1C2=CC=C(F)C=C2

OC[C@@H]1CNC[C@H]1C2=CC=C(F)C=C2

NC([C@@H]1CNCC[C@H]1C2=CC=C(F)C=C2)=O

OC([C@H]1CNCC[C@H]1C2=CC=C(OC)C=C2)=O

OC([C@@H]1CNCC[C@H]1C2=CC=C(OC)C=C2)=O

OC([C@@H]1CNCC[C@H]1C2=CC=C(F)C=C2)=O

OC[C@H]1CNCC[C@H]1C2=CC=C(OC)C=C2

OC[C@@H]1CNCC[C@H]1C2=CC=C(OC)C=C2

NC([C@H]1[C@@H](C2=CC=C(OC)C=C2)CNCC1)=O

NC([C@@H]1[C@@H](C2=CC=C(OC)C=C2)CNCC1)=O

OC([C@H]1[C@@H](C2=CC=C(OC)C=C2)CNCC1)=O

OC([C@@H]1[C@@H](C2=CC=C(OC)C=C2)CNCC1)=O

OC[C@H]1[C@@H](C2=CC=C(OC)C=C2)CNCC1

OC[C@@H]1[C@@H](C2=CC=C(OC)C=C2)CNCC1

NC([C@H]1[C@@H](C2=CC=C(F)C=C2)CNCC1)=O

NC([C@@H]1[C@@H](C2=CC=C(F)C=C2)CNCC1)=O

OC([C@H]1[C@@H](C2=CC=C(F)C=C2)CNCC1)=O

OC([C@@H]1[C@@H](C2=CC=C(F)C=C2)CNCC1)=O

OC[C@H]1[C@@H](C2=CC=C(F)C=C2)CNCC1

OC[C@@H]1[C@@H](C2=CC=C(F)C=C2)CNCC1

N#C[C@H]1[C@@H](C2=CC=C(OC)C=C2)CNCC1

OC([C@H]1[C@@H](C2=CC=C(OC)C=C2)CC(F)(F)CC1)=O

OC([C@@H]1[C@@H](C2=CC=C(OC)C=C2)CC(F)(F)CC1)=O

OC[C@H]1[C@@H](C2=CC=C(OC)C=C2)CC(F)(F)CC1

OC[C@@H]1[C@@H](C2=CC=C(OC)C=C2)CC(F)(F)CC1

FC(C=C1)=CC=C1[C@H]2CNCC[C@H]2C#N

OC([C@H](CC1)[C@@H](C2=CC=C(OC)C=C2)CS1(=O)=O)=O

OC[C@H](CC1)[C@@H](C2=CC=C(OC)C=C2)CS1(=O)=O

References

[23] a) D. P. Affron, O. A. Davis, J. A. Bull, "Regio- and Stereospecific Synthesis of C-3 Functionalized Proline Derivatives by Palladium Catalyzed Directed C(sp^3)–H Arylation" *Org. Lett.* **2014**, *16*, 4956–4959. b) D. P. Affron, J. A. Bull, "Palladium-Catalyzed Directed C(sp^3)-H Arylation of Saturated Heterocycles at C-3 Using a Concise Optimization Approach" *Eur. J. Org. Chem.* **2016**, *2016*, 139–149.

[24] a) D. Antermite, D. P. Affron, J. A. Bull, "Regio- and Stereoselective Palladium-Catalyzed C(sp^3)-H Arylation of Pyrrolidines and Piperidines with C(3) Directing Groups" *Org. Lett.* **2018**, *20*, 3948–3952. b) J. A. Coleman, V. Navratna, D. Antermite, D. Yang, J. A. Bull, E. Gouaux, "Chemical and structural investigation of the paroxetine-human serotonin transporter complex" *eLife* **2020**, *9*, e56427. c) D. Antermite, A. J. P. White, L. Cararrubios, J. A. Bull, "On the Mechanism and Selectivity of Palladium-Catalyzed C(sp^3)–H Arylation of Pyrrolidines and Piperidines at Unactivated C4 Positions: Discovery of an Improved Dimethylaminoquinoline Amide Directing Group" *ACS Catal.* **2023**, *13,* 9597–9615.

[25] Piticari, A.-S.; Antermite, D.; Higham, J. I.; Moore, J. H.; Webster, M. P.; Bull, J. A. Stereoselective Palladium-Catalyzed C(sp^3)–H Mono-Arylation of Piperidines and Tetrahydropyrans with a C(4) Directing Group. *Adv. Synth. Catal.* **2022**, *364*, 1488–1497.

[28] (a) Z. Zhang, X. Li, M. Song, Y. Wan, D. Zheng, G. Zhang, G. Chen, "Selective Removal of Aminoquinoline Auxiliary by IBX Oxidation" *J. Org. Chem.* **2019**, *84*, 12792–12799.(b) M. Berger, R. Chauhan, C. A. B. Rodrigues, N. Maulide, "Bridging C−H Activation: Mild and Versatile Cleavage of the 8-Aminoquinoline Directing Group" *Chem. Eur. J.* **2016**, *22*, 16805–16808. (c) M. Shang, K. S. Feu, J. C. Vantourout, L. M. Barton, H. L. Osswald, N. Kato, K. Gagaring, C. W. McNamara, G. Chen, L. Hu, S. Ni, P. Fernández-Canelas, M. Chen, R. R. Merchant, T. Qin, S. L. Schreiber, B. Melillo, J.-Q. Yu, P. S. Baran, "Modular, stereocontrolled $C_β$ –H/$C_α$ –C activation of alkyl carboxylic acids" *Proc. Natl. Acad. Sci. U.S.A.* **2019**, *116*, 8721–8727. (d) G. He, S. Zhang, W. A. Nack, Q. Li, G. Chen, "Use of a Readily Removable Auxiliary Group for the Synthesis of Pyrrolidones by the Palladium-Catalyzed Intramolecular Amination of Unactivated γ C(sp^3)–H Bonds" *Angew. Chem. Int. Ed.* **2013**, *52*, 11124–11128.

[31] D. A. Evans, P. H. Carter, C. J. Dinsmore, J. C. Barrow, J. L. Katz, D. W. Kung, "Mild nitrosation and hydrolysis of polyfunctional amides" *Tetrahedron Lett.* **1997**, *38*, 4535–4538.

[35] H. Okabe, A. Naraoka, T. Isogawa, S. Oishi, H. Naka, "Acceptor-Controlled Transfer Dehydration of Amides to Nitriles" *Org. Lett.* **2019**, *21*, 4767–4770.

[40] K. C. Nicolaou, P. S. Baran, Y.-L. Zhong, K. Sugita, "Iodine(V) Reagents in Organic Synthesis. Part 1. Synthesis of Polycyclic Heterocycles via Dess−Martin Periodinane-Mediated Cascade Cyclization: Generality, Scope, and Mechanism of the Reaction" *J. Am. Chem. Soc.* **2002**, 124, 2212-2220.

[41] M. F. Jung, M. A. Lyster, "Quantitative dealkylation of alkyl esters via treatment with trimethylsilyl iodide. A new method for ester hydrolysis" *J. Am. Chem. Soc.* **1977**, 99, 968-969.

^{1}H, ^{13}C and ^{19}F NMR Spectra

N-(Quinolin-8-yl)tetrahydro-2H-thiopyran-4-carboxamide 1,1-dioxide (14)

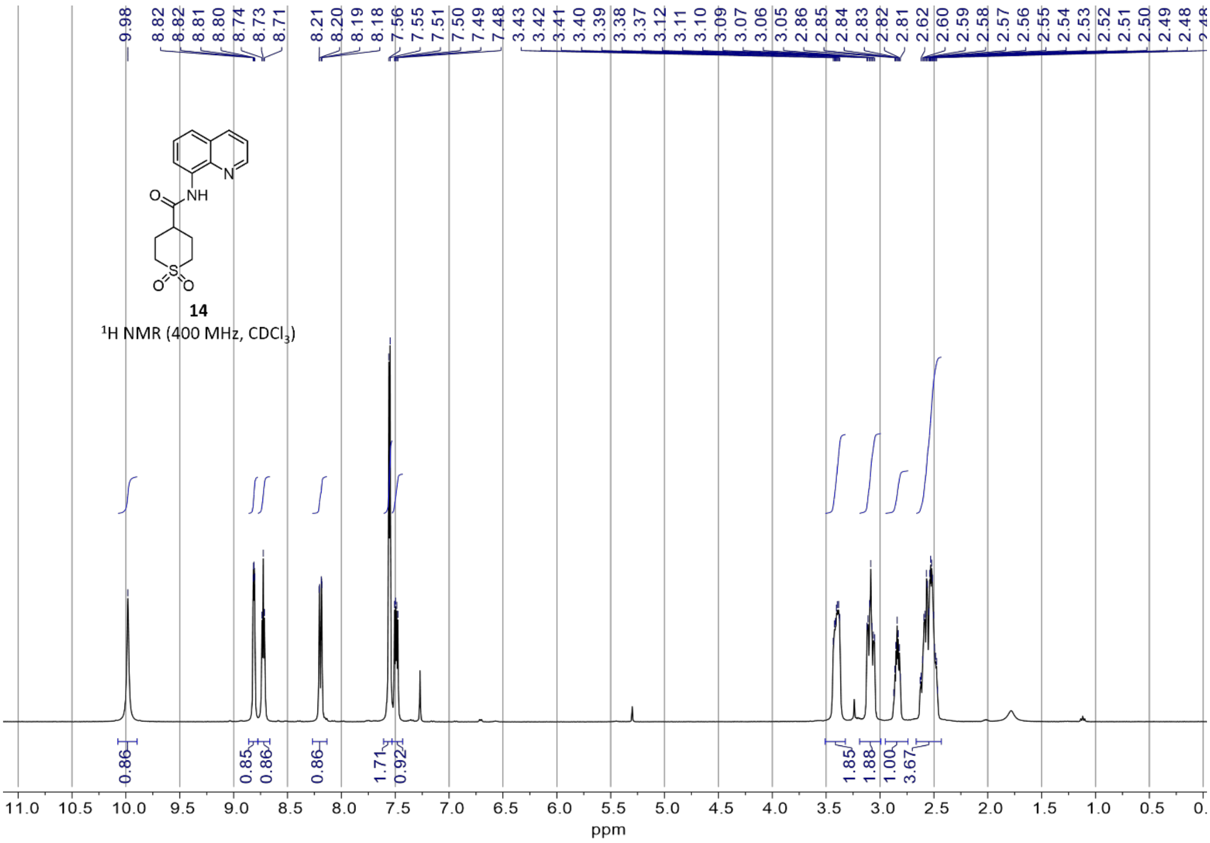

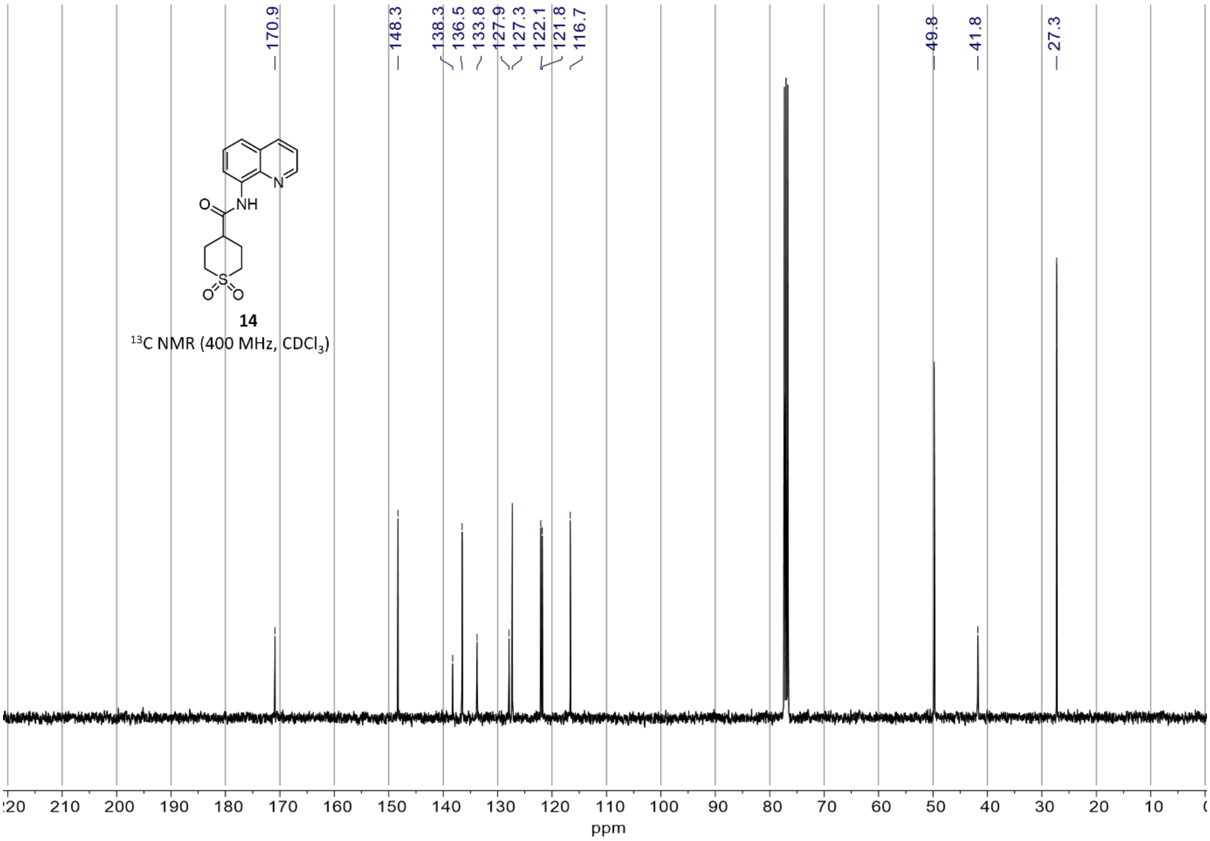

4,4-Difluoro-N-(quinolin-8-yl)cyclohexane-1-carboxamide (16)

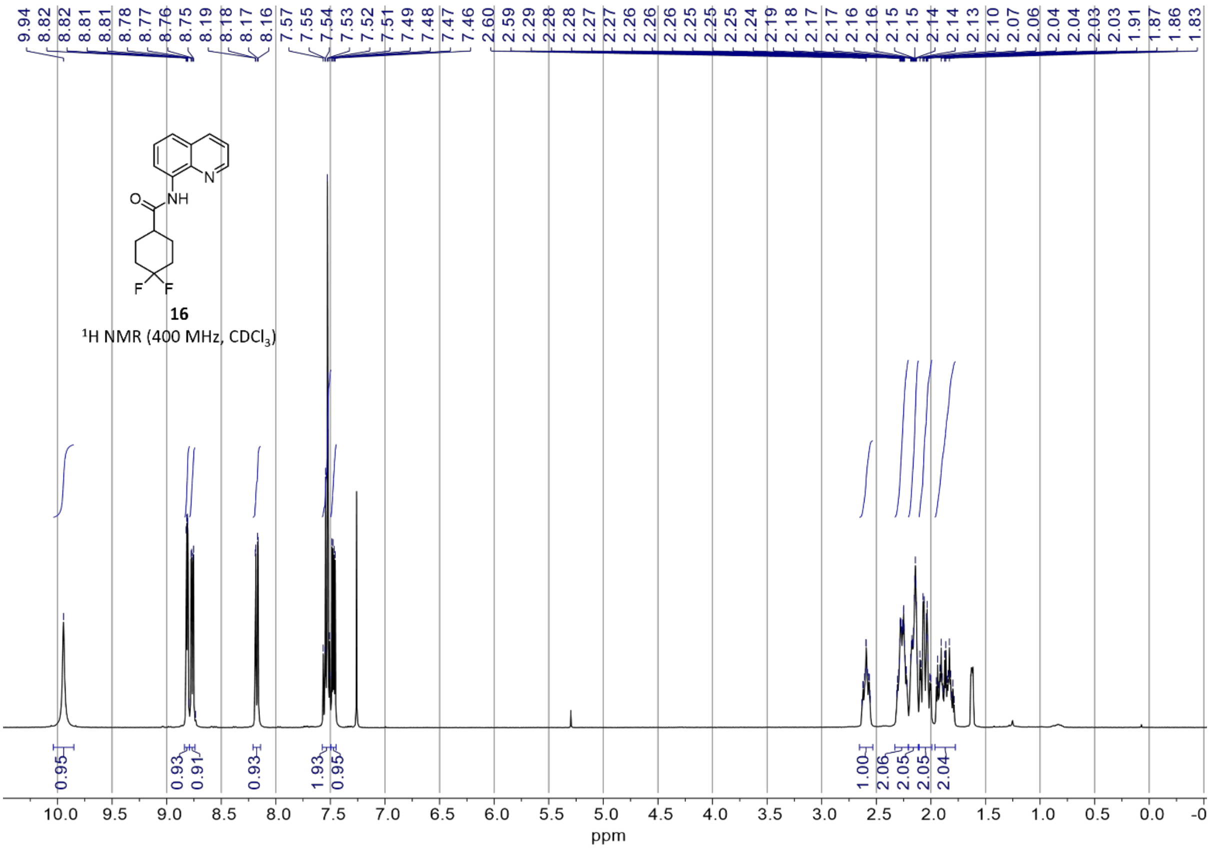

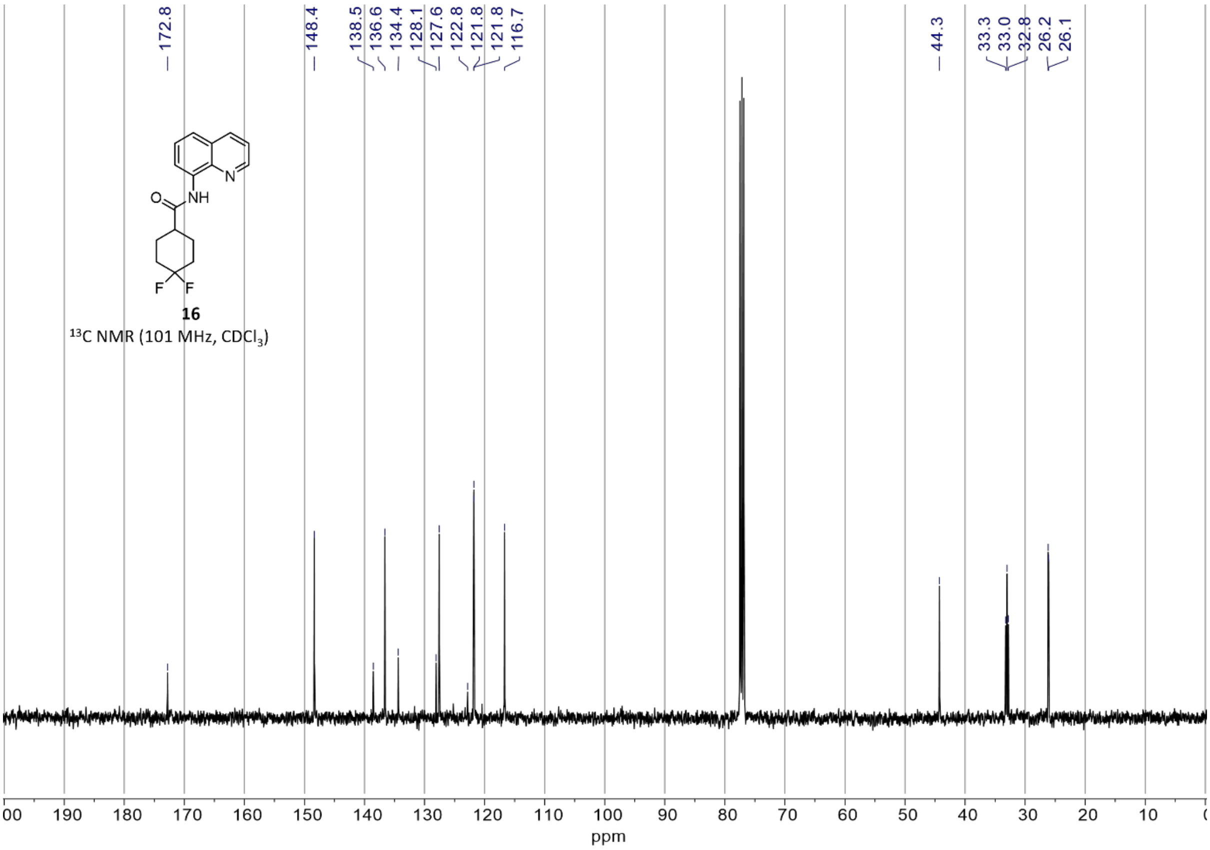

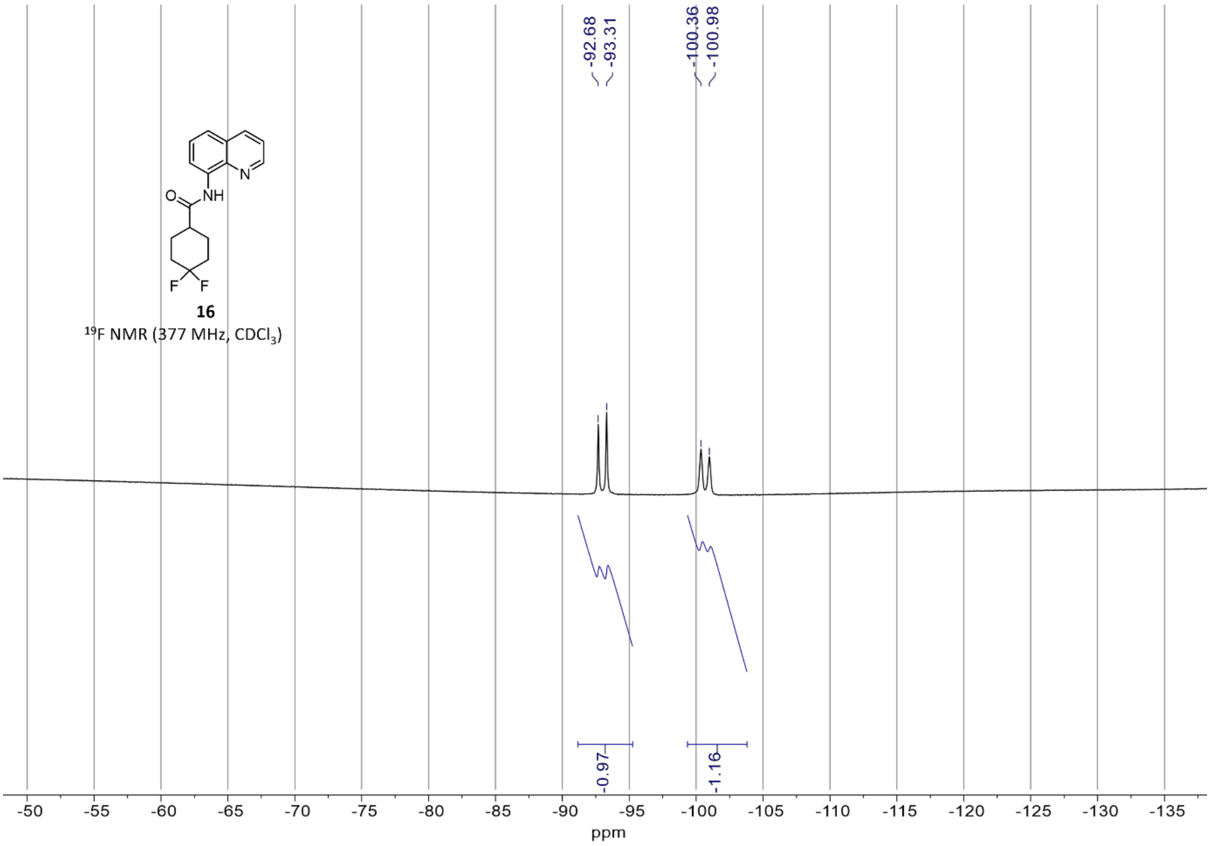

-92.68
-93.31
-100.36
-100.98
NH
O
N
F
F
16
^{19}F NMR (377 MHz, $CDCl_3$)
0.97
1.16
-50 -55 -60 -65 -70 -75 -80 -85 -90 -95 -100 -105 -110 -115 -120 -125 -130 -135
ppm

***cis*-(±)-Benzyl 3-(4-methoxyphenyl)-2-(quinolin-8-ylcarbamoyl)pyrrolidine-1-carboxylate (2a)**

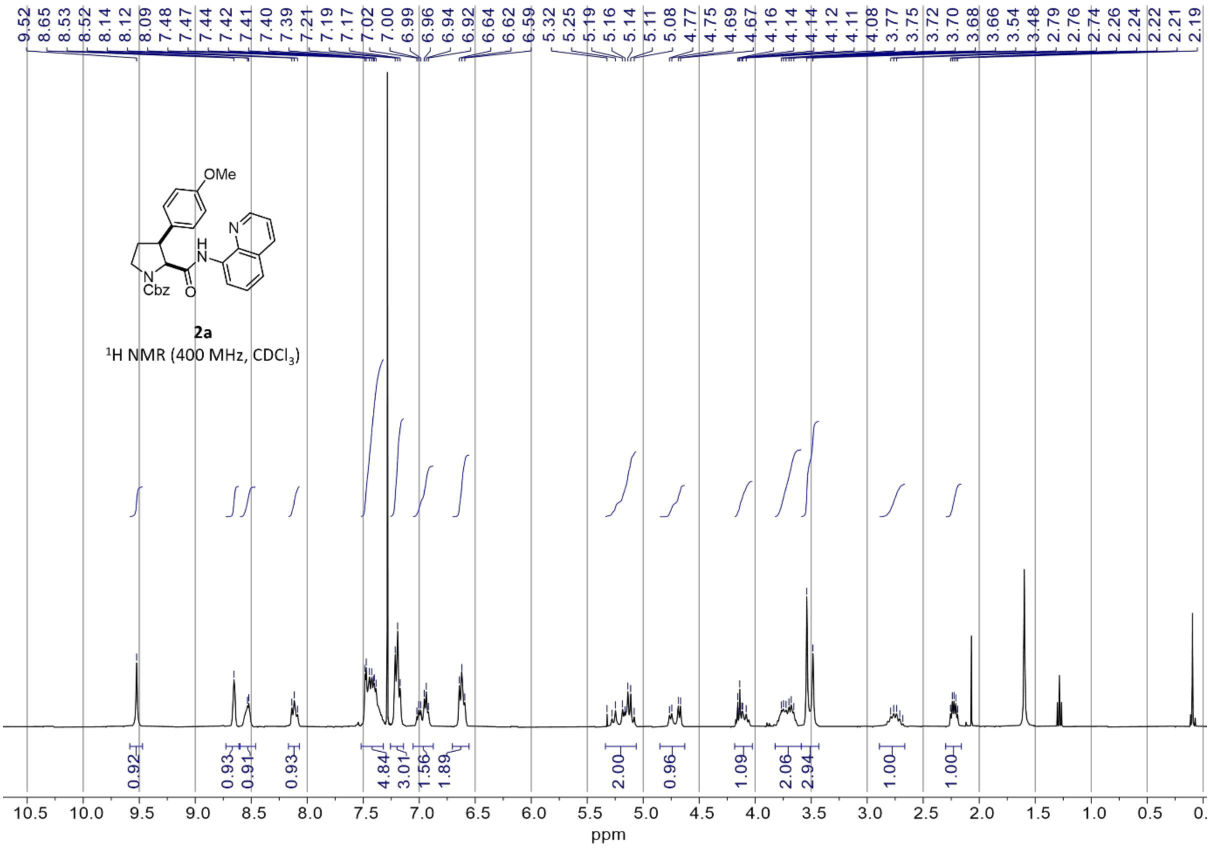

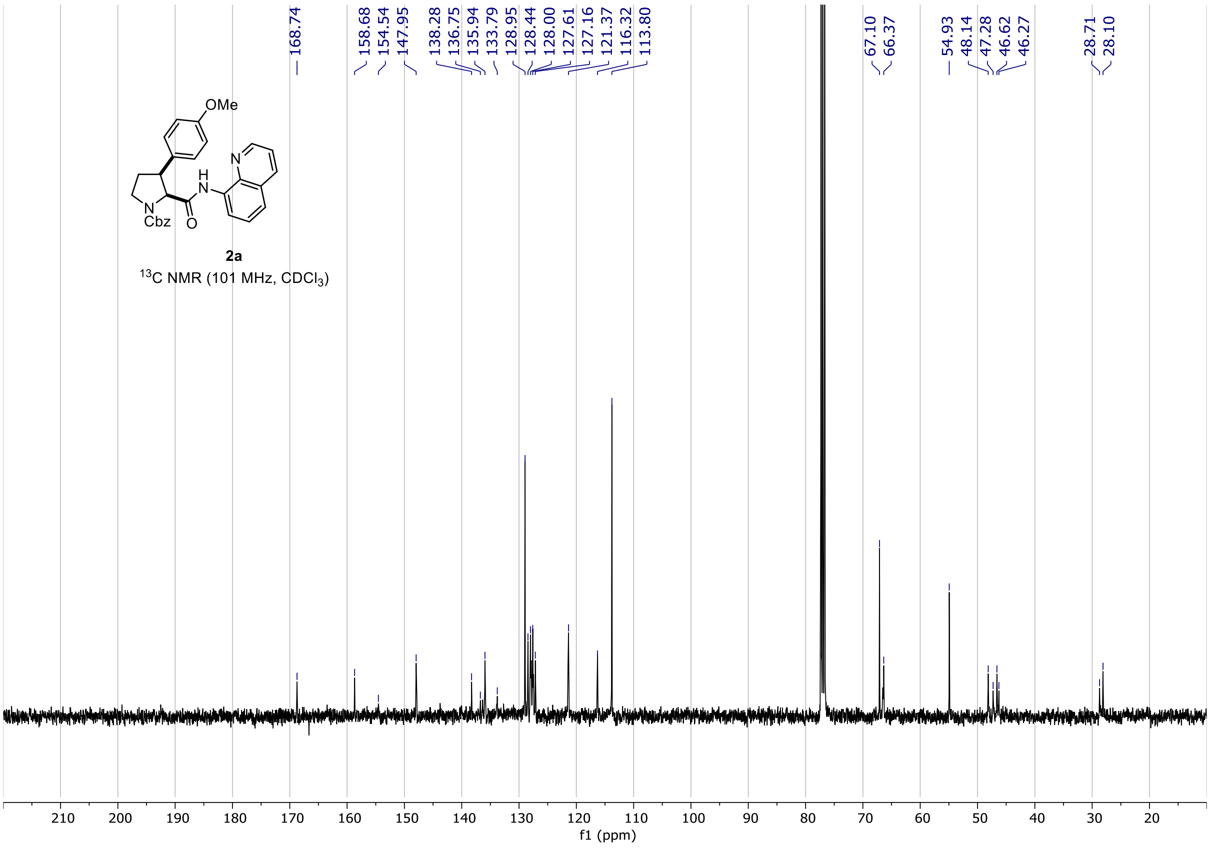

cis-(±)-Benzyl 3-(4-fluorophenyl)-2-(quinolin-8-ylcarbamoyl)pyrrolidine-1-carboxylate (2b)

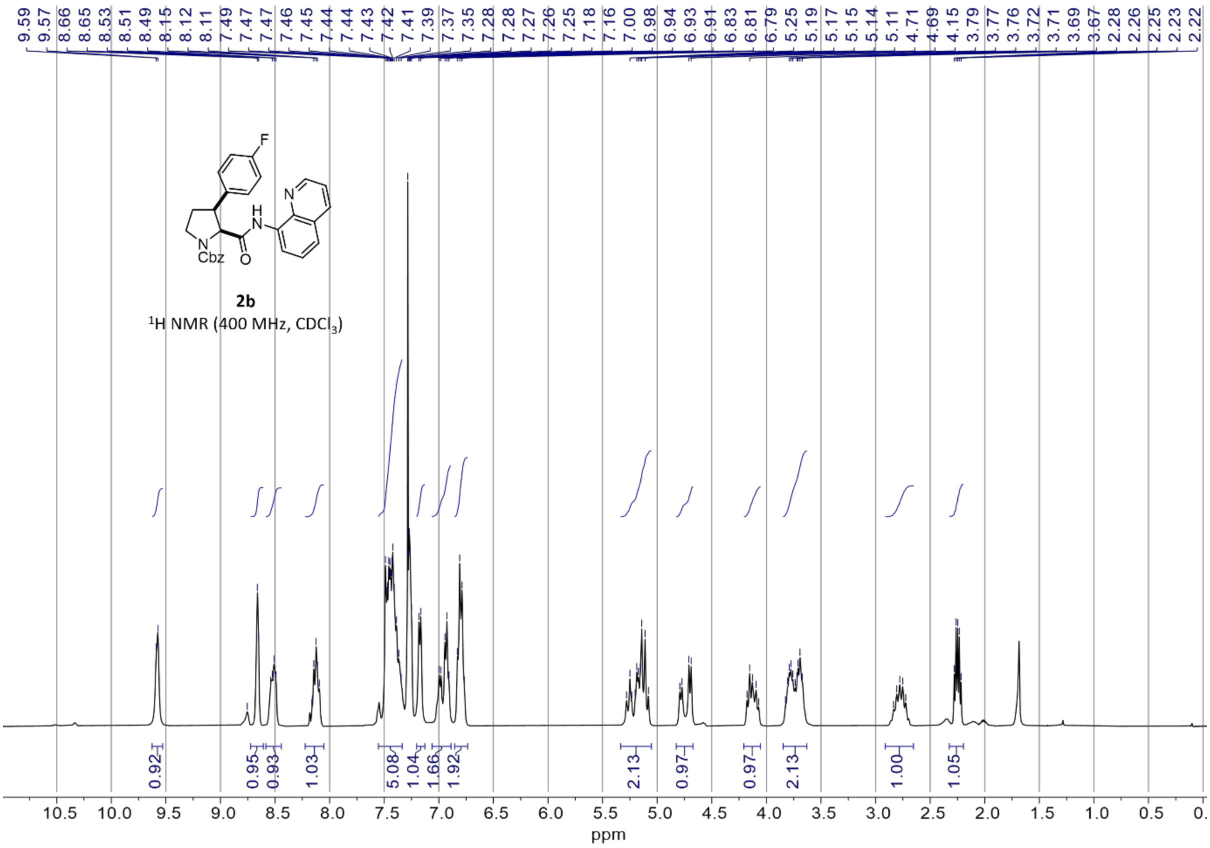

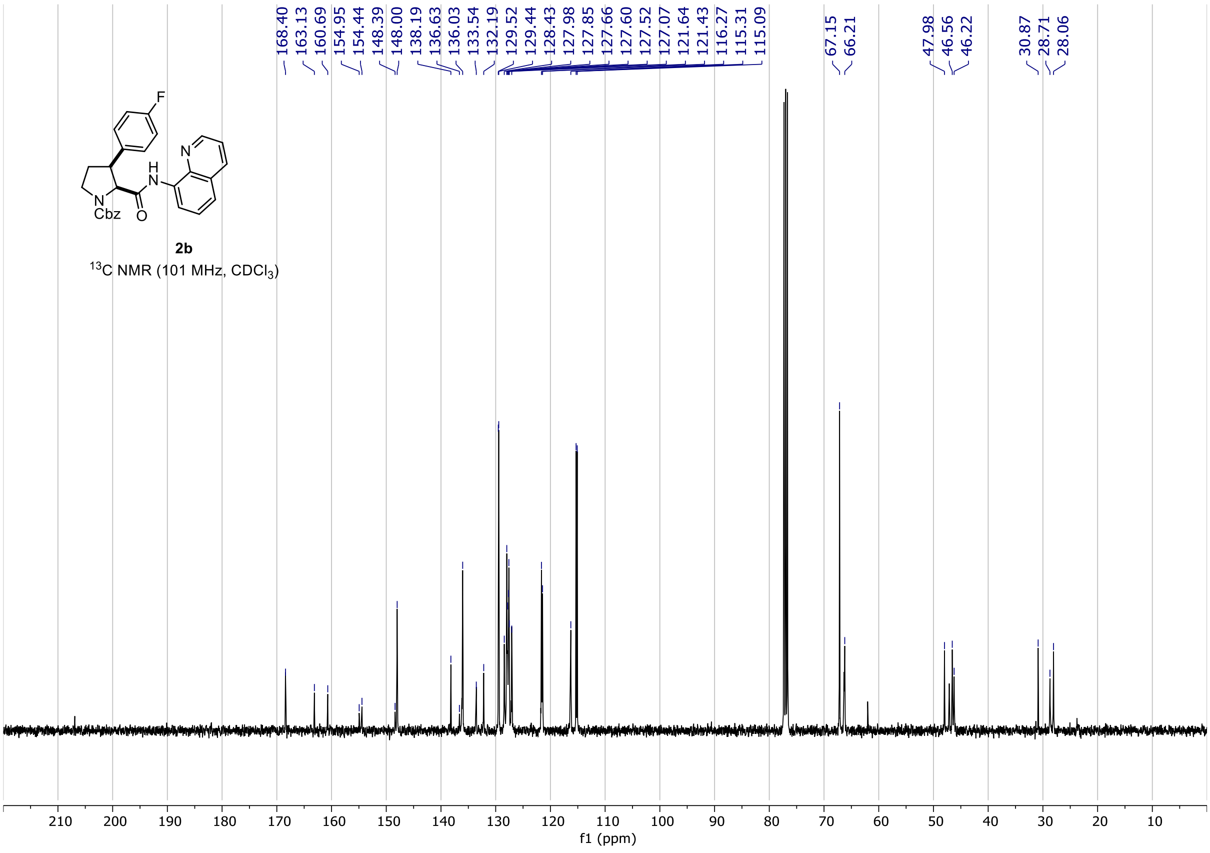

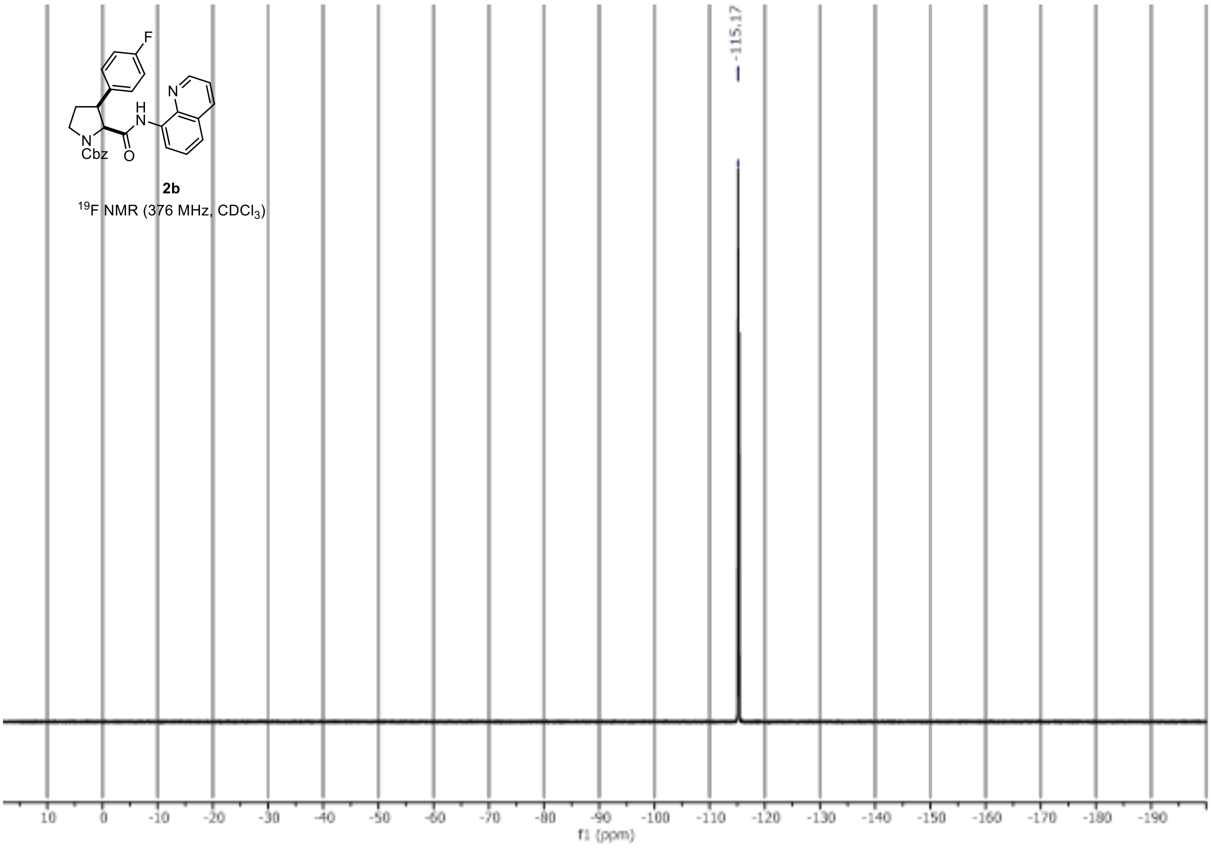
2b
^{19}F NMR (376 MHz, CDCl$_3$)
-115.17
f1 (ppm)

cis-(±)-Benzyl 3-(4-methoxyphenyl)-2-(quinolin-8-ylcarbamoyl)piperidine-1-carboxylate (4a)

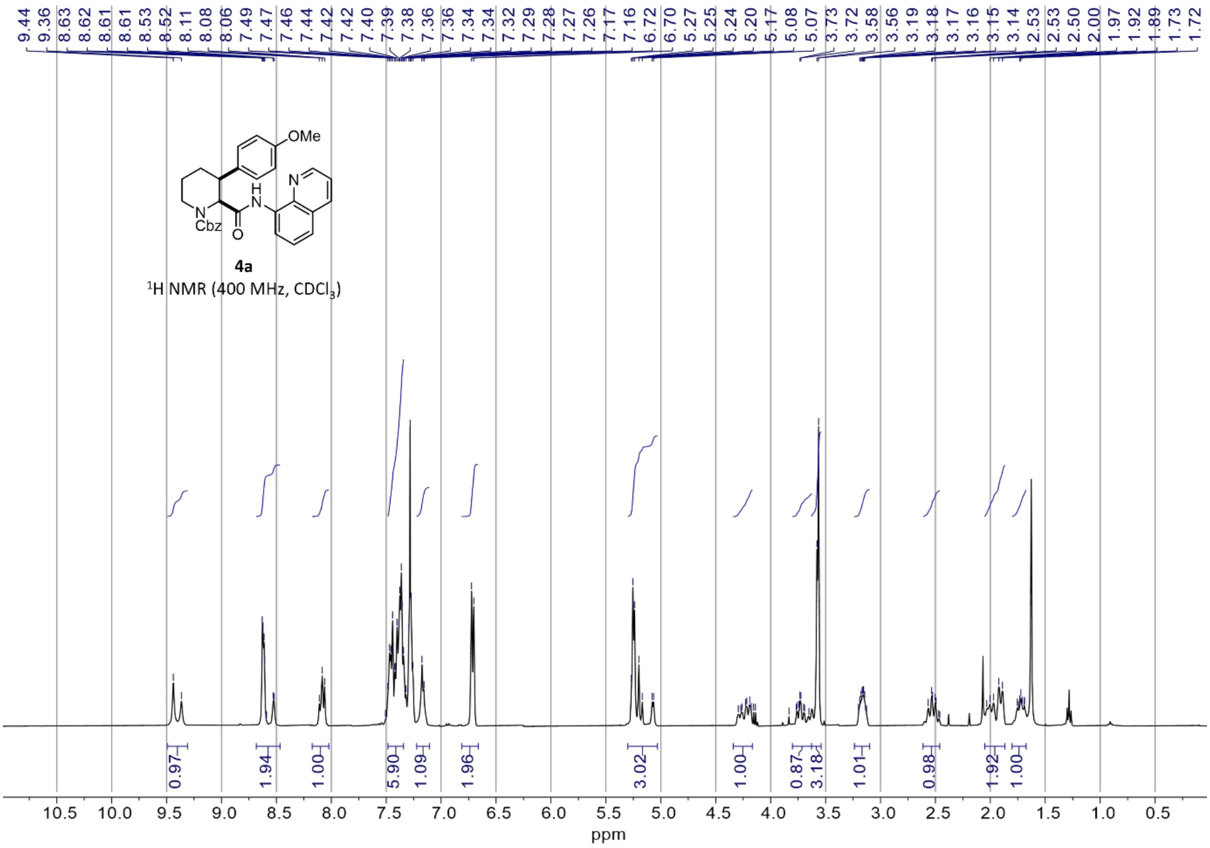

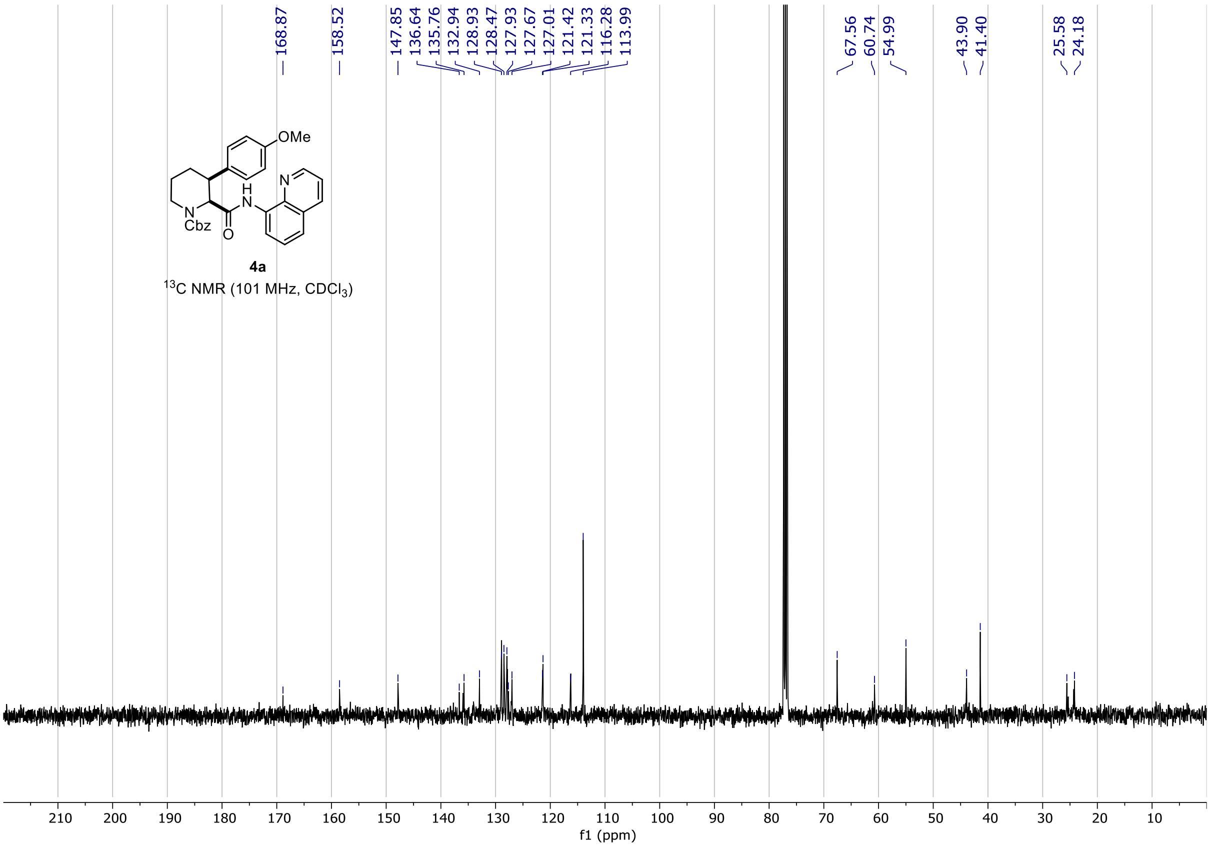

***cis*-(±)-1-((Benzyloxy)carbonyl)-3-(4-methoxyphenyl)pyrrolidine-2-carboxylic acid (20a)**

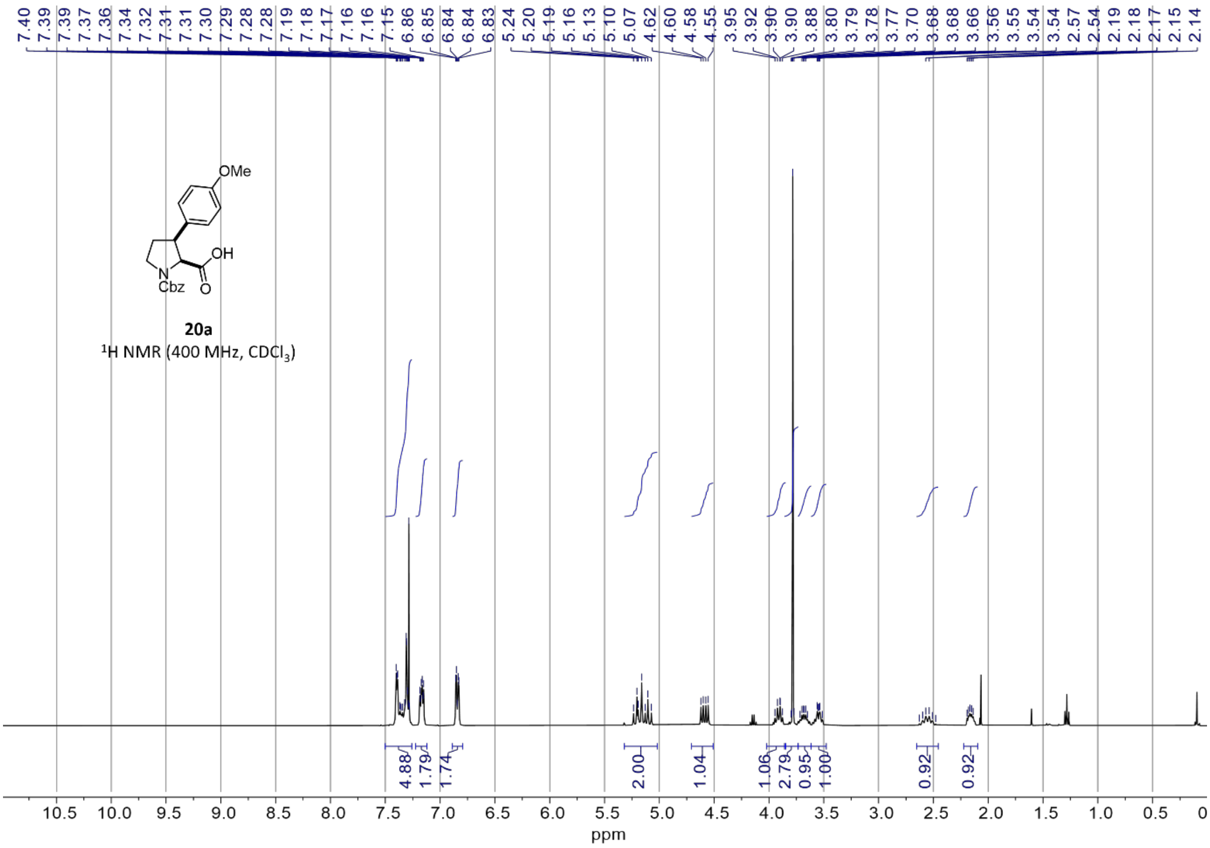

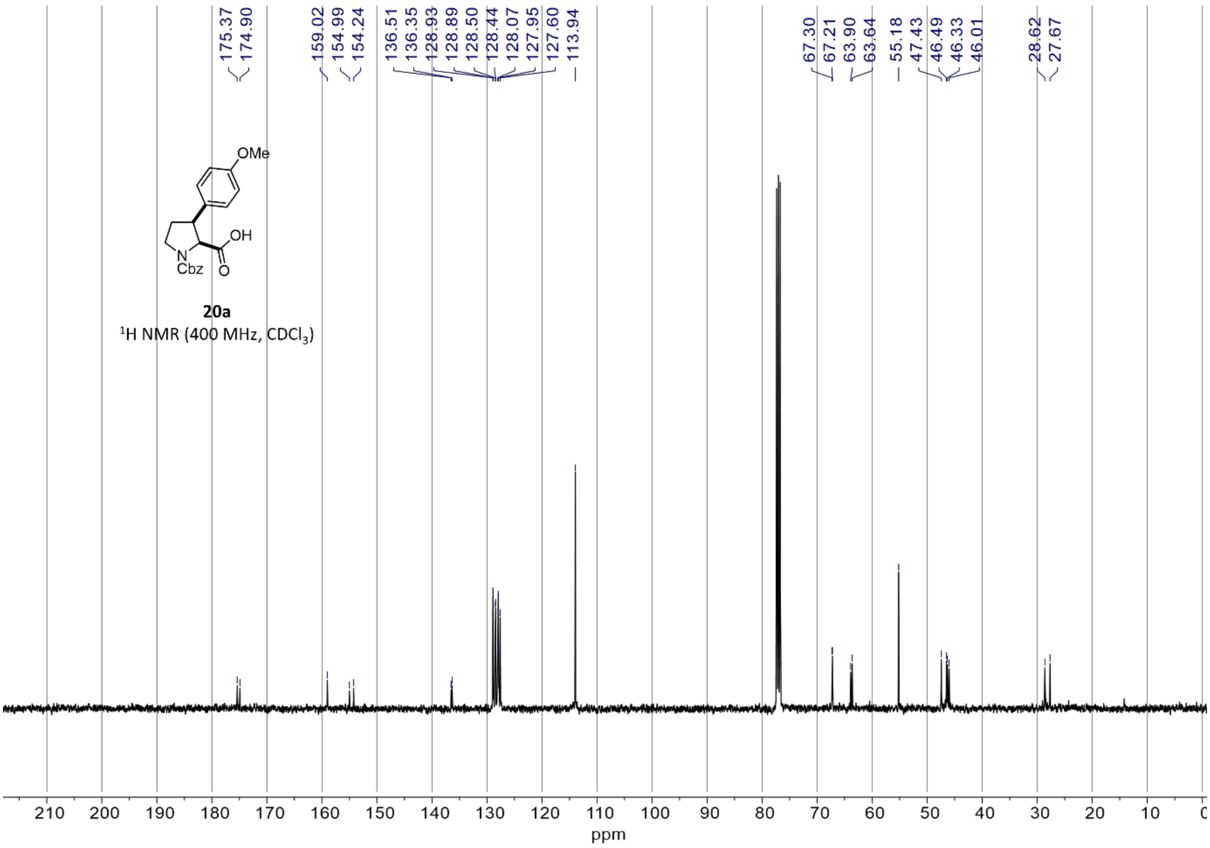

***cis*-(±)-1-((Benzyloxy)carbonyl)-3-(4-fluorophenyl)pyrrolidine-2-carboxylic acid (20b)**

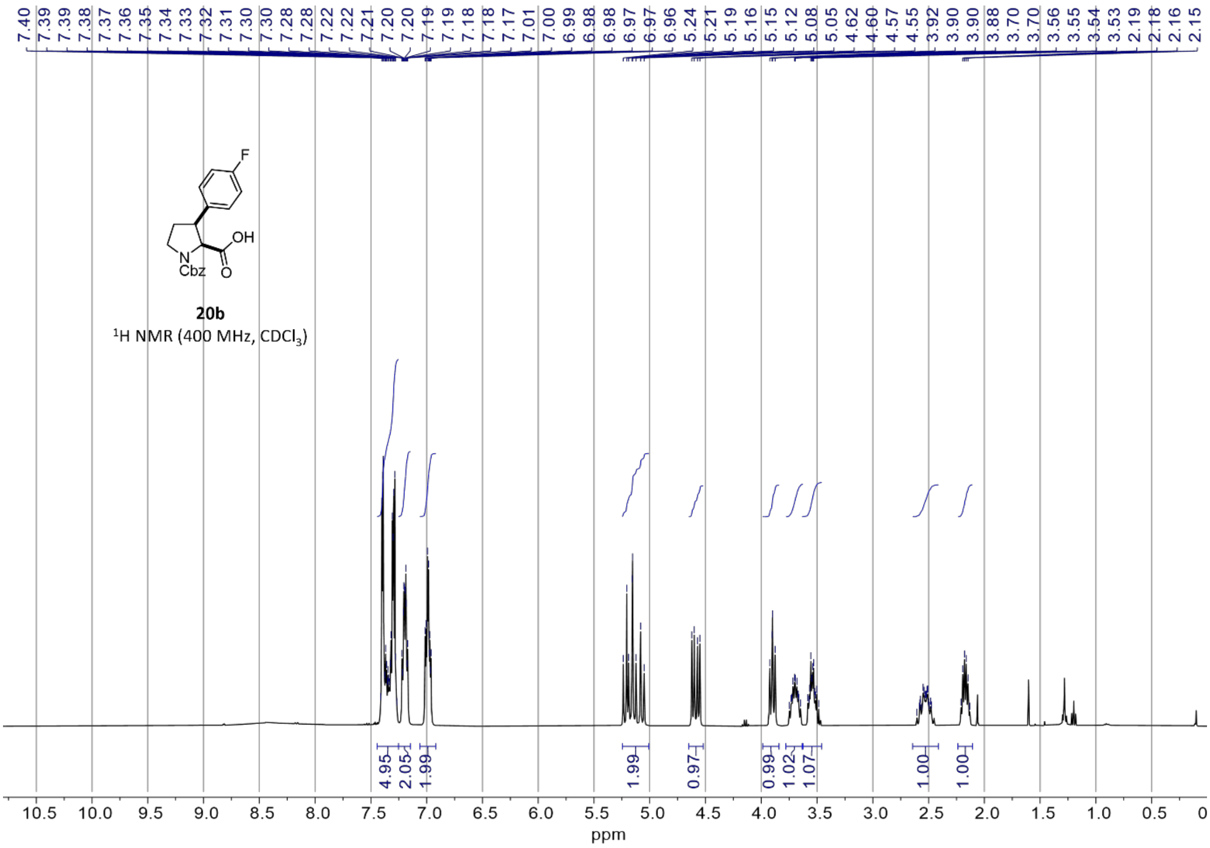

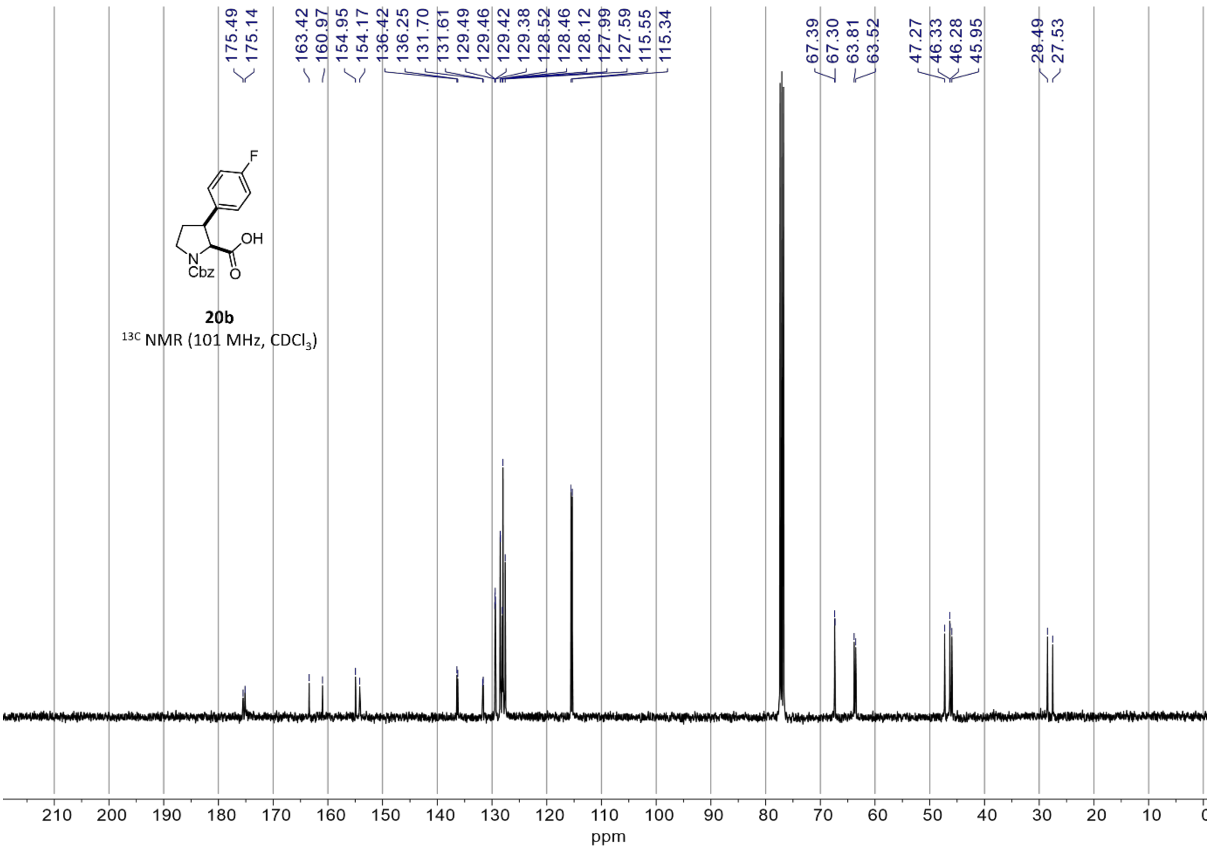

20b

^{19}F NMR (376 MHz, $CDCl_3$)

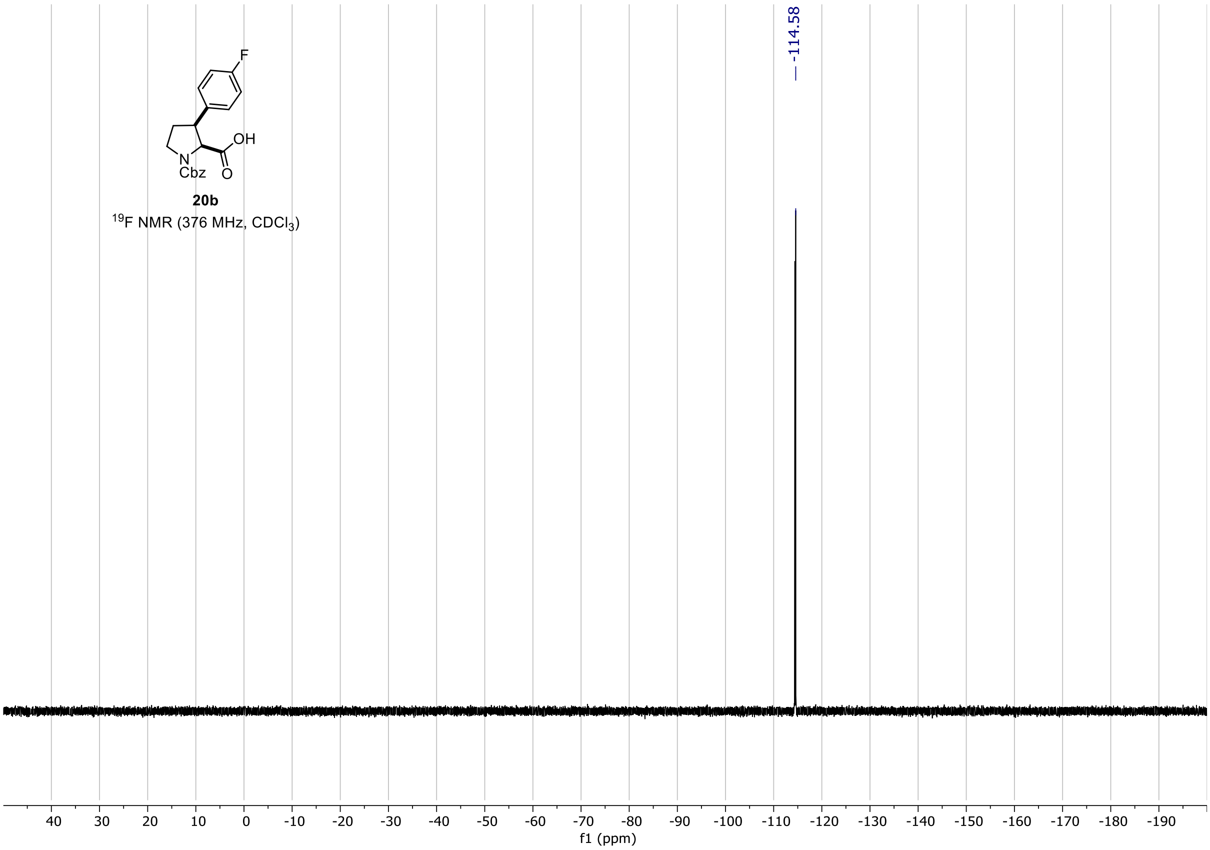

cis-(±)-Benzyl 2-carbamoyl-3-(4-methoxyphenyl)pyrrolidine-1-carboxylate (21a)

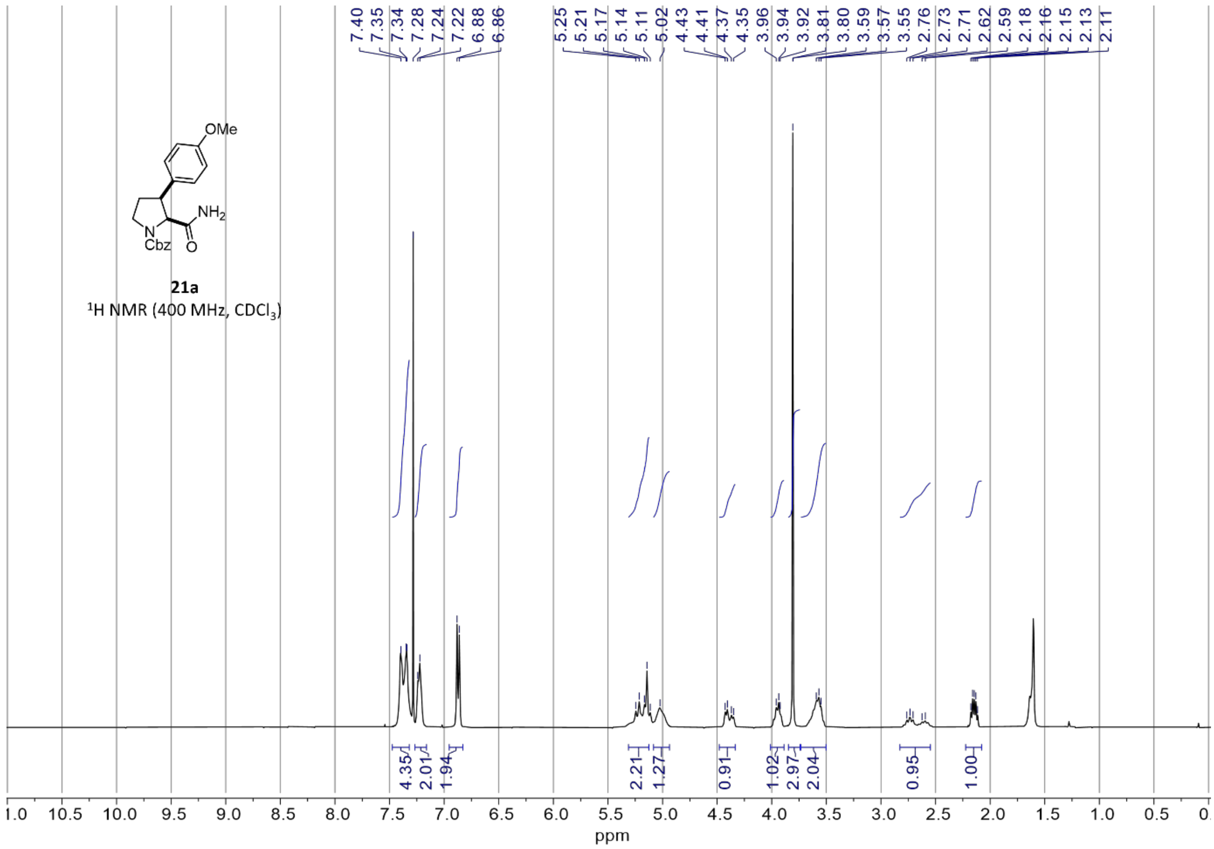

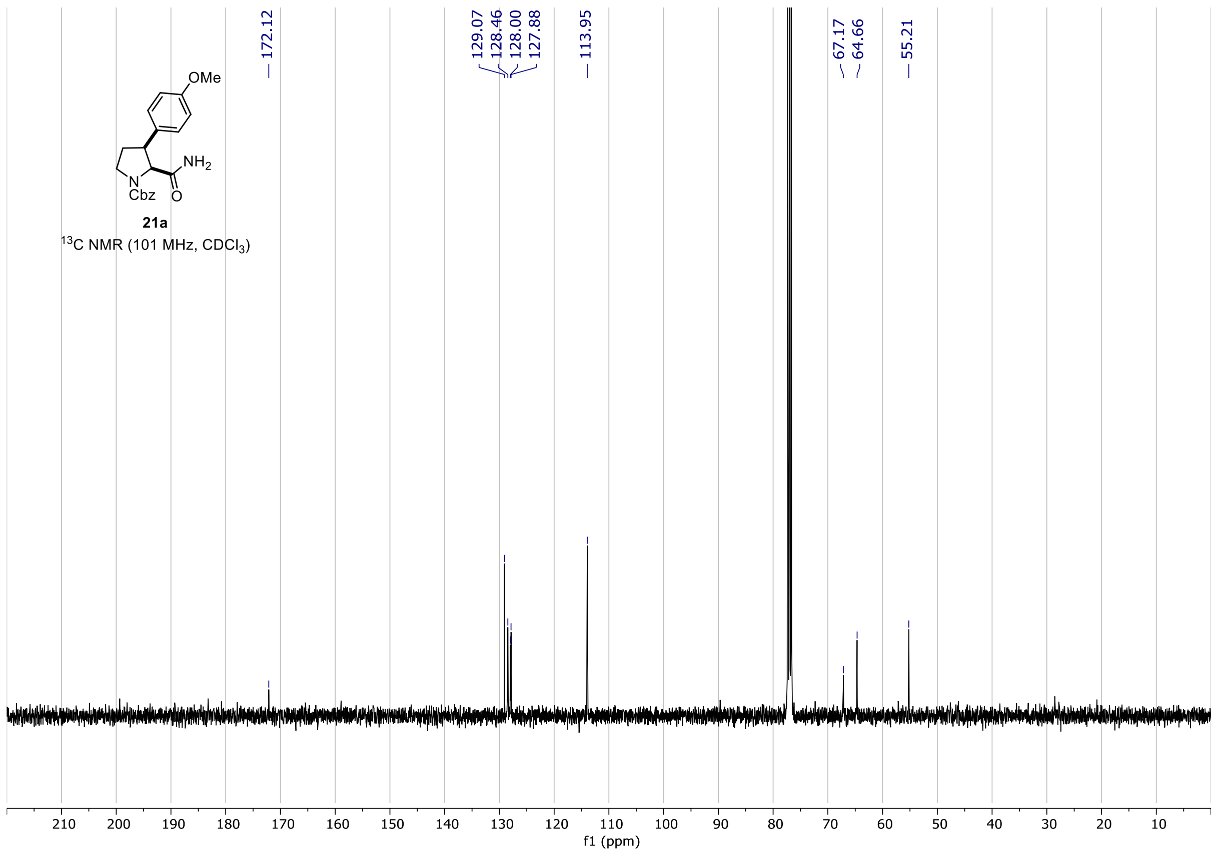

cis-(±)-Benzyl 2-carbamoyl-3-(4-fluorophenyl)pyrrolidine-1-carboxylate (21b)

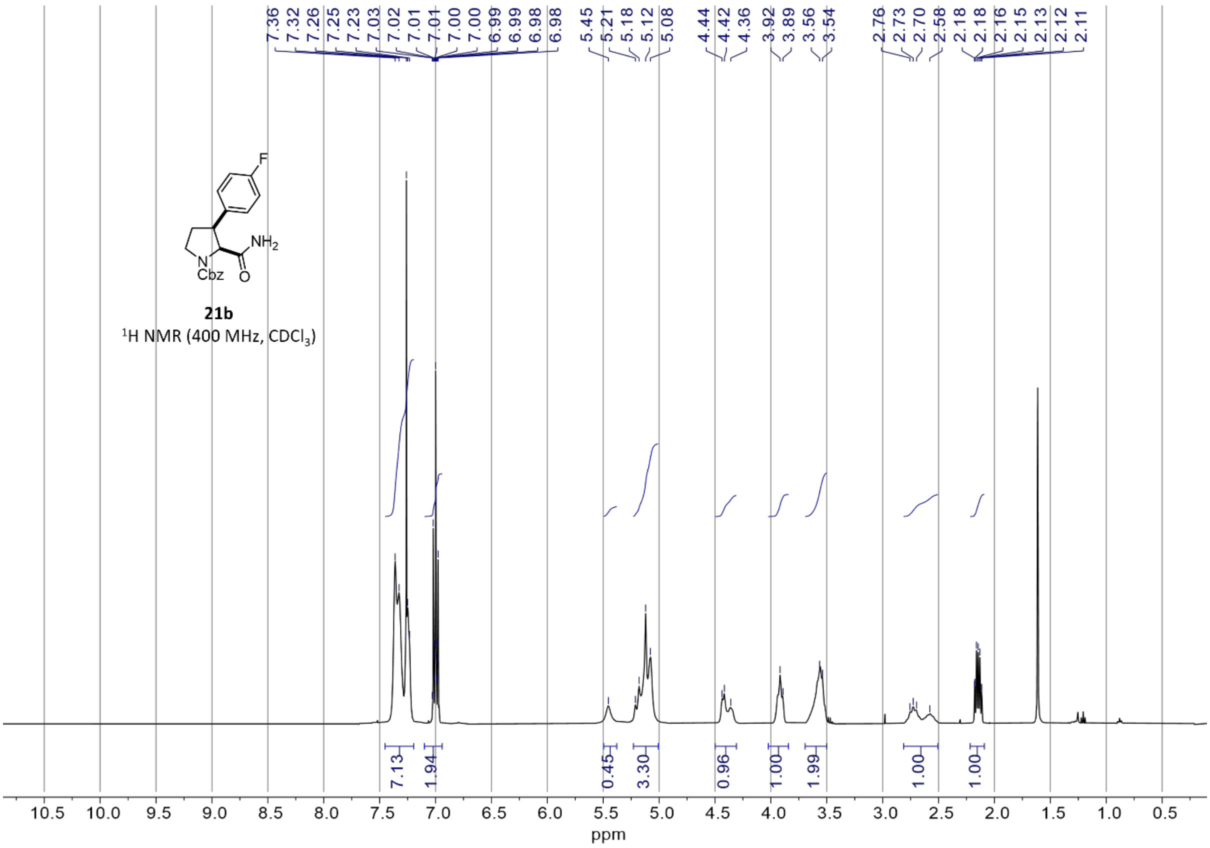

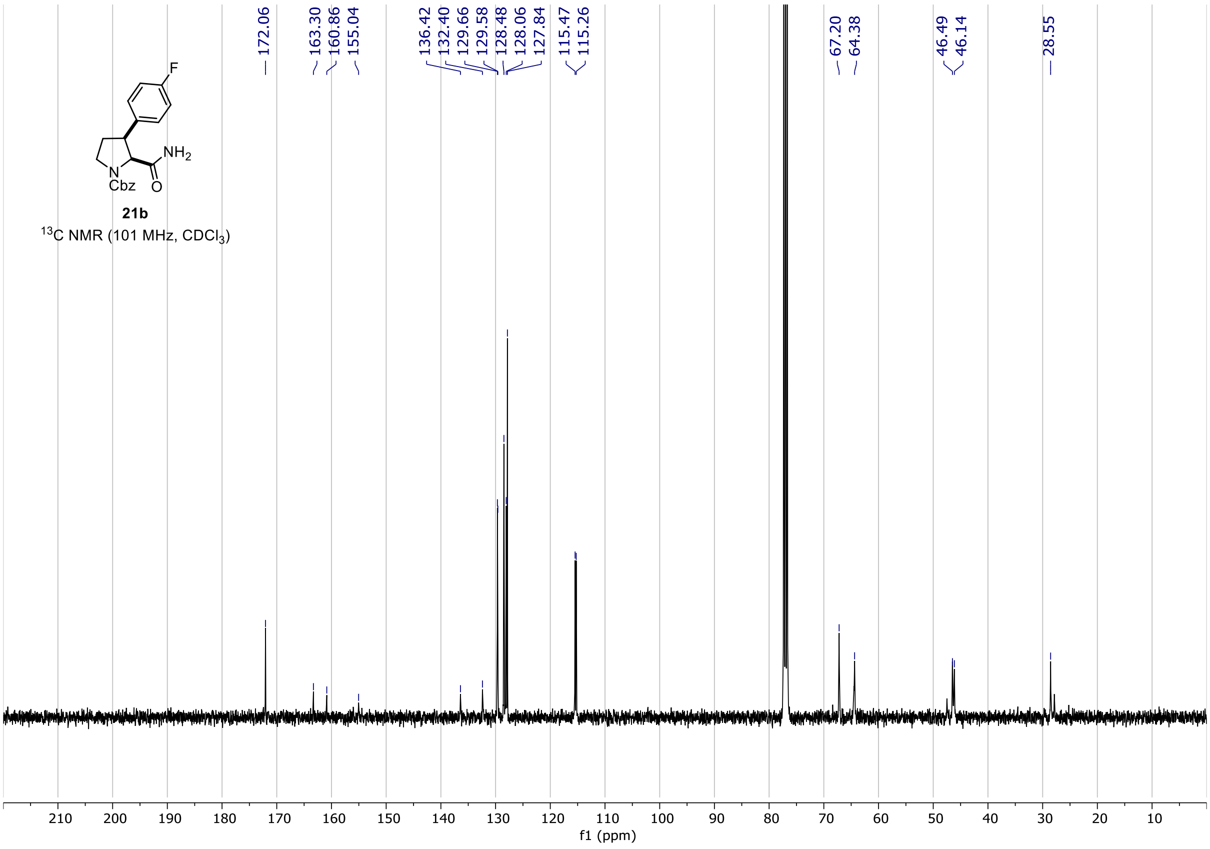

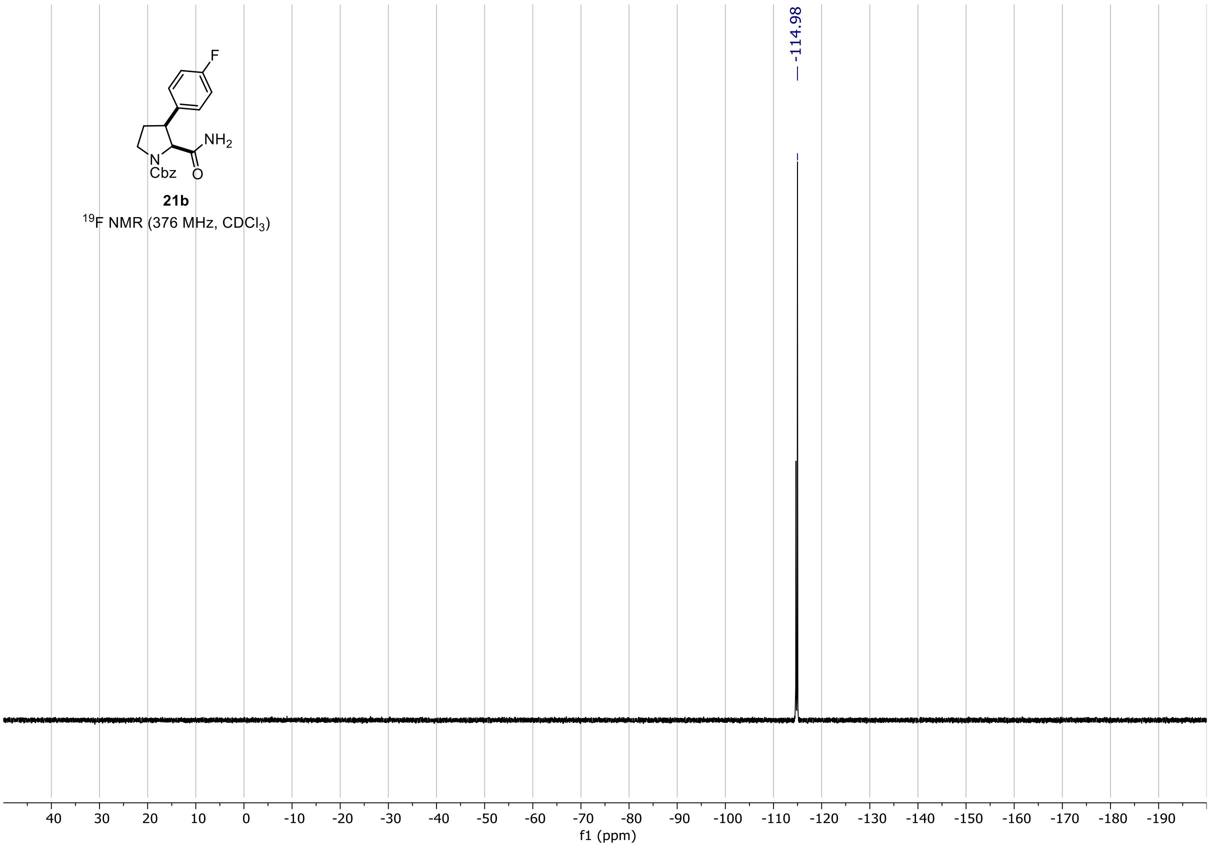
-114.98
F
NH_2
N
Cbz
O
21b
^{19}F NMR (376 MHz, $CDCl_3$)
40 30 20 10 0 -10 -20 -30 -40 -50 -60 -70 -80 -90 -100 -110 -120 -130 -140 -150 -160 -170 -180 -190
f1 (ppm)

cis-(±)-Benzyl 2-(hydroxymethyl)-3-(4-methoxyphenyl)pyrrolidine-1-carboxylate (22a)

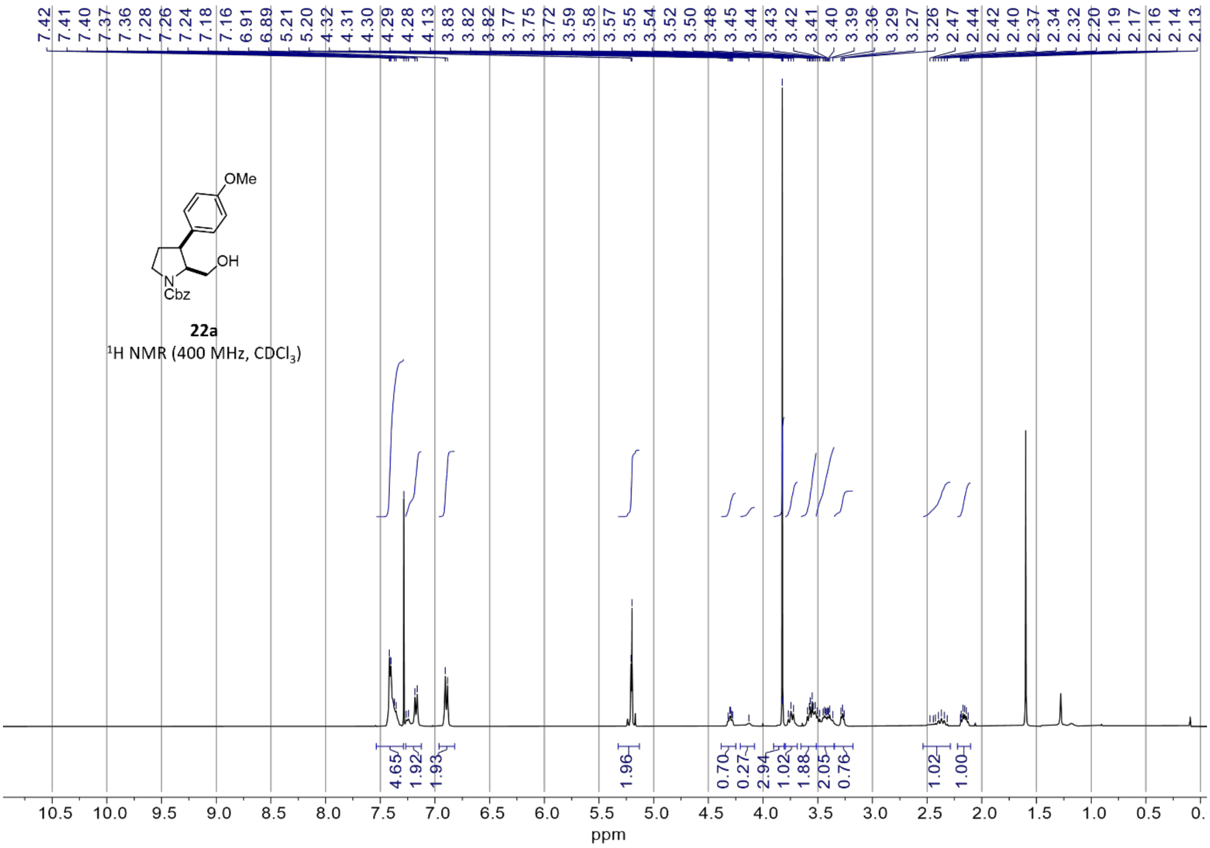

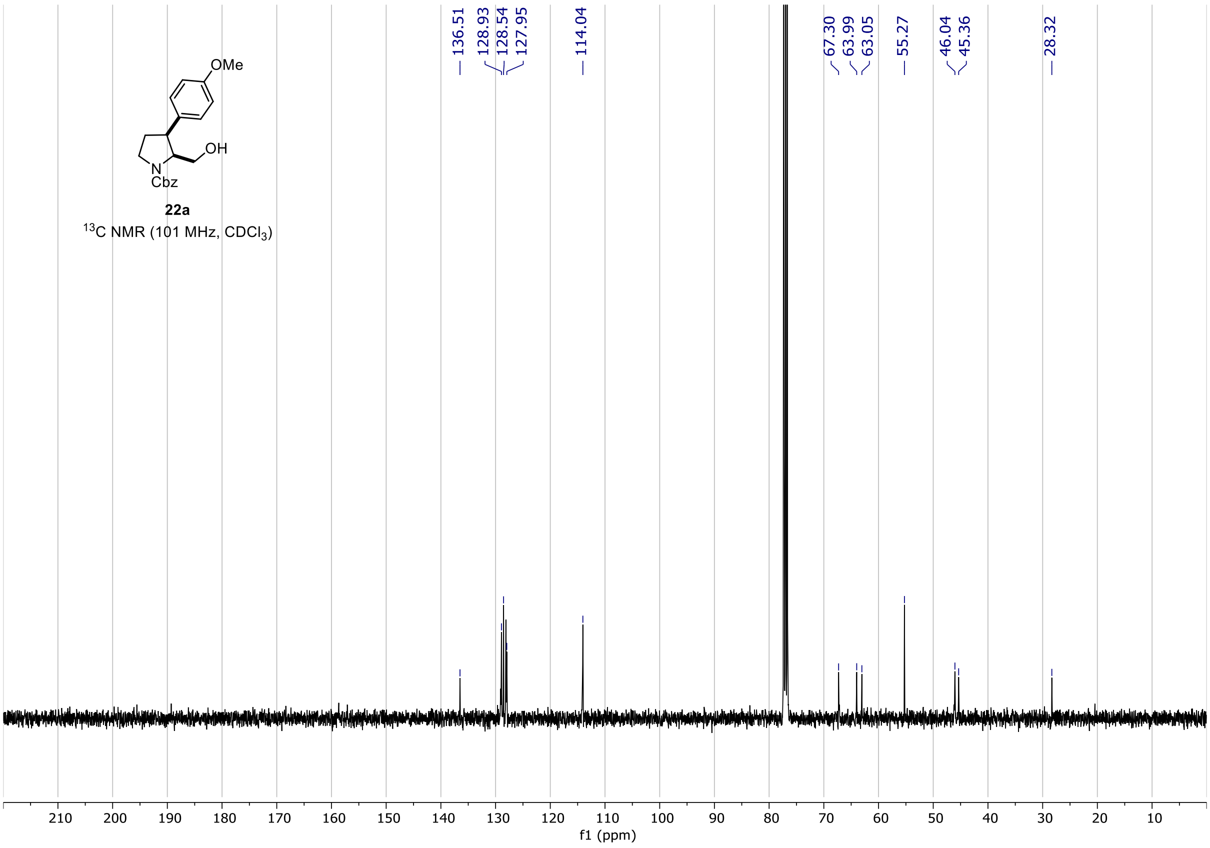

***cis*-(±)-Benzyl 3-(4-fluorophenyl)-2-(hydroxymethyl)pyrrolidine-1-carboxylate (22b)**

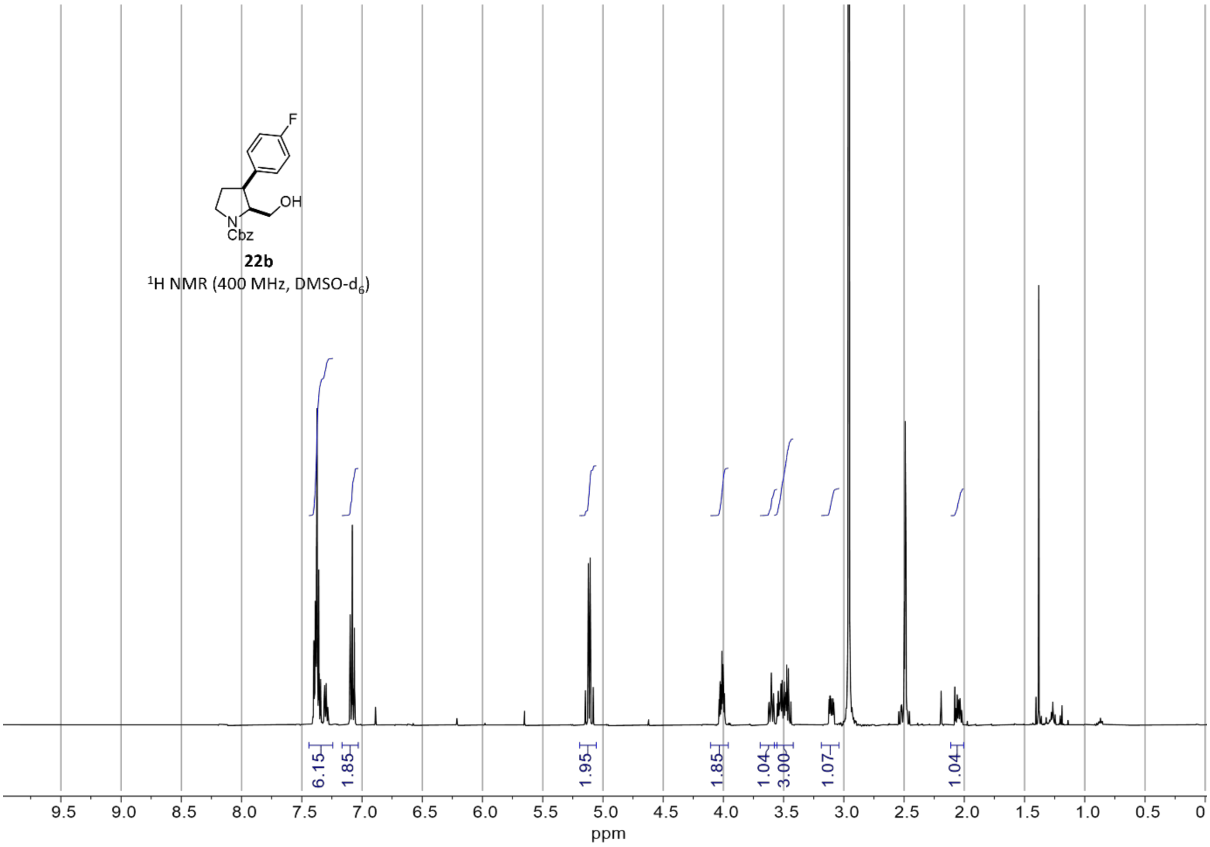

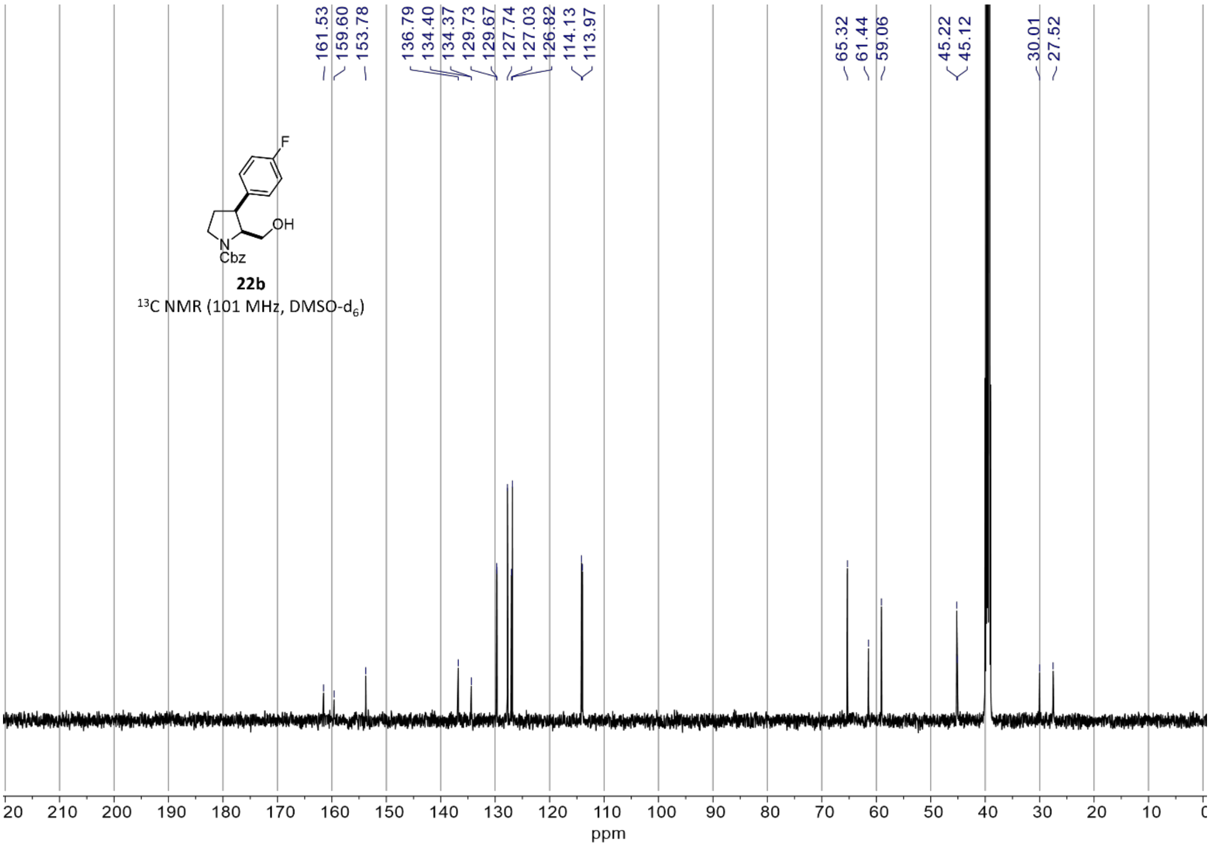

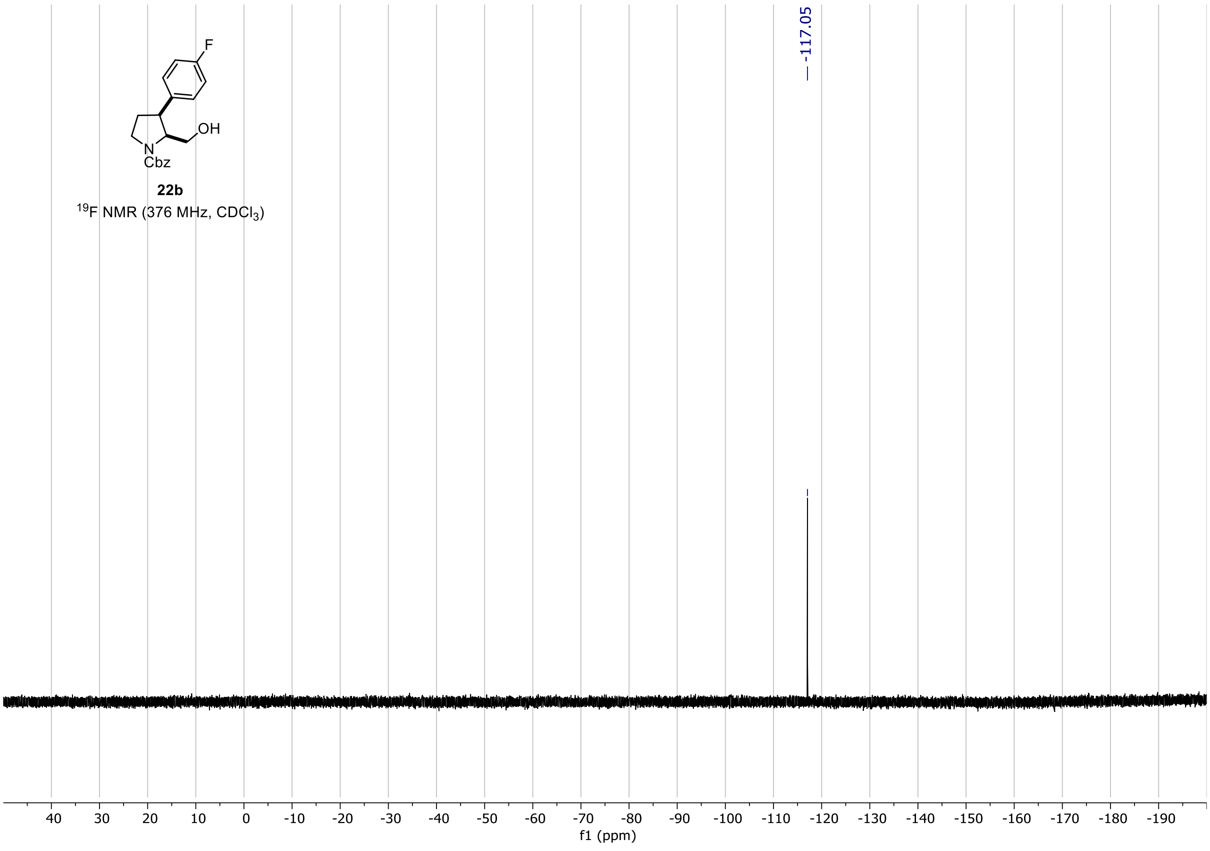
F
OH
N
Cbz
22b
19F NMR (376 MHz, CDCl3)
-117.05
40 30 20 10 0 -10 -20 -30 -40 -50 -60 -70 -80 -90 -100 -110 -120 -130 -140 -150 -160 -170 -180 -190
f1 (ppm)

cis-(±)-2-Carboxy-3-(4-methoxyphenyl)pyrrolidin-1-ium iodide (FRAG1)

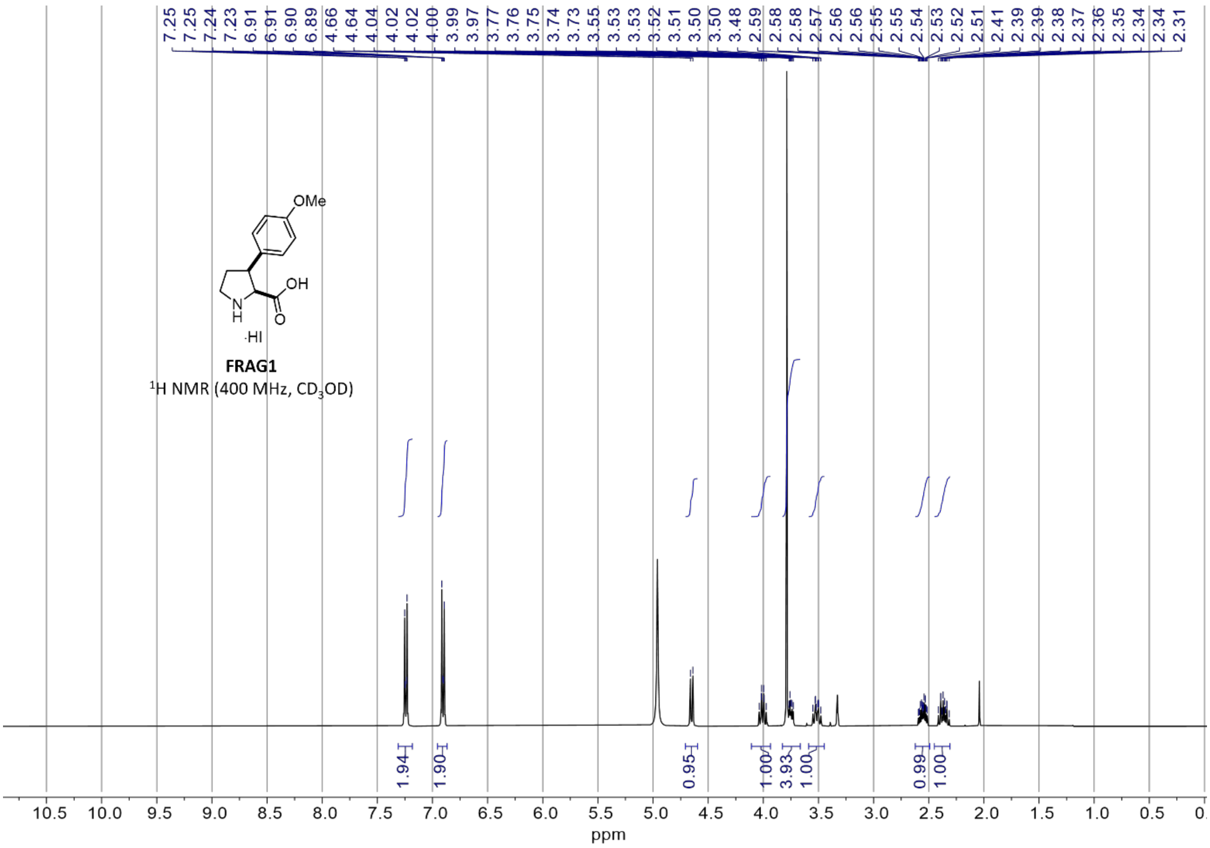

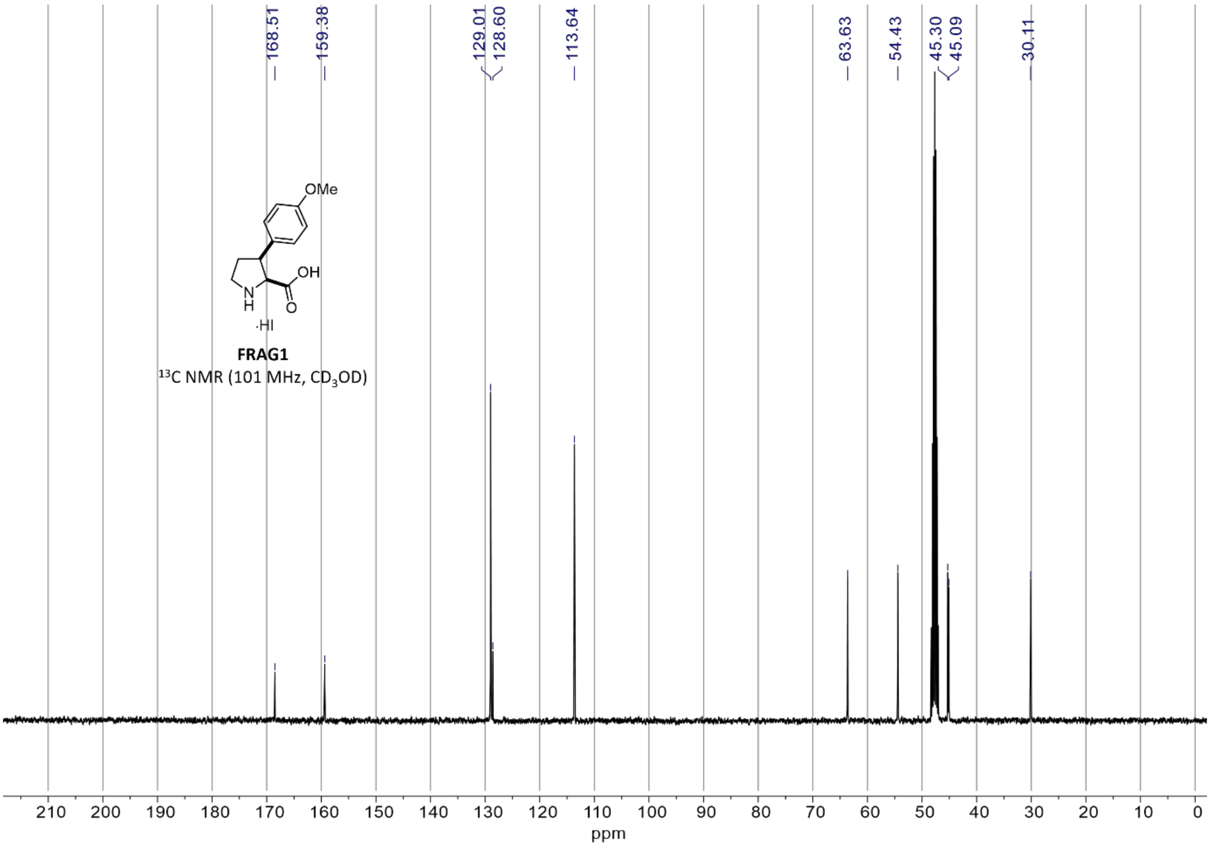

***cis*-(±)-2-Carboxy-3-(4-fluorophenyl)pyrrolidin-1-ium iodide (FRAG2)**

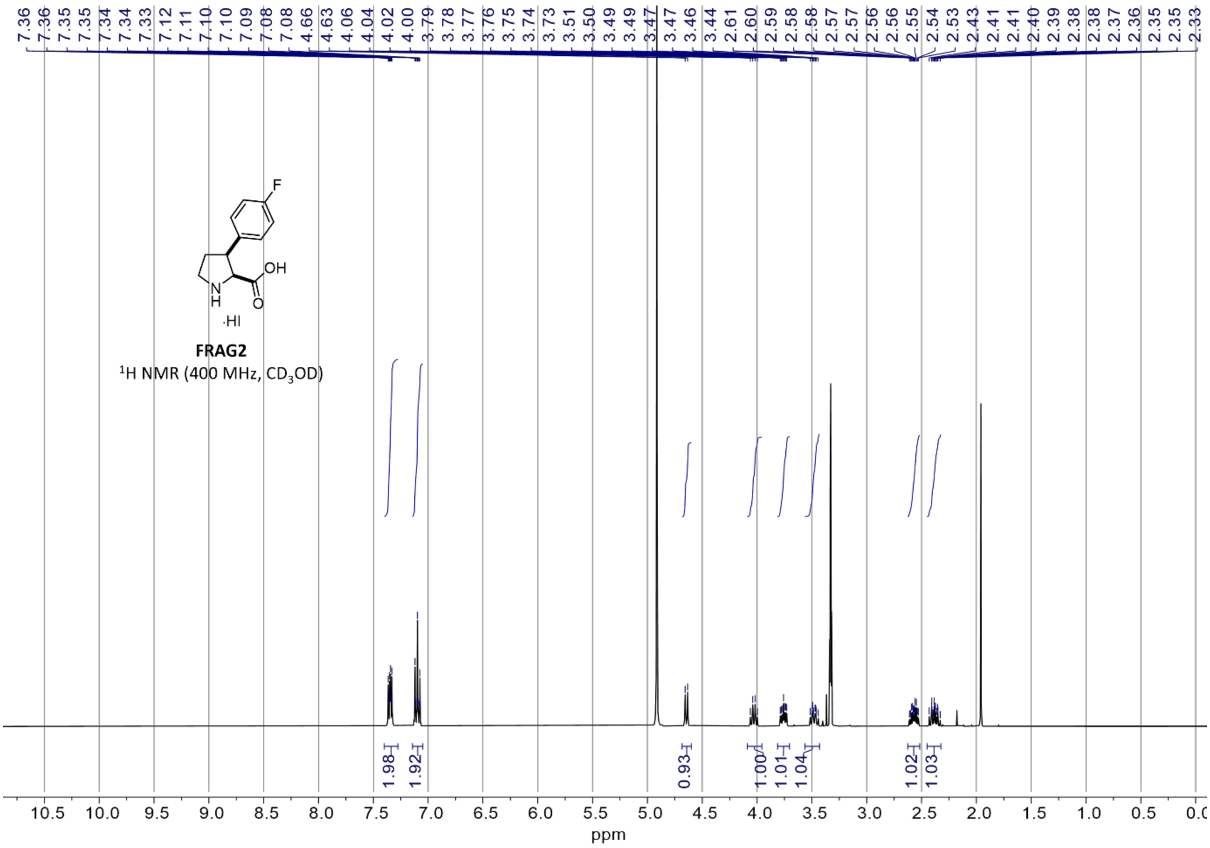

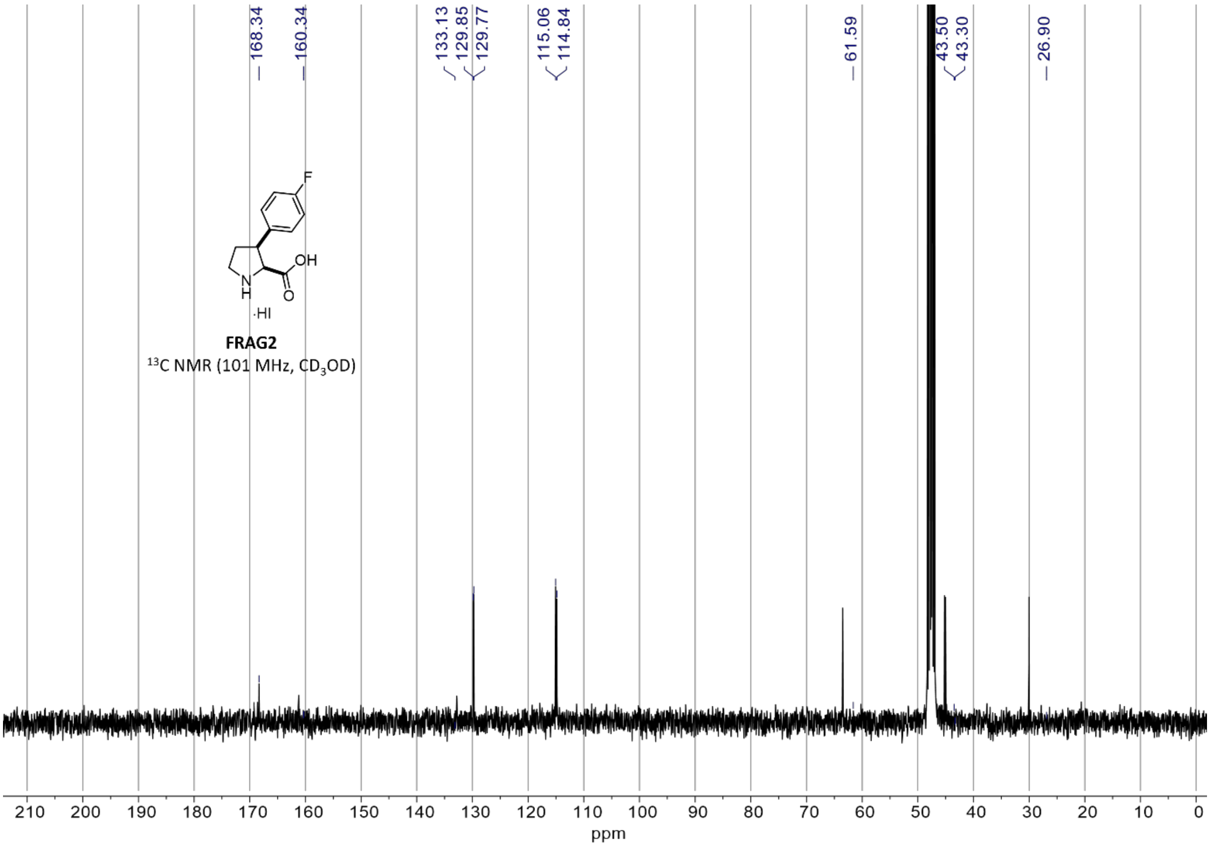

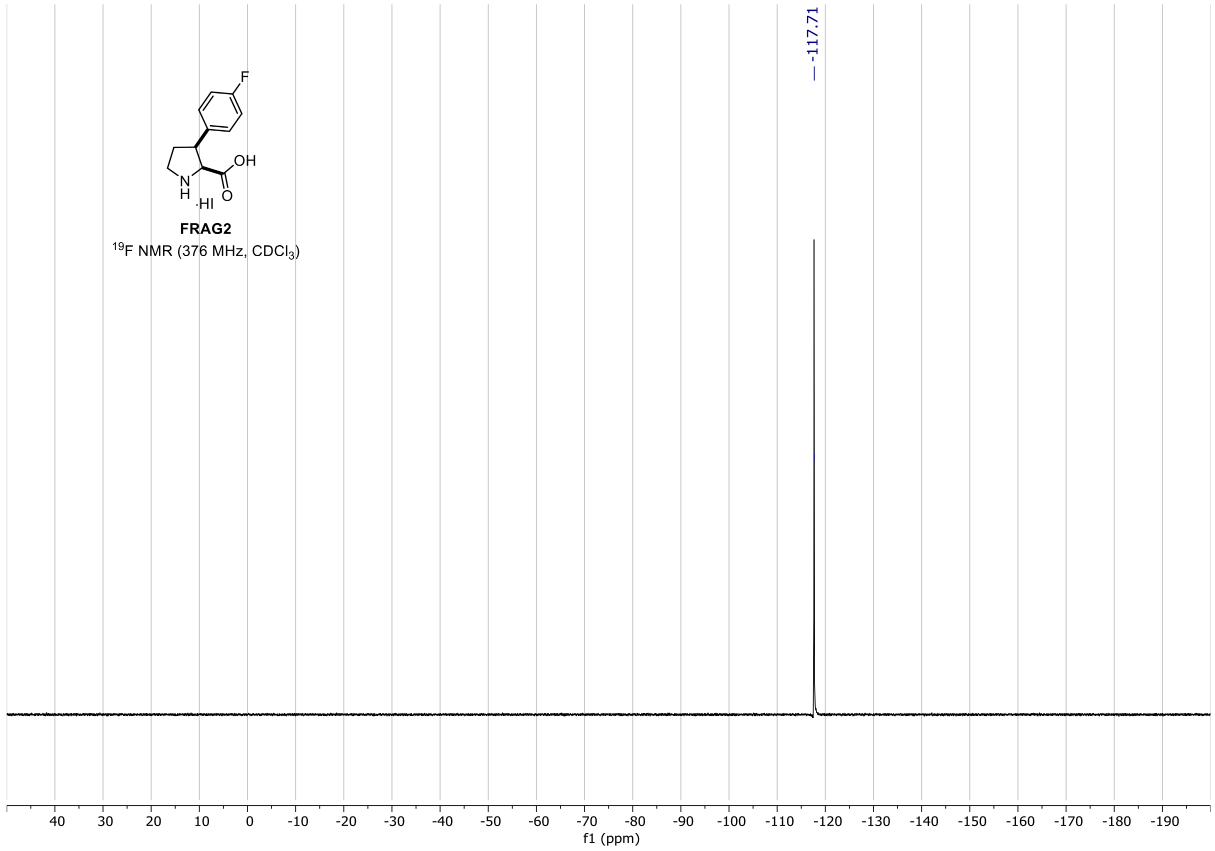
-117.71
F
OH
N
H
·HI
O
FRAG2
19F NMR (376 MHz, CDCl3)
40
30
20
10
0
-10
-20
-30
-40
-50
-60
-70
-80
-90
-100
-110
-120
-130
-140
-150
-160
-170
-180
-190
f1 (ppm)

cis-(±)-3-(4-Methoxyphenyl)pyrrolidine-2-carboxamide (FRAG3)

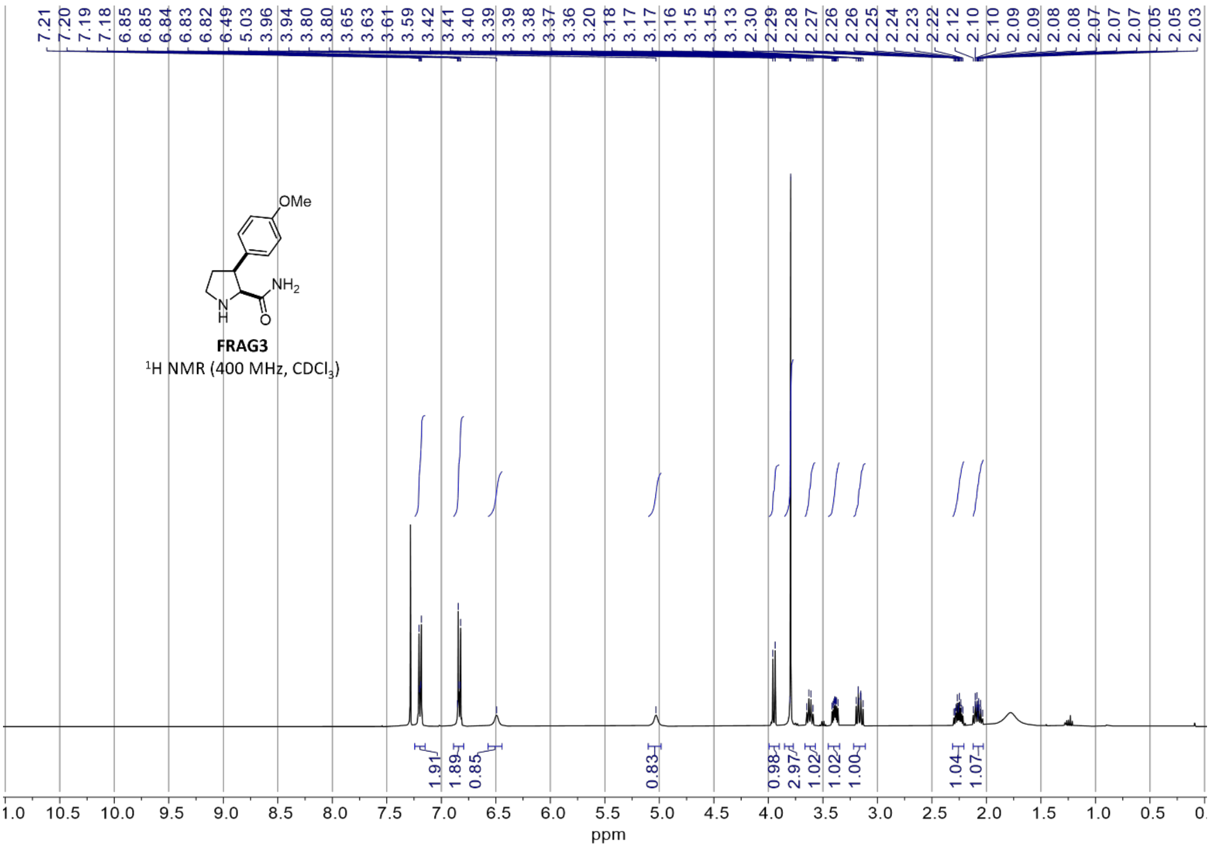

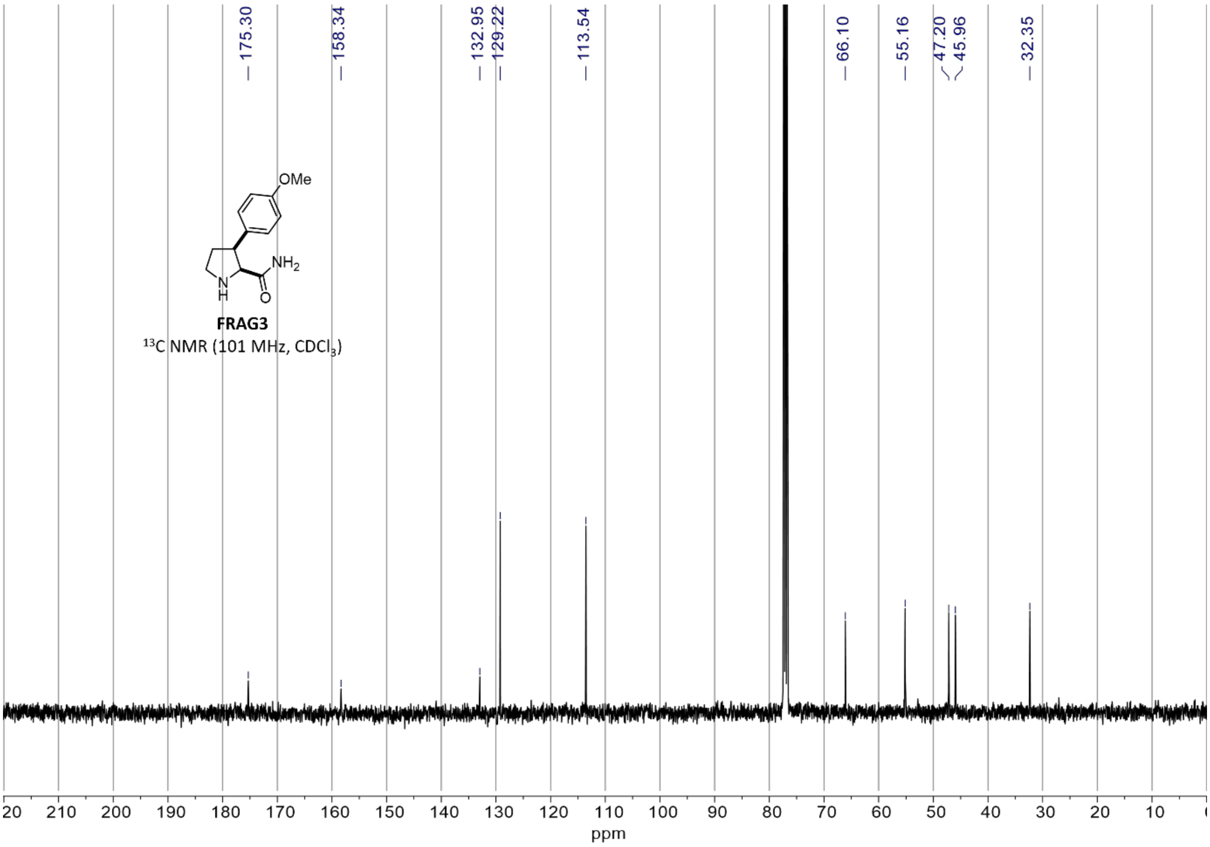

***cis*-(±)-3-(4-Fluorophenyl)pyrrolidine-2-carboxamide (FRAG4)**

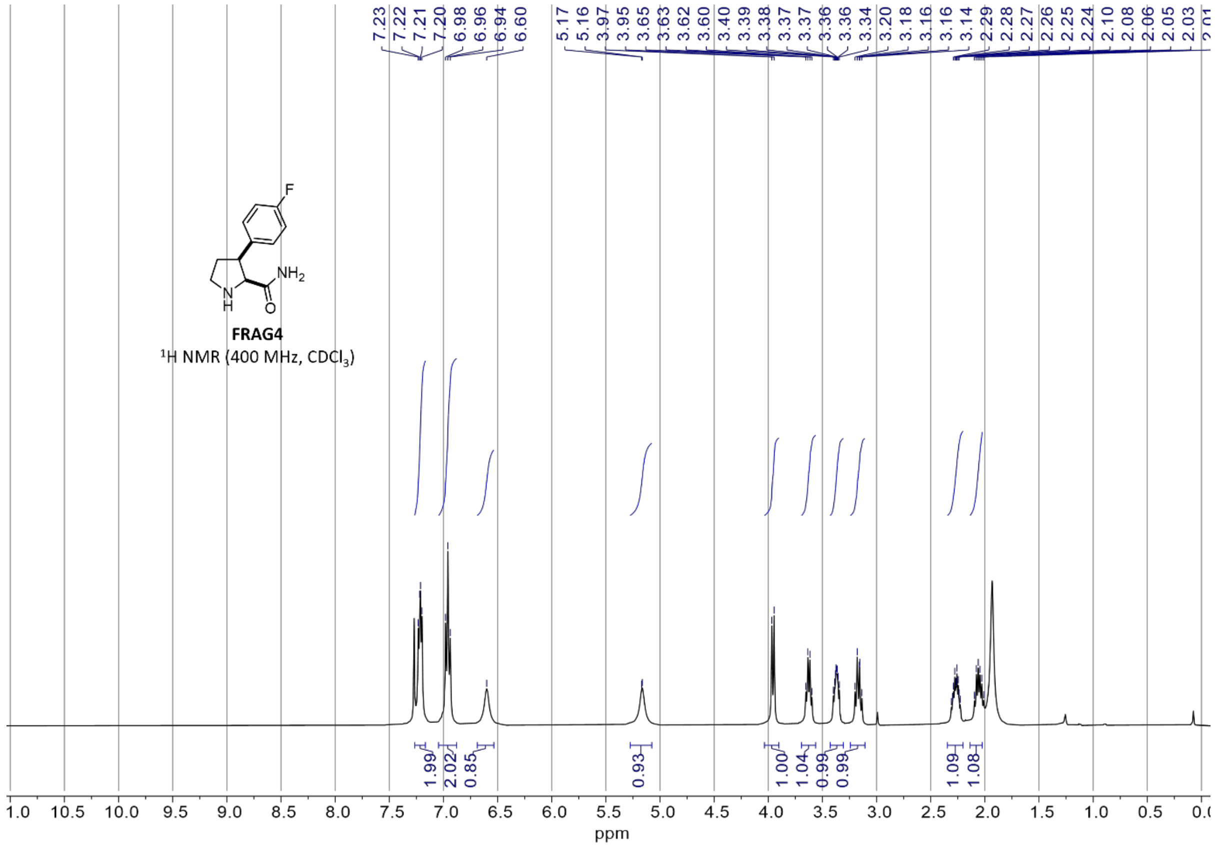

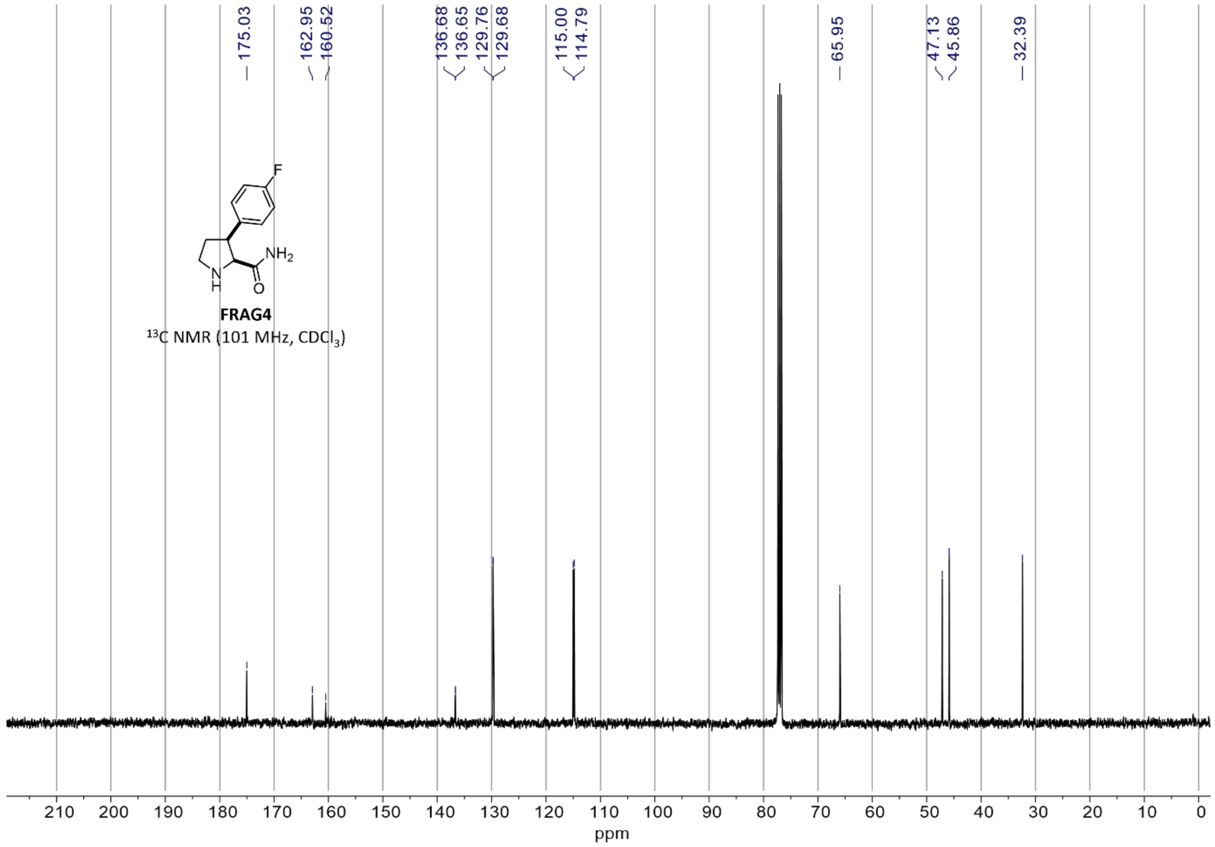

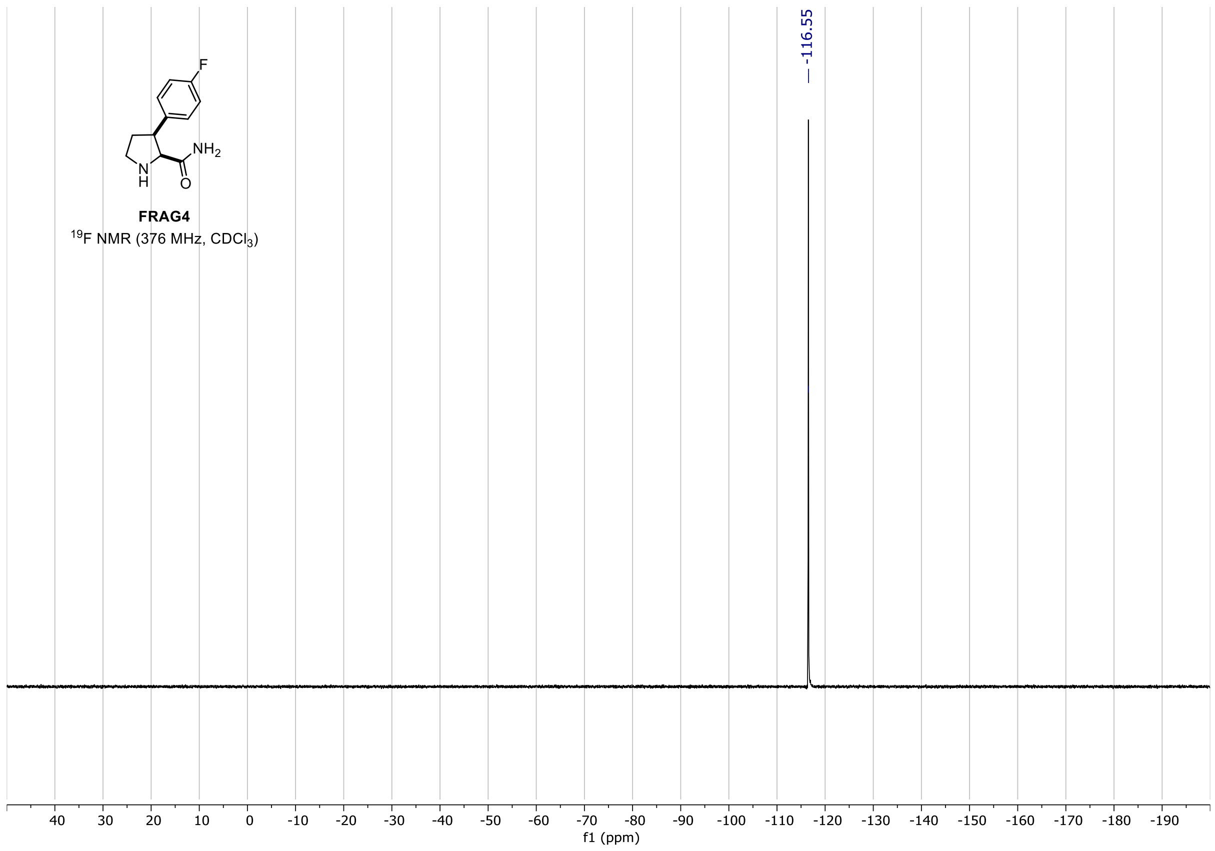
-116.55
F
NH2
N
H
O
FRAG4
19F NMR (376 MHz, CDCl3)
40 30 20 10 0 -10 -20 -30 -40 -50 -60 -70 -80 -90 -100 -110 -120 -130 -140 -150 -160 -170 -180 -190
f1 (ppm)

cis-(±)-3-((4-Methoxyphenyl)pyrrolidin-2-yl)methanol (FRAG5)

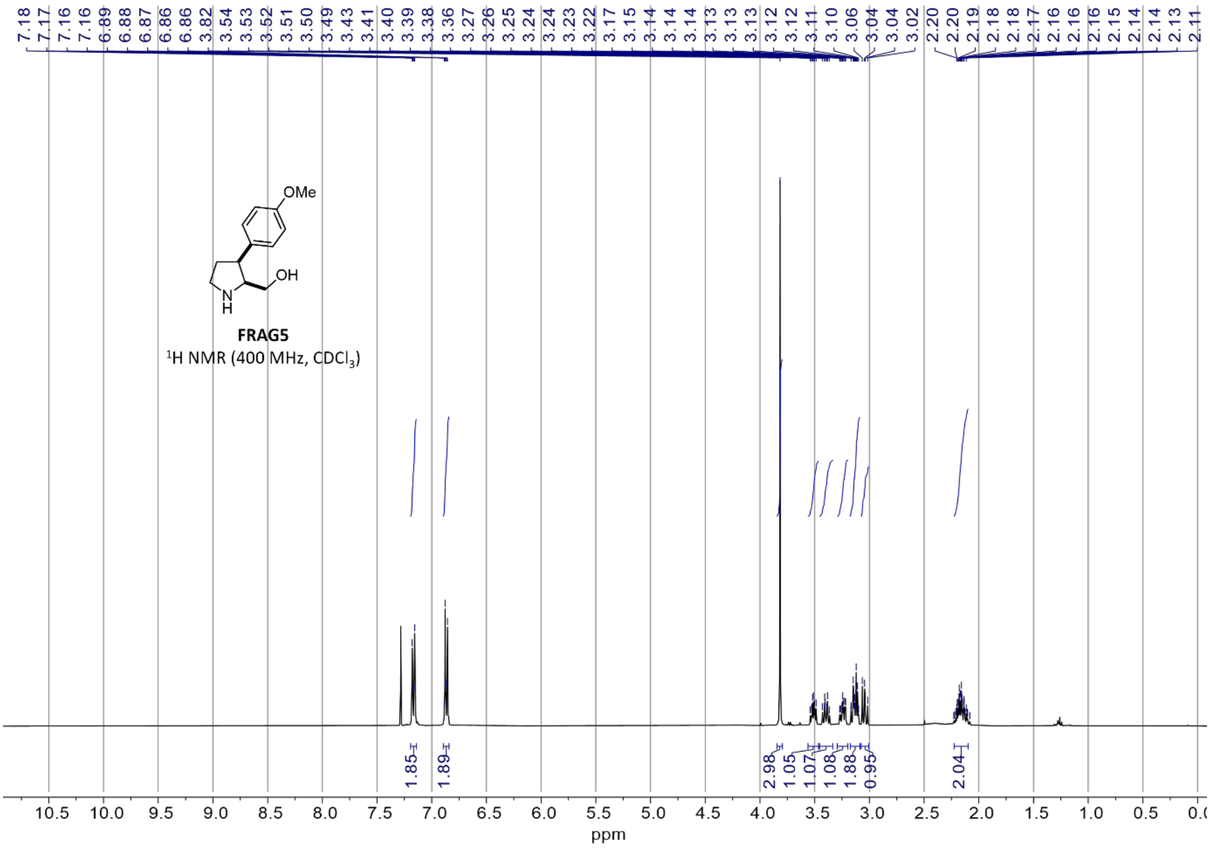

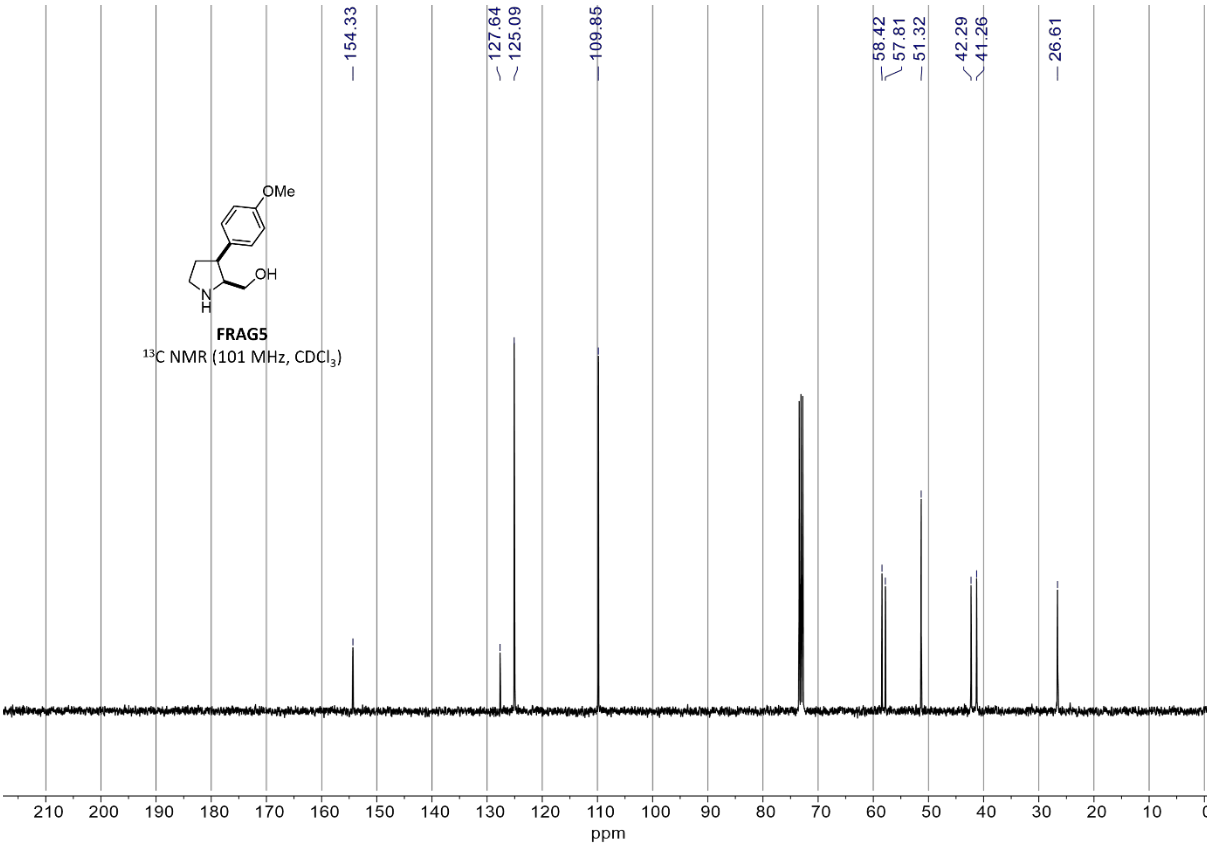

cis-(±)-3-((4-Fluorophenyl)pyrrolidin-2-yl)methanol (FRAG6)

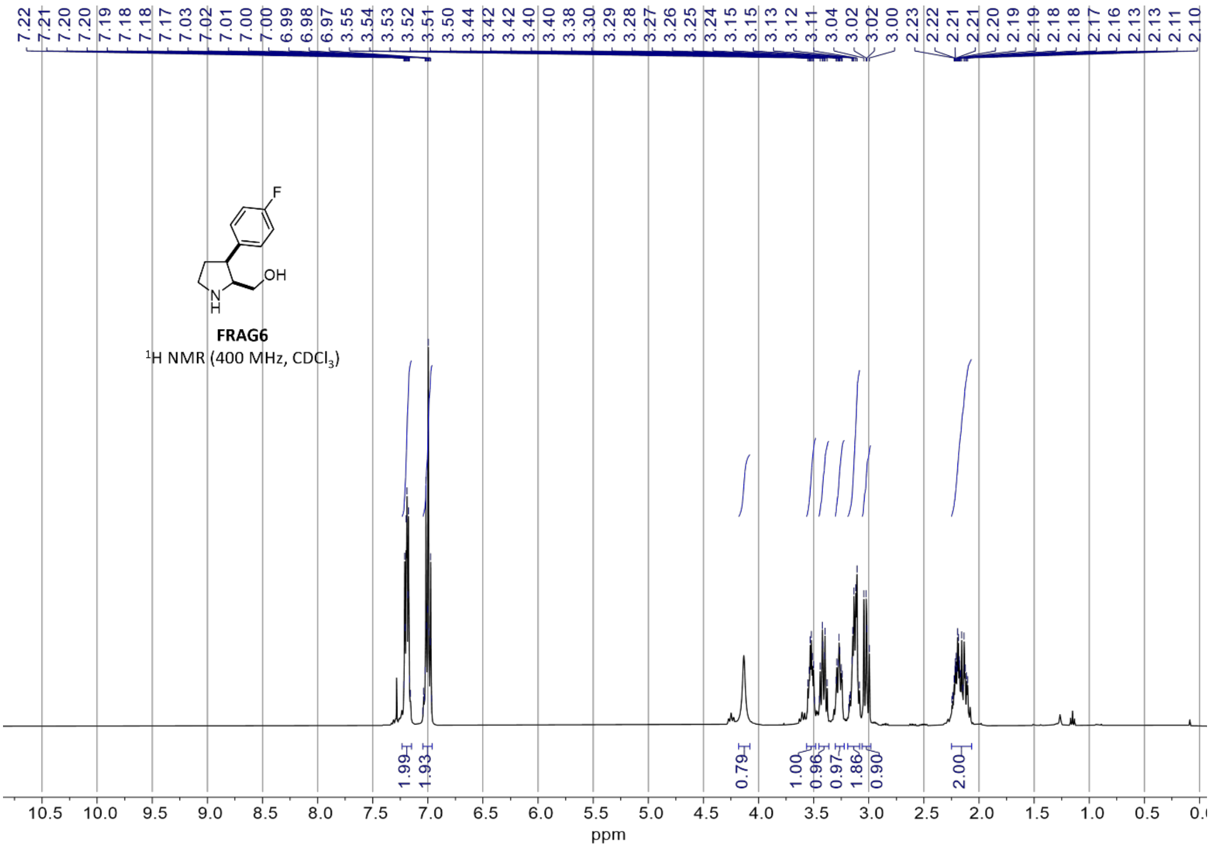

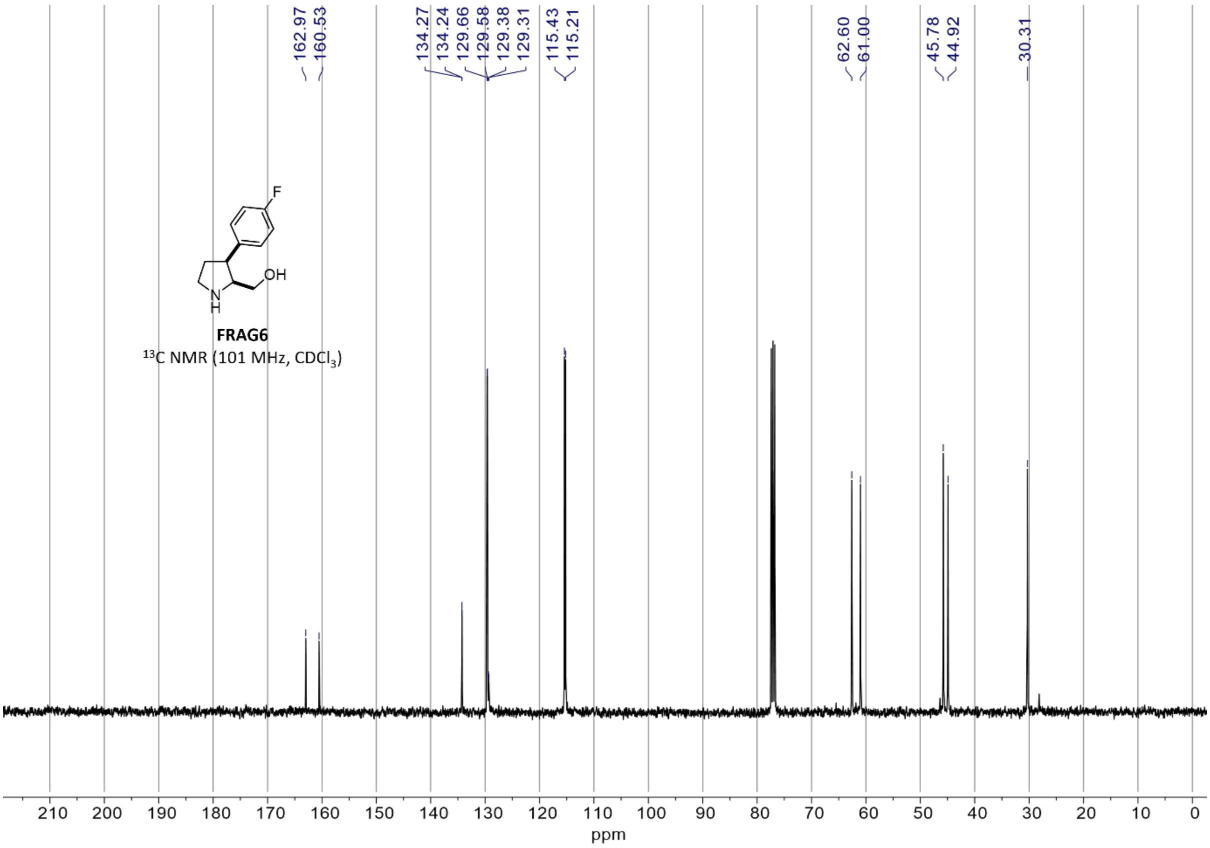

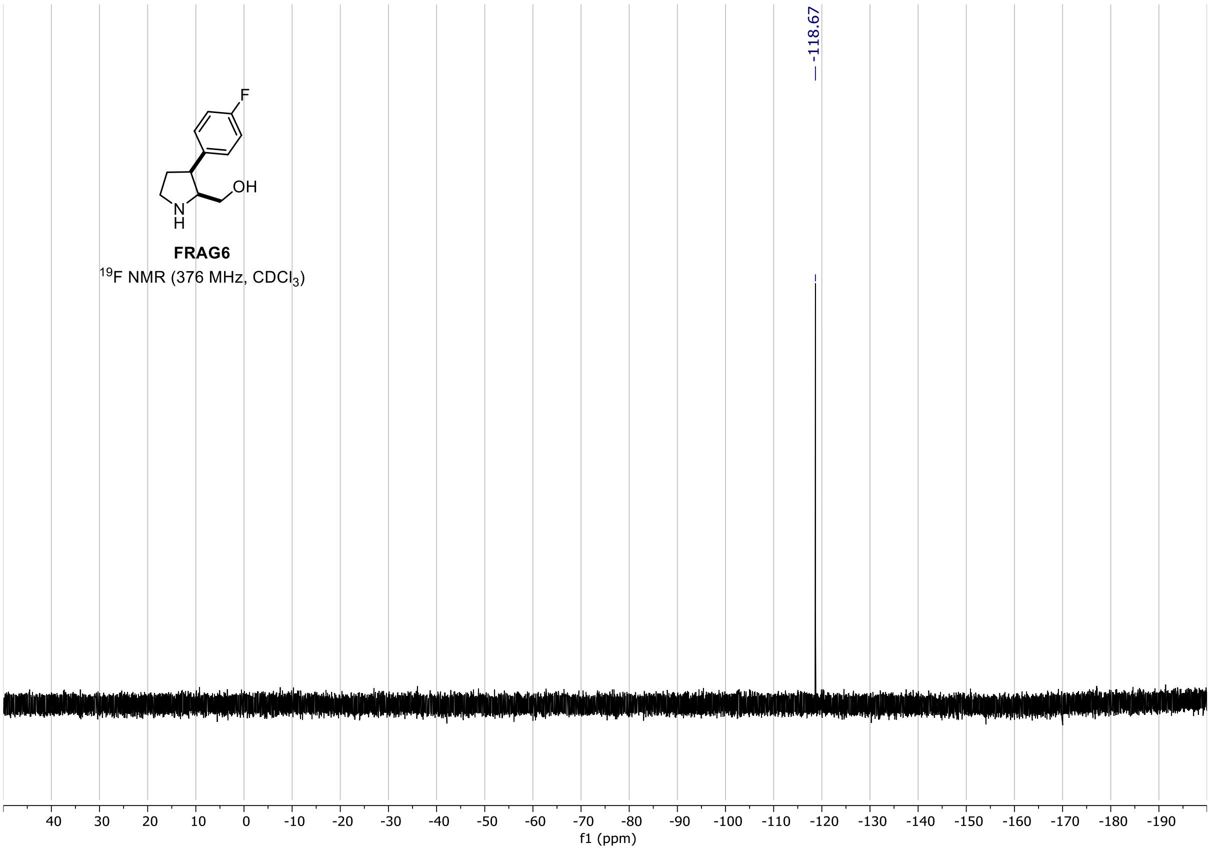

FRAG6

^{19}F NMR (376 MHz, $CDCl_3$)

***cis*-(±)-Benzyl 2-carbamoyl-3-(4-methoxyphenyl)piperidine-1-carboxylate (23a)**

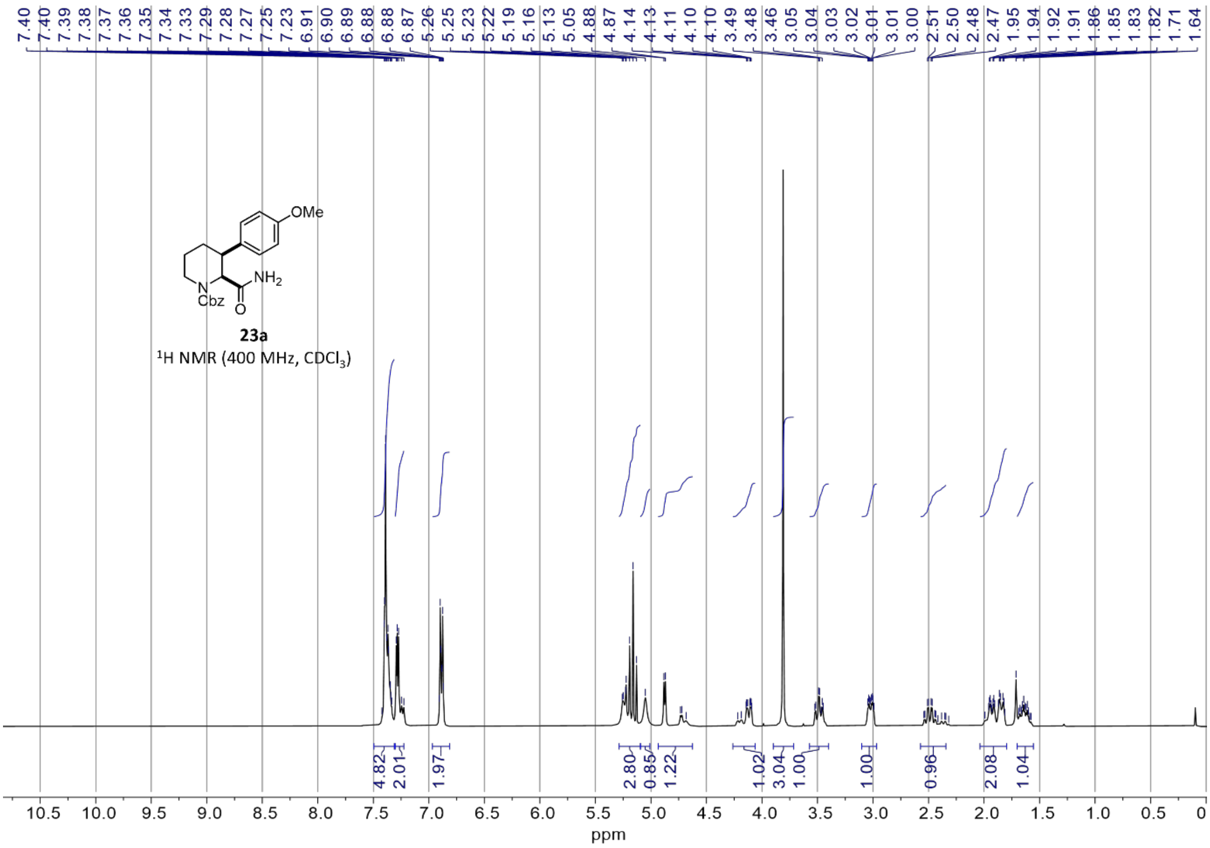

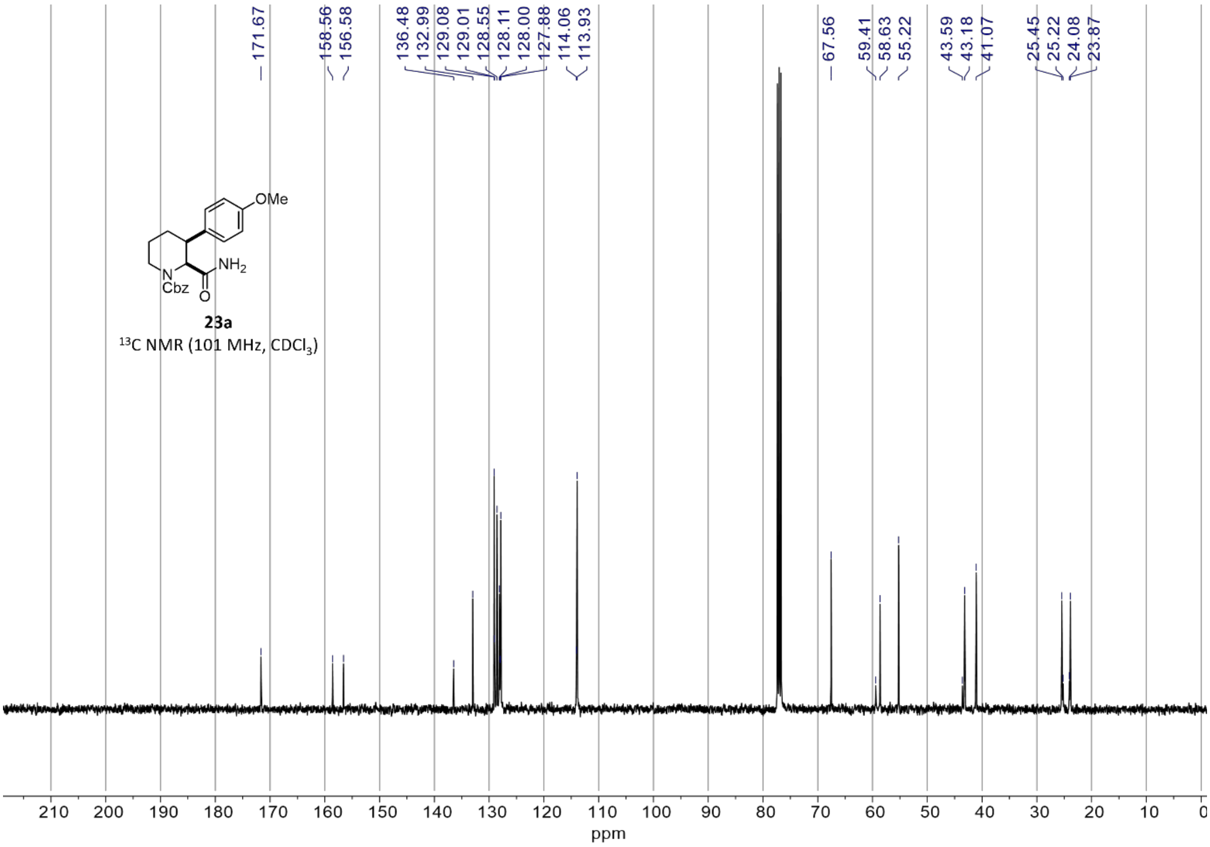

cis-(±)-3-(4-Methoxyphenyl)piperidine-2-carboxamide (FRAG7)

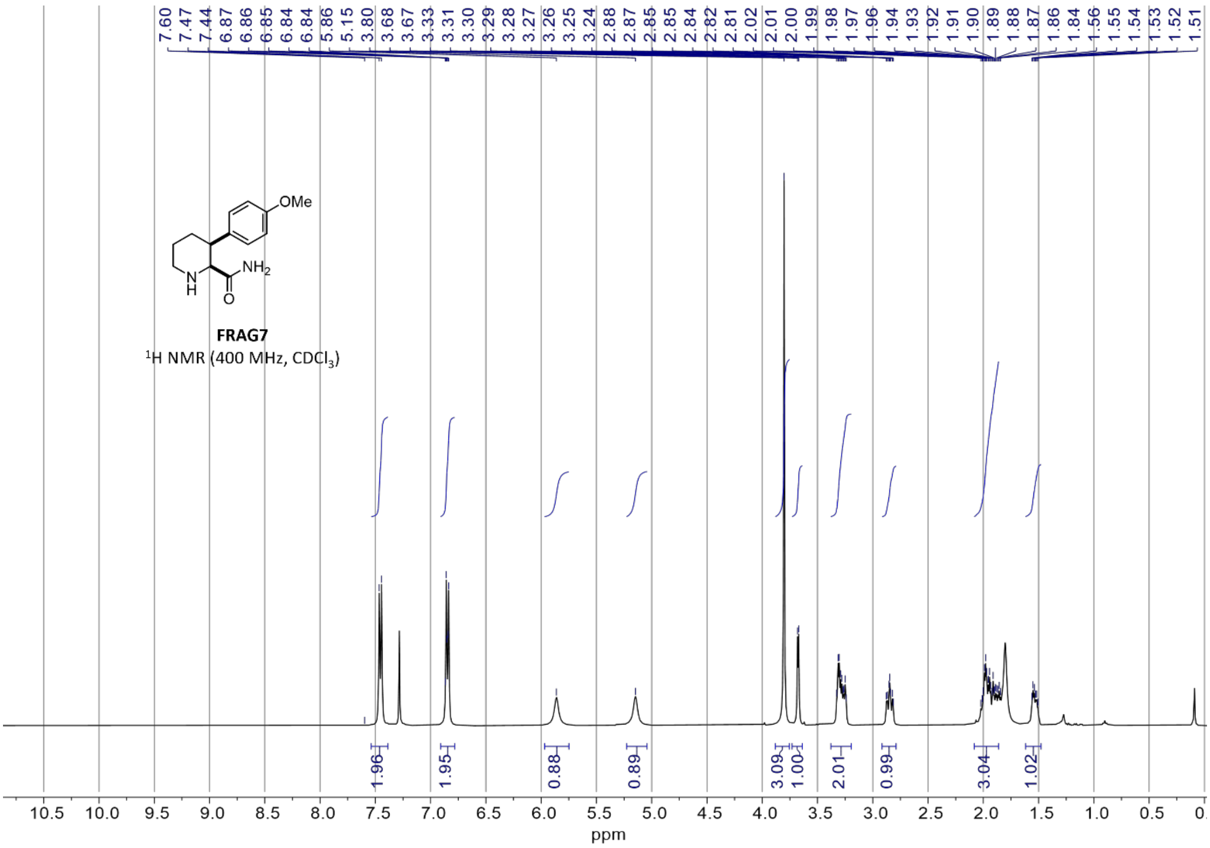

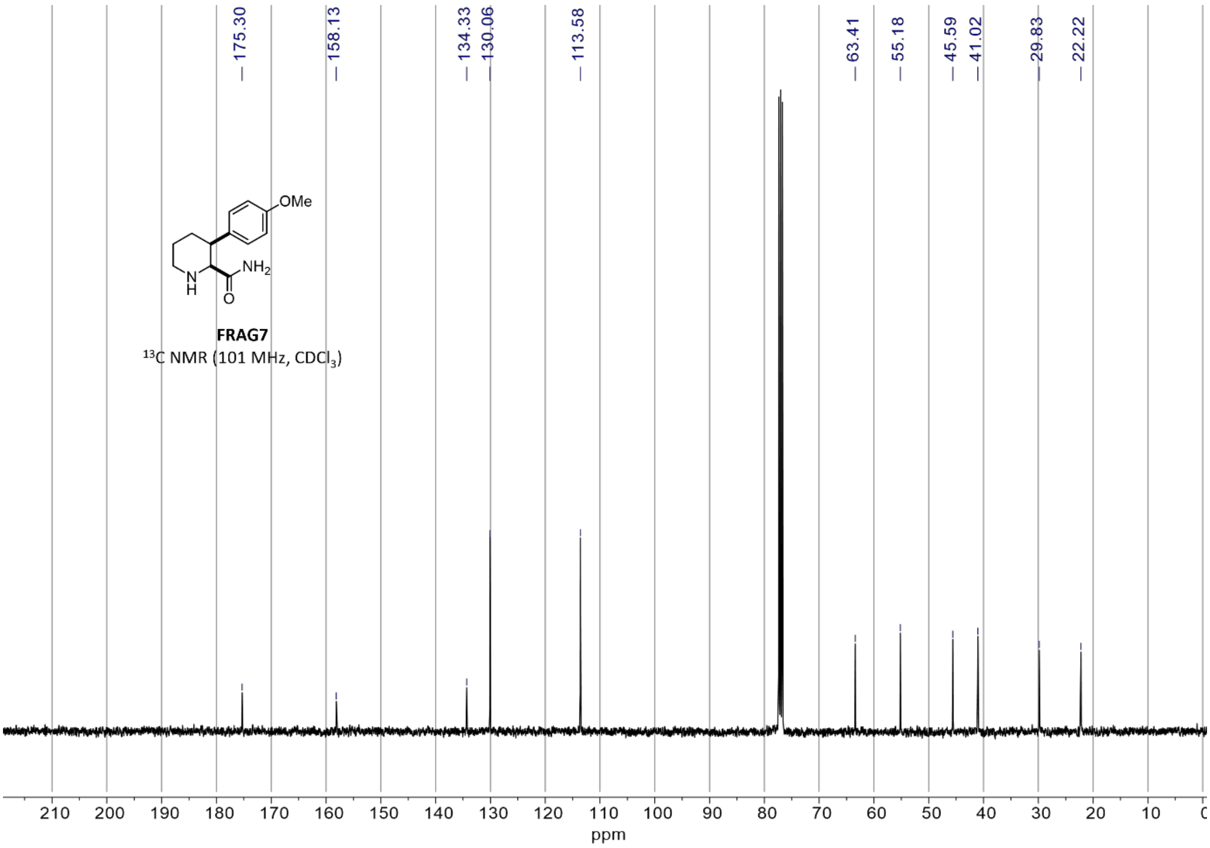

cis-(±)-_tert_-Butyl 3-(4-methoxyphenyl)-4-(quinolin-8-ylcarbamoyl)pyrrolidine-1-carboxylate (6a)

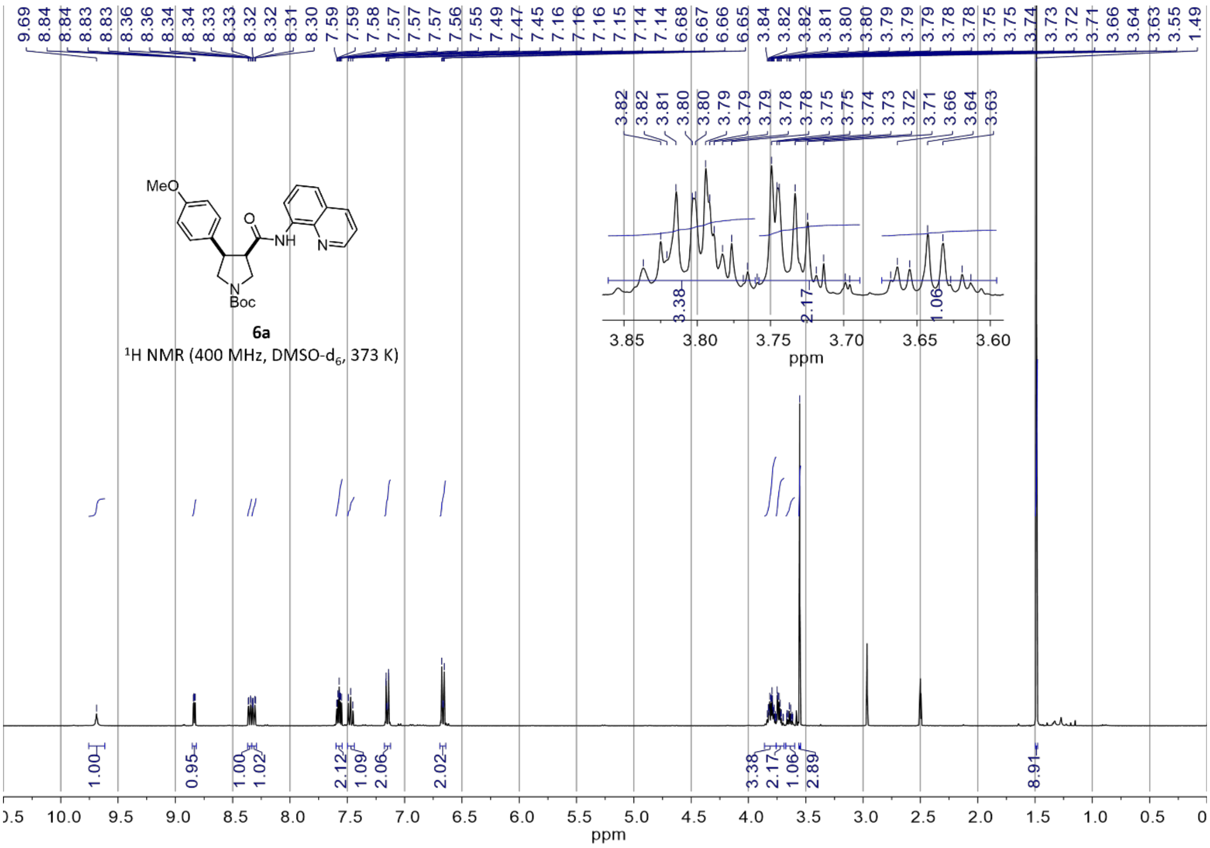

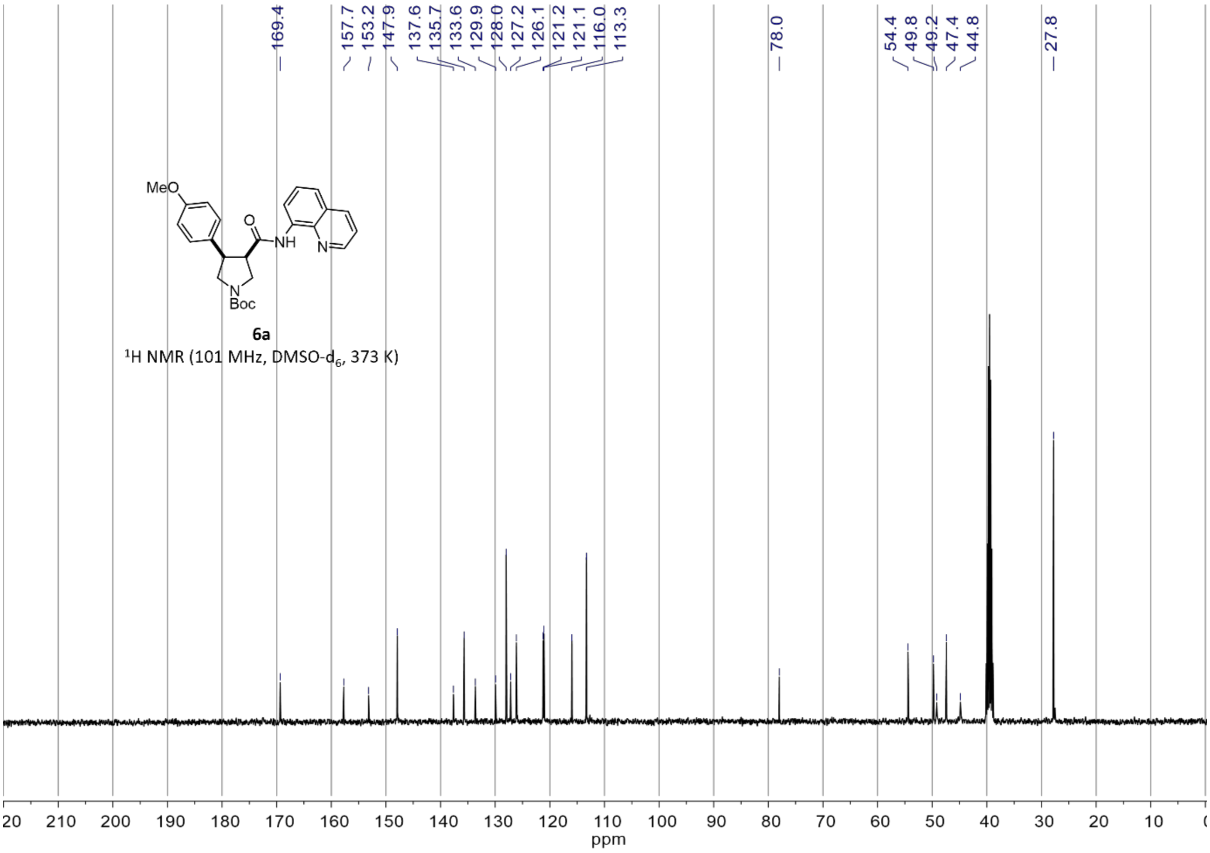

trans-(±)-_tert_-Butyl 3-(4-methoxyphenyl)-4-(quinolin-8-ylcarbamoyl)pyrrolidine-1-carboxylate (7a)

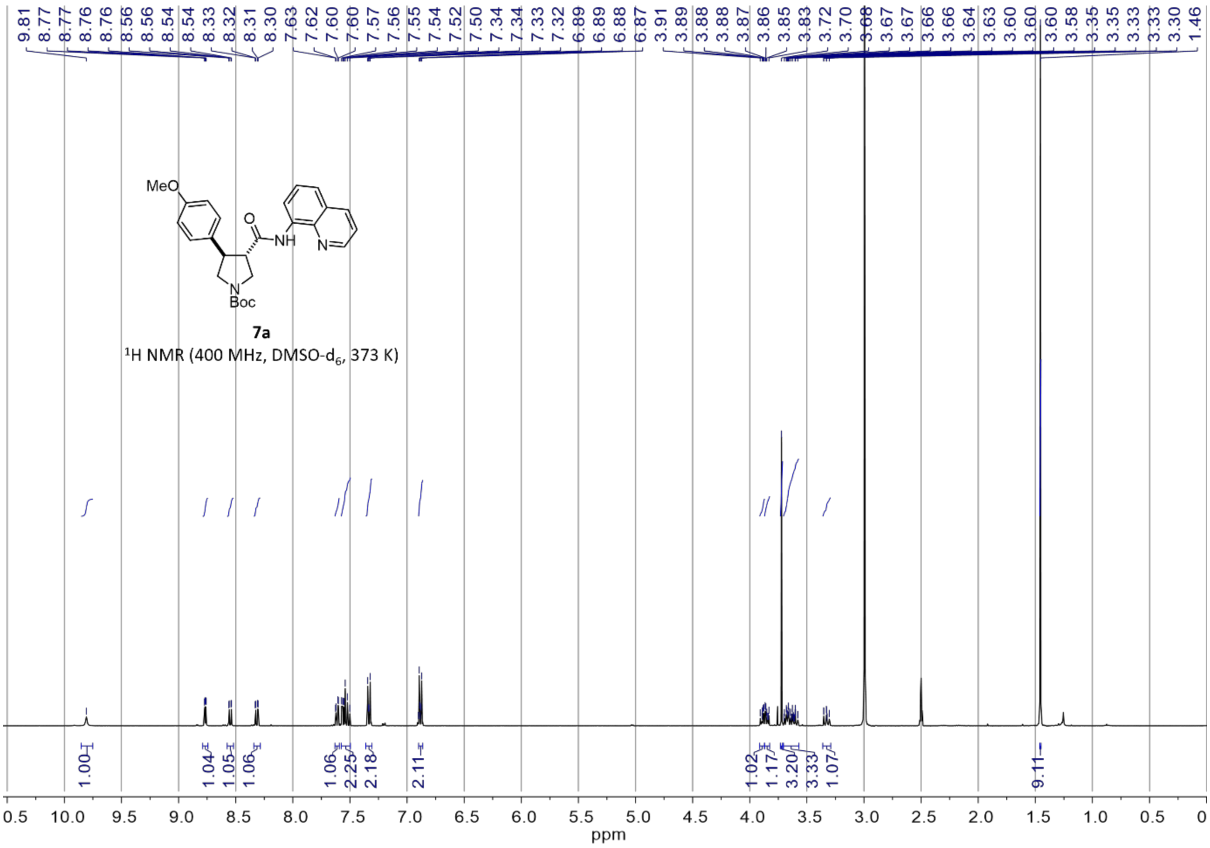

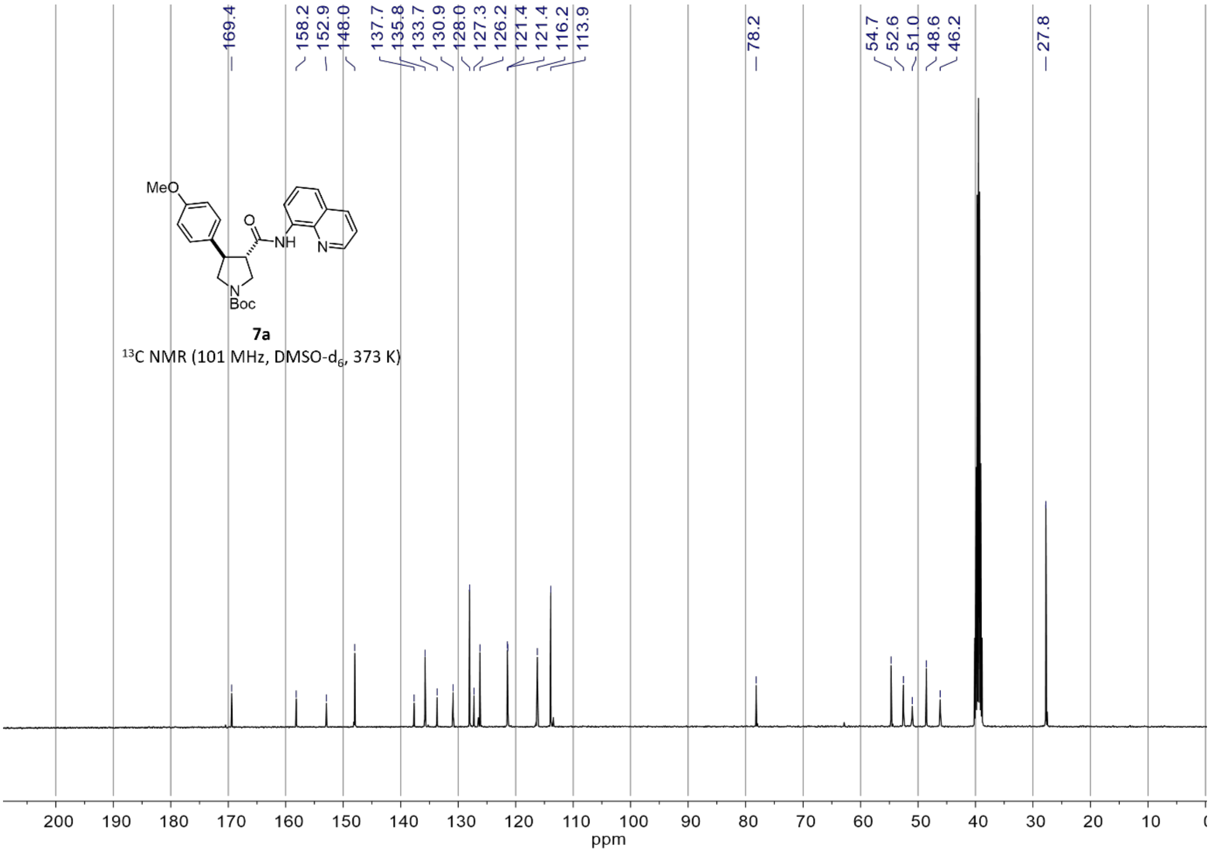

cis-(±)-_tert_-Butyl 3-(4-fluorophenyl)-4-(quinolin-8-ylcarbamoyl)pyrrolidine-1-carboxylate (6b)

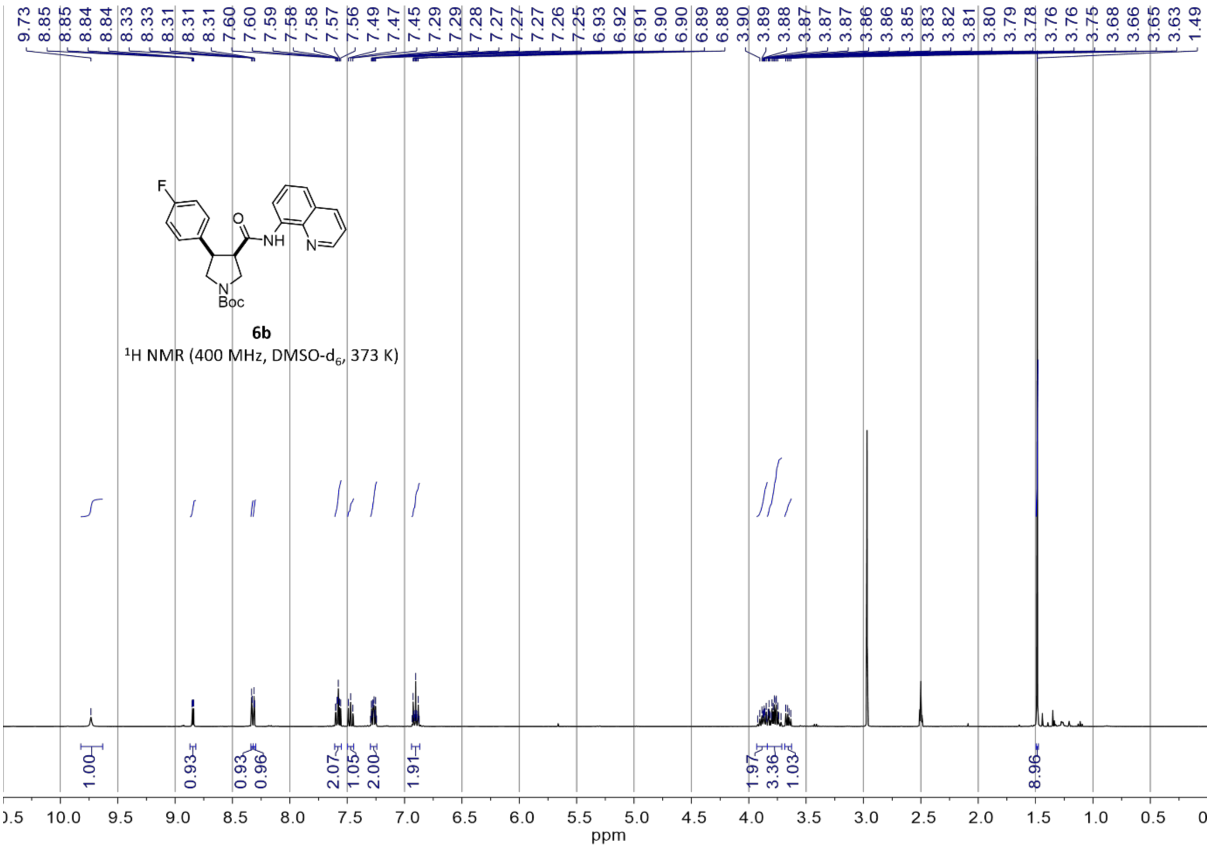

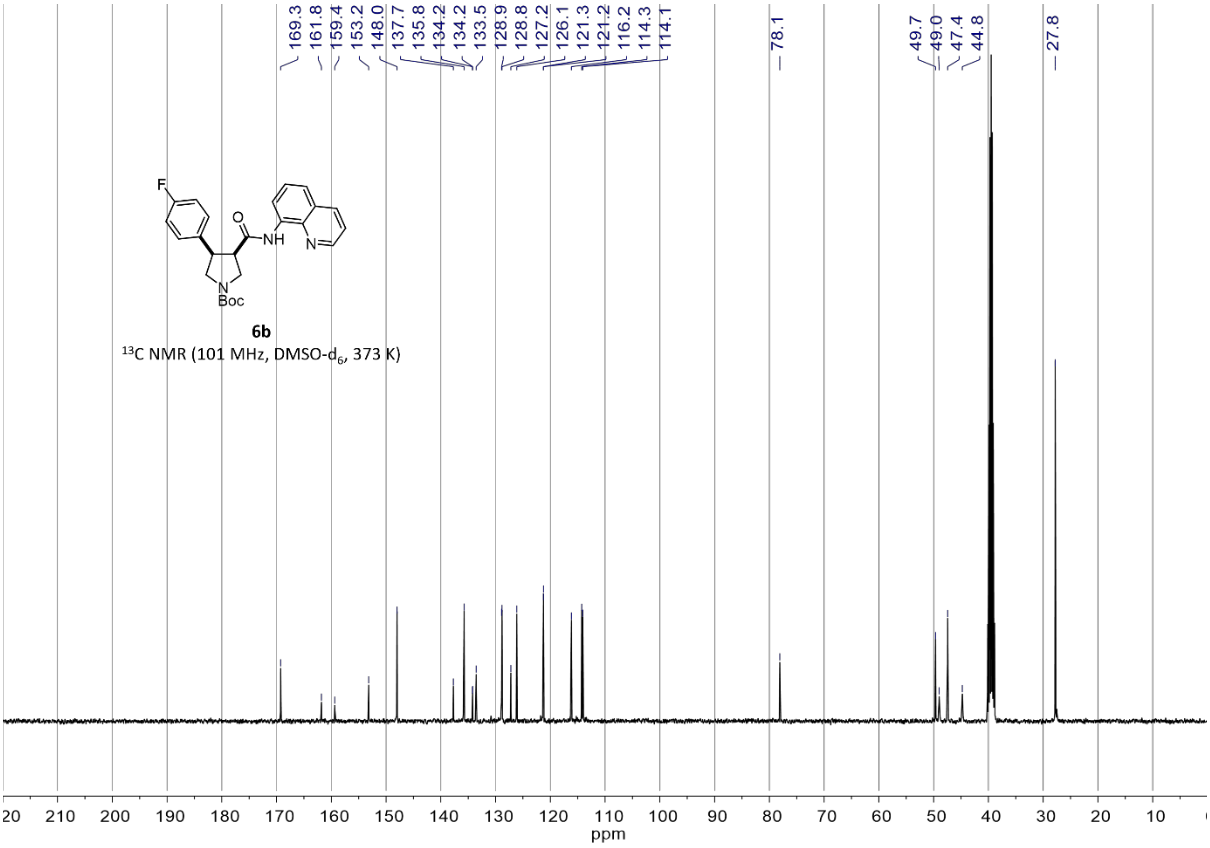

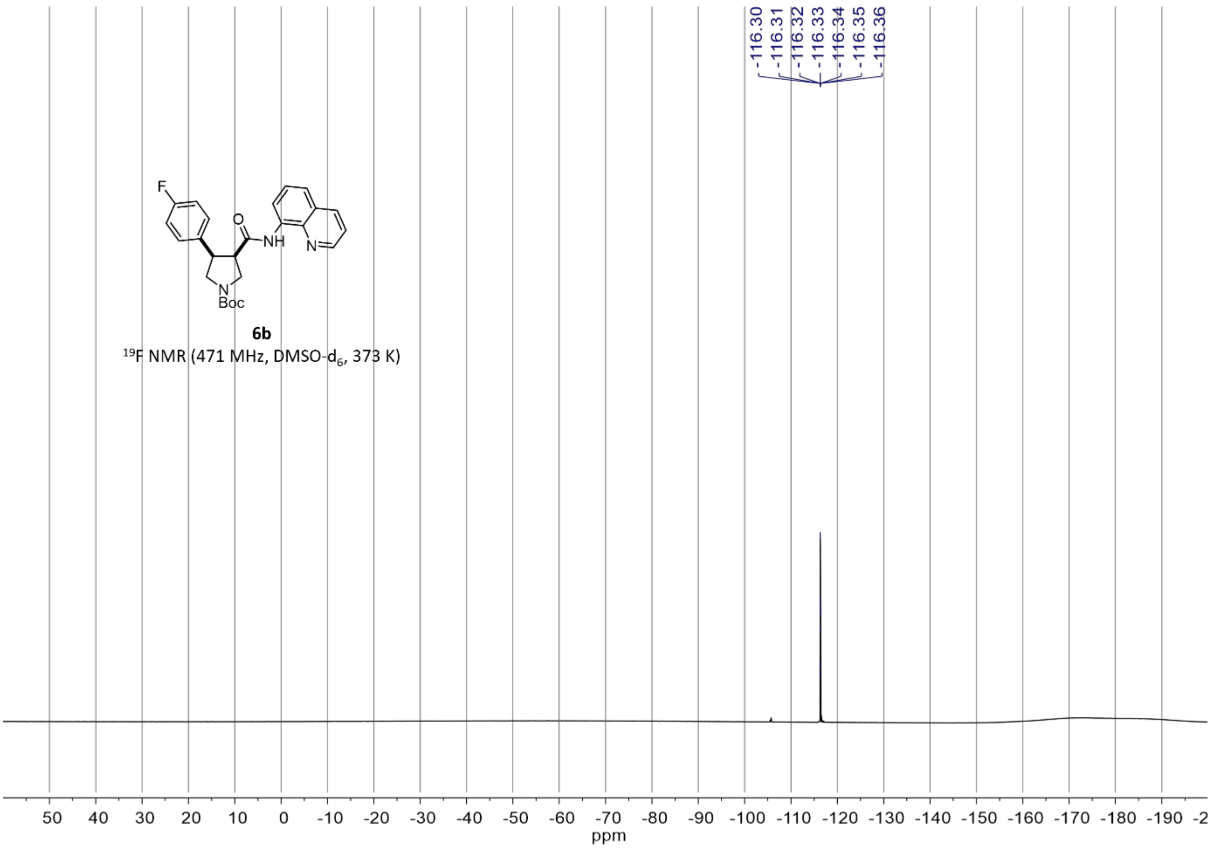

6b

^{19}F NMR (471 MHz, DMSO-d$_6$, 373 K)

trans-(±)-_tert_-Butyl 3-(4-fluorophenyl)-4-(quinolin-8-ylcarbamoyl)pyrrolidine-1-carboxylate (7b)

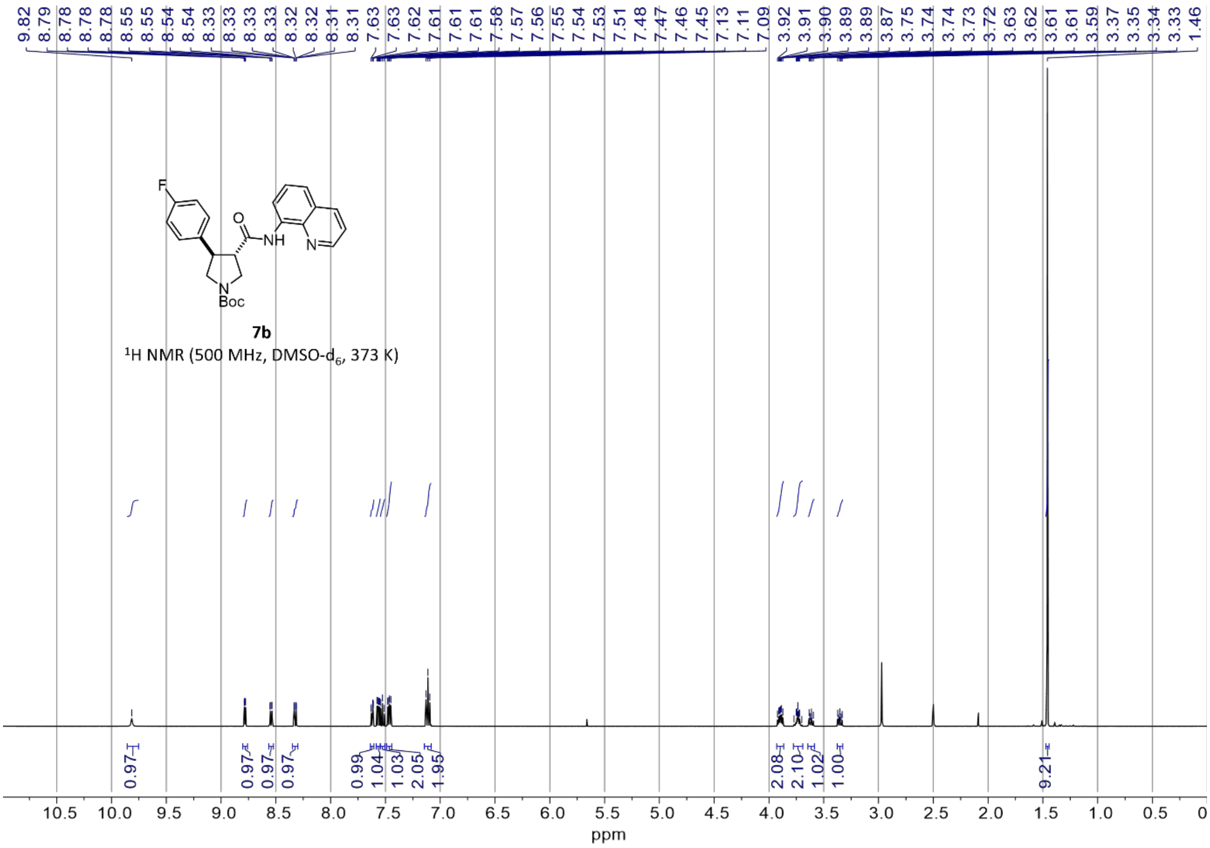

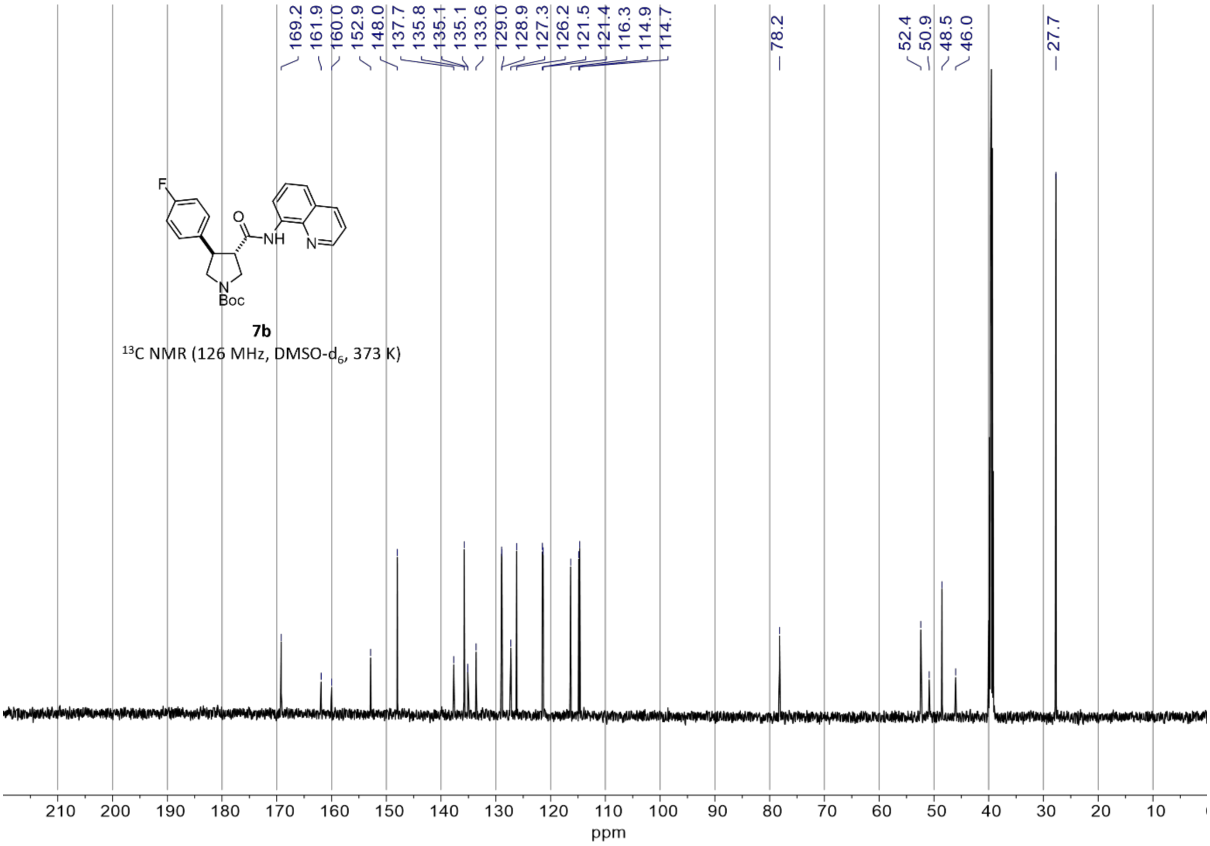

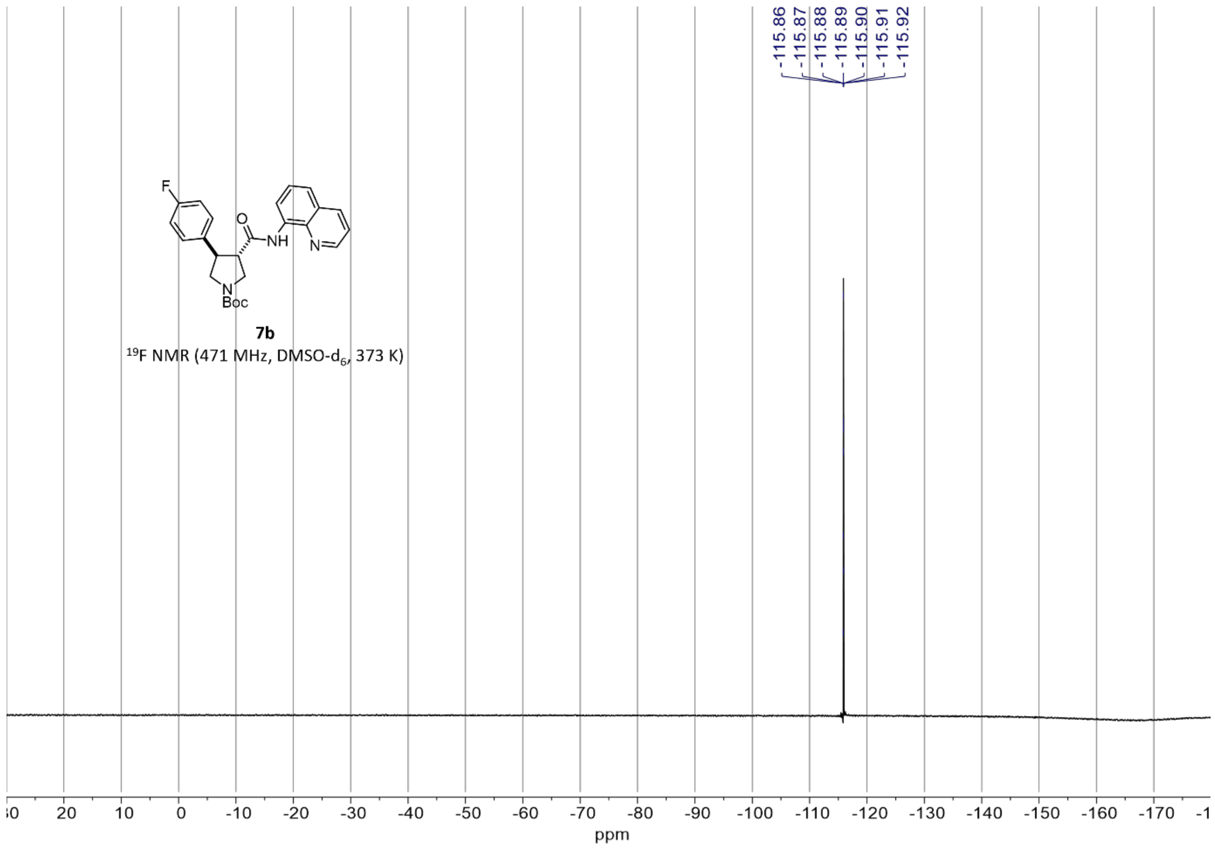
-115.86
-115.87
-115.88
-115.89
-115.90
-115.91
-115.92
F
O
NH
N
N
Boc
7b
19F NMR (471 MHz, DMSO-d6, 373 K)
20 10 0 -10 -20 -30 -40 -50 -60 -70 -80 -90 -100 -110 -120 -130 -140 -150 -160 -170
ppm

***cis*-(±)-*tert*-Butyl 4-(4-methoxyphenyl)-3-(quinolin-8-ylcarbamoyl)piperidine-1-carboxylate (9a)**

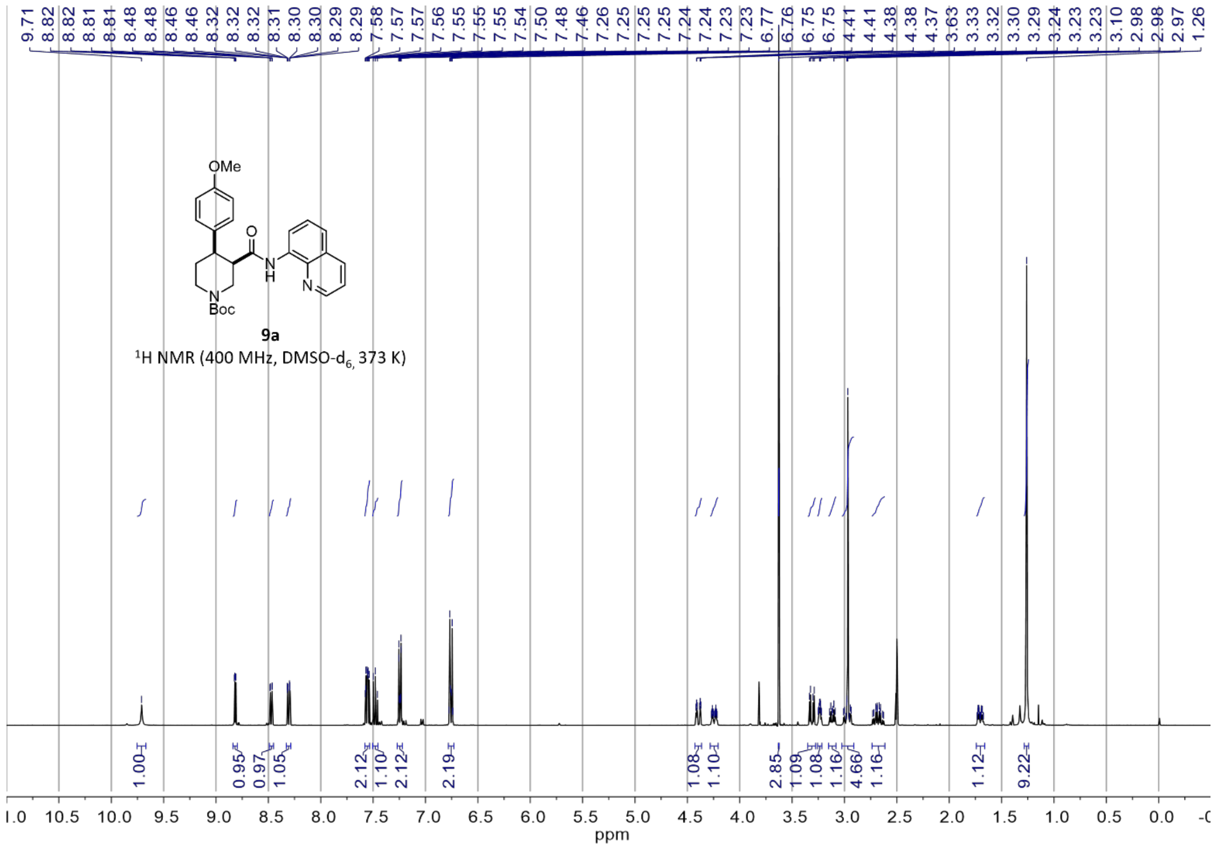

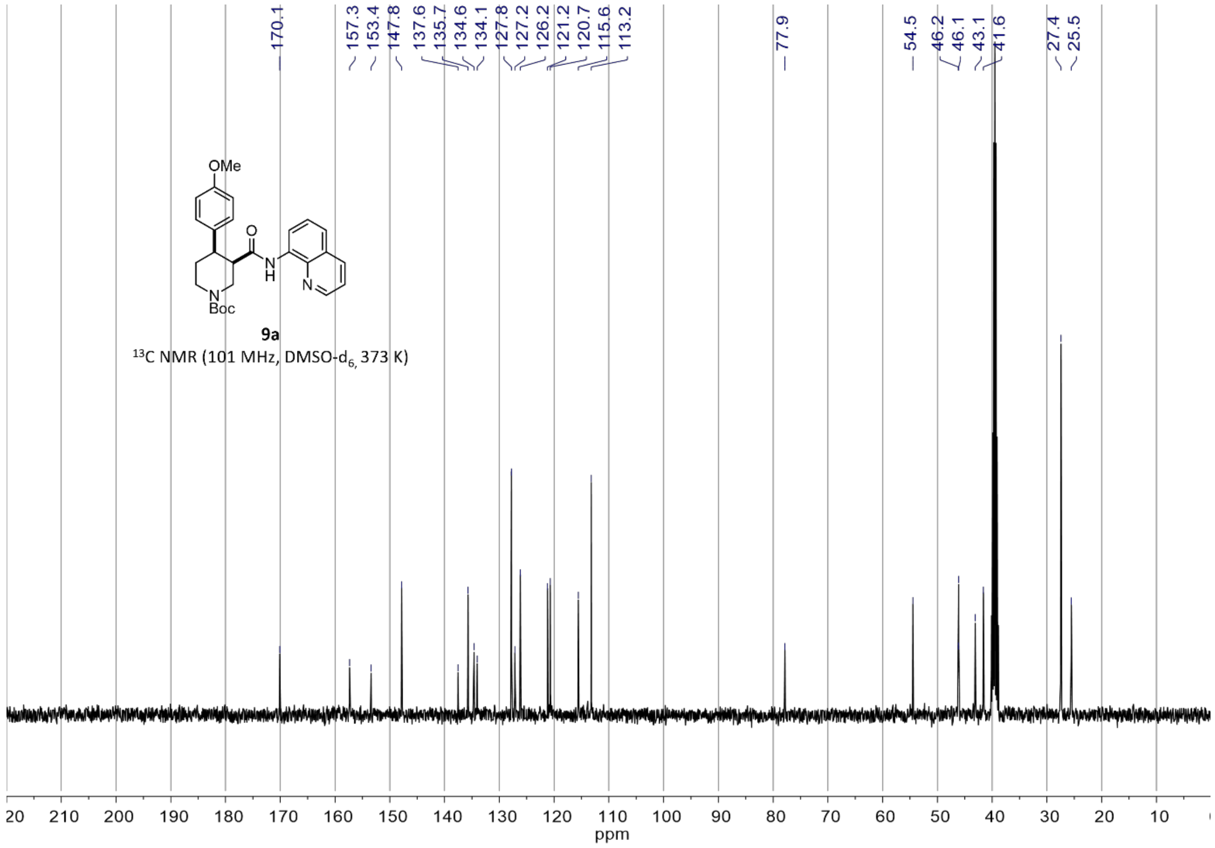

***trans*-(±)-*tert*-Butyl 4-(4-methoxyphenyl)-3-(quinolin-8-ylcarbamoyl)piperidine-1-carboxylate (10a)**

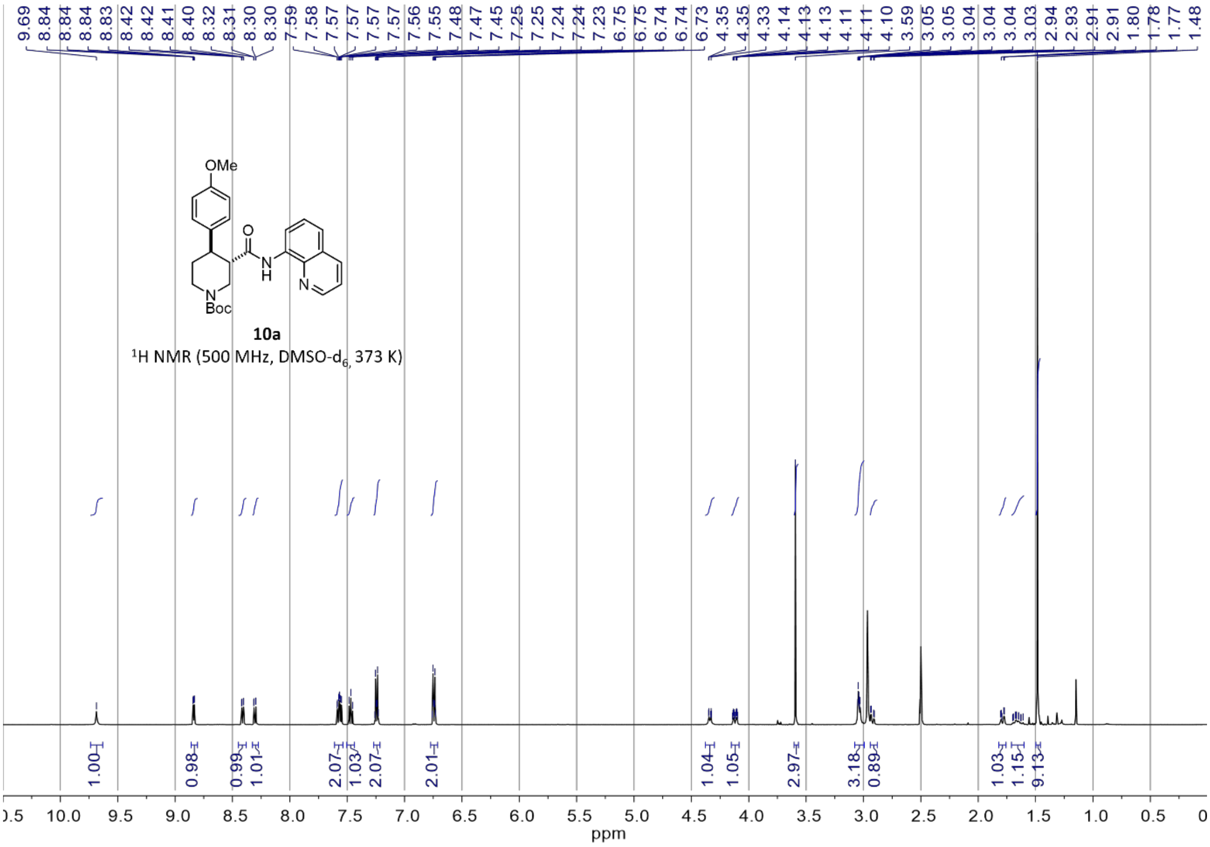

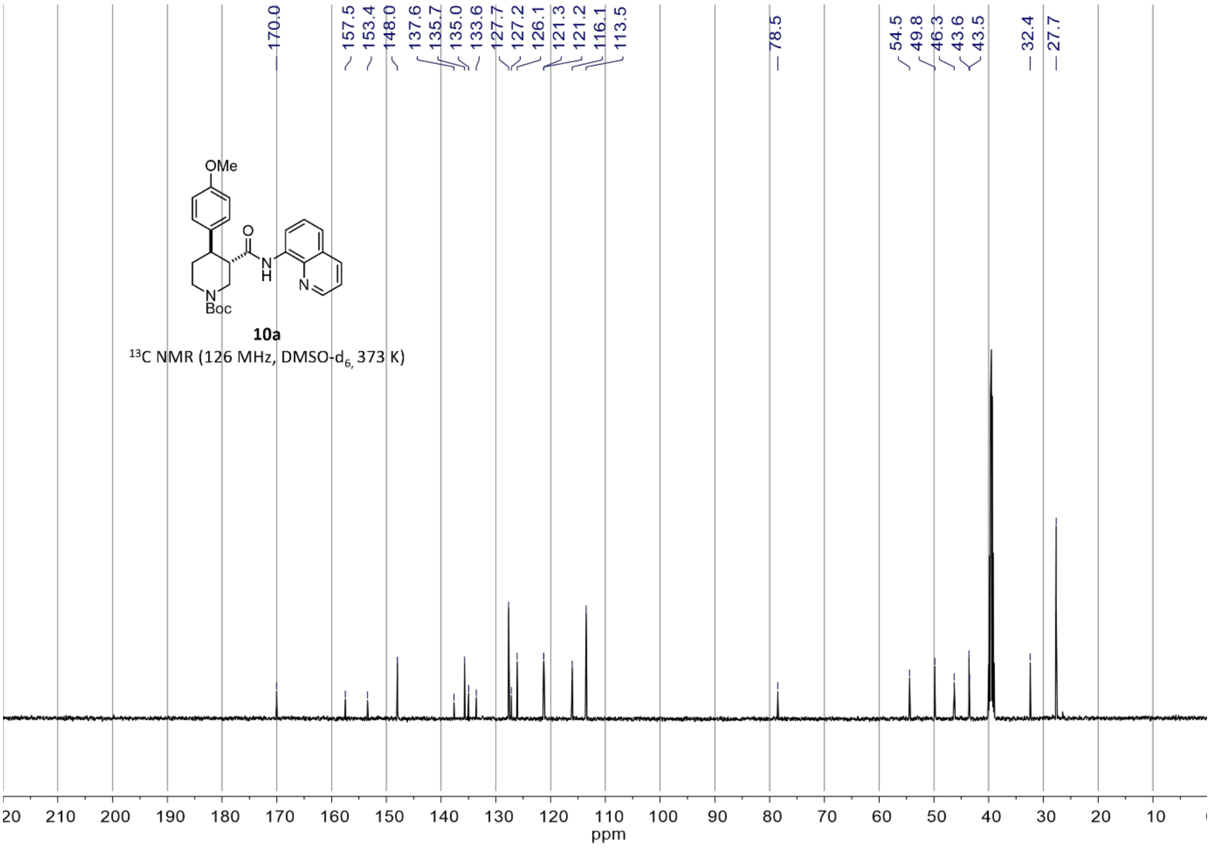

trans-(±)-_tert_-Butyl 4-(4-fluorophenyl)-3-(quinolin-8-ylcarbamoyl)piperidine-1-carboxylate (10b)

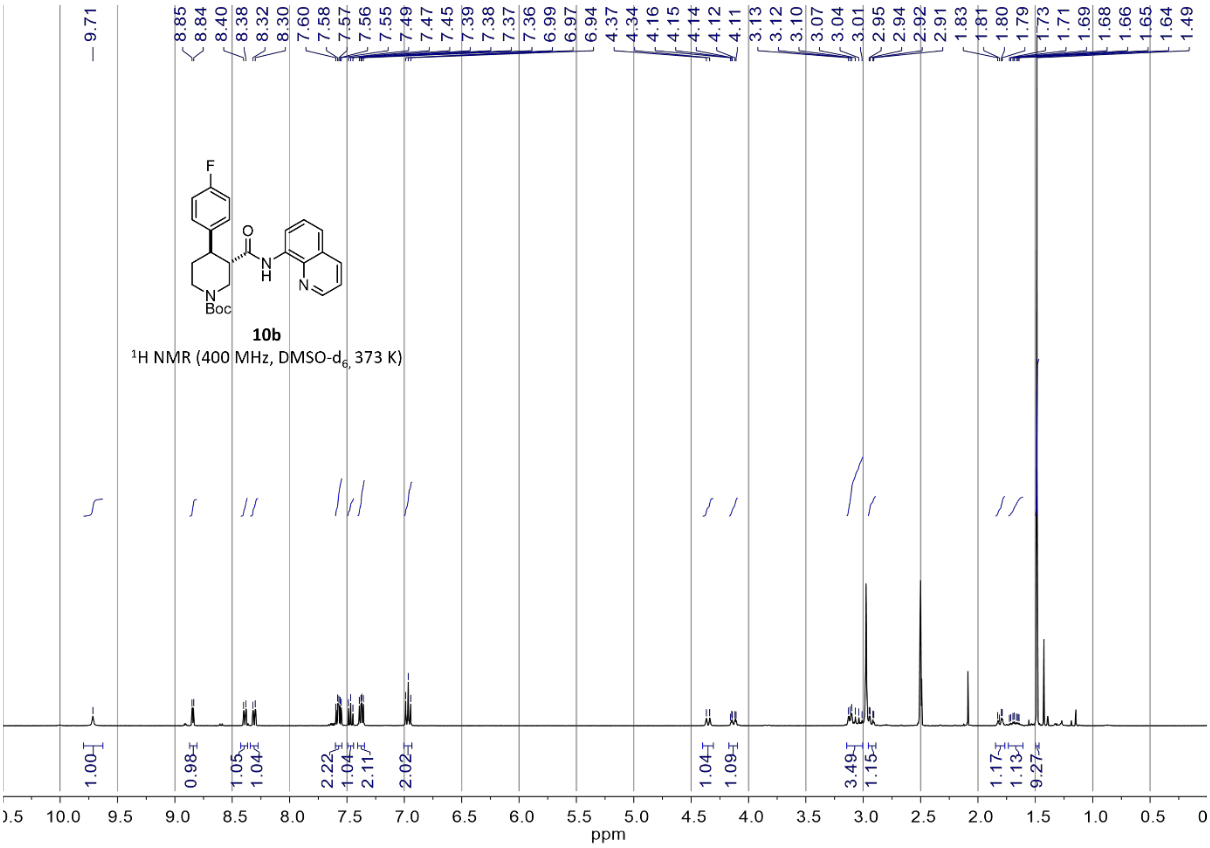

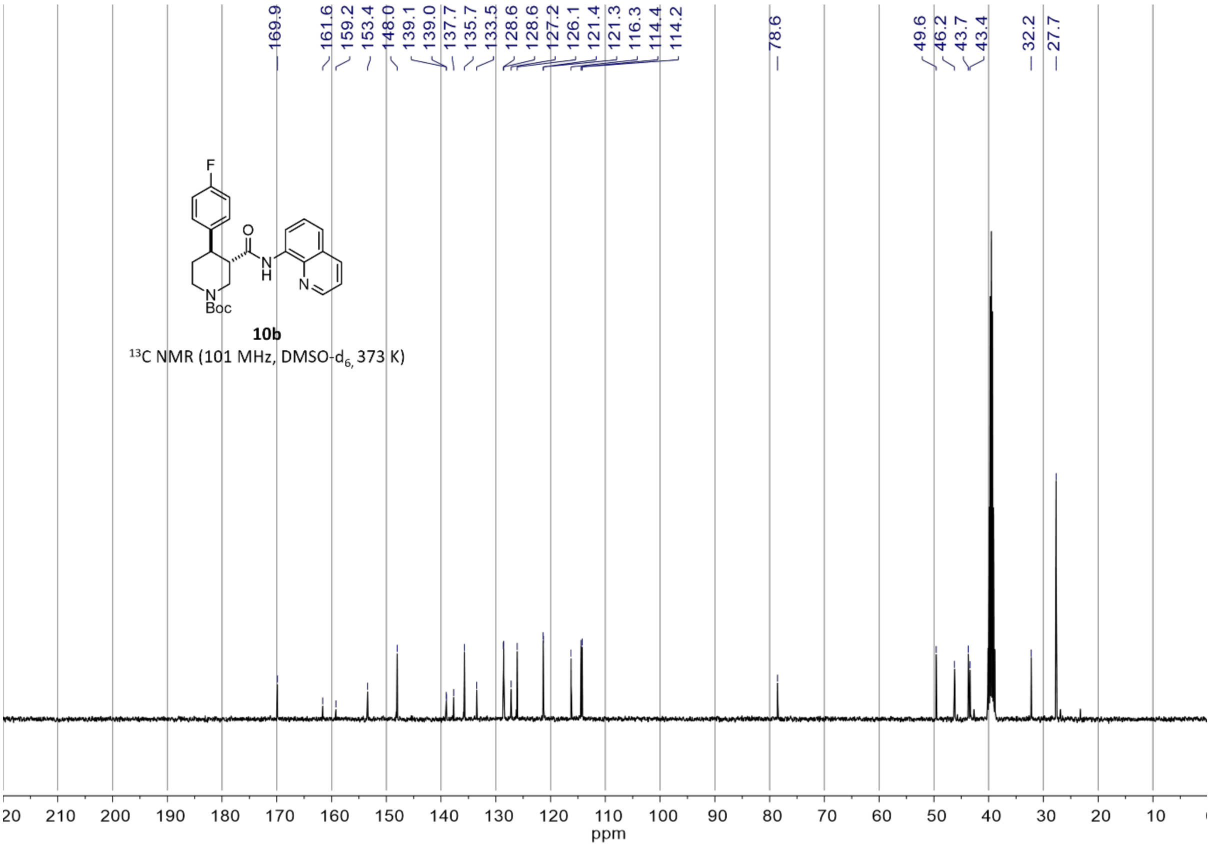

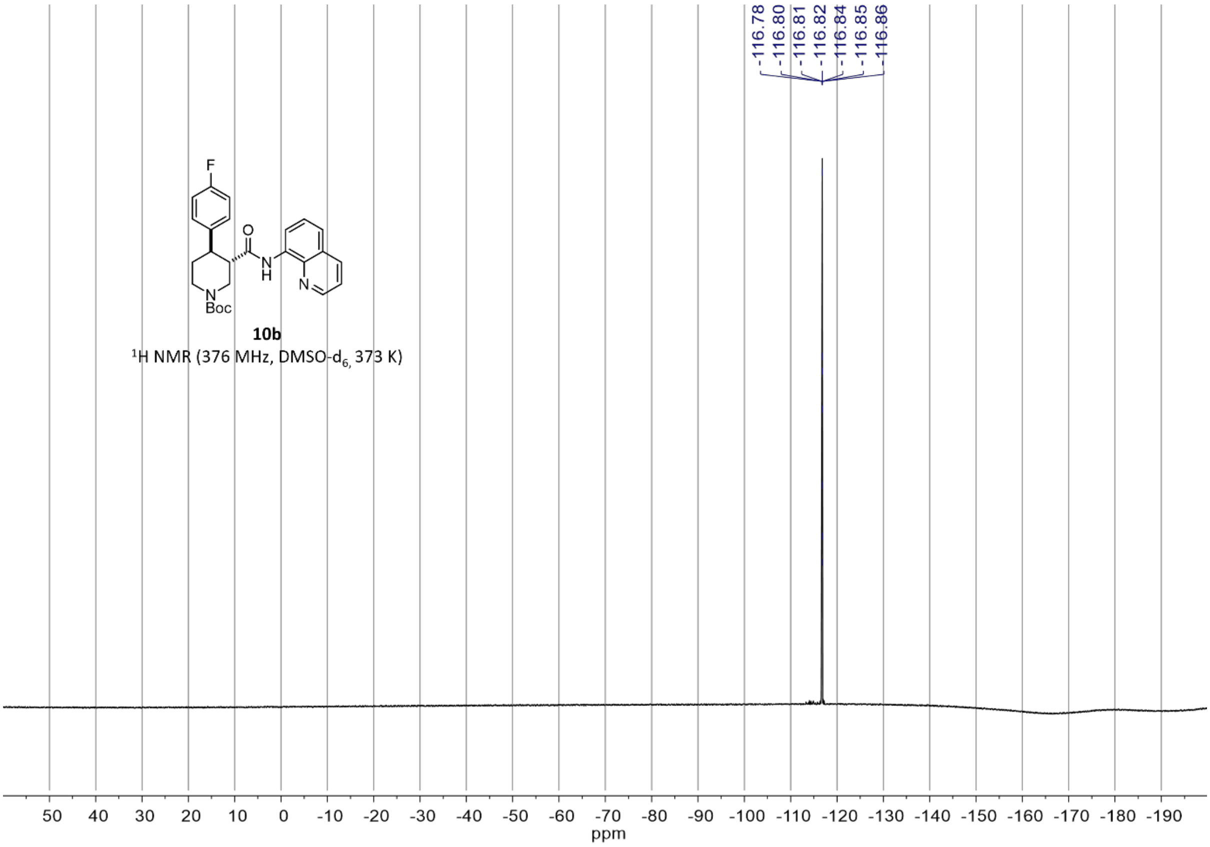
-116.78
-116.80
-116.81
-116.82
-116.84
-116.85
-116.86
F
O
N
H
N
N
Boc
10b
^{1}H NMR (376 MHz, DMSO-d$_{6,}$ 373 K)
50 40 30 20 10 0 -10 -20 -30 -40 -50 -60 -70 -80 -90 -100 -110 -120 -130 -140 -150 -160 -170 -180 -190
ppm

cis-(±)-1-(tert-Butoxycarbonyl)-4-(4-methoxyphenyl)pyrrolidine-3-carboxylic acid (27a)

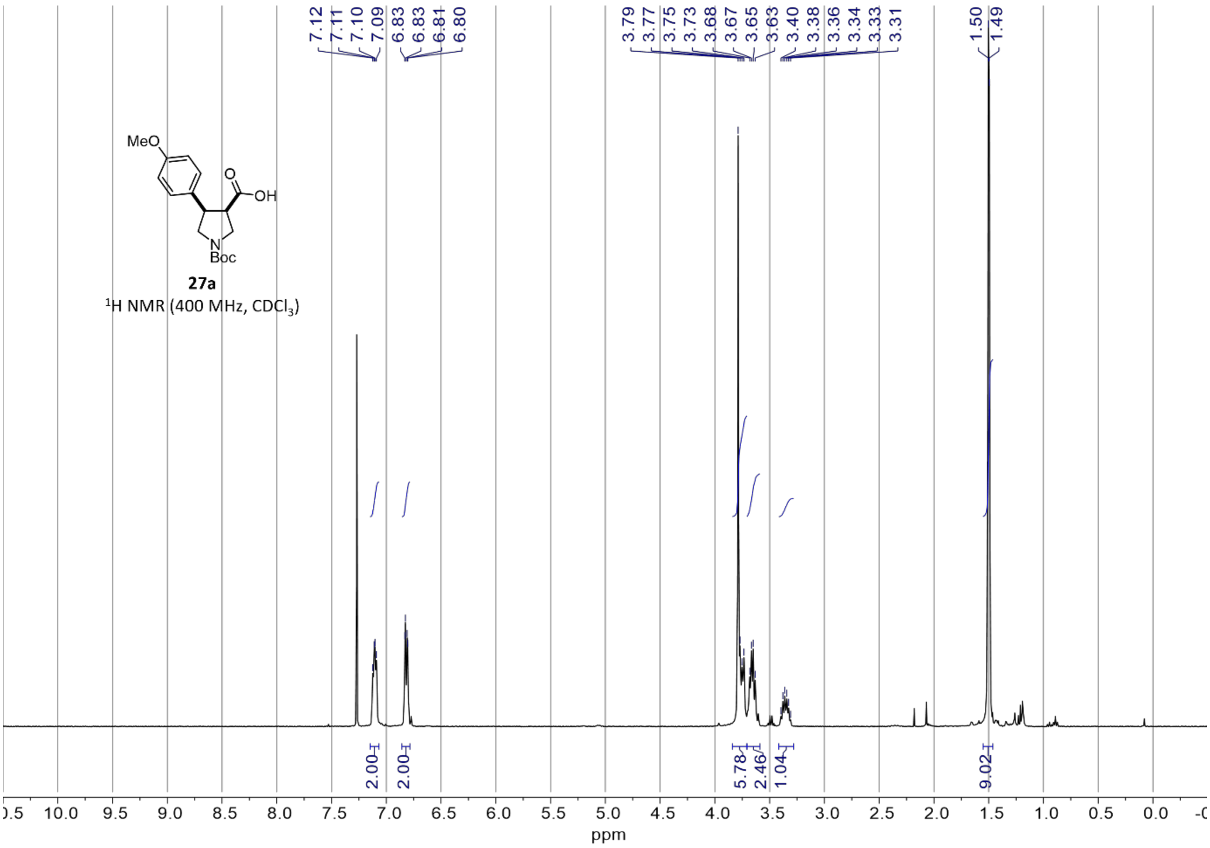

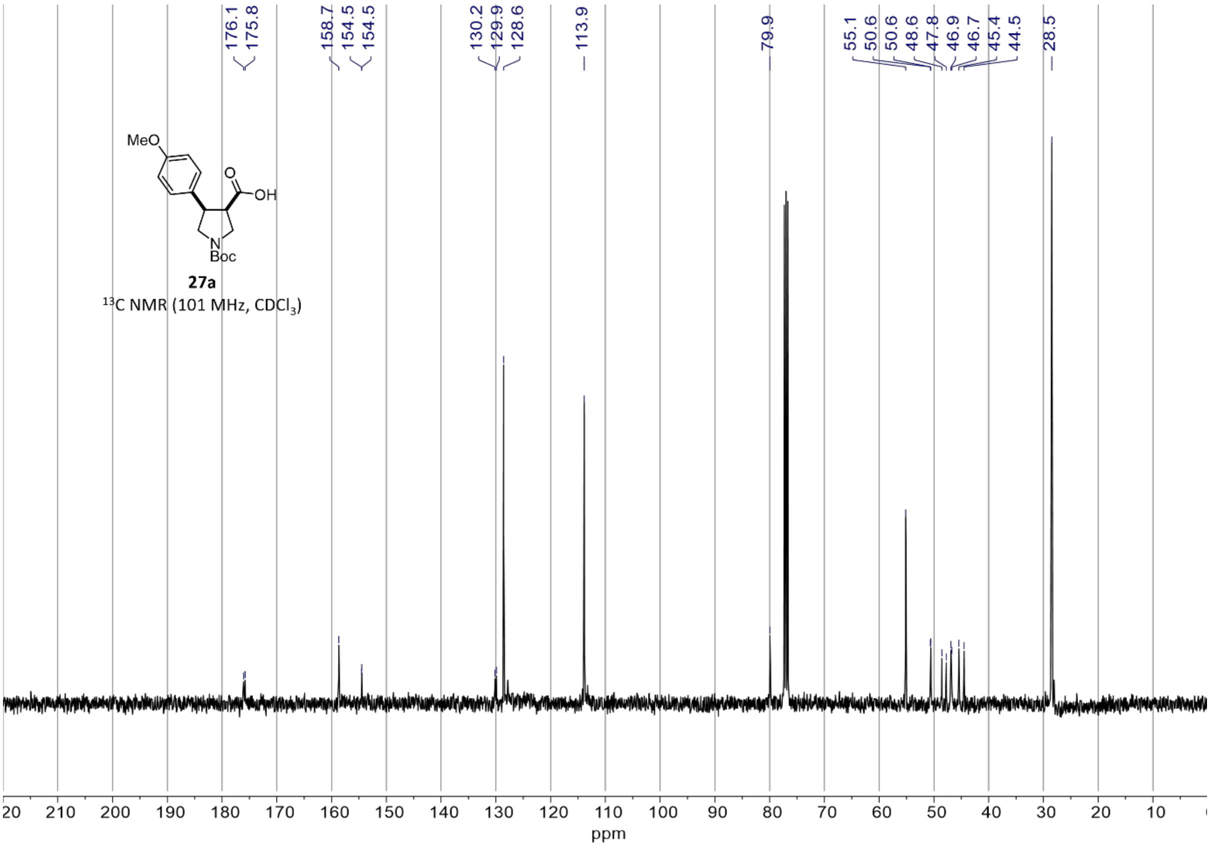

***cis*-(±)-1-(*tert*-Butoxycarbonyl)-4-(4-fluorophenyl)pyrrolidine-3-carboxylic acid (27b)**

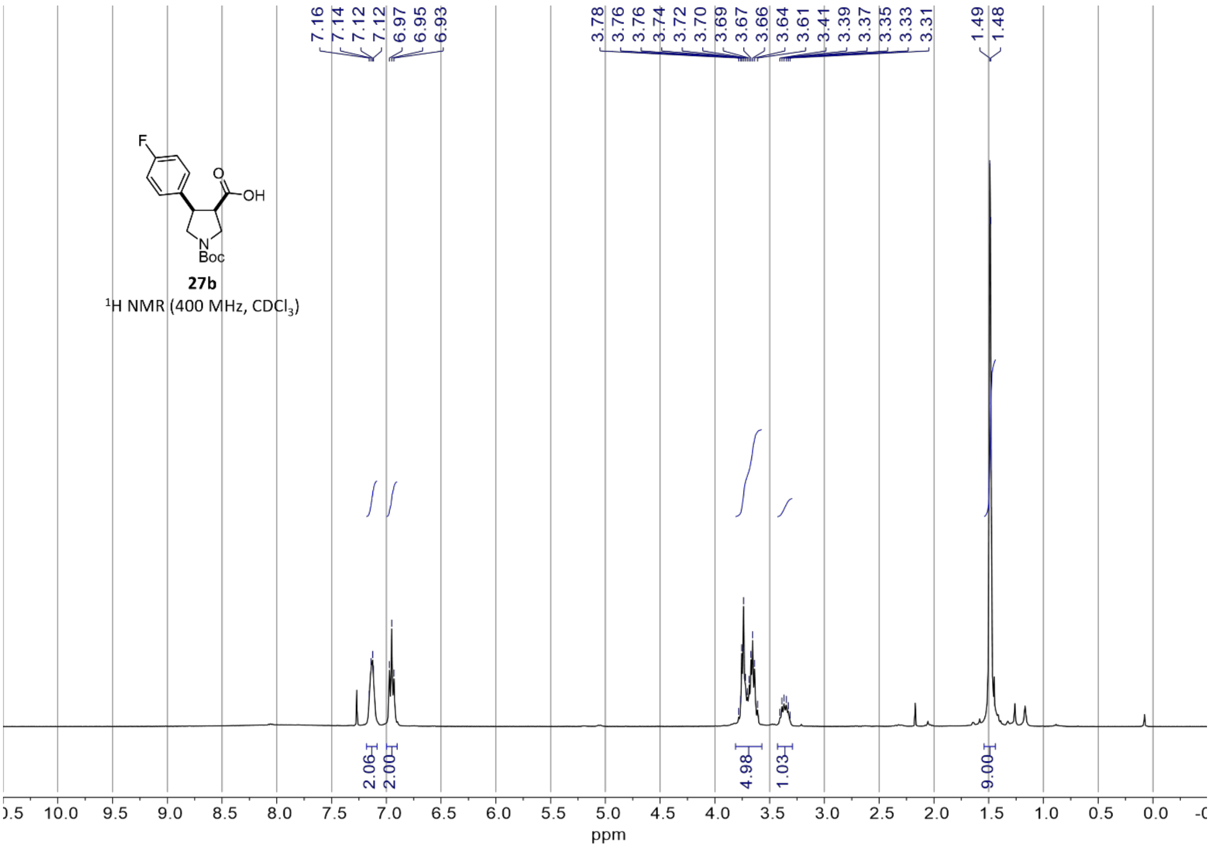

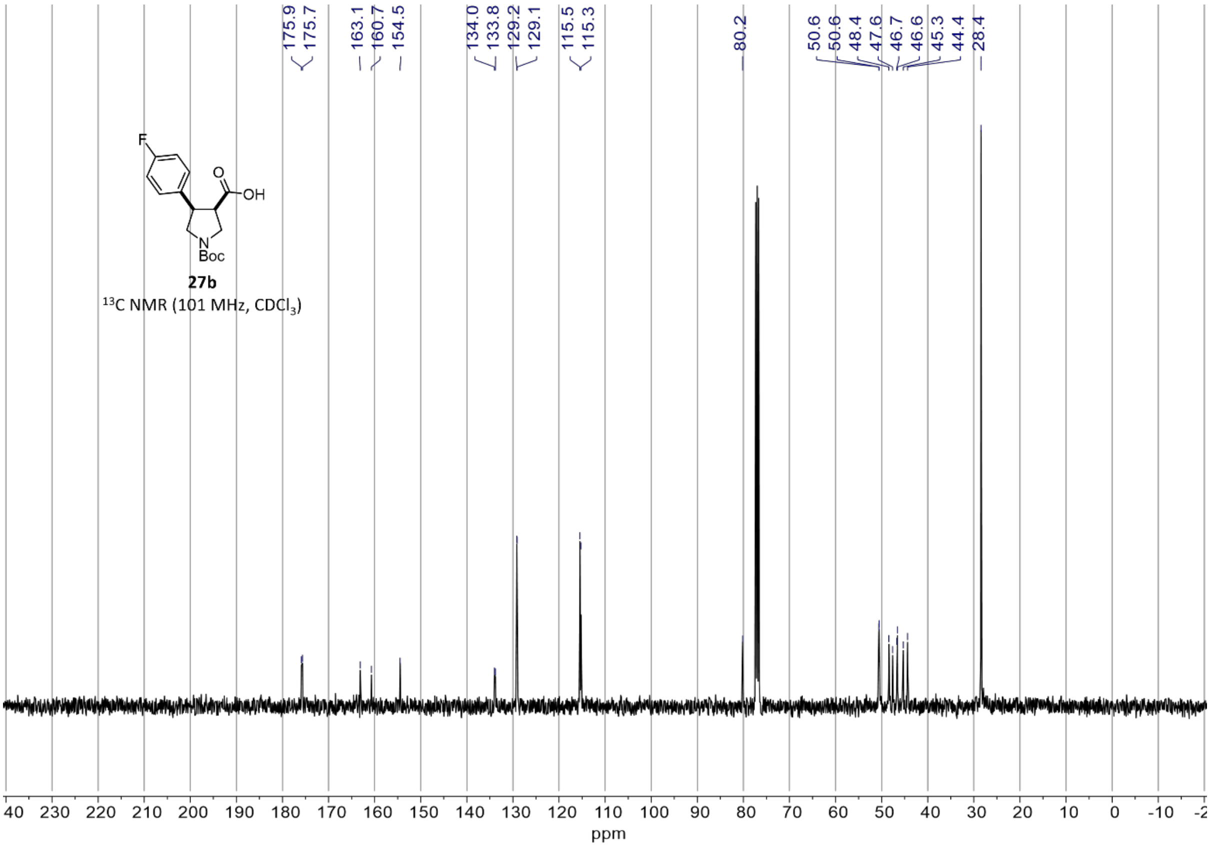

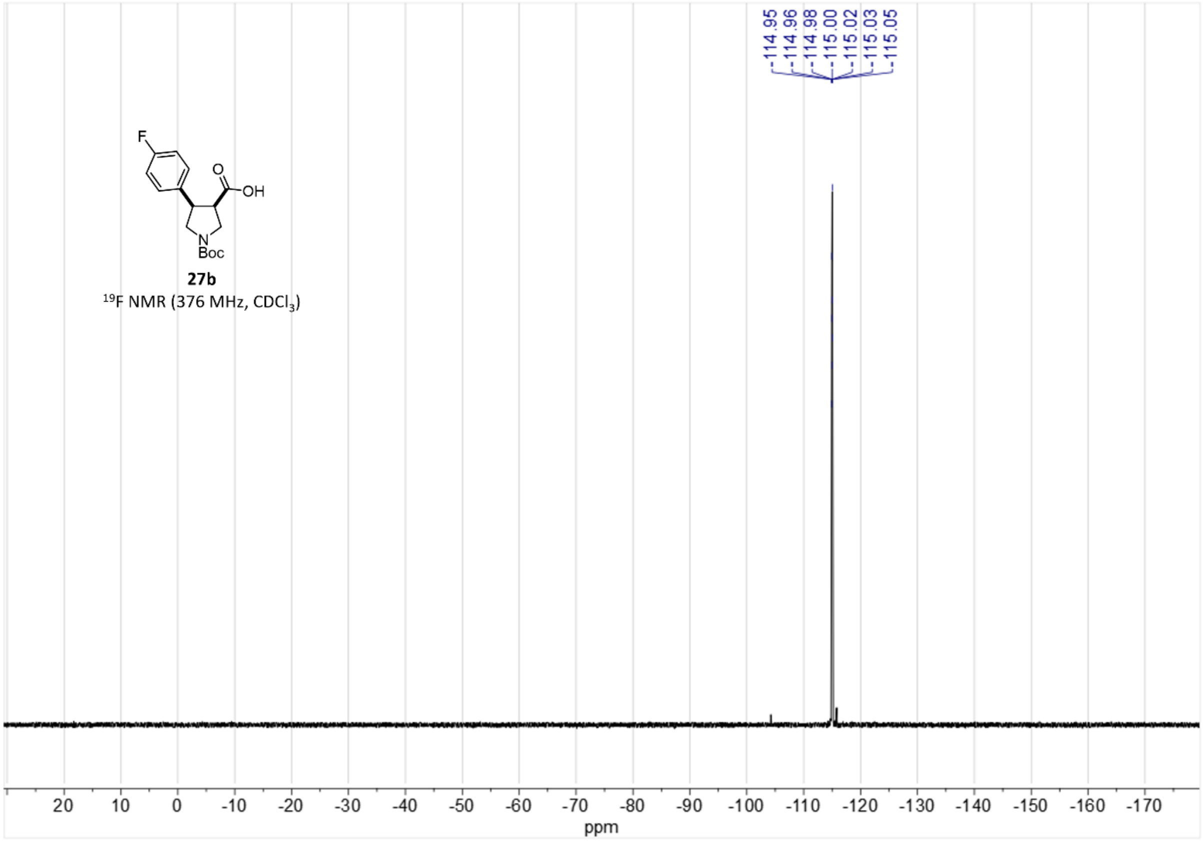
-114.95
-114.96
-114.98
-115.00
-115.02
-115.03
-115.05
F
O
OH
N
Boc
27b
19F NMR (376 MHz, CDCl3)
20 10 0 -10 -20 -30 -40 -50 -60 -70 -80 -90 -100 -110 -120 -130 -140 -150 -160 -170
ppm

trans-(±)-1-(_tert_-Butoxycarbonyl)-4-(4-methoxyphenyl)pyrrolidine-3-carboxylic acid (24a)

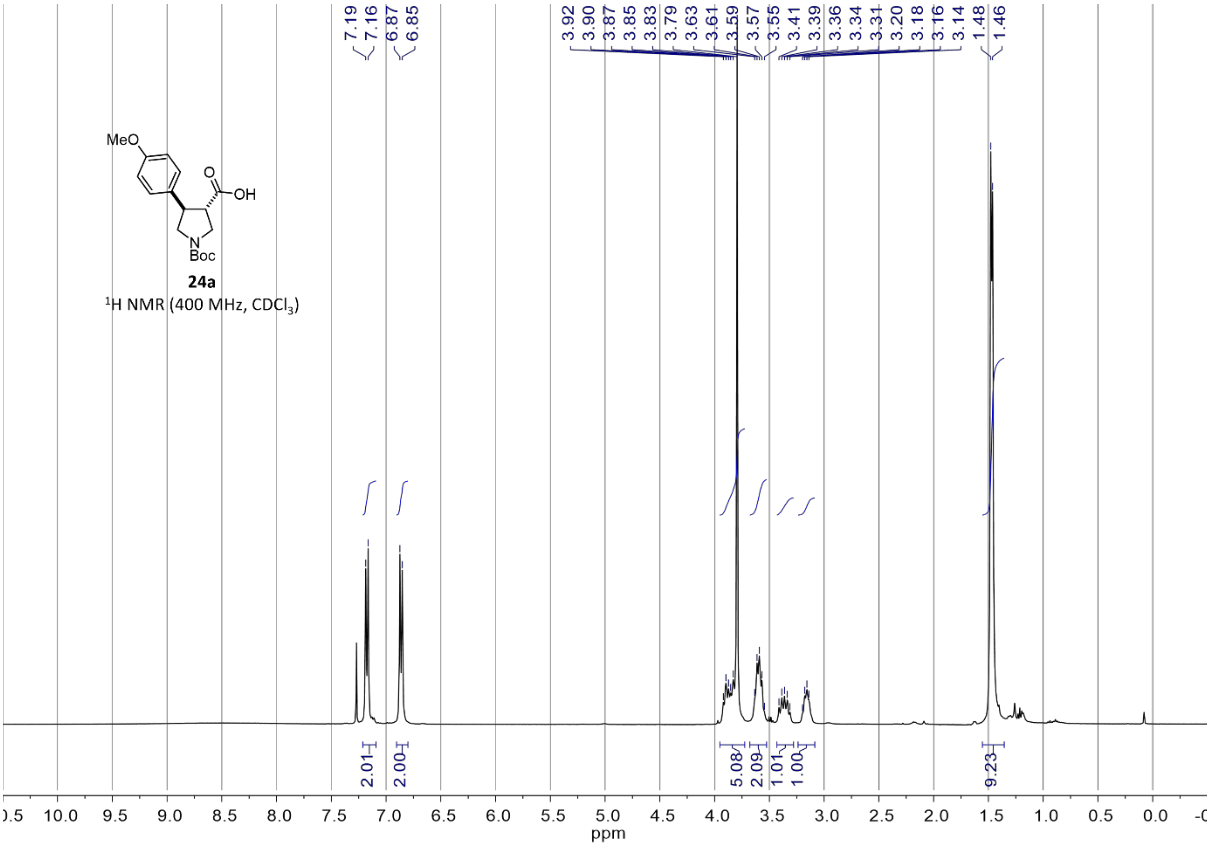

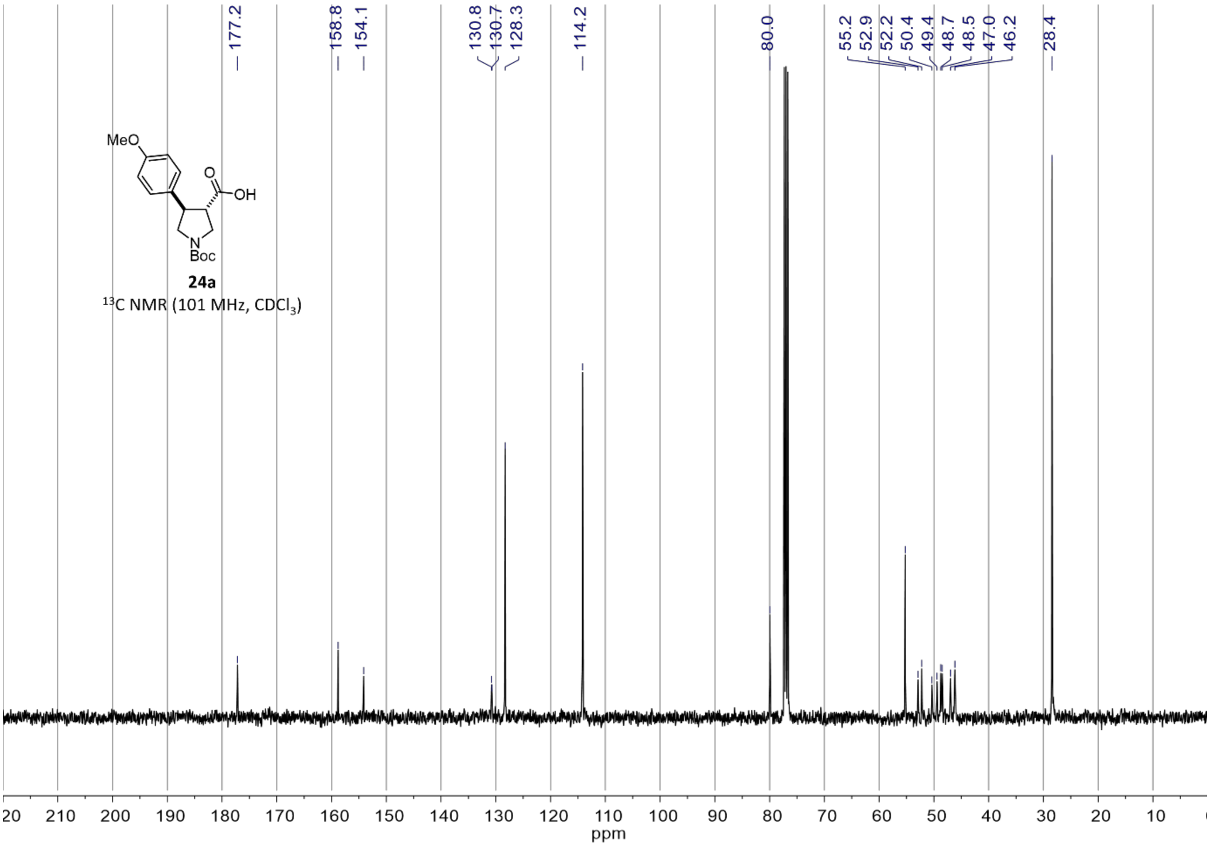

***trans*-(±)-1-(*tert*-Butoxycarbonyl)-4-(4-fluorophenyl)pyrrolidine-3-carboxylic acid (24b)**

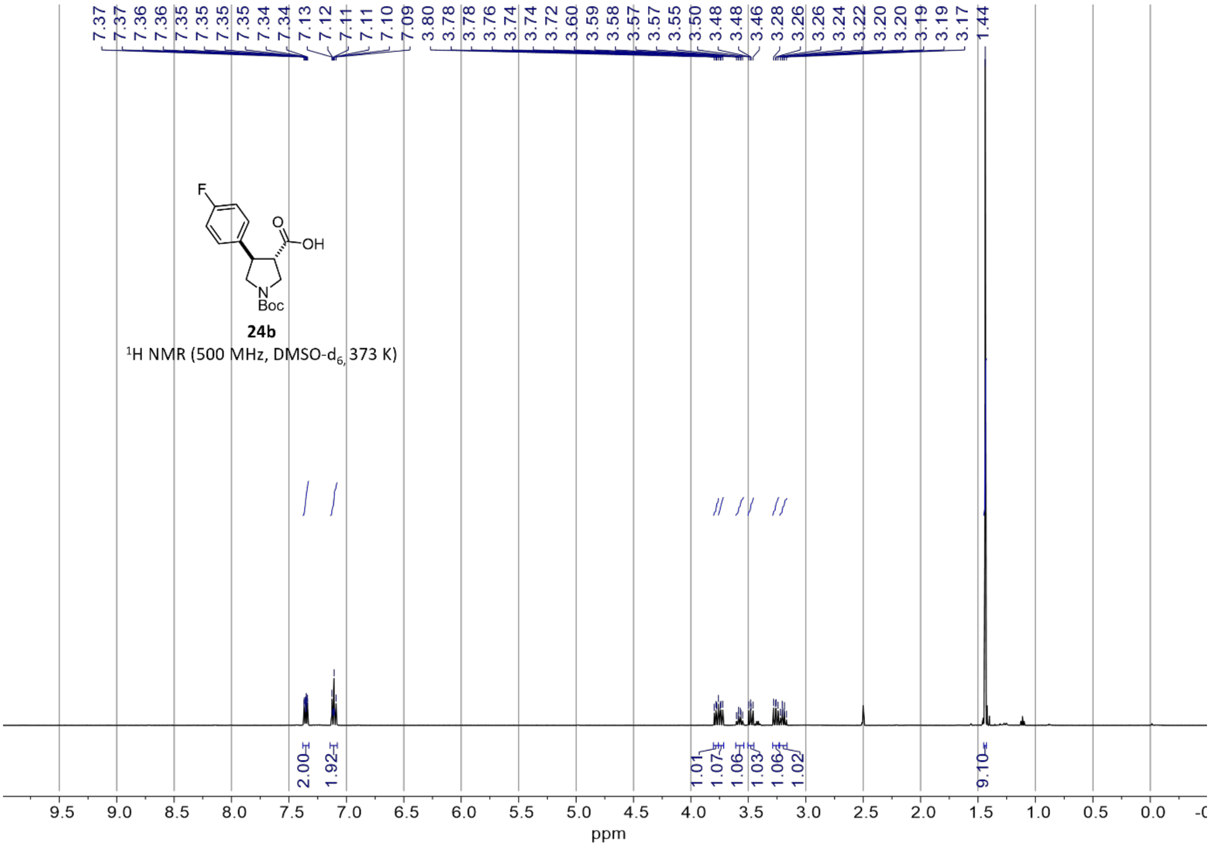

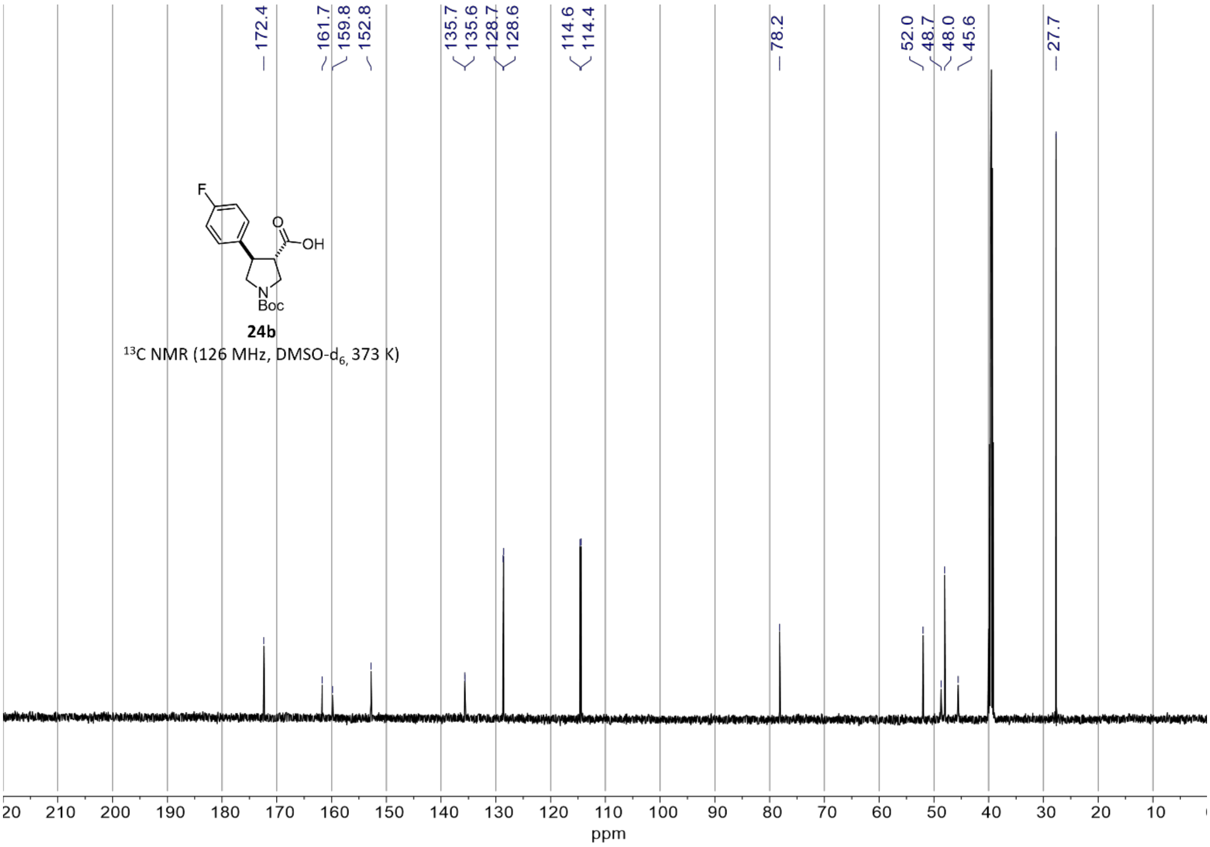

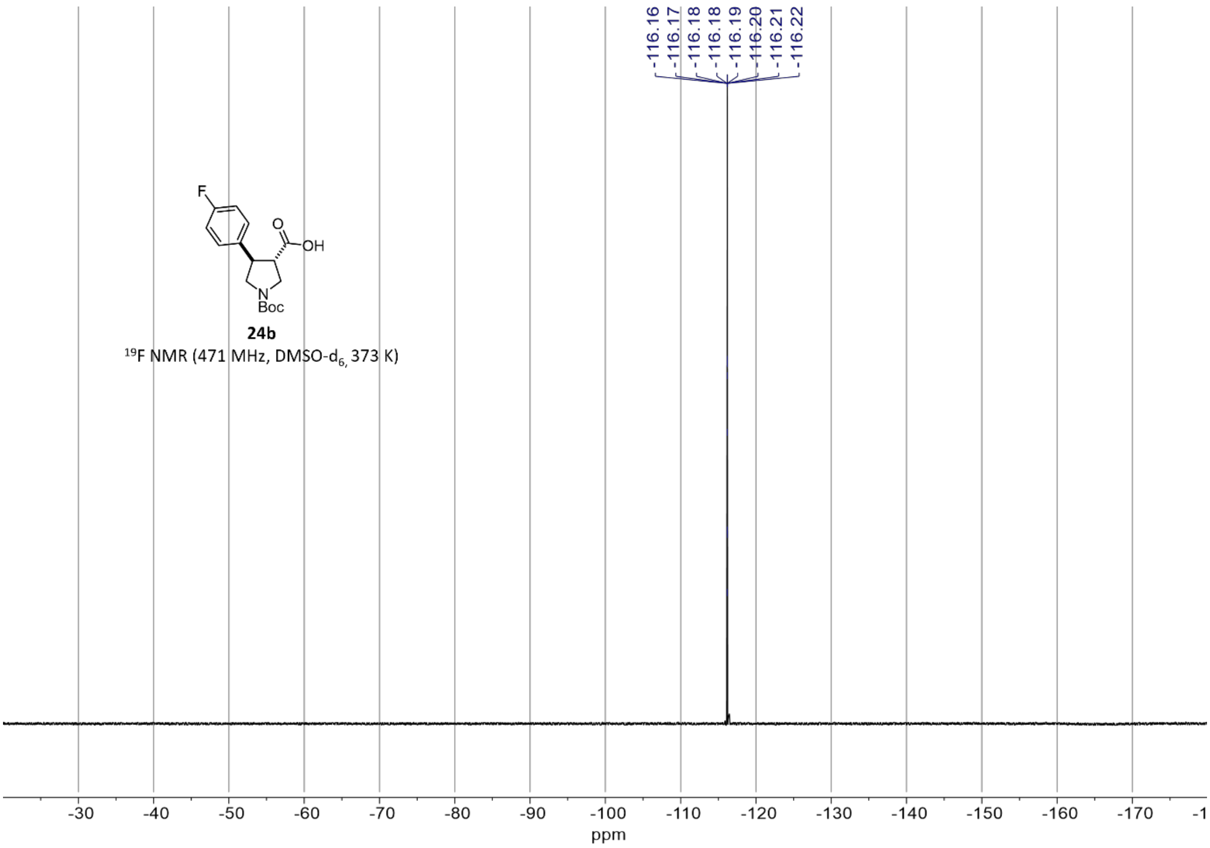
-116.16
-116.17
-116.18
-116.18
-116.19
-116.20
-116.21
-116.22
F
O
OH
N
Boc
24b
^{19}F NMR (471 MHz, DMSO-d_6, 373 K)
-30 -40 -50 -60 -70 -80 -90 -100 -110 -120 -130 -140 -150 -160 -170 -1
ppm

cis-(±)-tert-Butyl 3-carbamoyl-4-(4-fluorophenyl)pyrrolidine-1-carboxylate (30b)

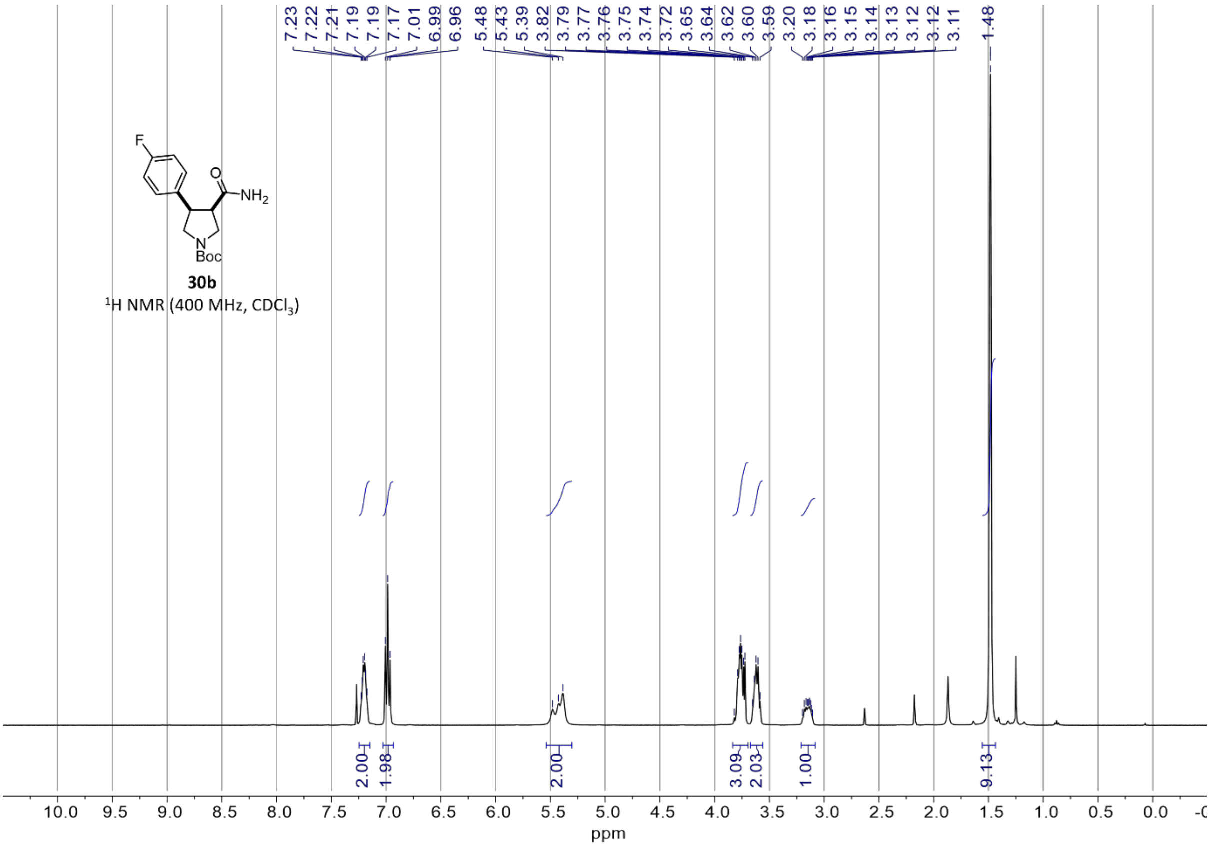

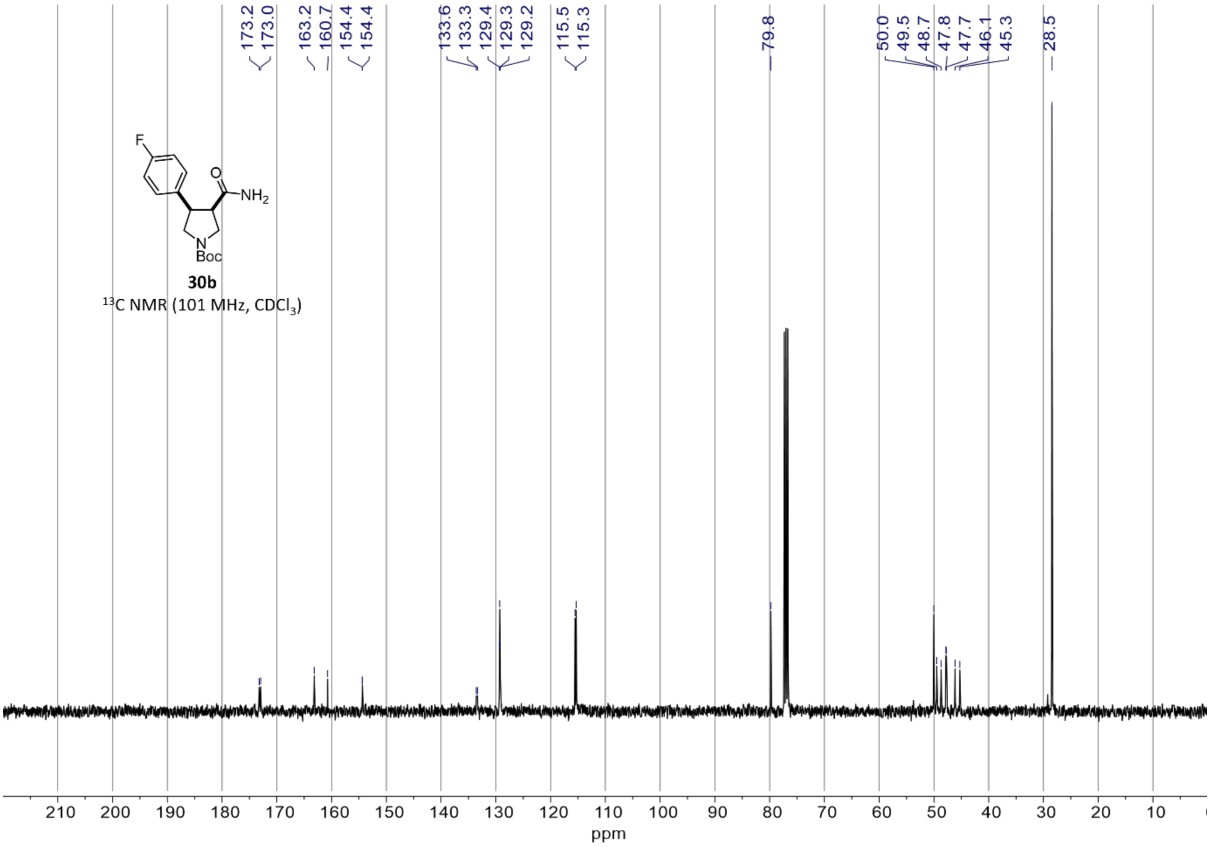

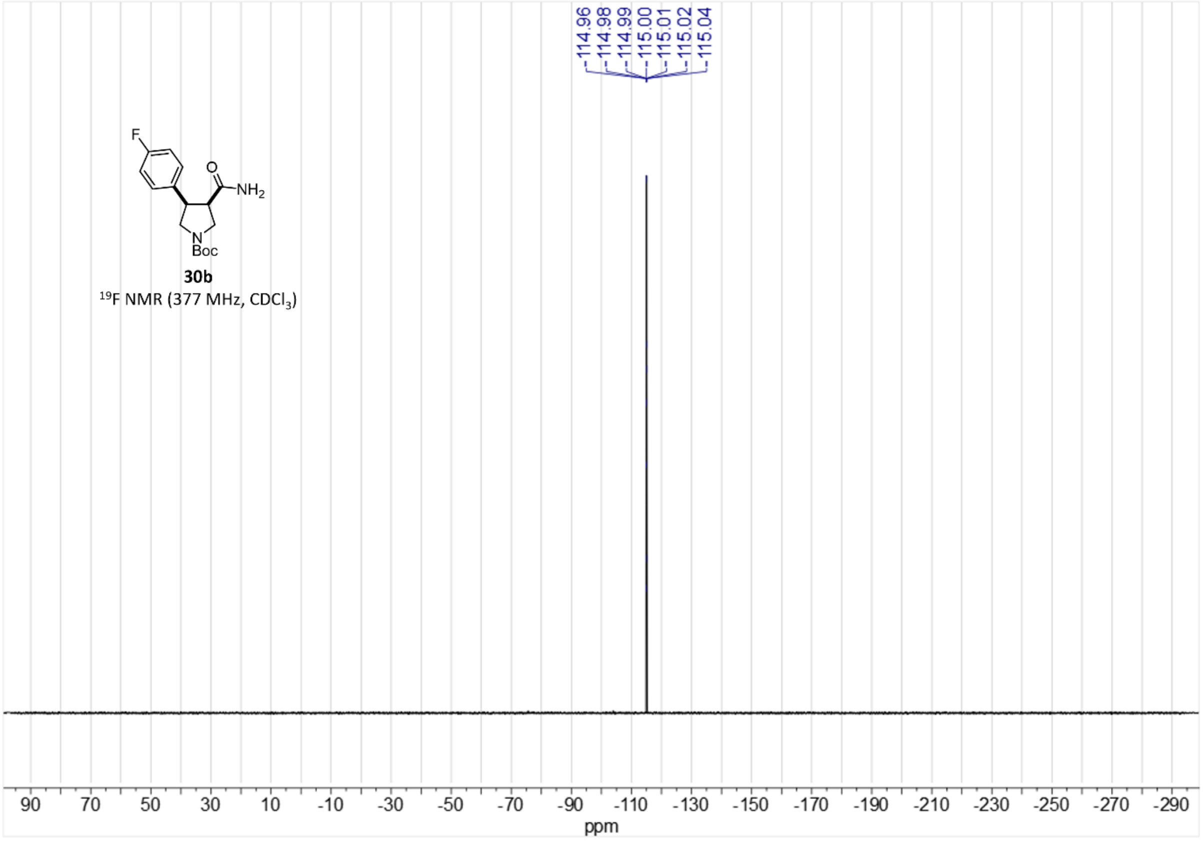
-114.96
-114.98
-114.99
-115.00
-115.01
-115.02
-115.04
F
O
NH2
N
Boc
30b
19F NMR (377 MHz, CDCl3)
90
70
50
30
10
-10
-30
-50
-70
-90
-110
-130
-150
-170
-190
-210
-230
-250
-270
-290
ppm

trans-(±)-_tert_-Butyl 3-carbamoyl-4-(4-methoxyphenyl)pyrrolidine-1-carboxylate (32a)

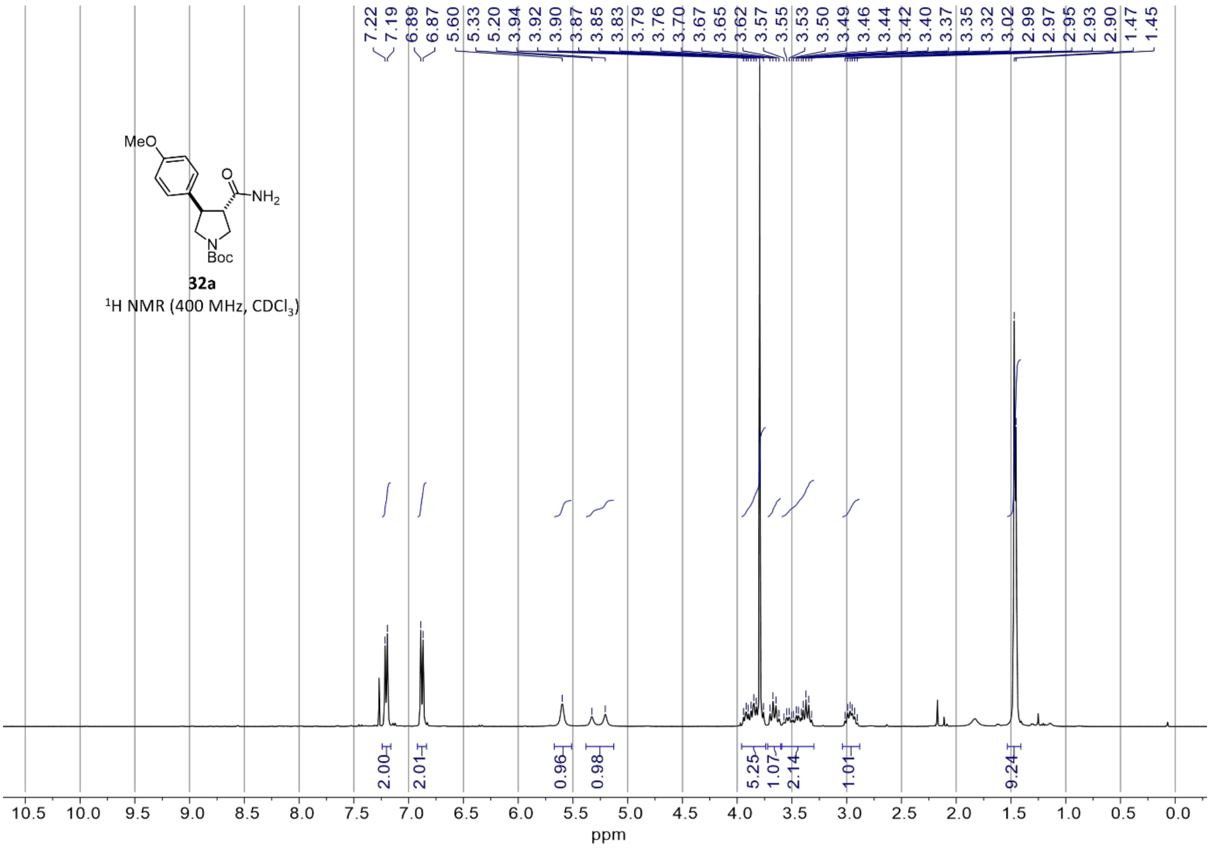

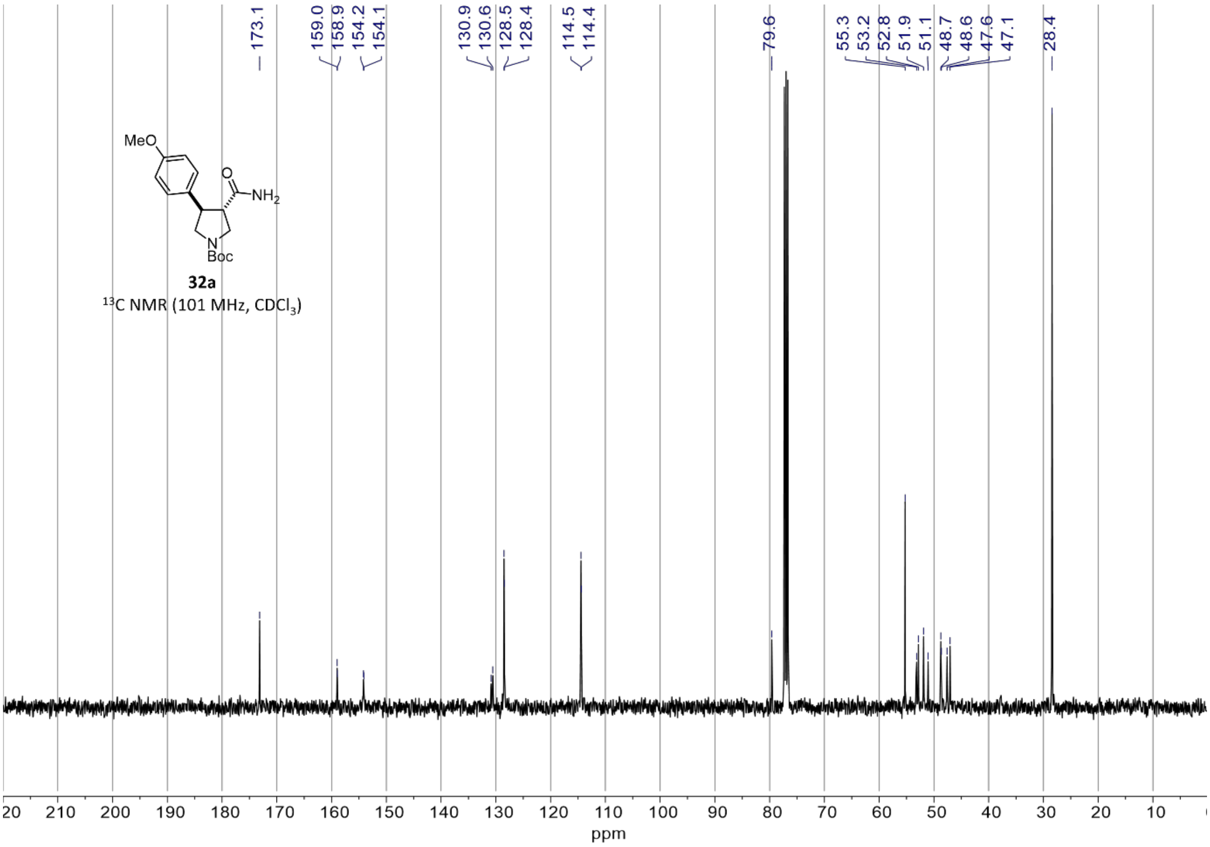

trans-(±)-_tert_-Butyl 3-carbamoyl-4-(4-fluorophenyl)pyrrolidine-1-carboxylate (32b)

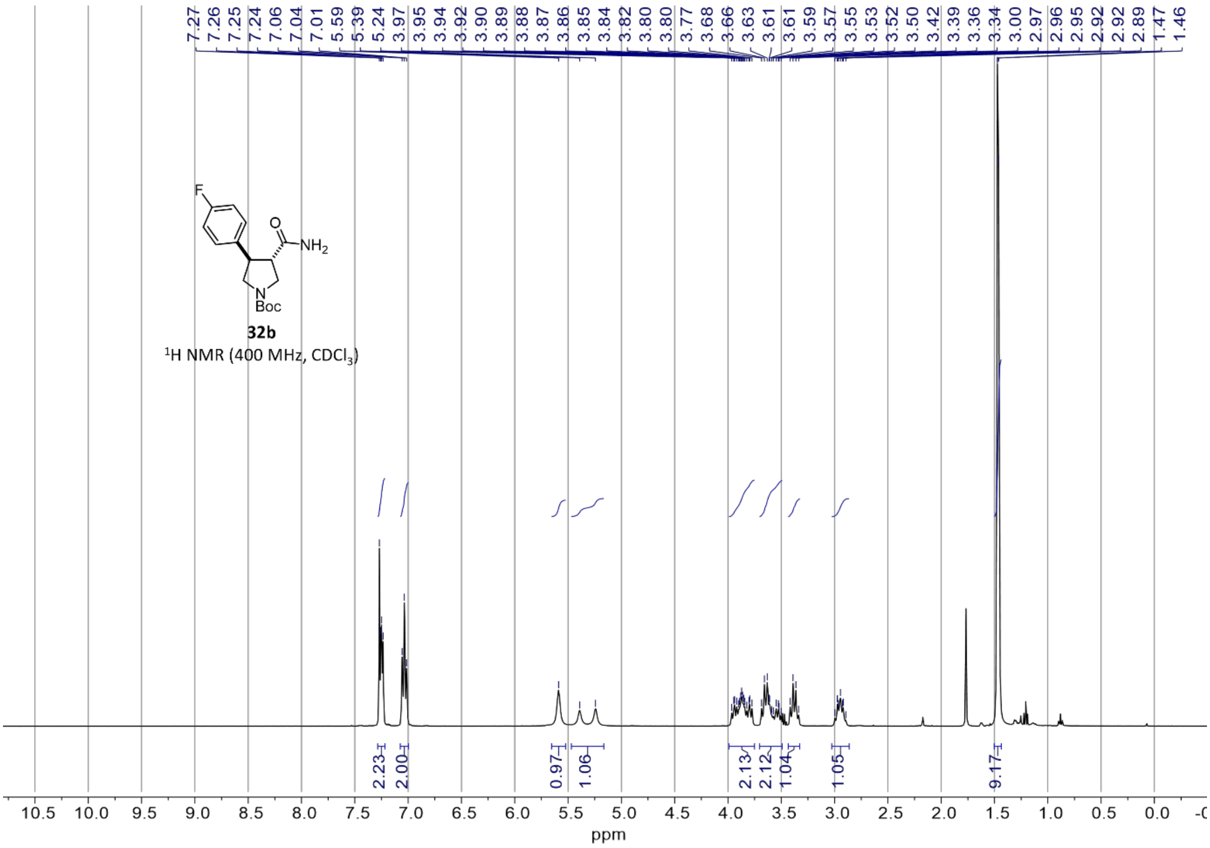

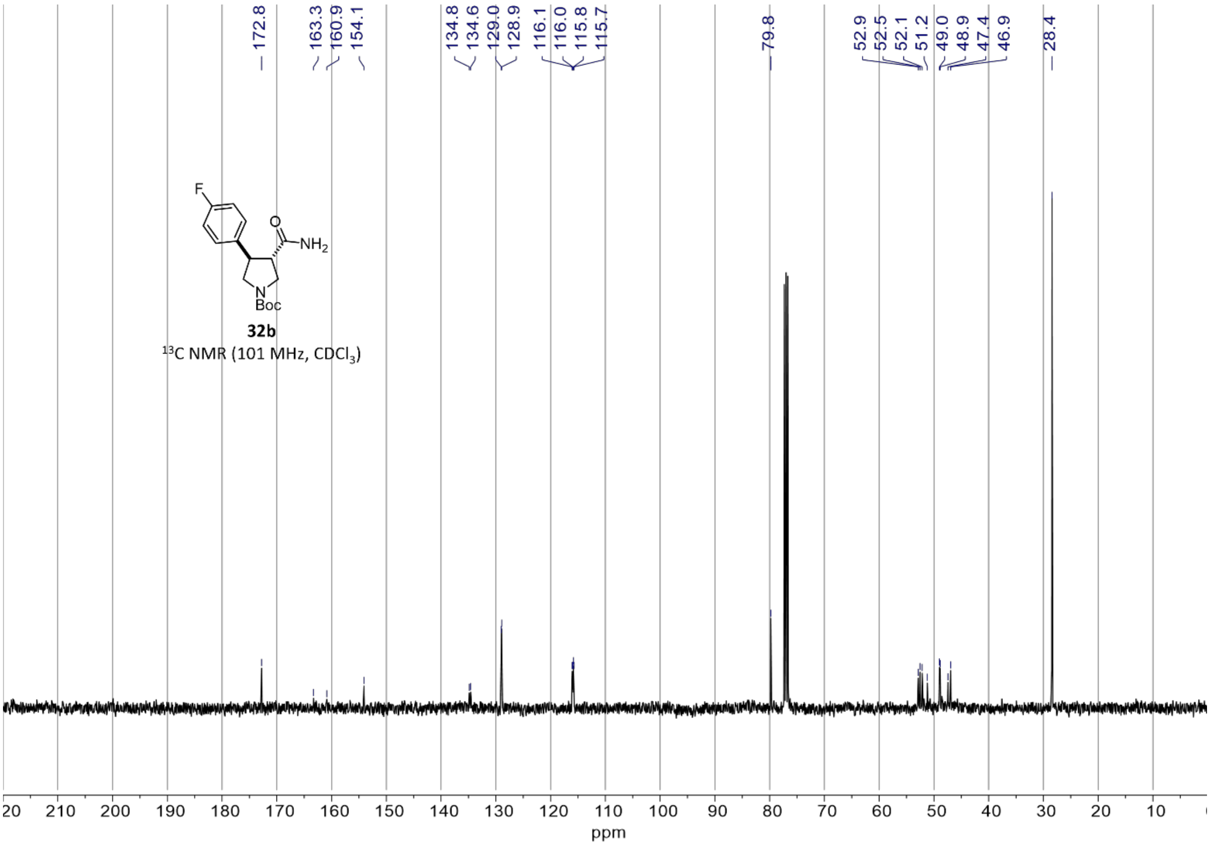

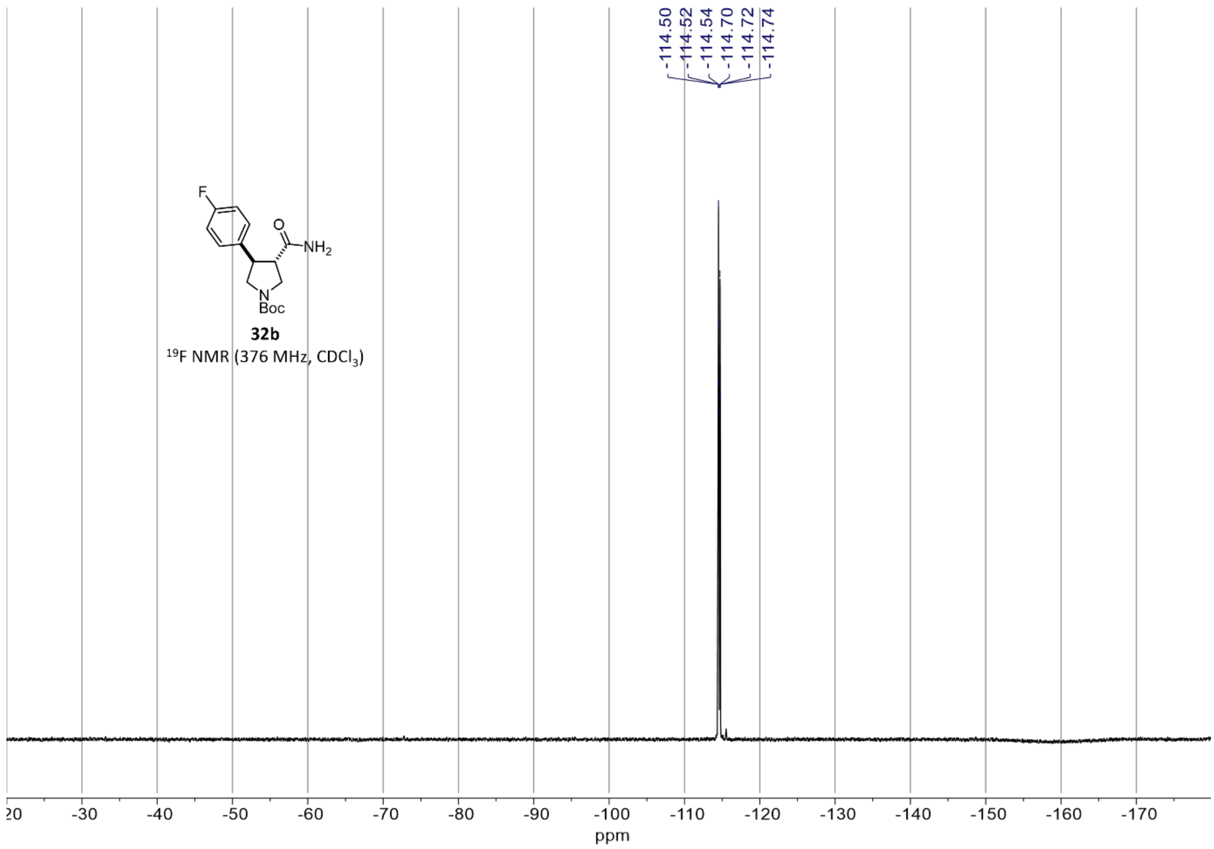

32b

^{19}F NMR (376 MHz, $CDCl_3$)

cis-(±)-*tert*-Butyl 3-(hydroxymethyl)-4-(4-methoxyphenyl)pyrrolidine-1-carboxylate (28a)

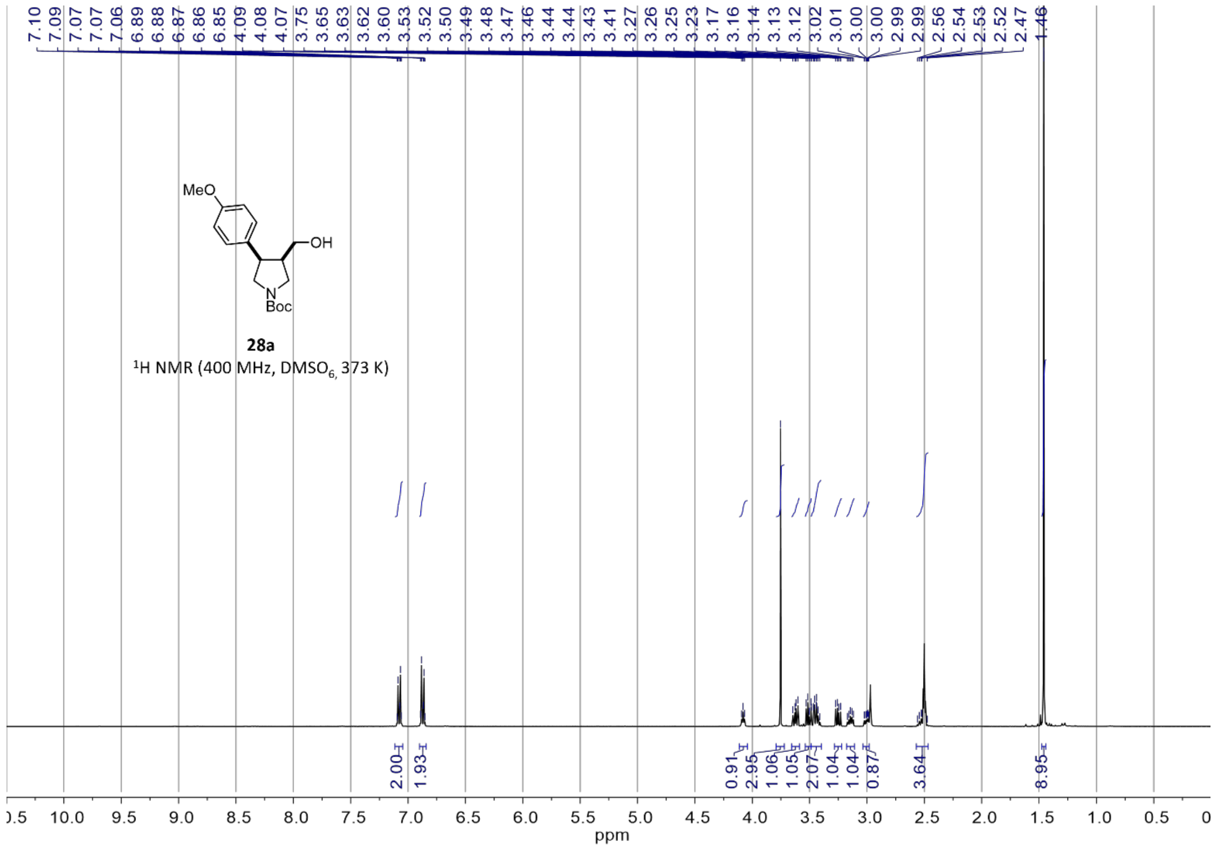

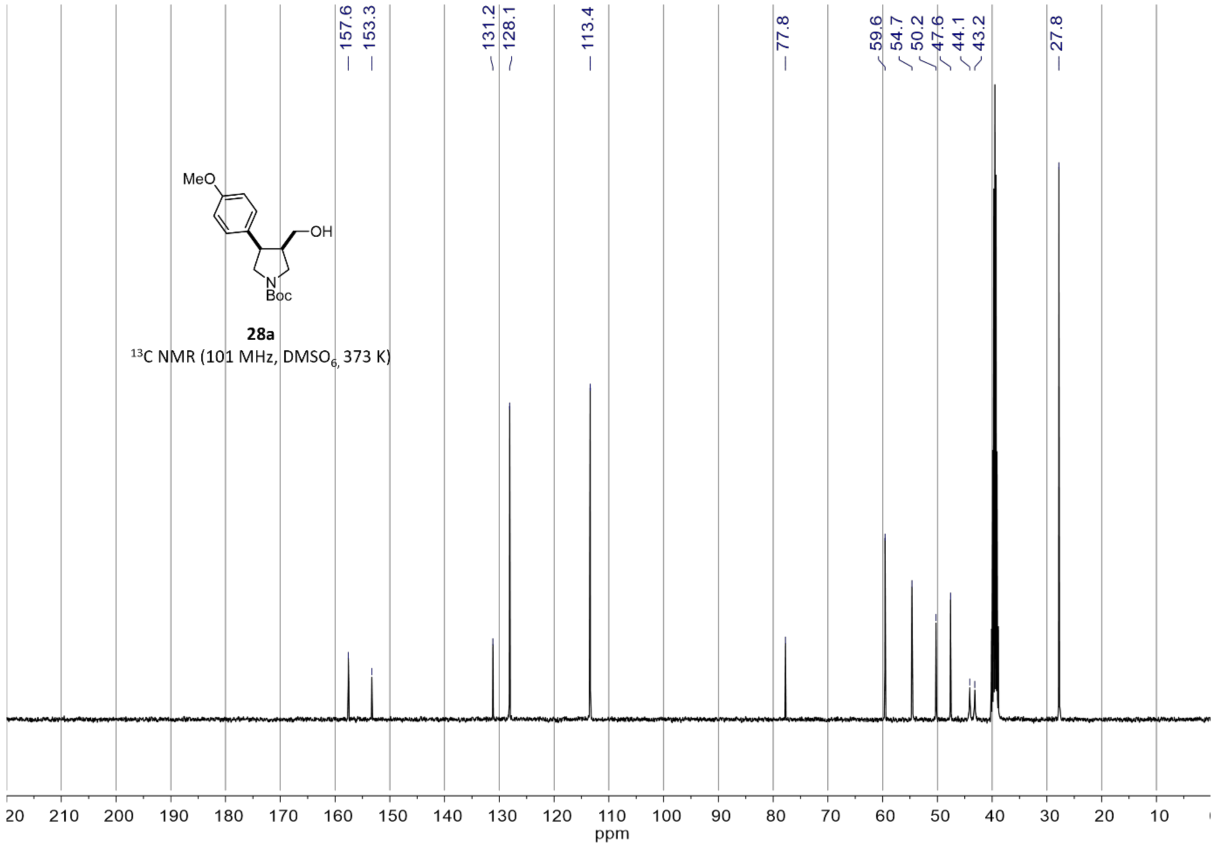

cis-(±)-tert-Butyl 3-(hydroxymethyl)-4-(4-fluorophenyl)pyrrolidine-1-carboxylate (28b)

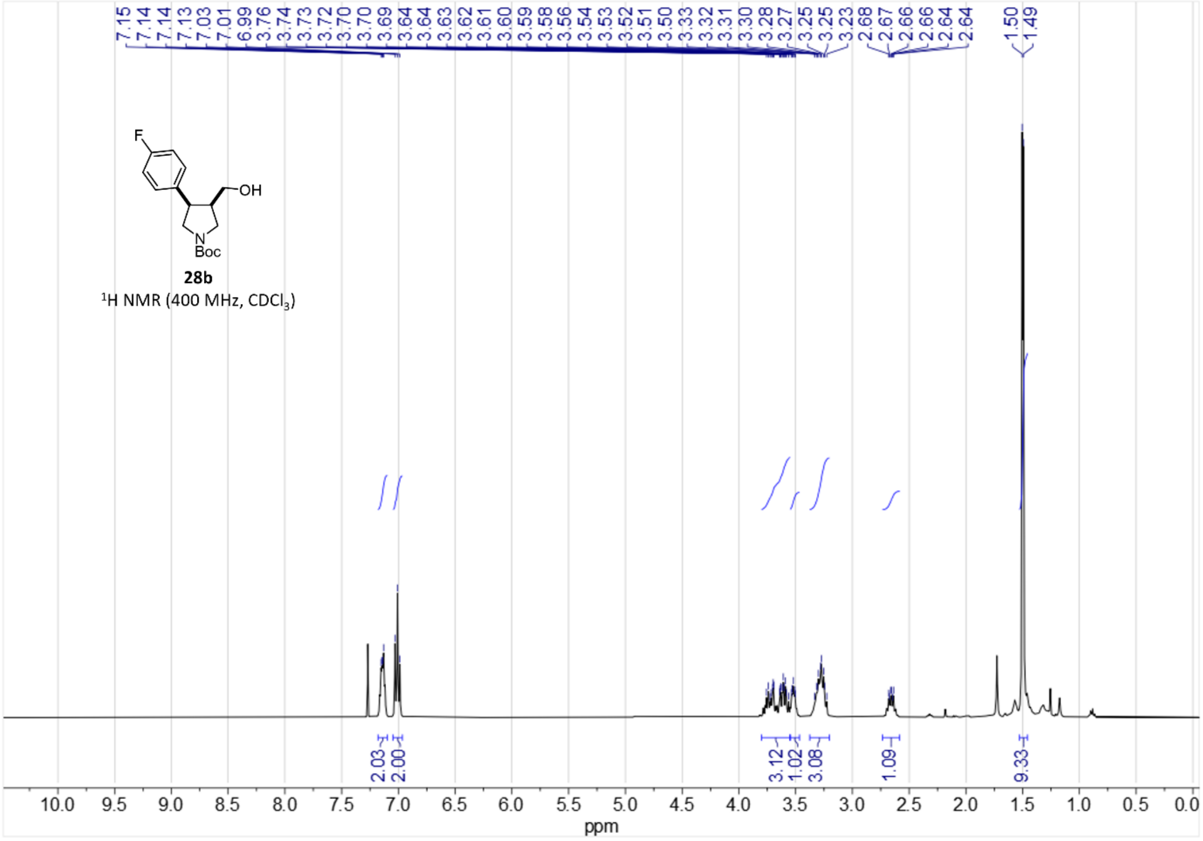

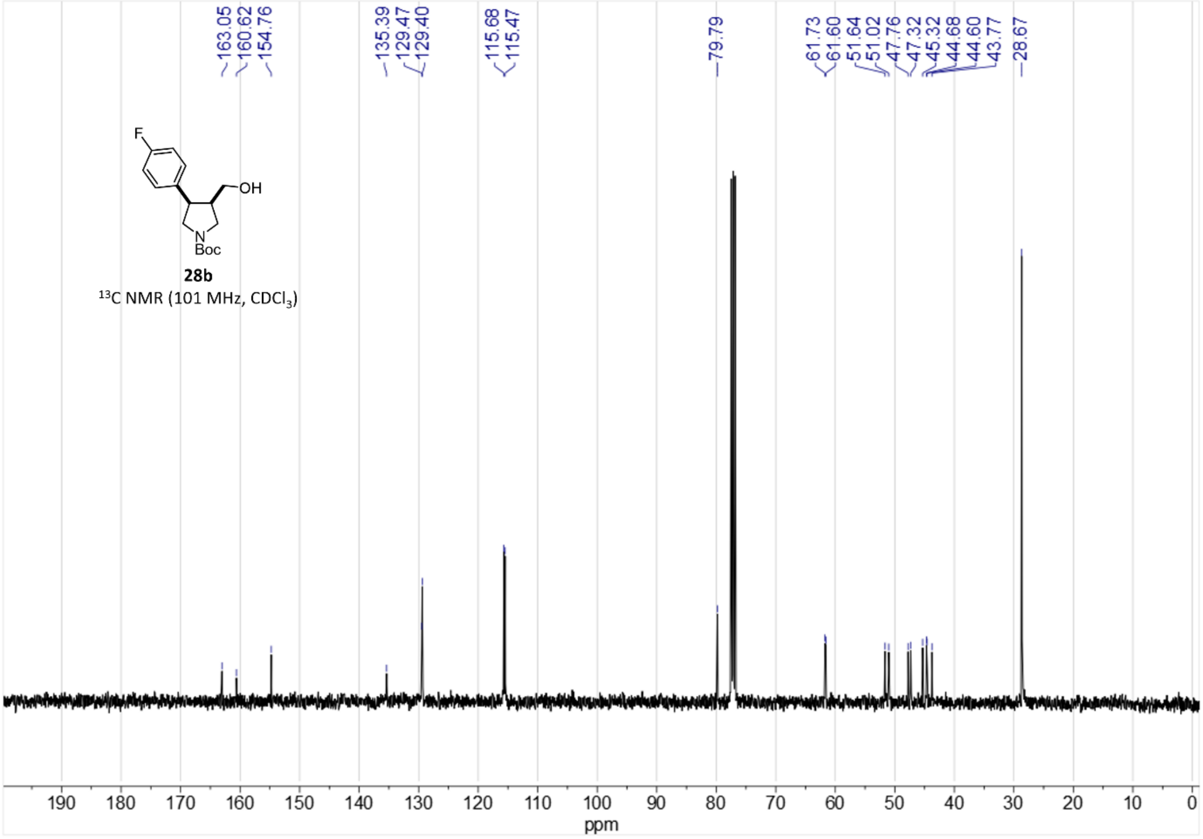

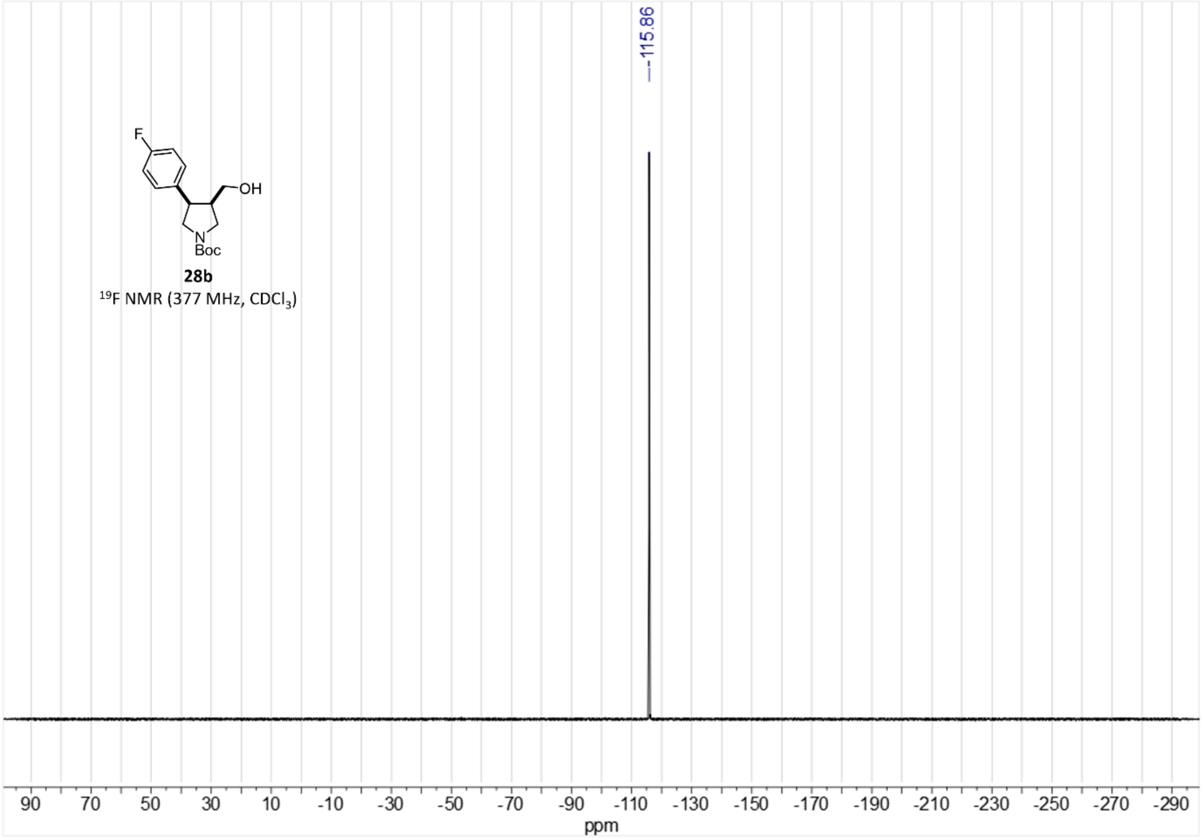

F
OH
N
Boc
28b
^{19}F NMR (377 MHz, $CDCl_3$)
-115.86
90
70
50
30
10
-10
-30
-50
-70
-90
-110
-130
-150
-170
-190
-210
-230
-250
-270
-290
ppm

trans-(±)-_tert_-Butyl 3-(hydroxymethyl)-4-(4-methoxyphenyl)pyrrolidine-1-carboxylate (29a)

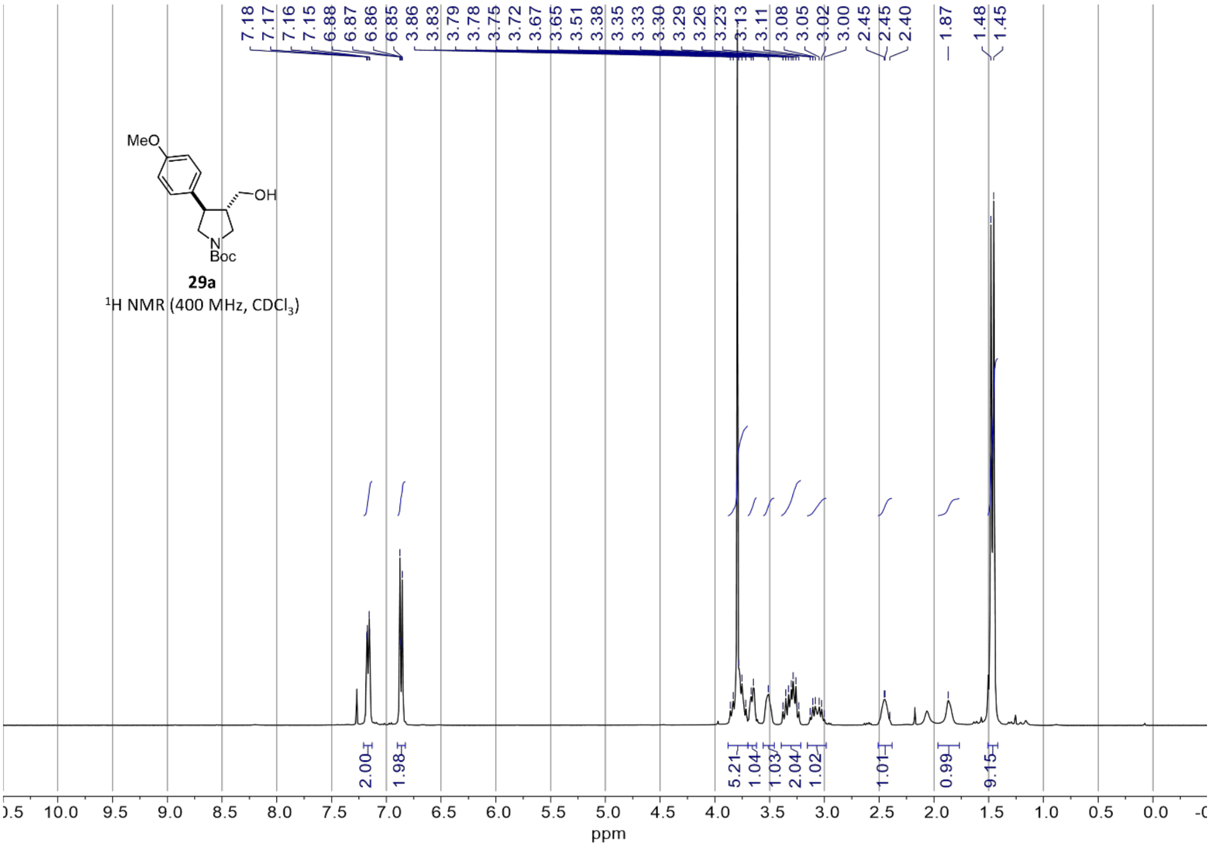

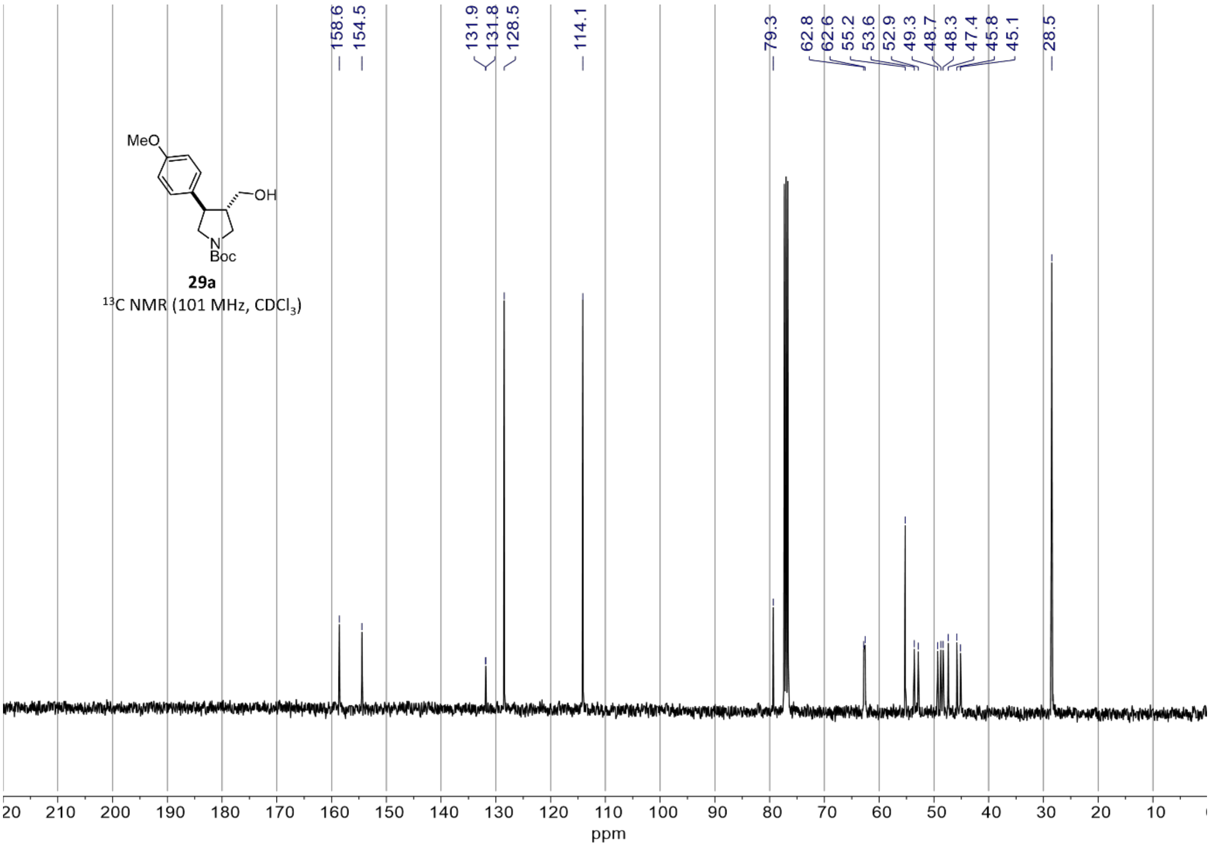

trans-(±)-_tert_-Butyl 3-(hydroxymethyl)-4-(4-fluorophenyl)pyrrolidine-1-carboxylate (29b)

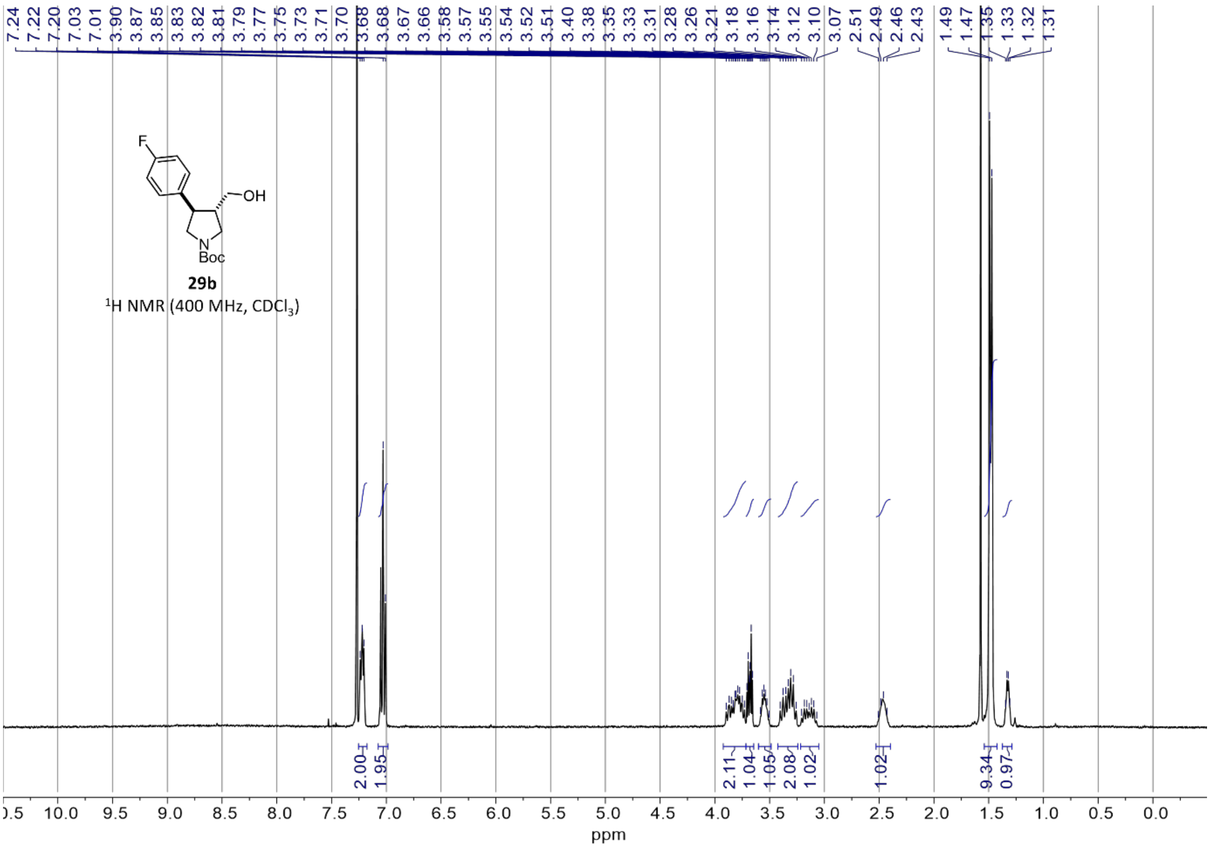

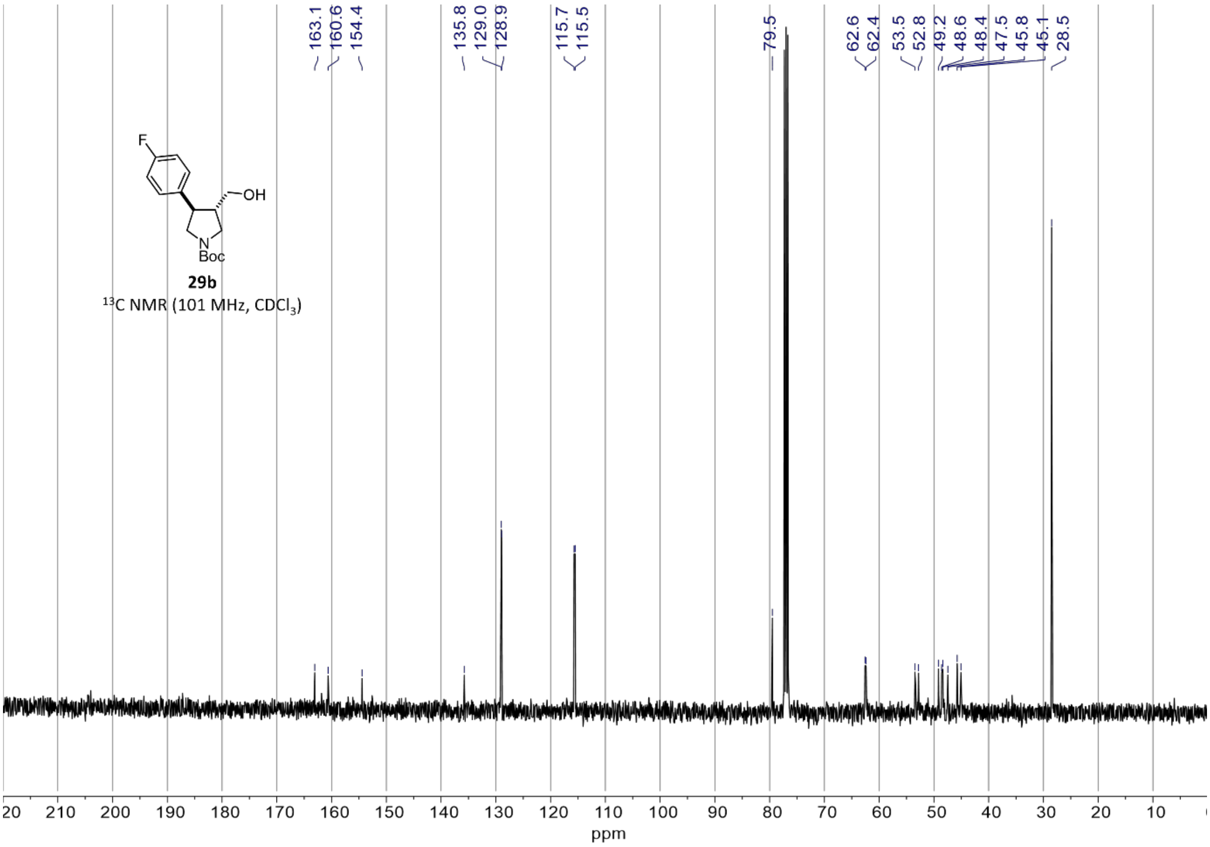

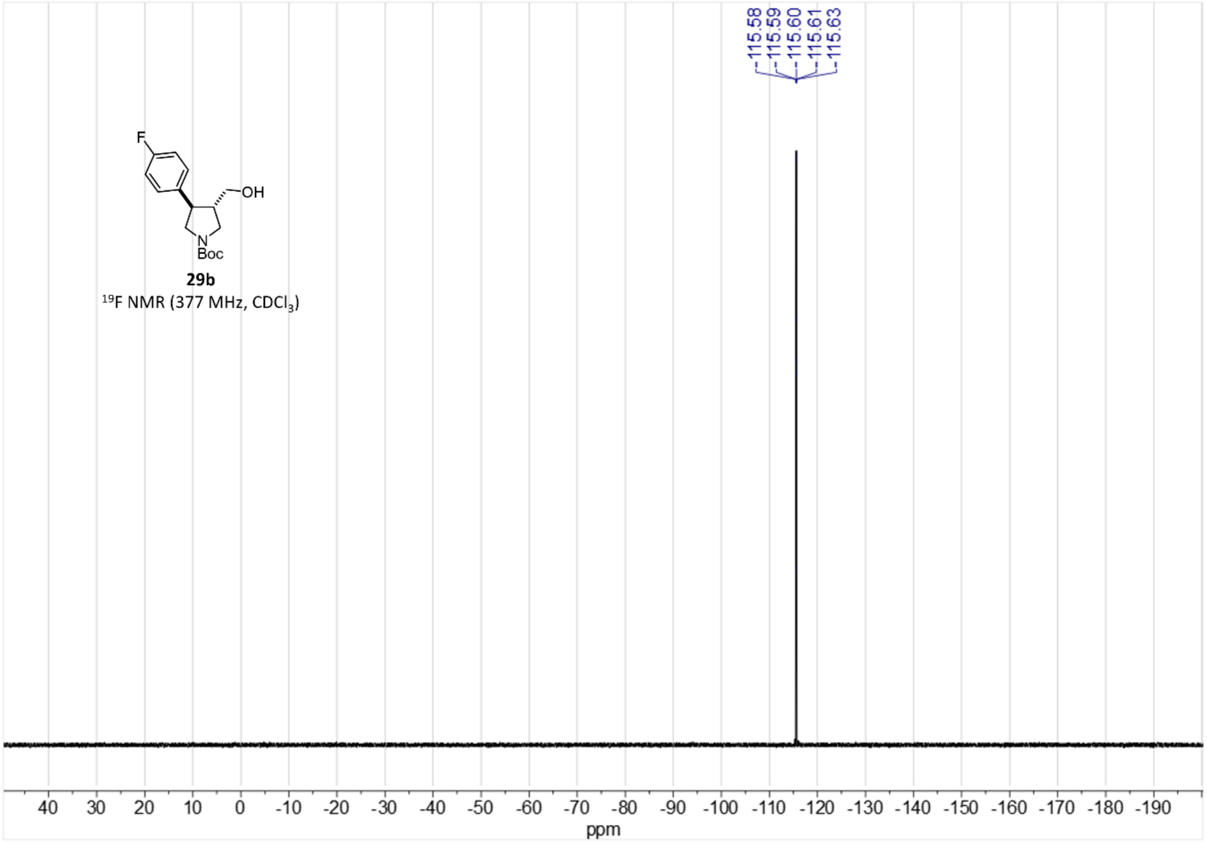

-115.58
-115.59
-115.60
-115.61
-115.63
F
OH
N
Boc
29b
^{19}F NMR (377 MHz, $CDCl_3$)
40 30 20 10 0 -10 -20 -30 -40 -50 -60 -70 -80 -90 -100 -110 -120 -130 -140 -150 -160 -170 -180 -190
ppm

trans-(±)-_tert_-Butyl 3-cyano-4-(4-methoxyphenyl)pyrrolidine-1-carboxylate (S1)

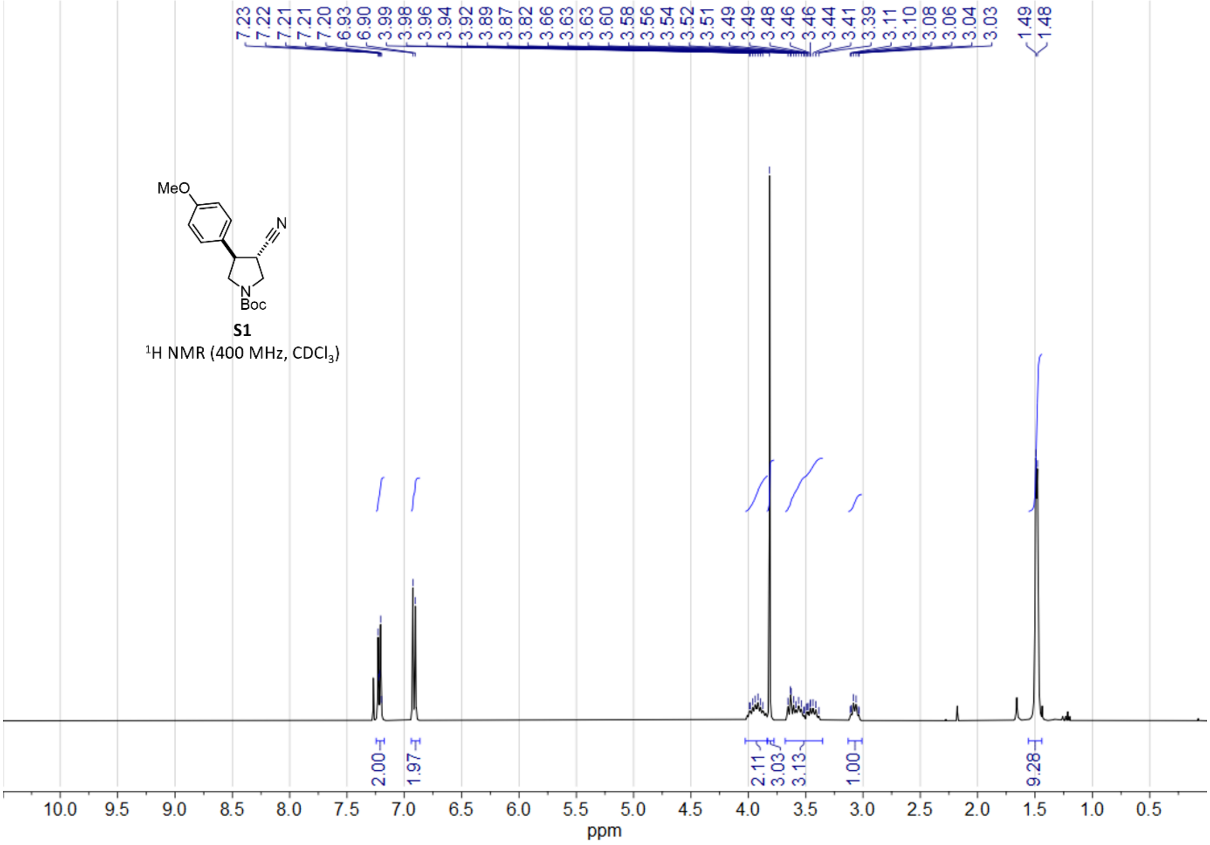

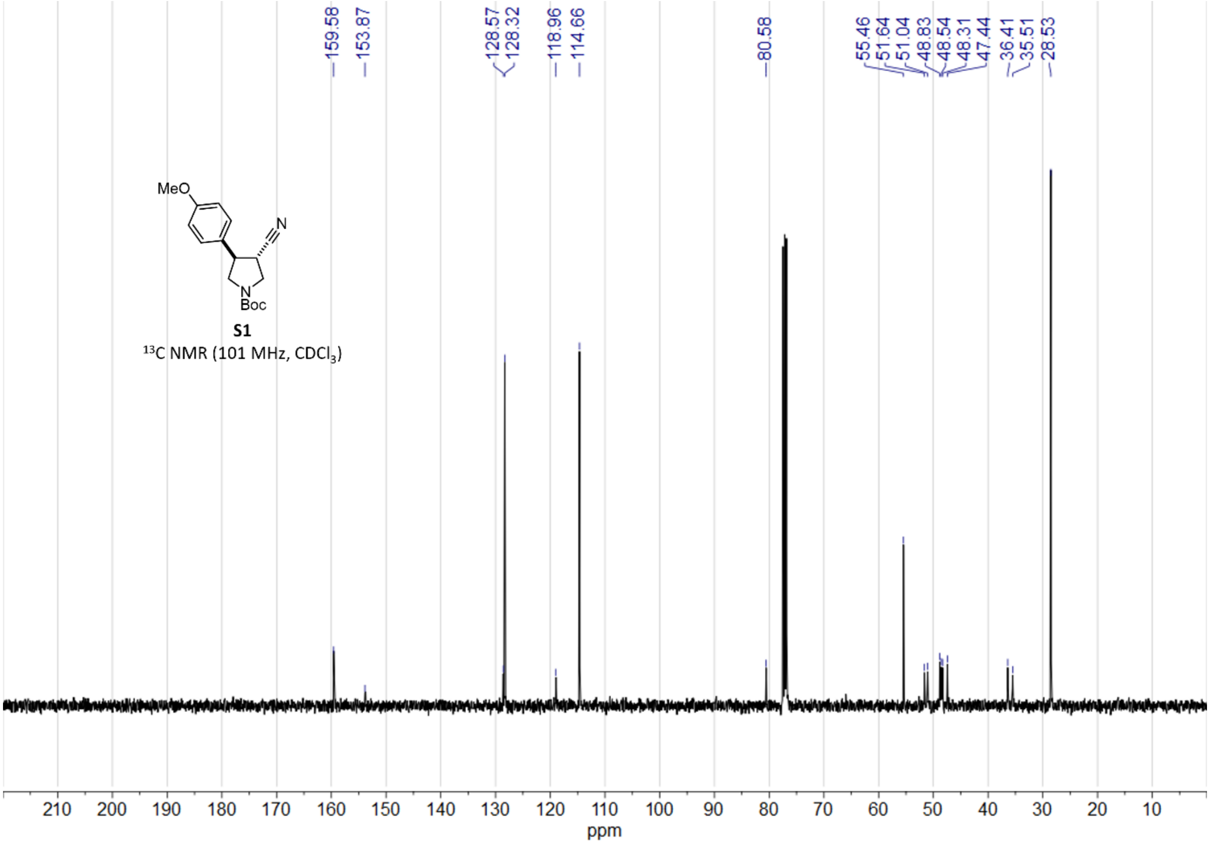

***cis*-(±)-3-Carboxy-4-(4-methoxyphenyl)pyrrolidin-1-ium chloride (FRAG8)**

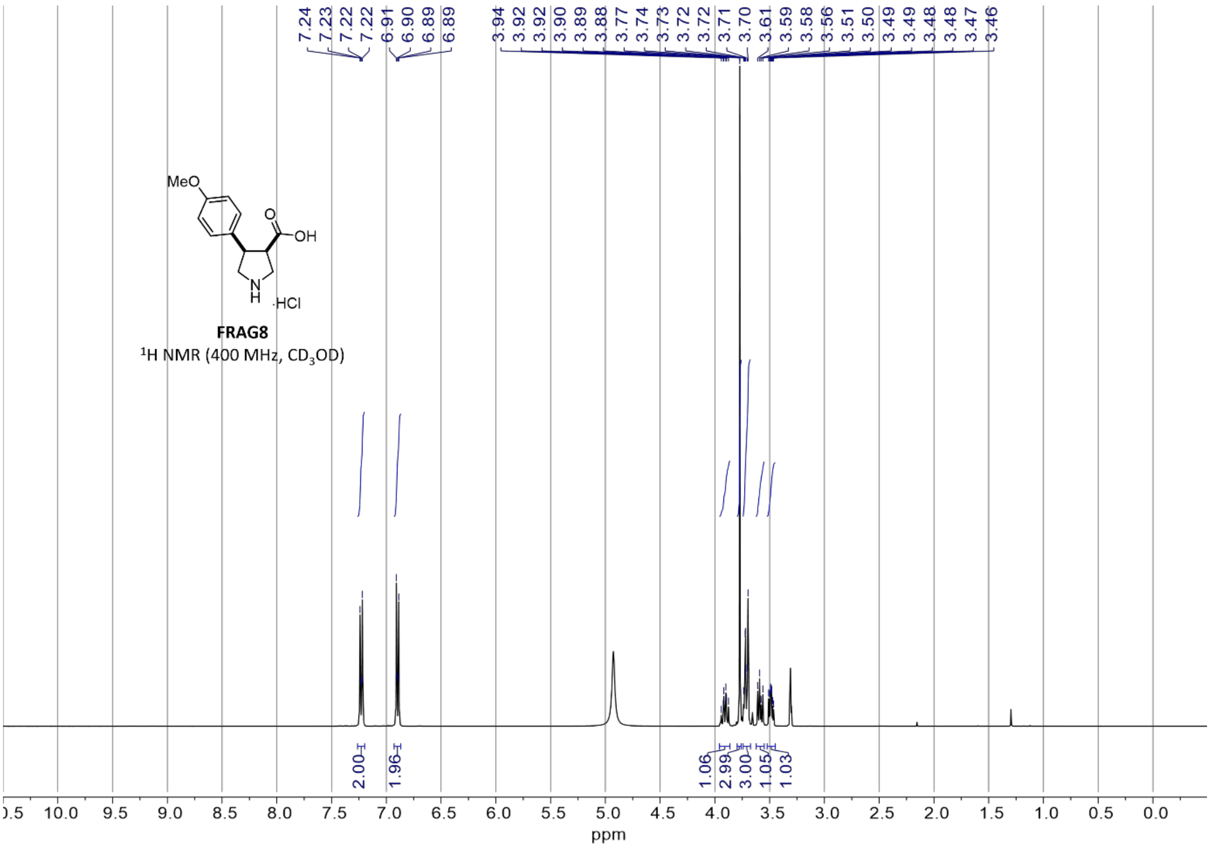

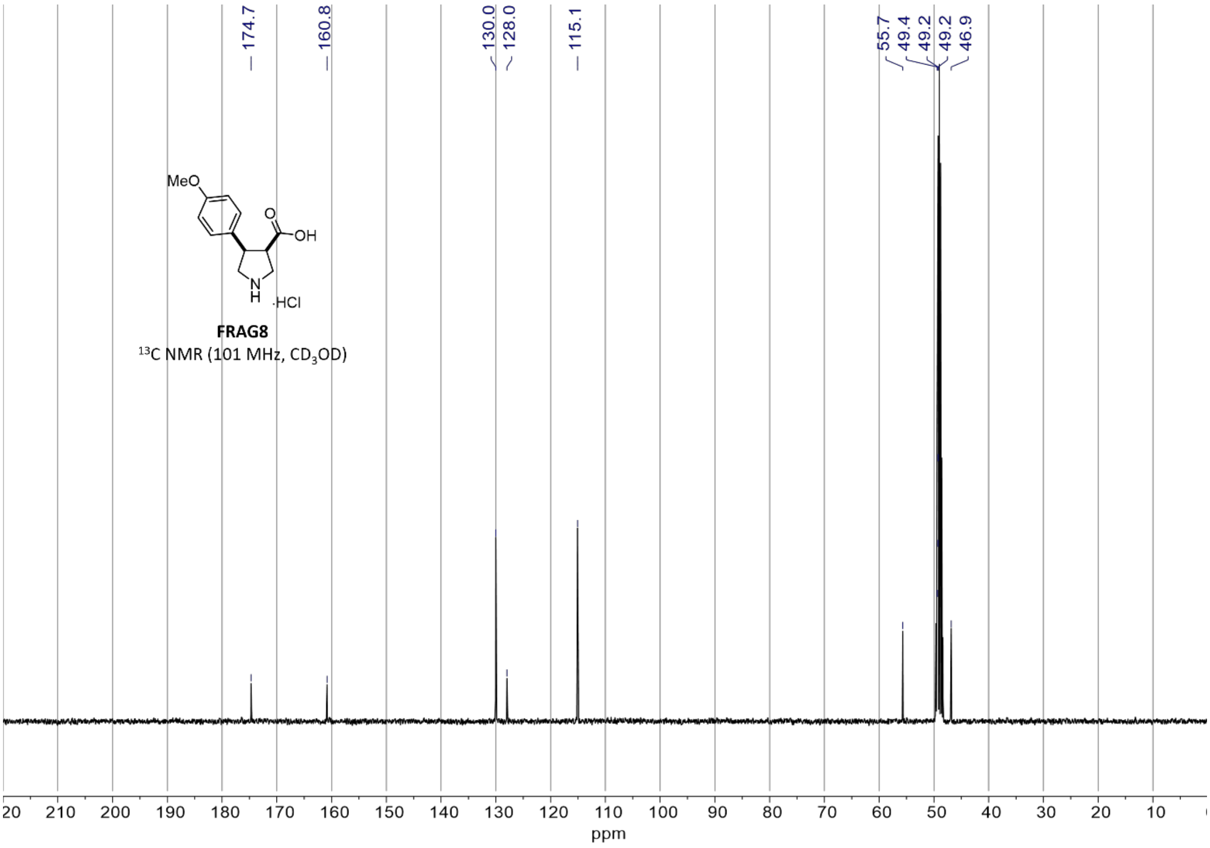

***trans*-(±)-3-Carboxy-4-(4-methoxyphenyl)pyrrolidin-1-ium chloride (FRAG10)**

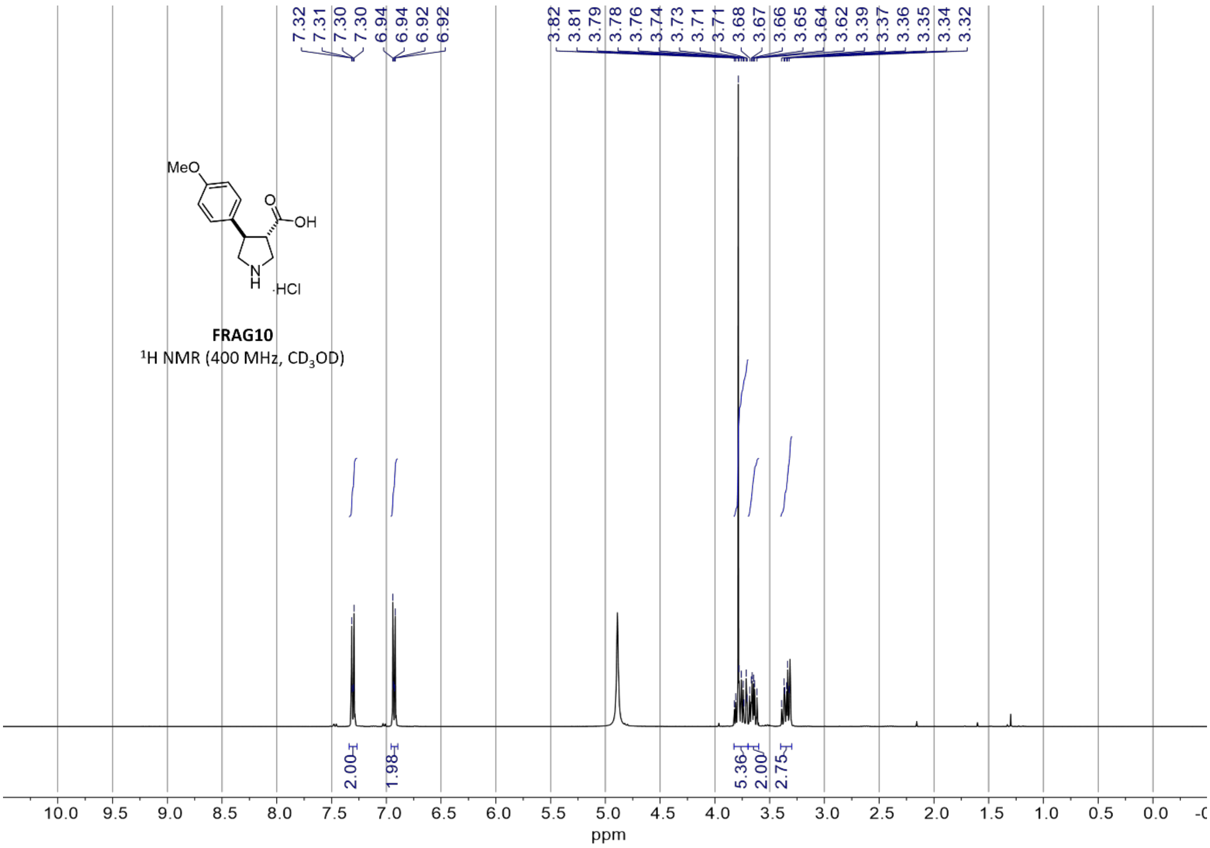

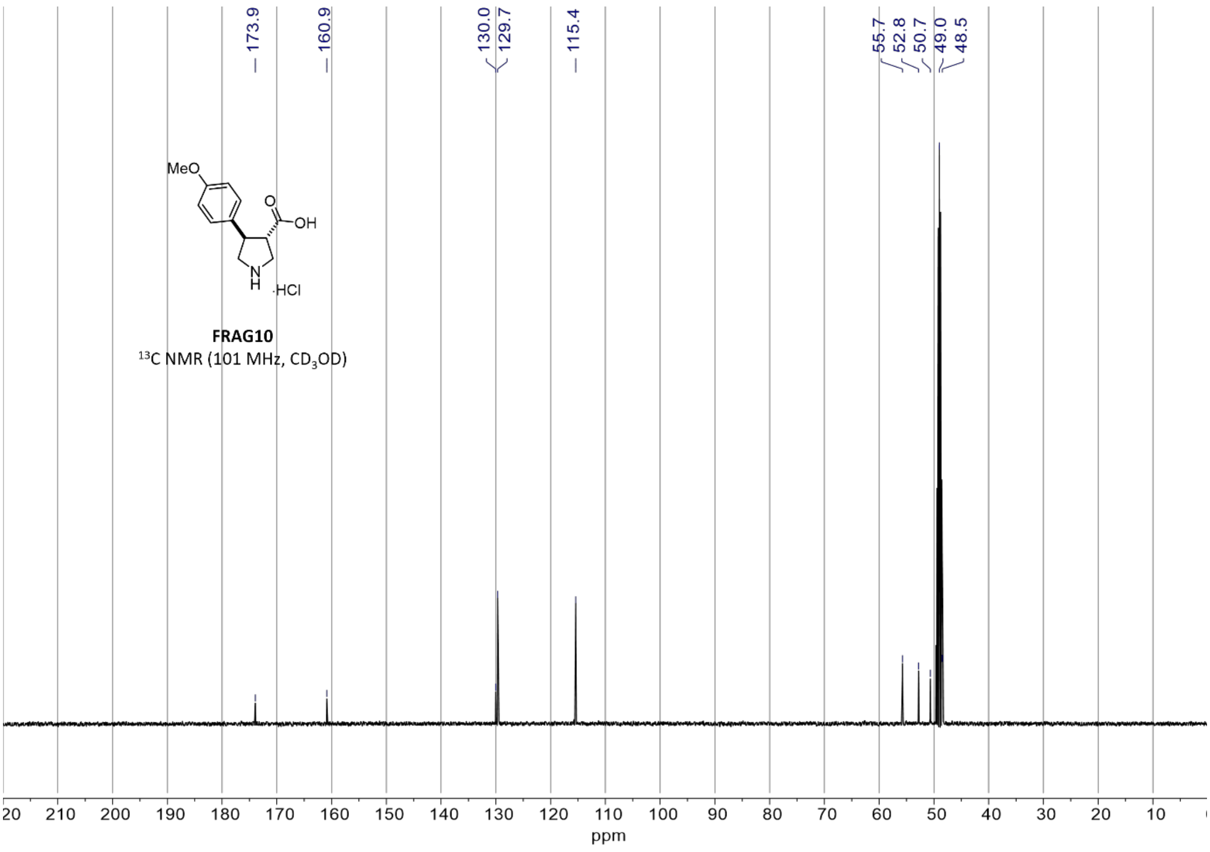

***cis*-(±)-3-Carboxy-4-(4-fluorophenyl)pyrrolidin-1-ium chloride (FRAG9)**

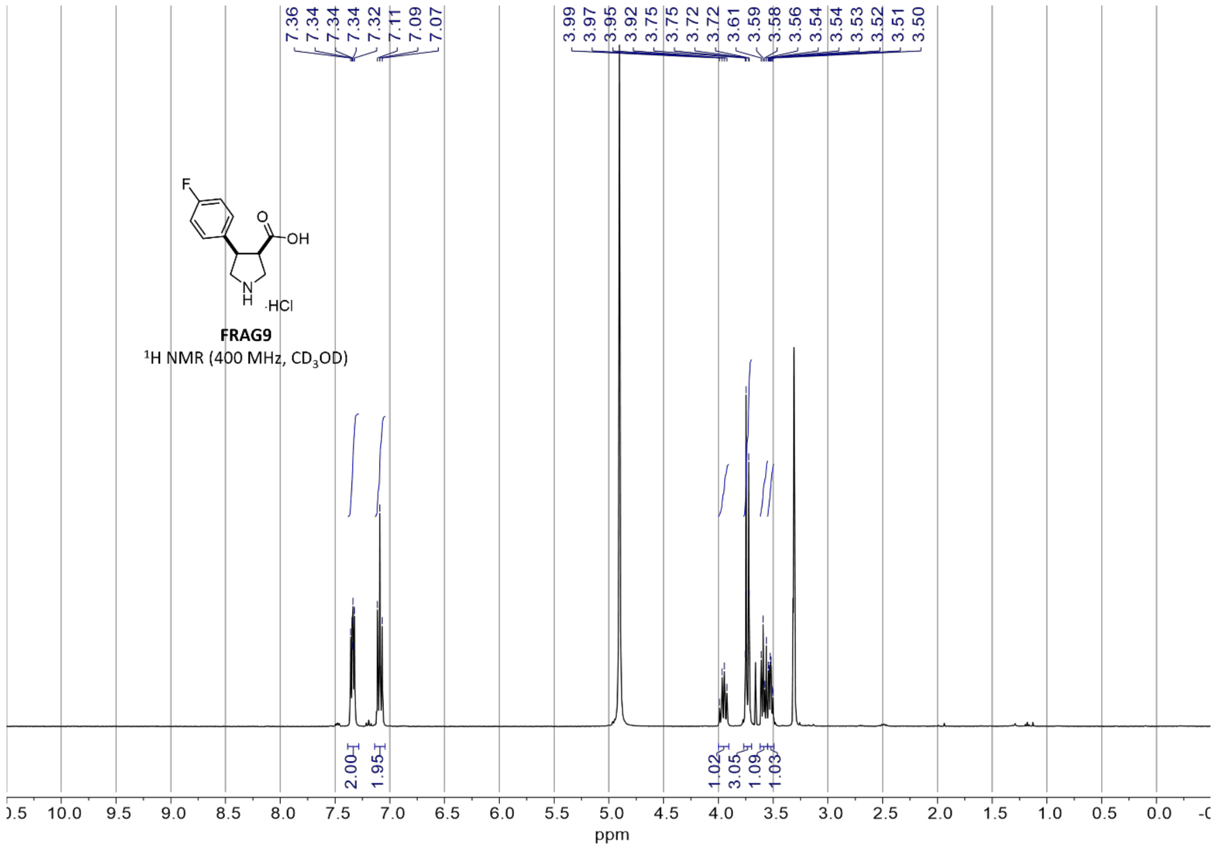

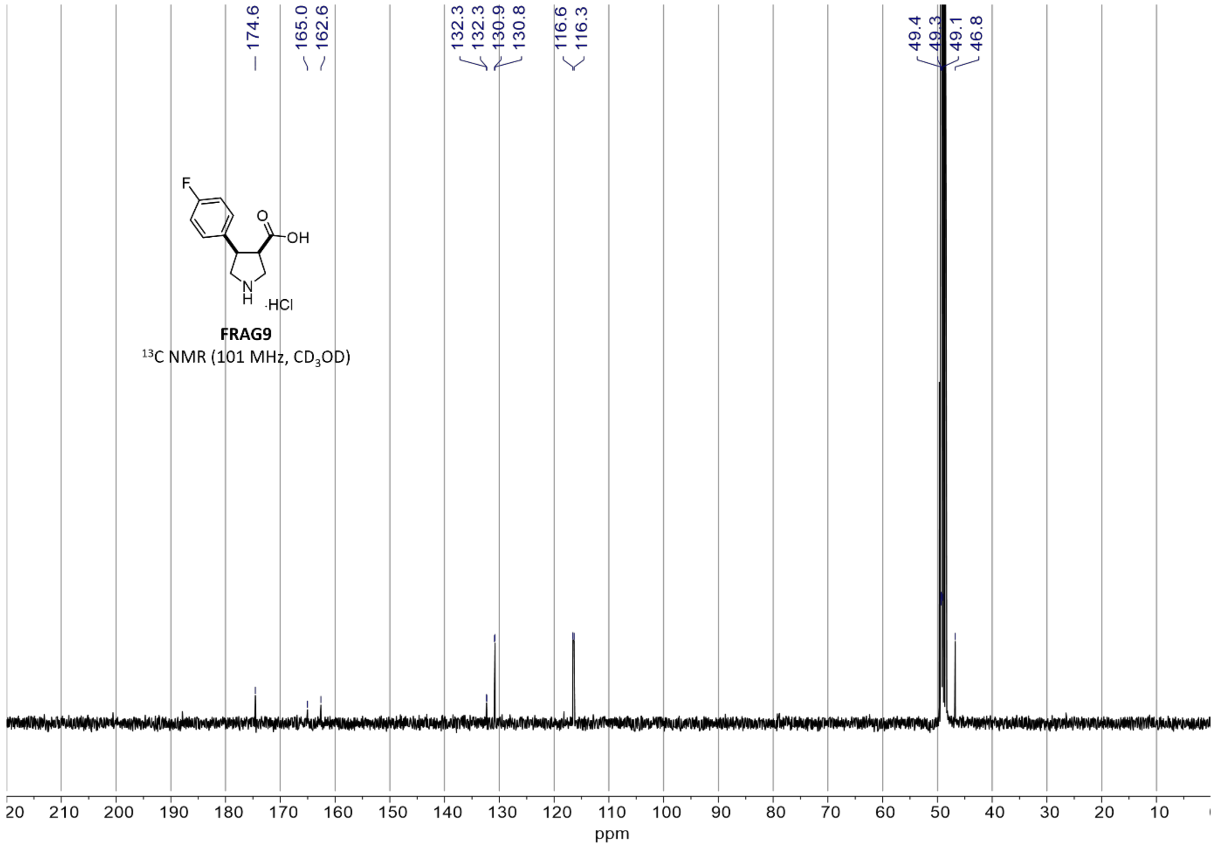

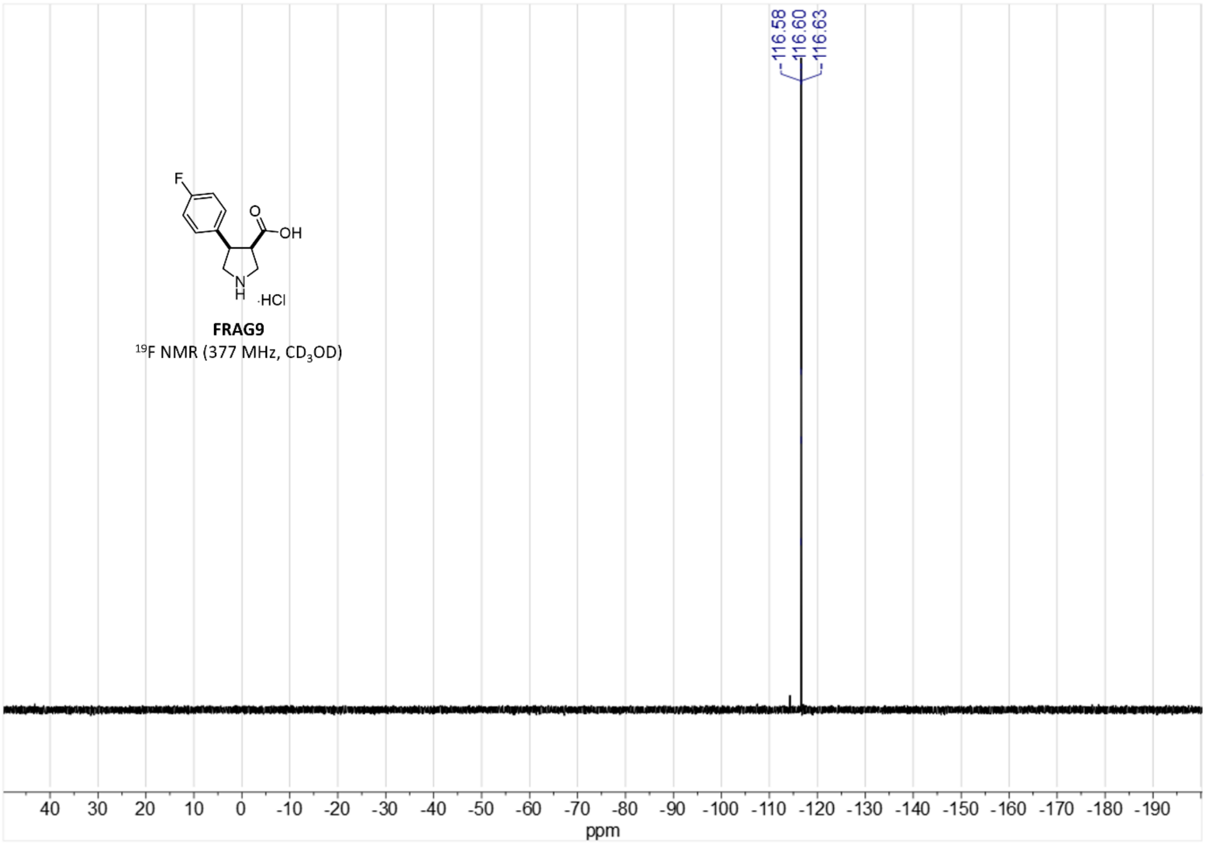
-116.58
-116.60
-116.63
F
O
OH
N
H
·HCl
FRAG9
19F NMR (377 MHz, CD3OD)
40 30 20 10 0 -10 -20 -30 -40 -50 -60 -70 -80 -90 -100 -110 -120 -130 -140 -150 -160 -170 -180 -190
ppm

***trans*-(±)-3-Carboxy-4-(4-fluorophenyl)pyrrolidin-1-ium chloride (FRAG11)**

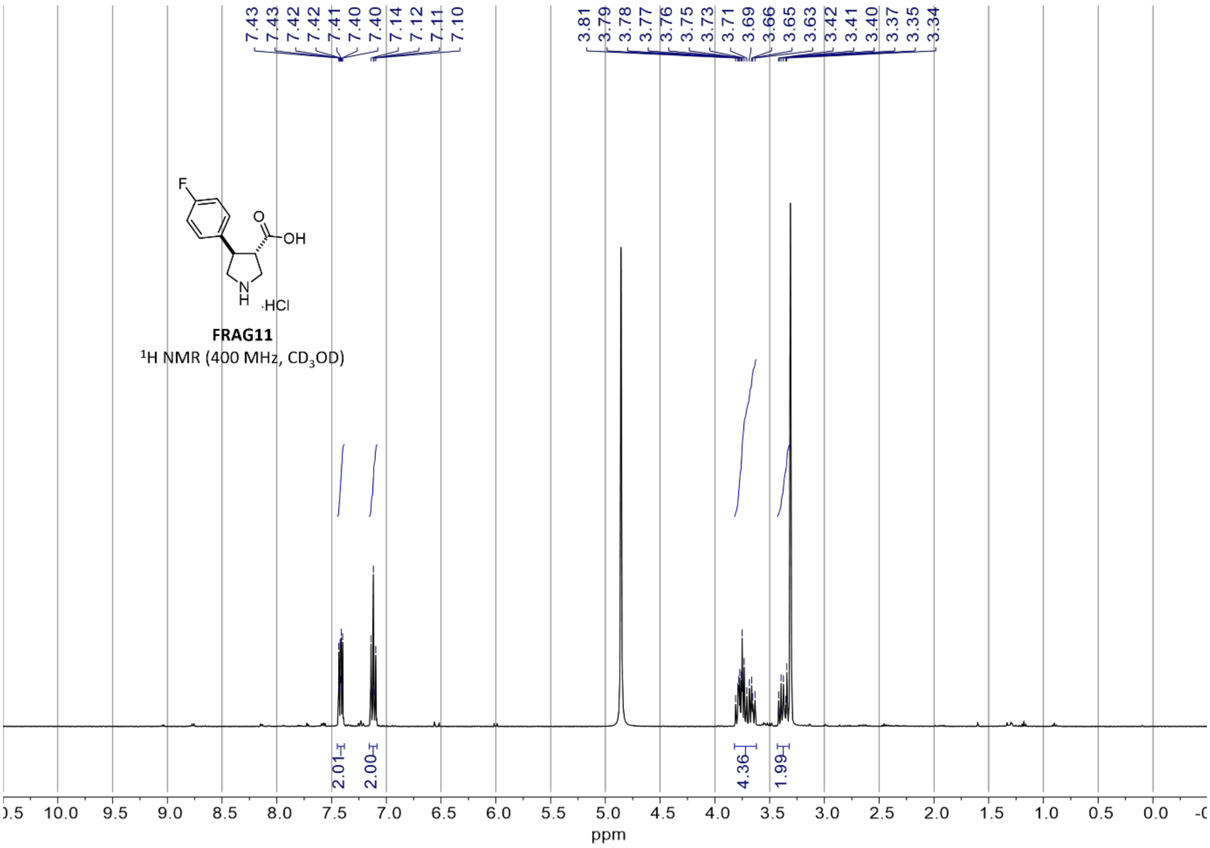

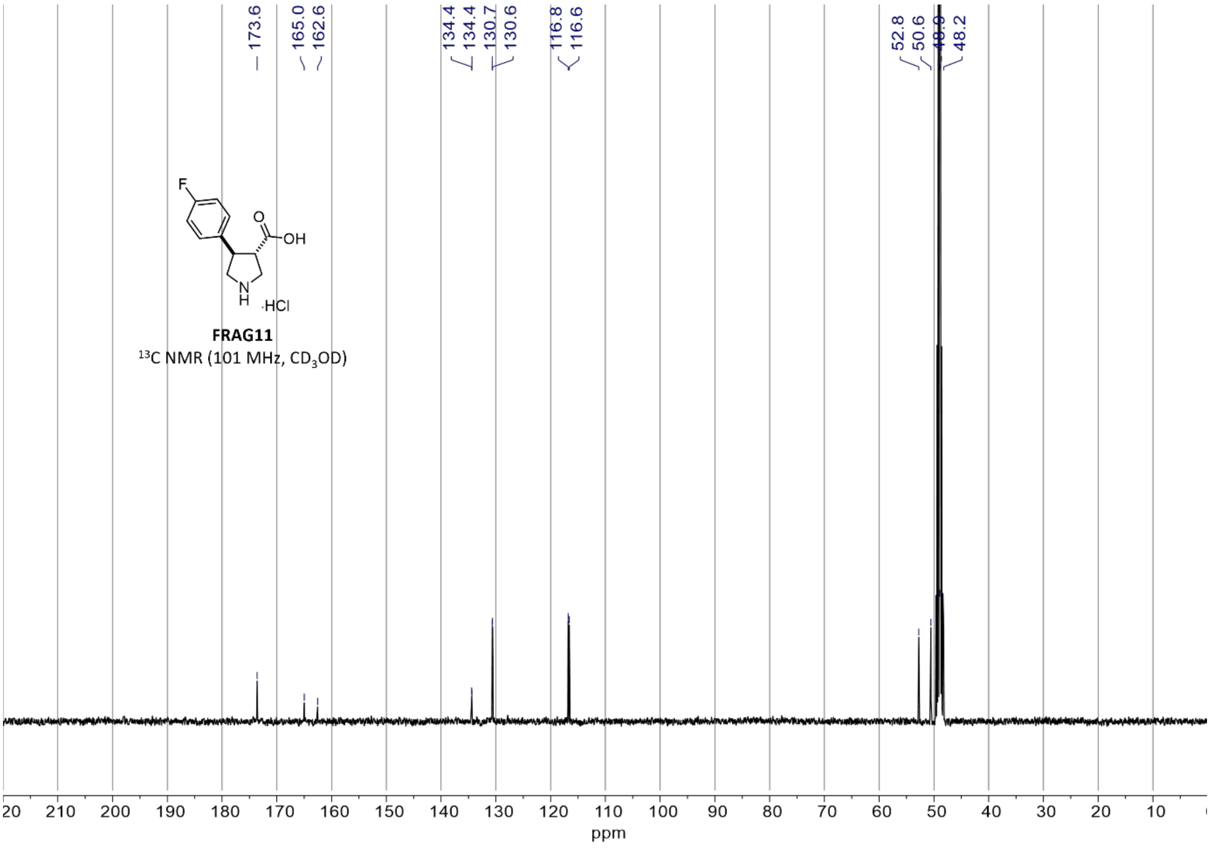

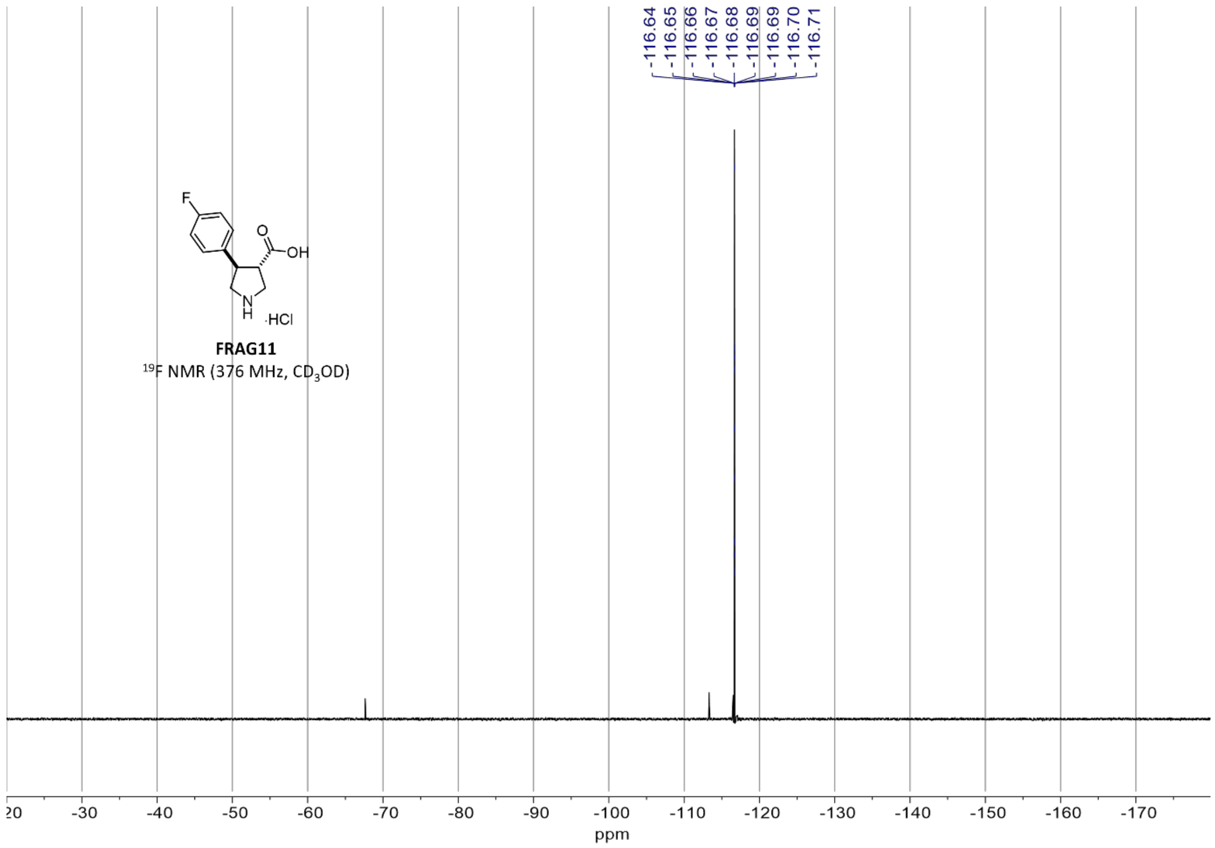
-116.64
-116.65
-116.66
-116.67
-116.68
-116.69
-116.70
-116.71
FRAG11
^{19}F NMR (376 MHz, CD_3OD)
-30 -40 -50 -60 -70 -80 -90 -100 -110 -120 -130 -140 -150 -160 -170
ppm

trans-(±)-3-(4-Fluorophenyl)-4-(methoxycarbonyl)pyrrolidin-1-ium chloride (S3)

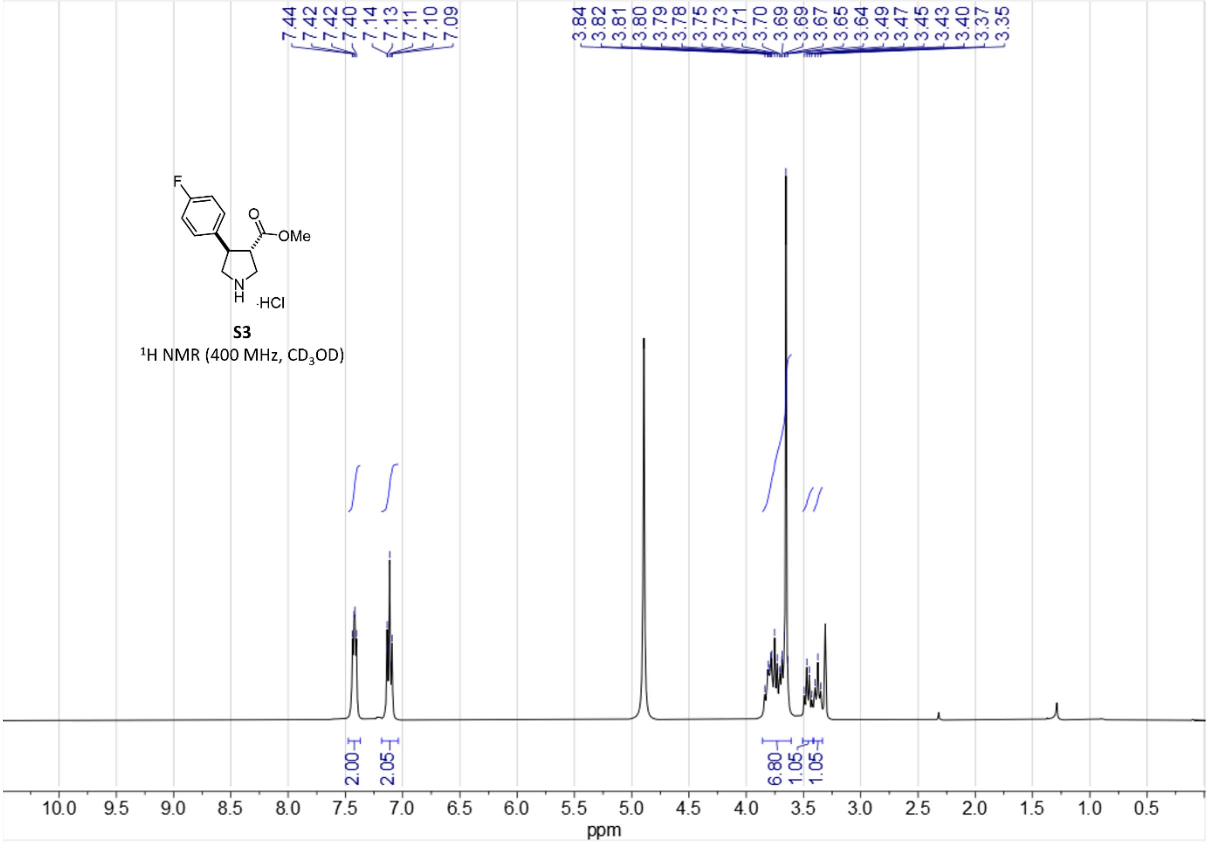

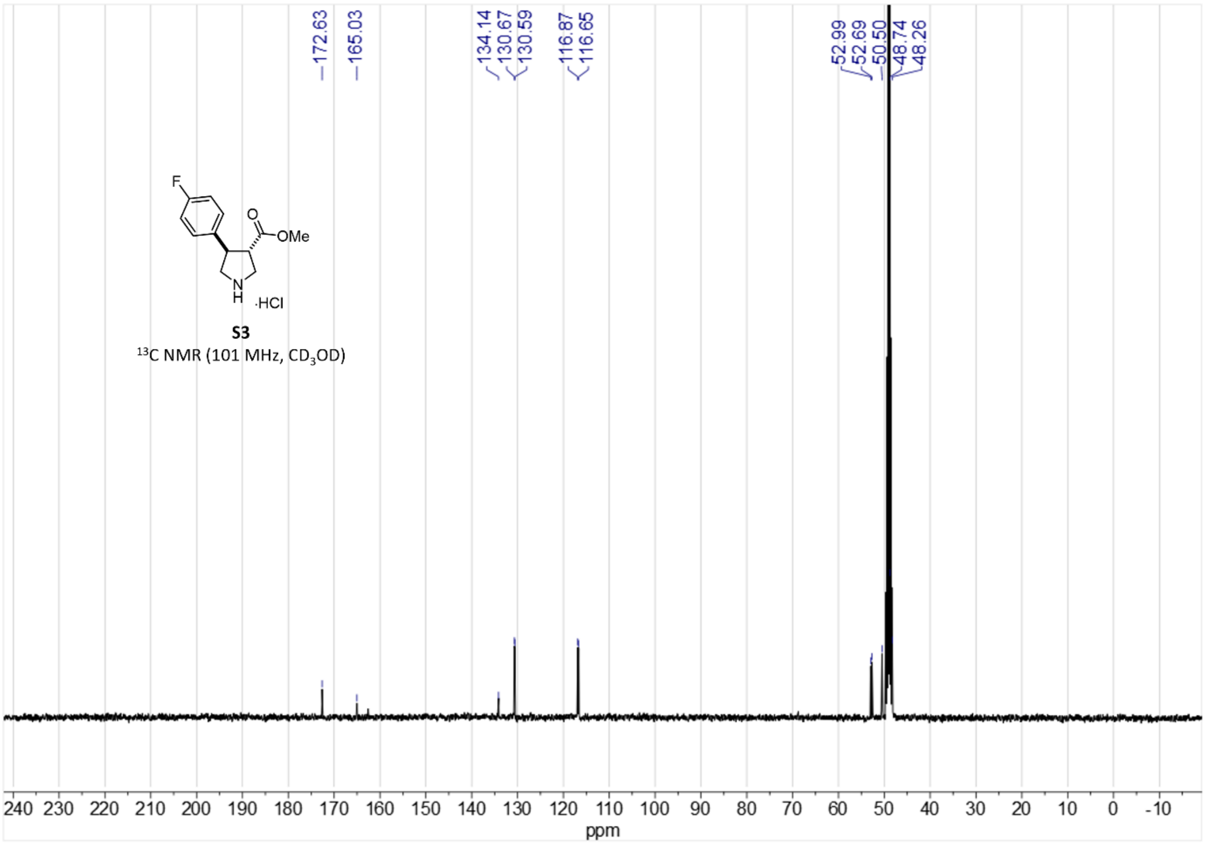

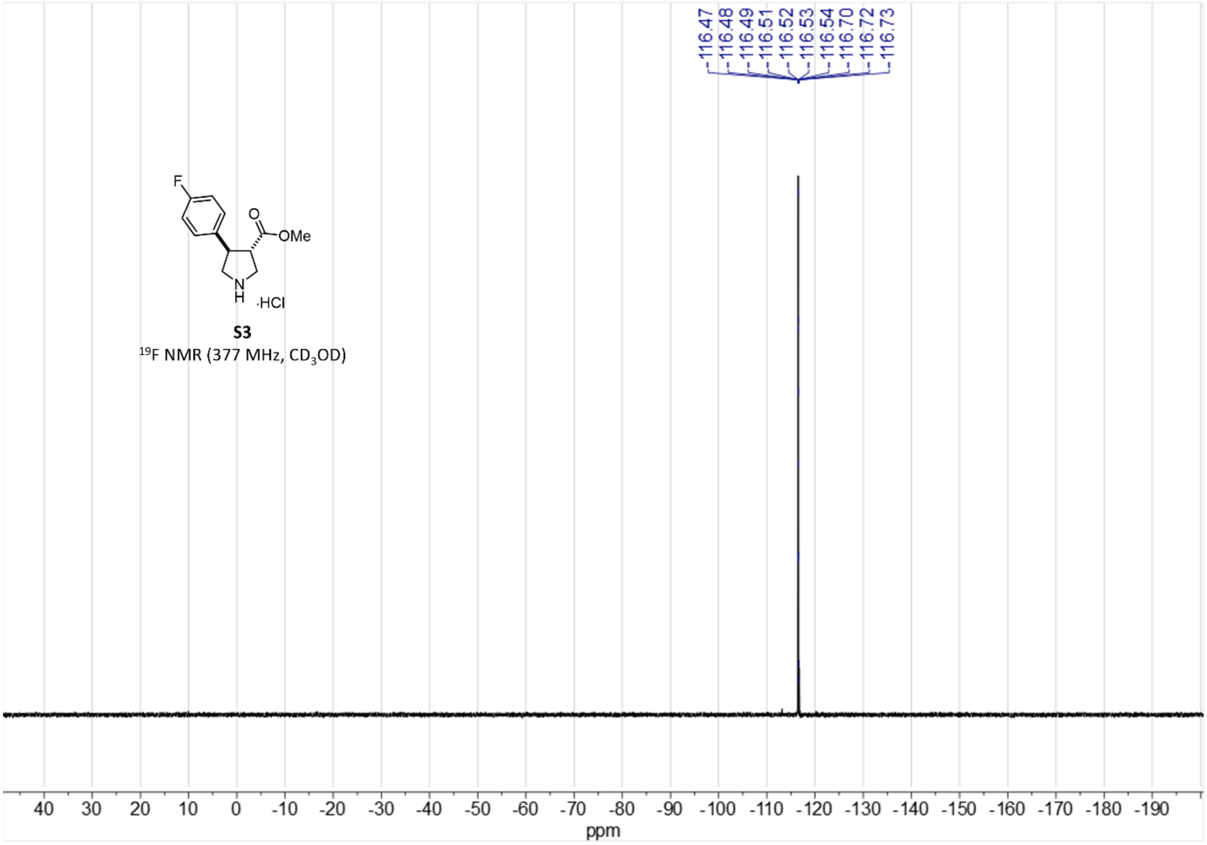
-116.47
-116.48
-116.49
-116.51
-116.52
-116.53
-116.54
-116.70
-116.72
-116.73
F
O
OMe
N
H
·HCl
S3
19F NMR (377 MHz, CD3OD)
40 30 20 10 0 -10 -20 -30 -40 -50 -60 -70 -80 -90 -100 -110 -120 -130 -140 -150 -160 -170 -180 -190
ppm

trans-(±)-3-Carbamoyl-4-(4-methoxyphenyl)pyrrolidin-1-ium chloride (FRAG14)

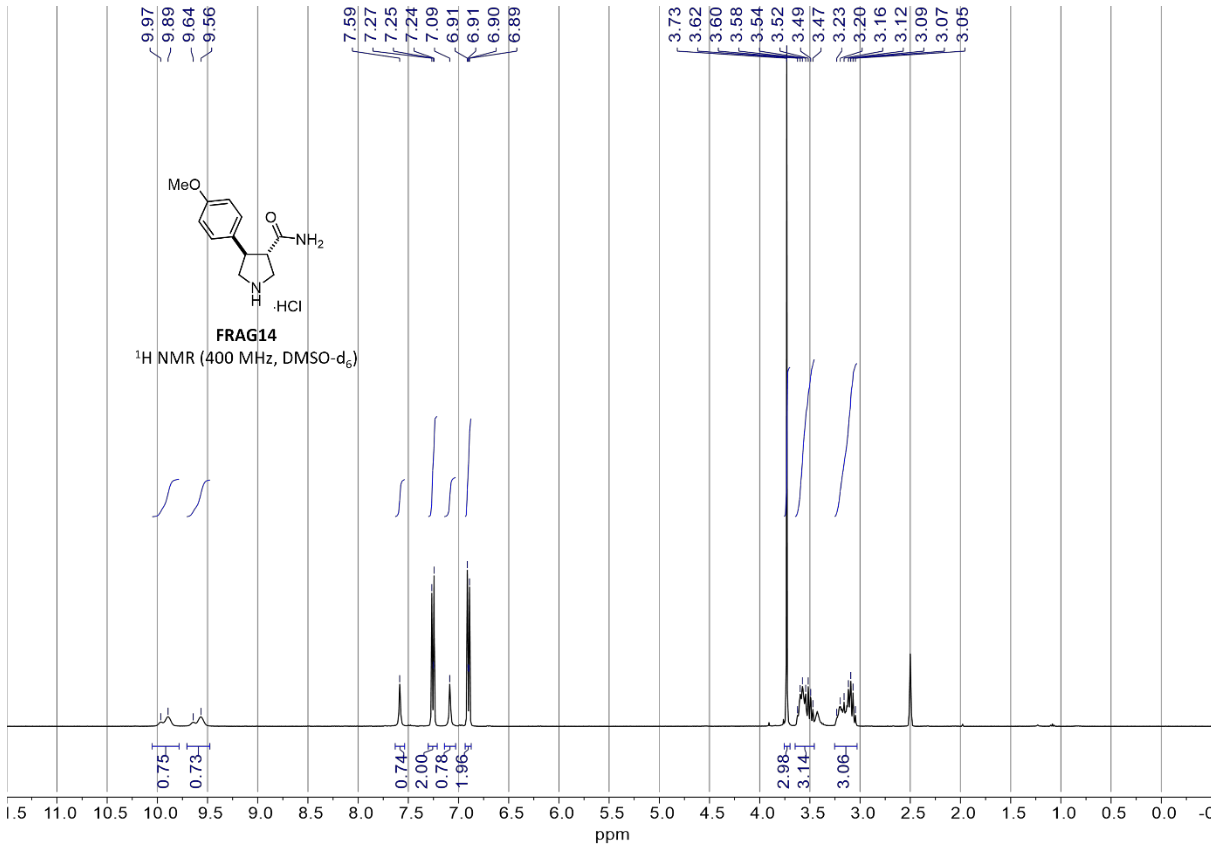

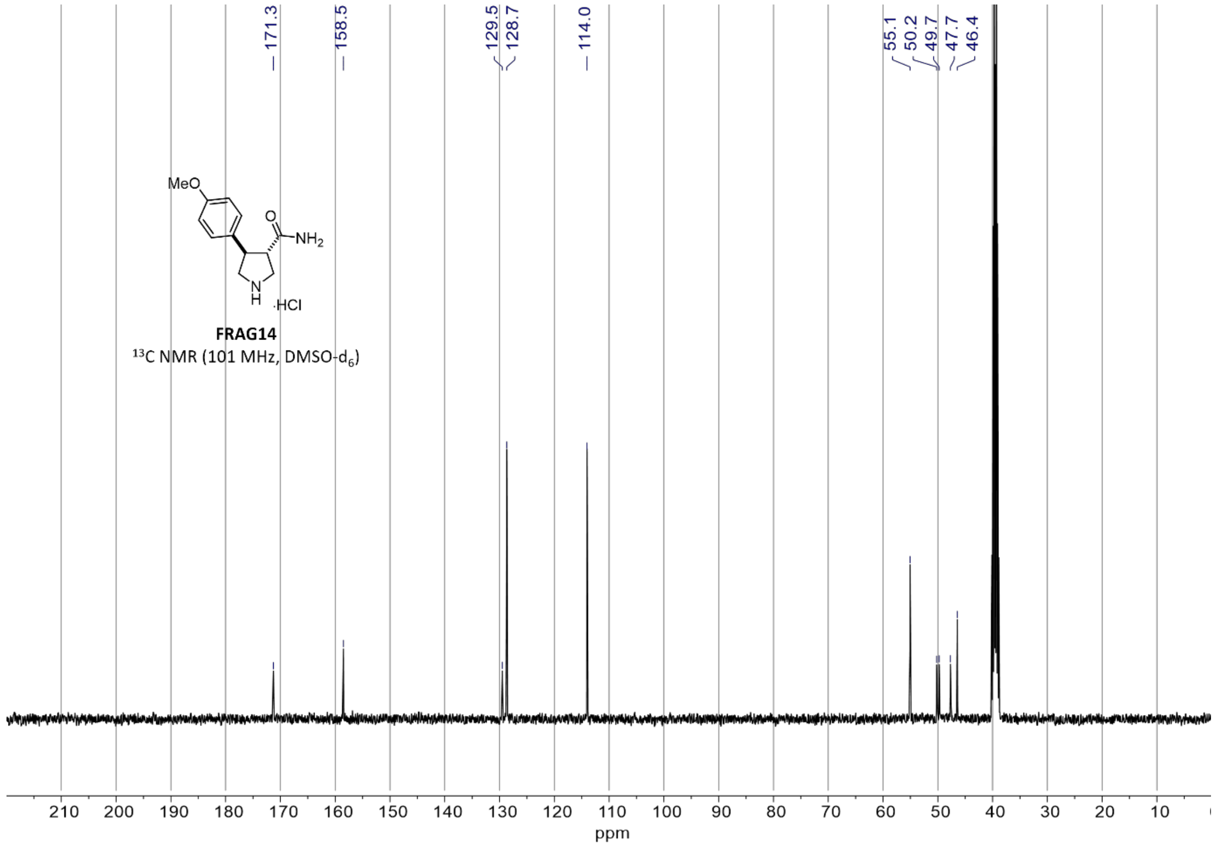

***cis*-(±)-3-Carbamoyl-4-(4-fluorophenyl)pyrrolidin-1-ium chloride (FRAG13)**

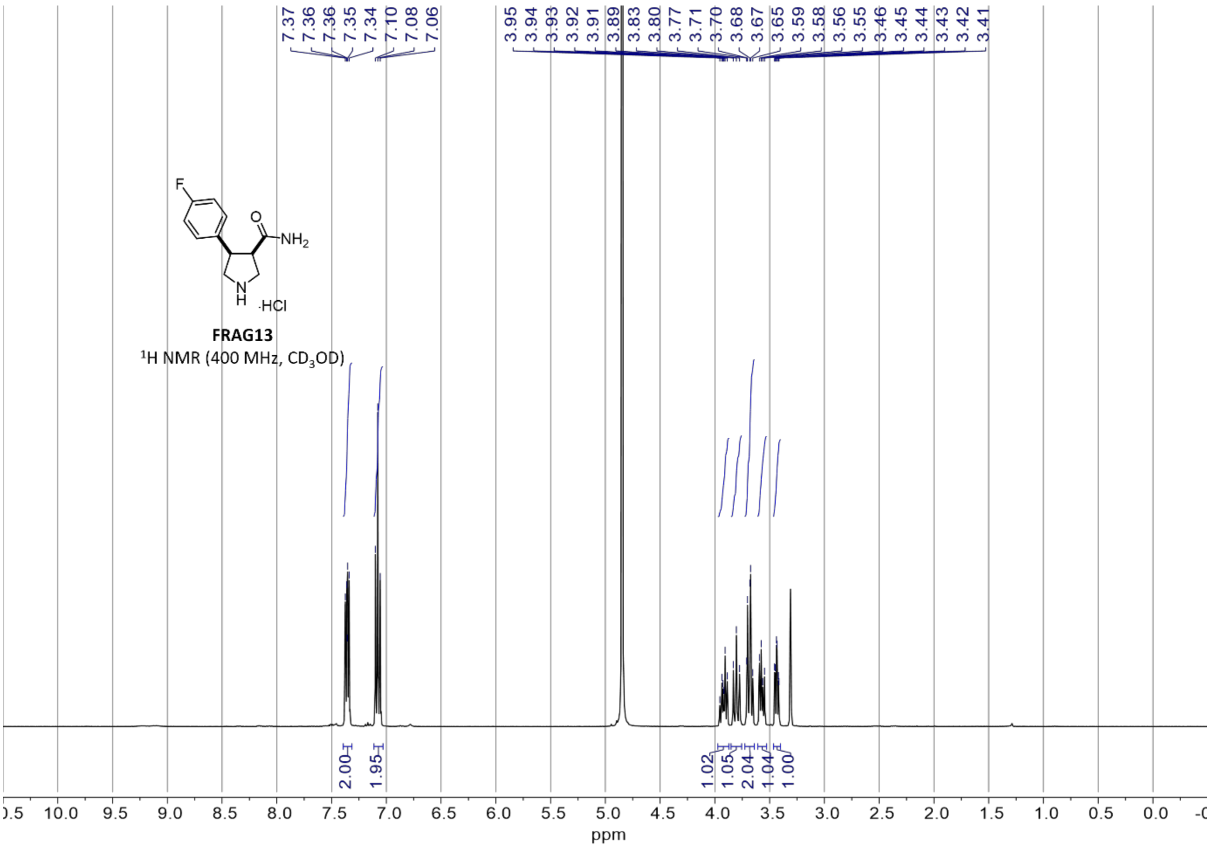

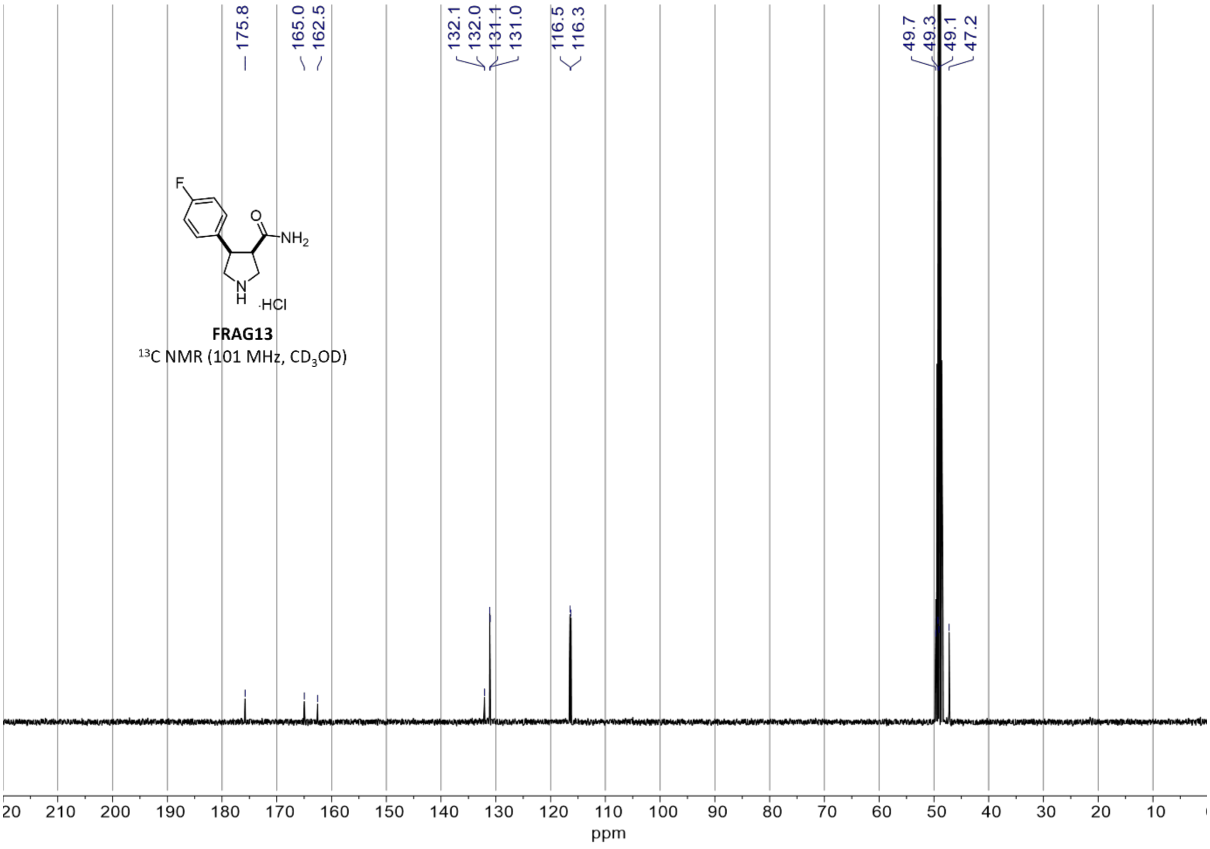

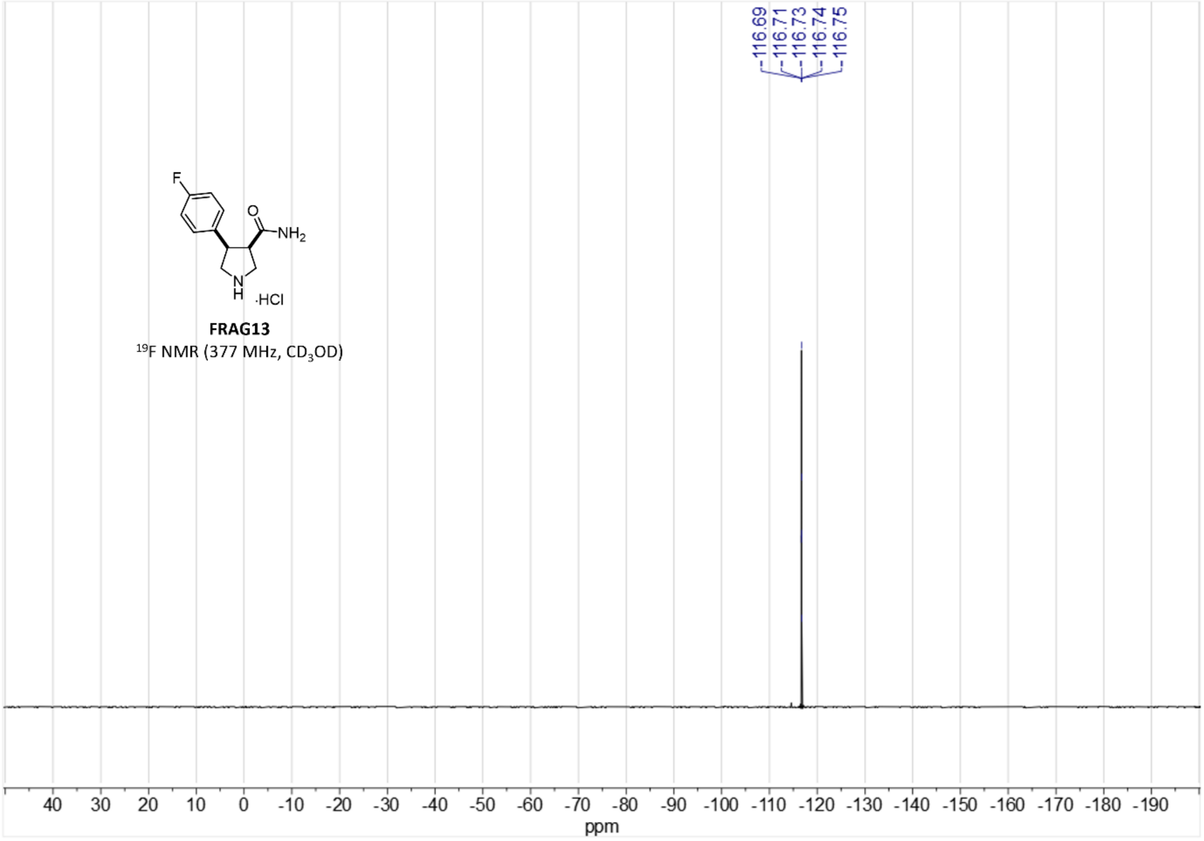

FRAG13
^{19}F NMR (377 MHz, CD_3OD)

***trans*-(±)-3-Carbamoyl-4-(4-fluorophenyl)pyrrolidin-1-ium chloride (FRAG15)**

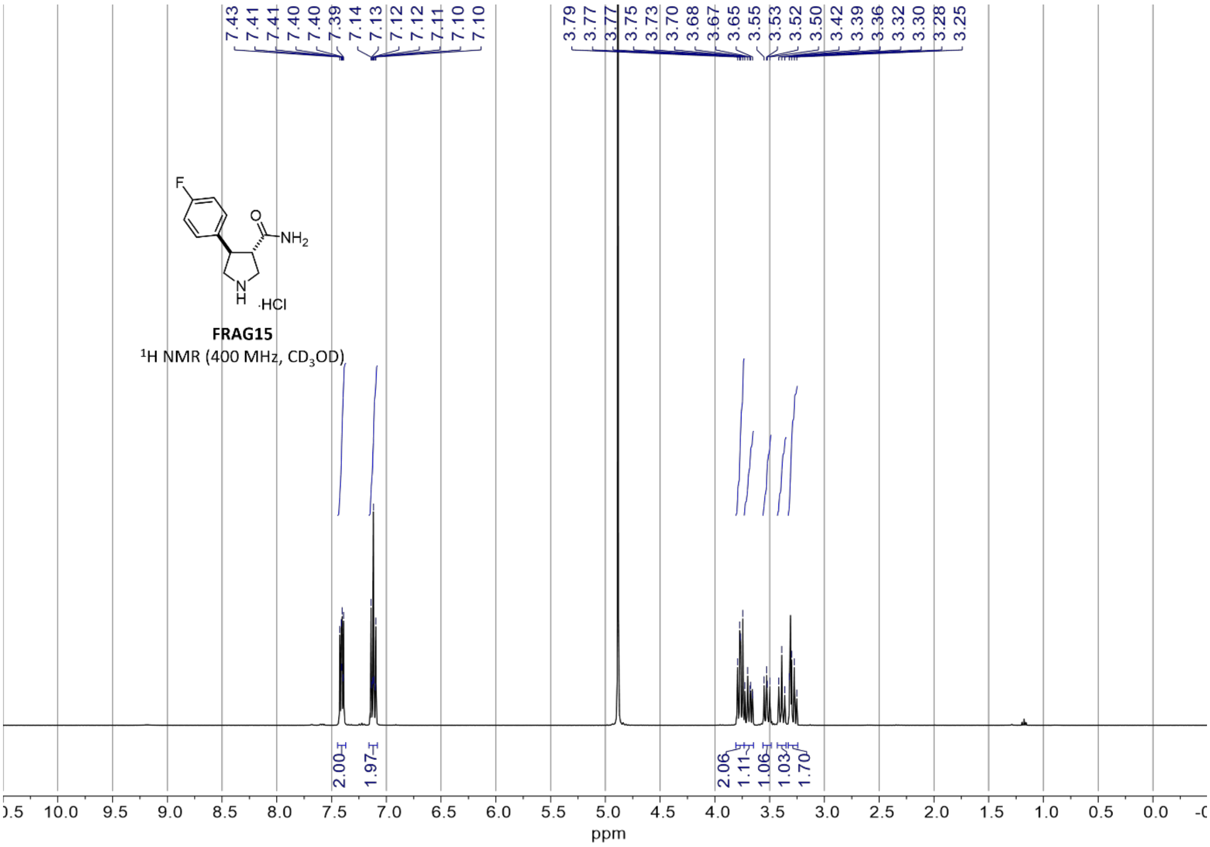

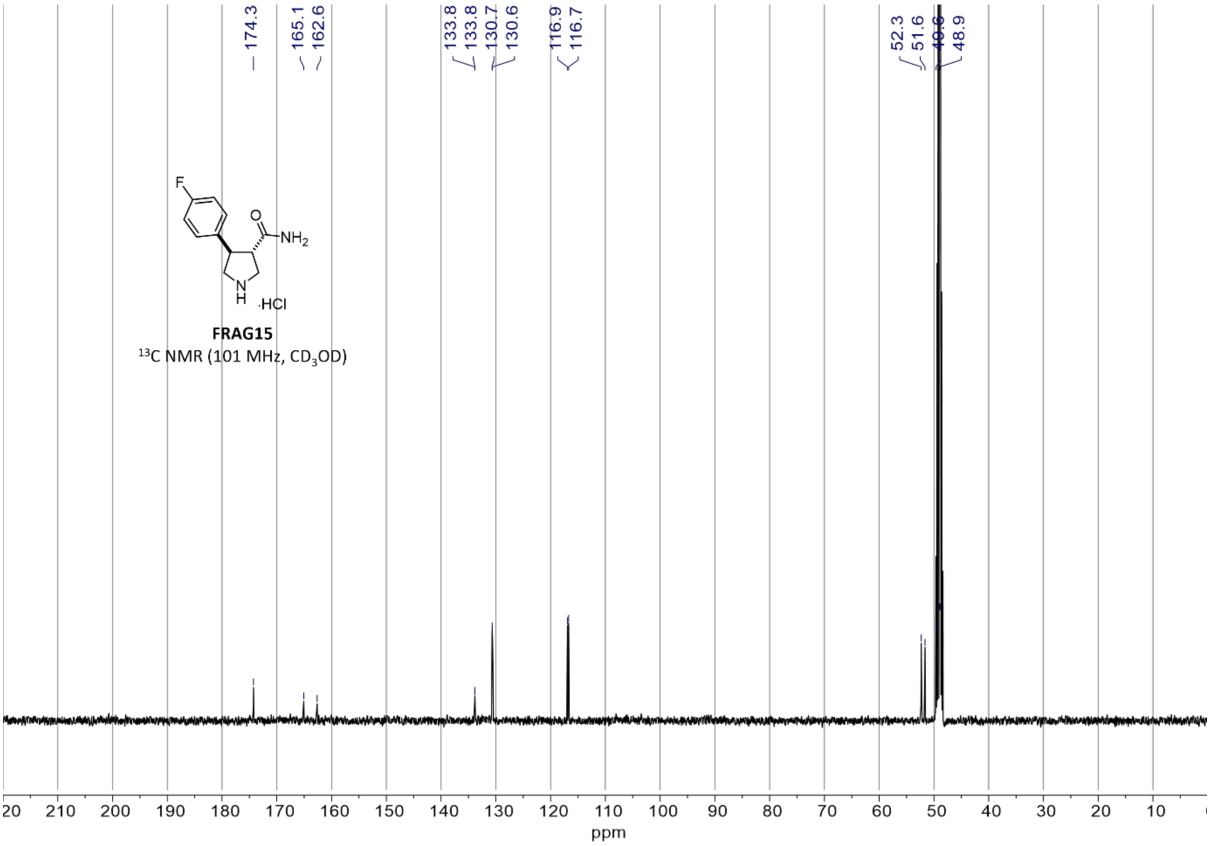

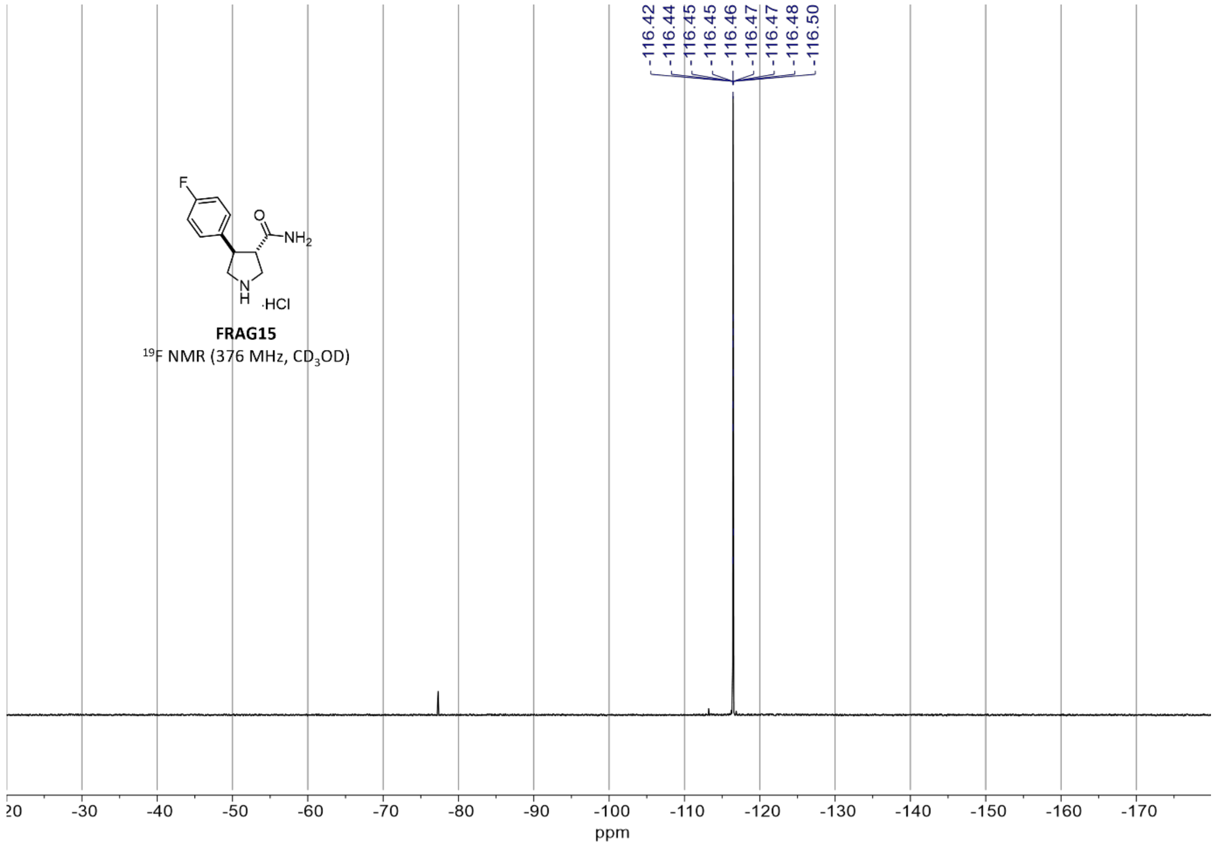

FRAG15

^{19}F NMR (376 MHz, CD_3OD)

cis-(±)-3-(Hydroxymethyl)-4-(4-methoxyphenyl)pyrrolidin-1-ium chloride (FRAG16)

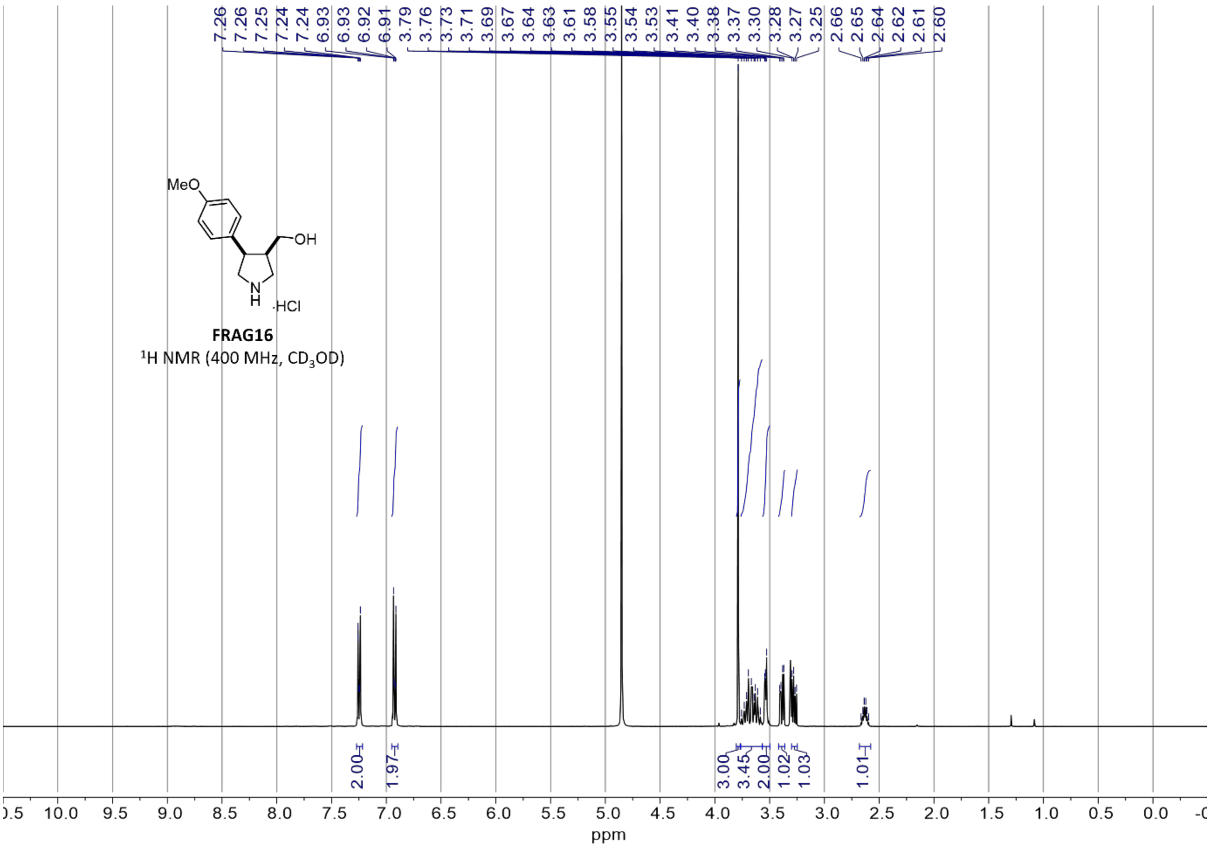

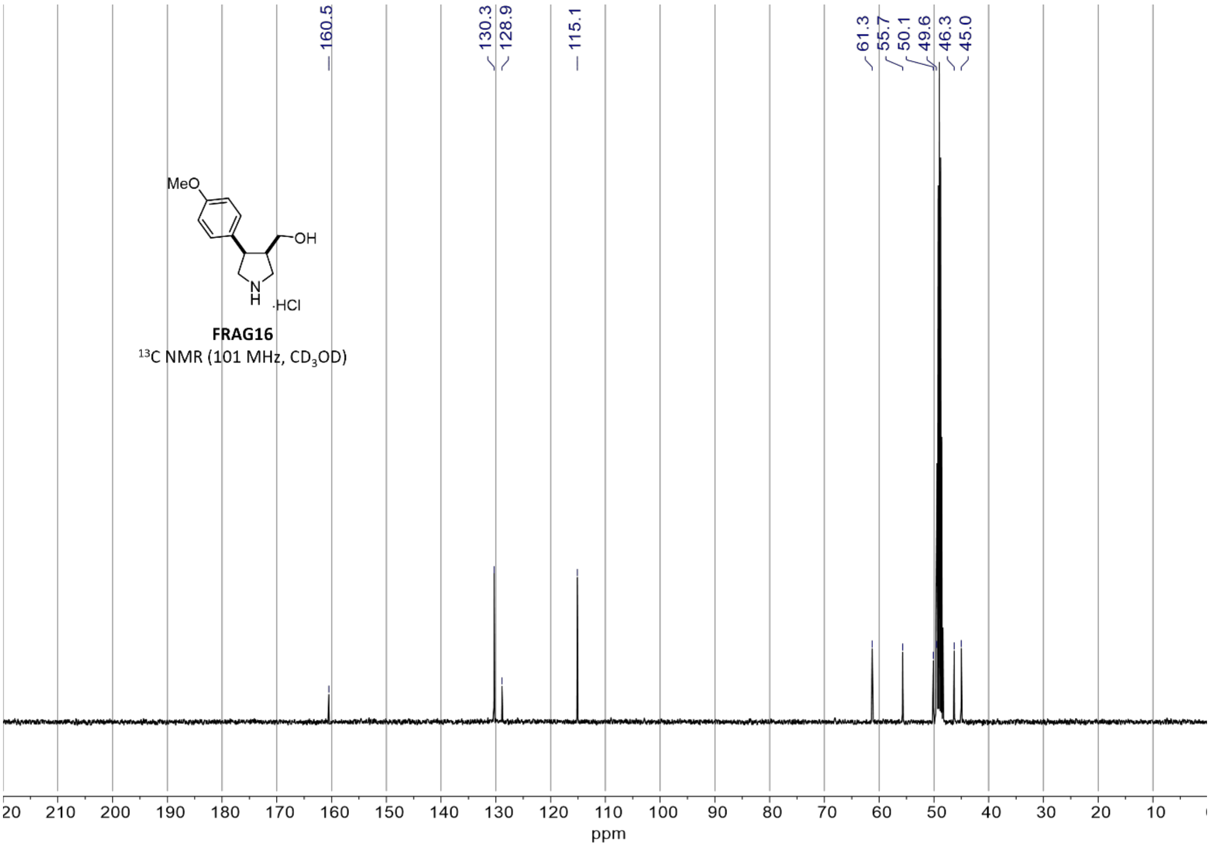

***trans*-(±)-3-(Hydroxymethyl)-4-(4-methoxyphenyl)pyrrolidin-1-ium chloride (FRAG18)**

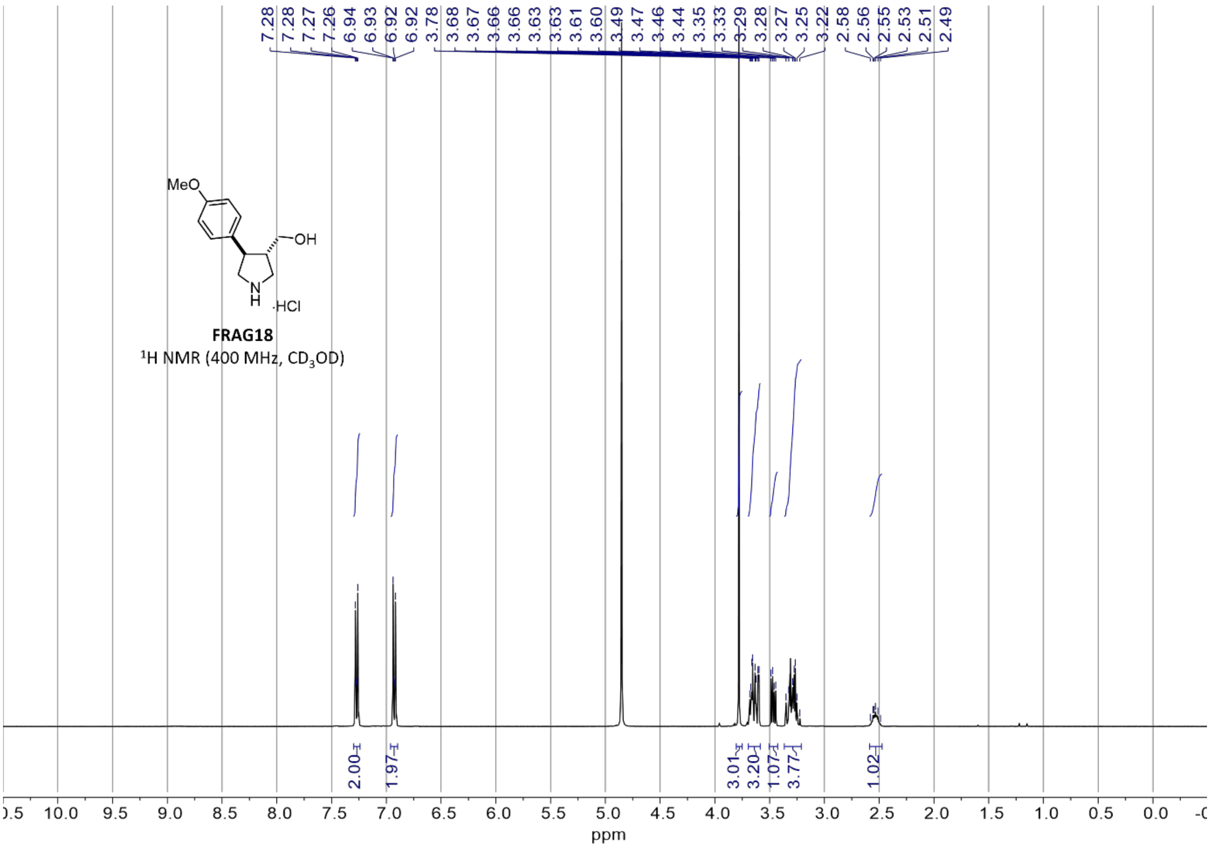

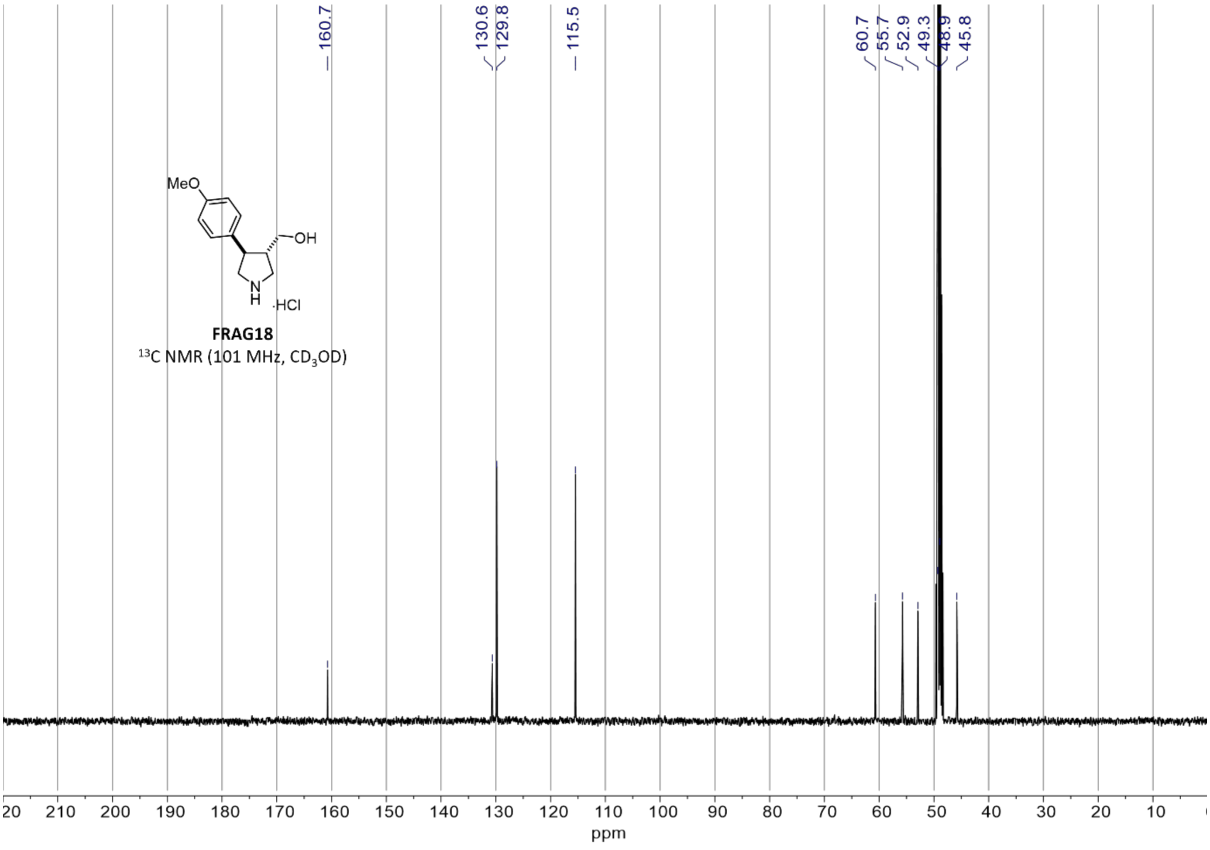

cis-(±)-3-(Hydroxymethyl)-4-(4-fluorophenyl)pyrrolidin-1-ium chloride (FRAG17)

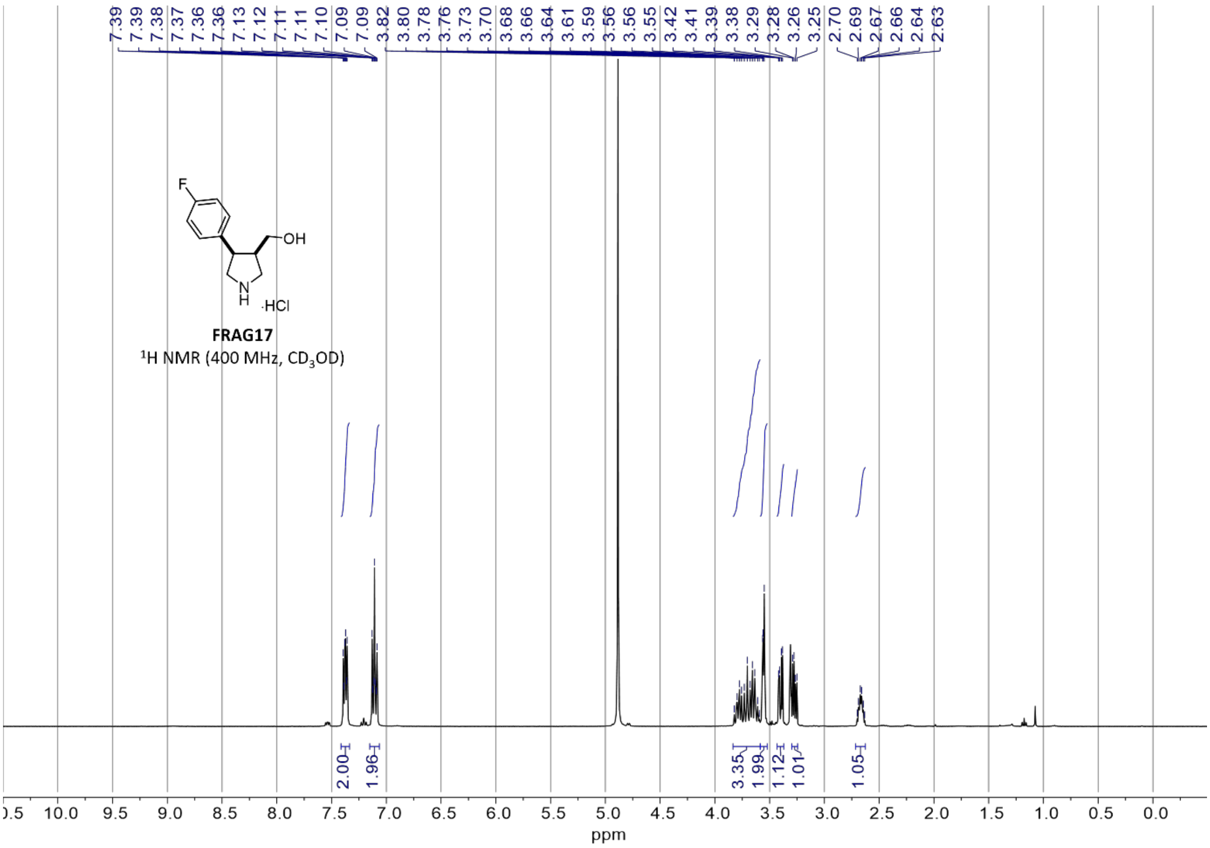

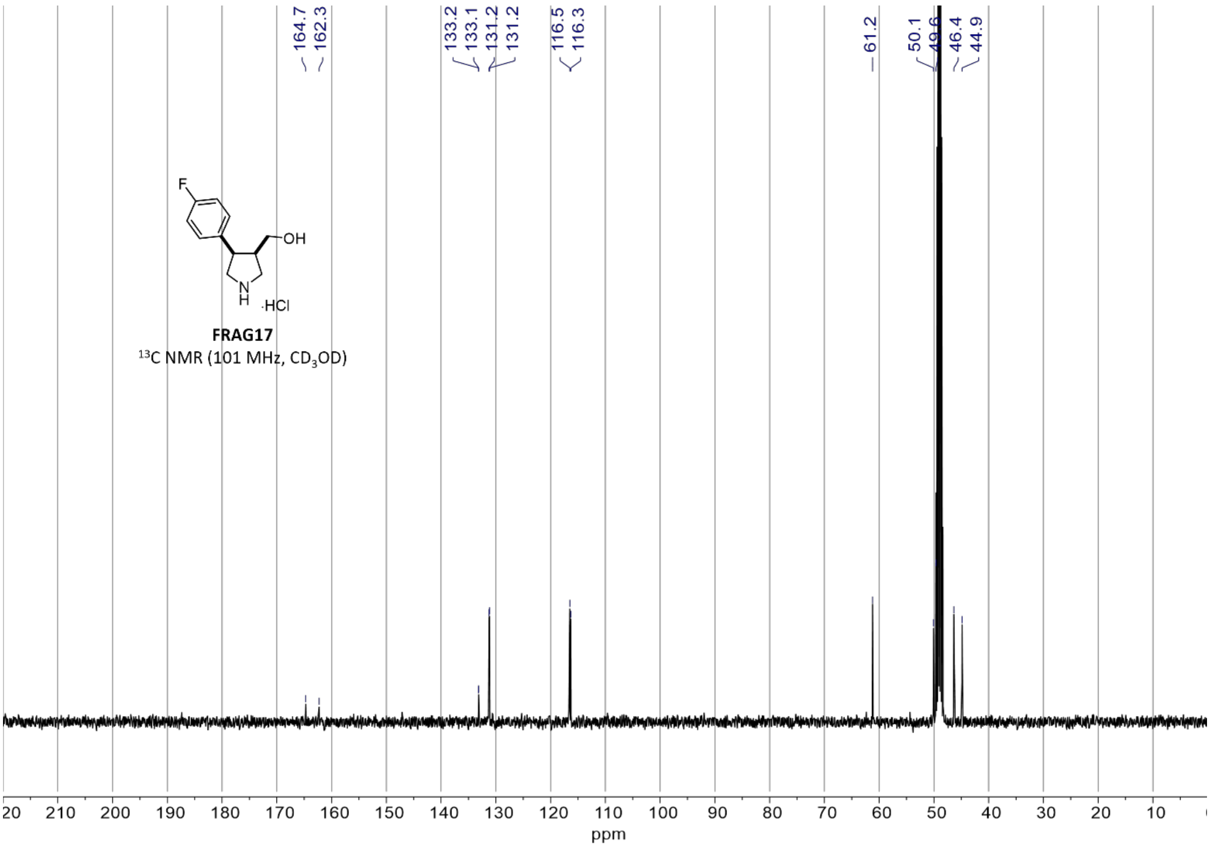

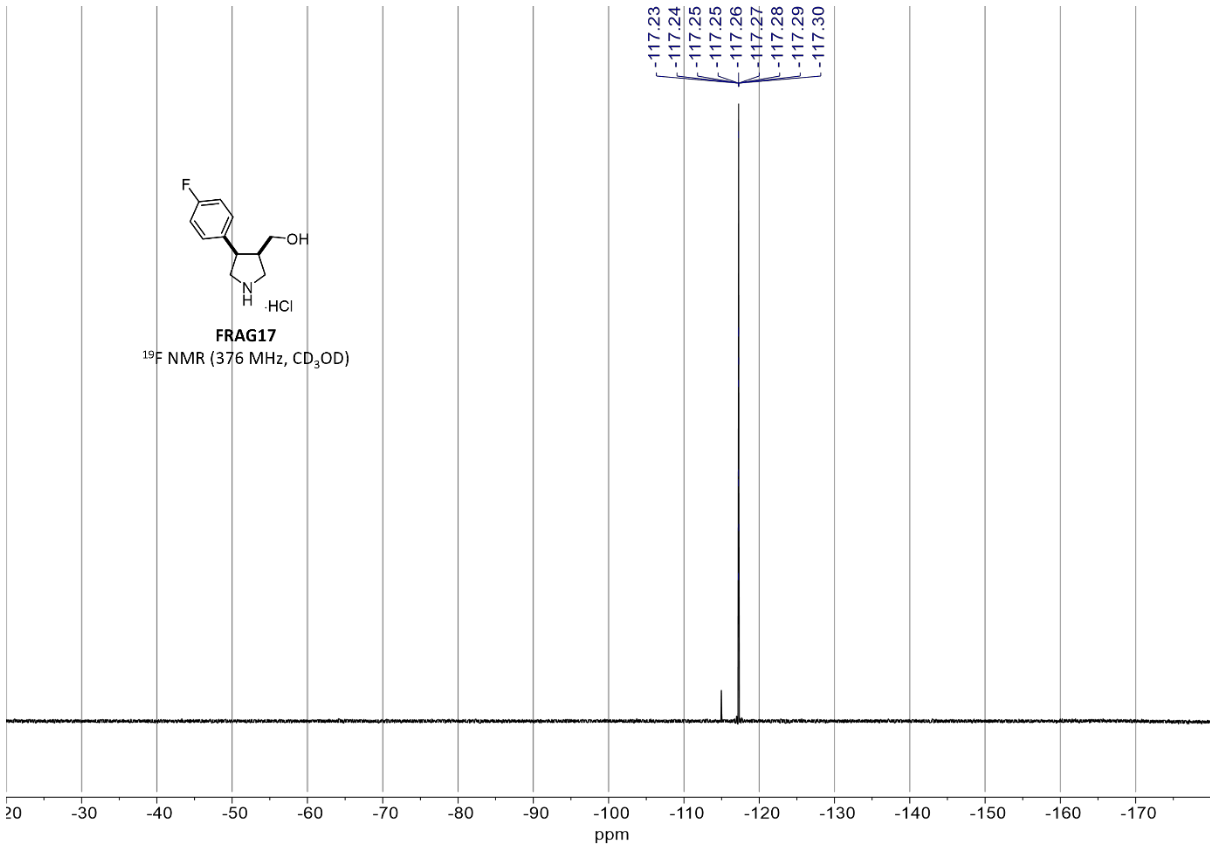

-117.23
-117.24
-117.25
-117.25
-117.26
-117.27
-117.28
-117.29
-117.30
F
OH
N
H
·HCl
FRAG17
^{19}F NMR (376 MHz, CD_3OD)
-20
-30
-40
-50
-60
-70
-80
-90
-100
-110
-120
-130
-140
-150
-160
-170
ppm

***trans*-(±)-3-(Hydroxymethyl)-4-(4-fluorophenyl)pyrrolidin-1-ium chloride (FRAG19)**

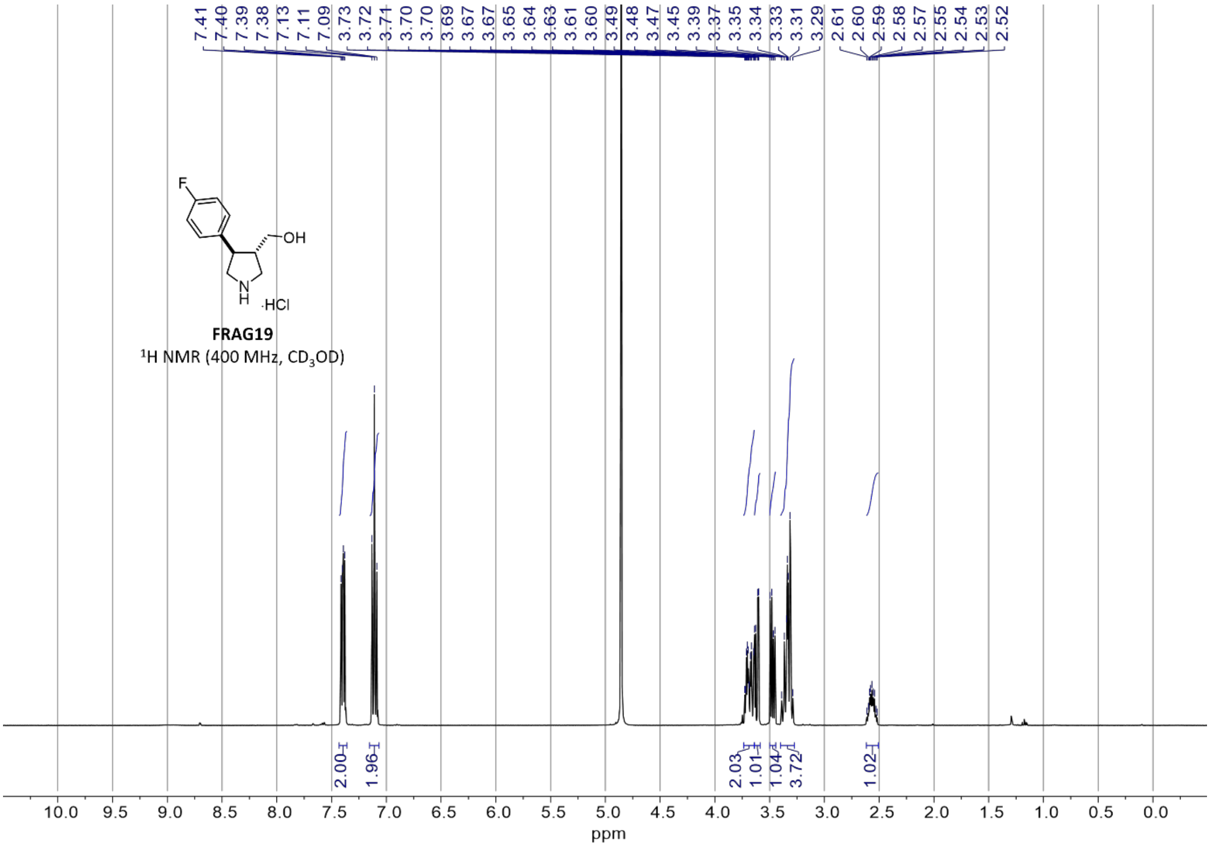

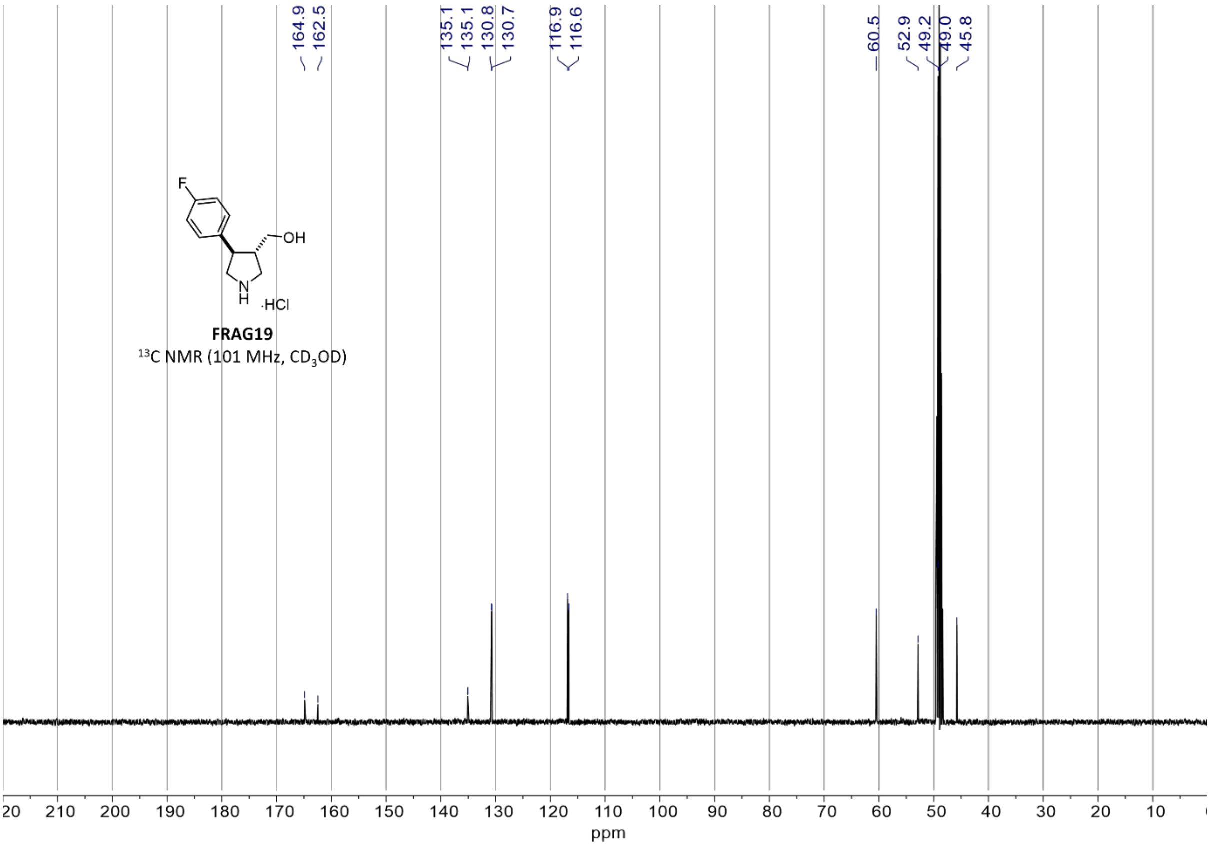

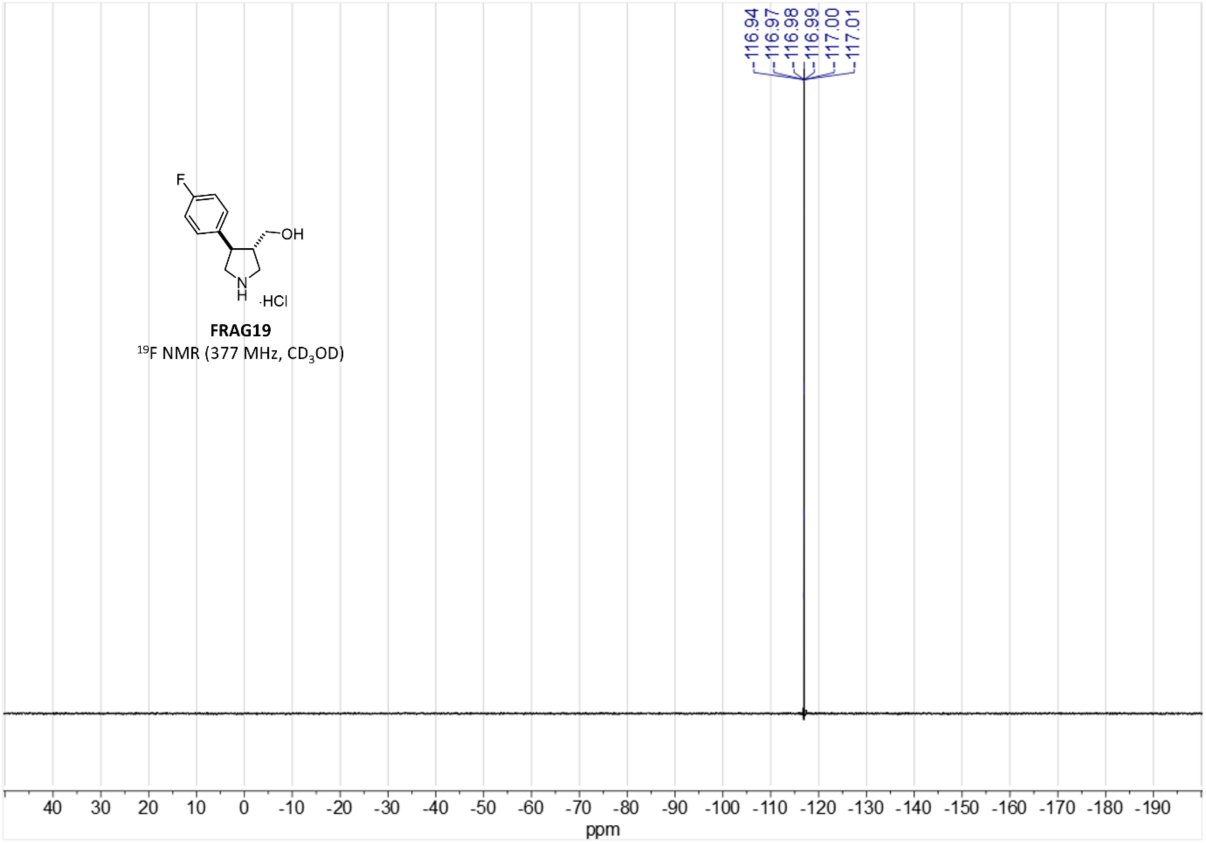
-116.94
-116.97
-116.98
-116.99
-117.00
-117.01
F
OH
N
H
·HCl
FRAG19
^{19}F NMR (377 MHz, CD_3OD)
40 30 20 10 0 -10 -20 -30 -40 -50 -60 -70 -80 -90 -100 -110 -120 -130 -140 -150 -160 -170 -180 -190
ppm

cis-(±)-(*tert*-Butoxycarbonyl)-4-(4-methoxyphenyl)piperidine-3-carboxylic acid (35a)

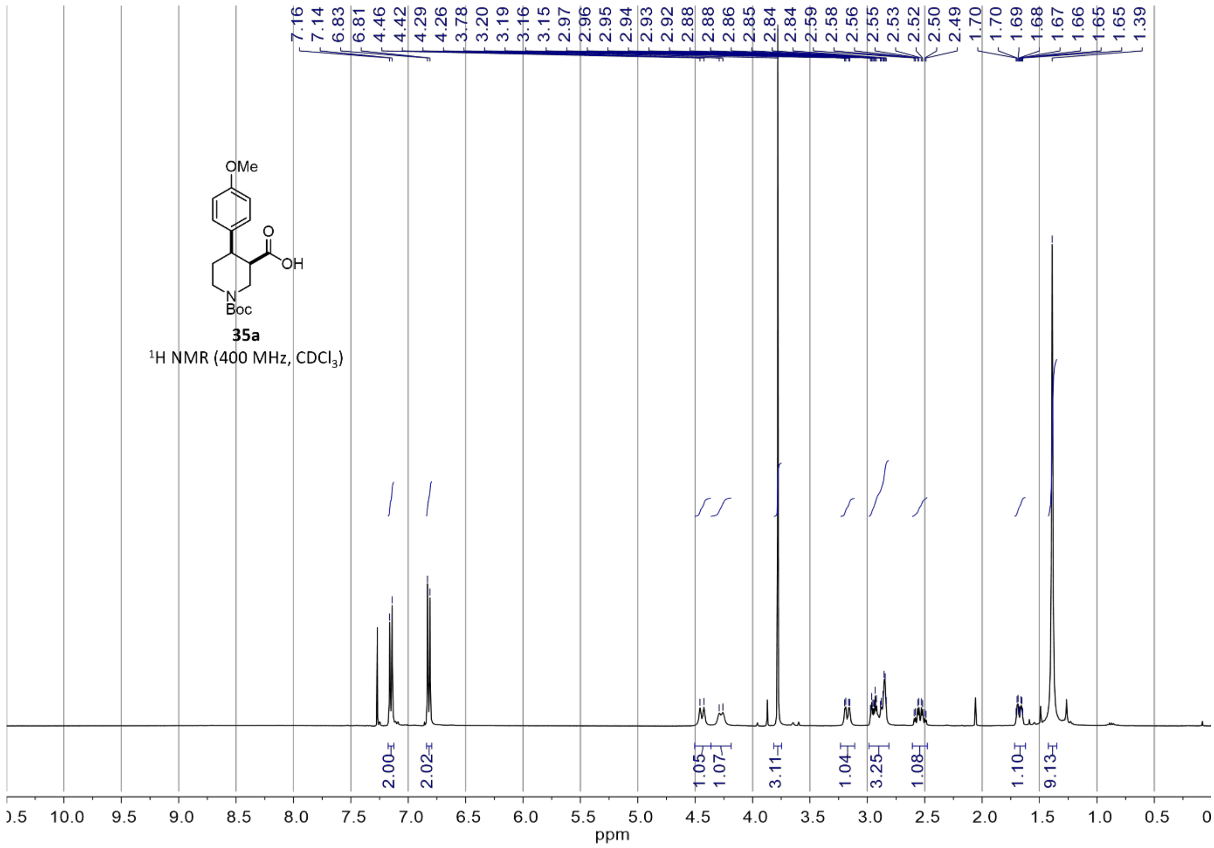

35a

^{1}H NMR (400 MHz, $CDCl_3$)

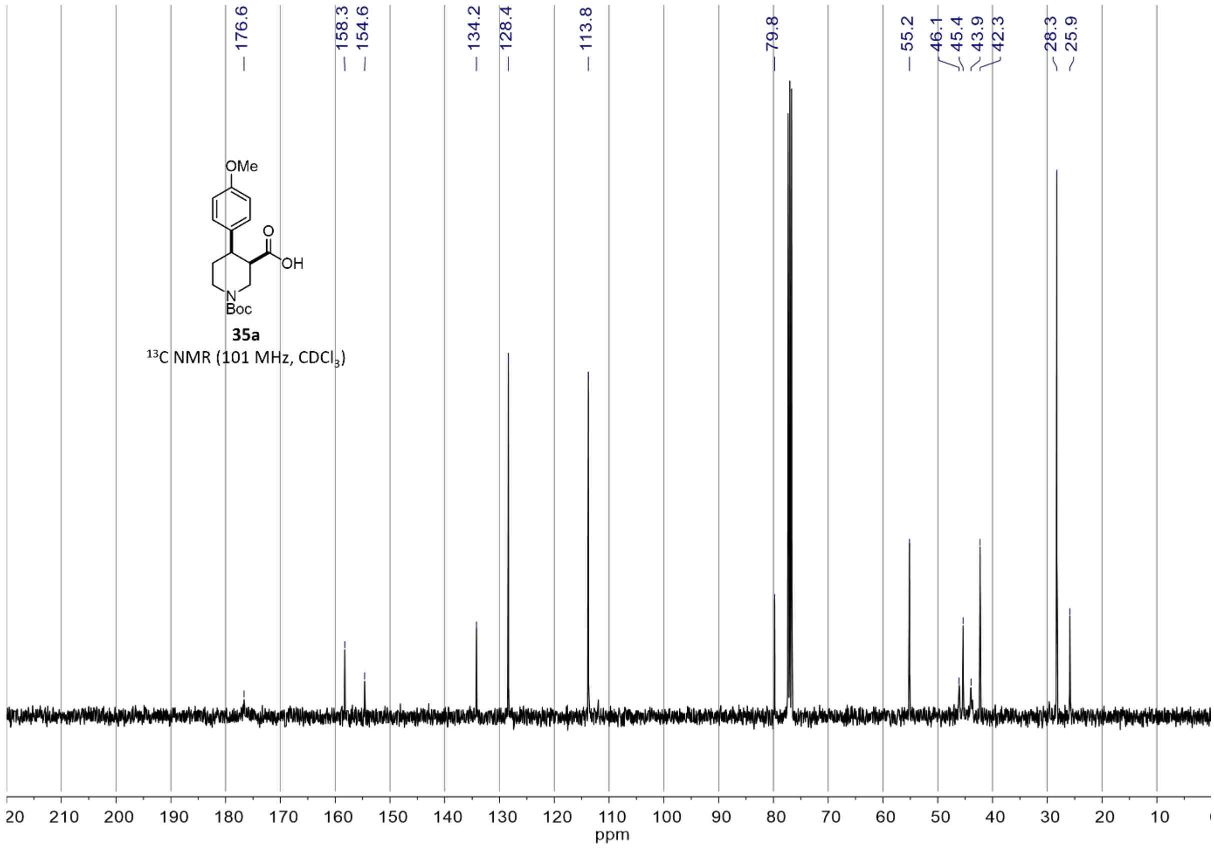

35a

^{13}C NMR (101 MHz, $CDCl_3$)

***trans*-(±)-1-(*tert*-Butoxycarbonyl)-4-(4-methoxyphenyl)piperidine-3-carboxylic acid (36a)**

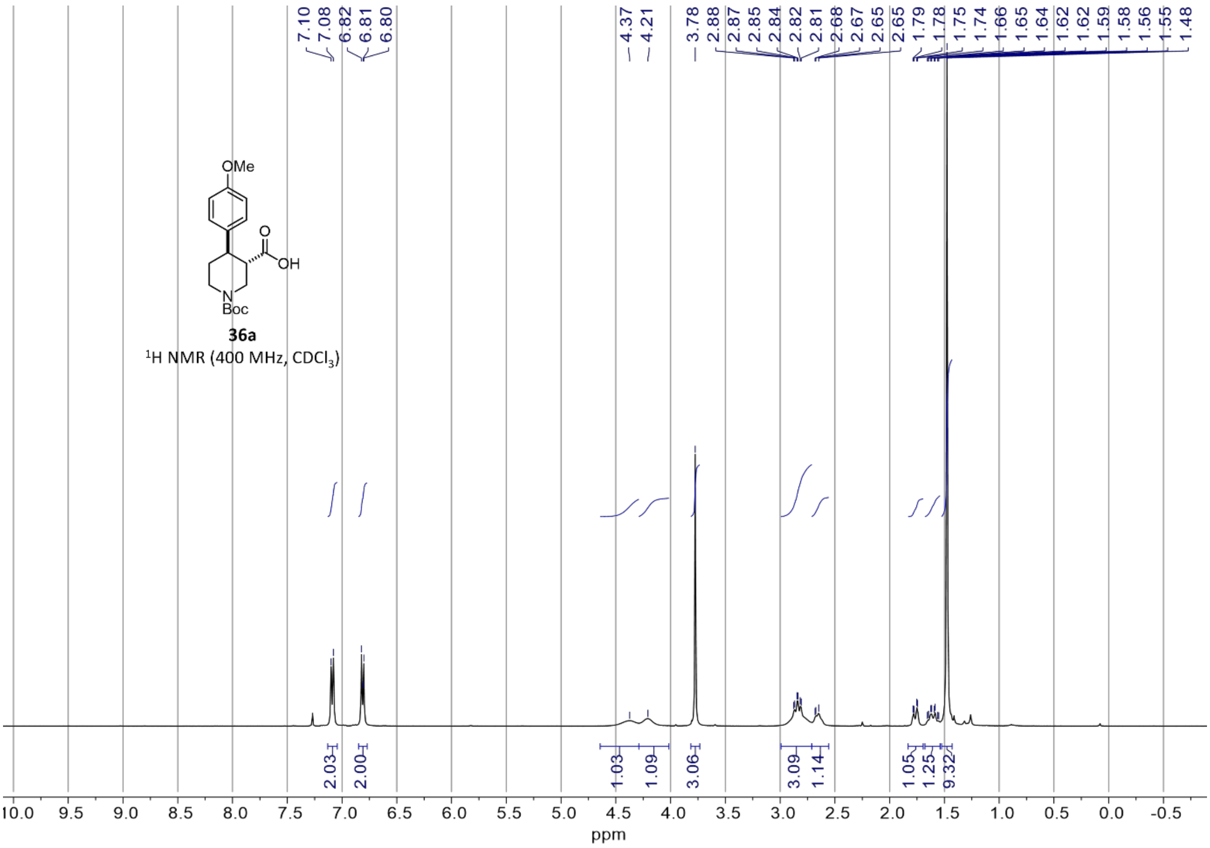

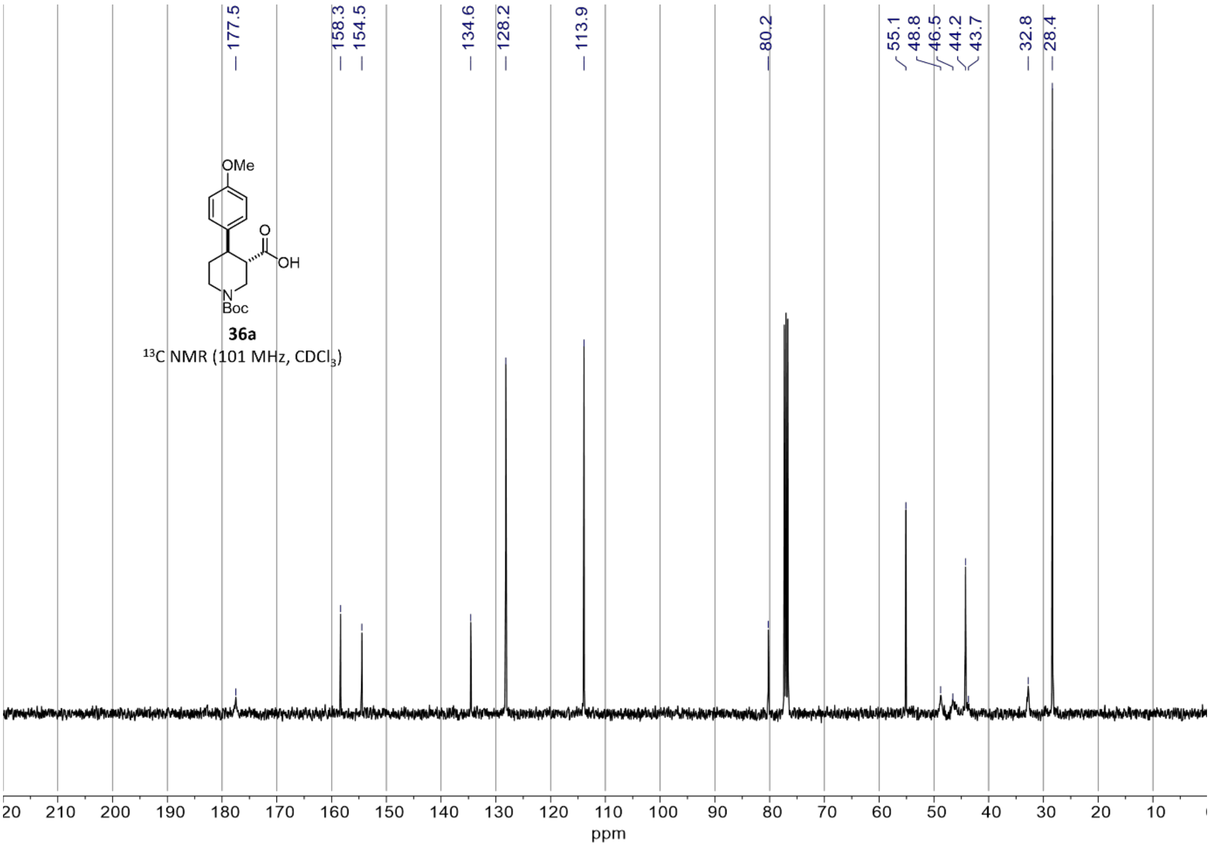

***trans*-(±)-(*tert*-Butoxycarbonyl)-4-(4-fluorophenyl)piperidine-3-carboxylic acid (36b)**

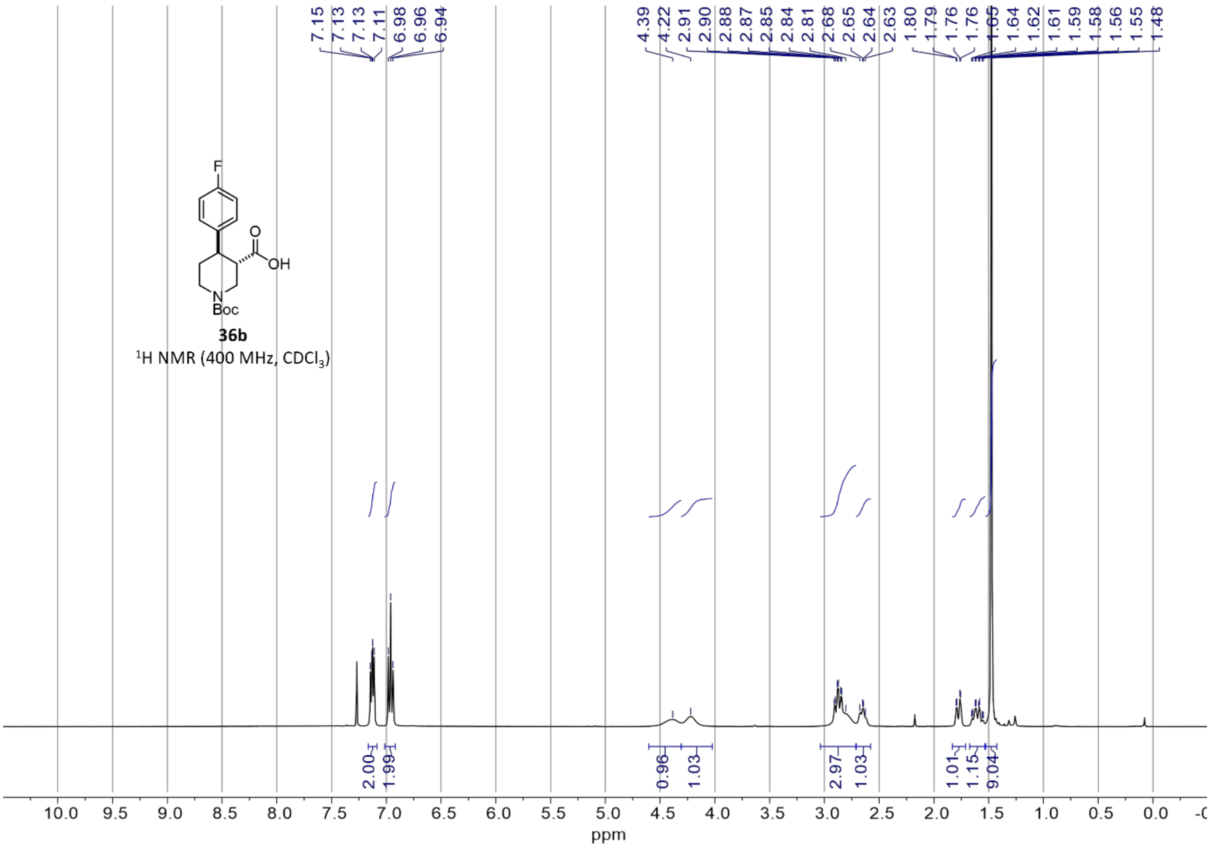

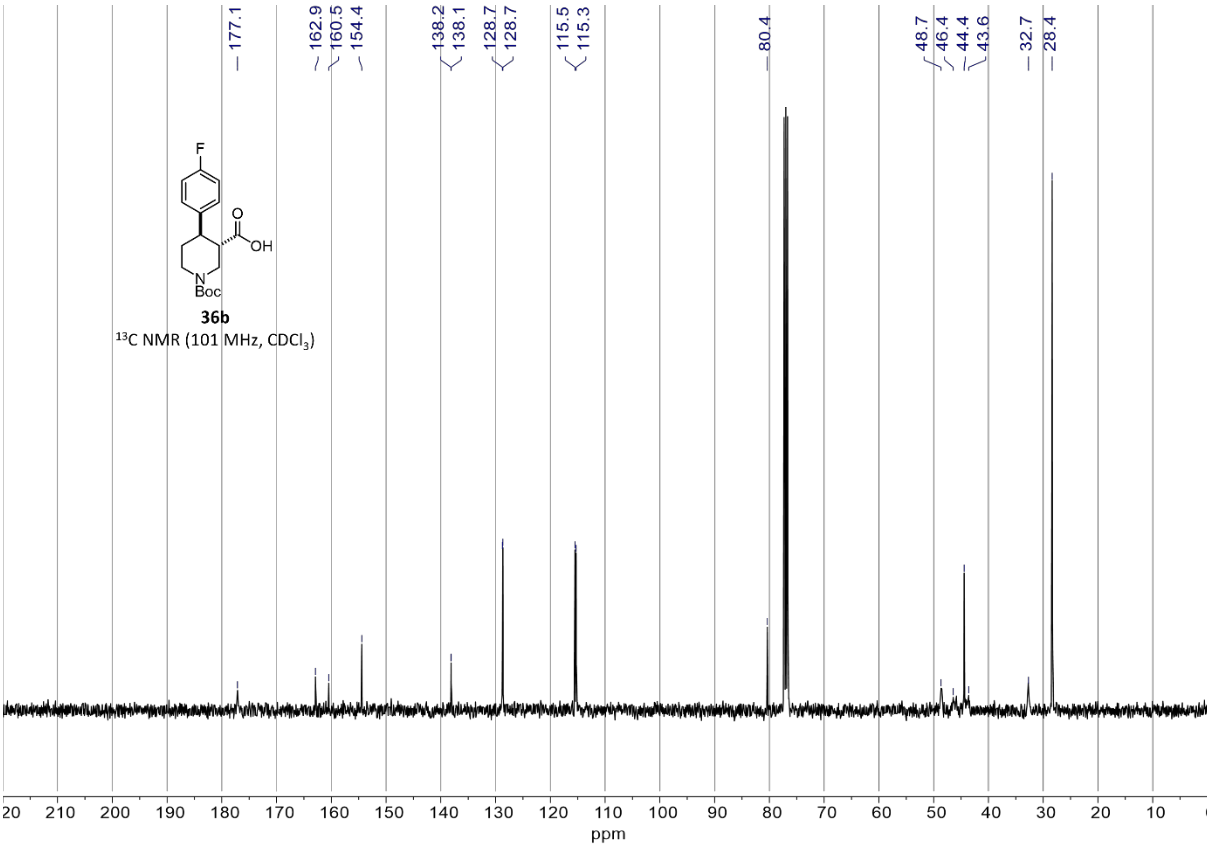

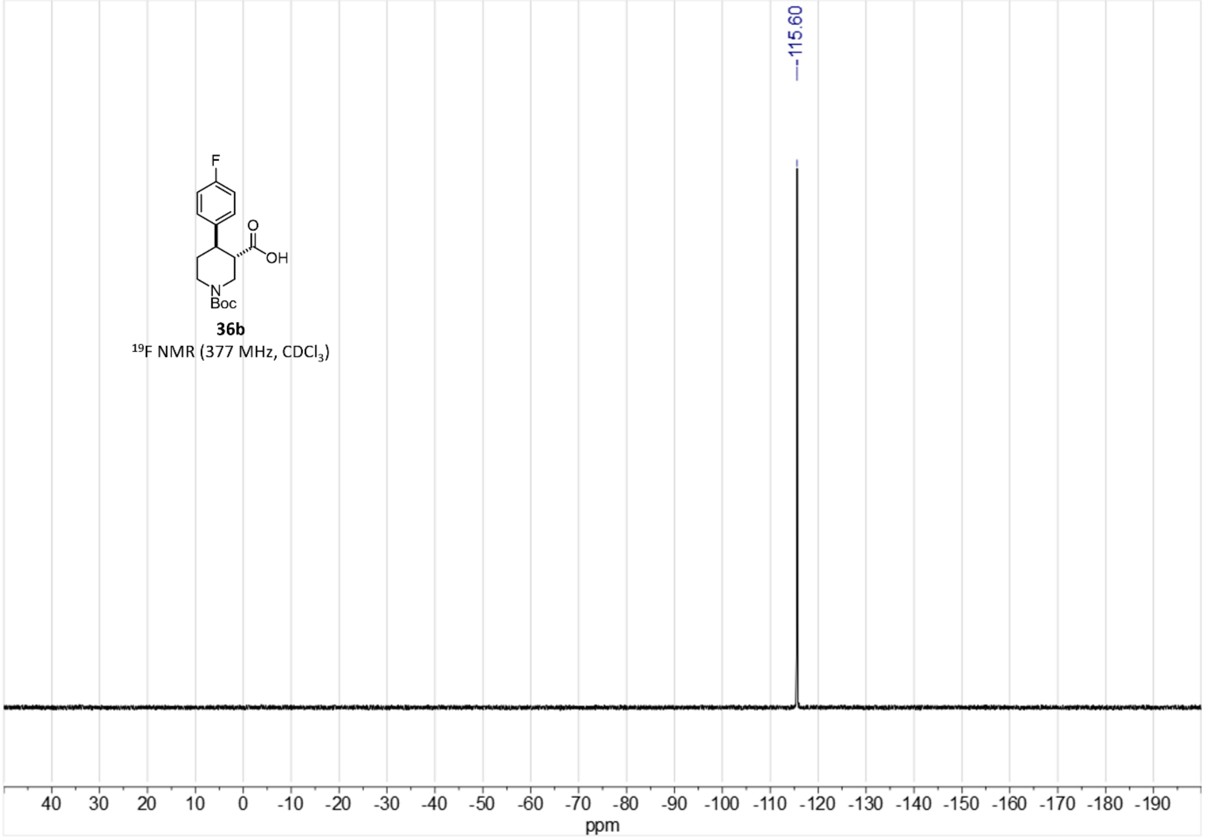

36b

^{19}F NMR (377 MHz, $CDCl_3$)

trans-(±)-tert-Butyl 3-carbamoyl-4-(4-fluorophenyl)piperidine-1-carboxylate (39b)

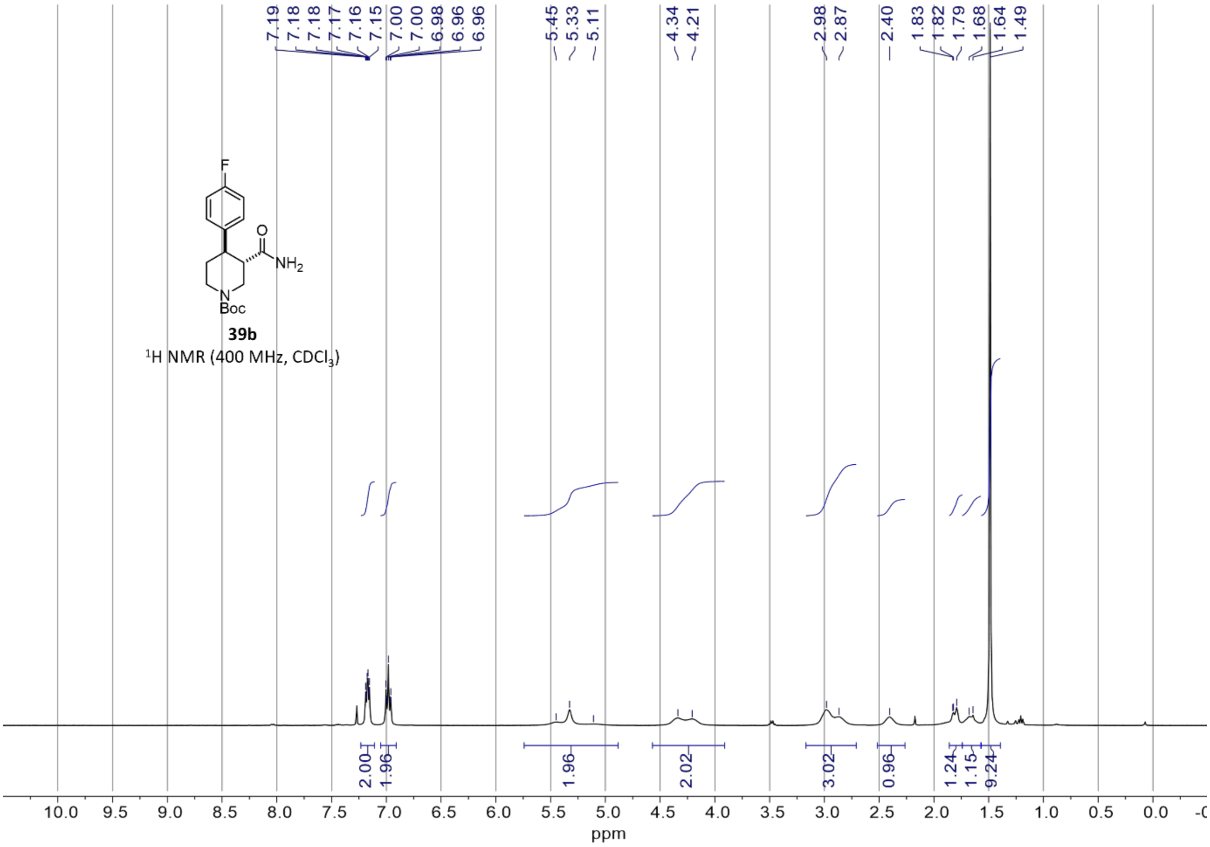

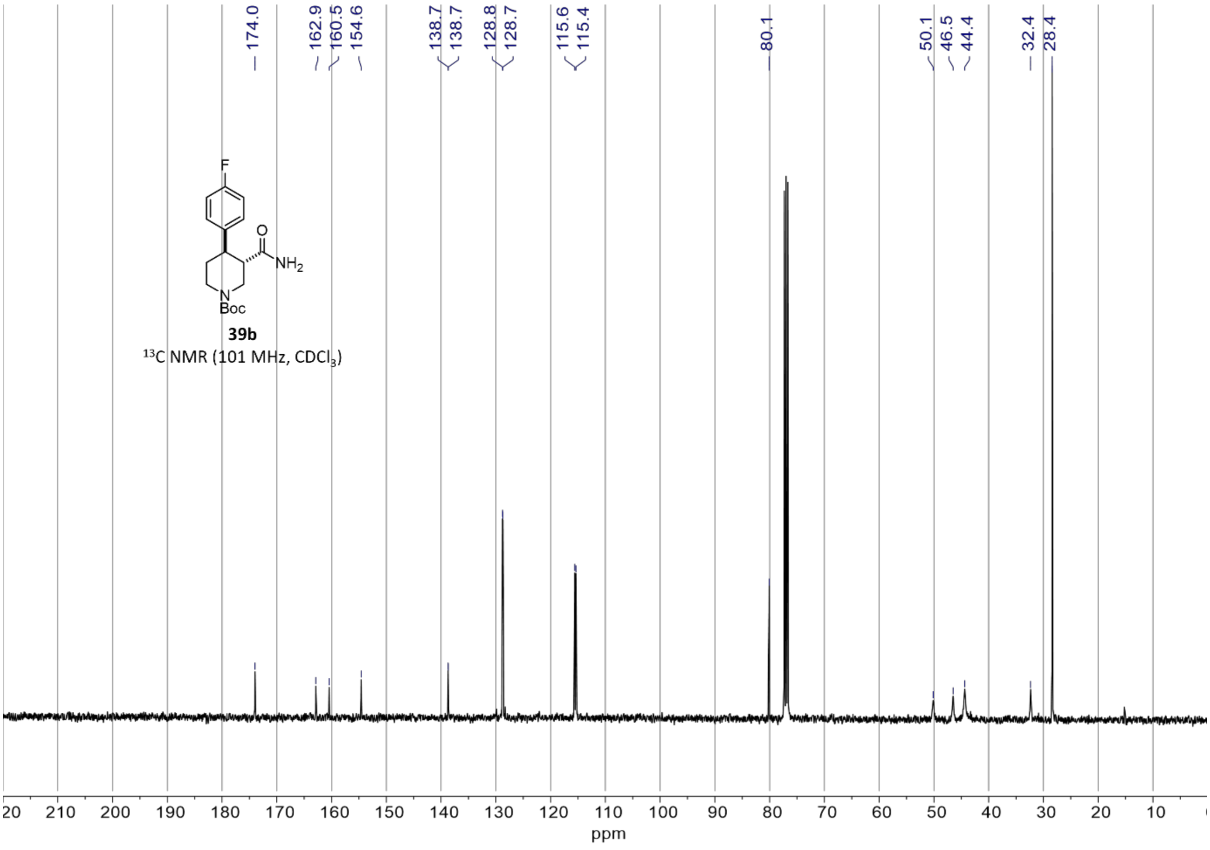

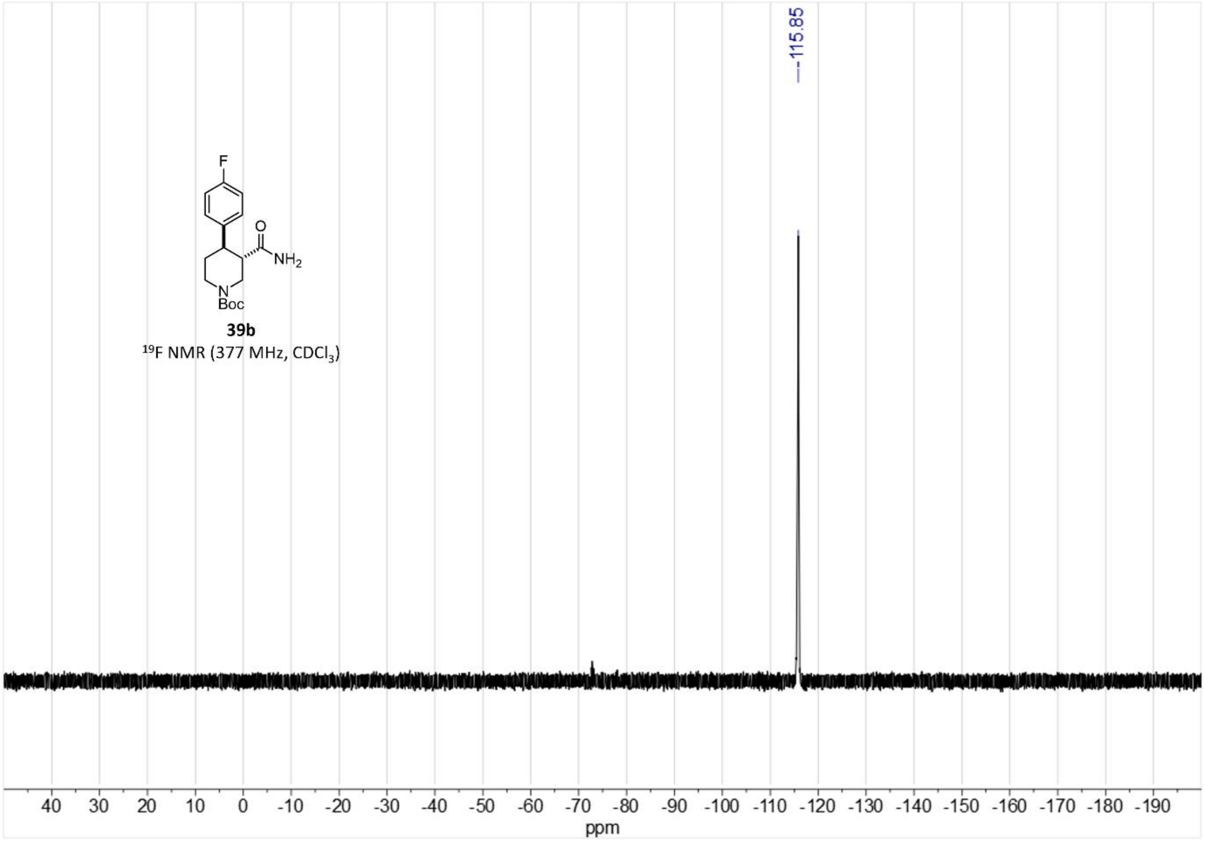

-115.85
F
O
NH2
N
Boc
39b
19F NMR (377 MHz, CDCl3)
40 30 20 10 0 -10 -20 -30 -40 -50 -60 -70 -80 -90 -100 -110 -120 -130 -140 -150 -160 -170 -180 -190
ppm

cis-(±)-tert-Butyl 3-(hydroxymethyl)-4-(4-methoxyphenyl)piperidine-1-carboxylate (37a)

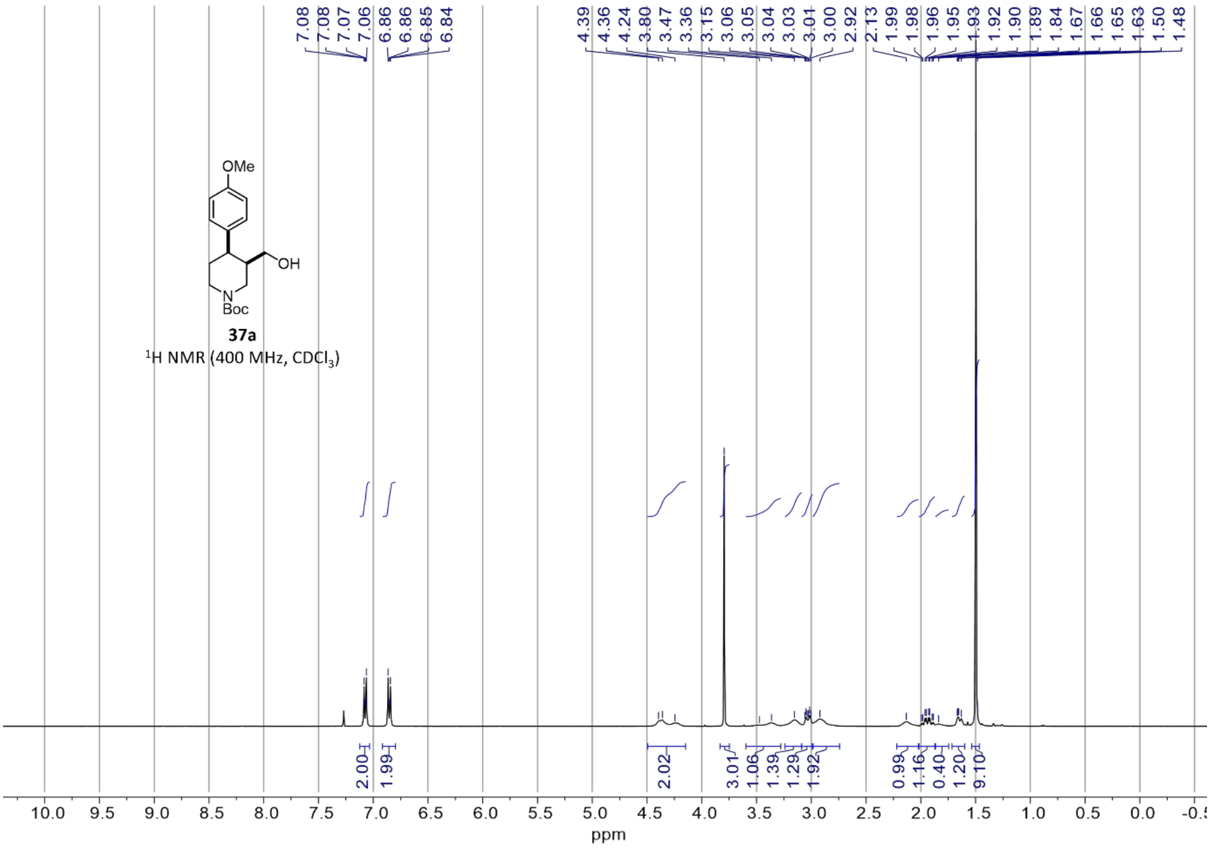

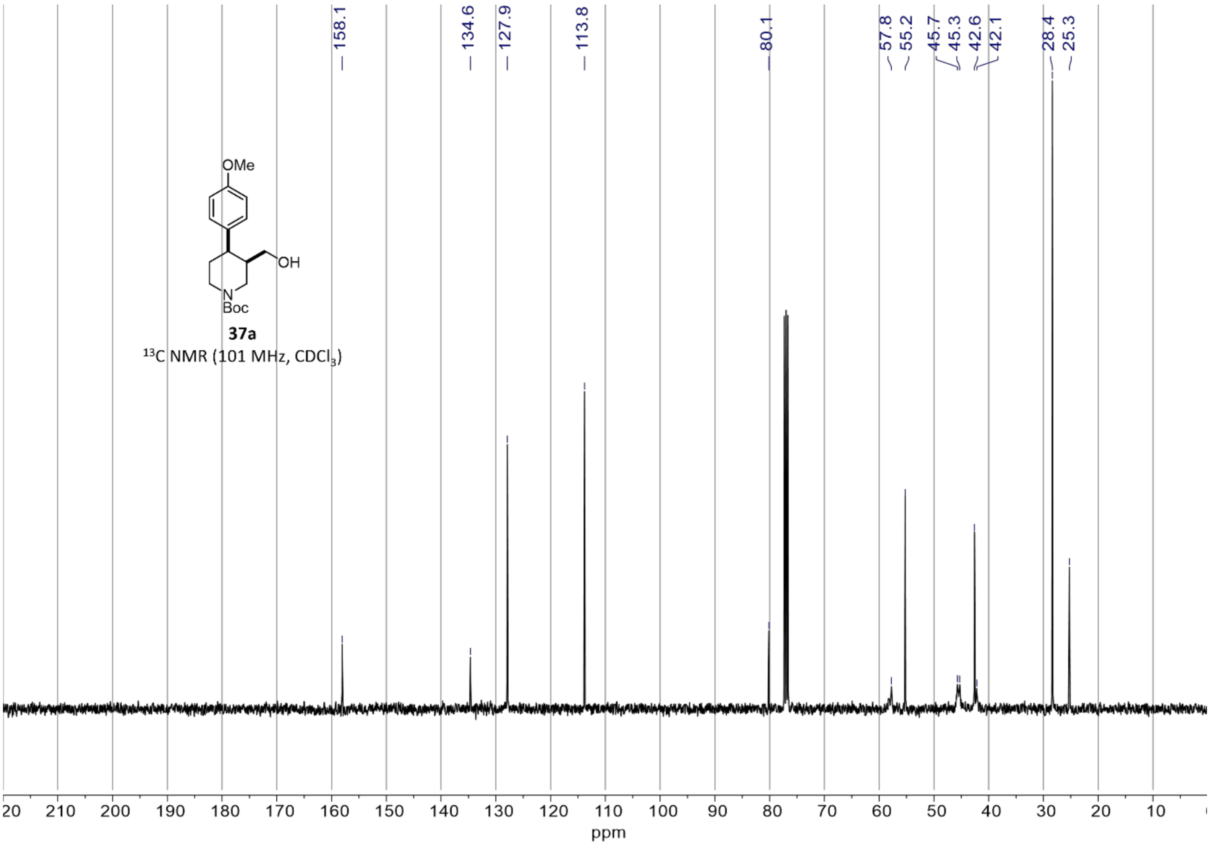

trans-(±)-_tert_-Butyl 3-(hydroxymethyl)-4-(4-methoxyphenyl)piperidine-1-carboxylate (38a)

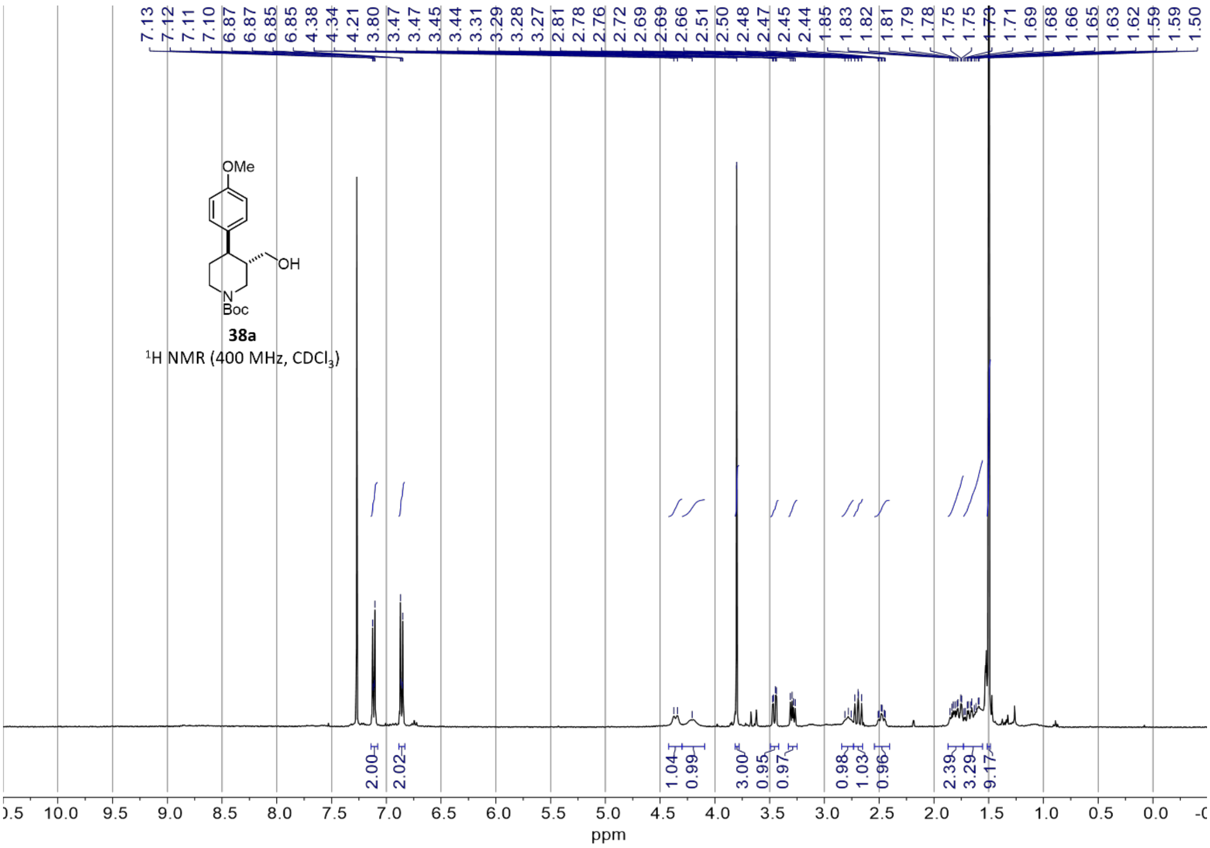

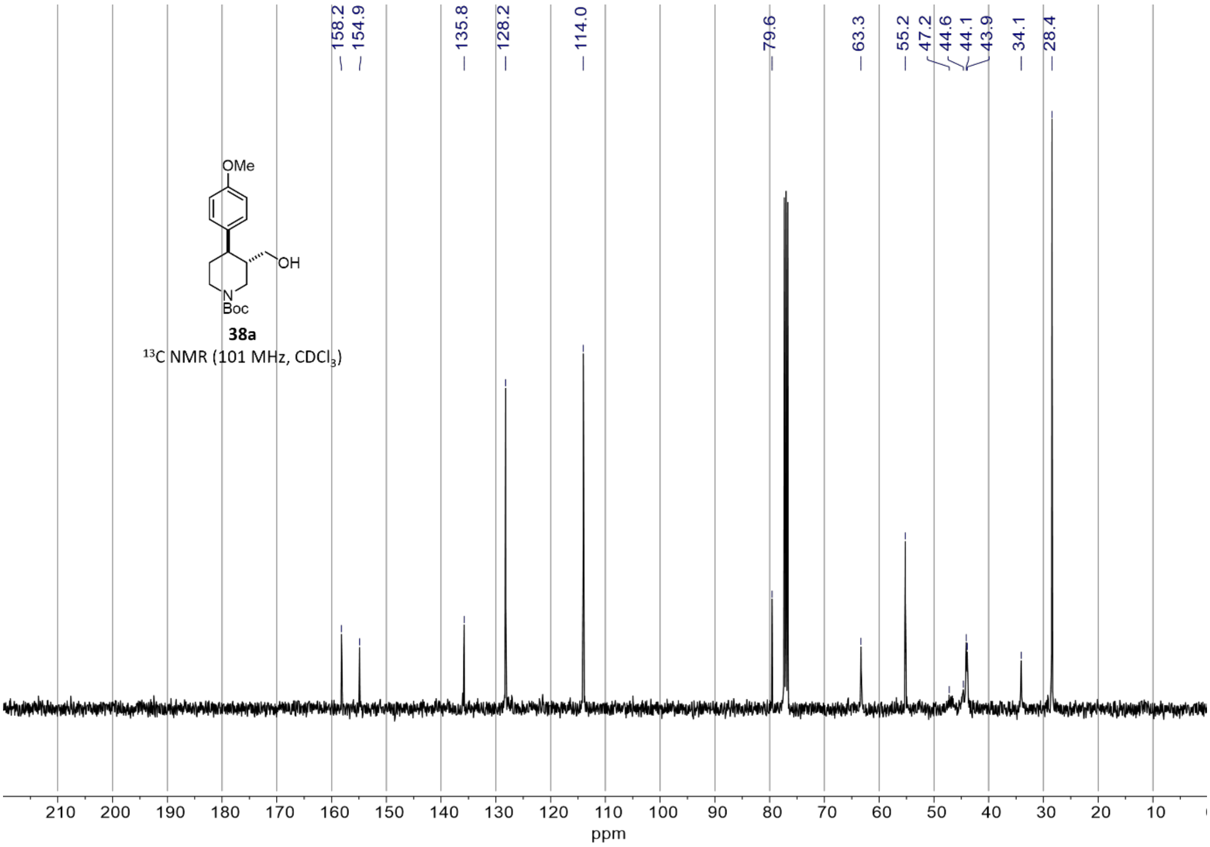

***trans-(±)-tert*-Butyl 3-cyano-4-(4-fluorophenyl)piperidine-1-carboxylate (S2)**

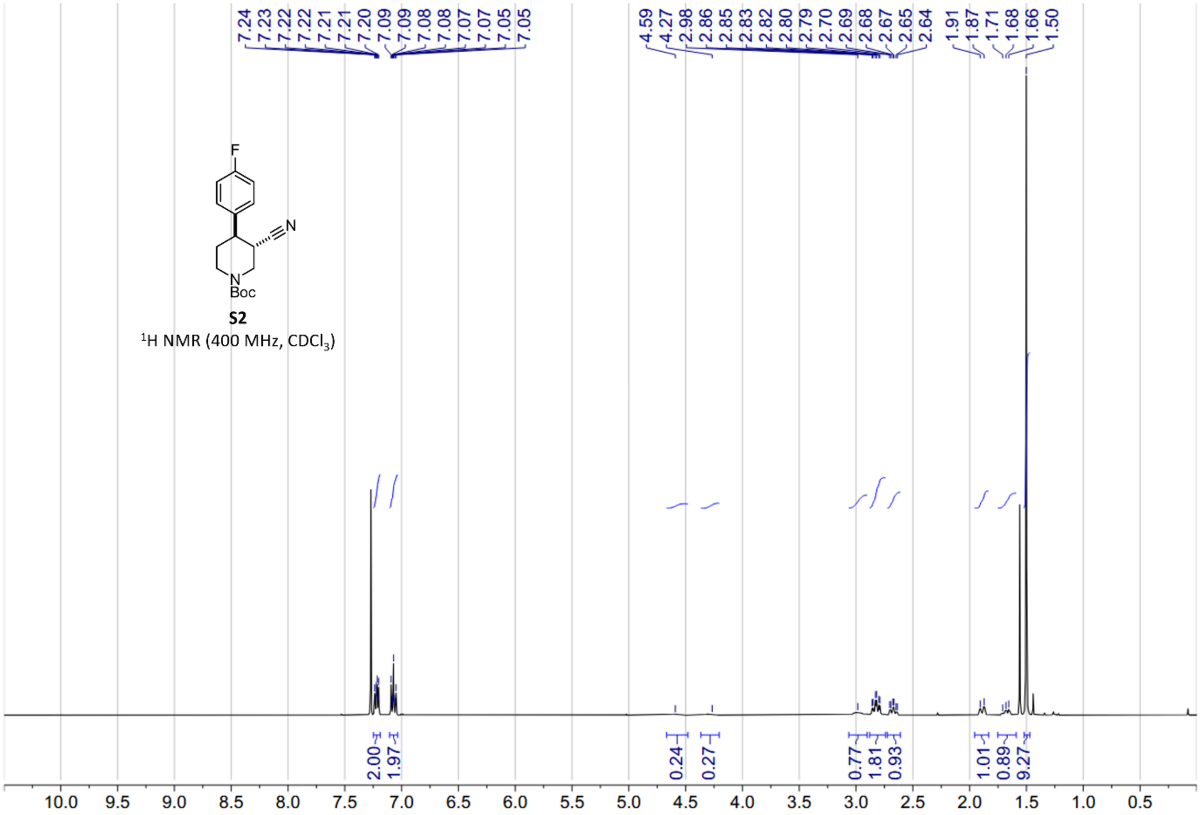

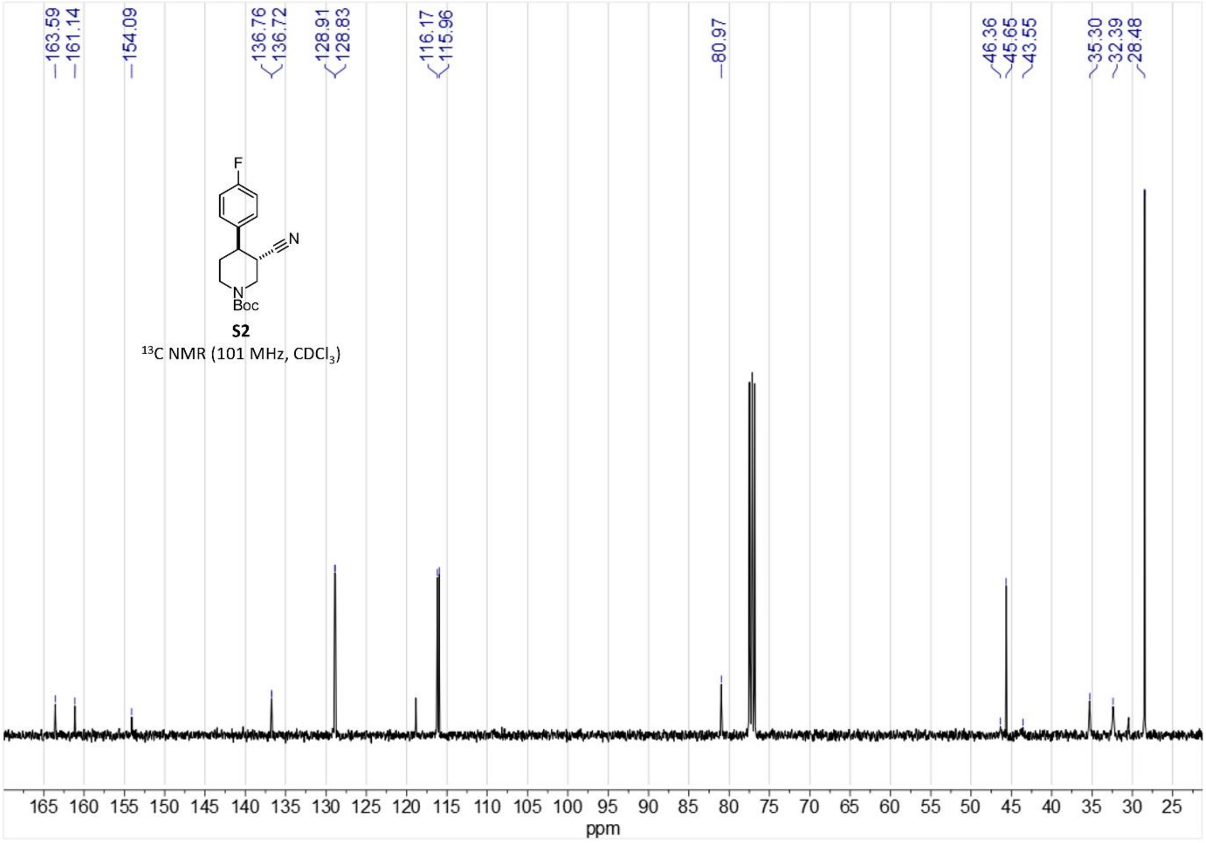

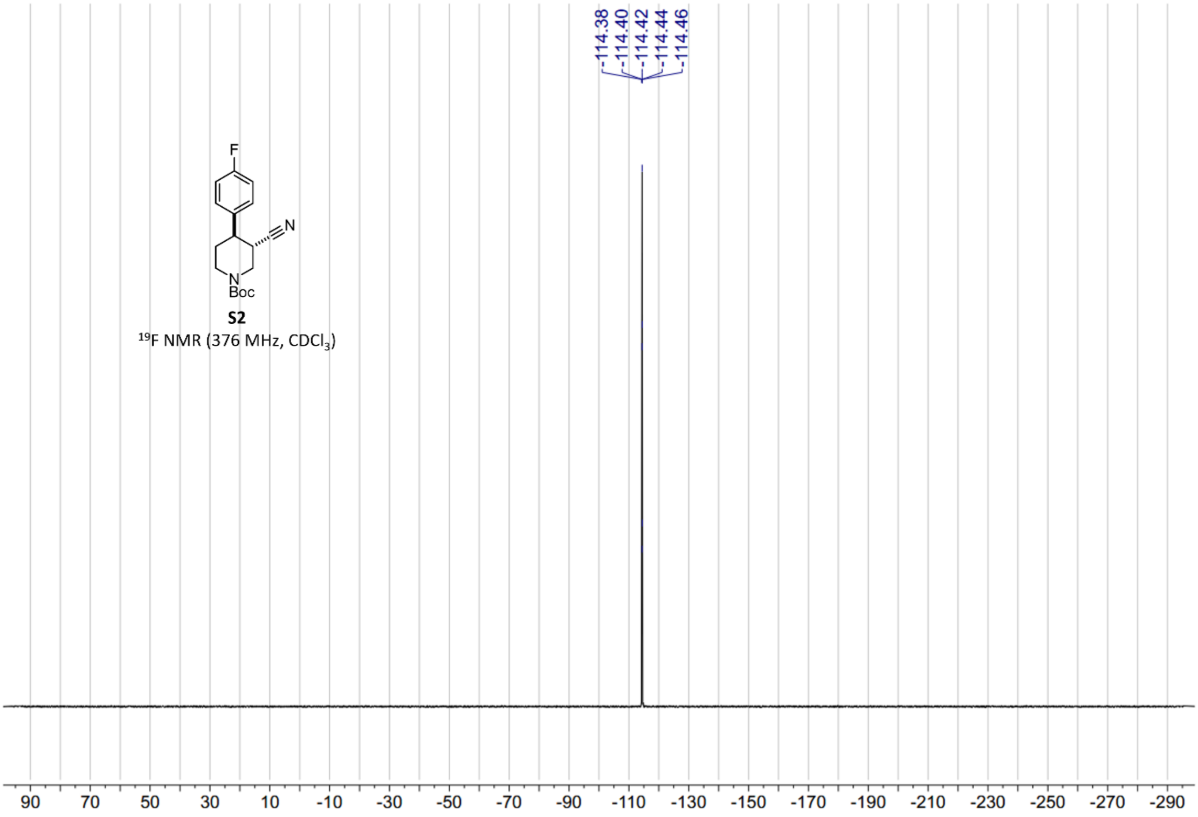
-114.38
-114.40
-114.42
-114.44
-114.46
F
N
N
Boc
S2
^{19}F NMR (376 MHz, $CDCl_3$)
90 70 50 30 10 -10 -30 -50 -70 -90 -110 -130 -150 -170 -190 -210 -230 -250 -270 -290

cis-(±)-3-Carboxy-4-(4-methoxyphenyl)piperidin-1-ium chloride (FRAG20)

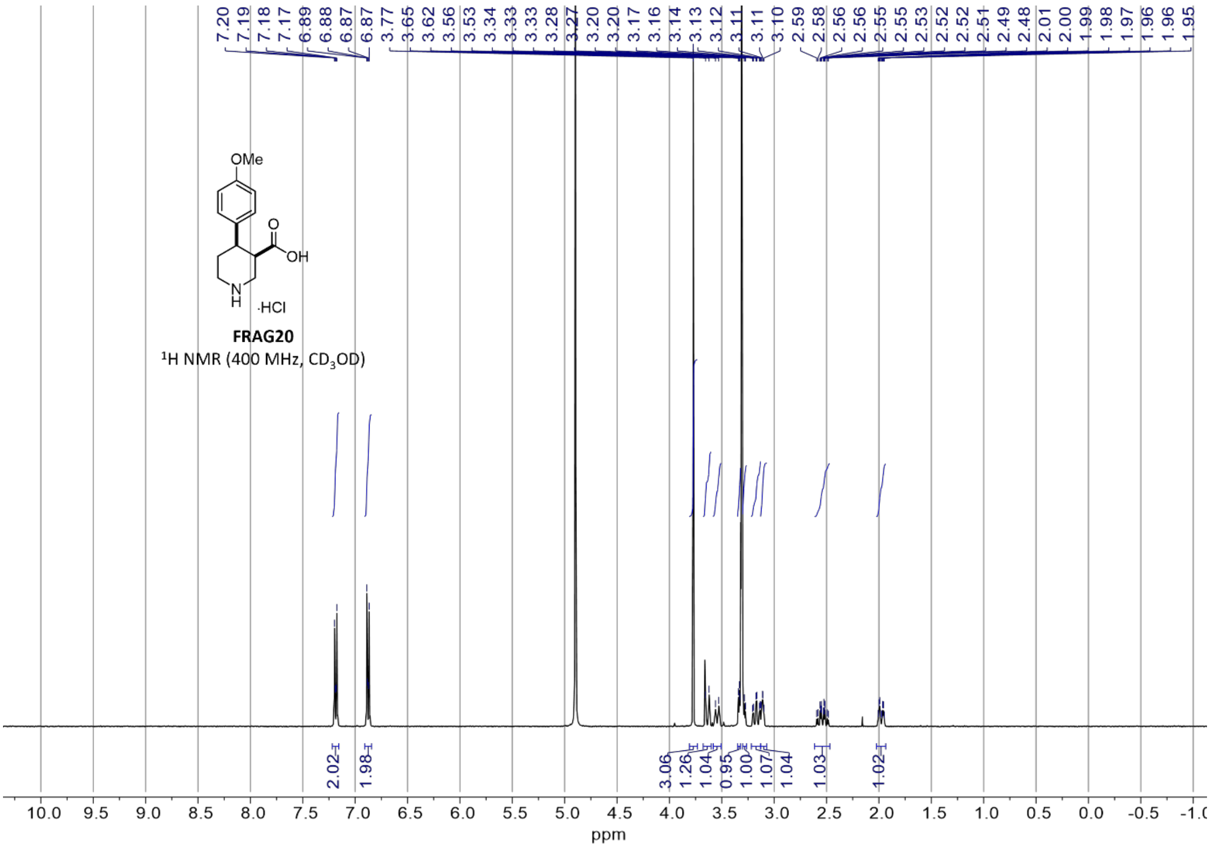

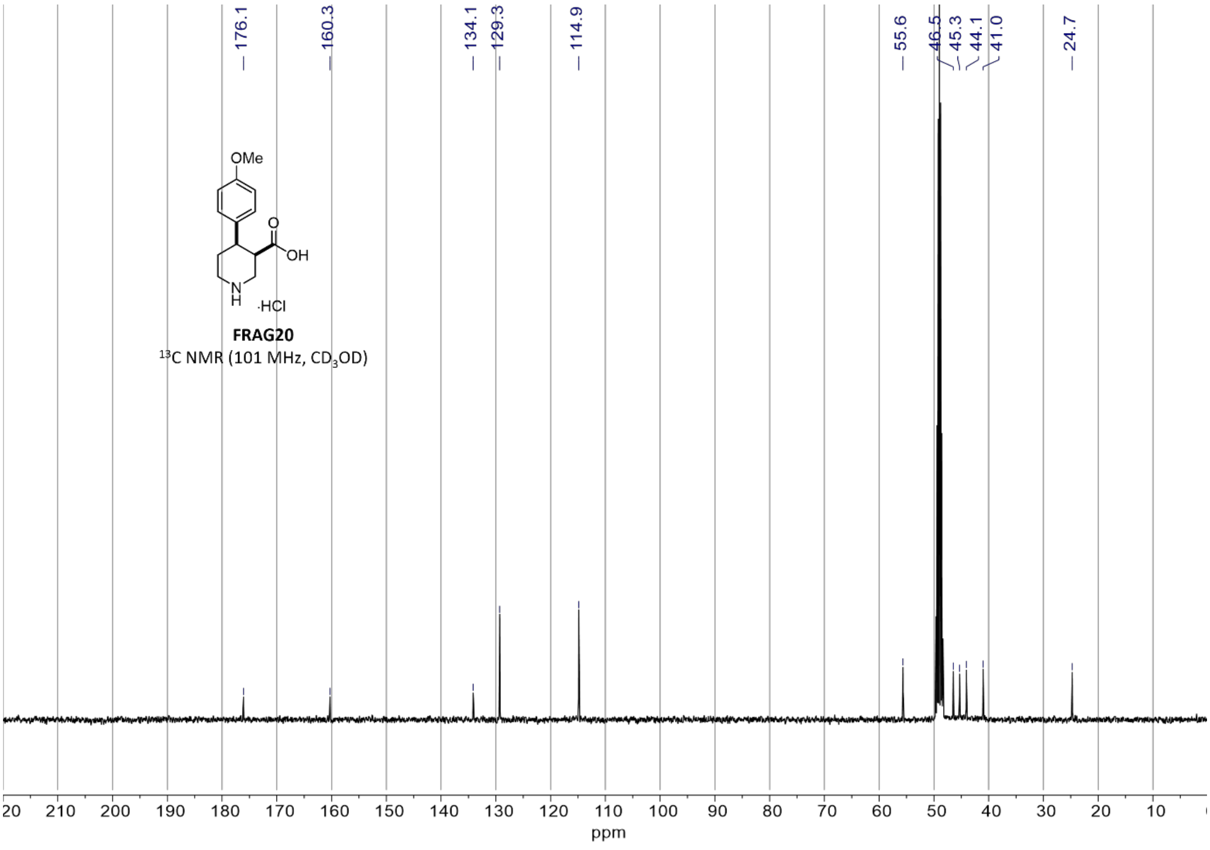

trans-(±)-3-Carboxy-4-(4-methoxyphenyl)piperidin-1-ium chloride (FRAG22)

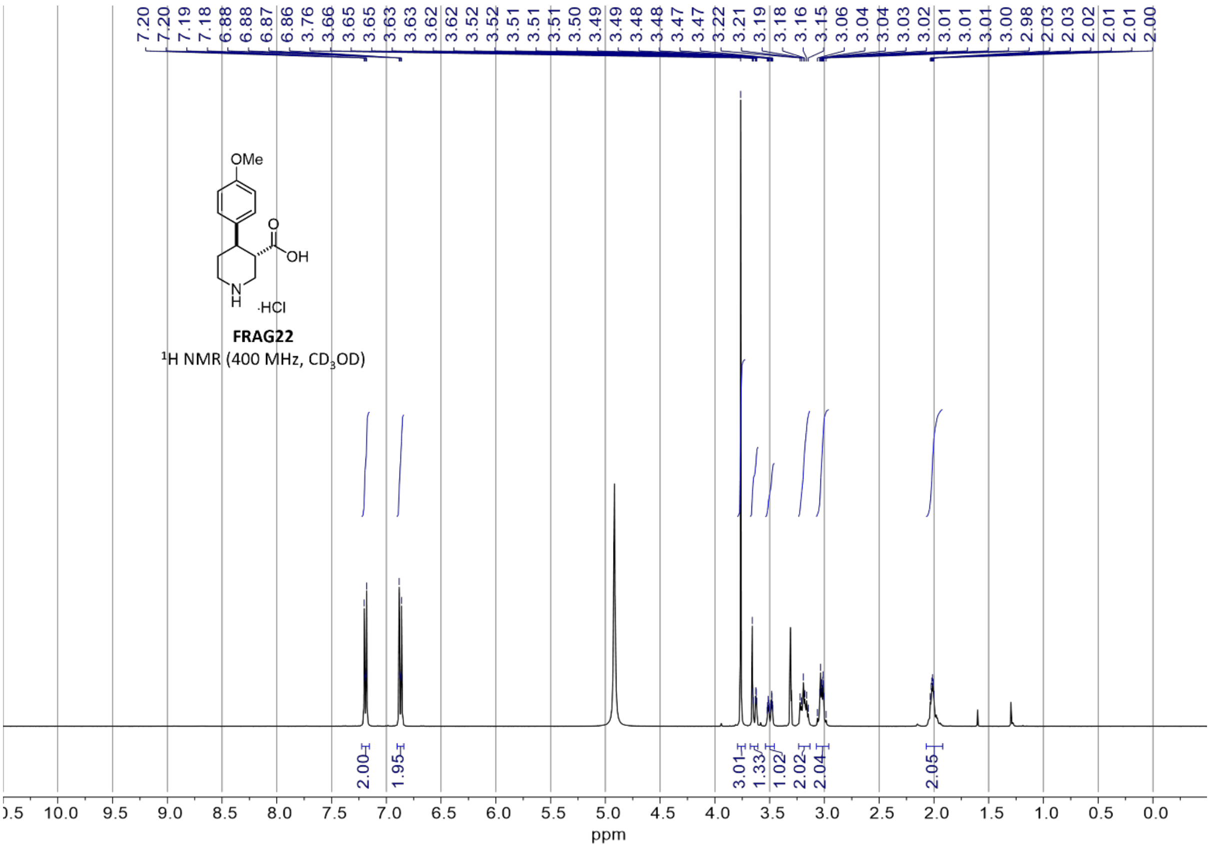

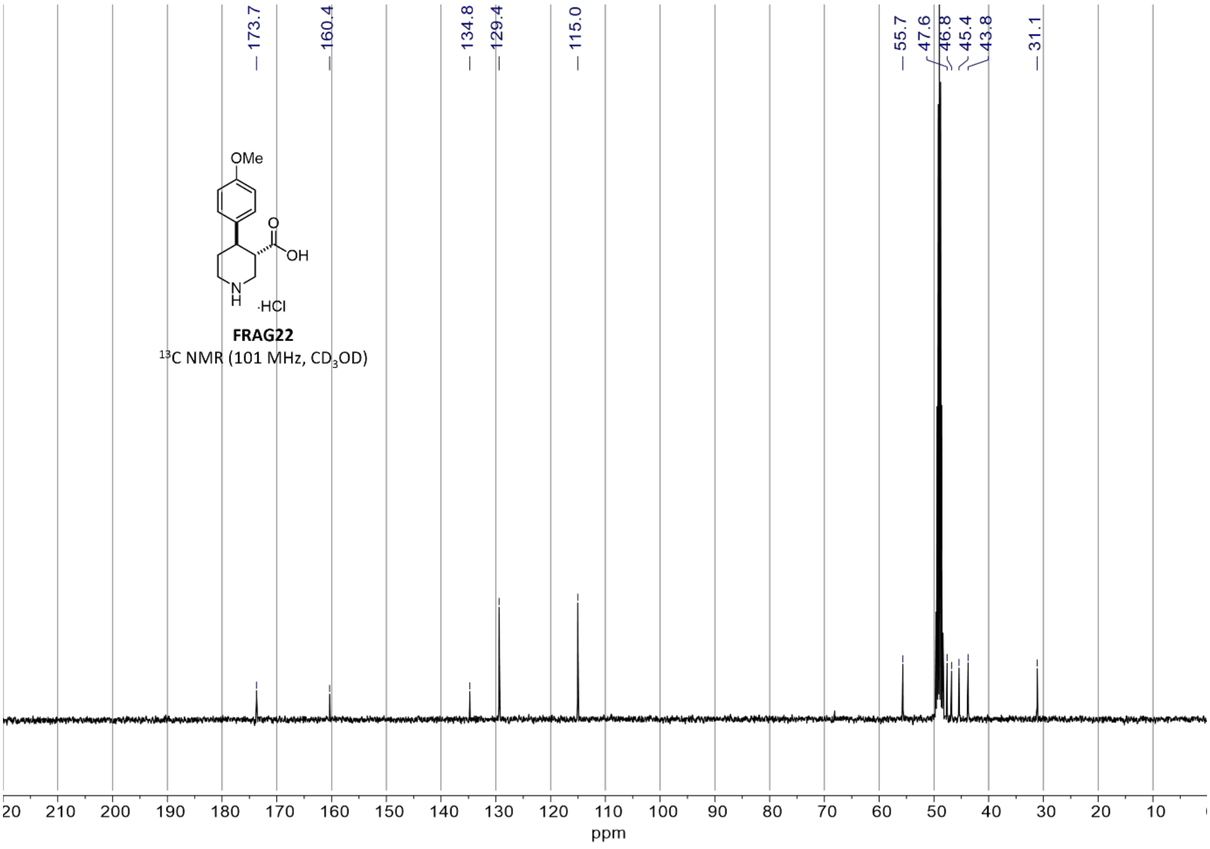

trans-(±)-3-Carboxy-4-(4-fluorophenyl)piperidin-1-ium chloride (FRAG23)

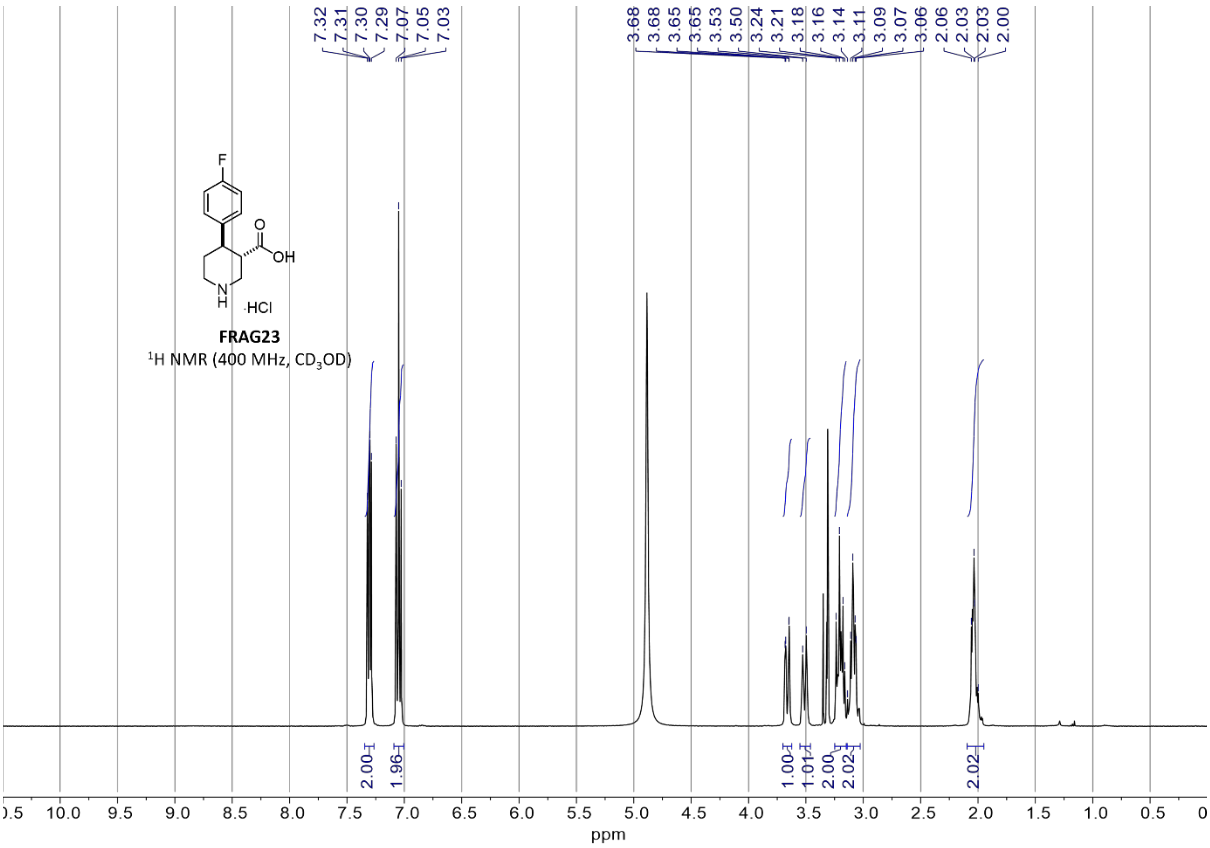

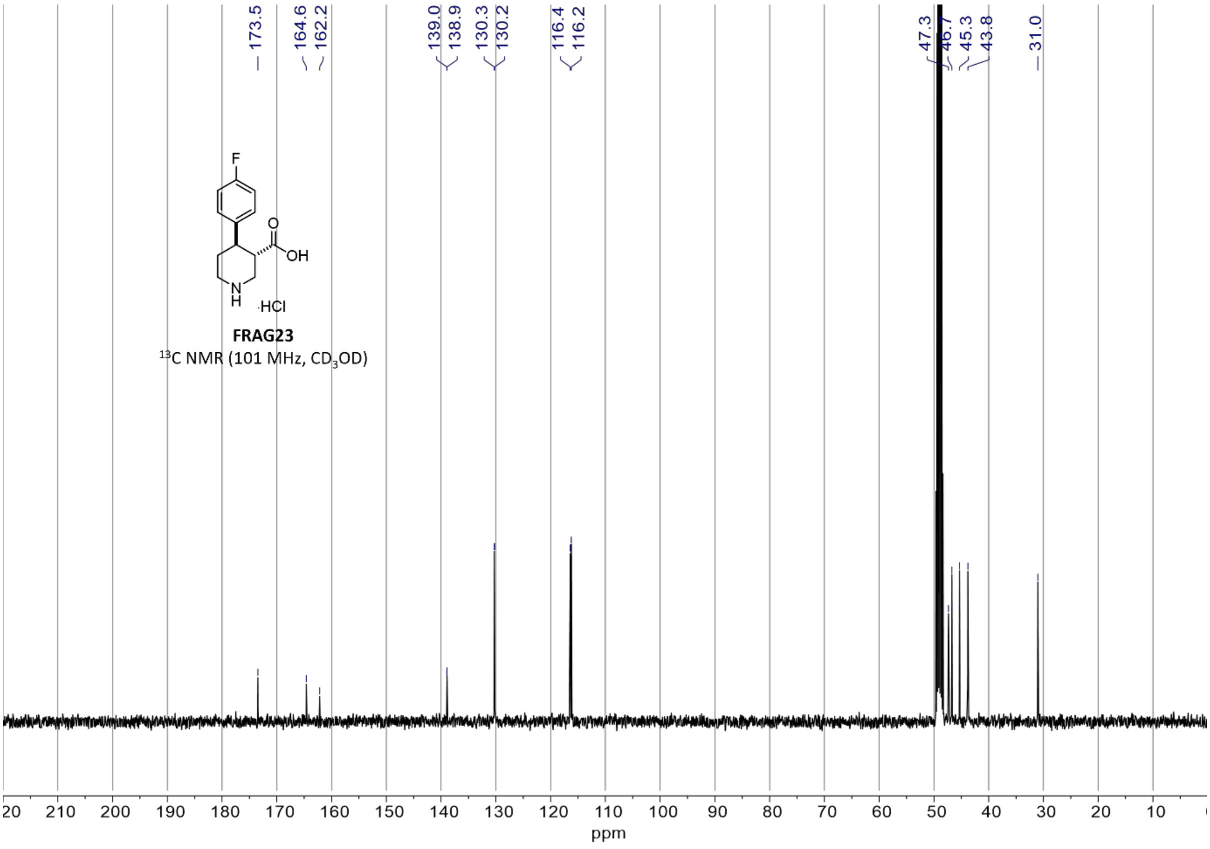

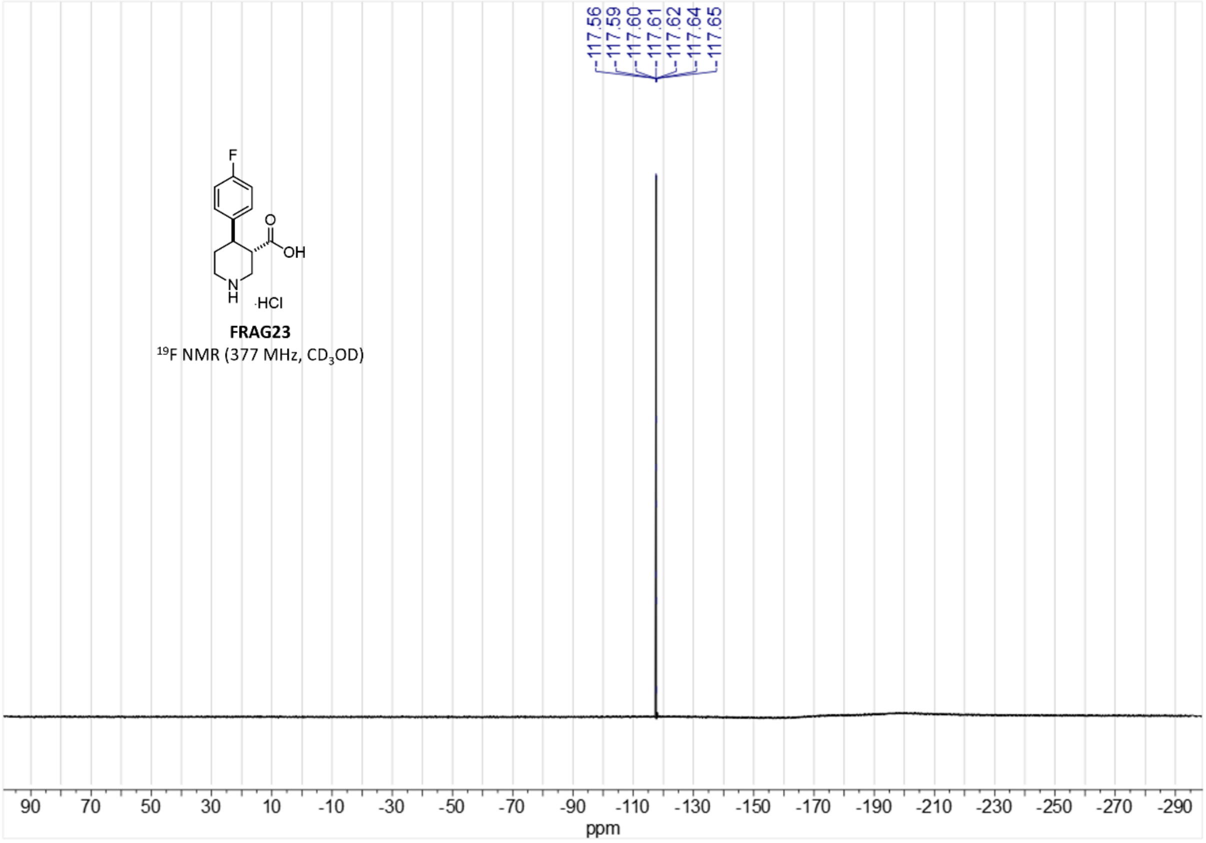
-117.56
-117.59
-117.60
-117.61
-117.62
-117.64
-117.65
F
O
OH
N
H
·HCl
FRAG23
19F NMR (377 MHz, CD3OD)
90 70 50 30 10 -10 -30 -50 -70 -90 -110 -130 -150 -170 -190 -210 -230 -250 -270 -290
ppm

trans-(±)-3-Carbamoyl-4-(4-fluorophenyl)piperidin-1-ium chloride (FRAG27)

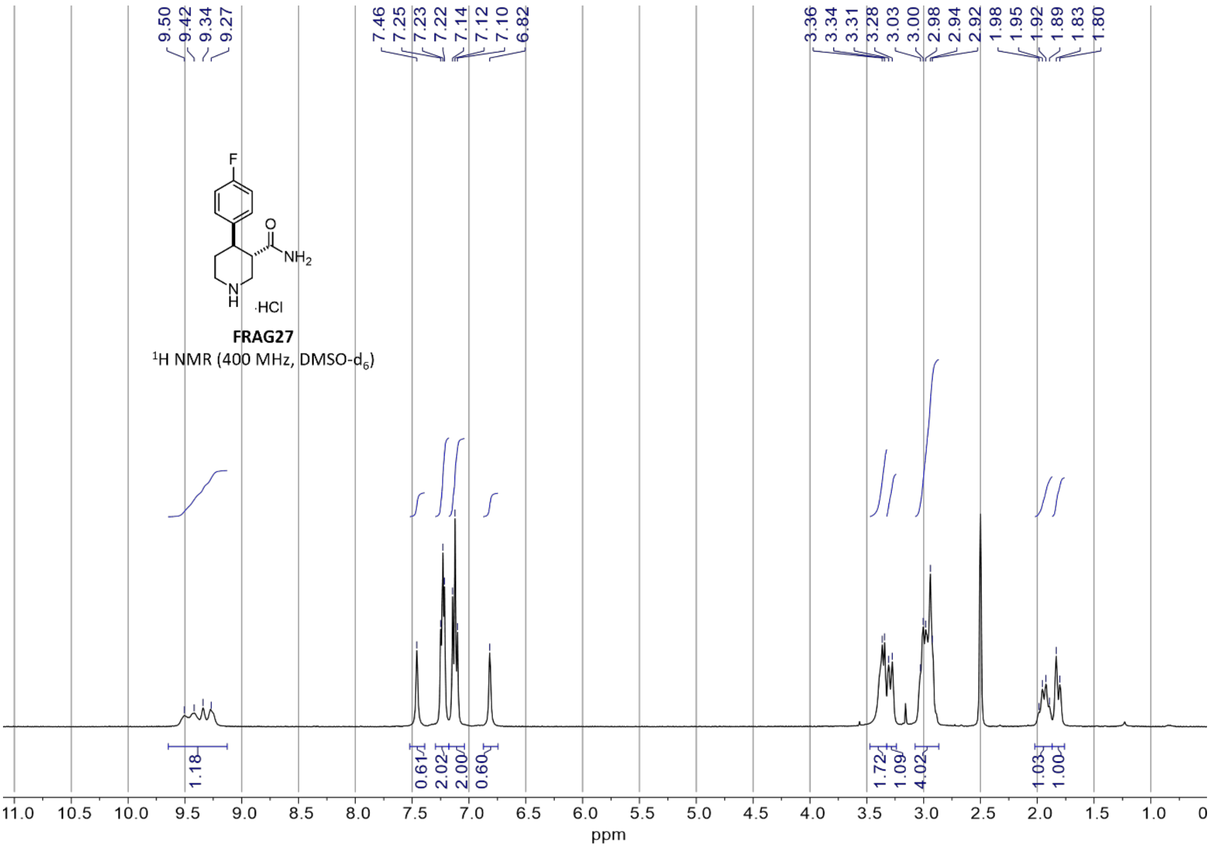

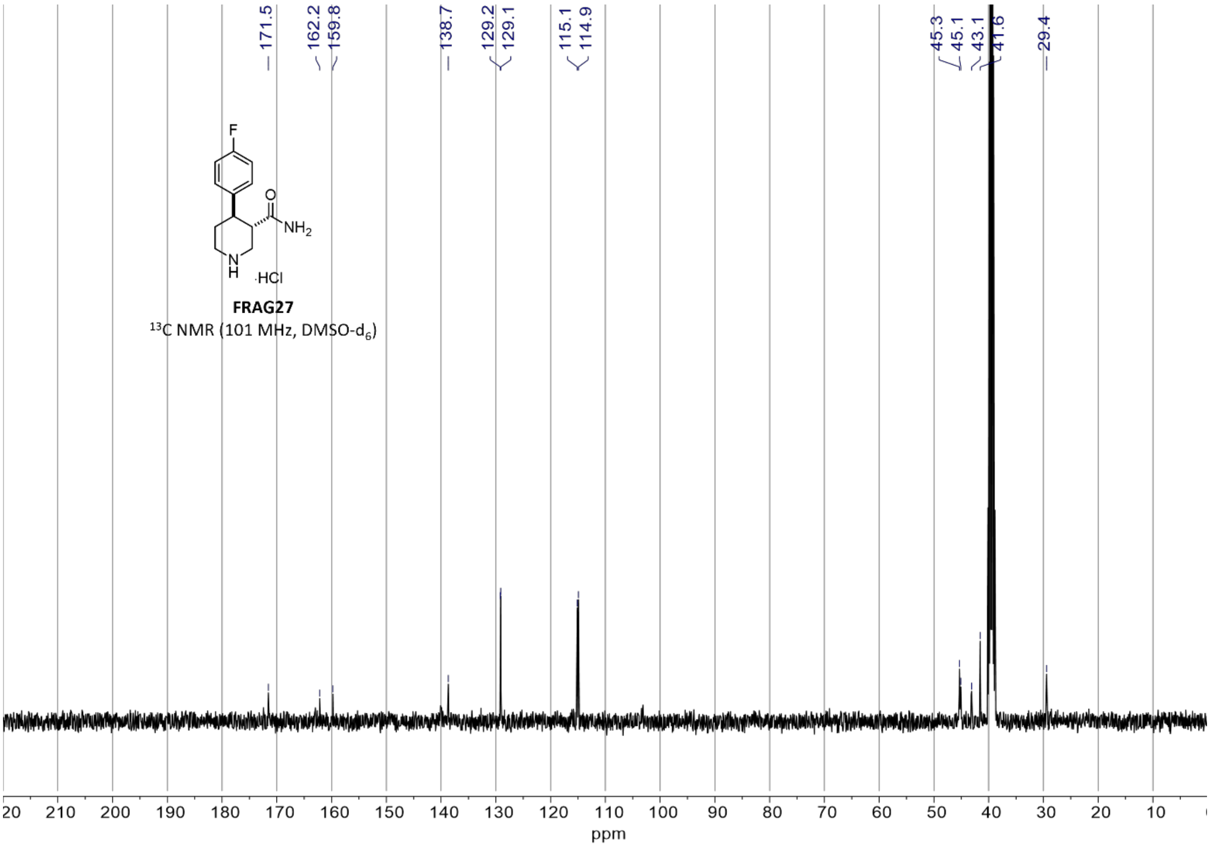

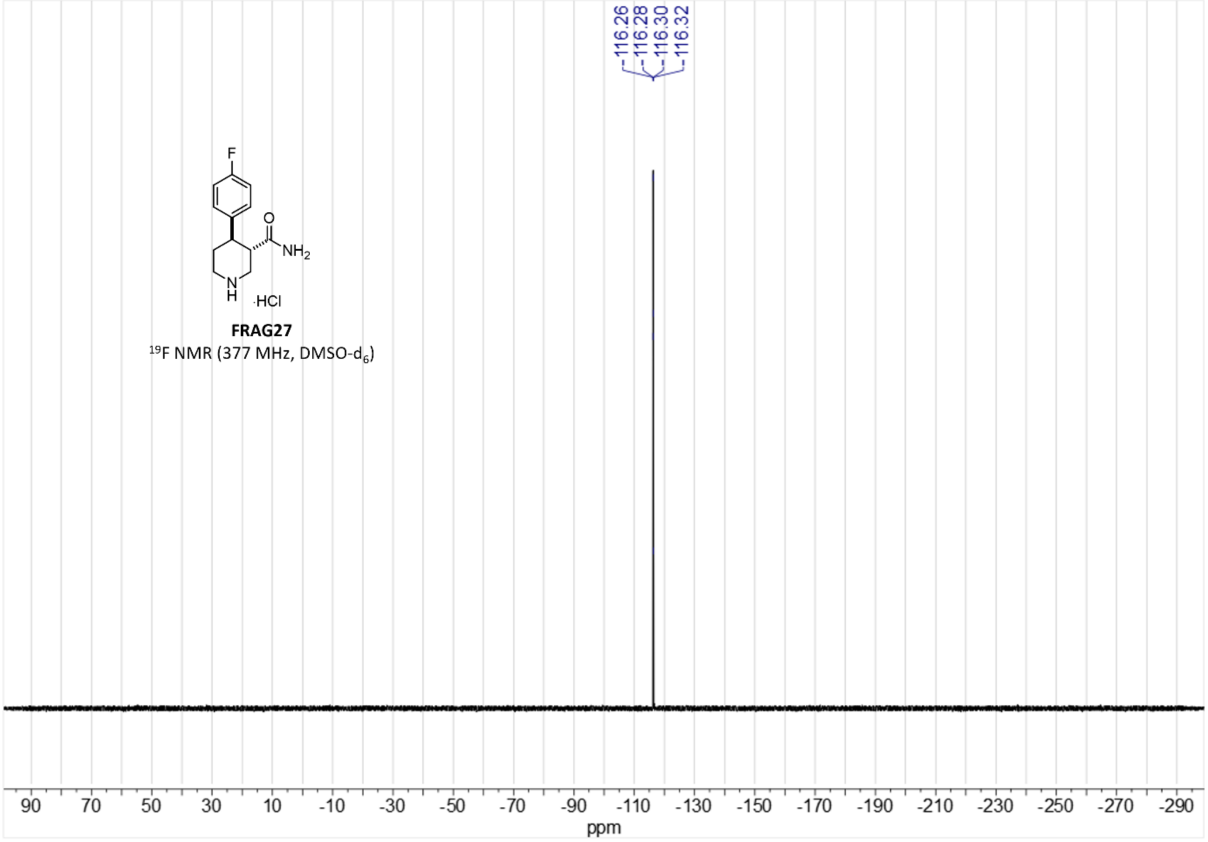

FRAG27

^{19}F NMR (377 MHz, DMSO-d_6)

***cis*-(±)-3-(Hydroxymethyl)-4-(4-methoxyphenyl)piperidin-1-ium chloride (FRAG28)**

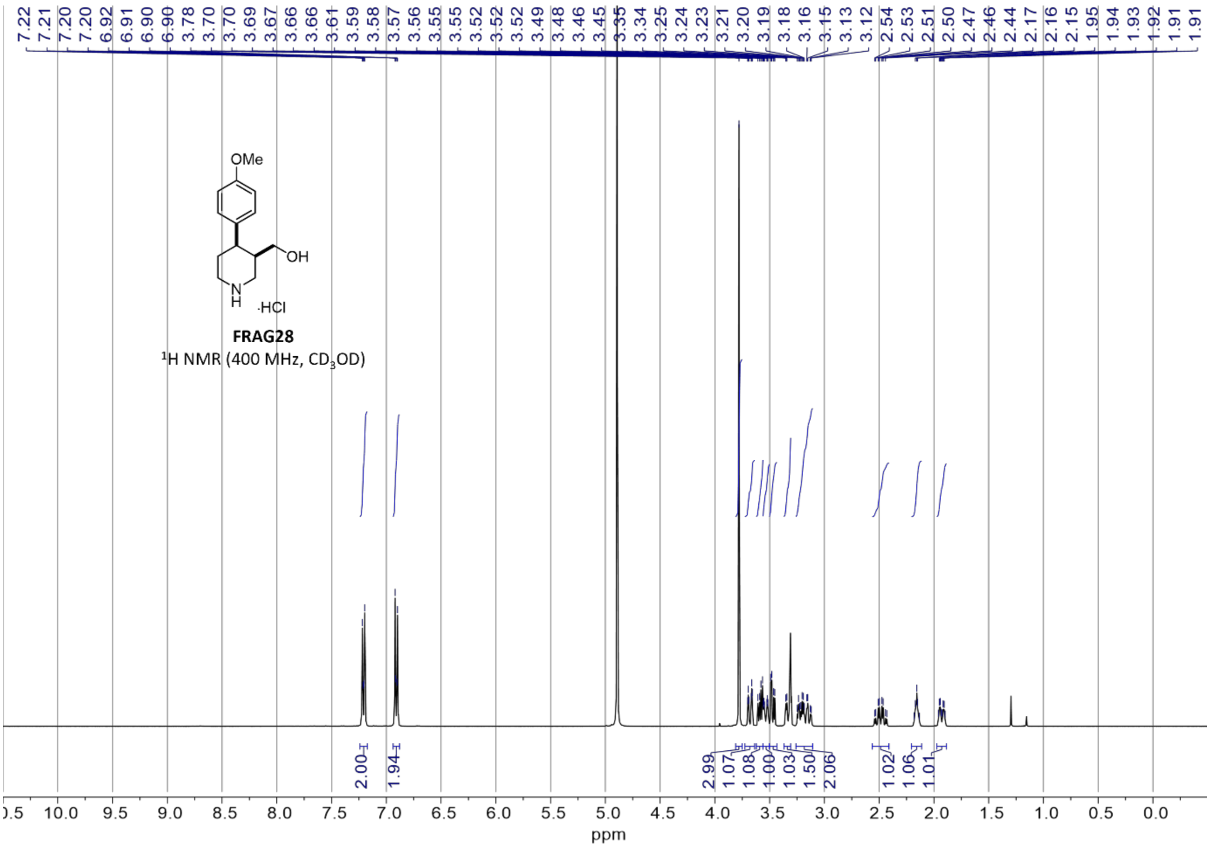

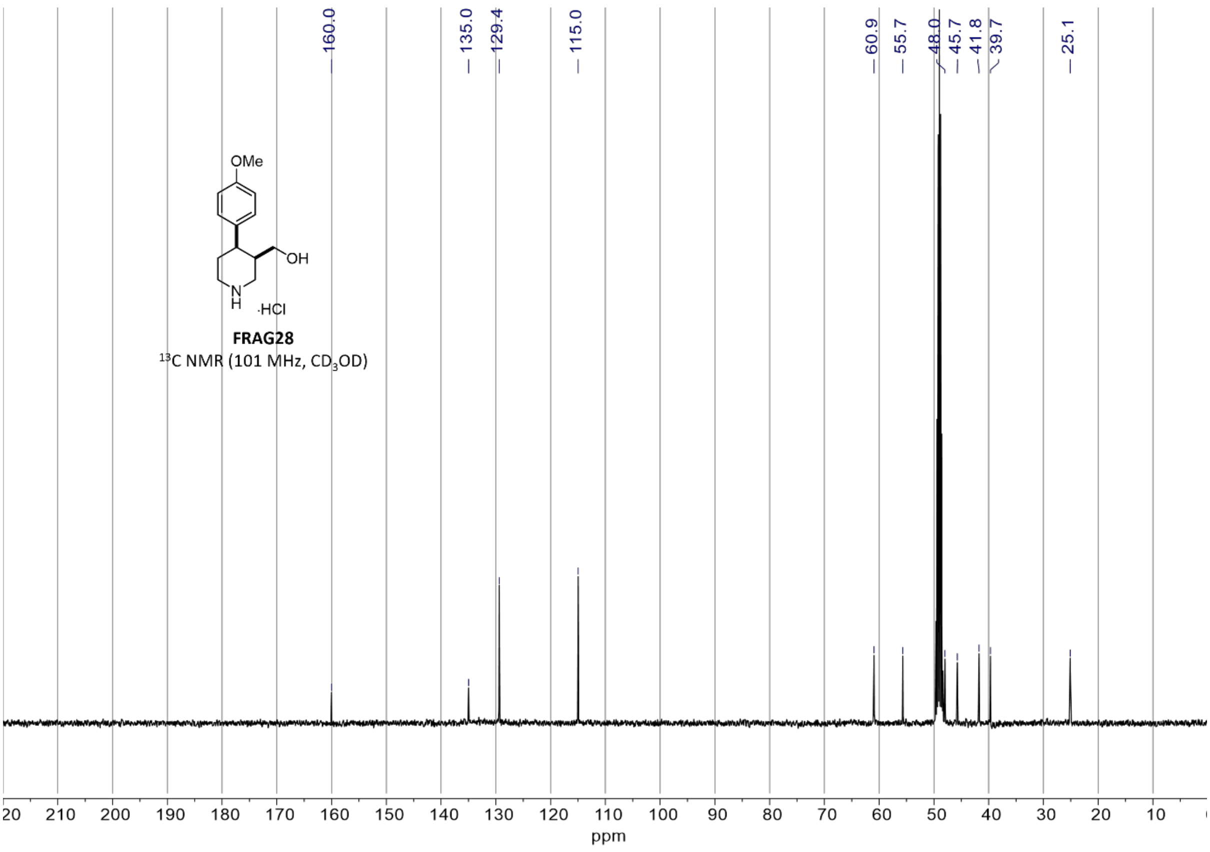

***trans*-(±)-3-(Hydroxymethyl)-4-(4-methoxyphenyl)piperidin-1-ium chloride (FRAG30)**

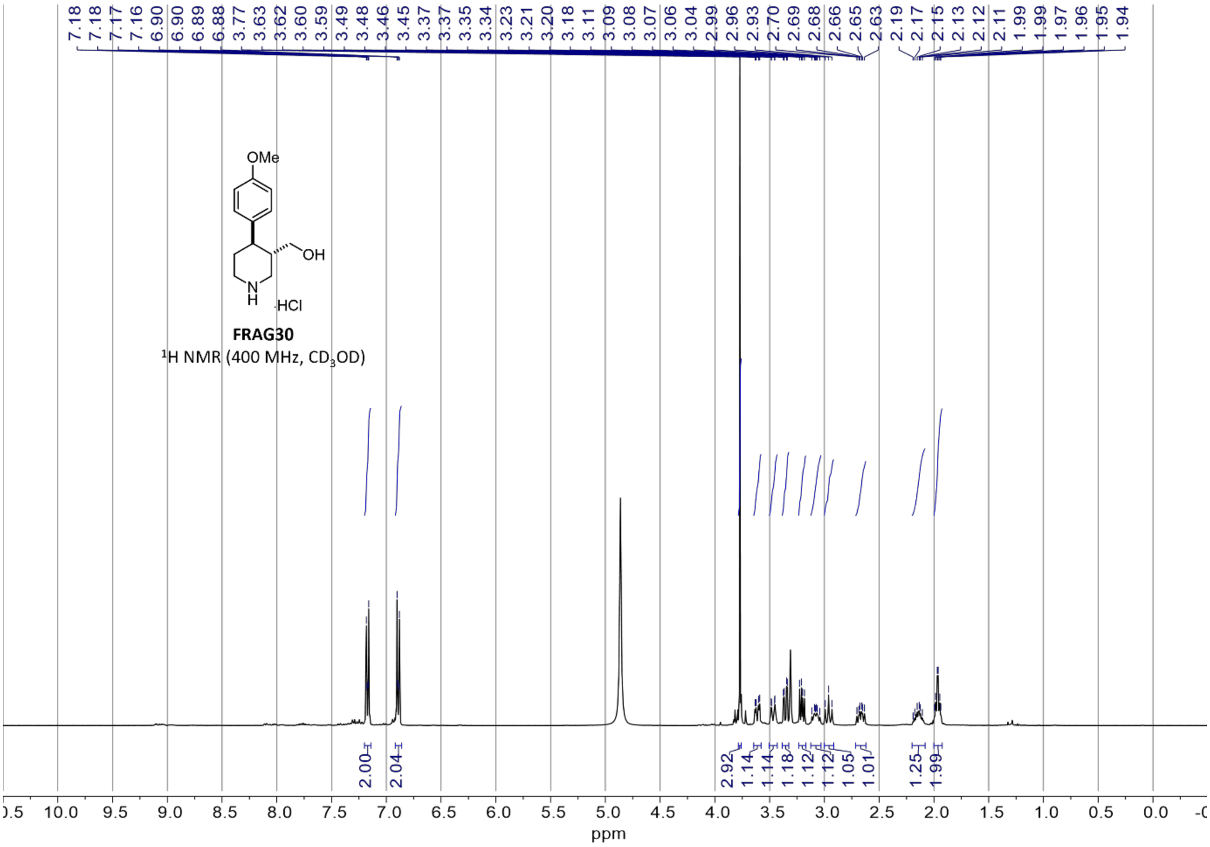

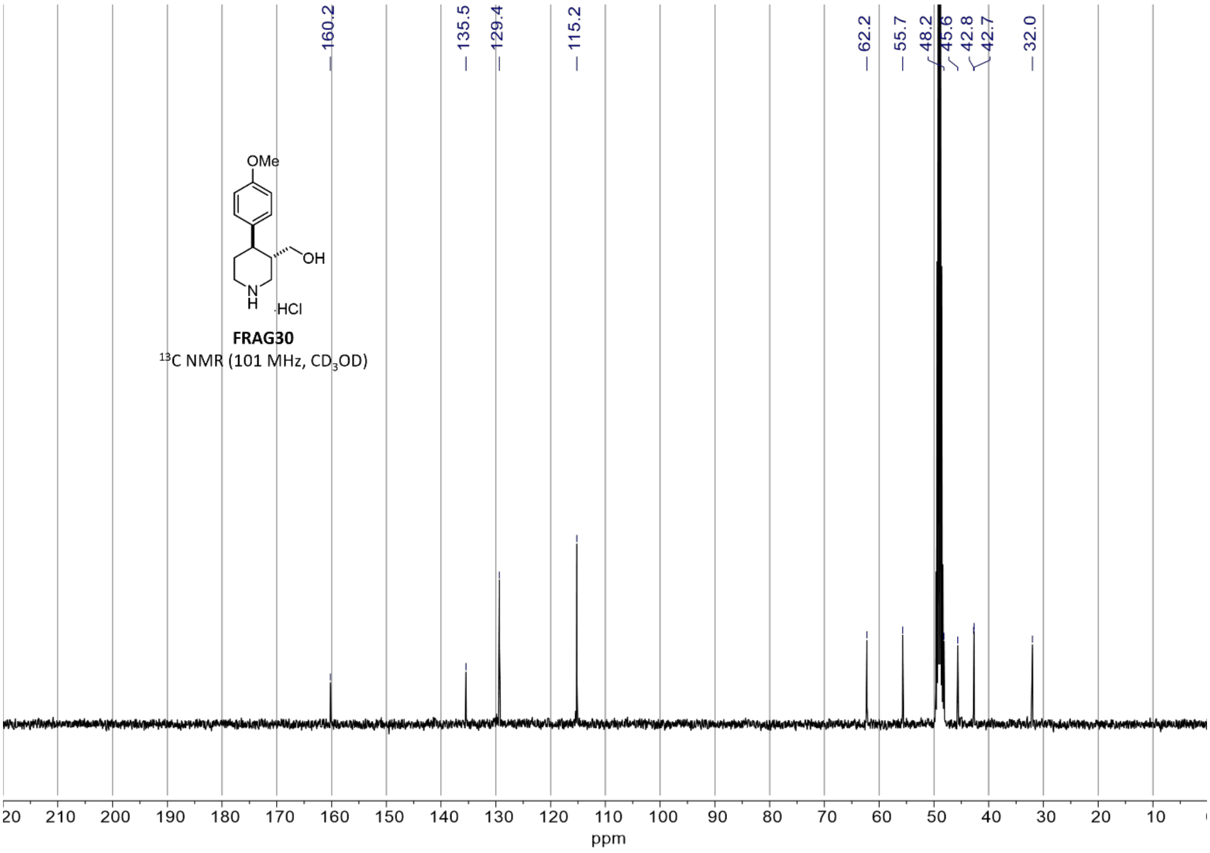

tert-Butyl _cis_-(±)-6,16-dioxo-3,3a,7,7a,16,16a-hexahydro-6_H_-pyrido[2,3-a]pyrrolo[3',4':3,4] pyrrolo[2,1-l]phenoxazine-2(1_H_)-carboxylate (31)

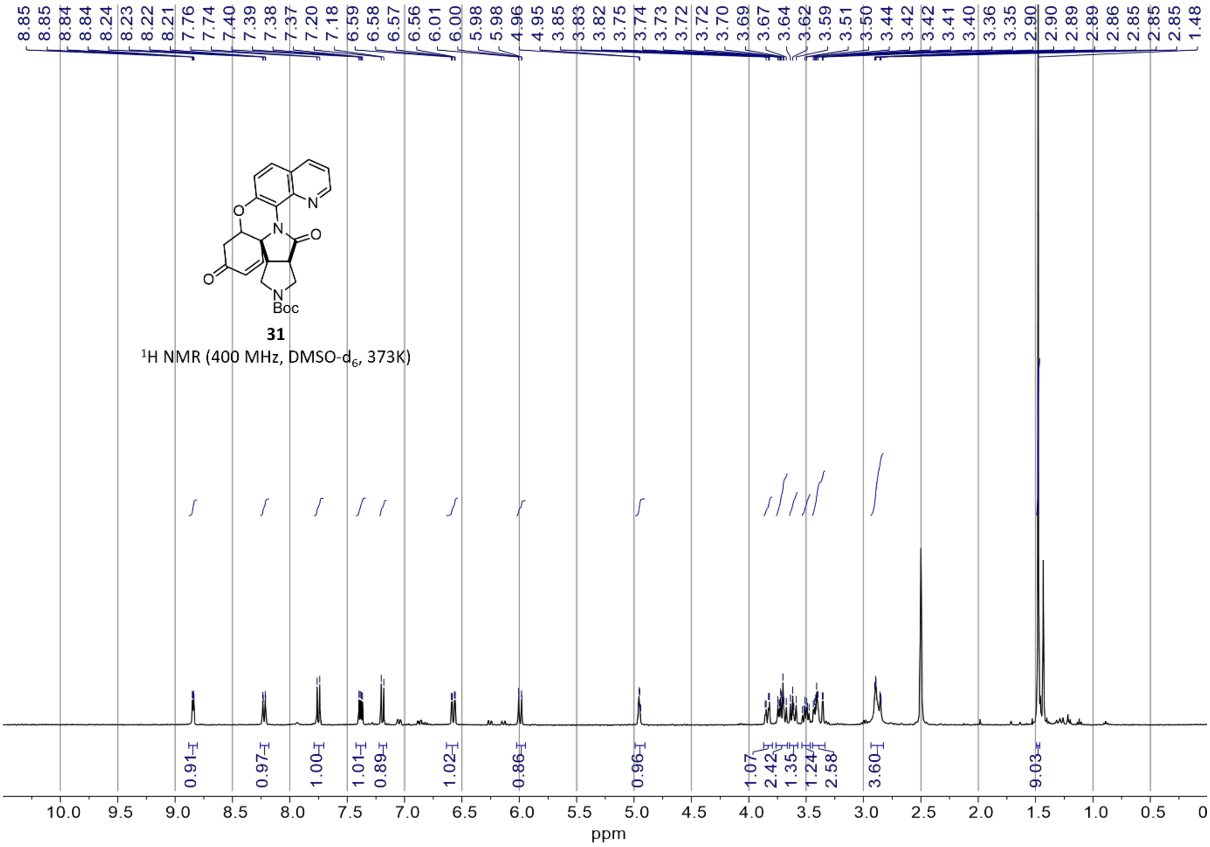

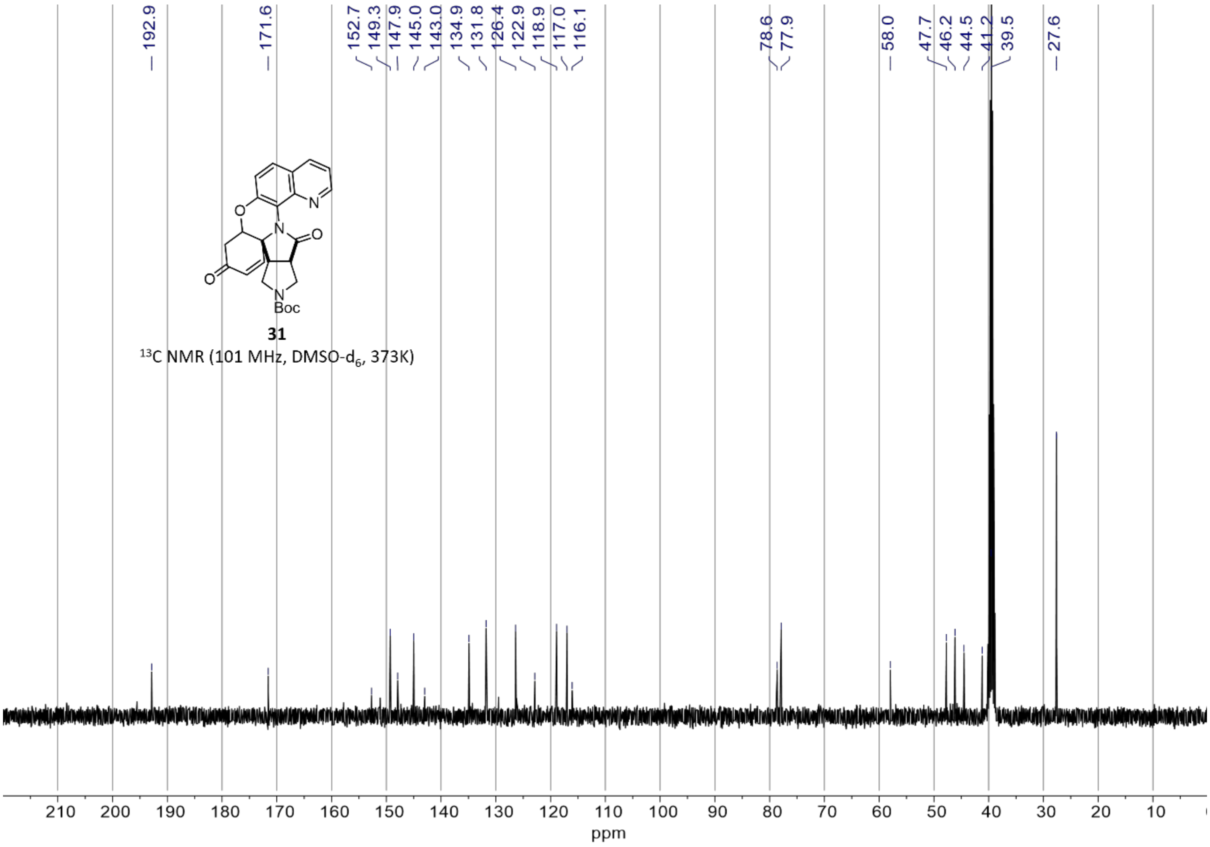

***tert*-Butyl *cis*-(±)-3',4-dioxo-2',3',3a',6',7',7a'-hexahydrospiro[cyclohexane-1,1'-pyrrolo[3,4-c]pyridine]-2,5-diene-5'(4'H)-carboxylate (40)**

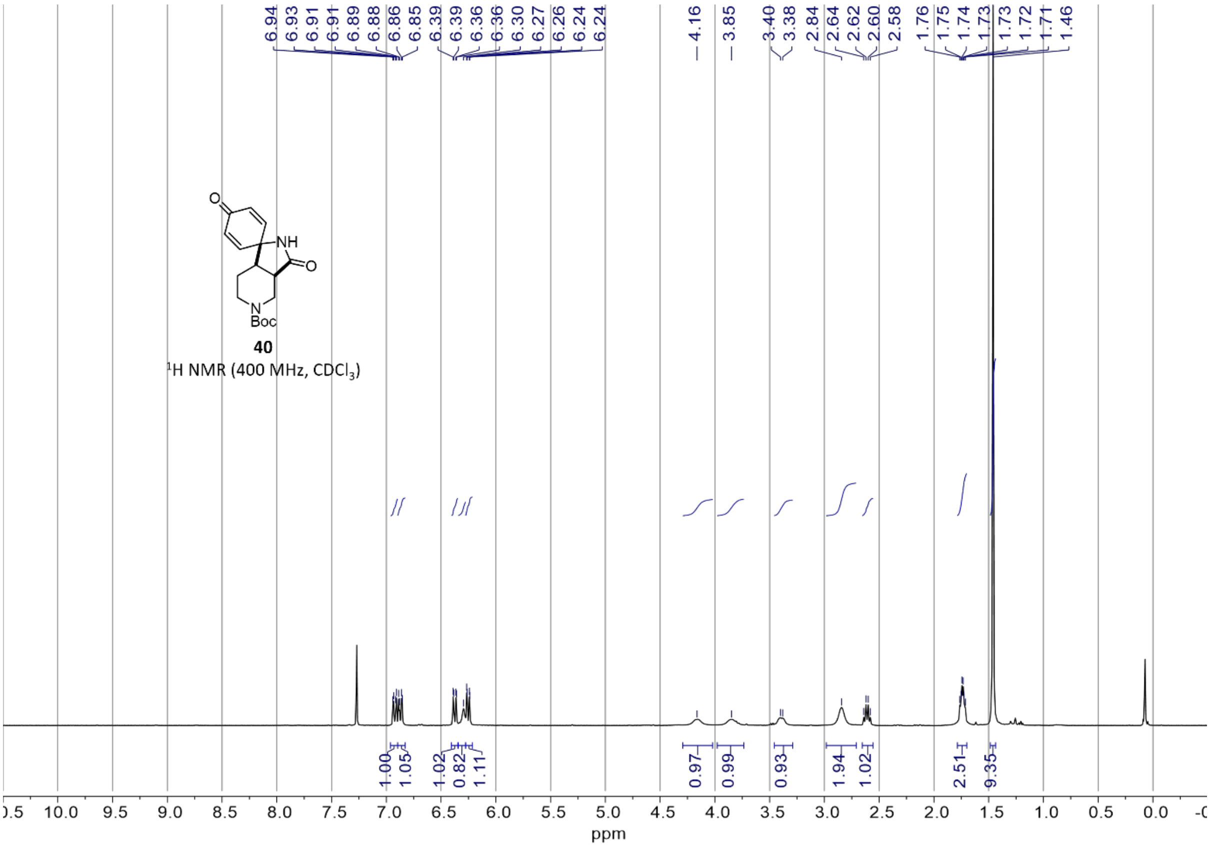

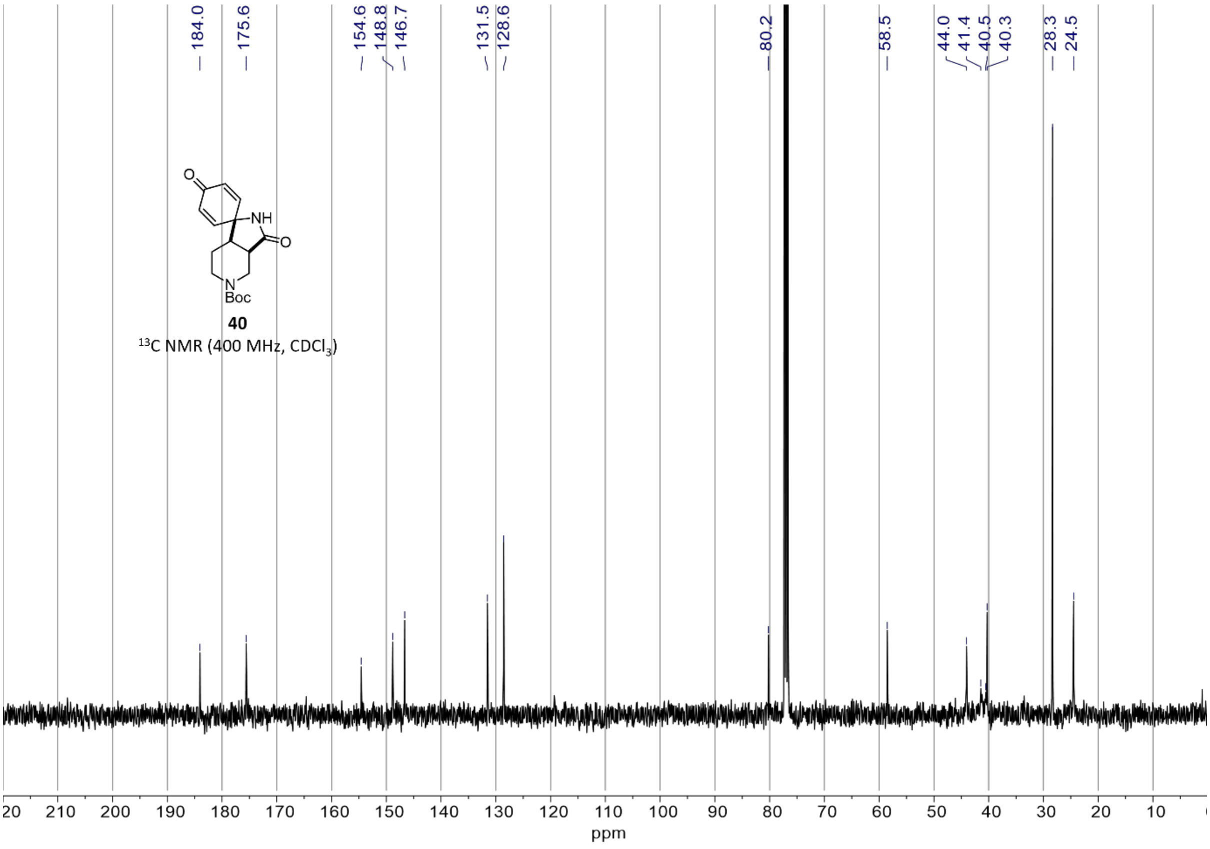

tert-Butyl _trans_-(±)-3',4-dioxo-2',3',3a',6',7',7a'-hexahydrospiro[cyclohexane-1,1'-pyrrolo[3,4-c]pyridine]-2,5-diene-5'(4'H)-carboxylate (41)

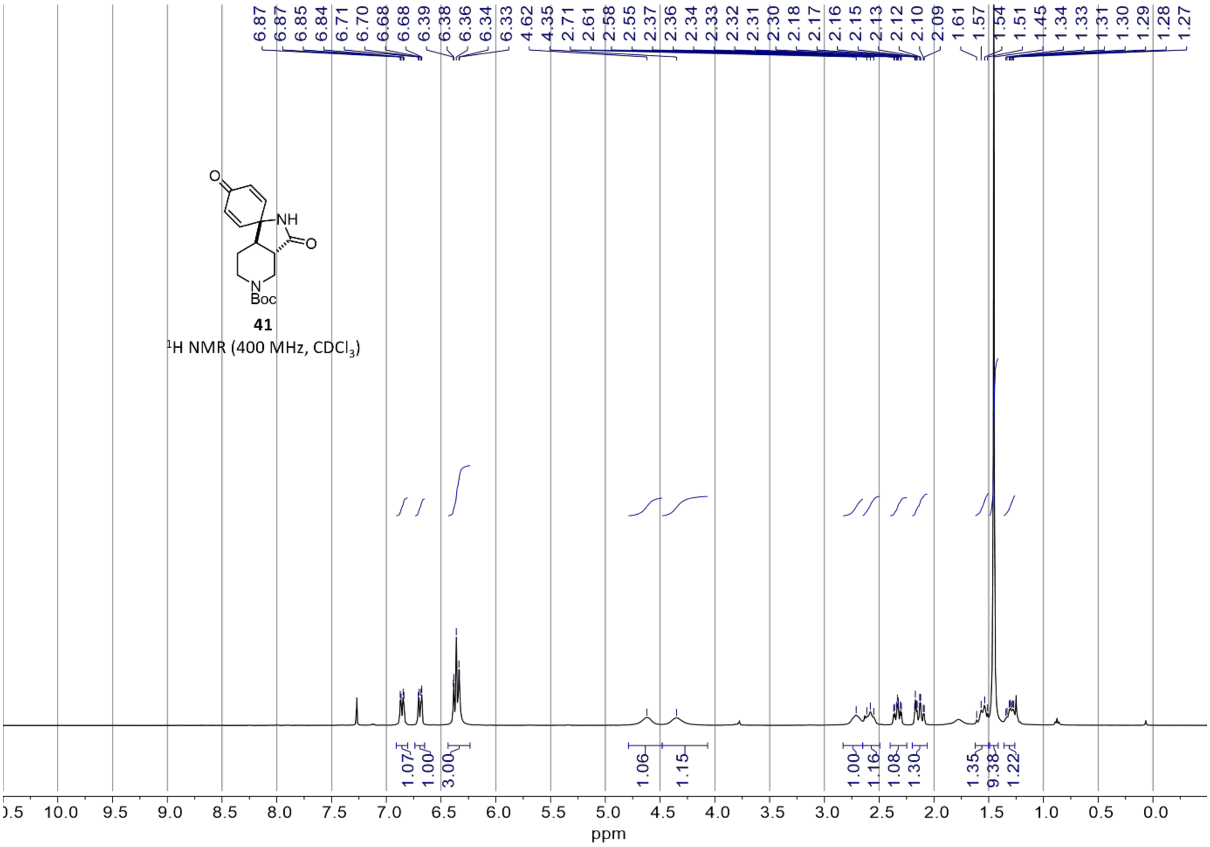

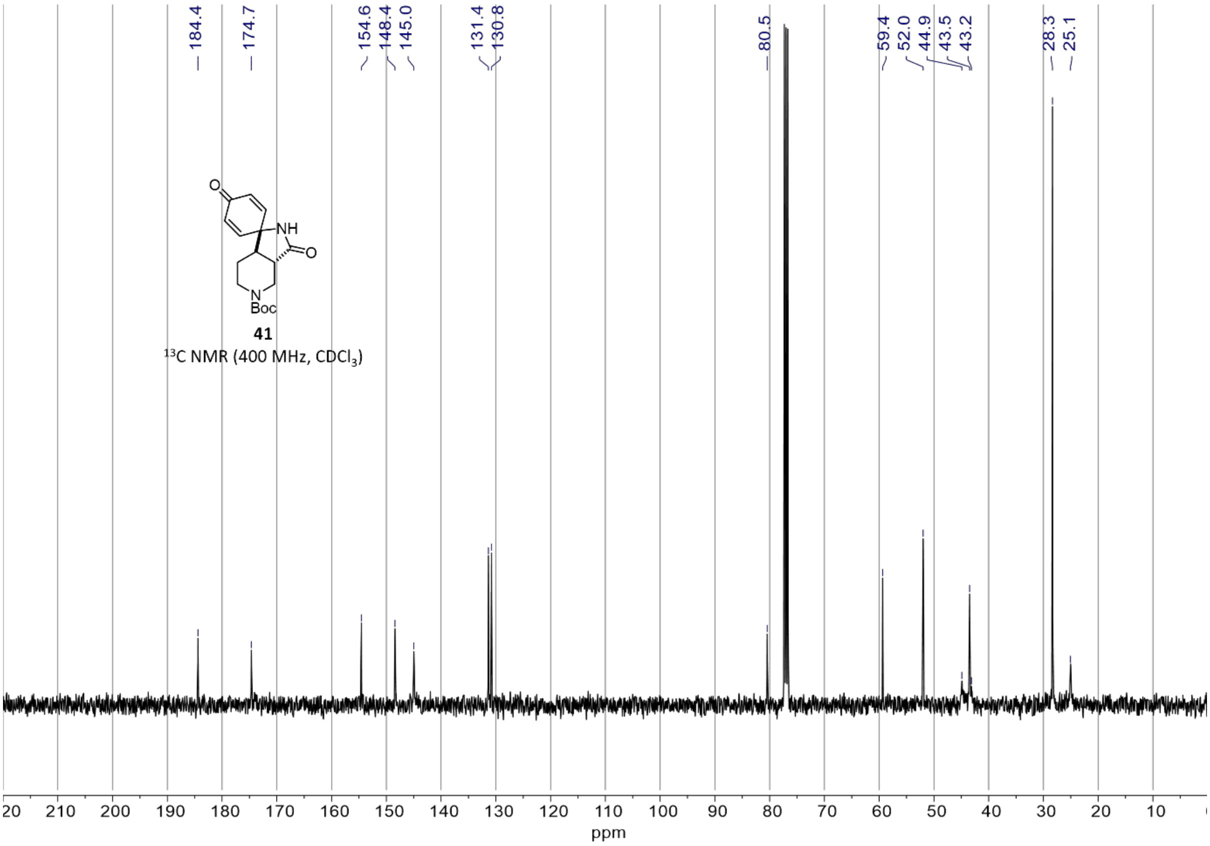

trans-(±)-3',4-Dioxo-2',3',3a',4',5',6',7',7a'-octahydrospiro[cyclohexane-1,1'-pyrrolo[3,4-c]pyridine]-2,5-dien-5'-ium chloride (42)

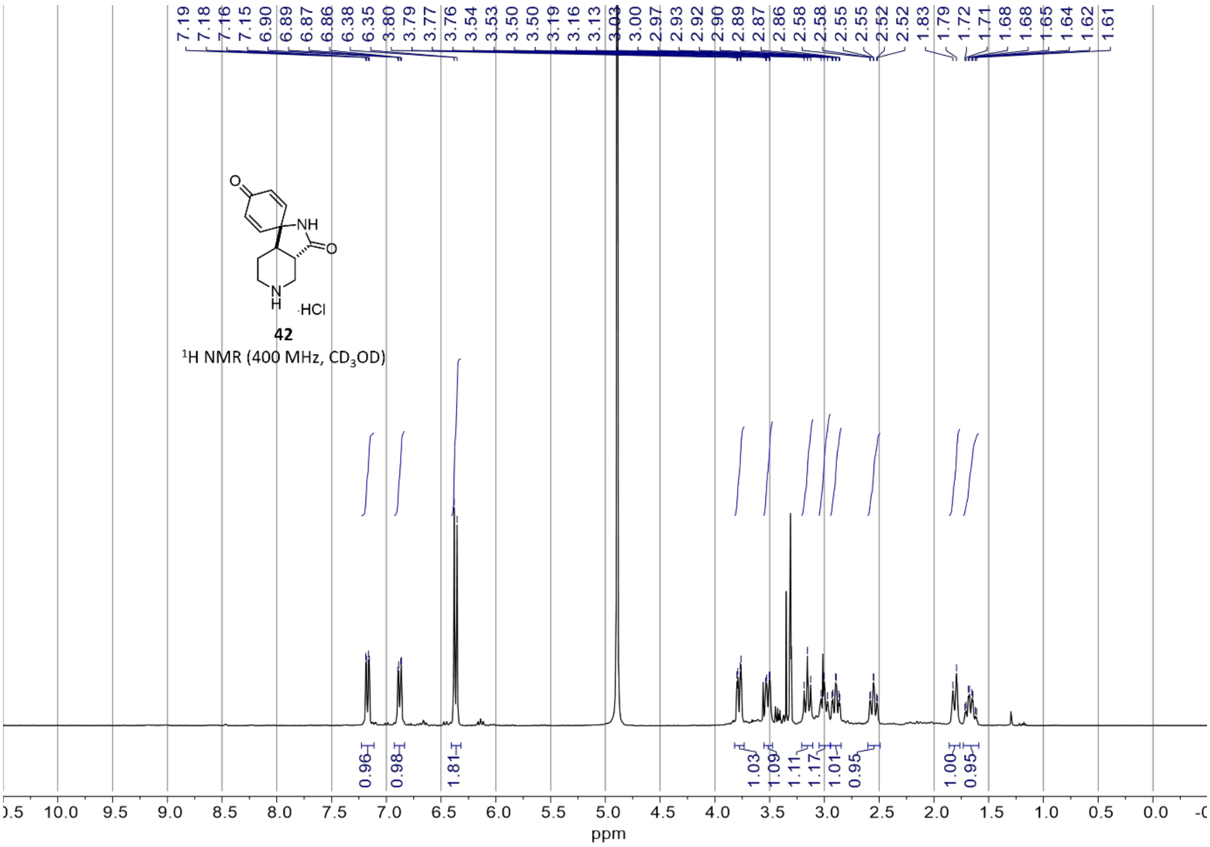

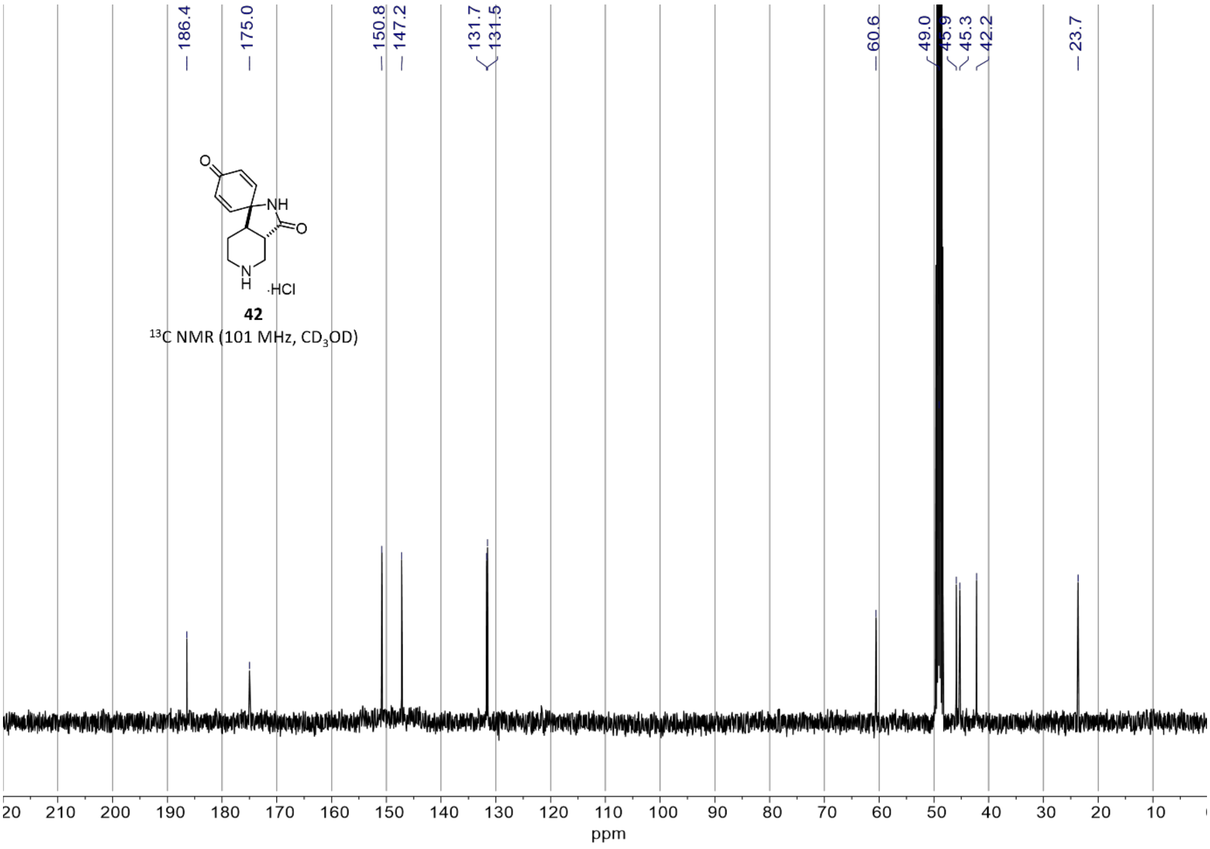

tert-Butyl-cis-(±)-3-(4-methoxyphenyl)-4-(quinolin-8-ylcarbamoyl)piperidine-1-carboxylate (12a)

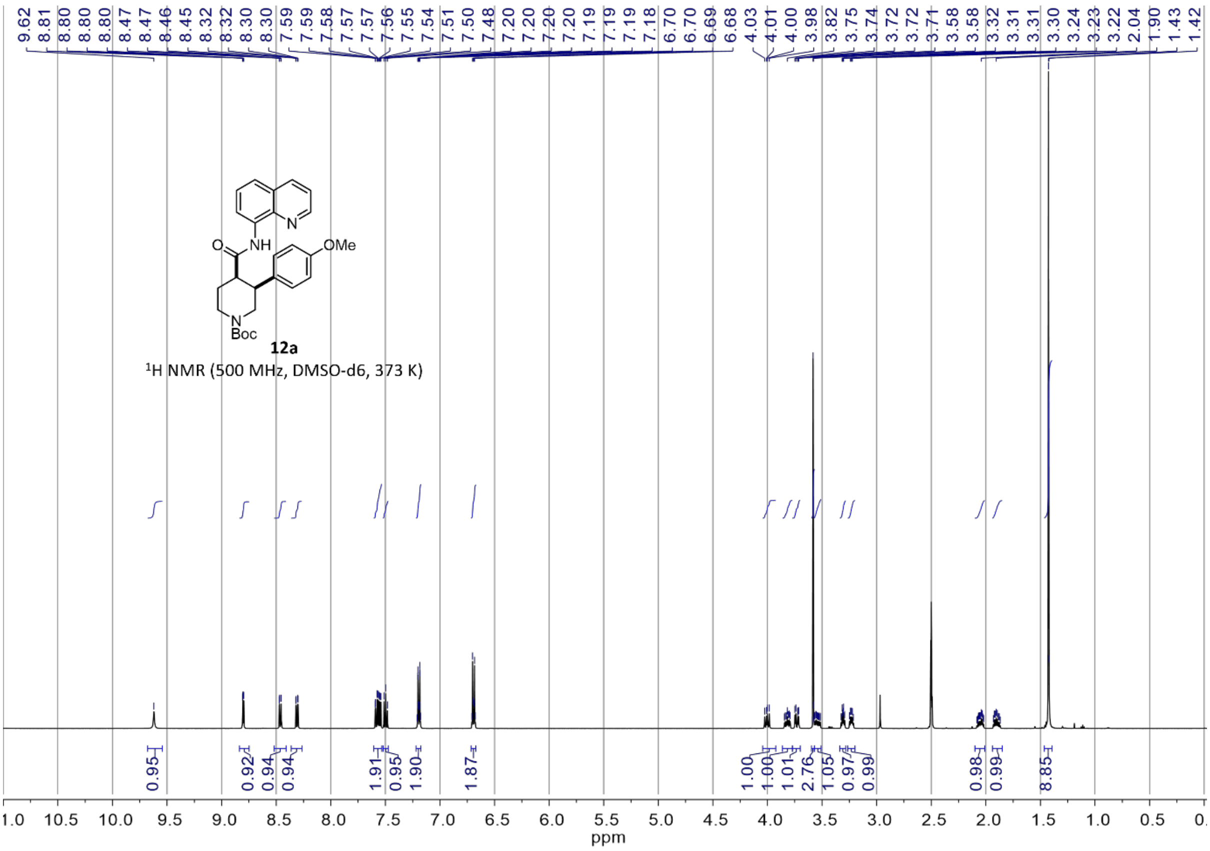

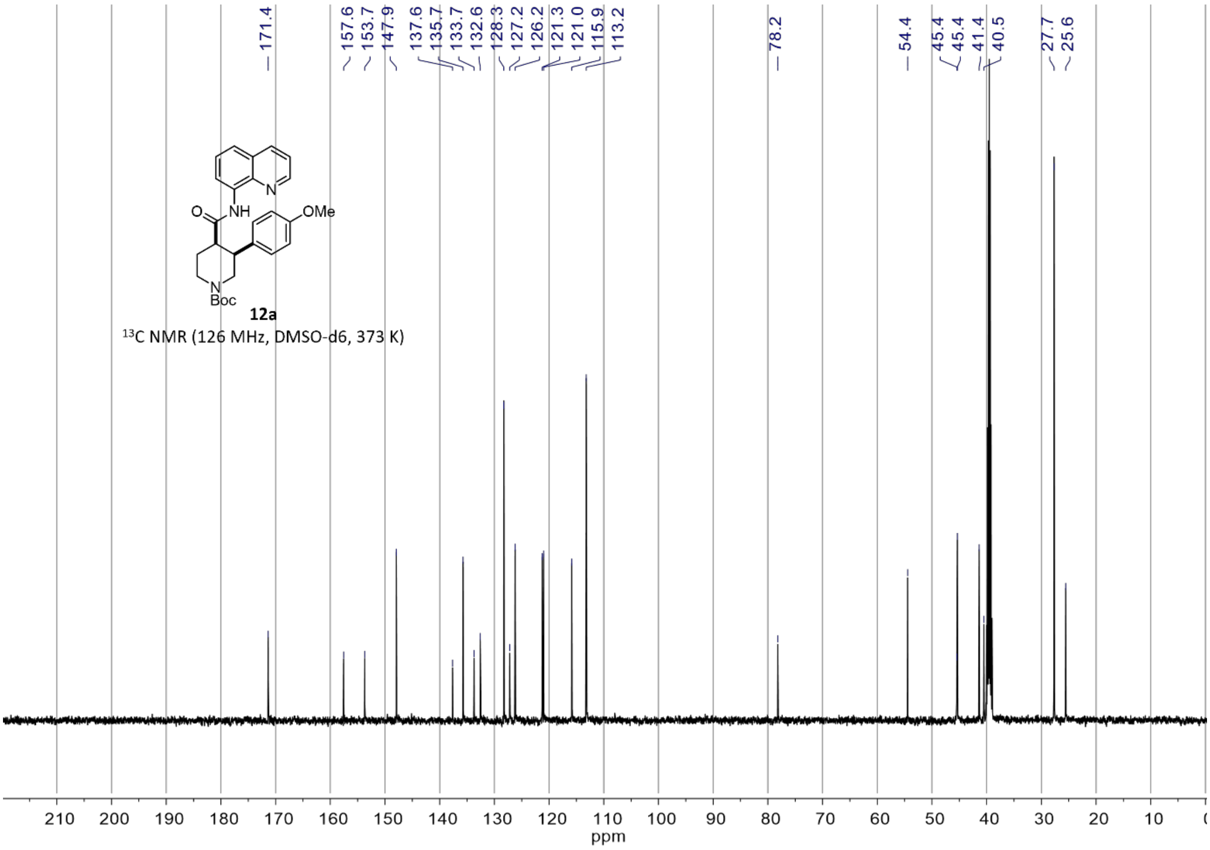

tert-Butyl-_cis_-(±)-3-(4-fluorophenyl)-4-(quinolin-8-ylcarbamoyl)piperidine-1-carboxylate (12b)

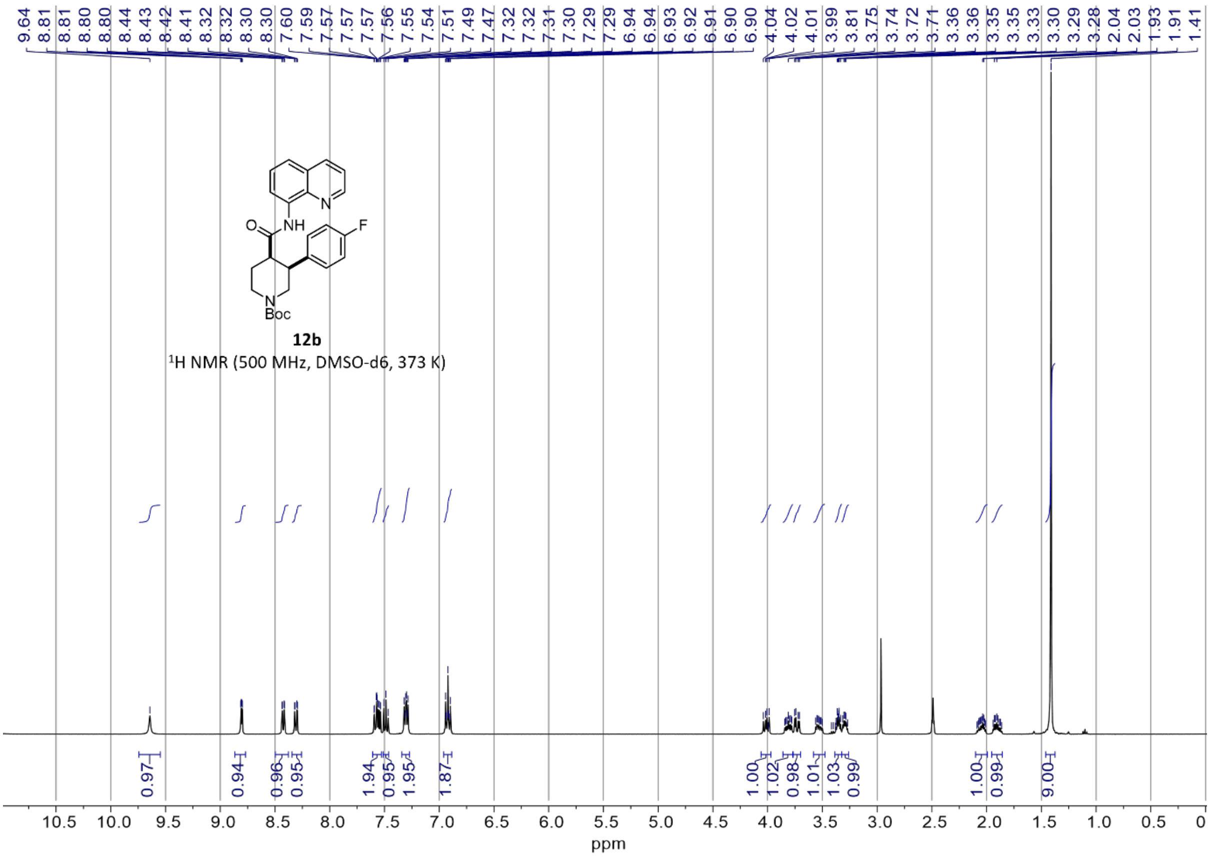

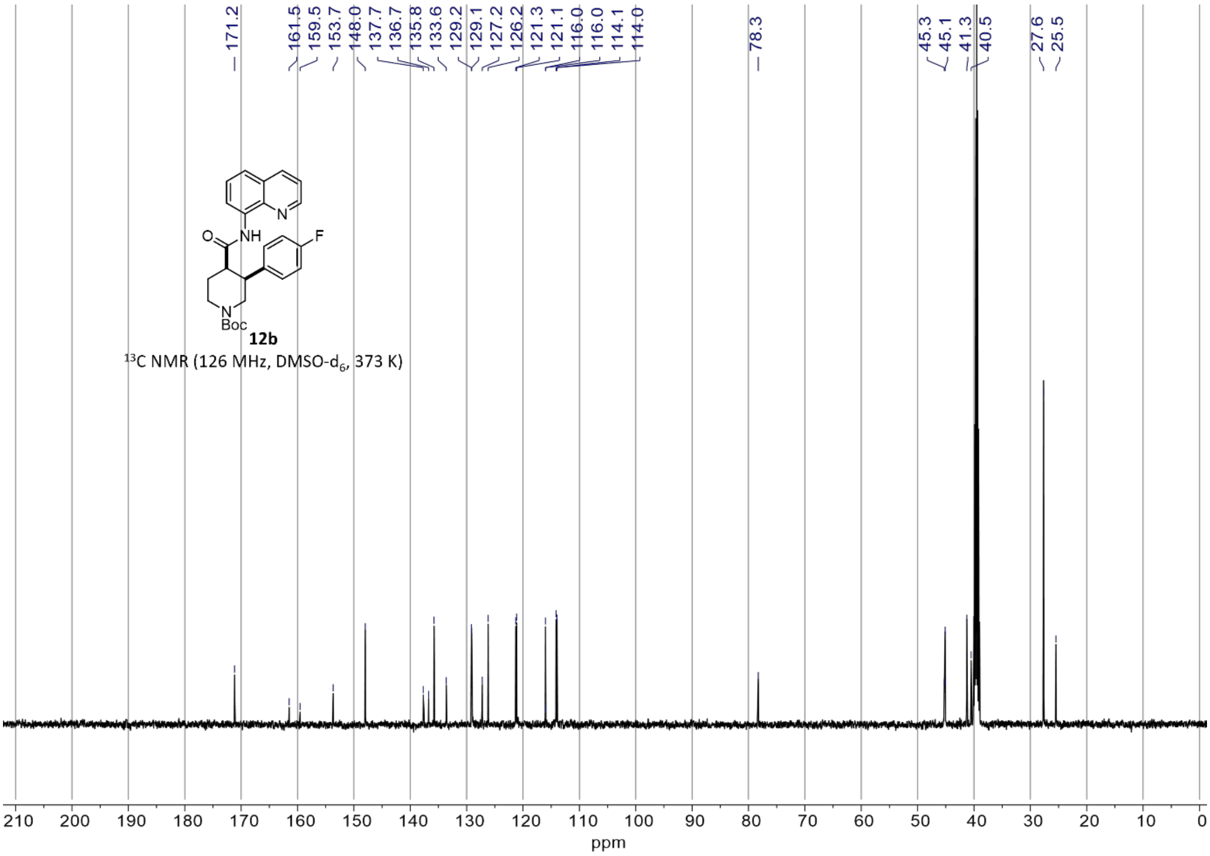

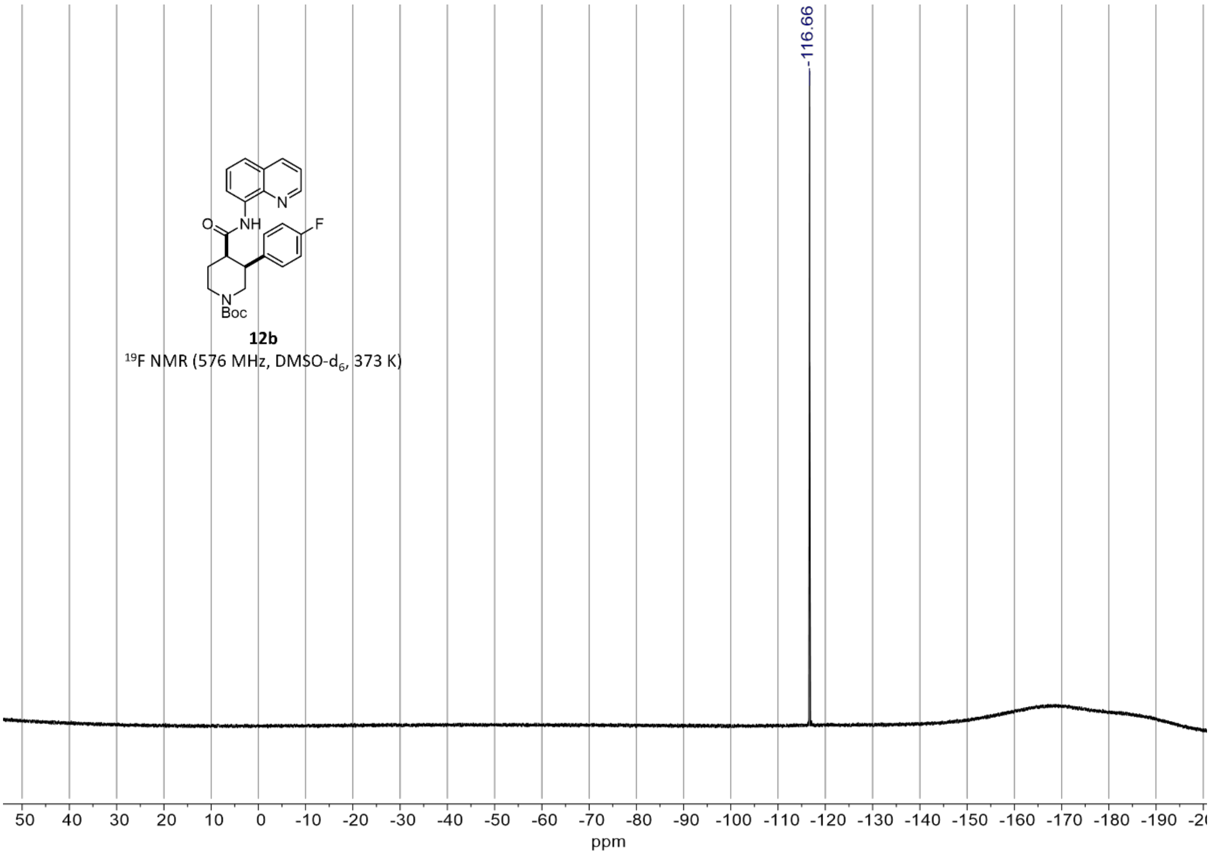

12b

^{19}F NMR (576 MHz, DMSO-d_6, 373 K)

tert-Butyl-trans-(±)-3-(4-methoxyphenyl)-4-(quinolin-8-ylcarbamoyl)piperidine-1-carboxylate (13a)

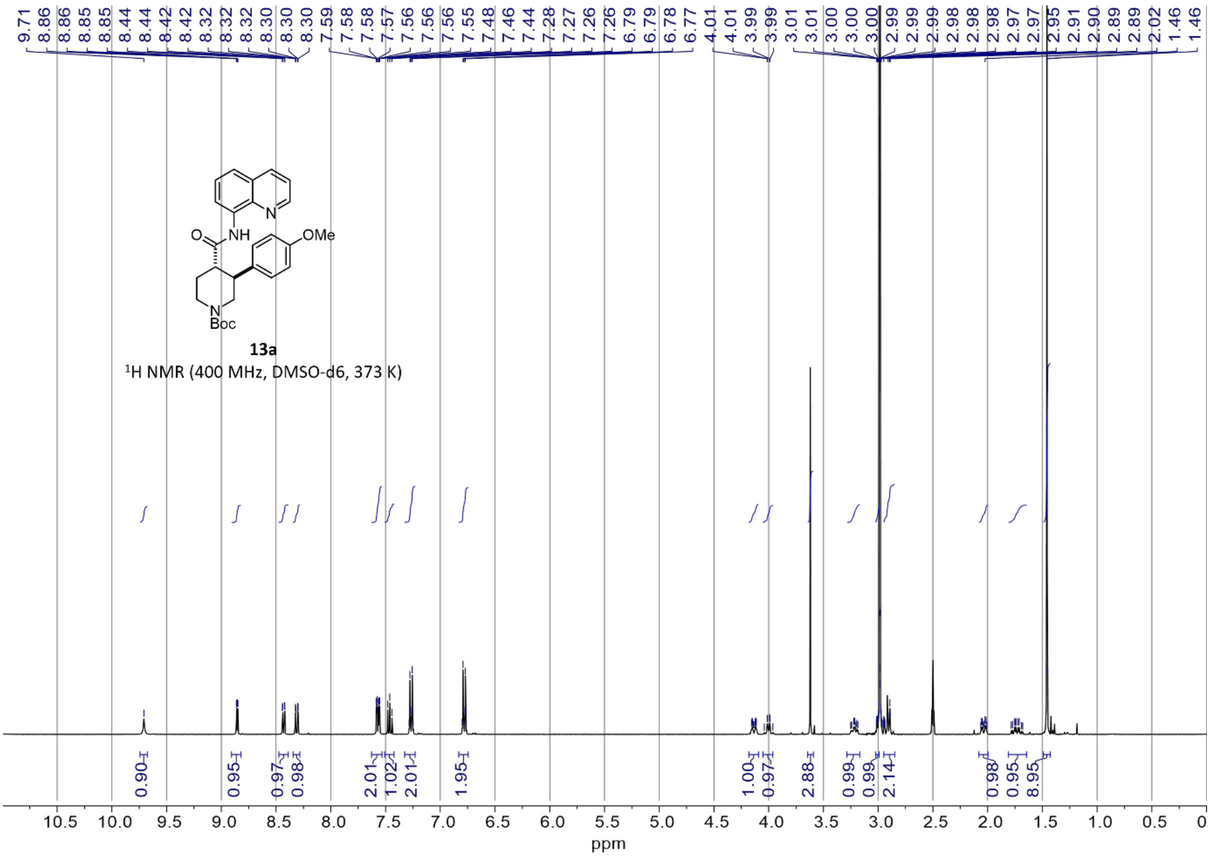

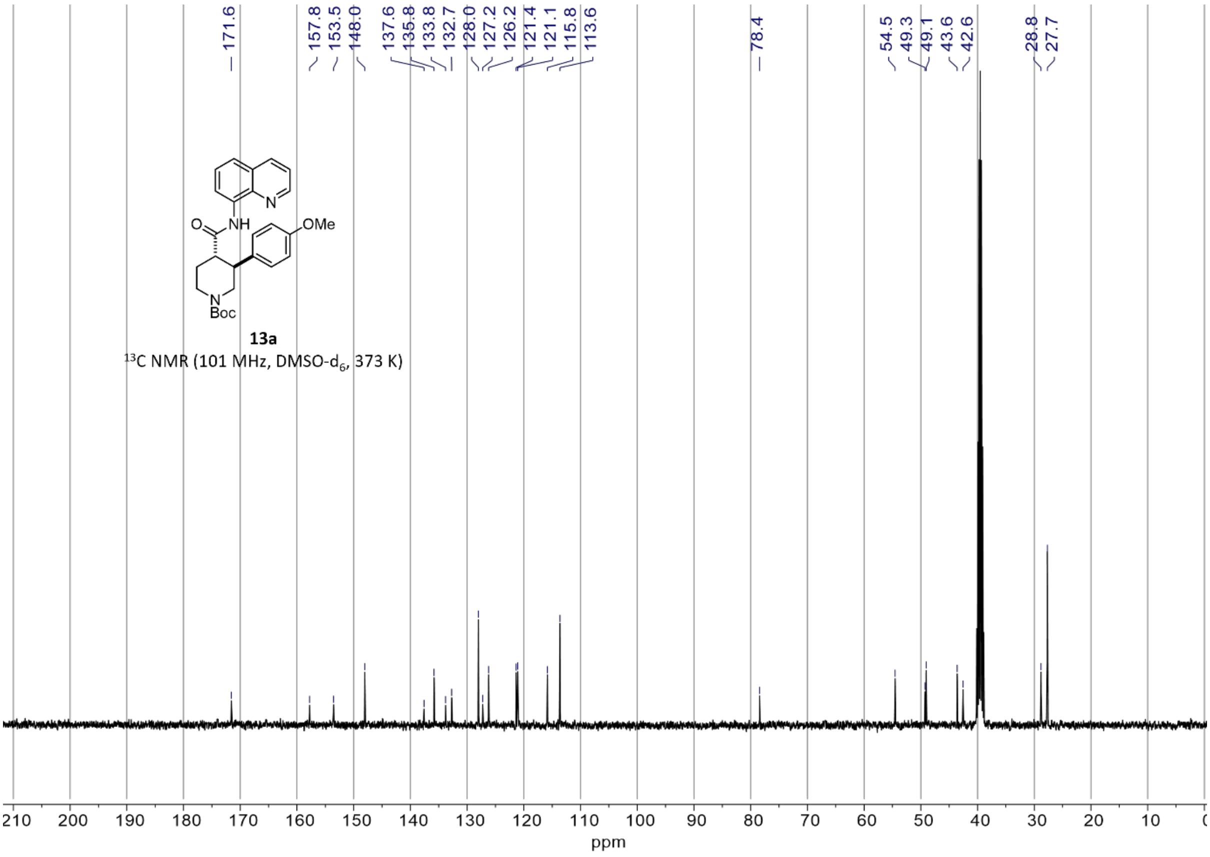

tert-Butyl-_trans_-(±)-3-(4-fluorophenyl)-4-(quinolin-8-ylcarbamoyl)piperidine-1-carboxylate (13b)

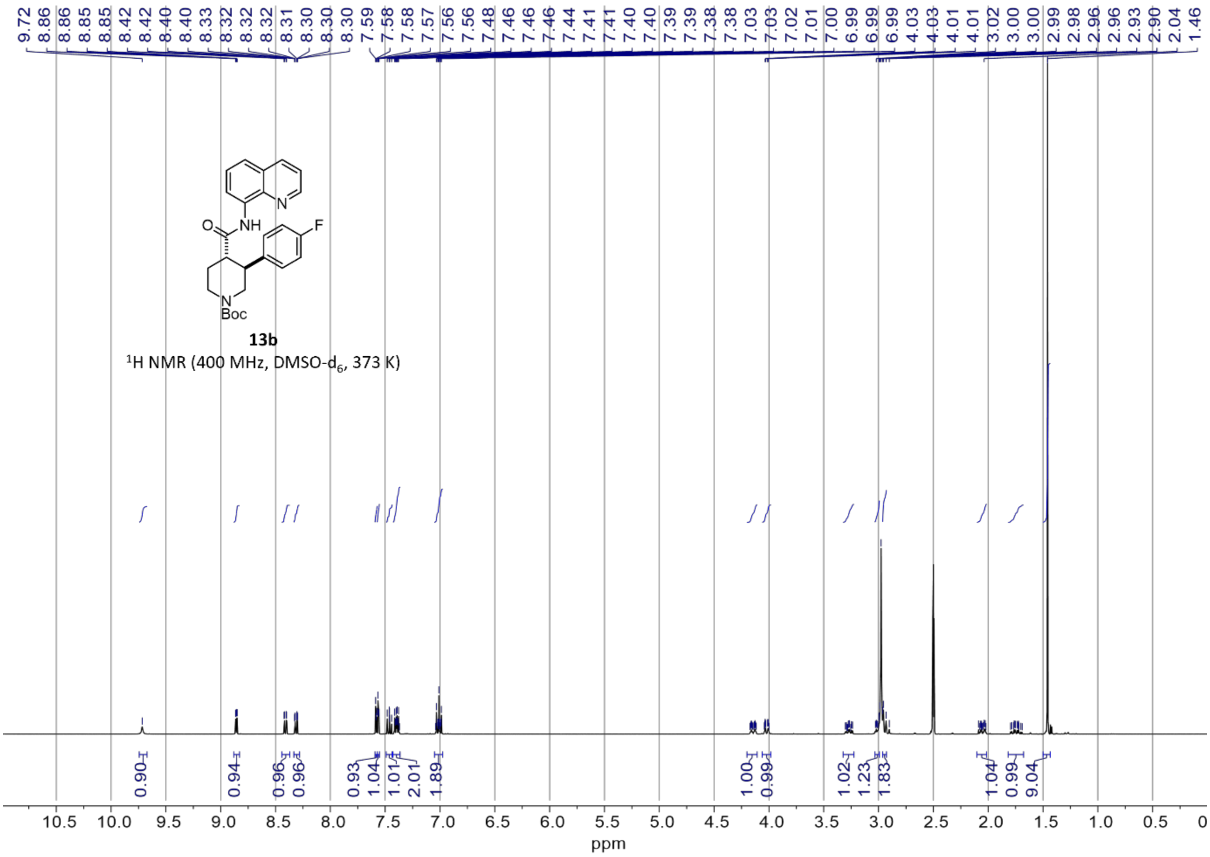

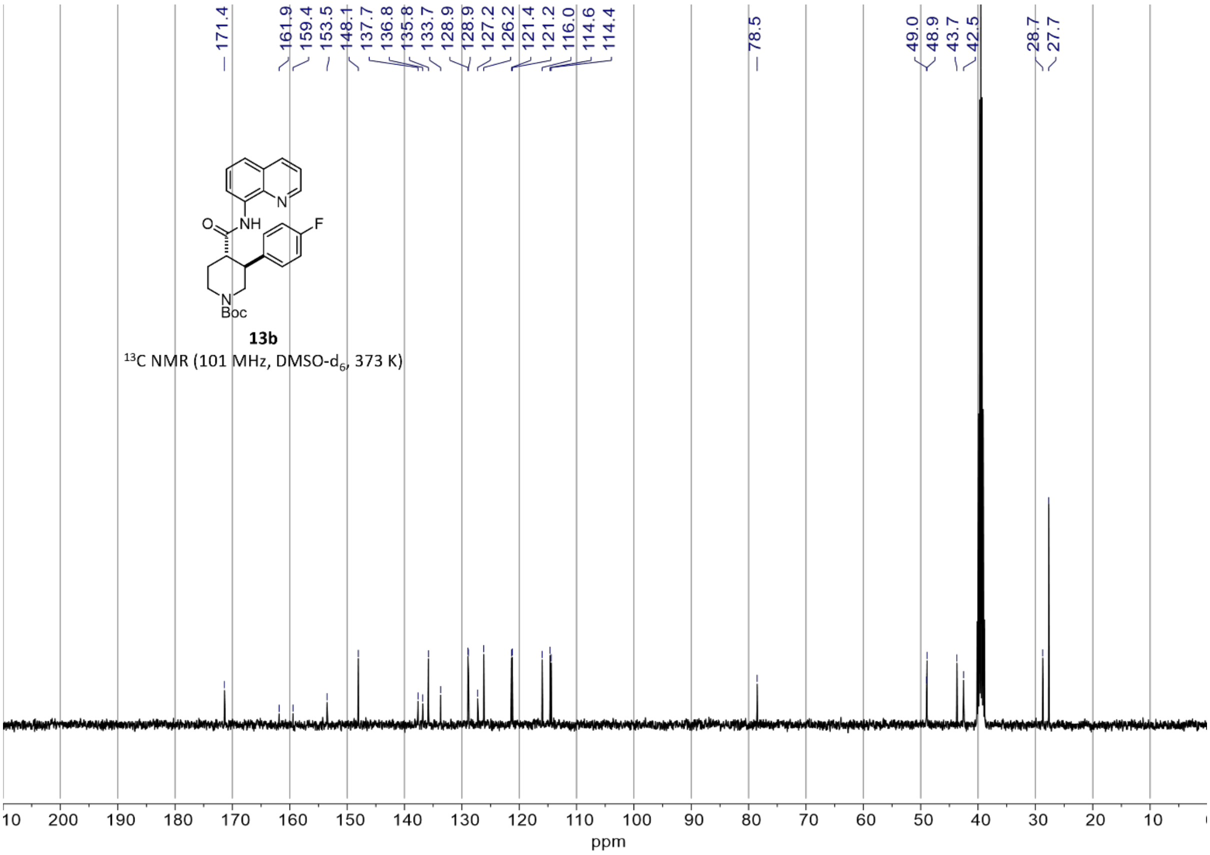

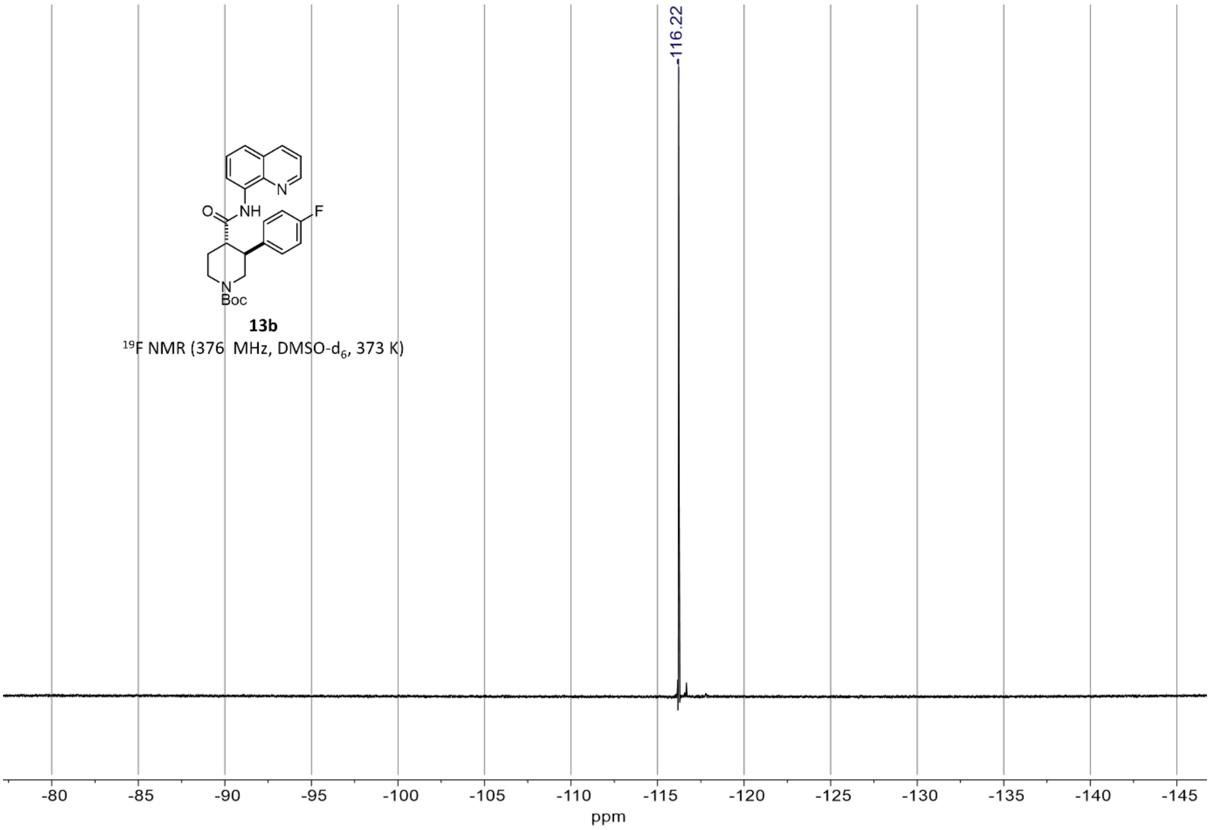

-116.22
O
NH
N
F
N
Boc
13b
19F NMR (376 MHz, DMSO-d6, 373 K)
-80 -85 -90 -95 -100 -105 -110 -115 -120 -125 -130 -135 -140 -145
ppm

cis-(±)-3-(4-Methoxyphenyl)-_N_-(quinolin-8-yl)tetrahydro-2H-thiopyran-4-carboxamide 1,1-dioxide (15a)

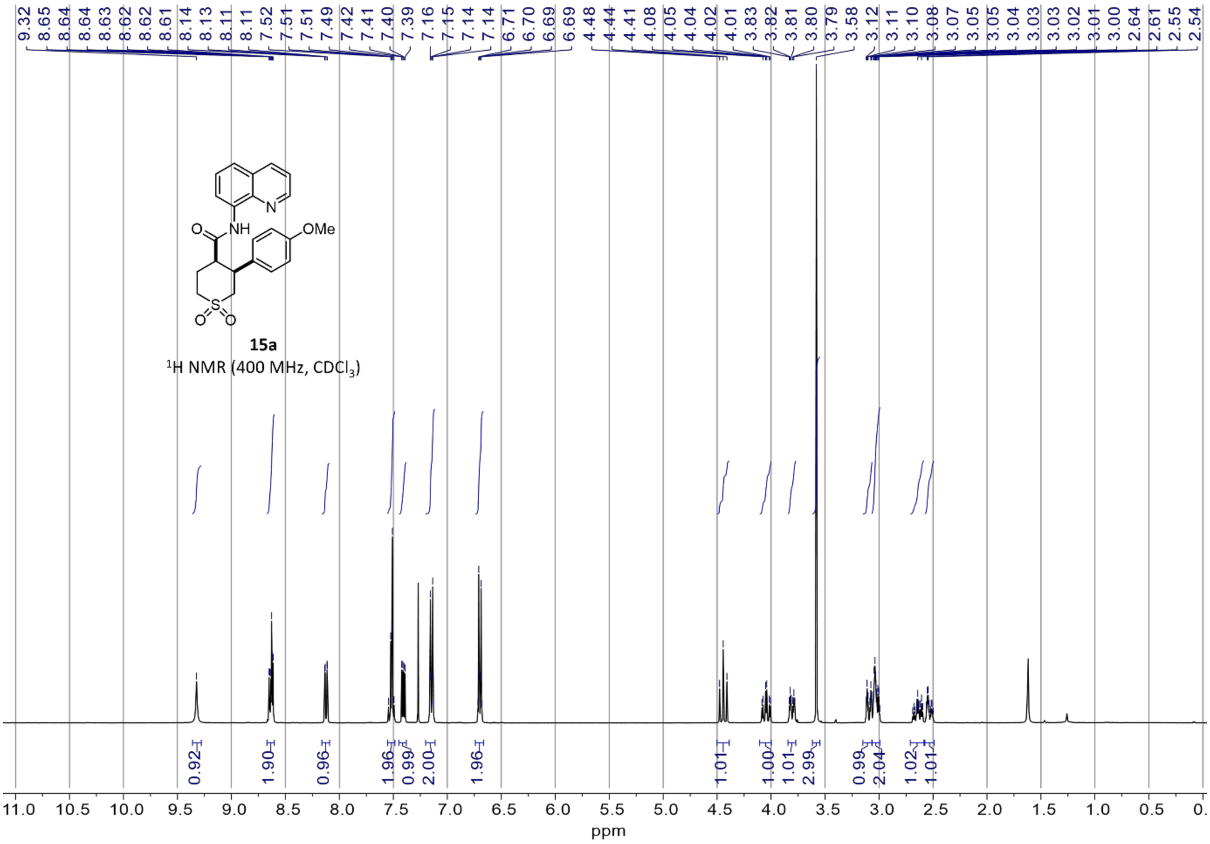

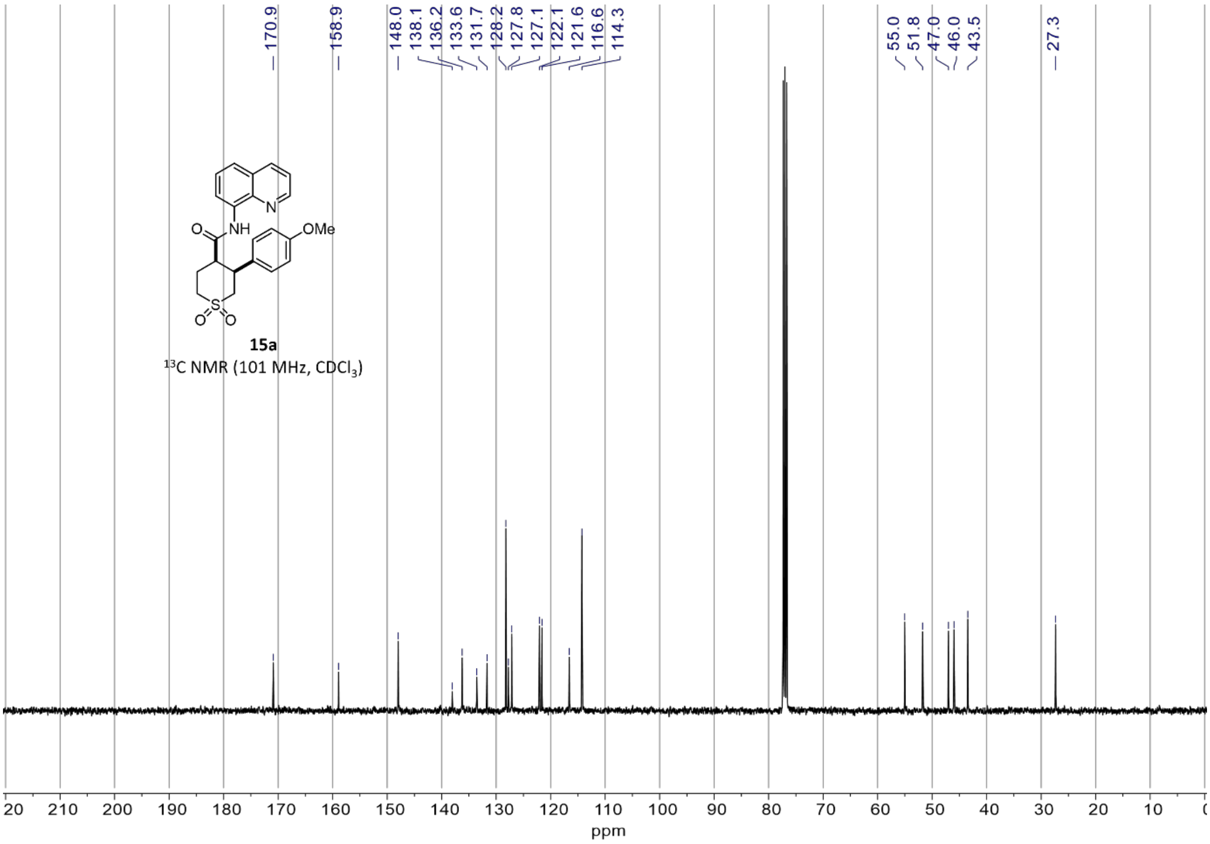

cis-(±)-4,4-Difluoro-2-(4-methoxyphenyl)-_N_-(quinolin-8-yl)cyclohexane-1-carboxamide (17a)

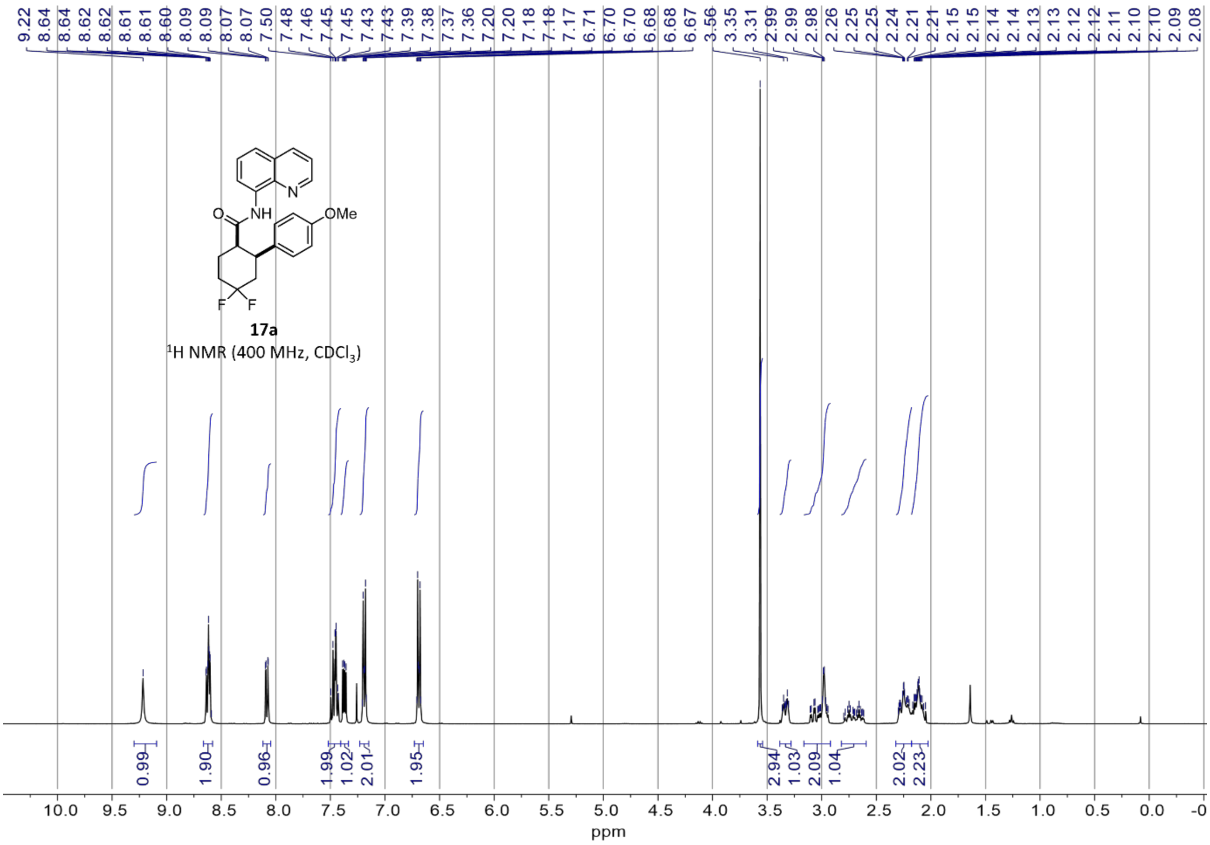

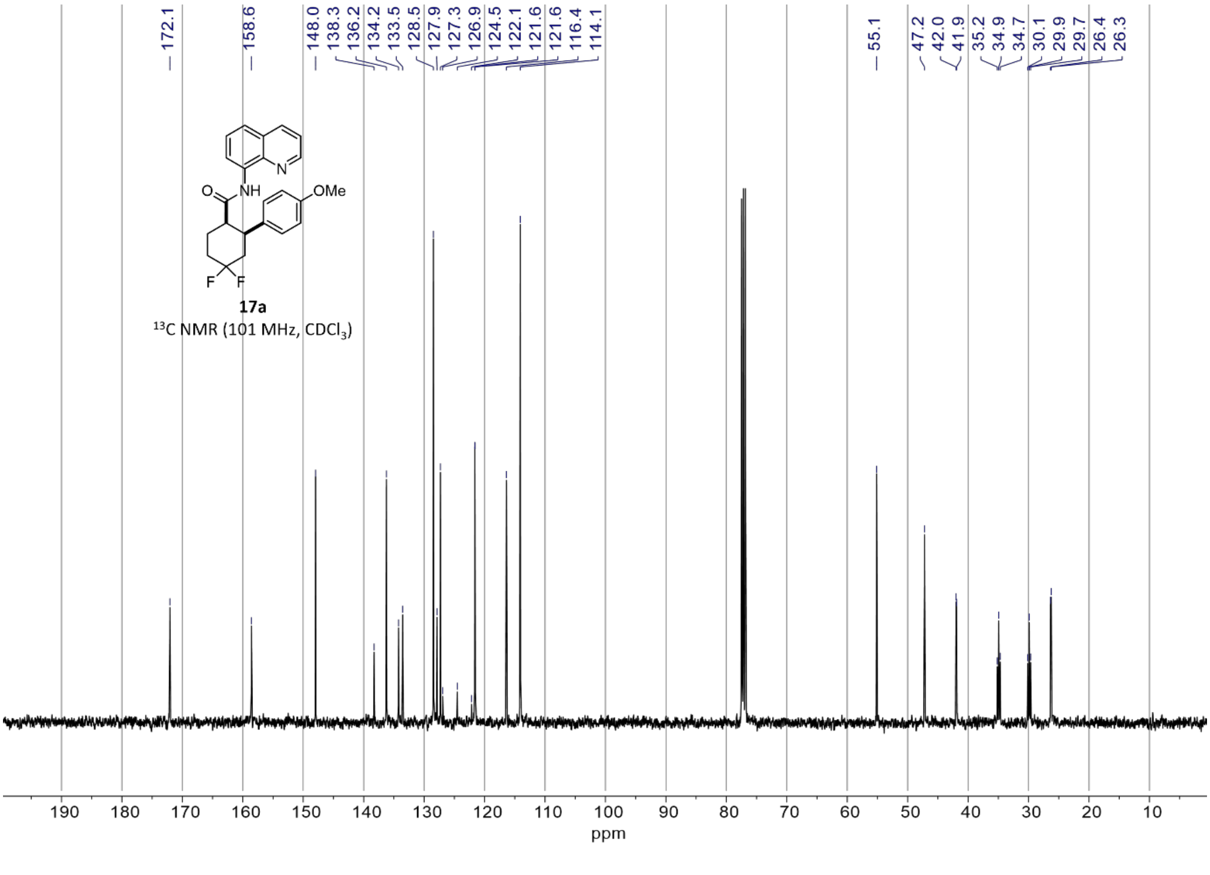

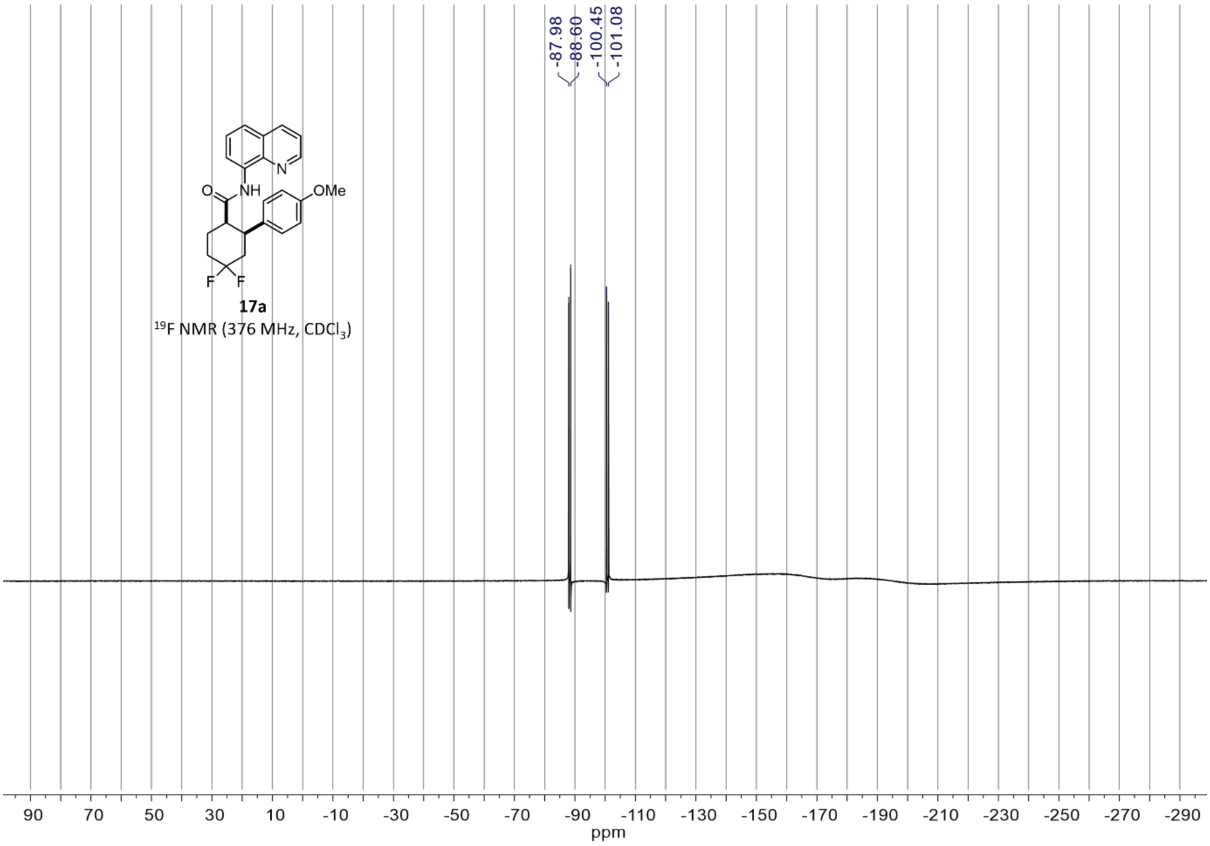
-87.98
-88.60
-100.45
-101.08
OMe
NH
N
O
F
F
17a
19F NMR (376 MHz, CDCl3)
90 70 50 30 10 -10 -30 -50 -70 -90 -110 -130 -150 -170 -190 -210 -230 -250 -270 -290
ppm

trans-(±)-4,4-Difluoro-2-(4-methoxyphenyl)-N-(quinolin-8-yl)cyclohexane-1-carboxamide (18a)

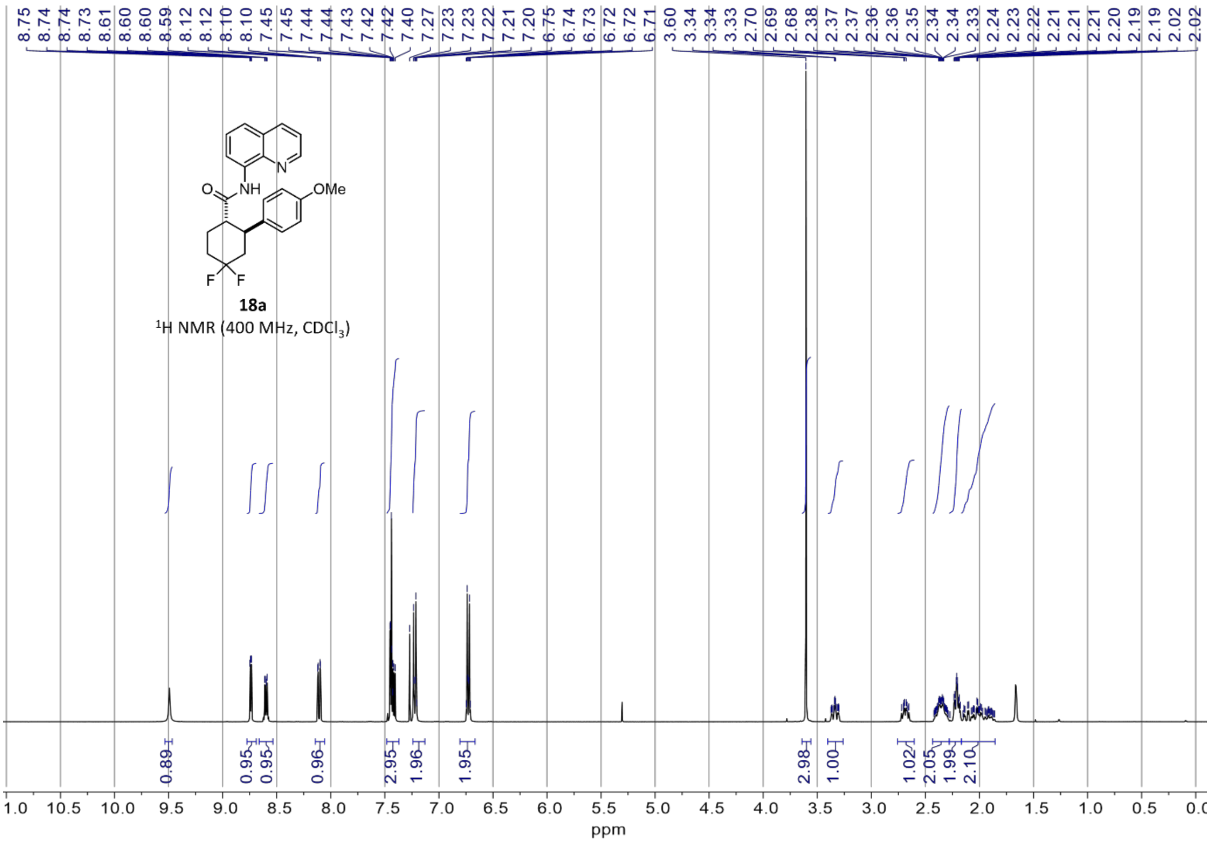

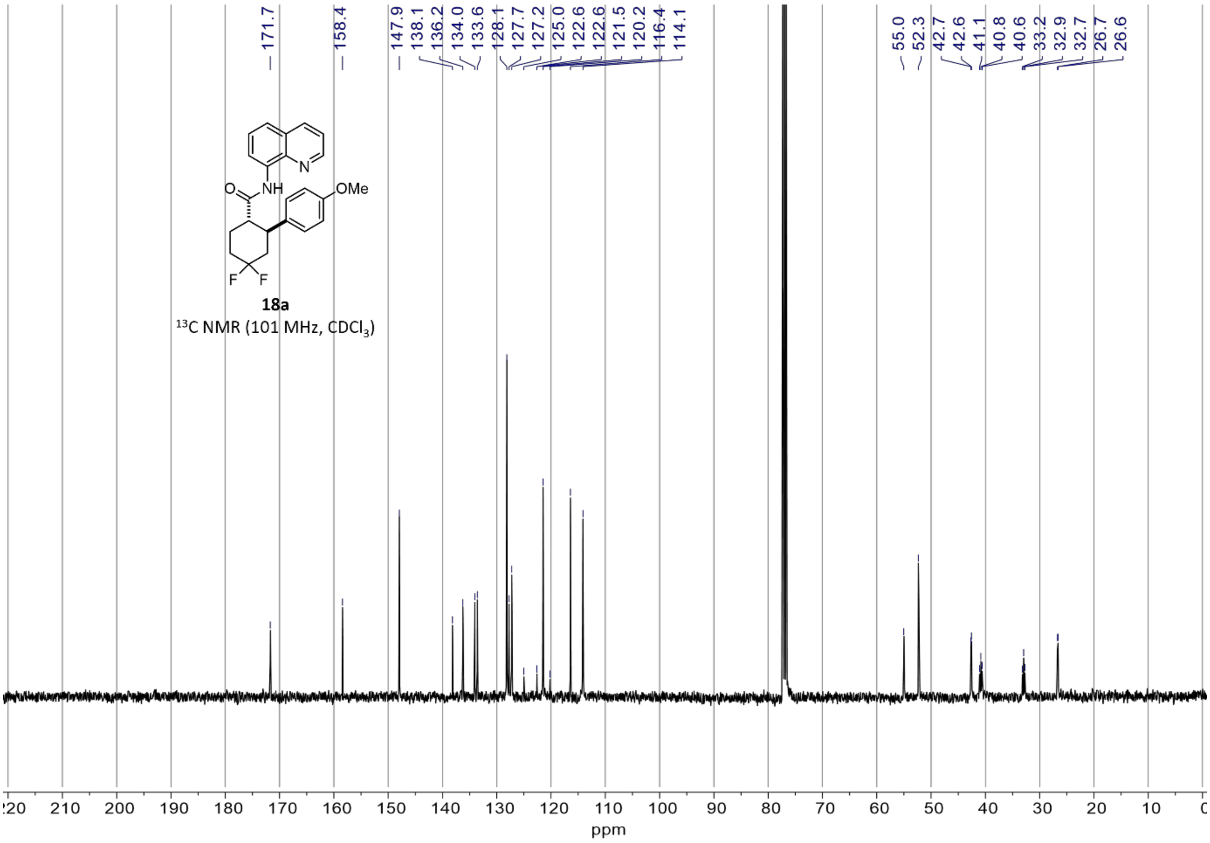

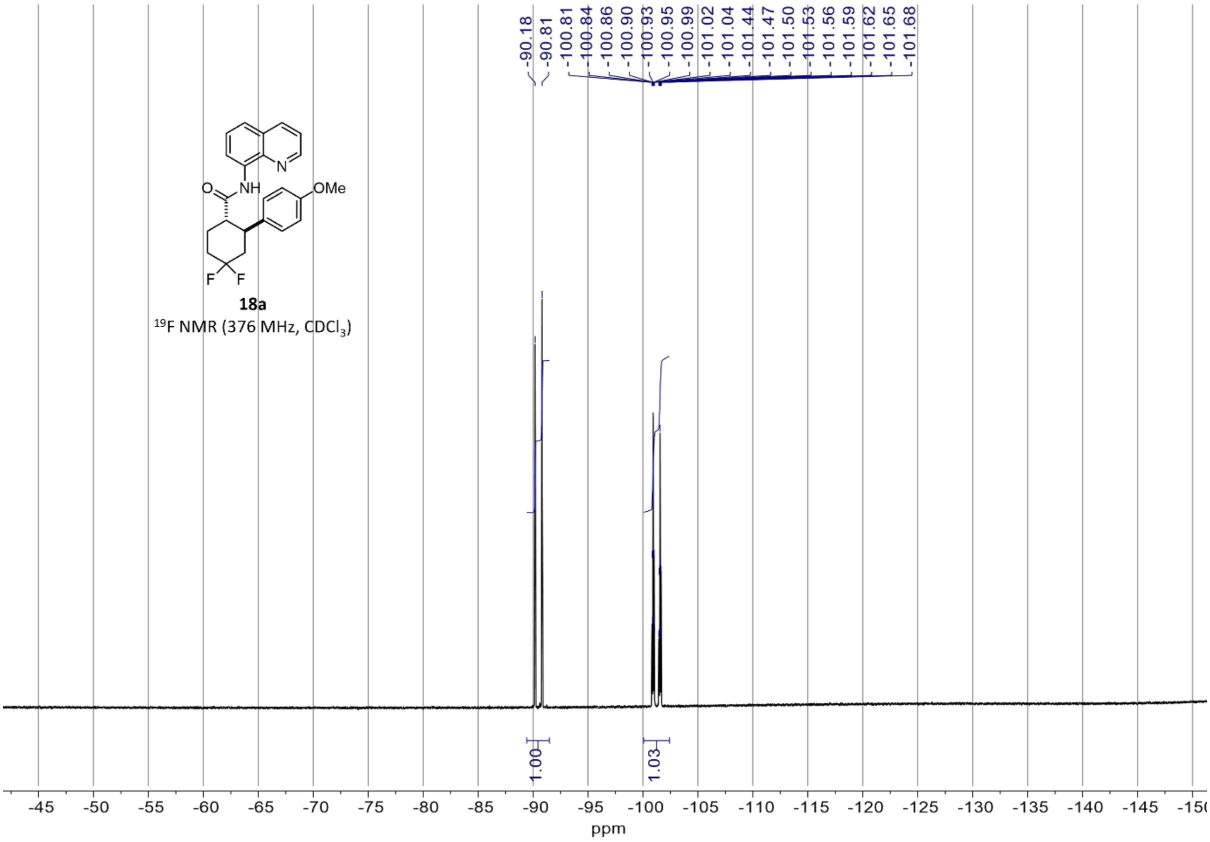

-90.18
-90.81
-100.81
-100.84
-100.86
-100.90
-100.93
-100.95
-100.99
-101.02
-101.04
-101.44
-101.47
-101.50
-101.53
-101.56
-101.59
-101.62
-101.65
-101.68
OMe
NH
N
O
F
F
18a
^{19}F NMR (376 MHz, CDCl$_3$)
1.00
1.03
-45
-50
-55
-60
-65
-70
-75
-80
-85
-90
-95
-100
-105
-110
-115
-120
-125
-130
-135
-140
-145
ppm

cis-(±)-1-(_tert_-Butoxycarbonyl)-3-(4-methoxyphenyl)piperidine-4-carboxylic acid (45a)

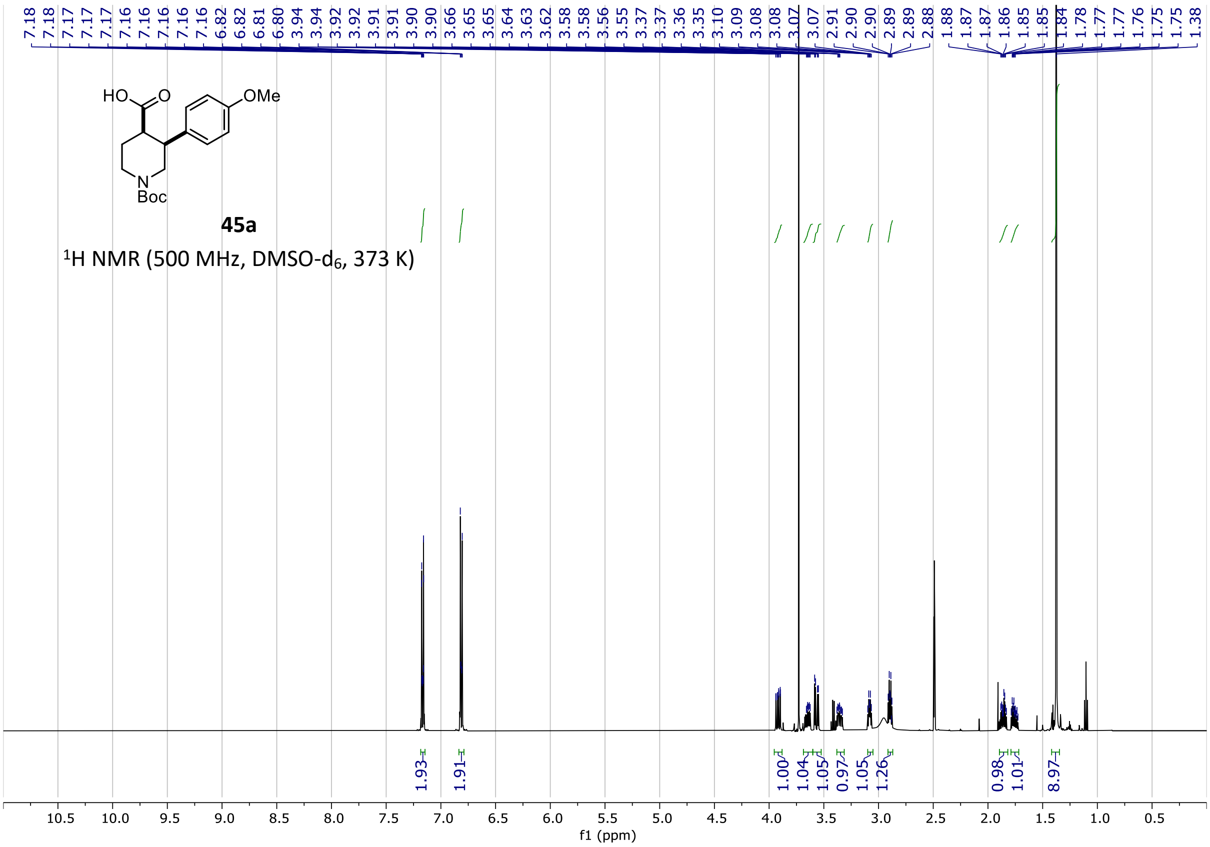

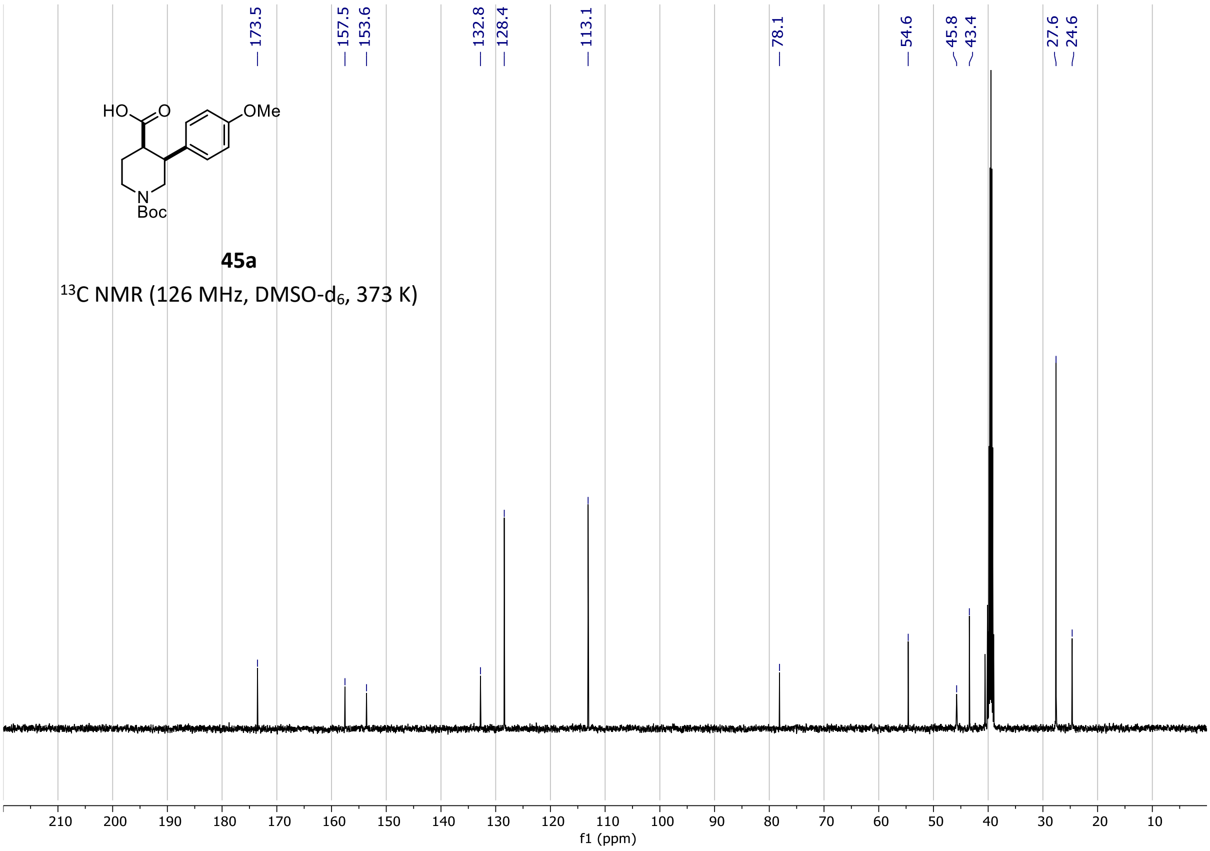

trans-(±)-1-(tert-Butoxycarbonyl)-3-(4-methoxyphenyl)piperidine-4-carboxylic acid (46a)

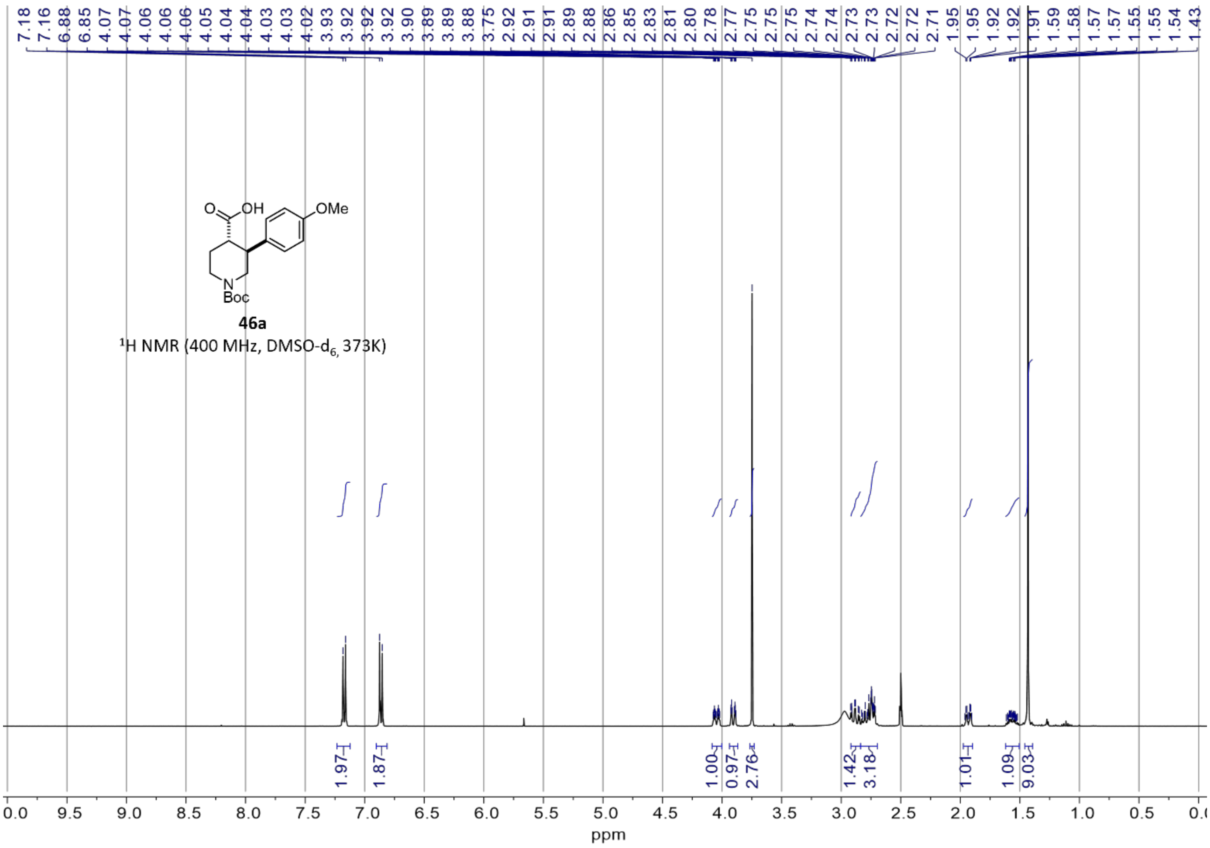

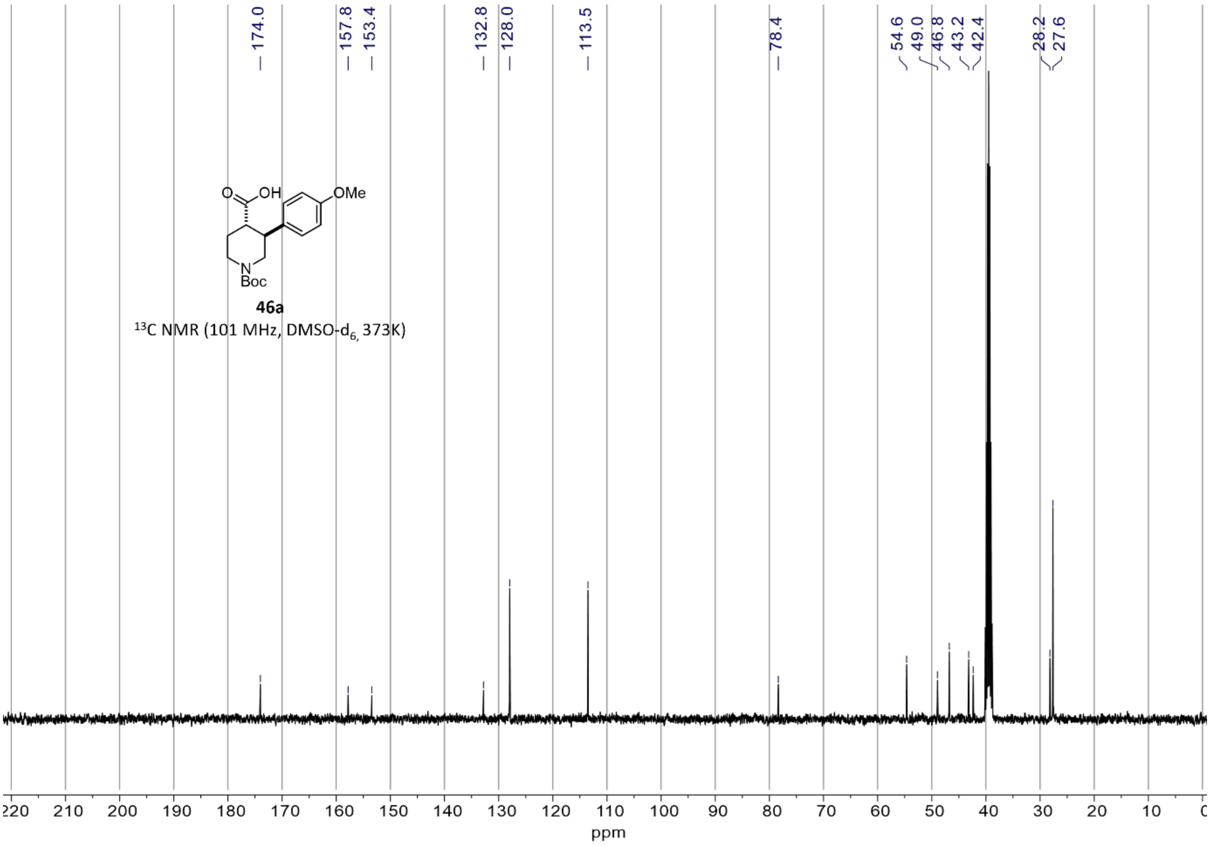

cis-(±)-1-(_tert_-Butoxycarbonyl)-3-(4-fluorophenyl)piperidine-4-carboxylic acid (45b)

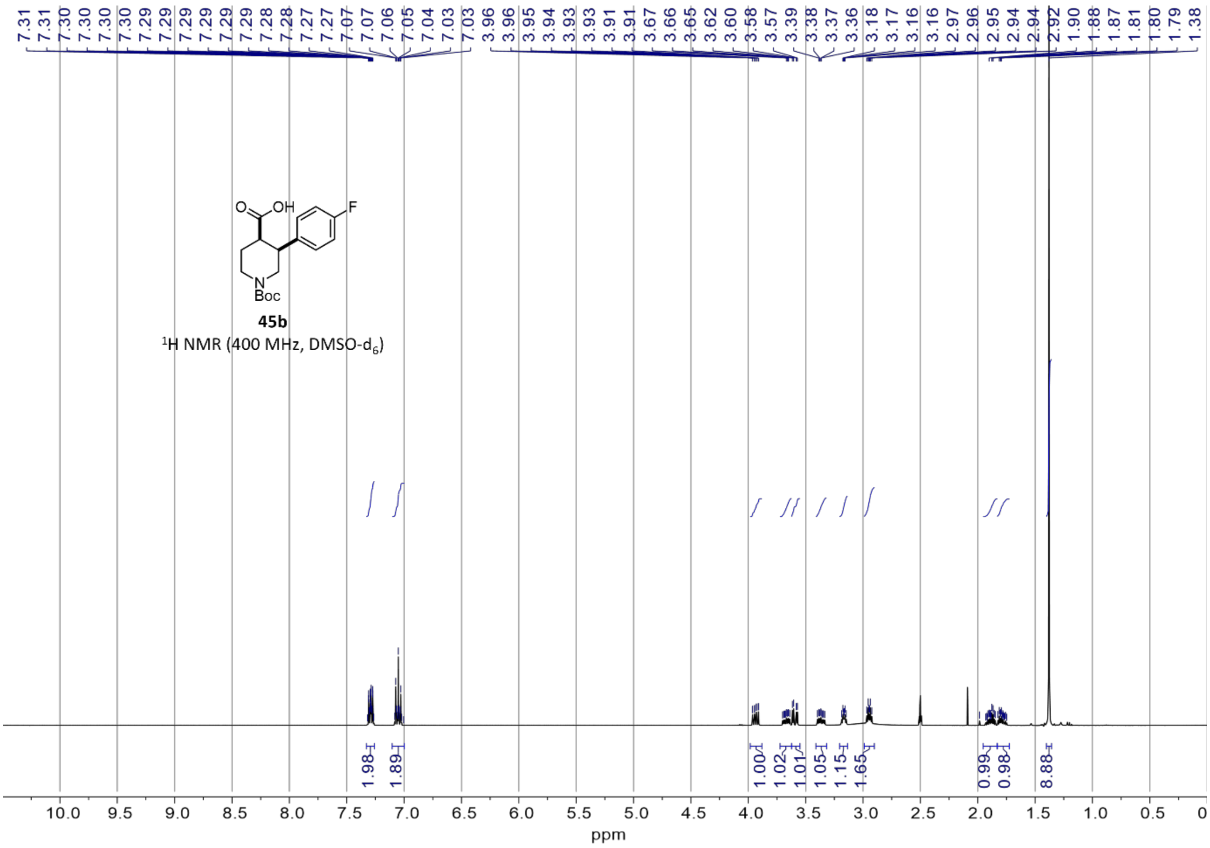

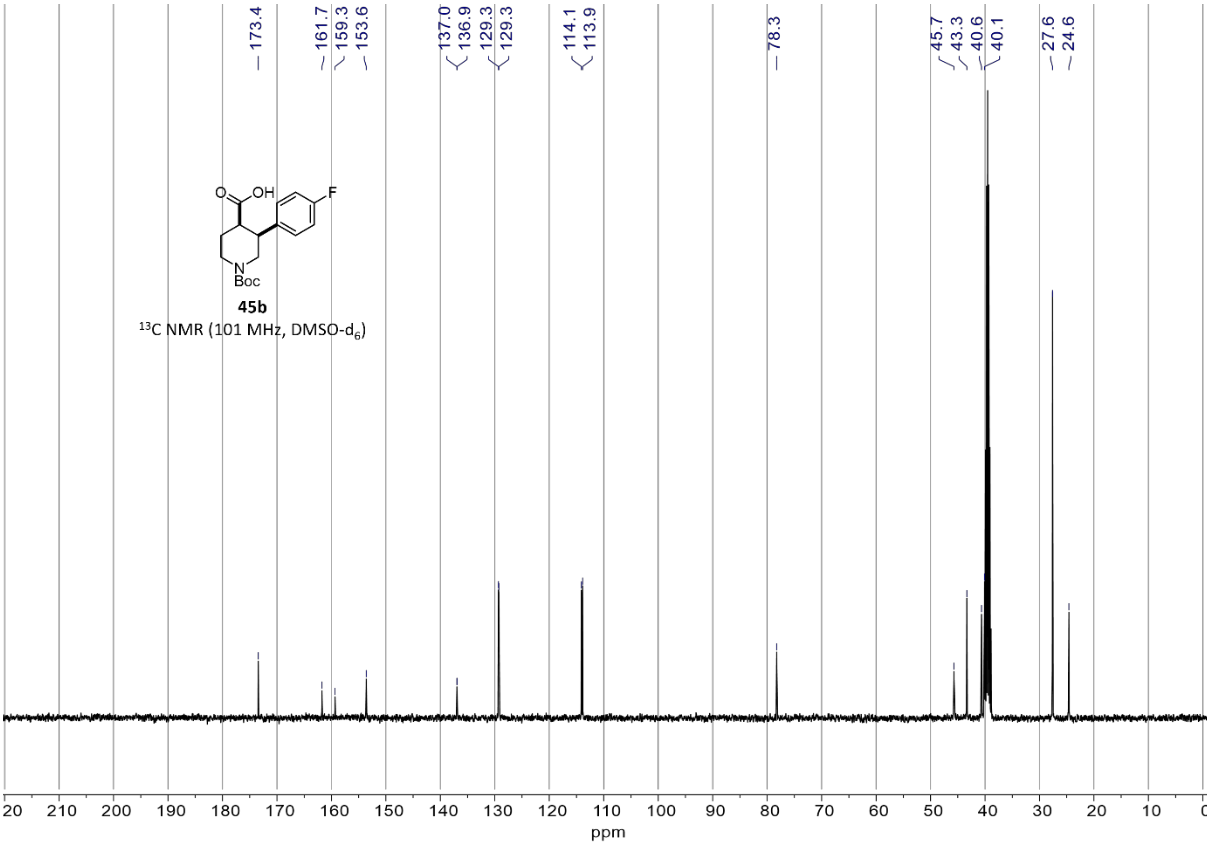

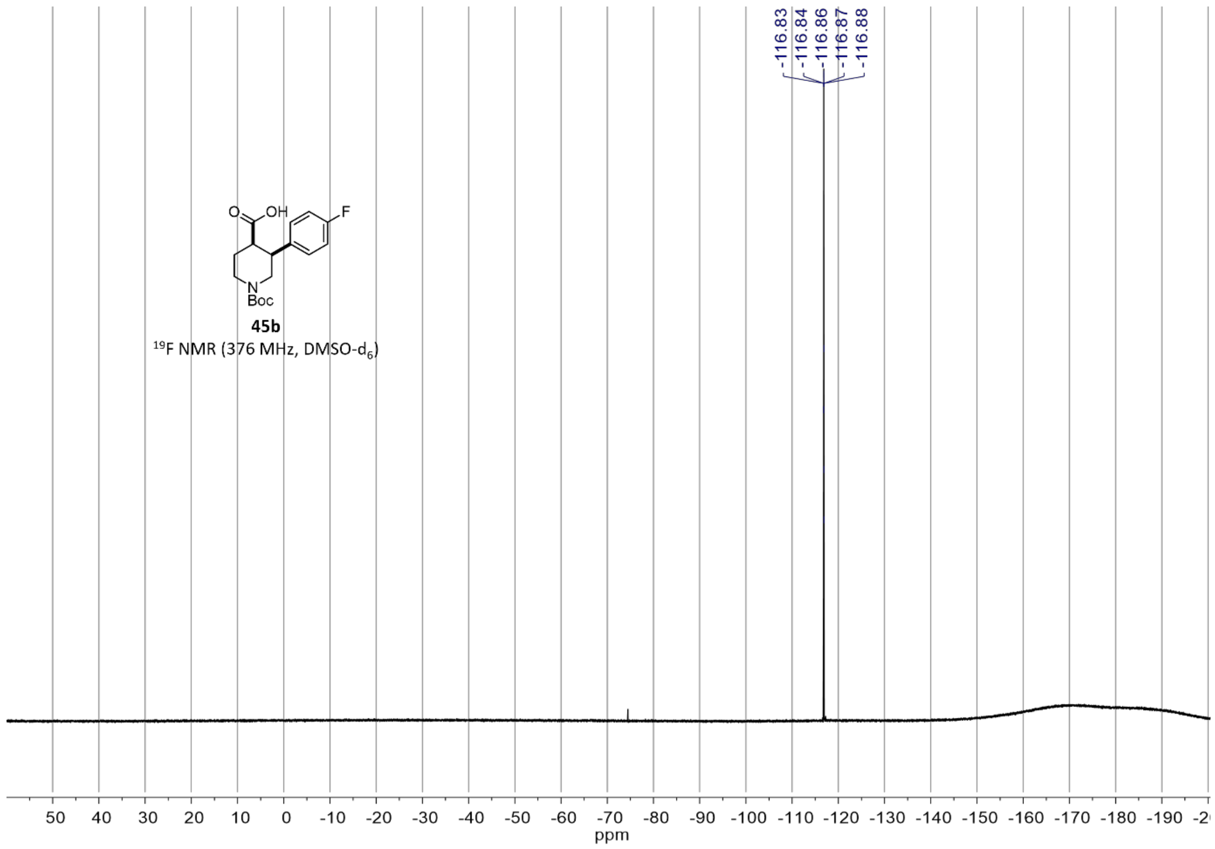
-116.83
-116.84
-116.86
-116.87
-116.88
O
OH
F
N
Boc
45b
19F NMR (376 MHz, DMSO-d6)
50 40 30 20 10 0 -10 -20 -30 -40 -50 -60 -70 -80 -90 -100 -110 -120 -130 -140 -150 -160 -170 -180 -190
ppm

trans-(±)-1-(tert-Butoxycarbonyl)-3-(4-fluorophenyl)piperidine-4-carboxylic acid (46b)

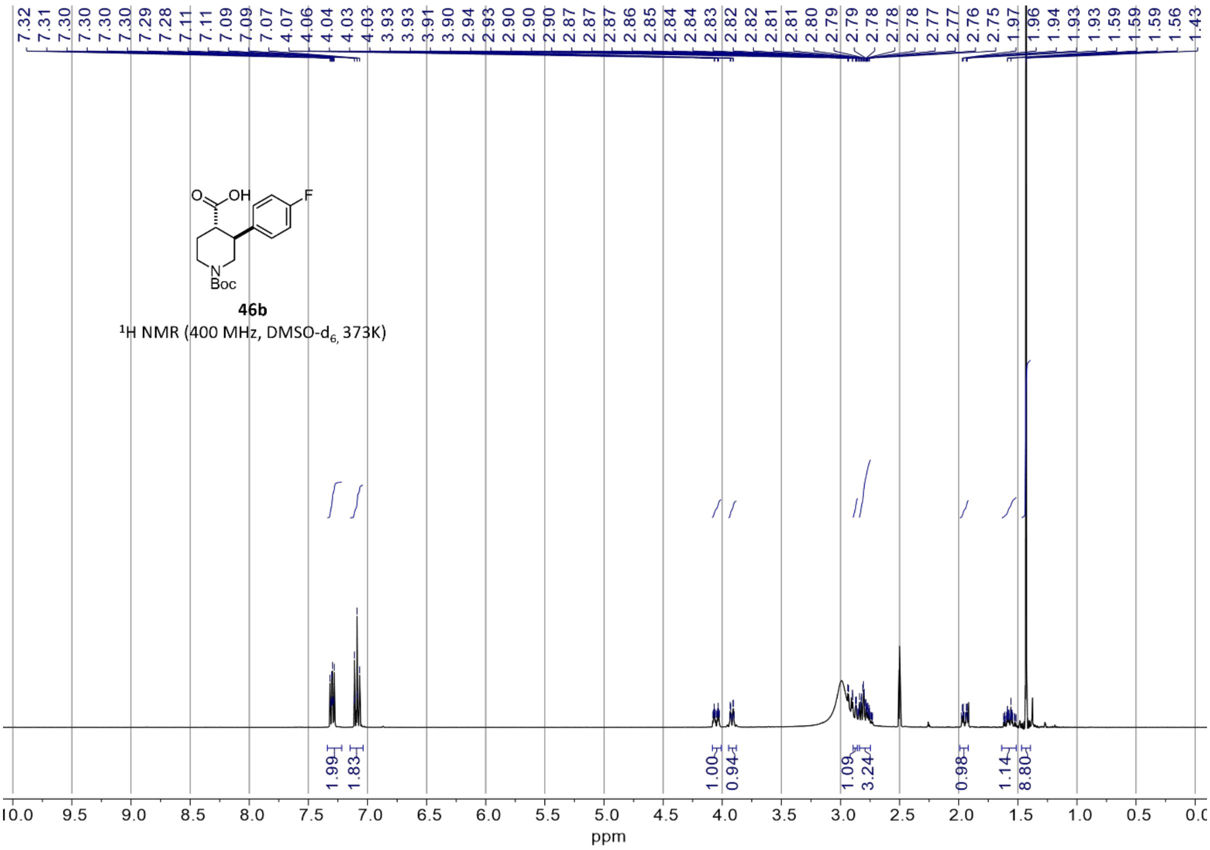

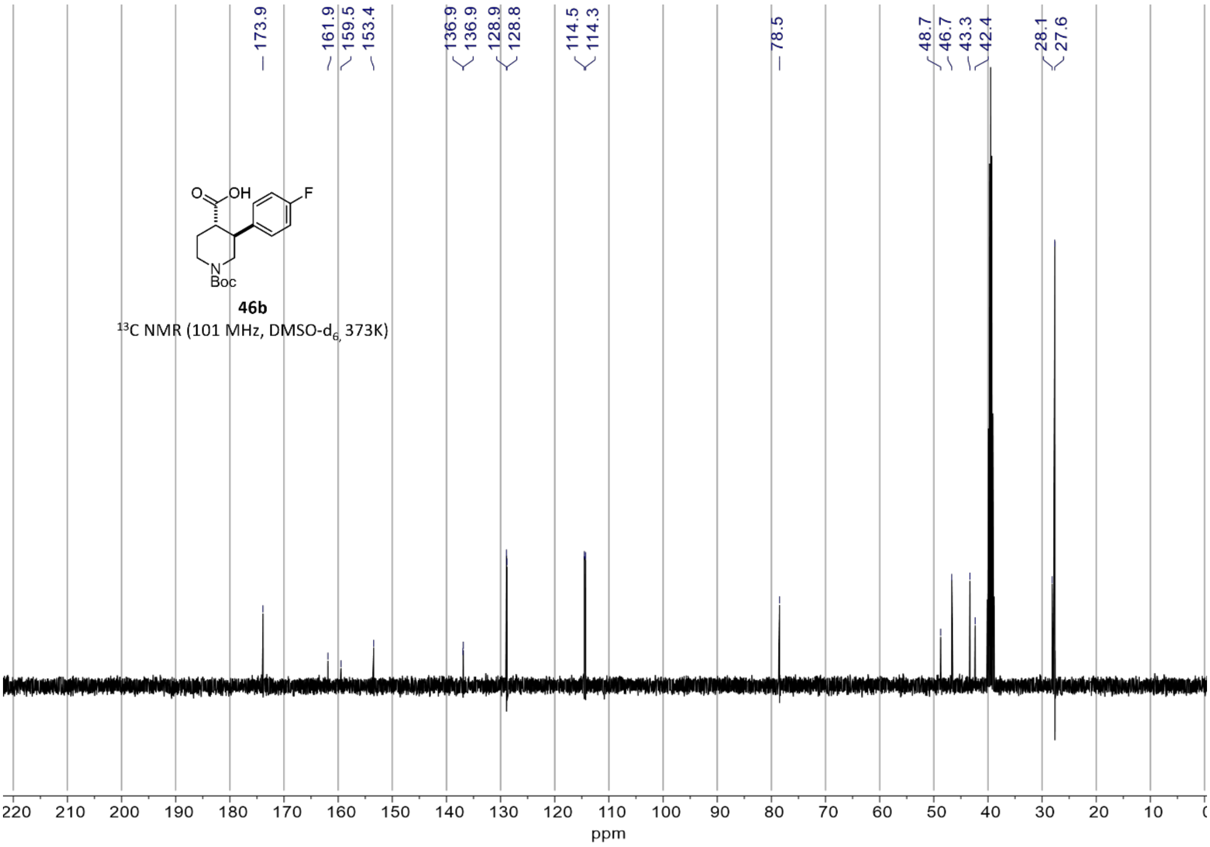

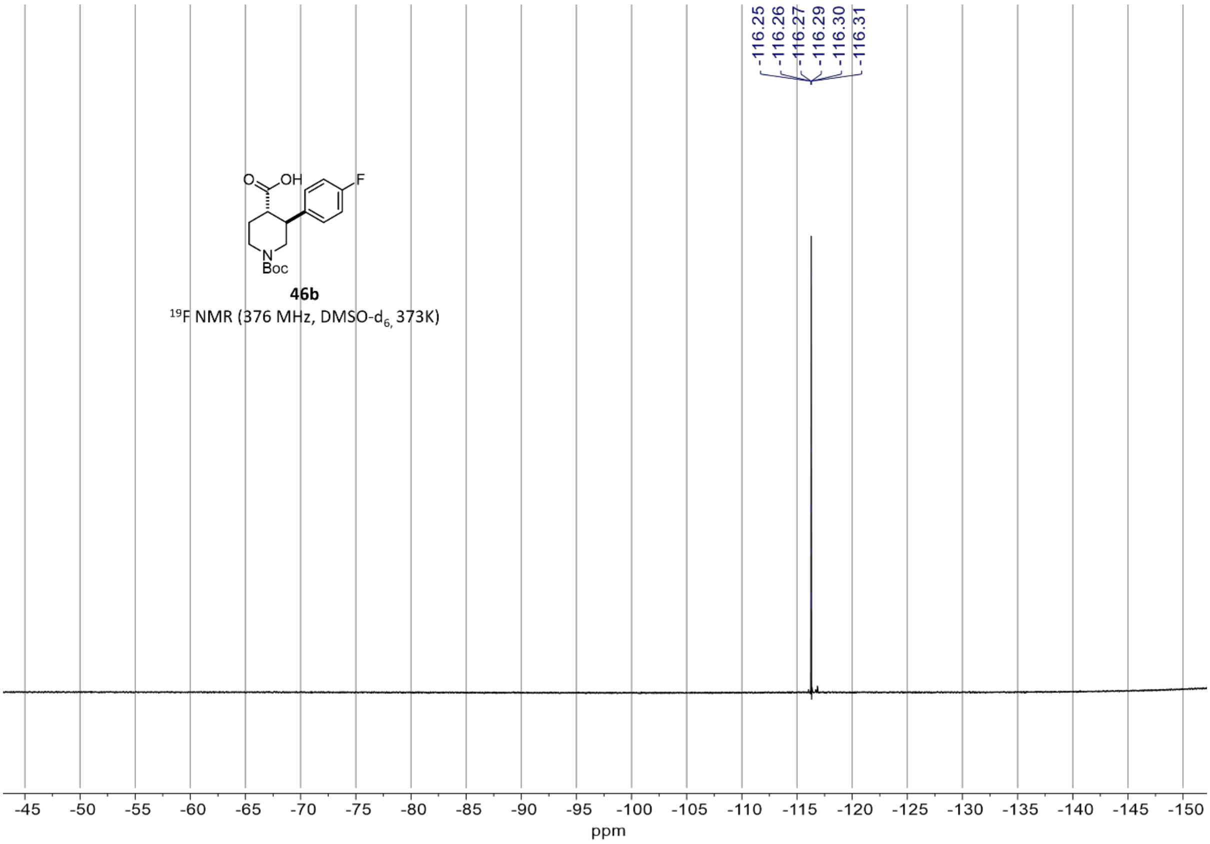
-116.25
-116.26
-116.27
-116.29
-116.30
-116.31
O
OH
F
N
Boc
46b
19F NMR (376 MHz, DMSO-d6, 373K)
-45 -50 -55 -60 -65 -70 -75 -80 -85 -90 -95 -100 -105 -110 -115 -120 -125 -130 -135 -140 -145 -150
ppm

***tert*-Butyl-*cis*-(±)-4-carbamoyl-3-(4-methoxyphenyl)piperidine-1-carboxylate (49a)**

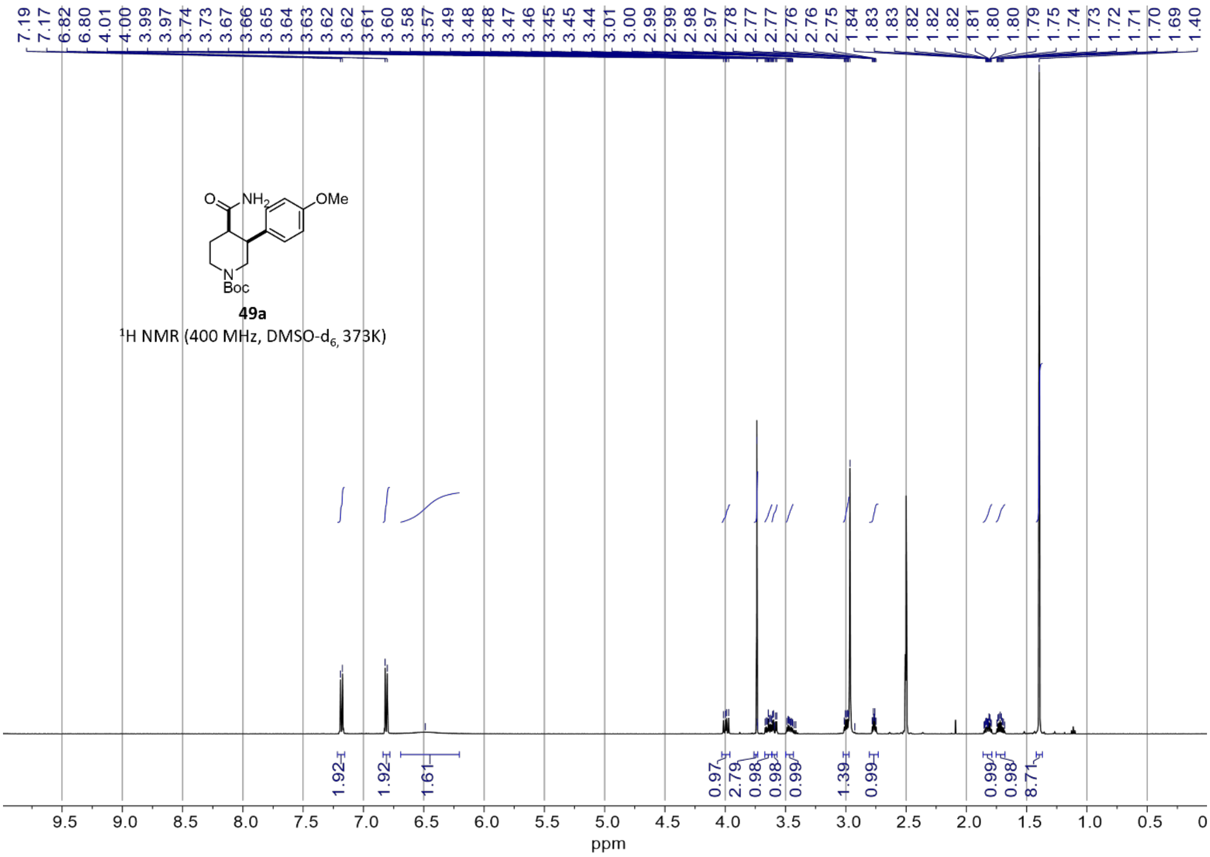

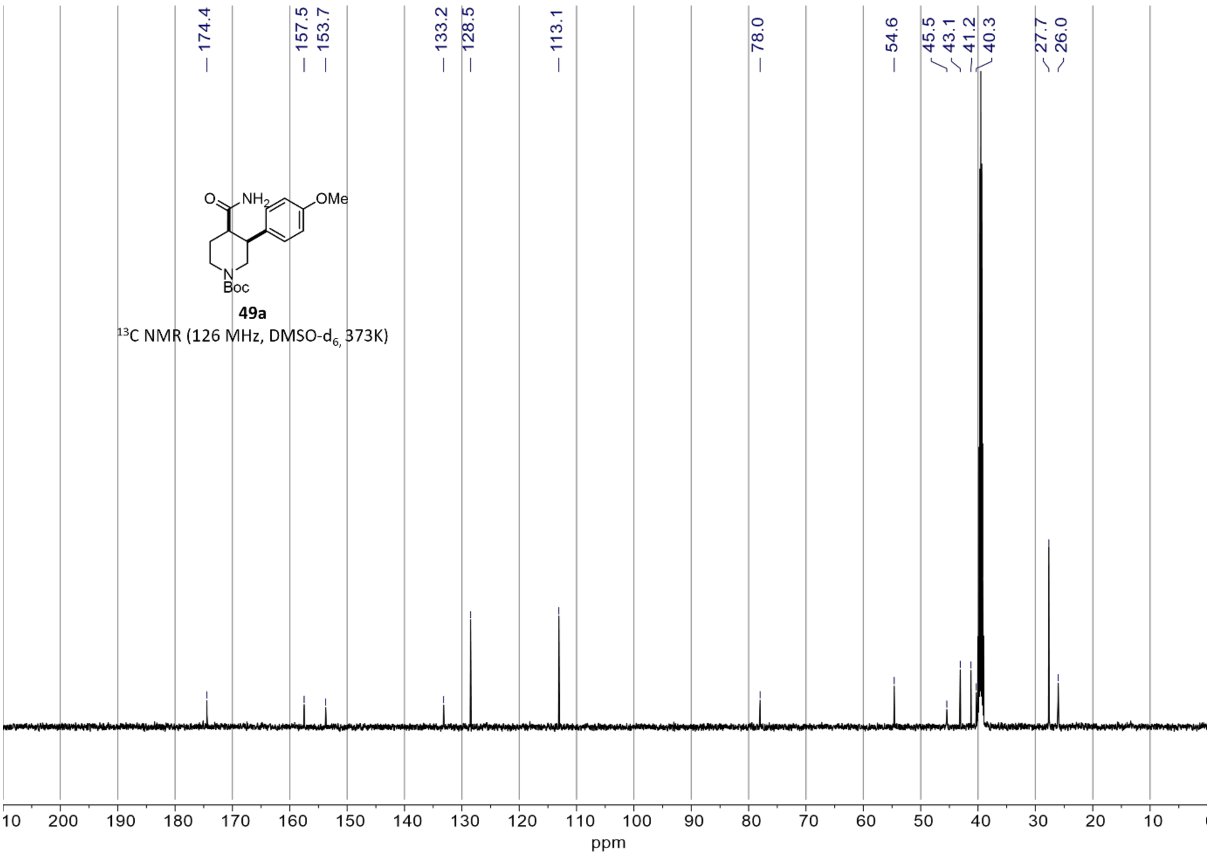

tert-Butyl-_trans_-(±)-4-carbamoyl-3-(4-methoxyphenyl)piperidine-1-carboxylate (50a)

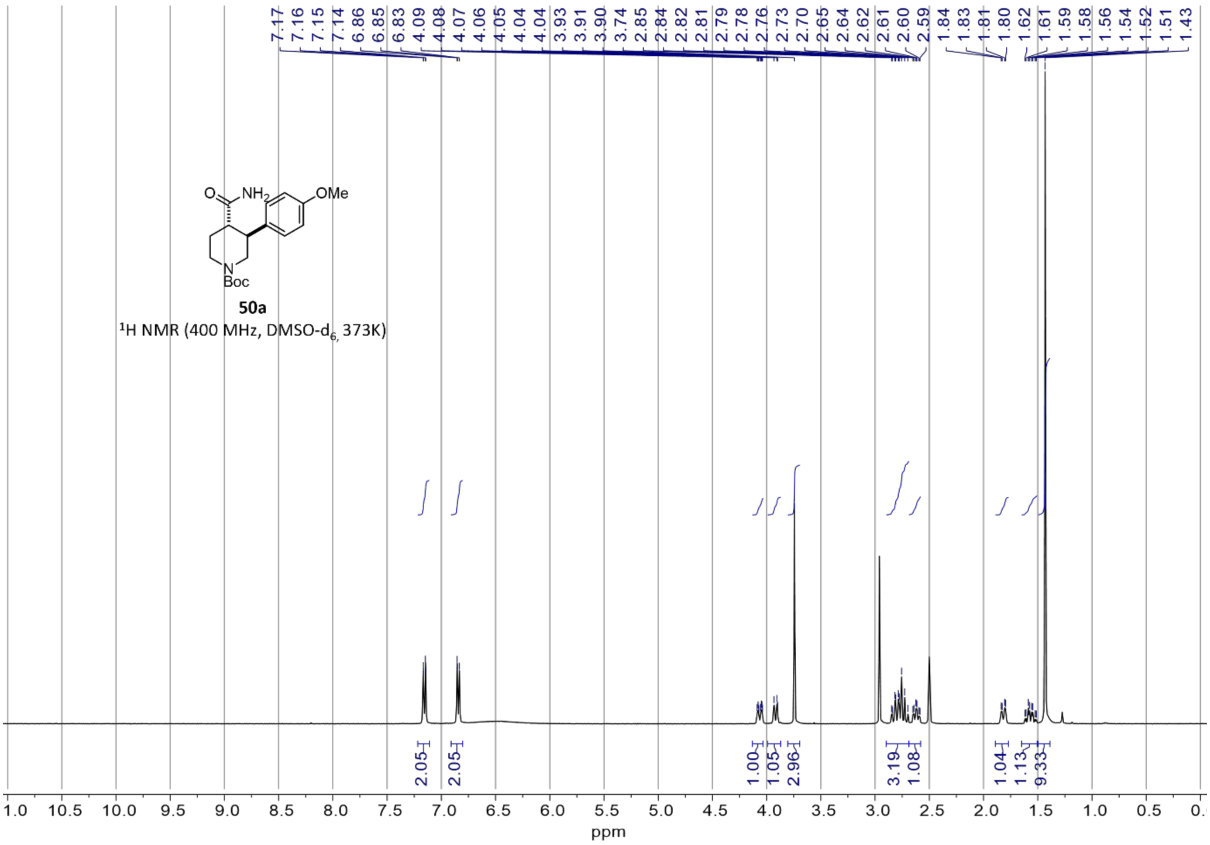

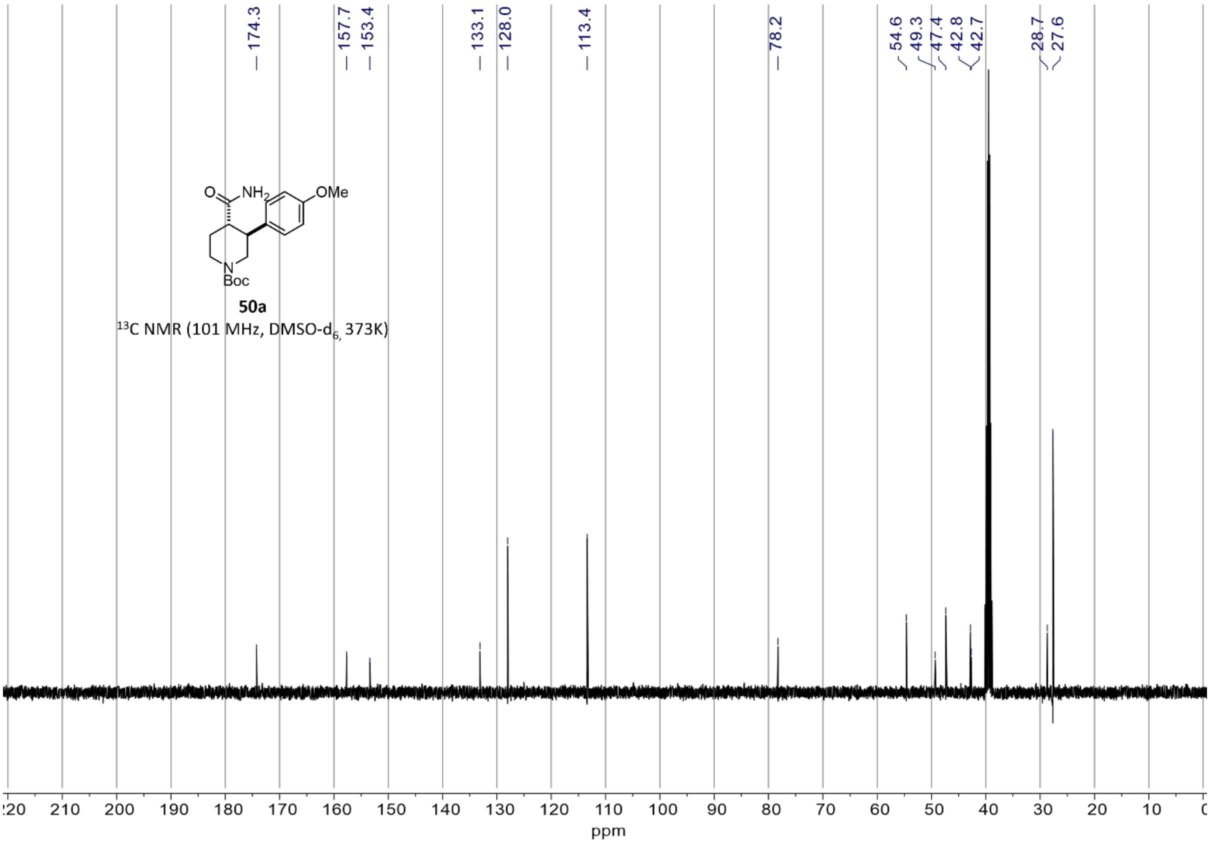

tert-Butyl-cis-(±)-4-carbamoyl-3-(4-fluorophenyl)piperidine-1-carboxylate (49b)

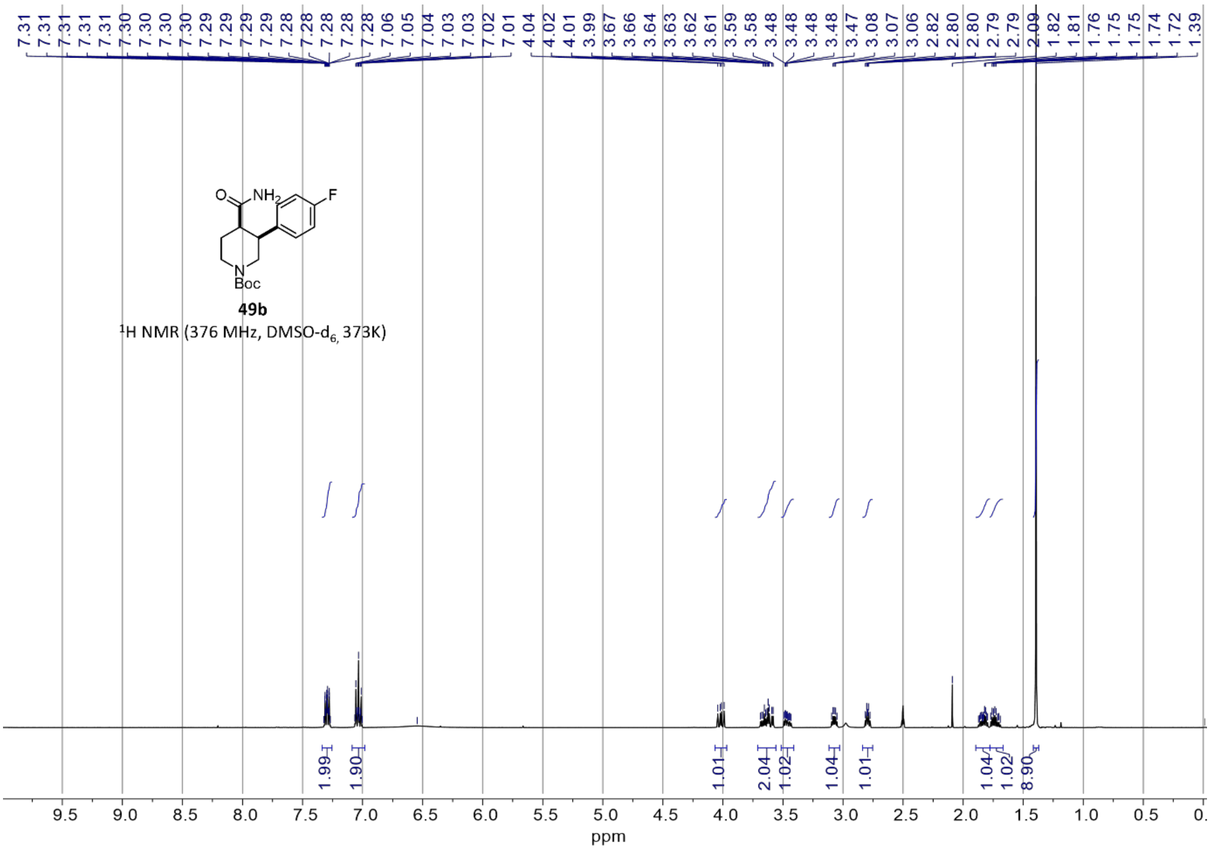

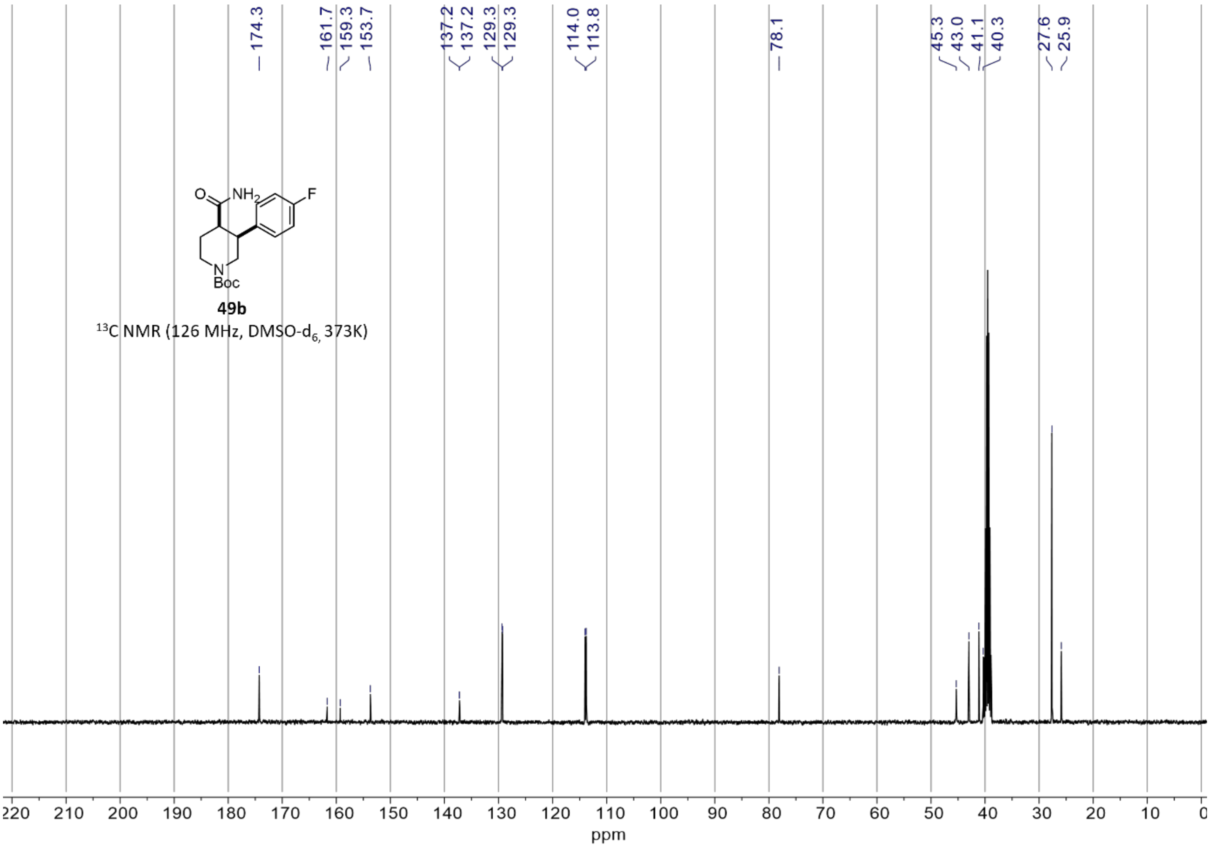

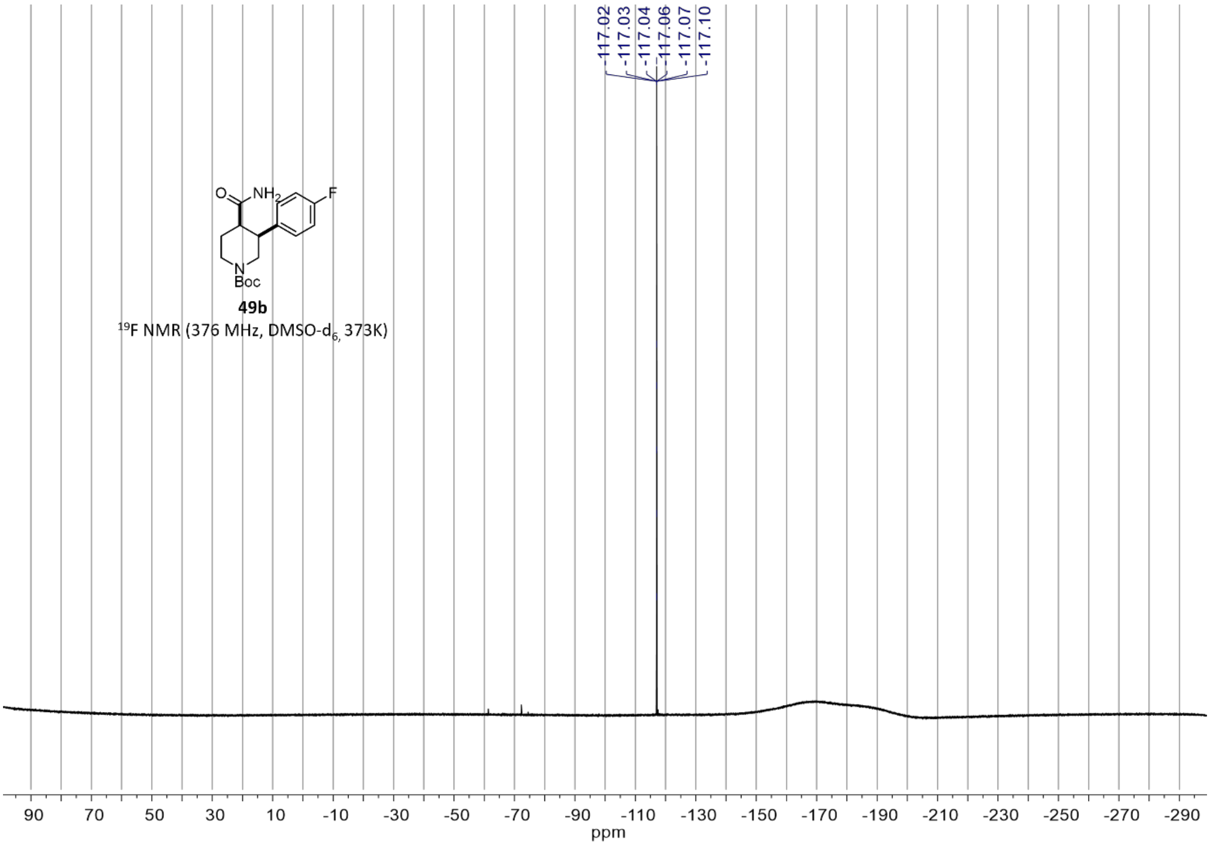

49b

^{19}F NMR (376 MHz, DMSO-d_6, 373K)

***tert*-Butyl-*trans*-(±)-4-carbamoyl-3-(4-fluorophenyl)piperidine-1-carboxylate (50b)**

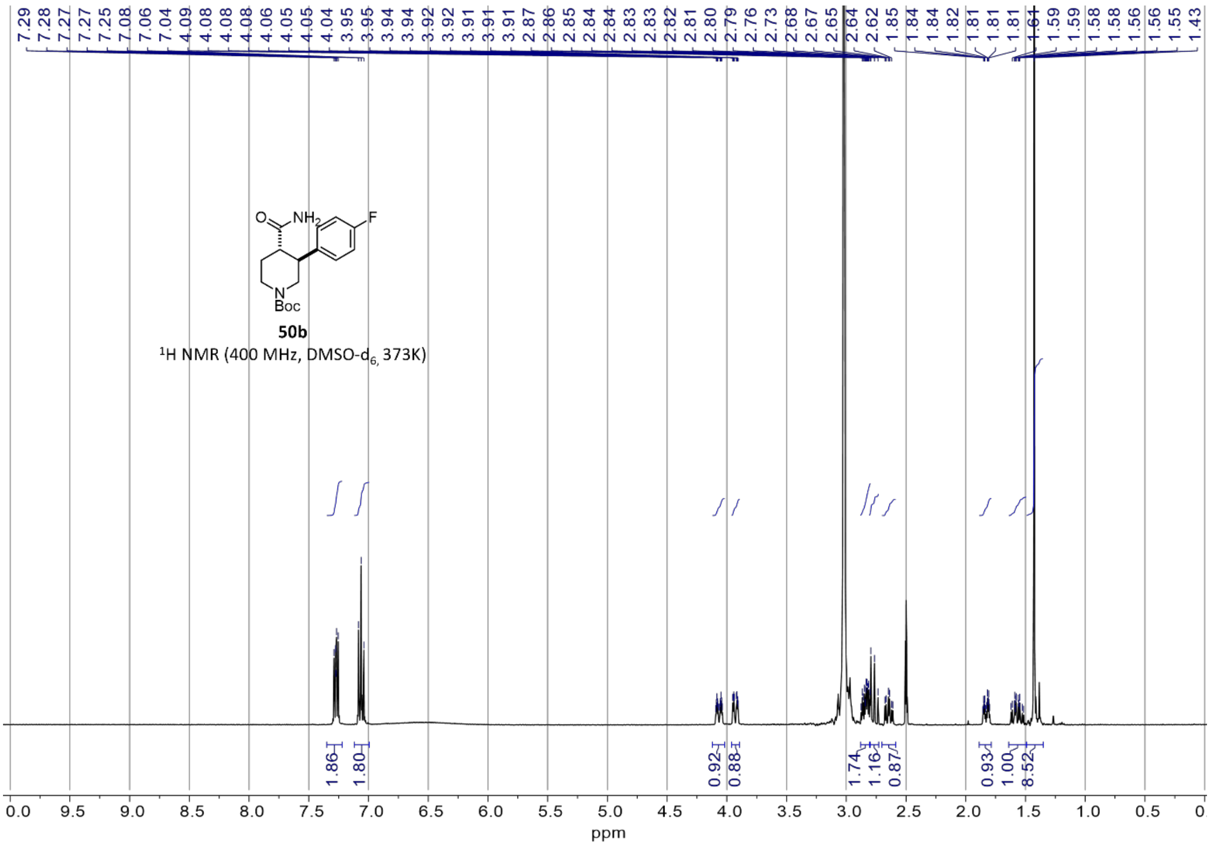

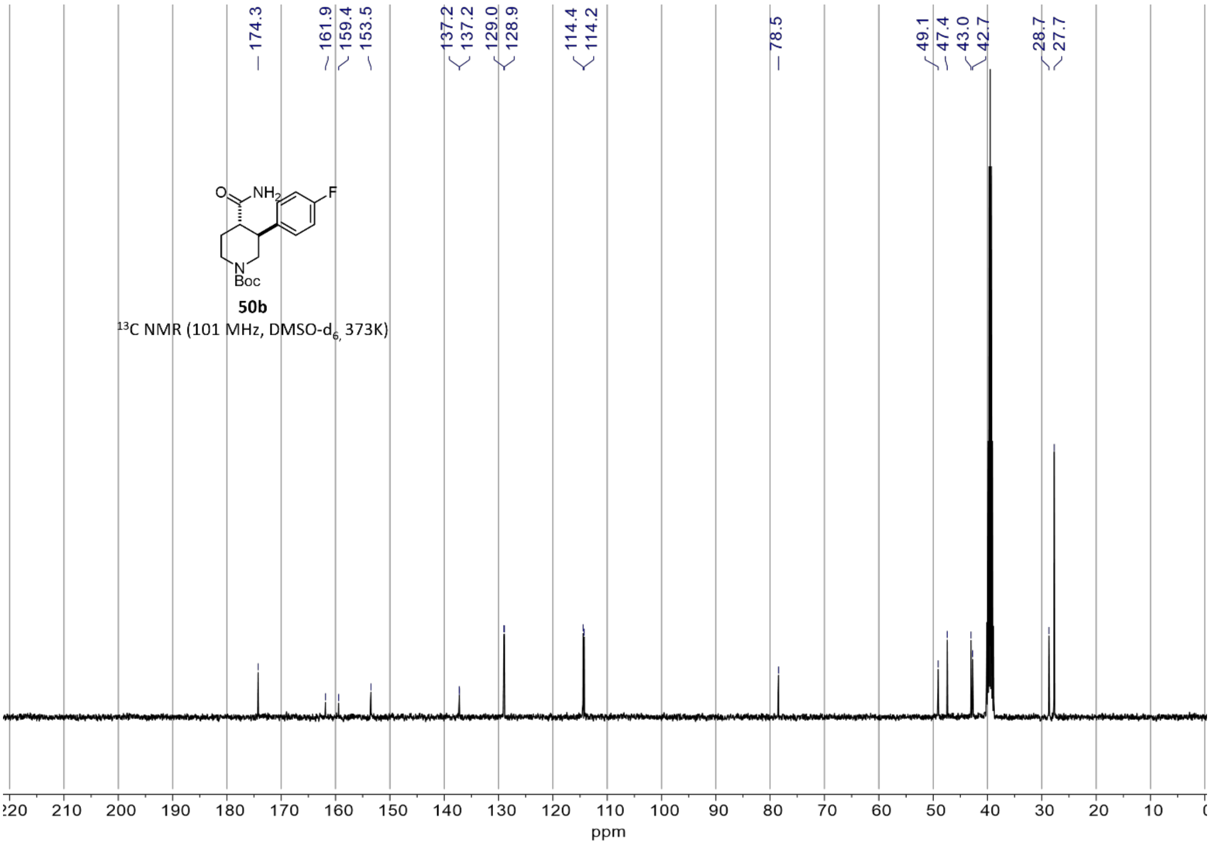

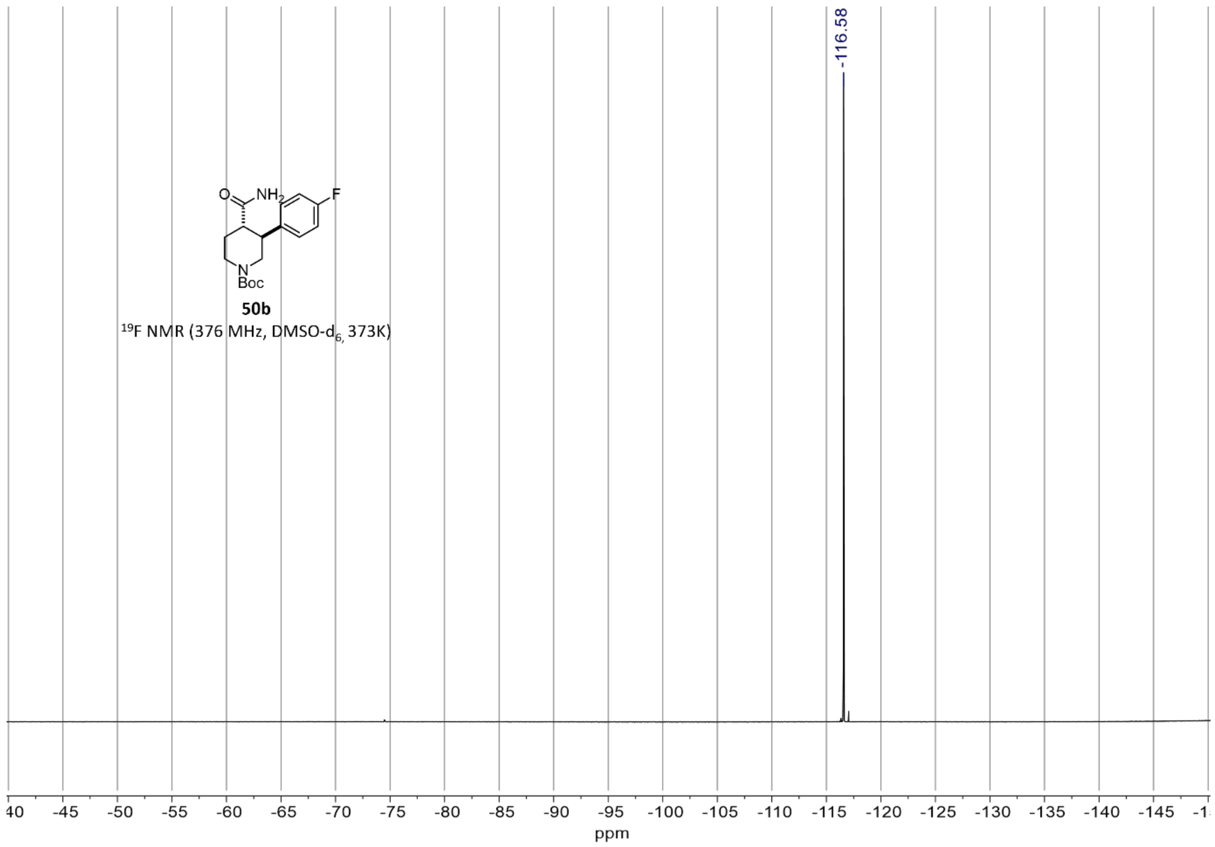

-116.58
O
NH2
F
N
Boc
50b
19F NMR (376 MHz, DMSO-d6, 373K)
40
-45
-50
-55
-60
-65
-70
-75
-80
-85
-90
-95
-100
-105
-110
-115
-120
-125
-130
-135
-140
-145
ppm

***tert*-Butyl-*cis*-(±)-4-carbamoyl-3-(4-methoxyphenyl)piperidine-1-carboxylate (51a)**

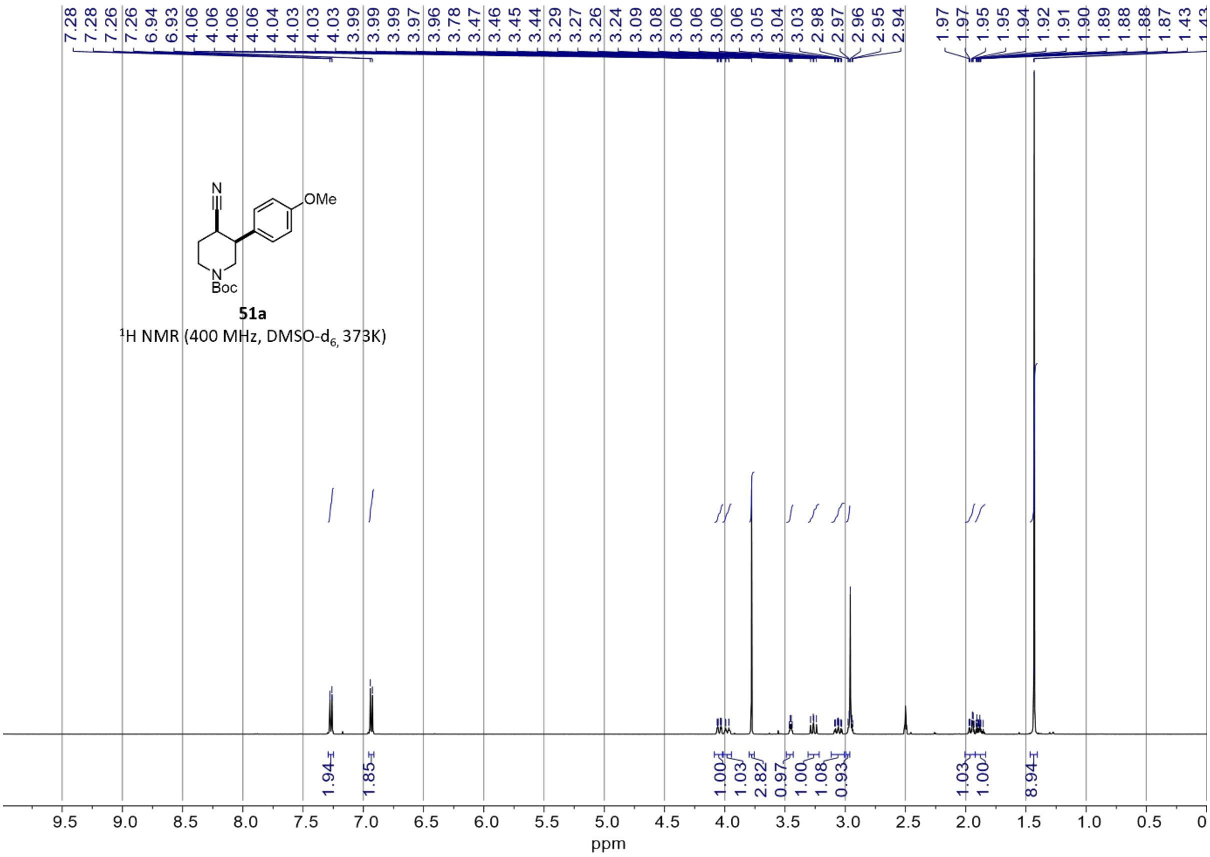

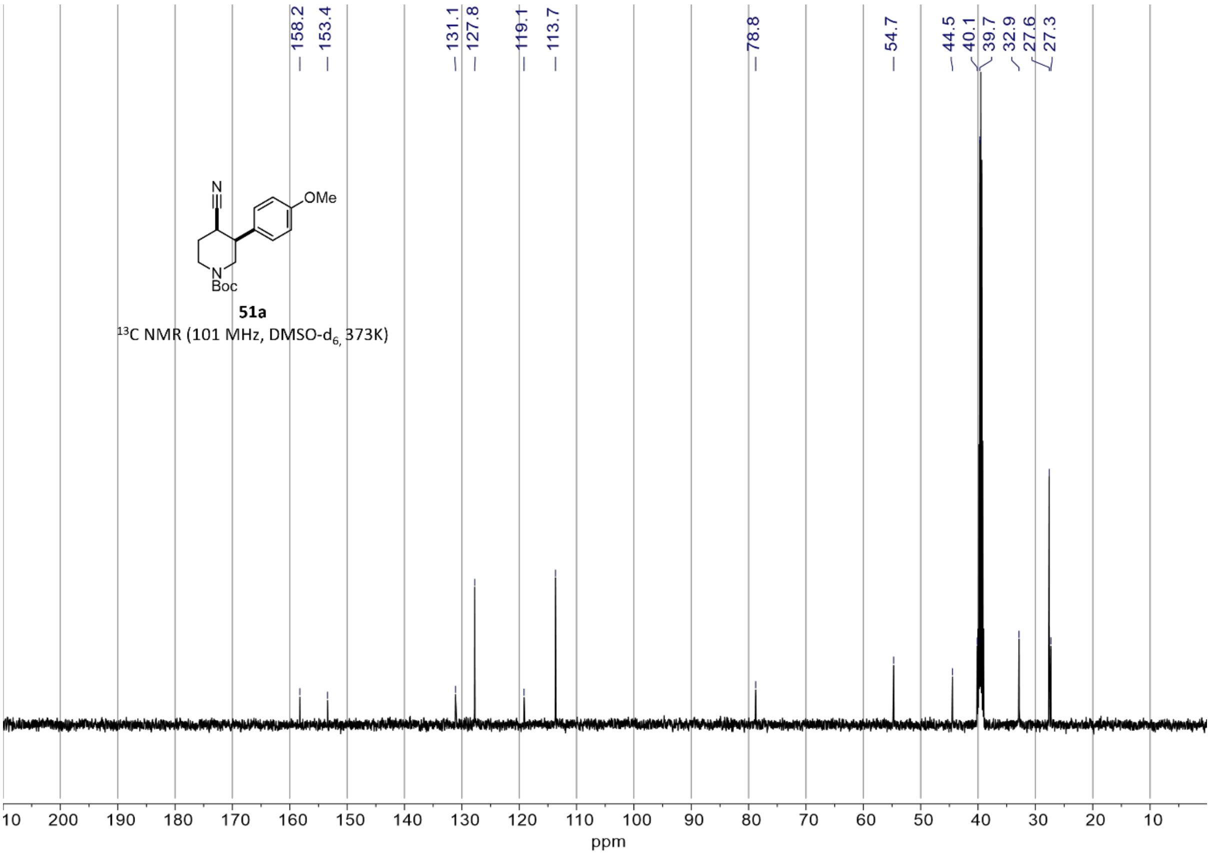

tert-Butyl-_cis_-(±)-4-cyano-3-(4-fluorophenyl)piperidine-1-carboxylate (51b)

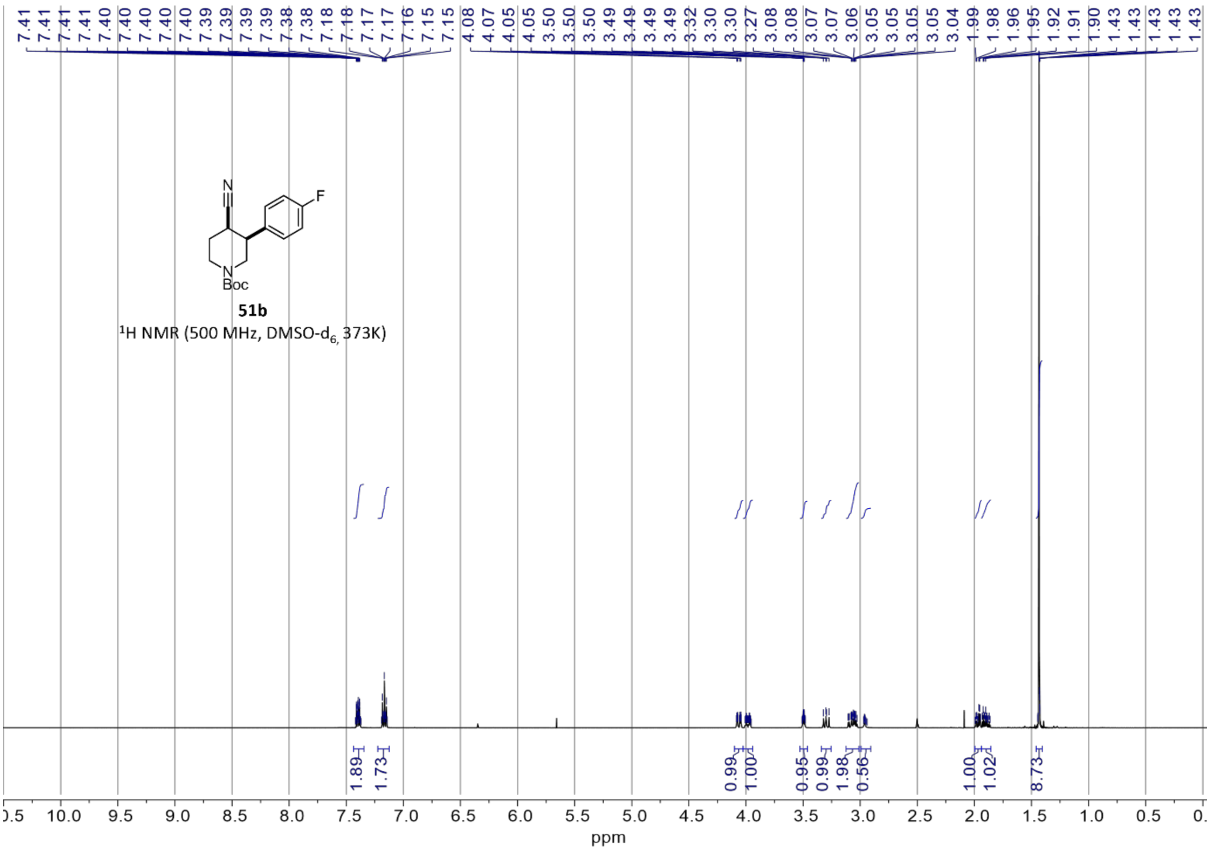

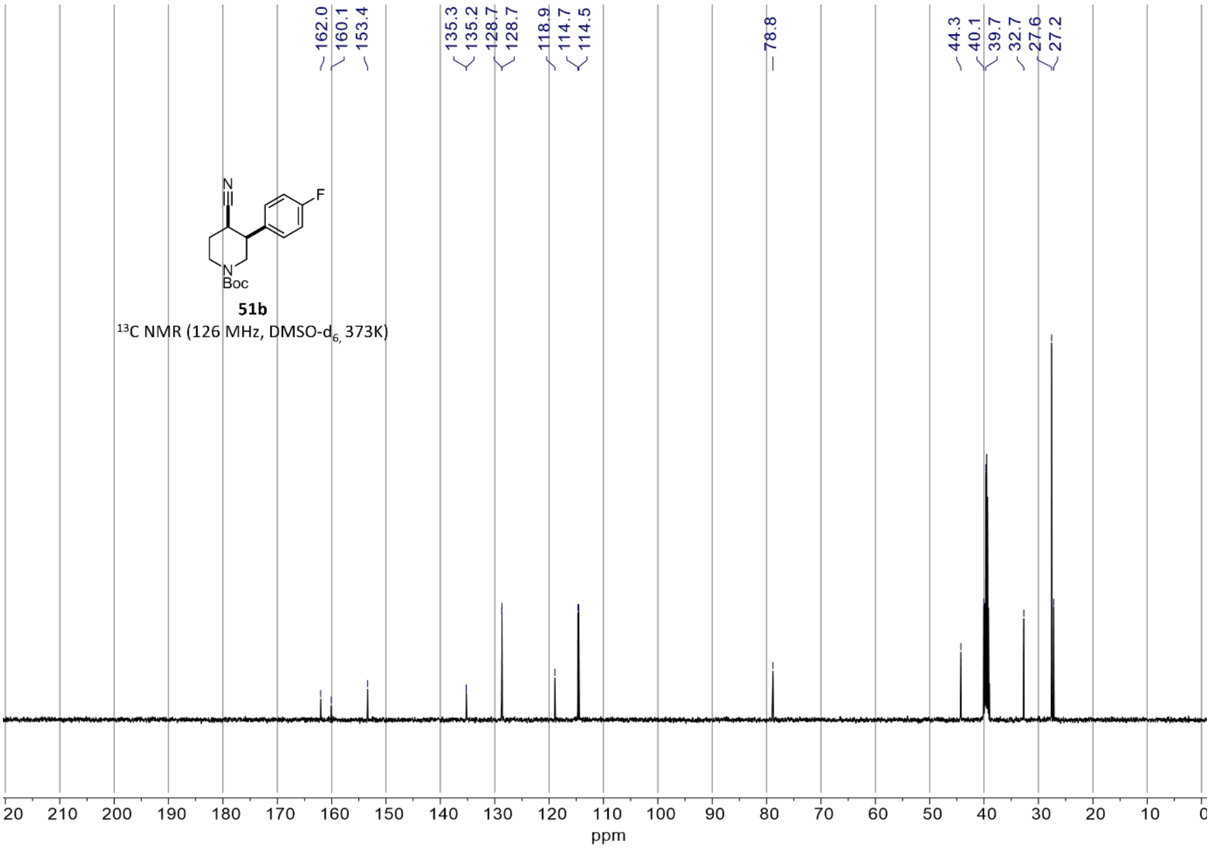

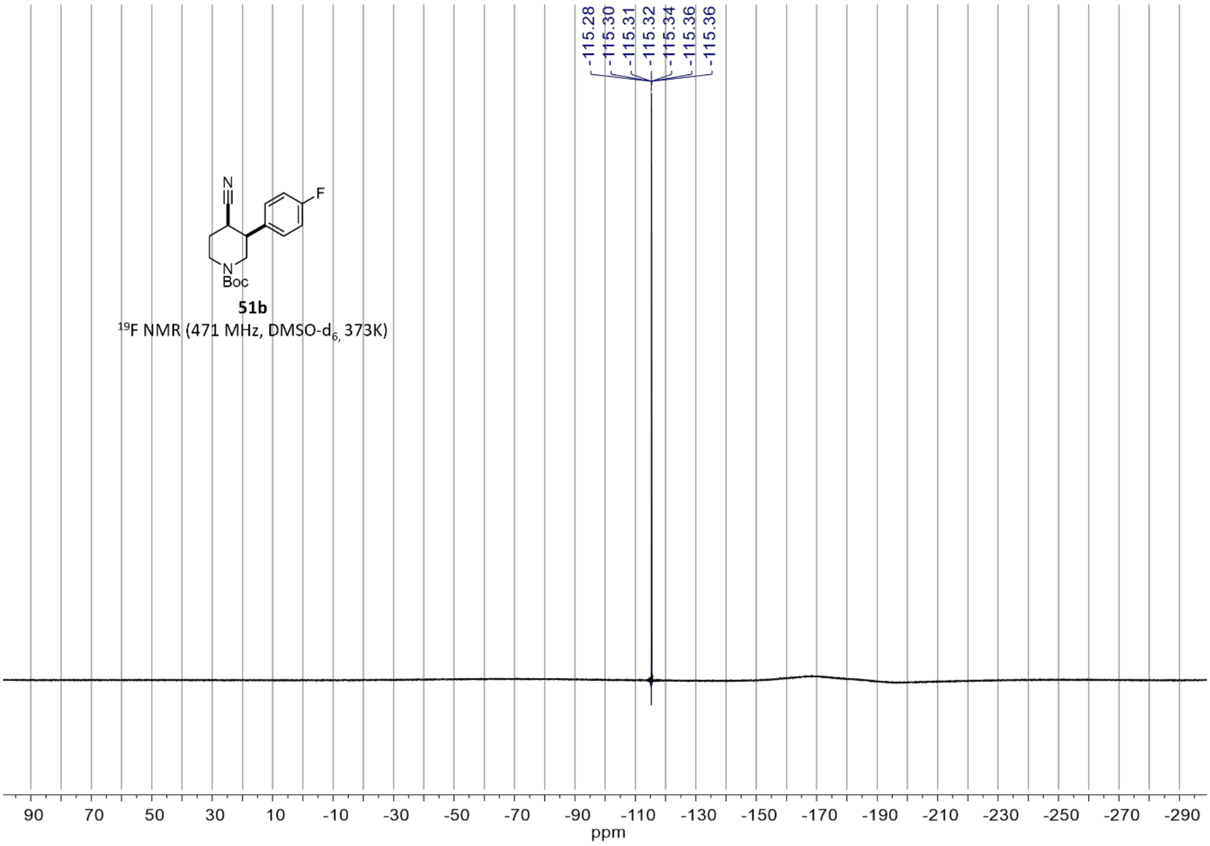

51b

^{19}F NMR (471 MHz, DMSO-d$_6$, 373K)

tert-Butyl-_cis_-(±)-4-(hydroxymethyl)-3-(4-methoxyphenyl)piperidine-1-carboxylate (47a)

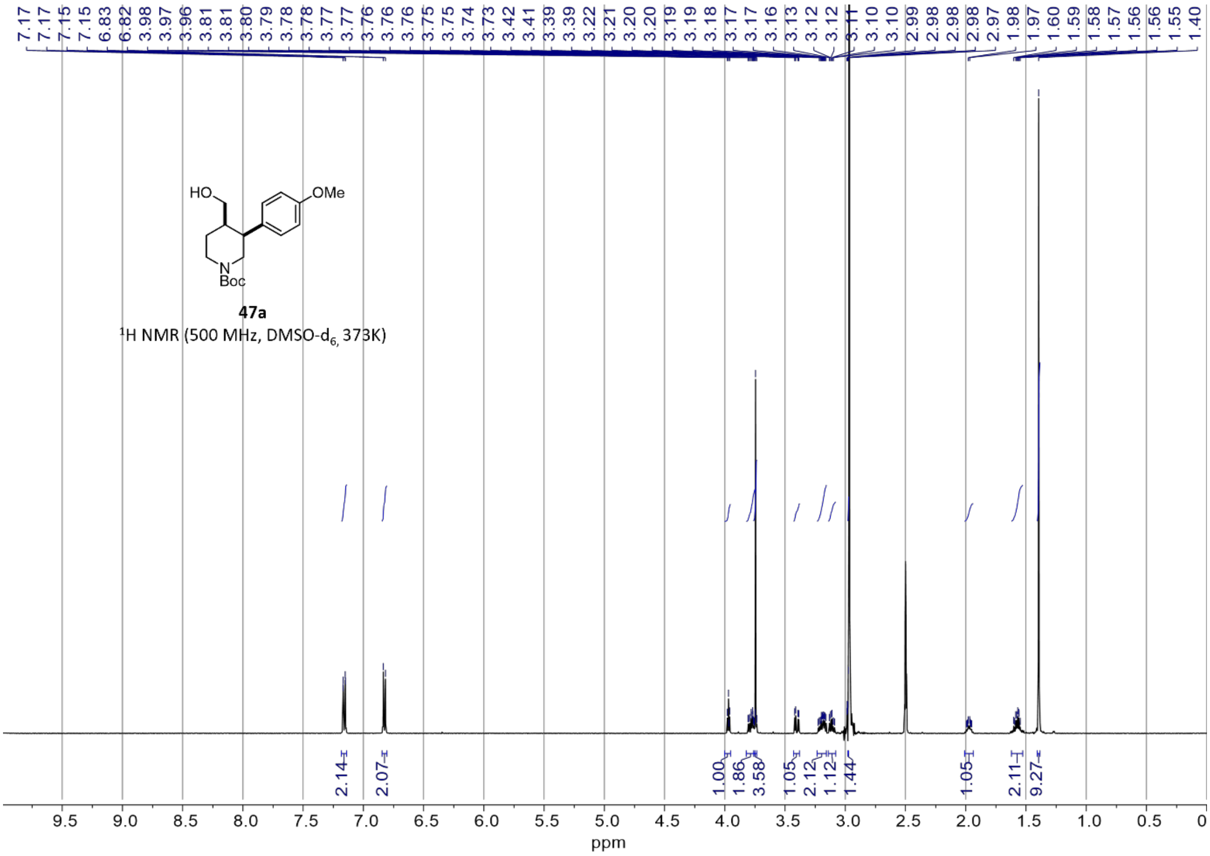

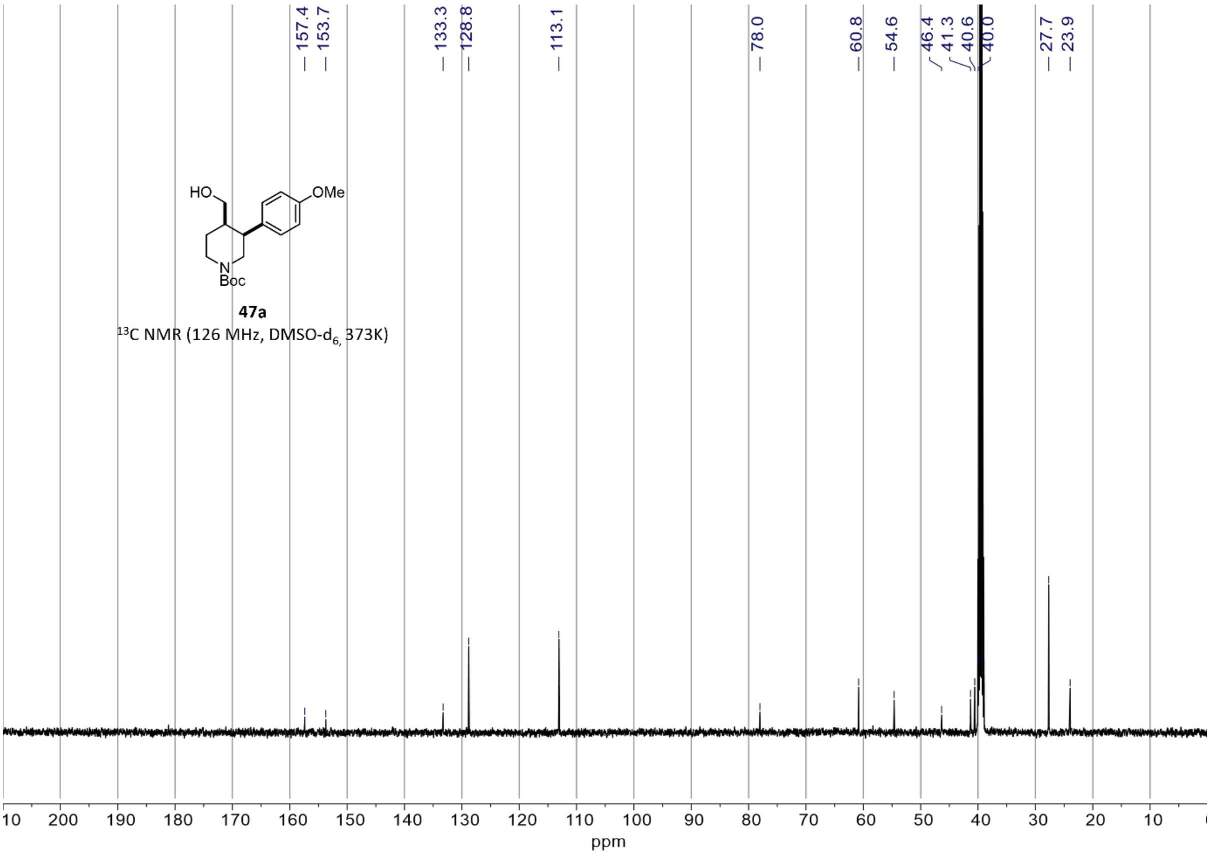

tert-Butyl-_trans_-(±)-4-(hydroxymethyl)-3-(4-methoxyphenyl)piperidine-1-carboxylate (48a)

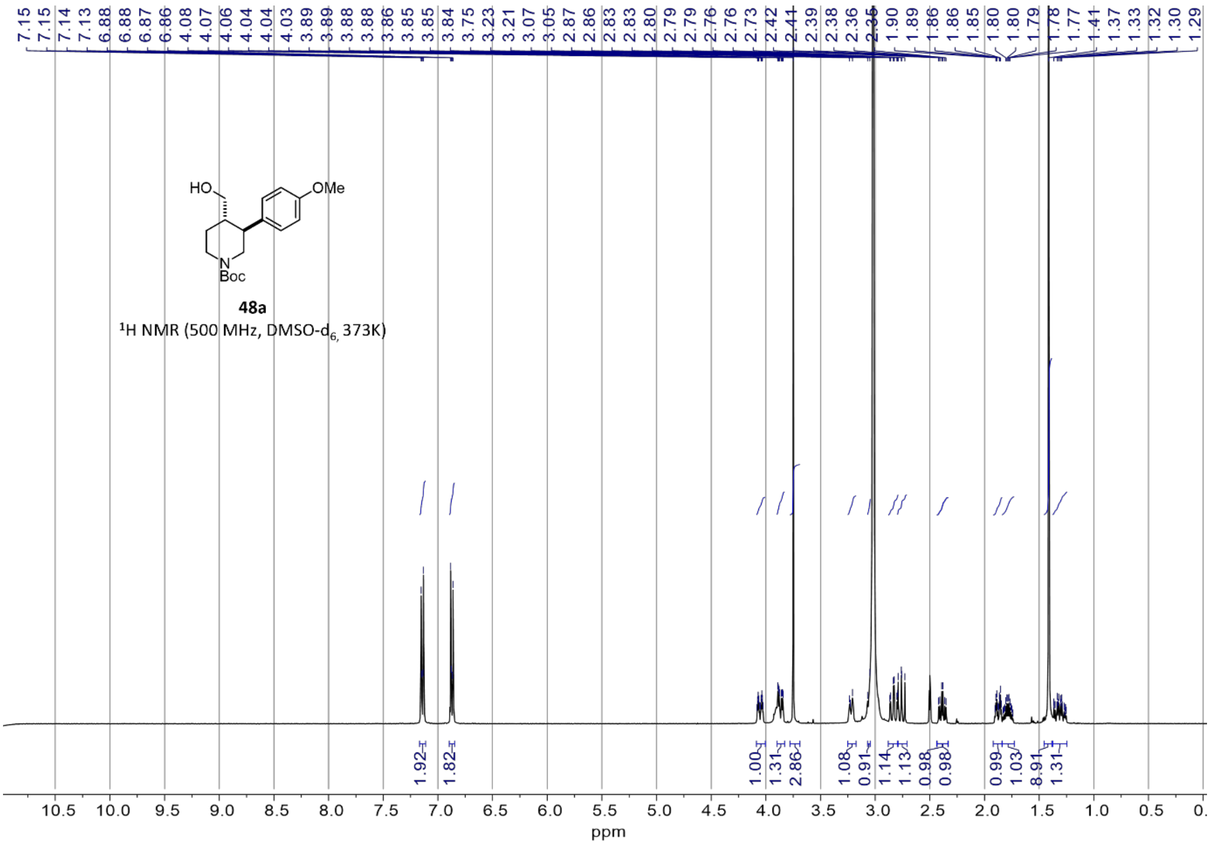

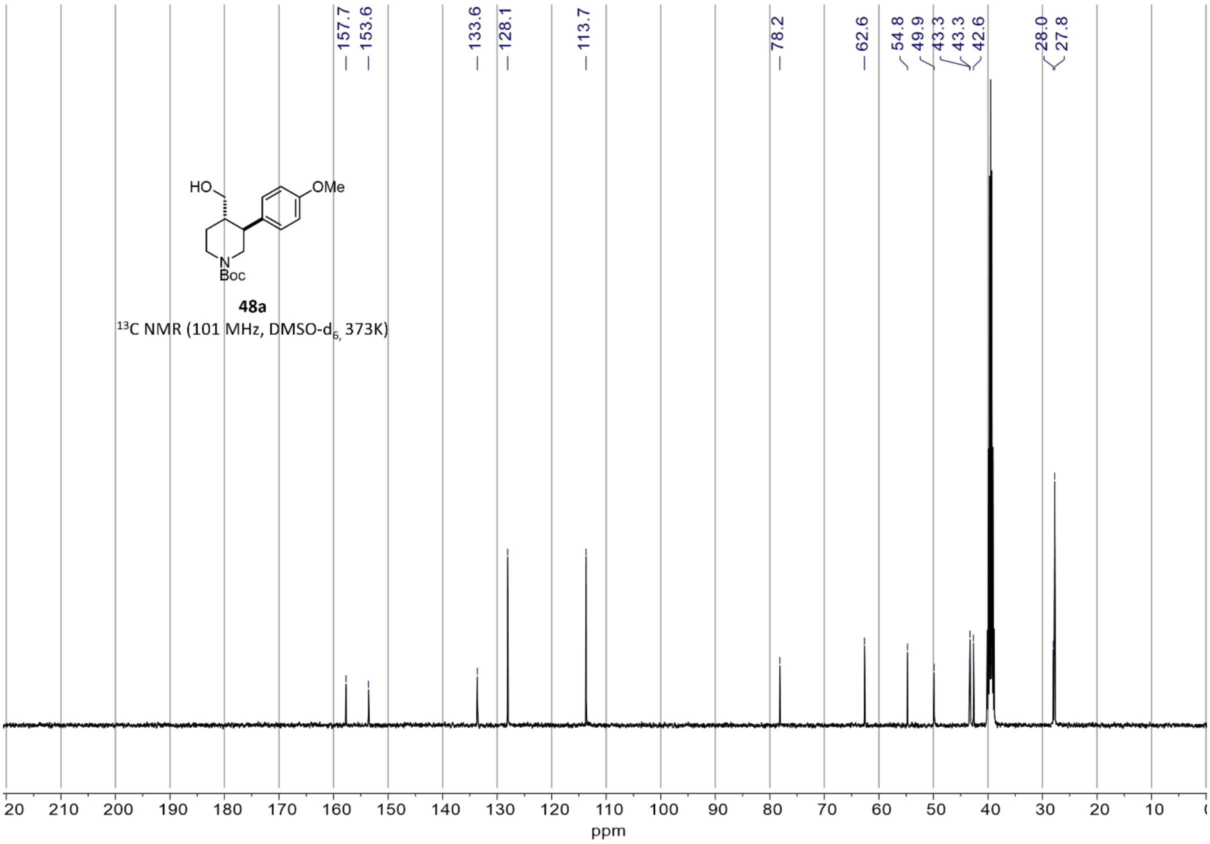

tert-Butyl-_cis_-(±)-3-(4-fluorophenyl)-4-(hydroxymethyl)piperidine-1-carboxylate (47b)

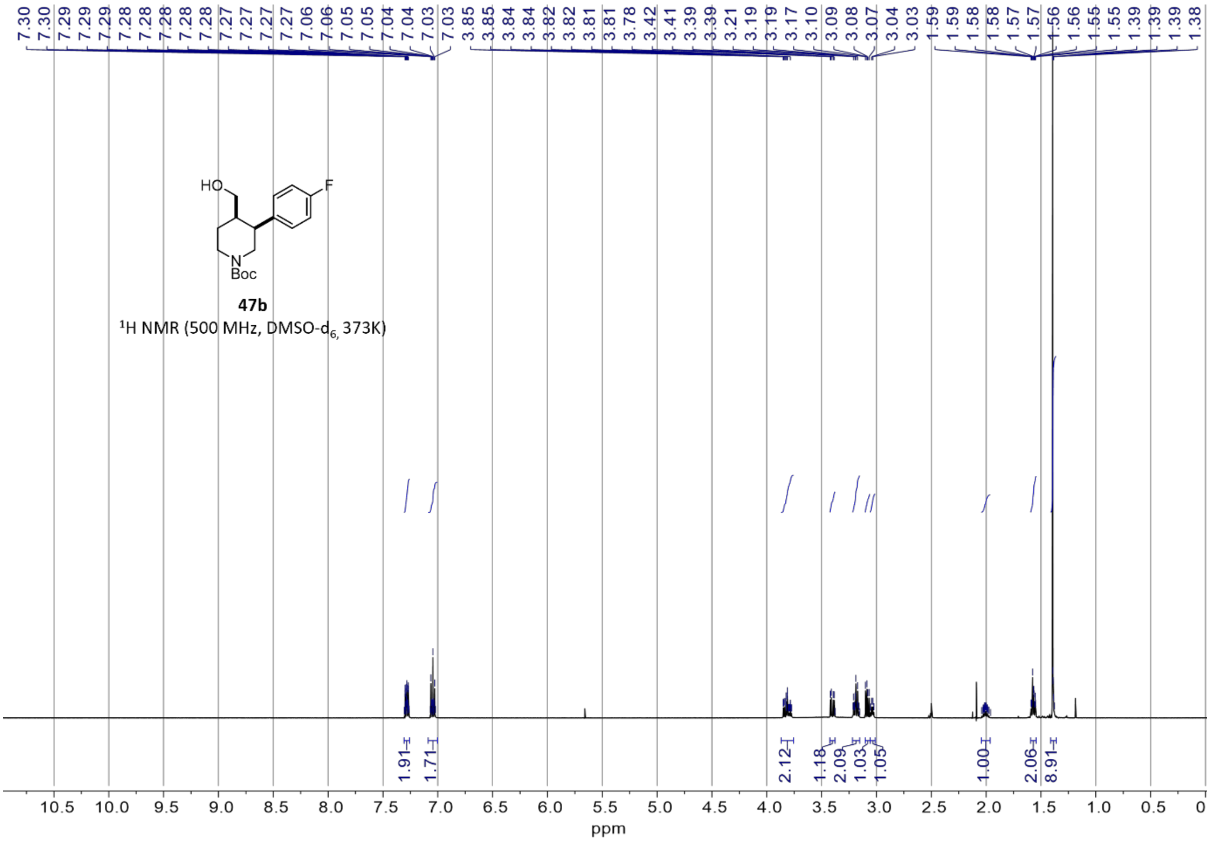

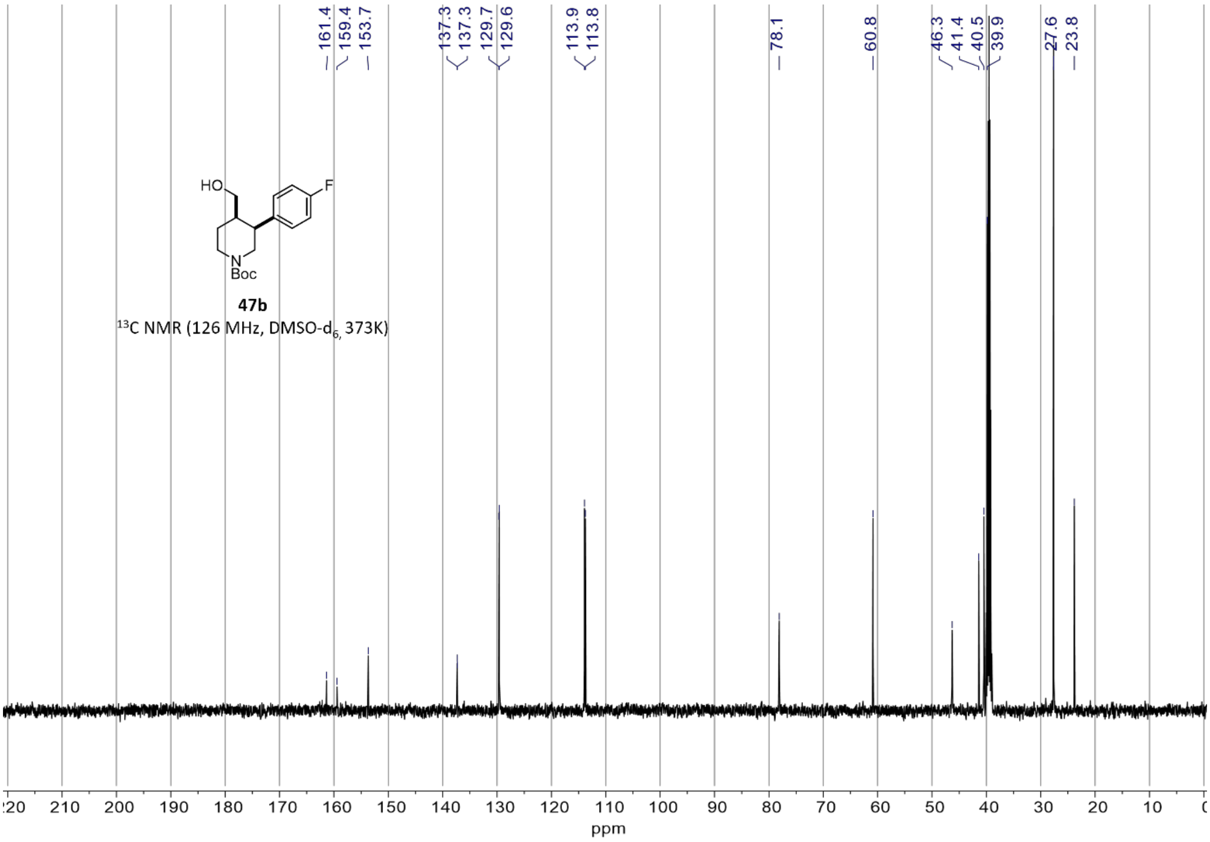

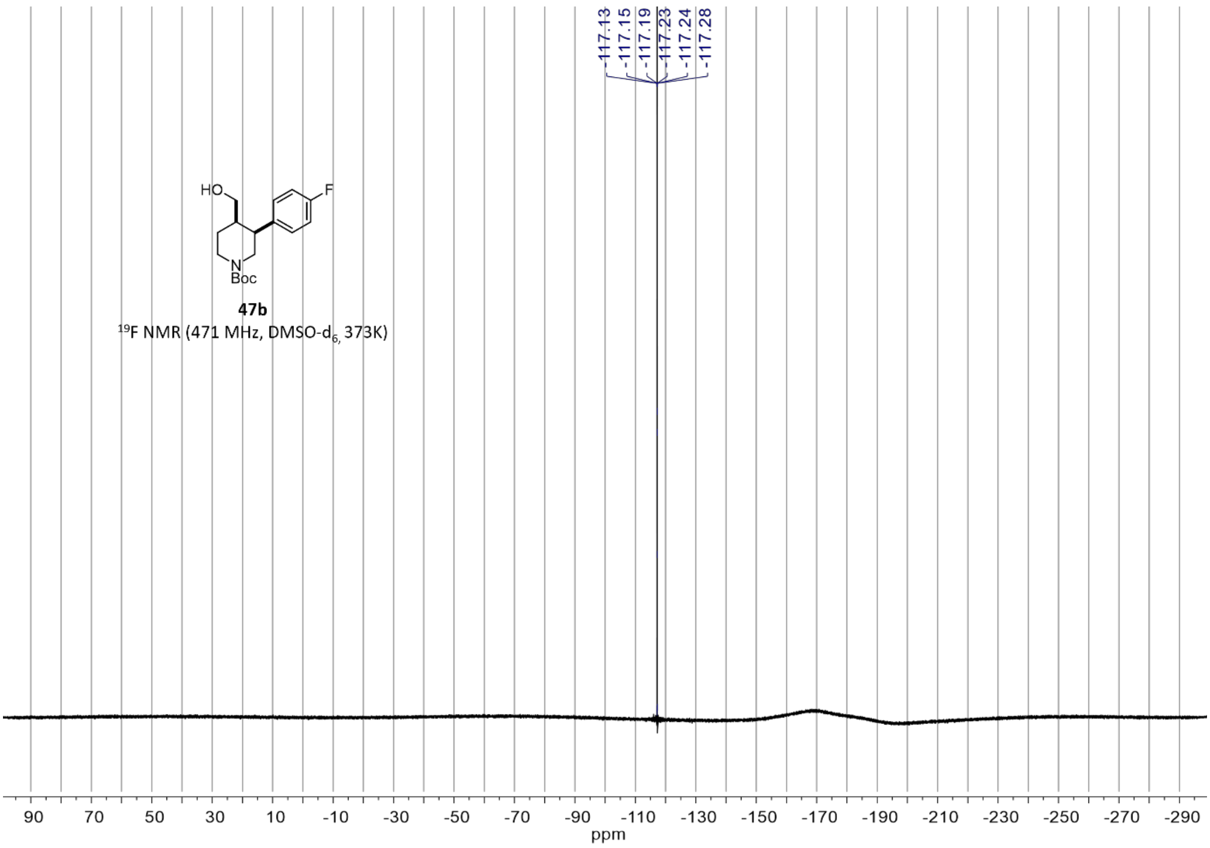

47b

^{19}F NMR (471 MHz, DMSO-d_6, 373K)

tert-Butyl-_trans_-(±)-3-(4-fluorophenyl)-4-(hydroxymethyl)piperidine-1-carboxylate (48b)

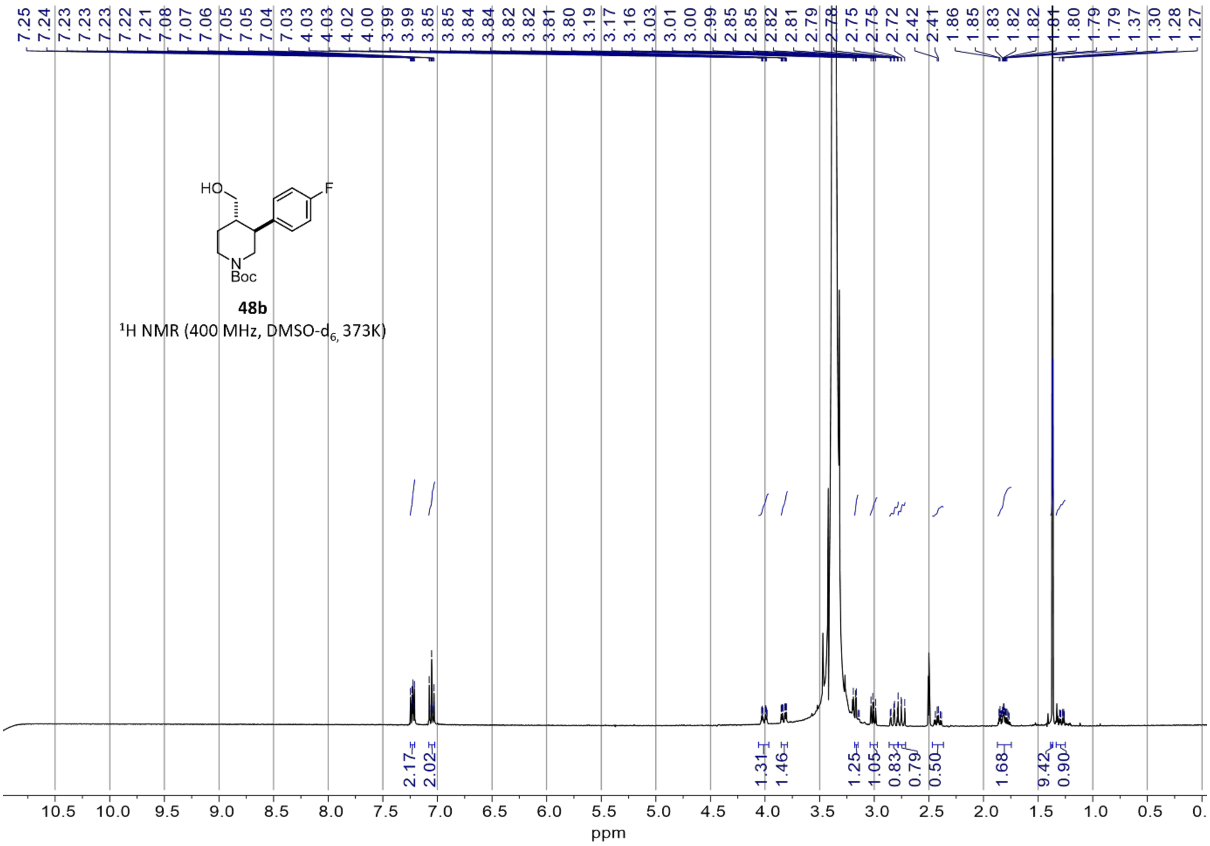

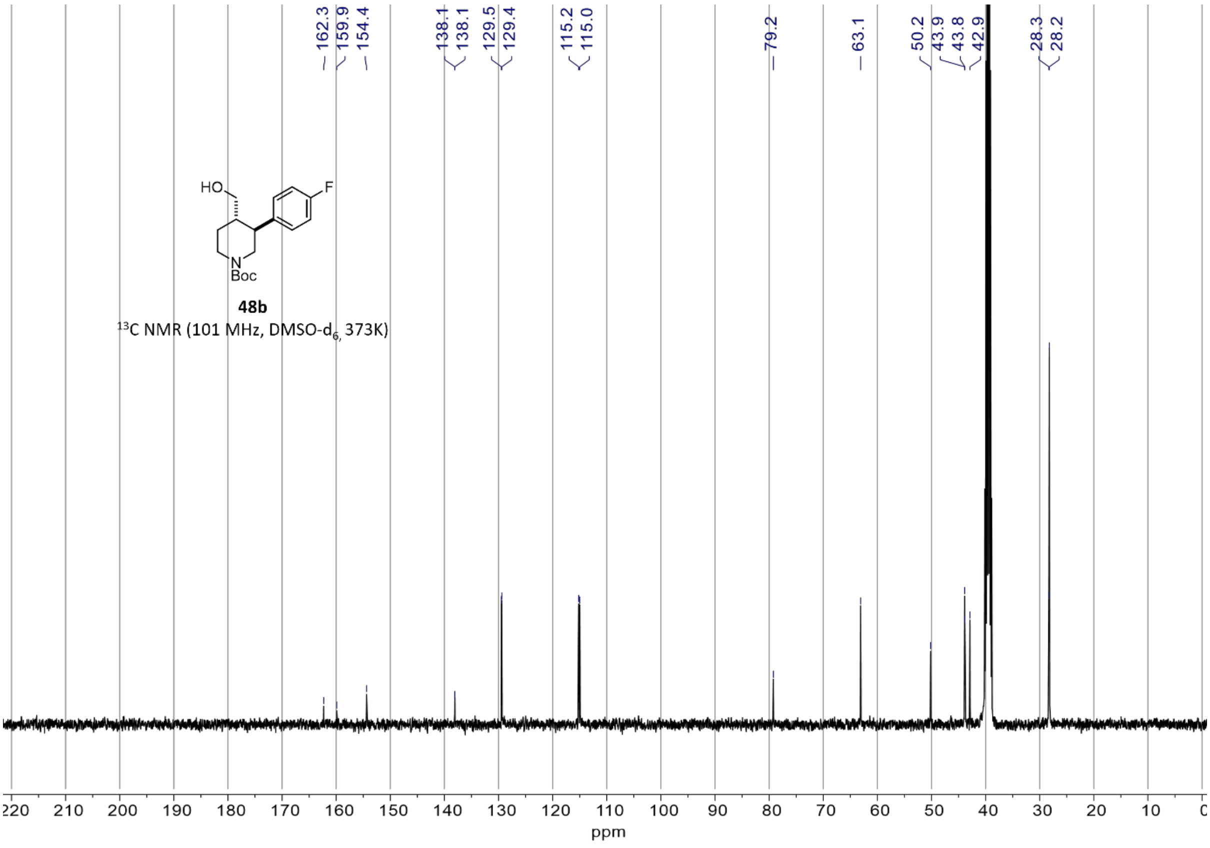

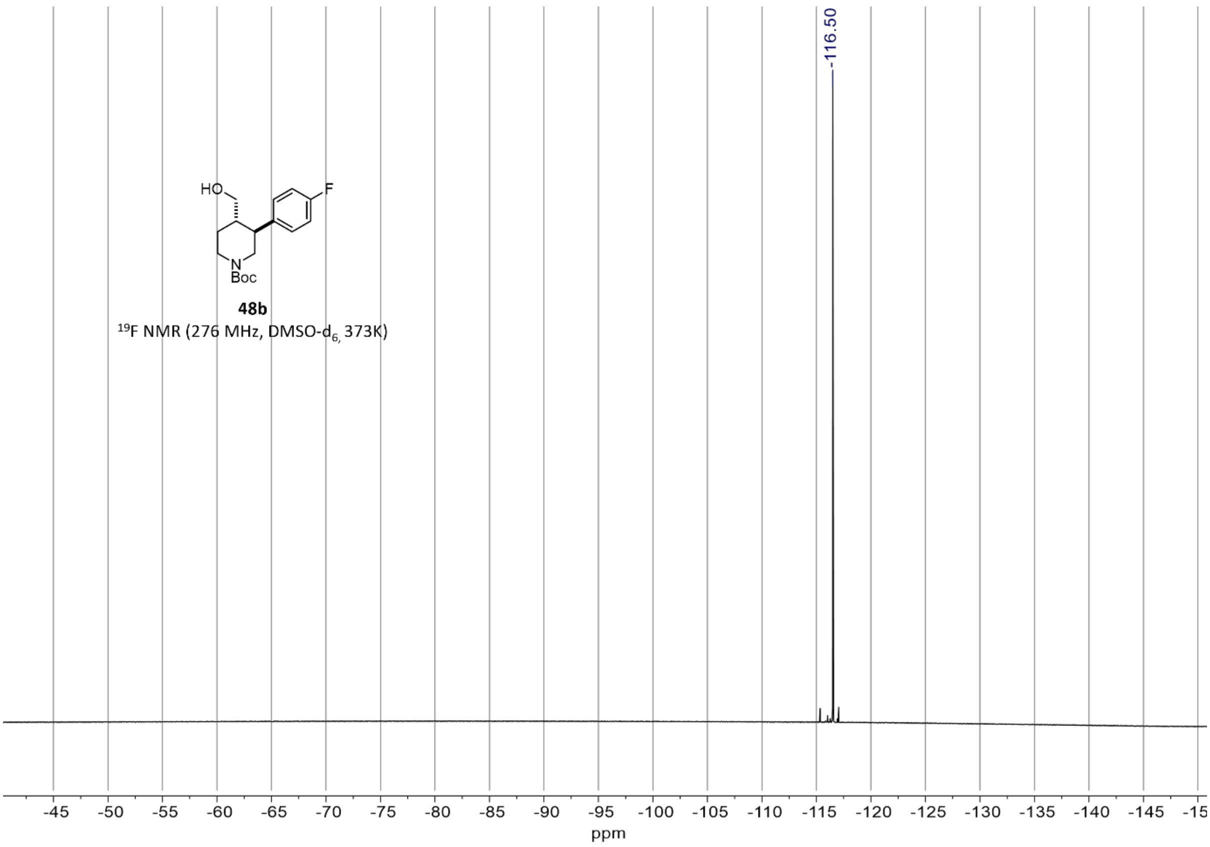

48b
^{19}F NMR (276 MHz, DMSO-d_6, 373K)

cis-(±)-4-Carboxy-3-(4-methoxyphenyl)piperidin-1-ium chloride (FRAG32)

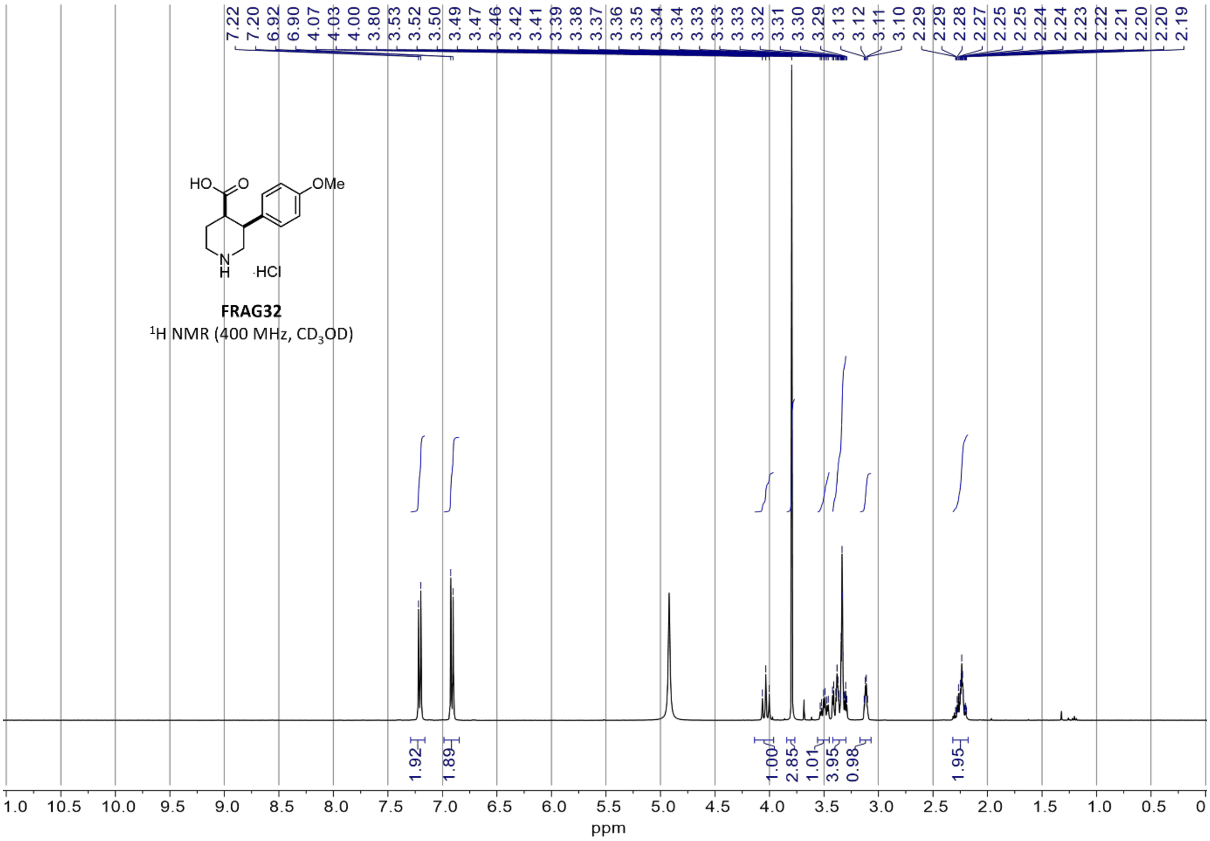

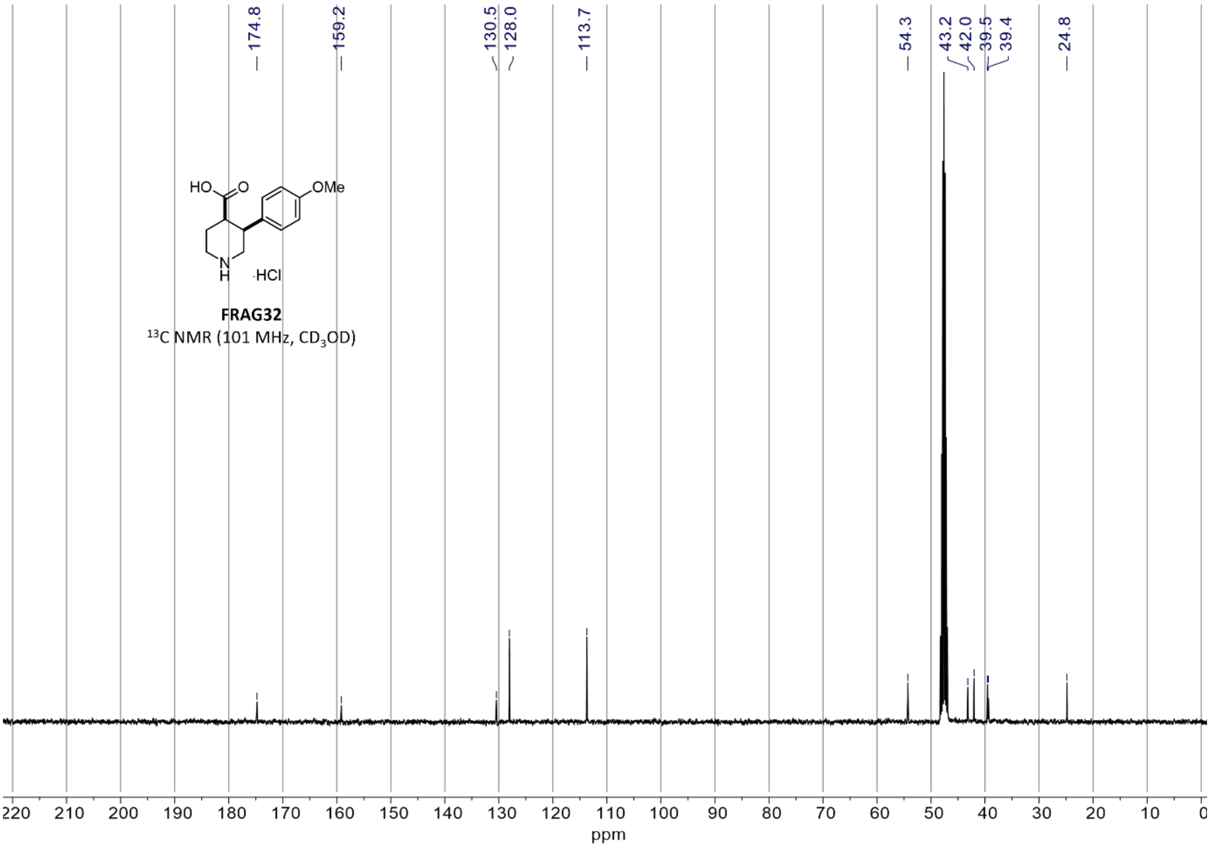

trans-(±)-4-Carboxy-3-(4-methoxyphenyl)piperidin-1-ium chloride (FRAG34)

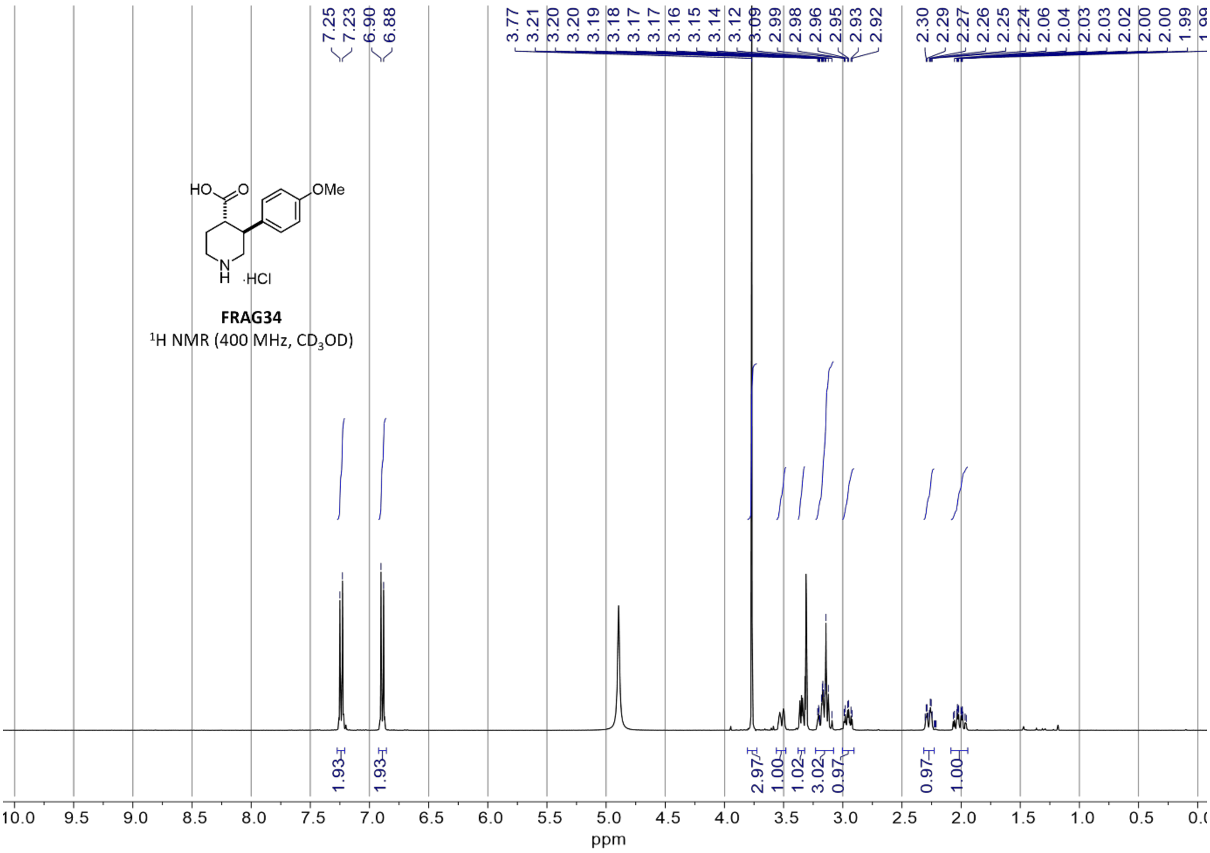

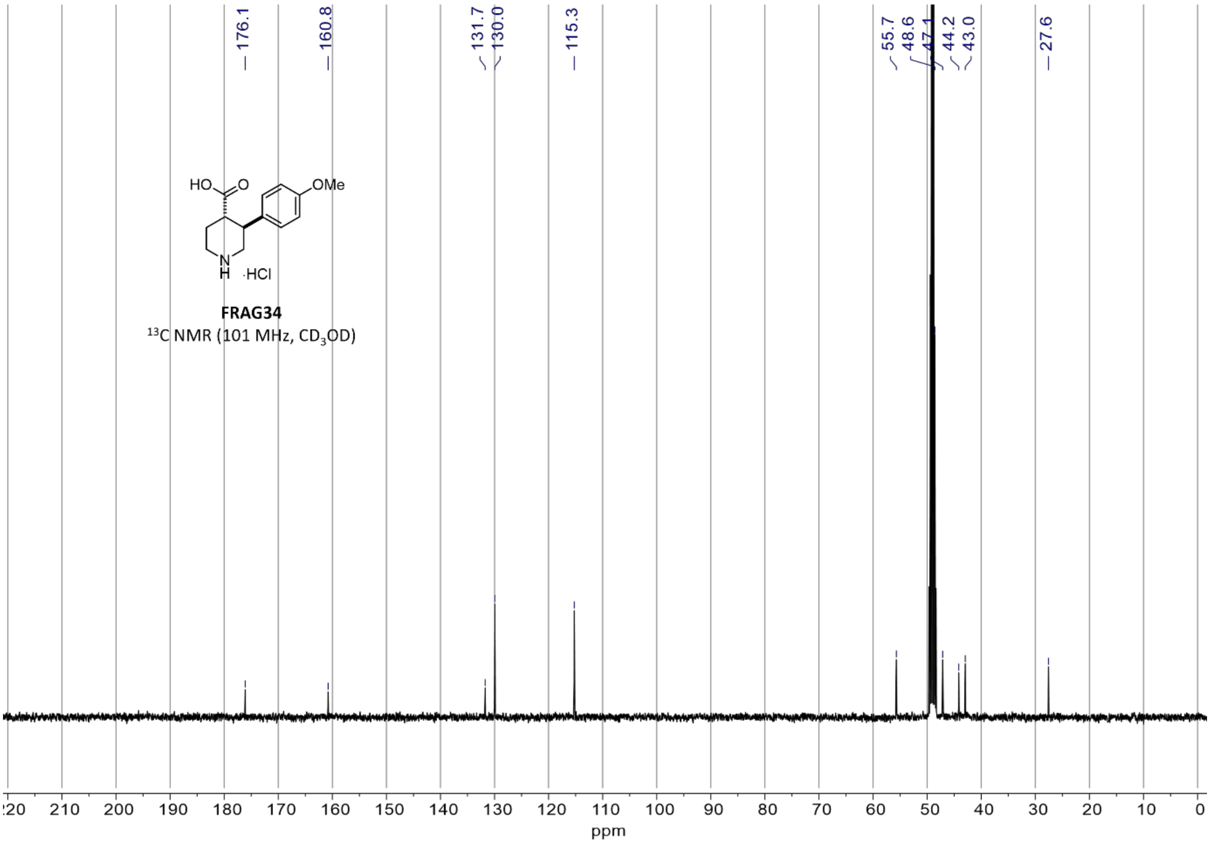

***cis*-(±)-4-Carboxy-3-(4-fluorophenyl)piperidin-1-ium chloride (FRAG33)**

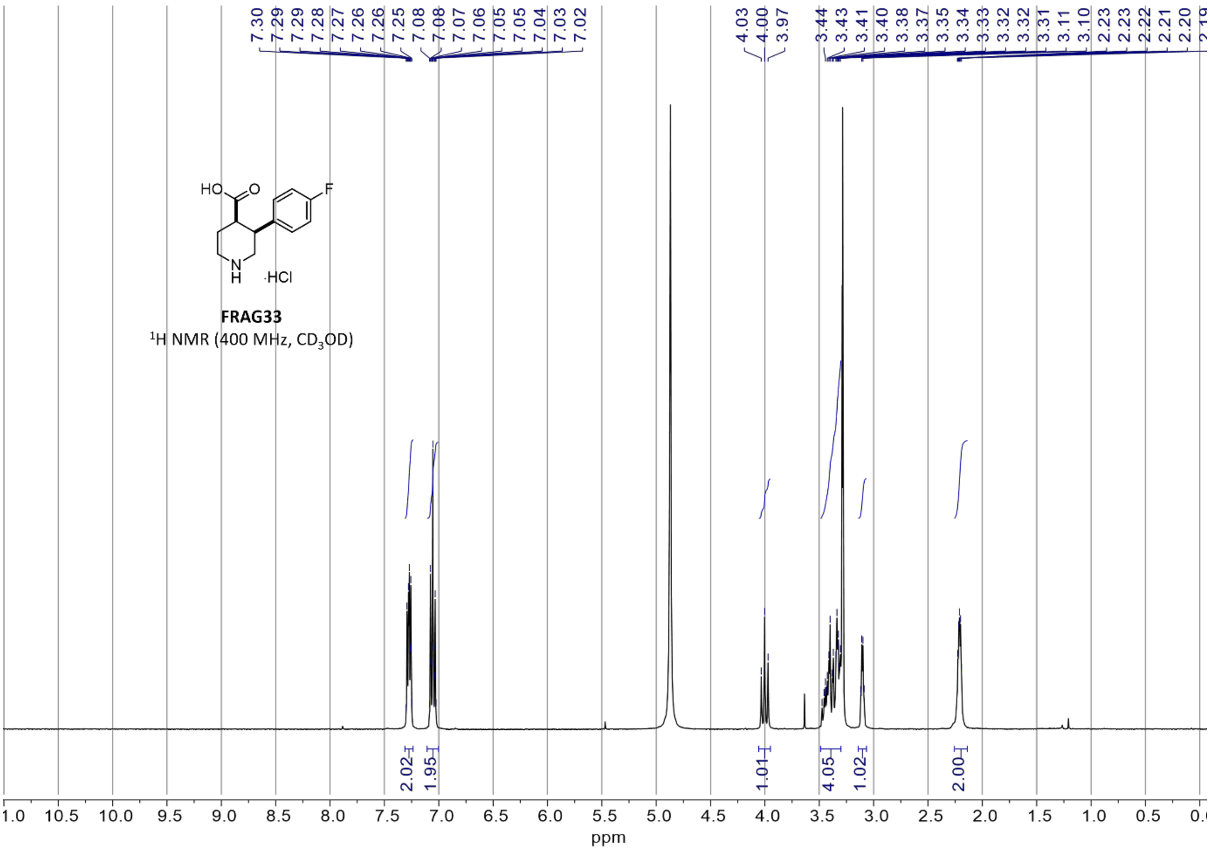

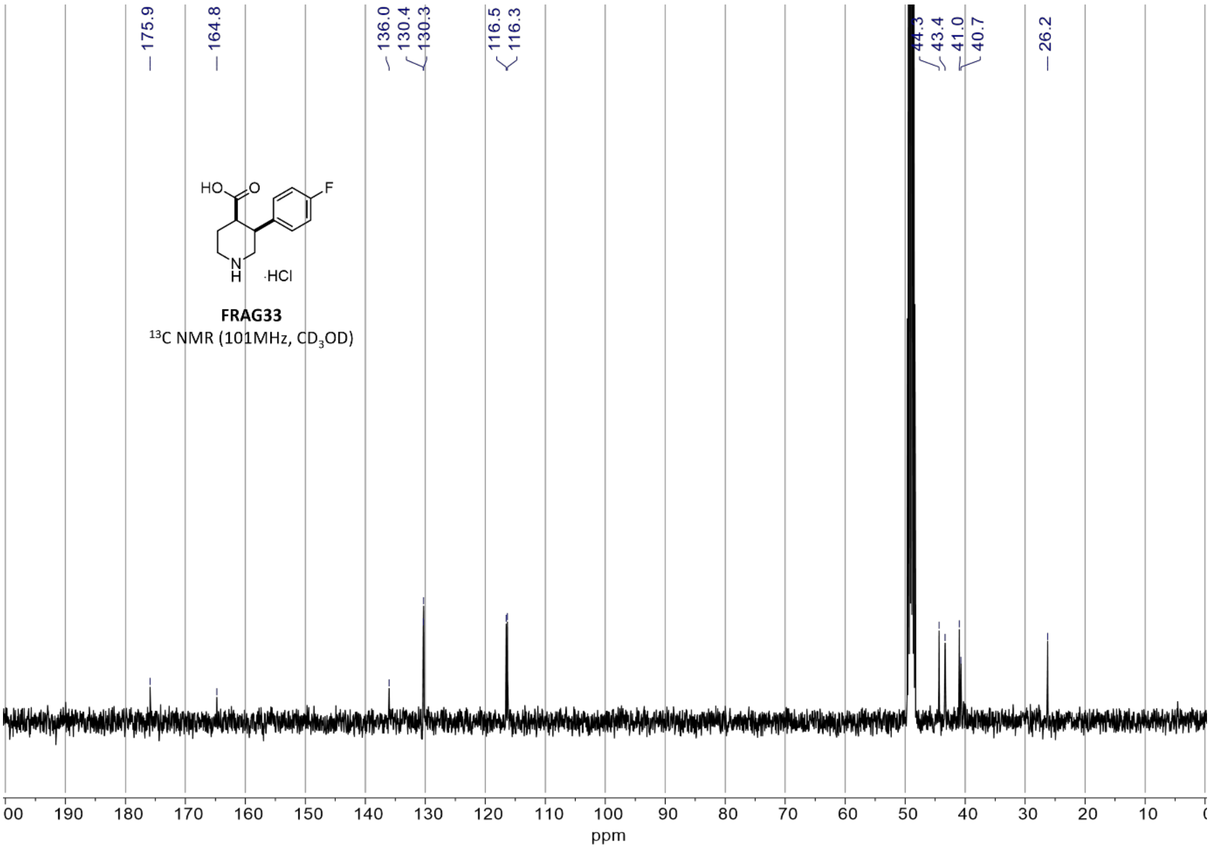

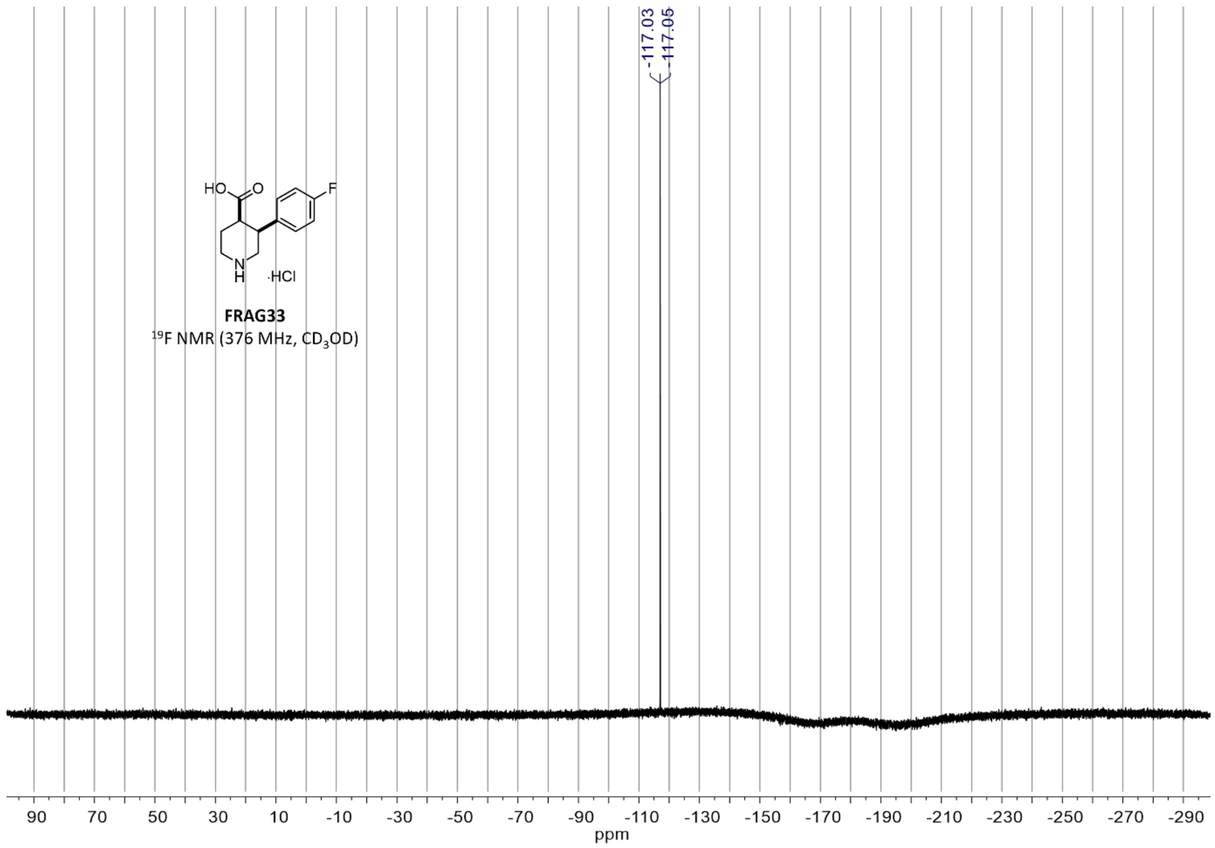

-117.03
-117.05
HO
O
F
N
H
·HCl
FRAG33
^{19}F NMR (376 MHz, CD_3OD)
90
70
50
30
10
-10
-30
-50
-70
-90
-110
-130
-150
-170
-190
-210
-230
-250
-270
-290
ppm

***trans*-(±)-4-Carboxy-3-(4-fluorophenyl)piperidin-1-ium chloride (FRAG35)**

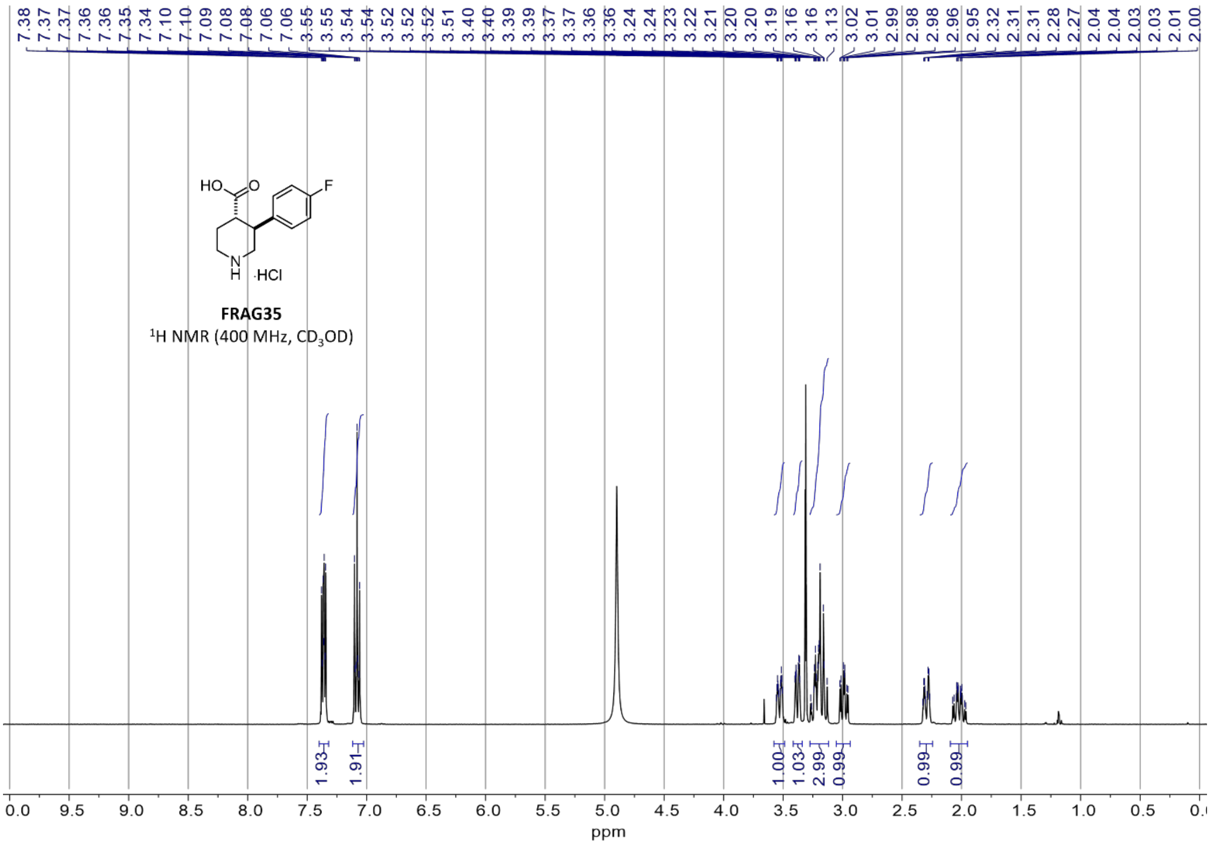

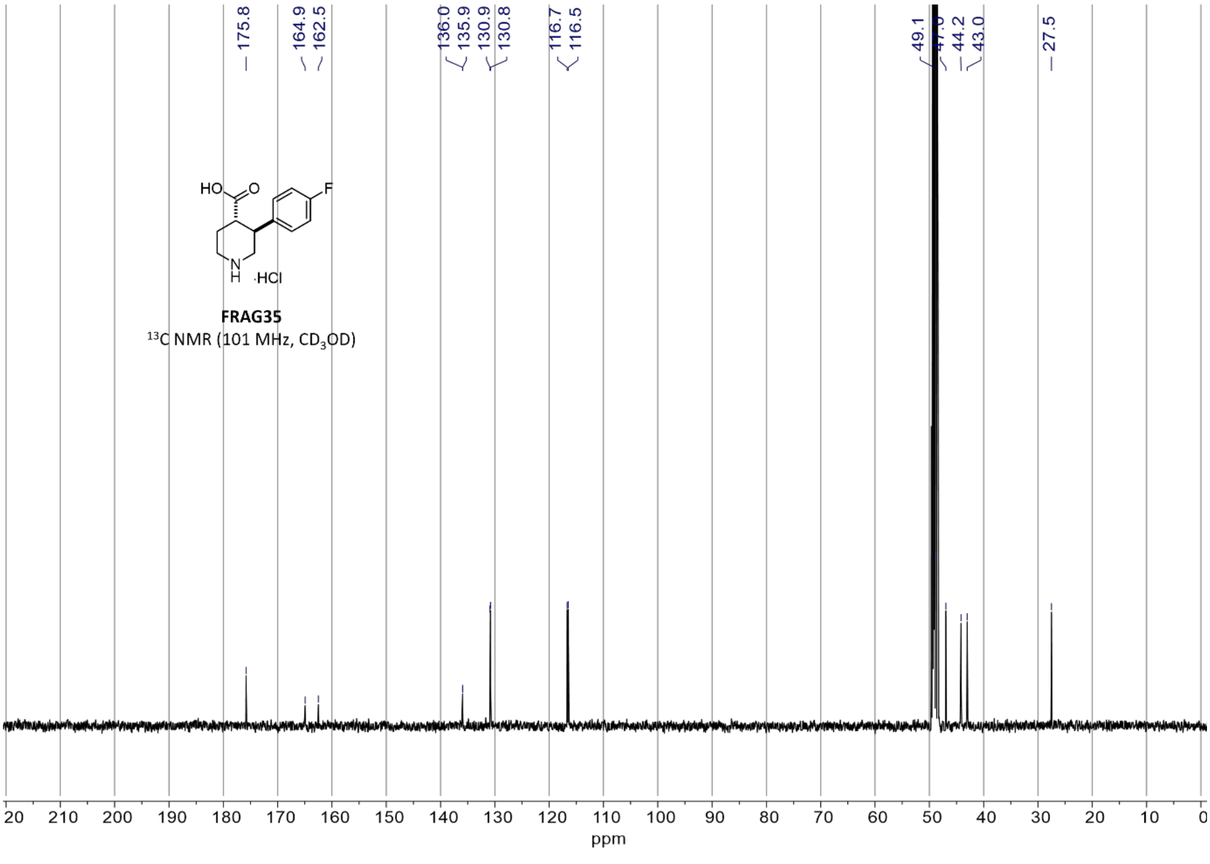

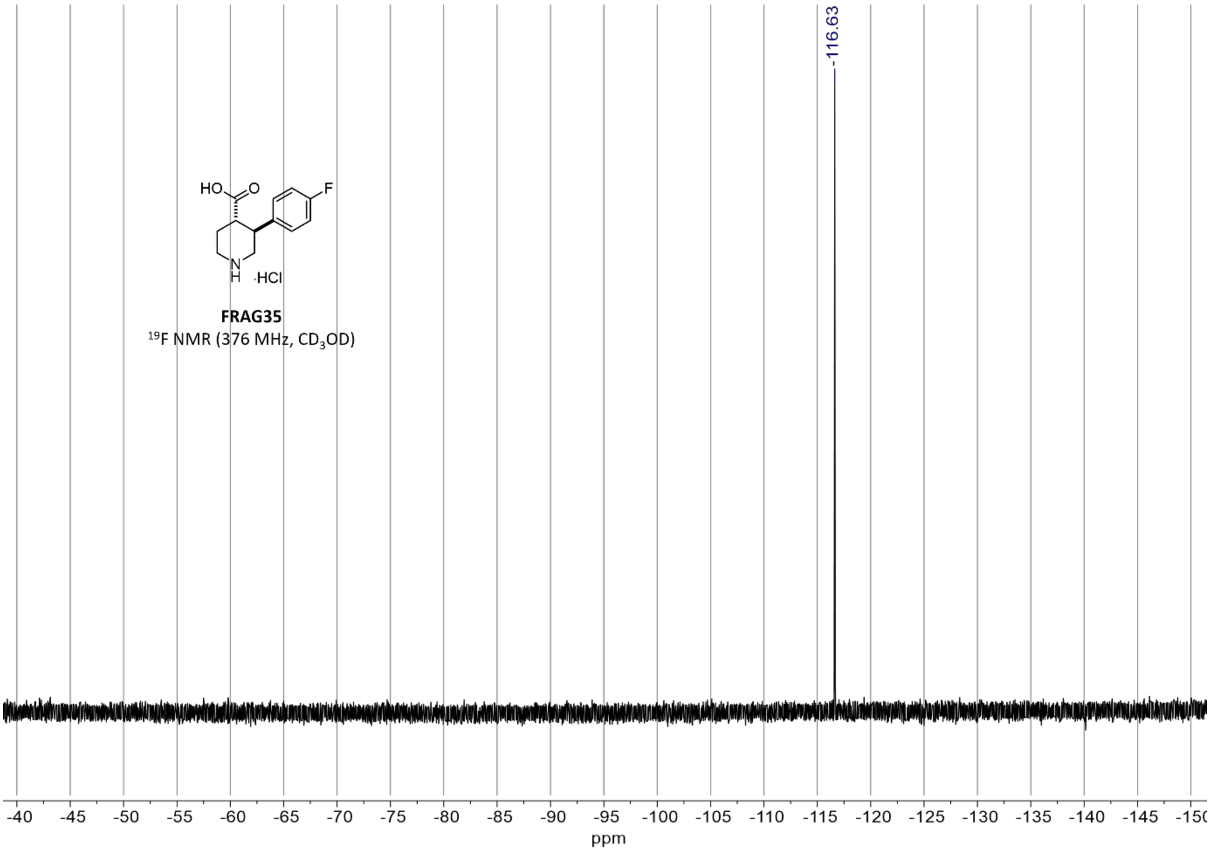

FRAG35
^{19}F NMR (376 MHz, CD_3OD)

cis-(±)-4-Carbamoyl-3-(4-methoxyphenyl)piperidin-1-ium chloride (FRAG36)

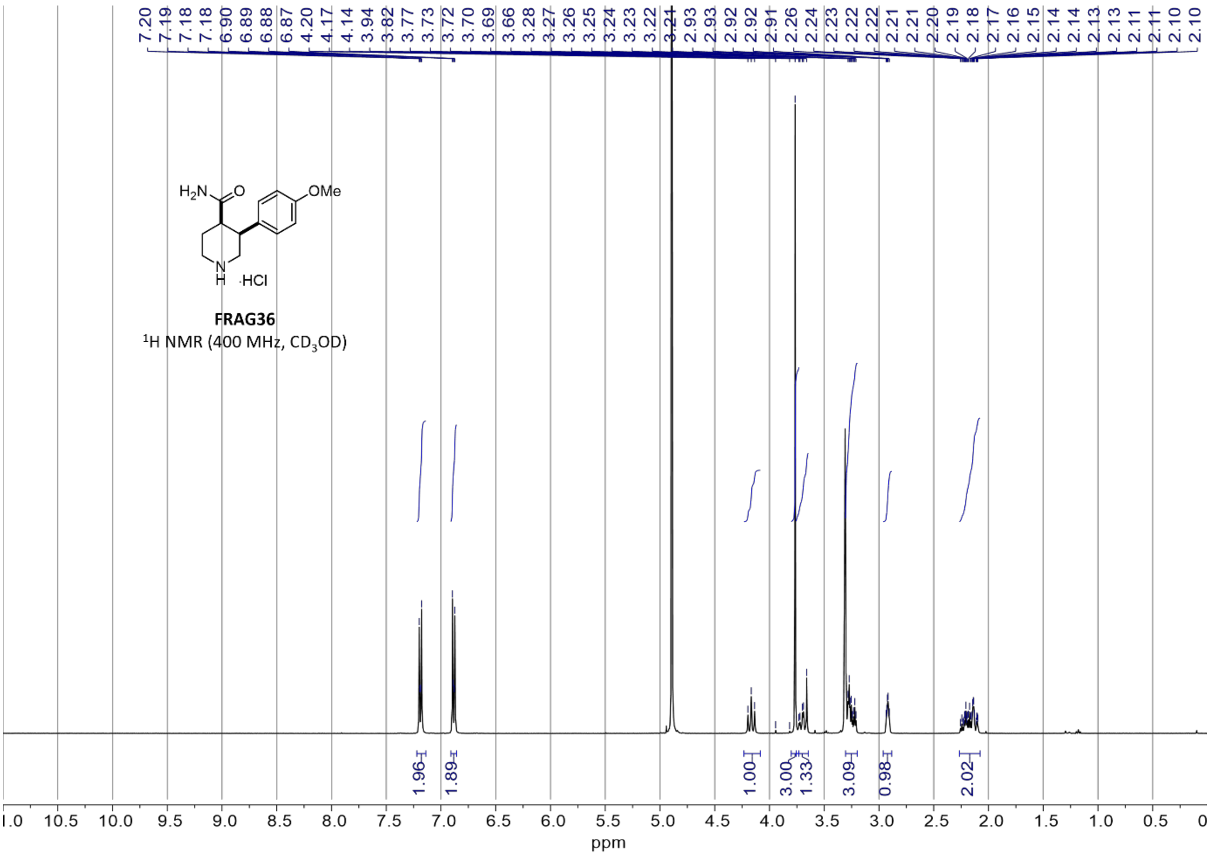

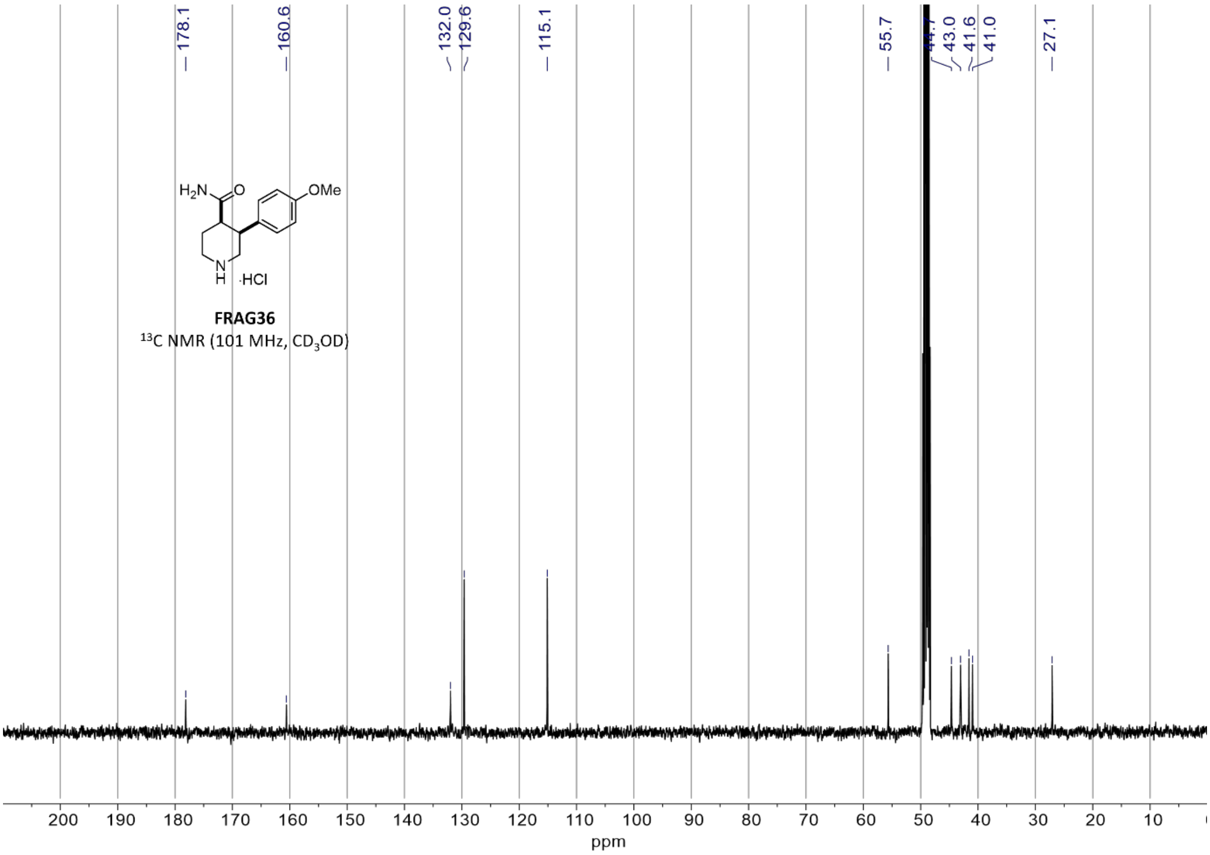

trans-(±)-4-Carbamoyl-3-(4-methoxyphenyl)piperidin-1-ium chloride (FRAG38)

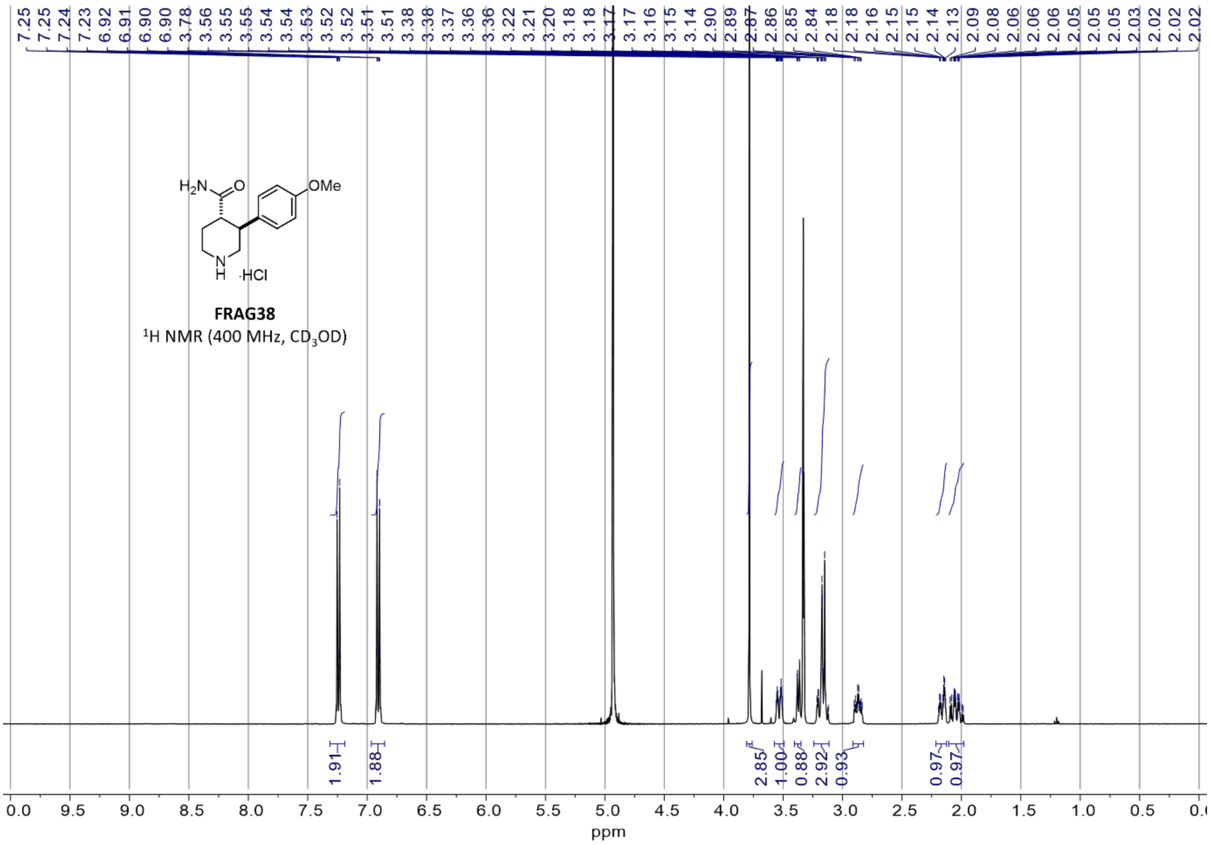

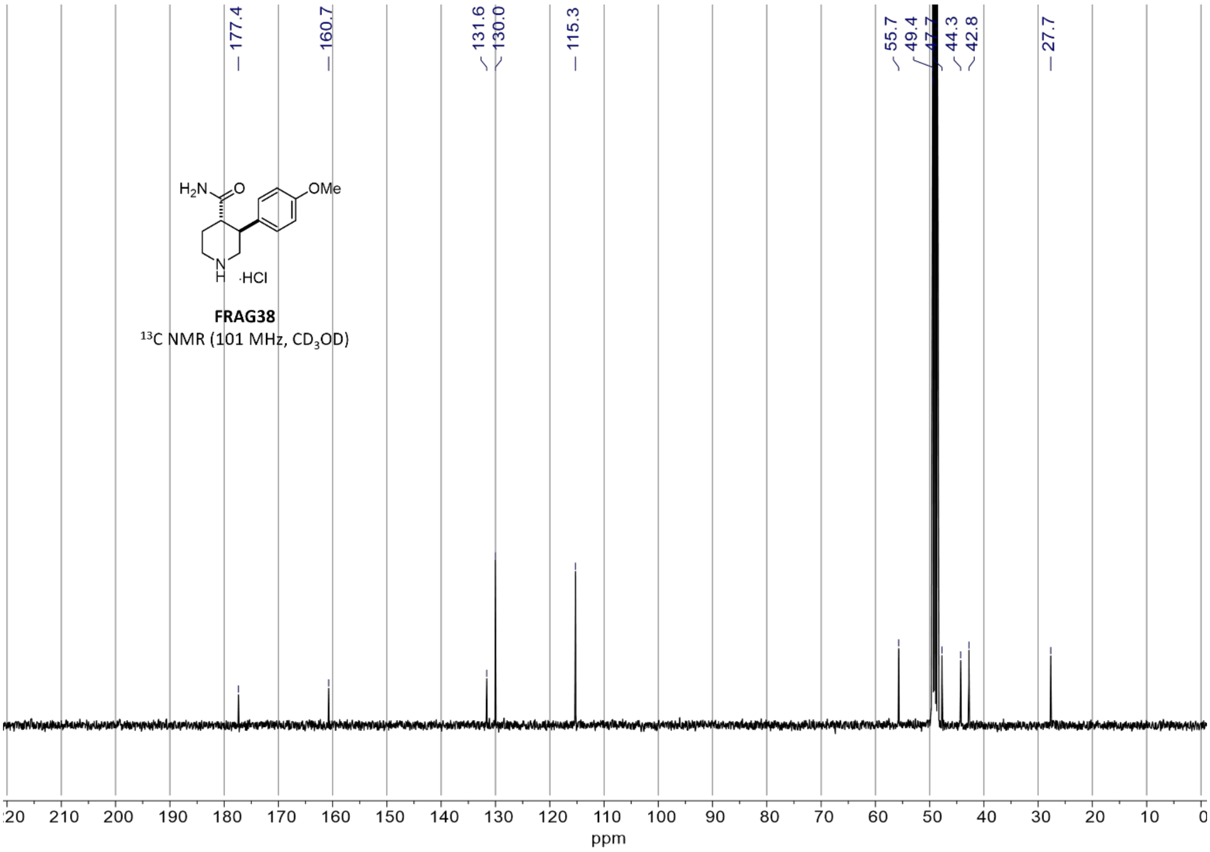

cis-(±)-4-Carbamoyl-3-(4-fluorophenyl)piperidin-1-ium chloride (FRAG37)

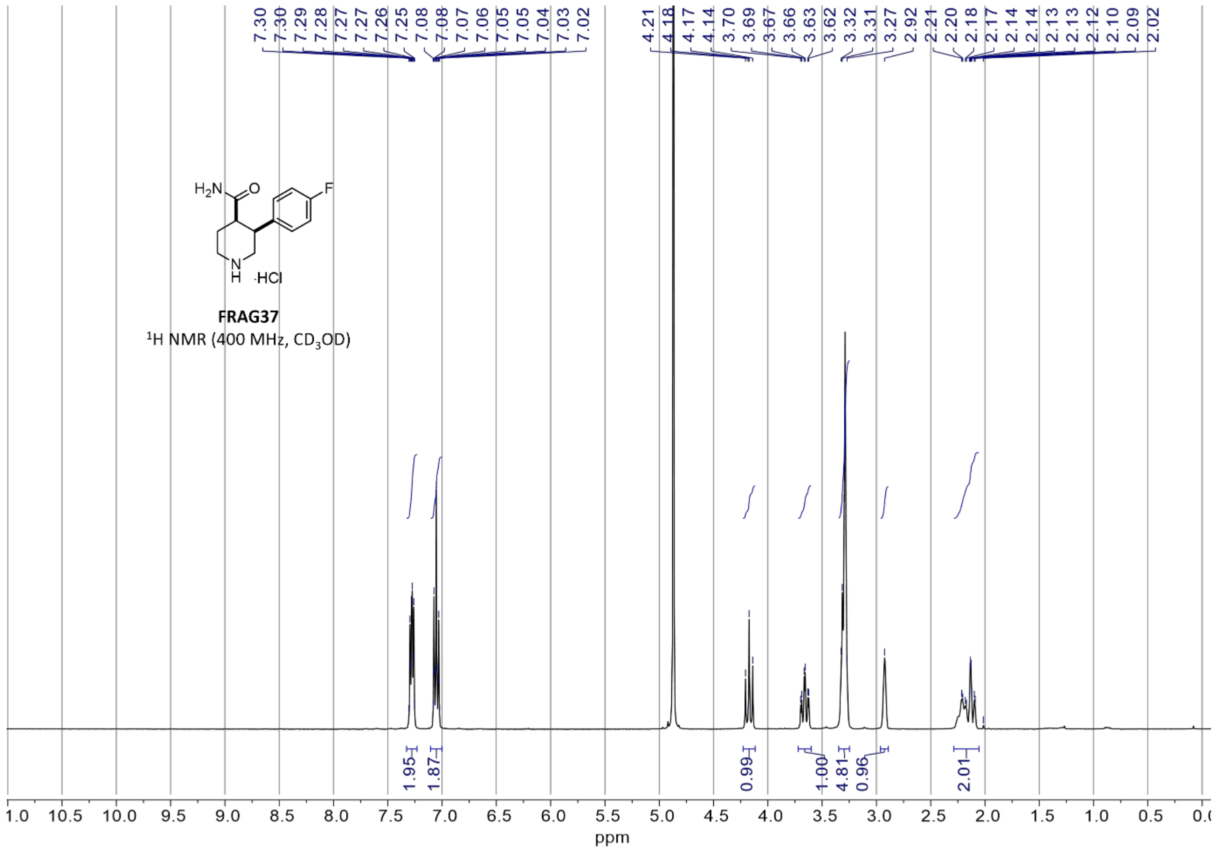

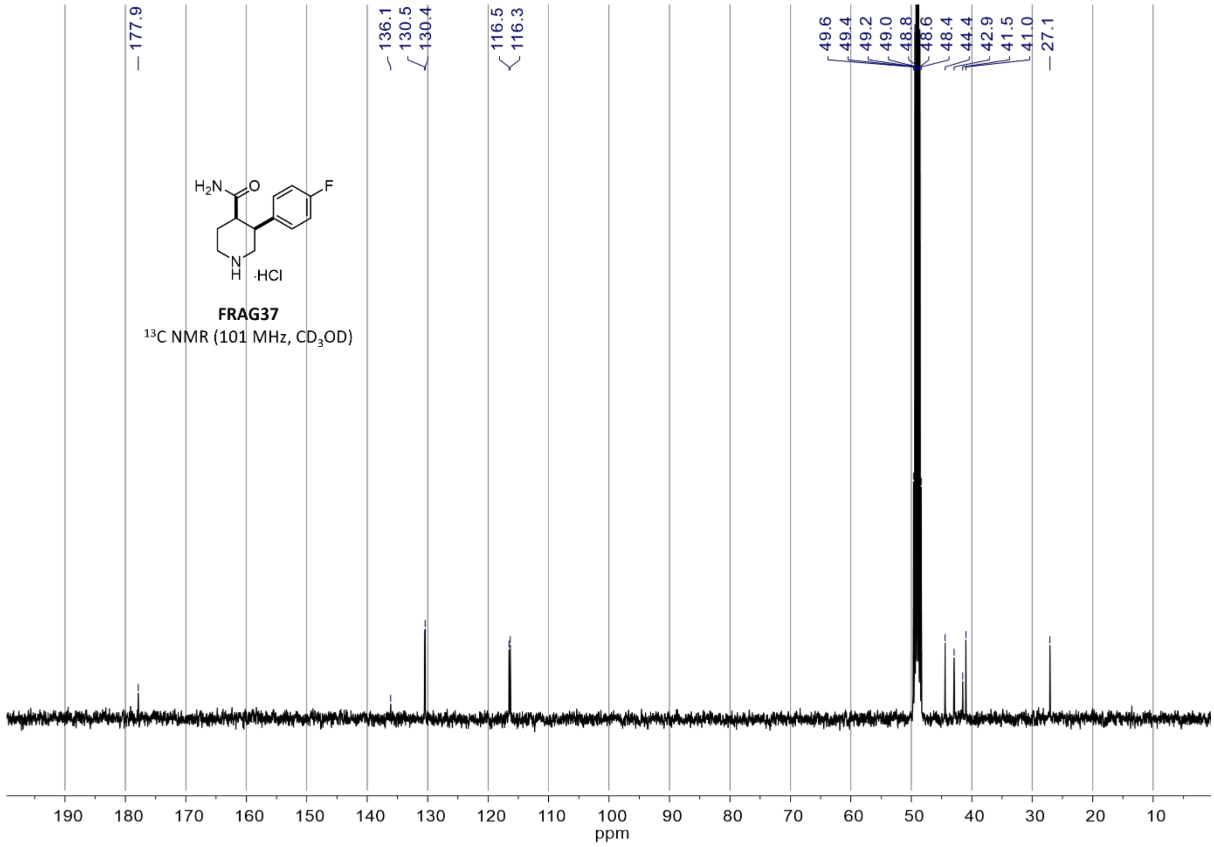

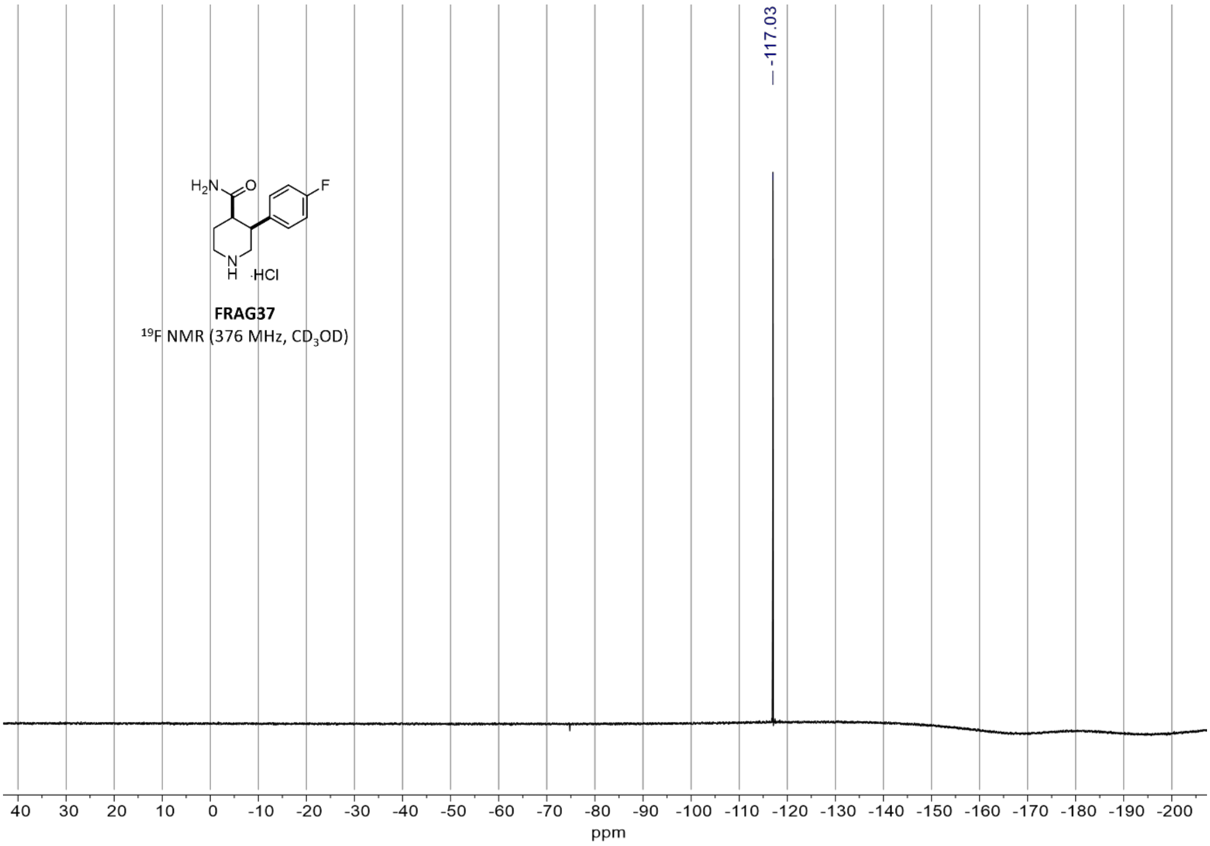
-117.03
H2N
O
F
N
H
·HCl
FRAG37
19F NMR (376 MHz, CD3OD)
40 30 20 10 0 -10 -20 -30 -40 -50 -60 -70 -80 -90 -100 -110 -120 -130 -140 -150 -160 -170 -180 -190 -200
ppm

trans-(±)-4-Carbamoyl-3-(4-fluorophenyl)piperidin-1-ium chloride (FRAG39)

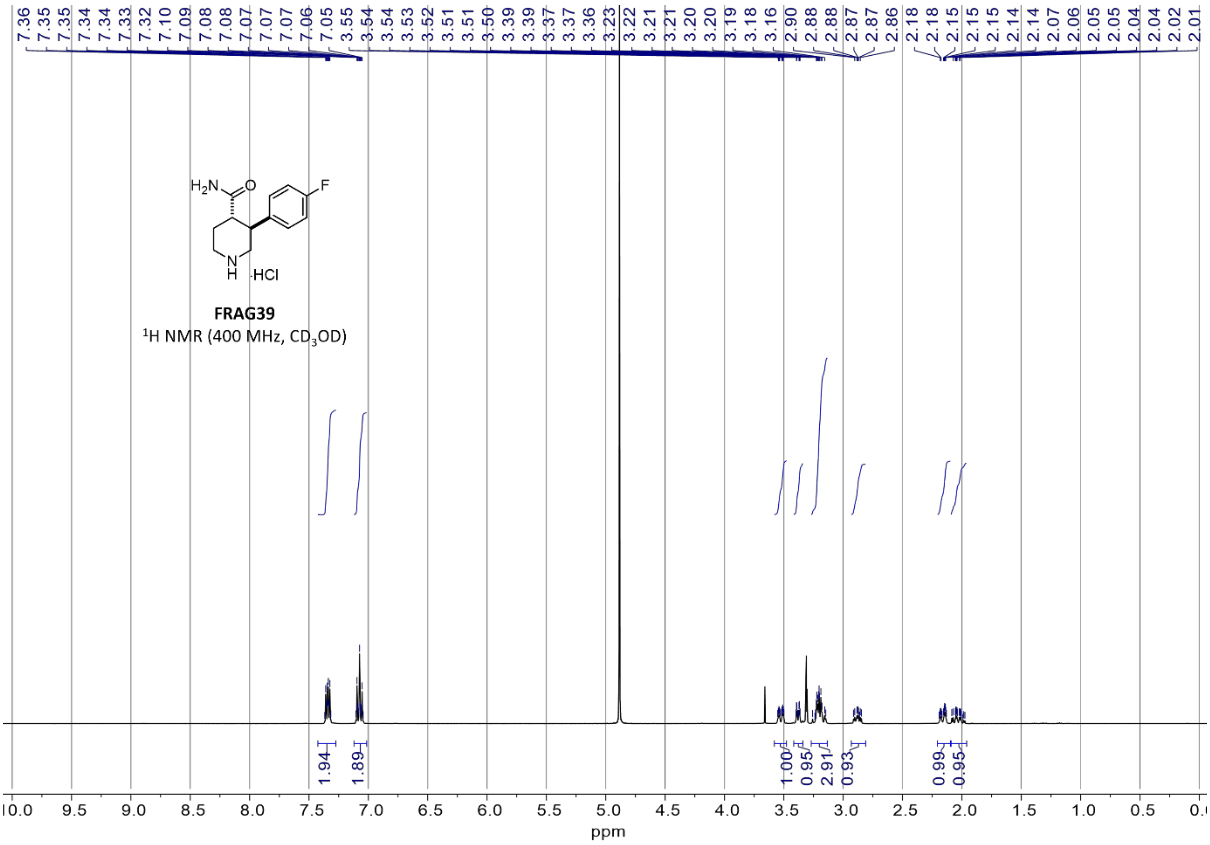

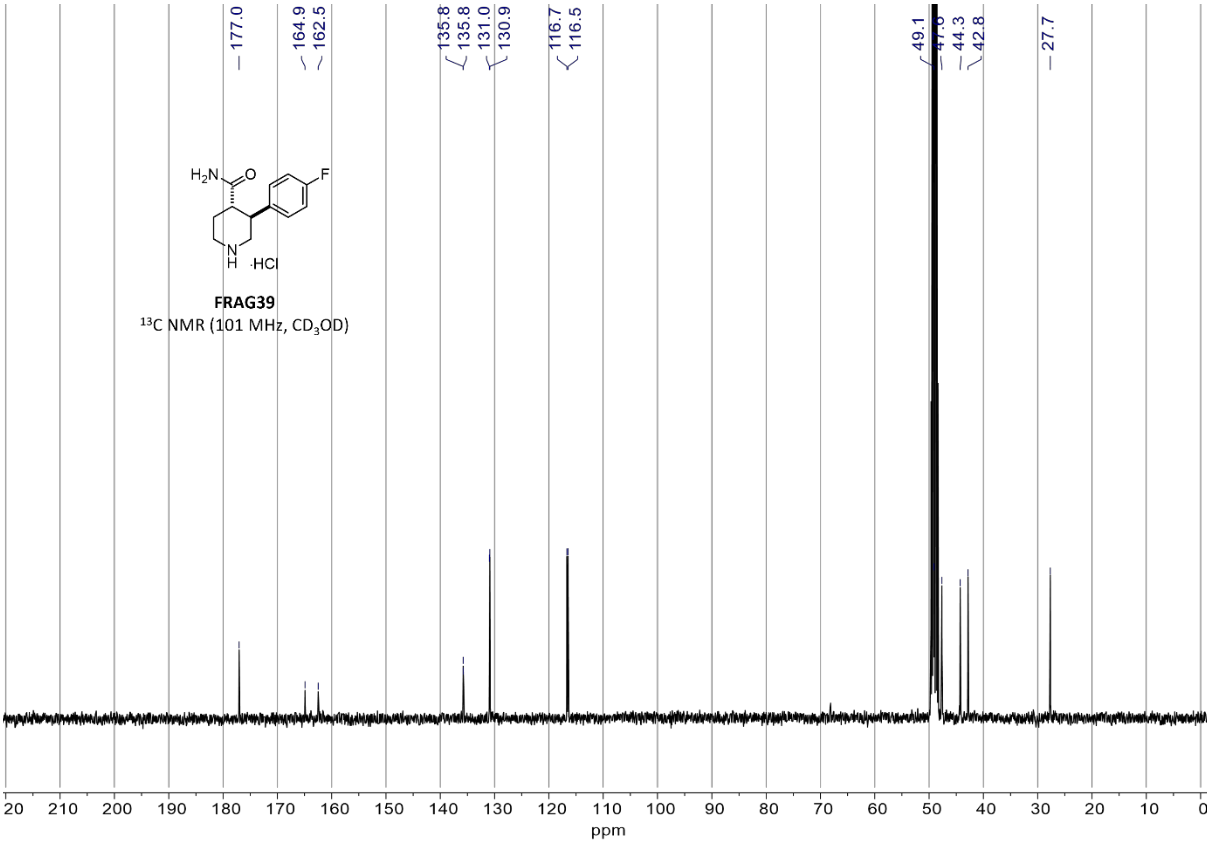

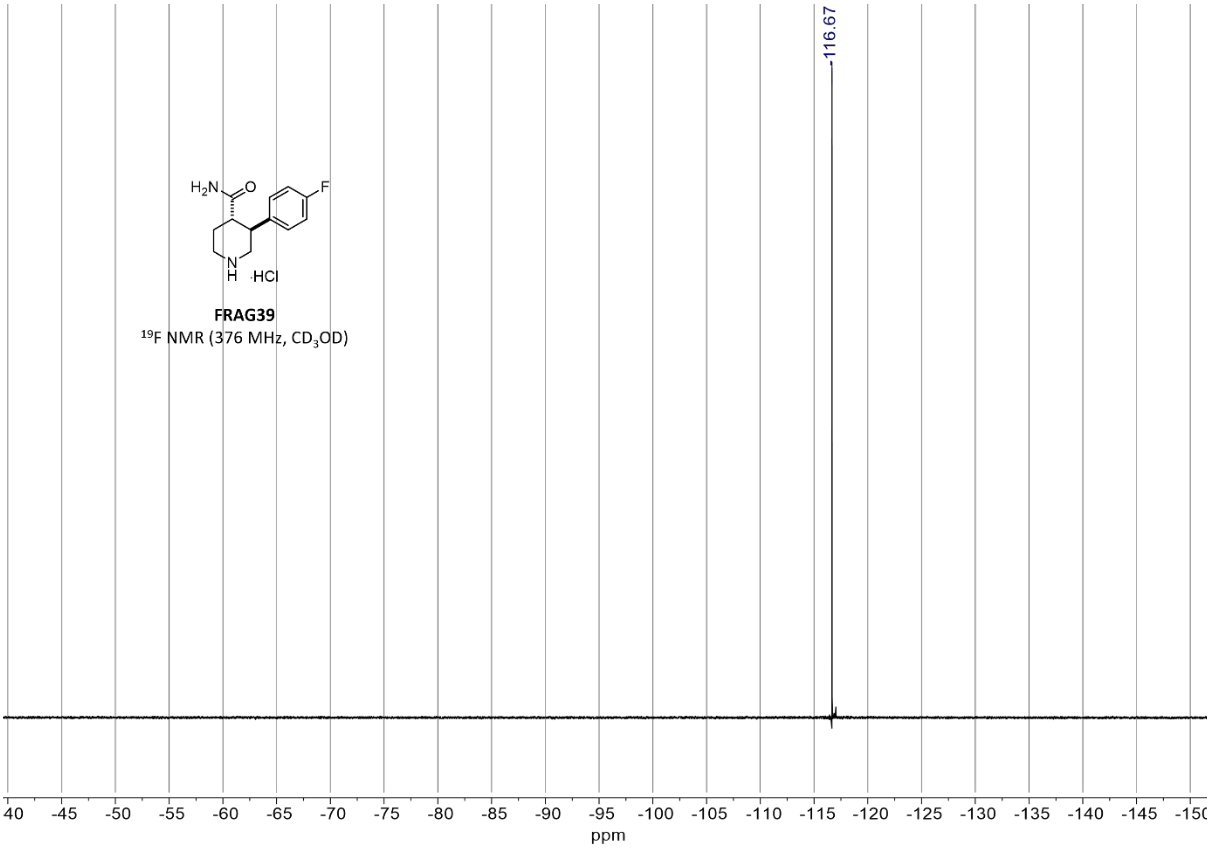
-116.67
H2N
O
F
N
H
·HCl
FRAG39
19F NMR (376 MHz, CD3OD)
40 -45 -50 -55 -60 -65 -70 -75 -80 -85 -90 -95 -100 -105 -110 -115 -120 -125 -130 -135 -140 -145 -150
ppm

cis-(±)-4-Cyano-3-(4-methoxyphenyl)piperidin-1-ium chloride (FRAG44)

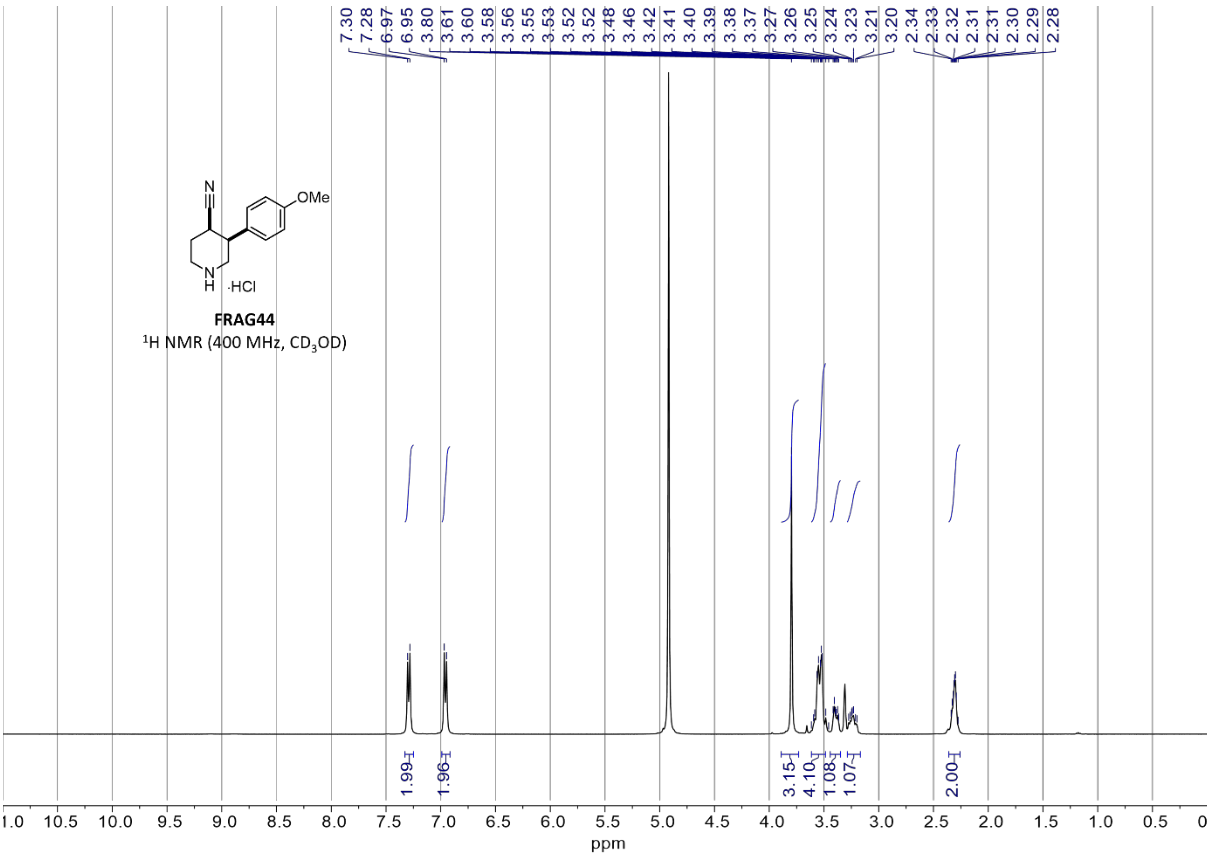

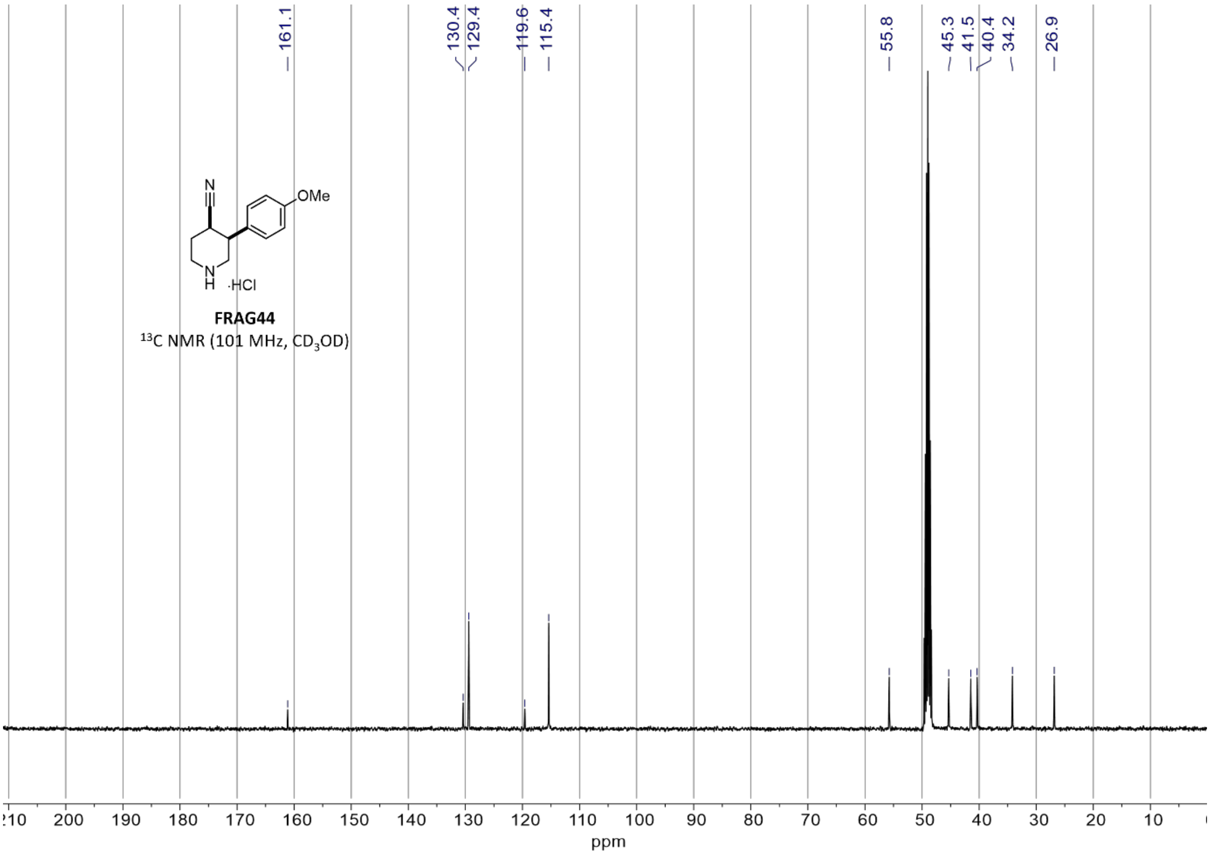

cis-(±)-4-Cyano-3-(4-fluorophenyl)piperidin-1-ium chloride (FRAG45)

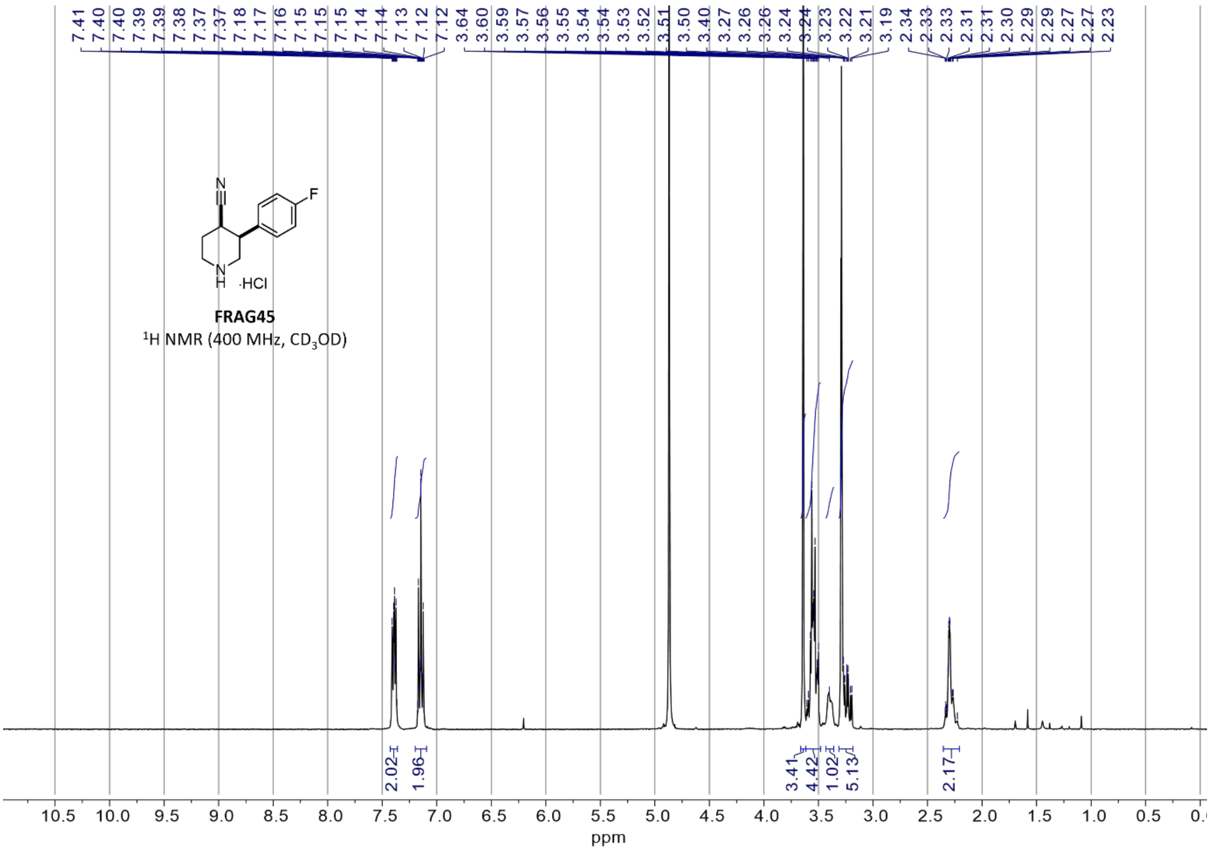

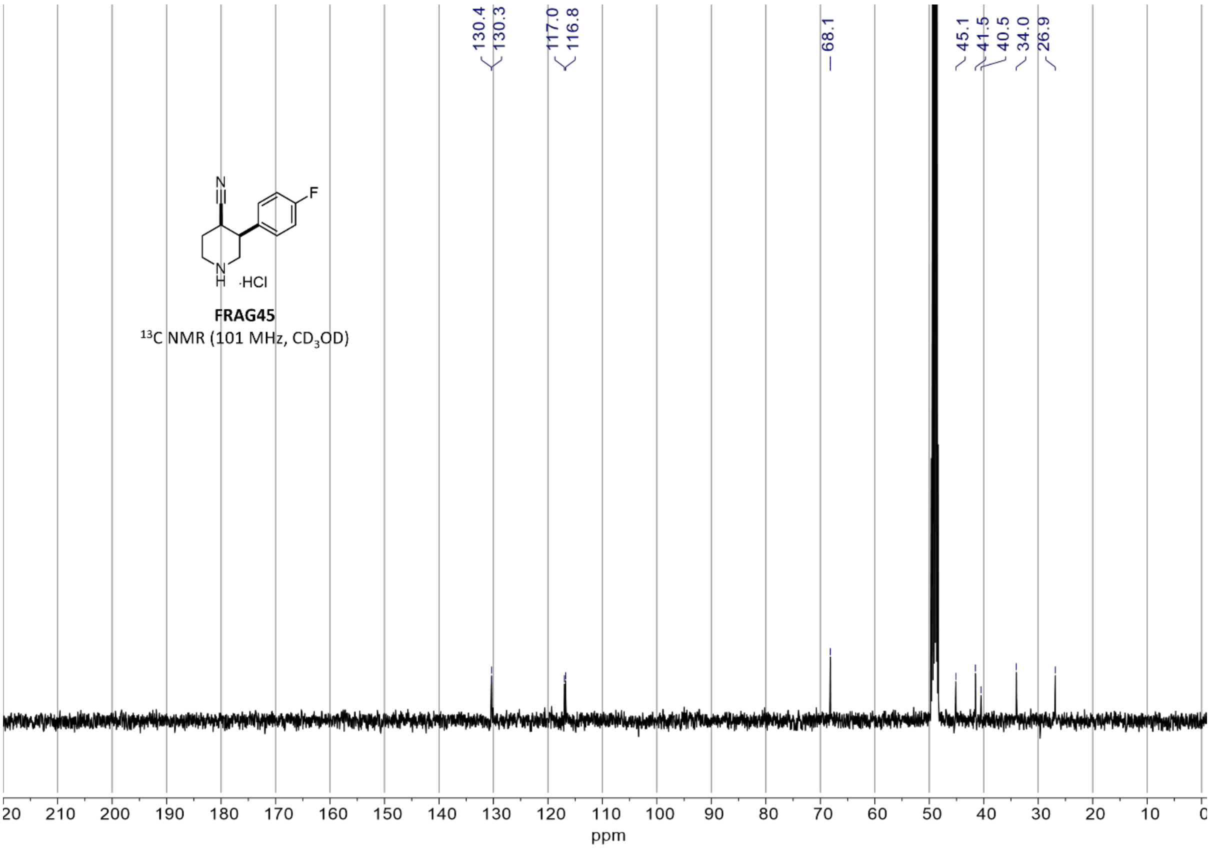

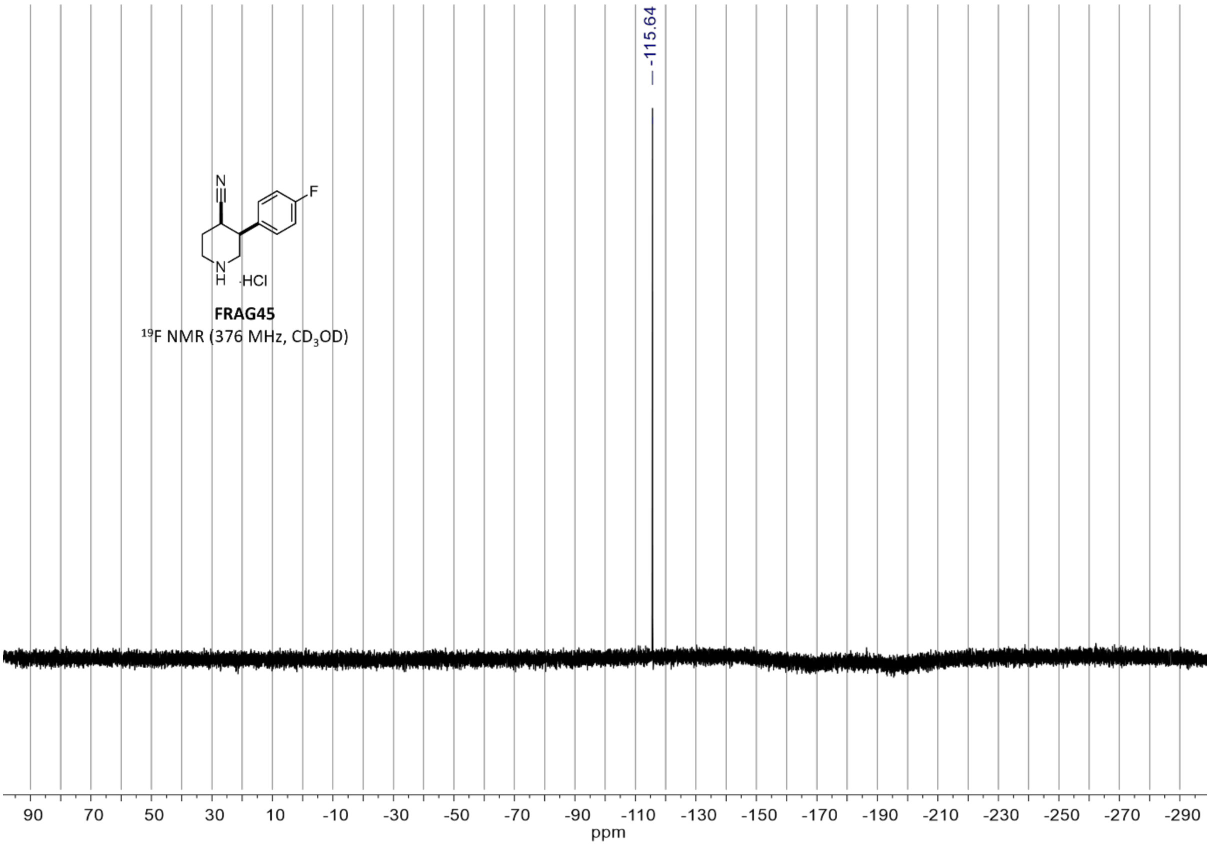
-115.64
N
F
H
·HCl
FRAG45
^{19}F NMR (376 MHz, CD_3OD)
90 70 50 30 10 -10 -30 -50 -70 -90 -110 -130 -150 -170 -190 -210 -230 -250 -270 -290
ppm

cis-(±)-4-(Hydroxymethyl)-3-(4-methoxyphenyl)piperidin-1-ium chloride (FRAG40)

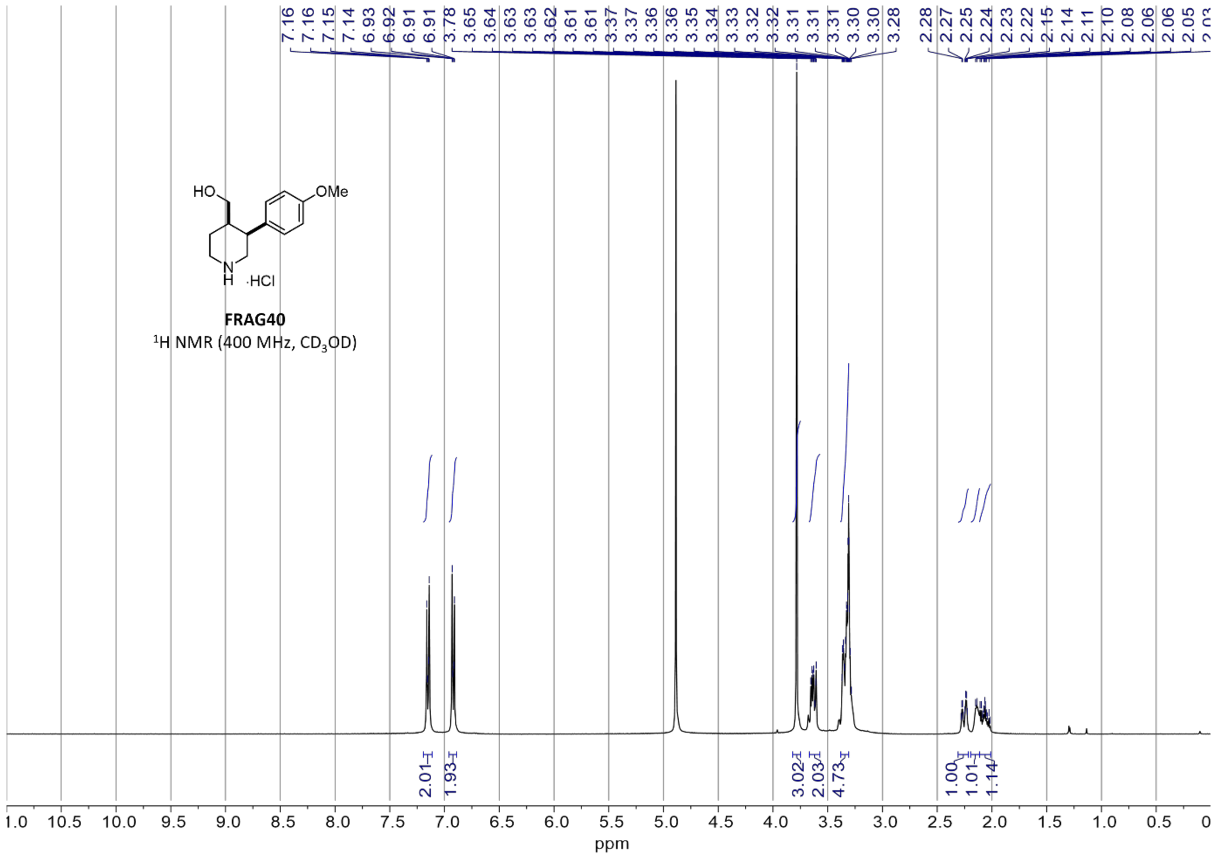

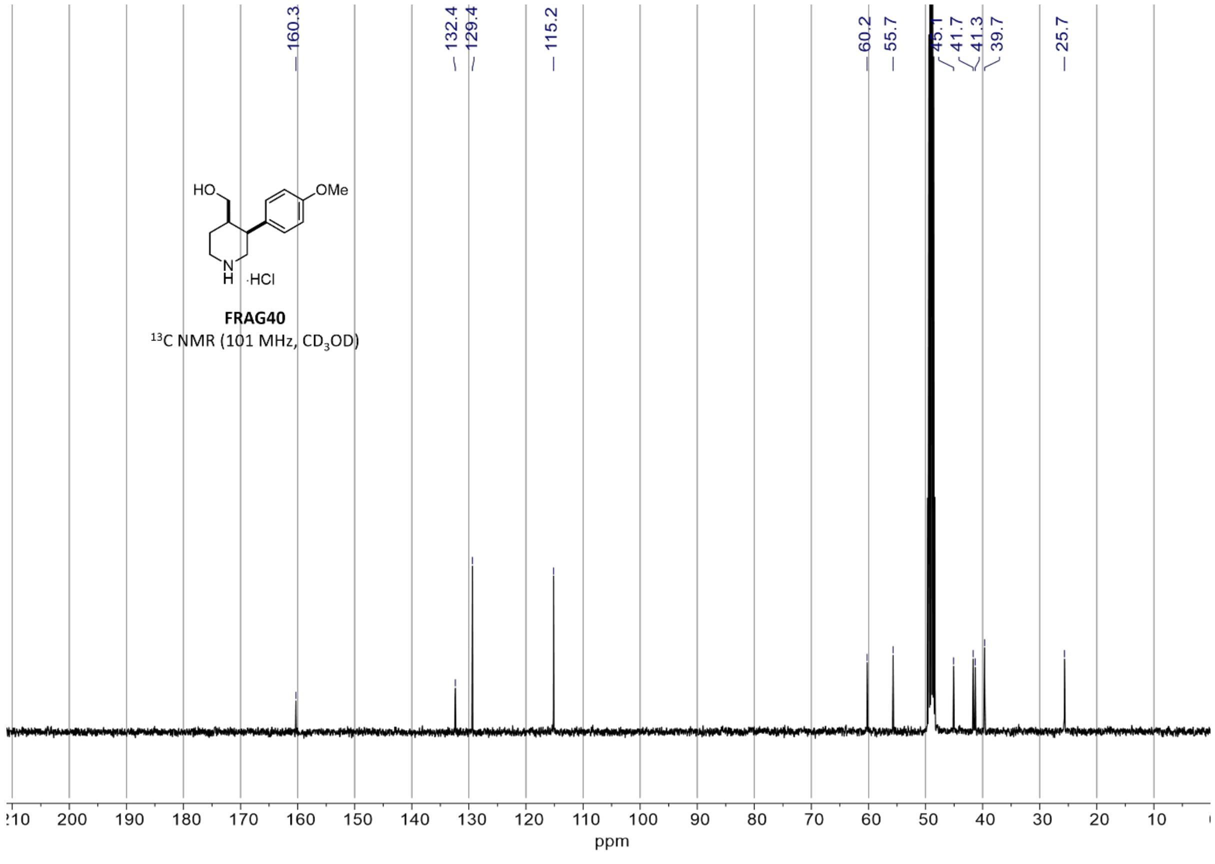

trans-(±)-4-(Hydroxymethyl)-3-(4-methoxyphenyl)piperidin-1-ium chloride (FRAG42)

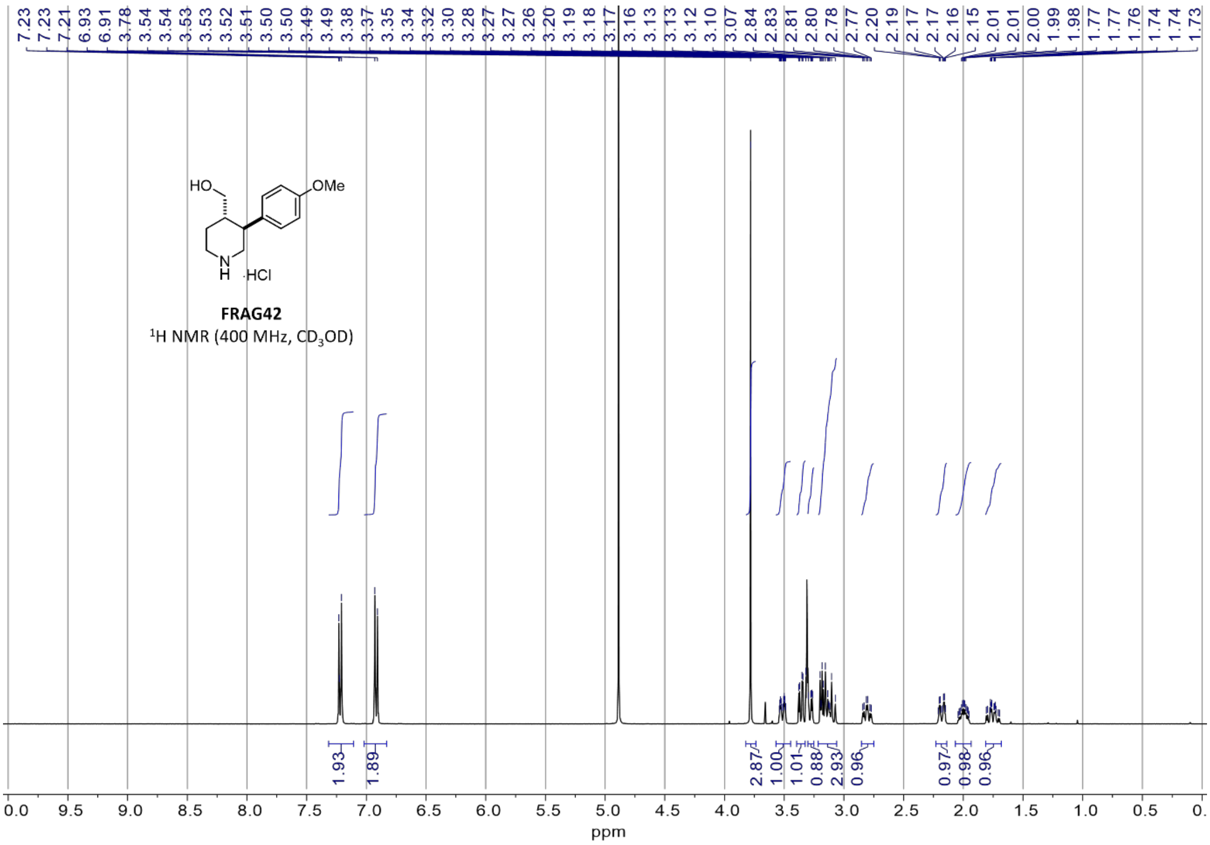

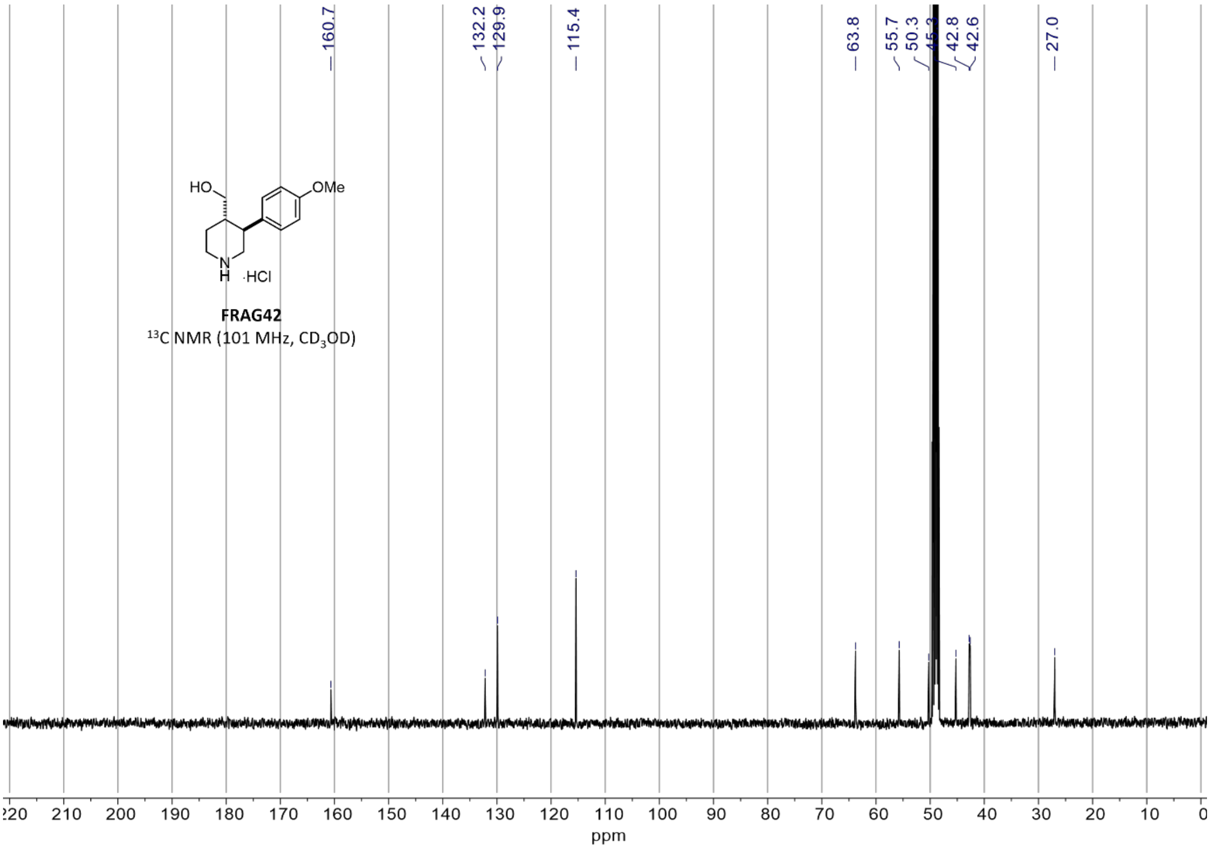

***cis*-(±)-4-(Hydroxymethyl)-3-(4-fluorophenyl)piperidin-1-ium chloride (FRAG41)**

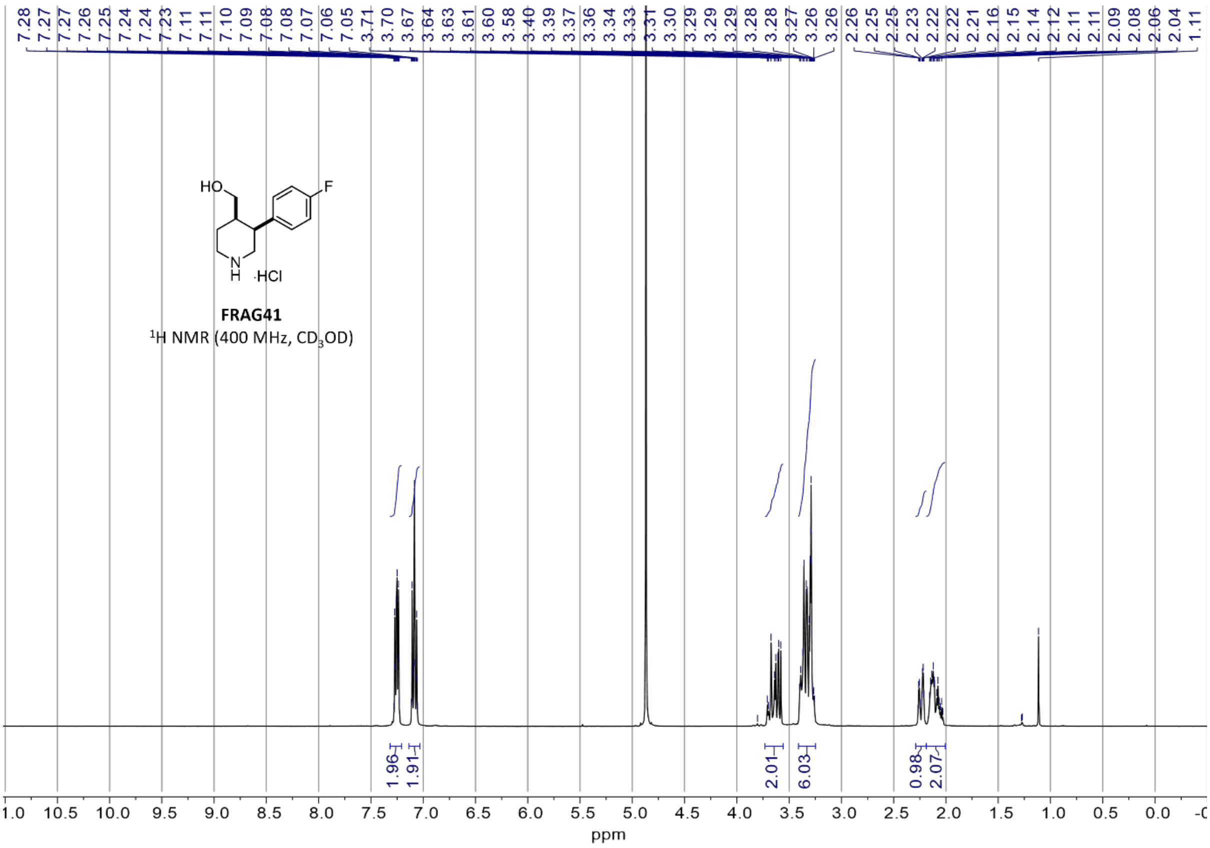

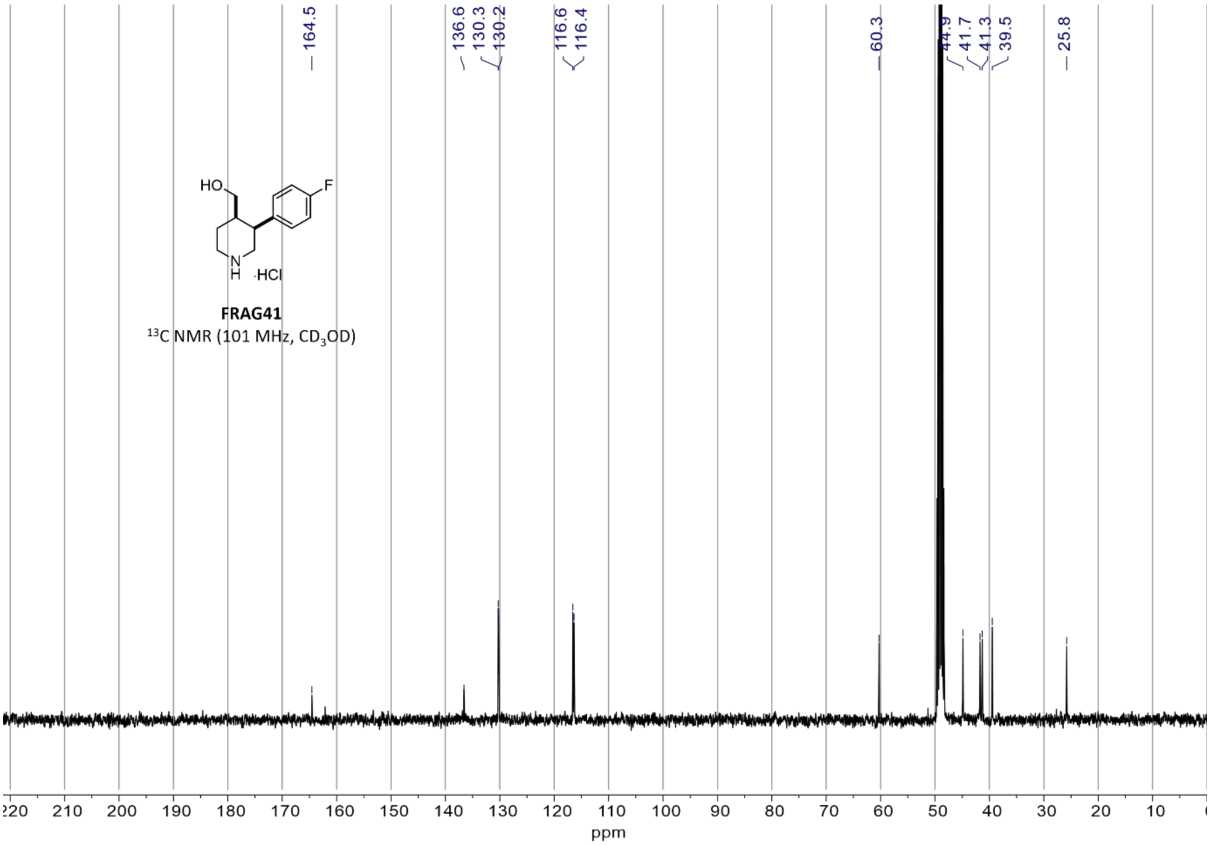

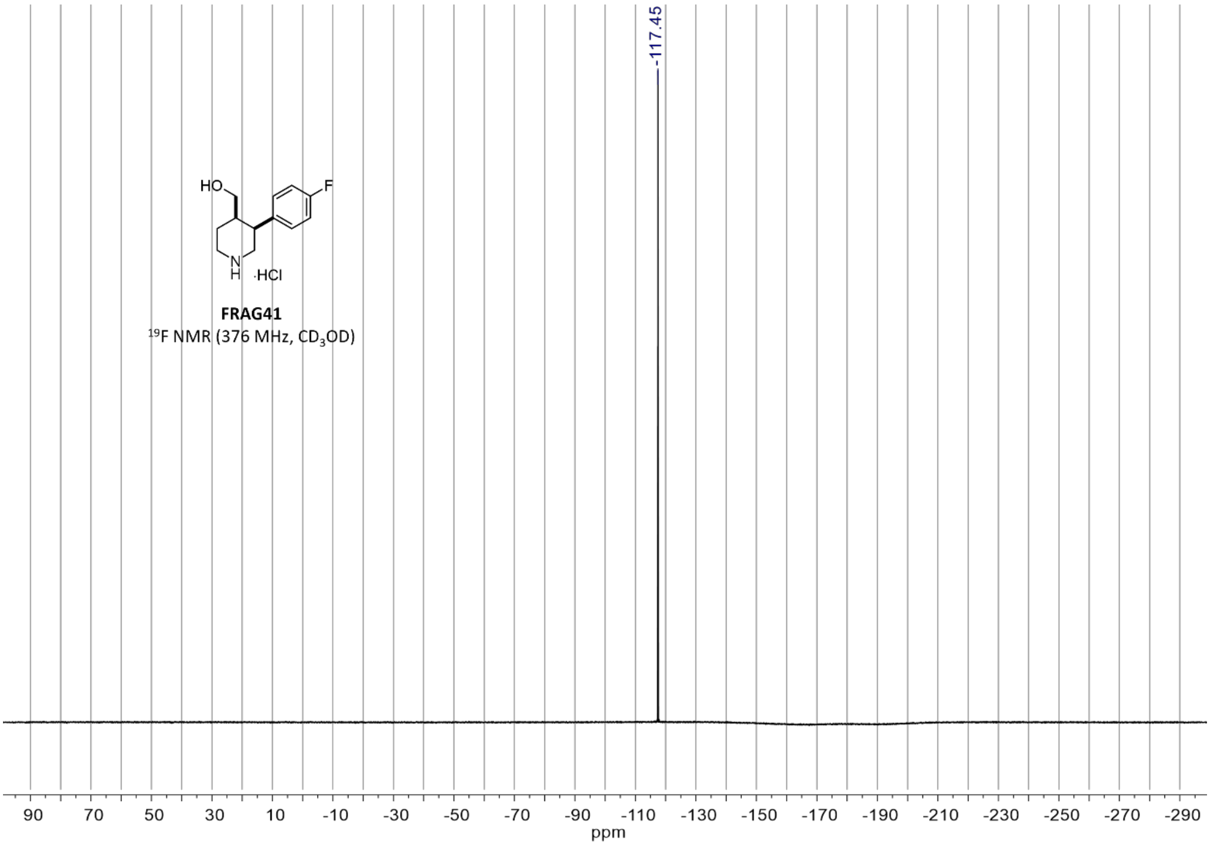

FRAG41
^{19}F NMR (376 MHz, CD_3OD)

trans-(±)-4-(Hydroxymethyl)-3-(4-fluorophenyl)piperidin-1-ium chloride (FRAG43)

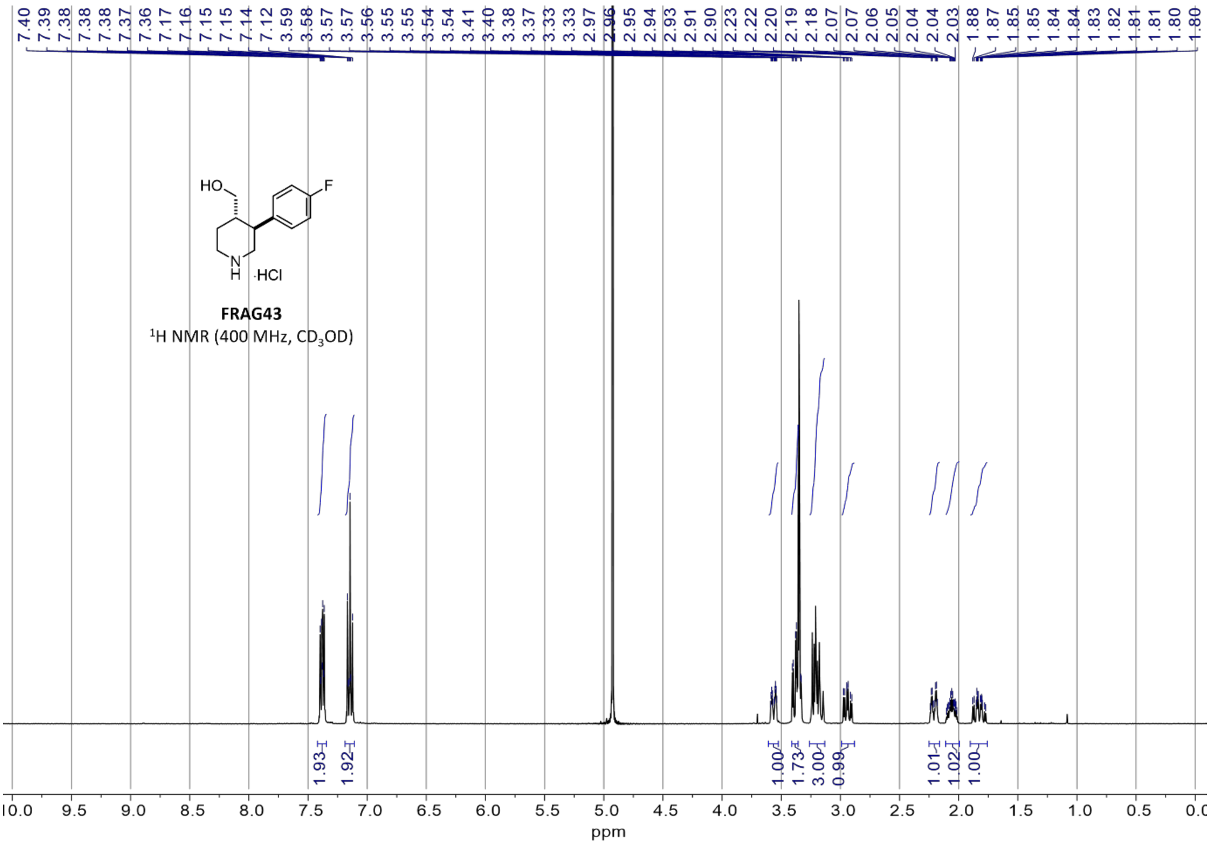

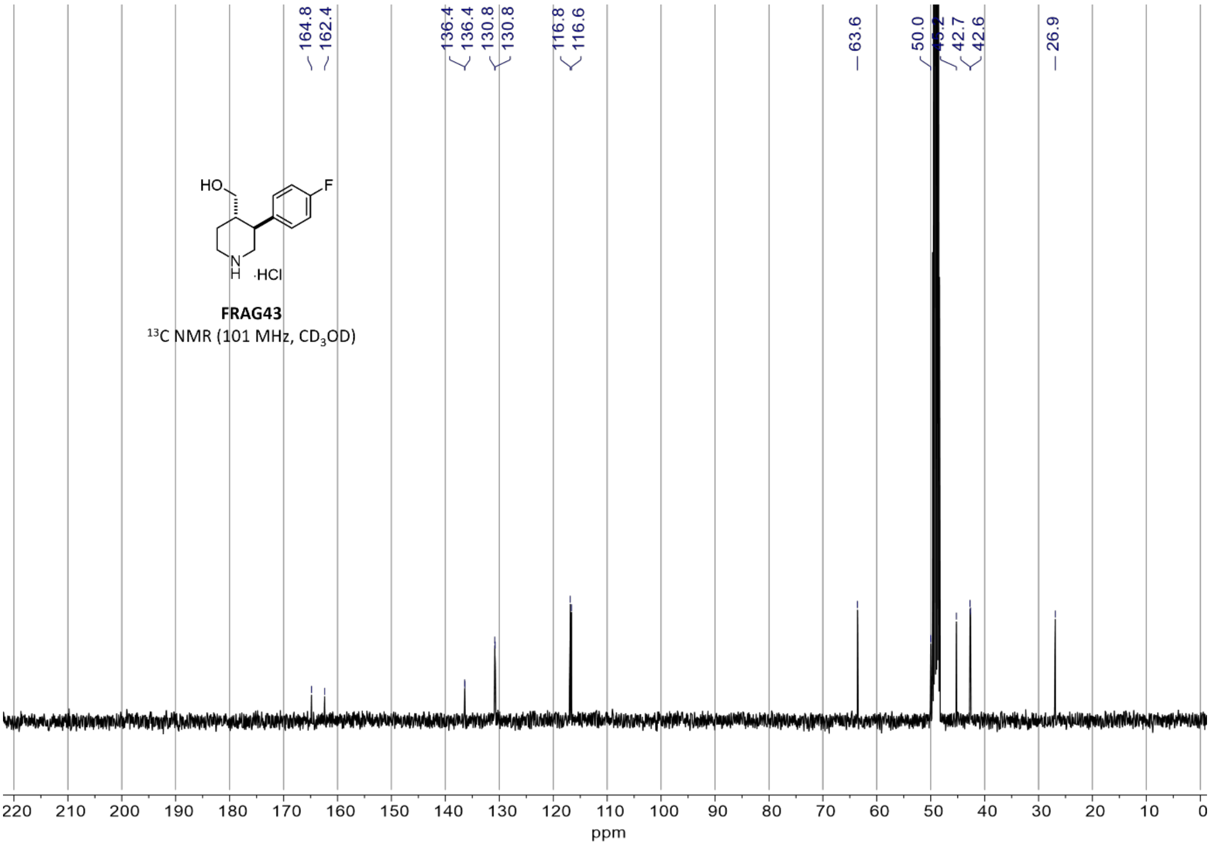

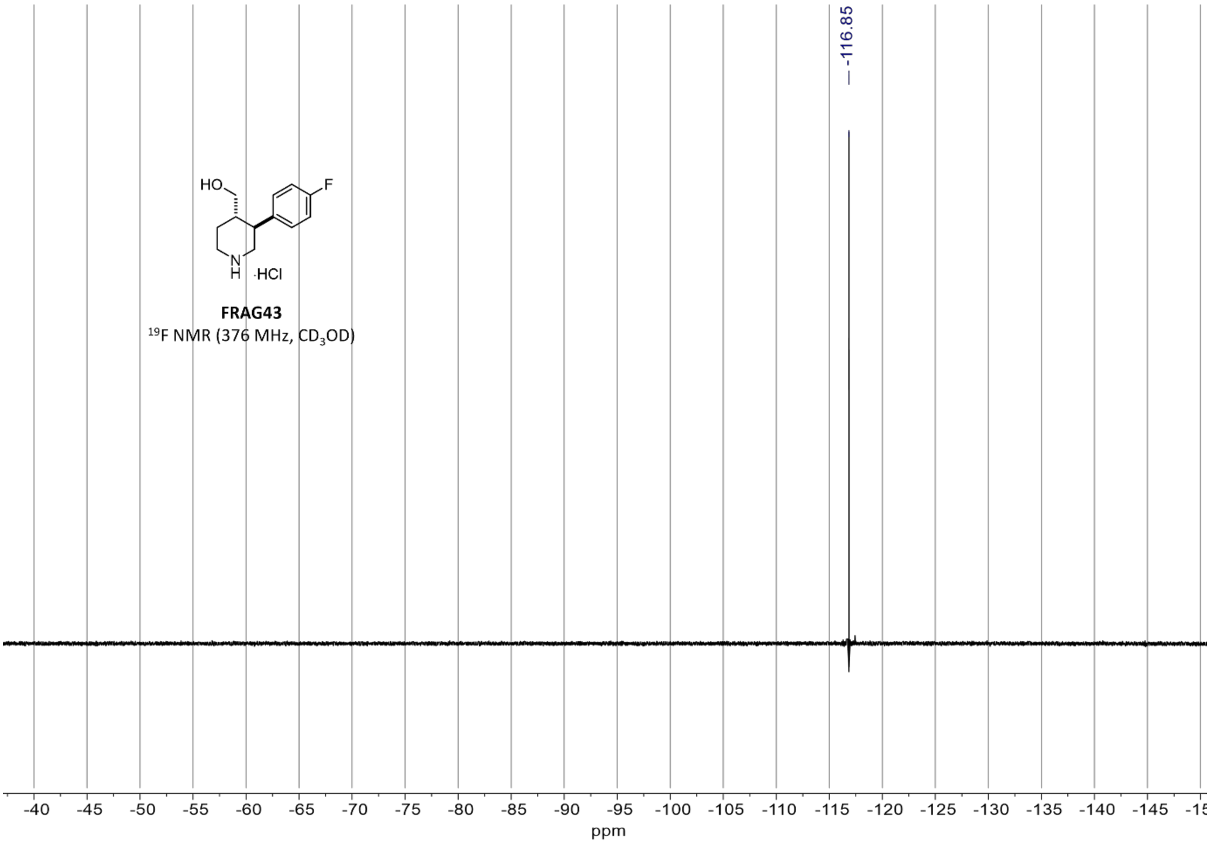
-116.85
HO
F
N
H
·HCl
FRAG43
^{19}F NMR (376 MHz, CD_3OD)
-40 -45 -50 -55 -60 -65 -70 -75 -80 -85 -90 -95 -100 -105 -110 -115 -120 -125 -130 -135 -140 -145
ppm

***cis*-(±)-3-(4-Methoxyphenyl)tetrahydro-2*H*-thiopyran-4-carboxylic acid 1,1-dioxide (FRAG46)**

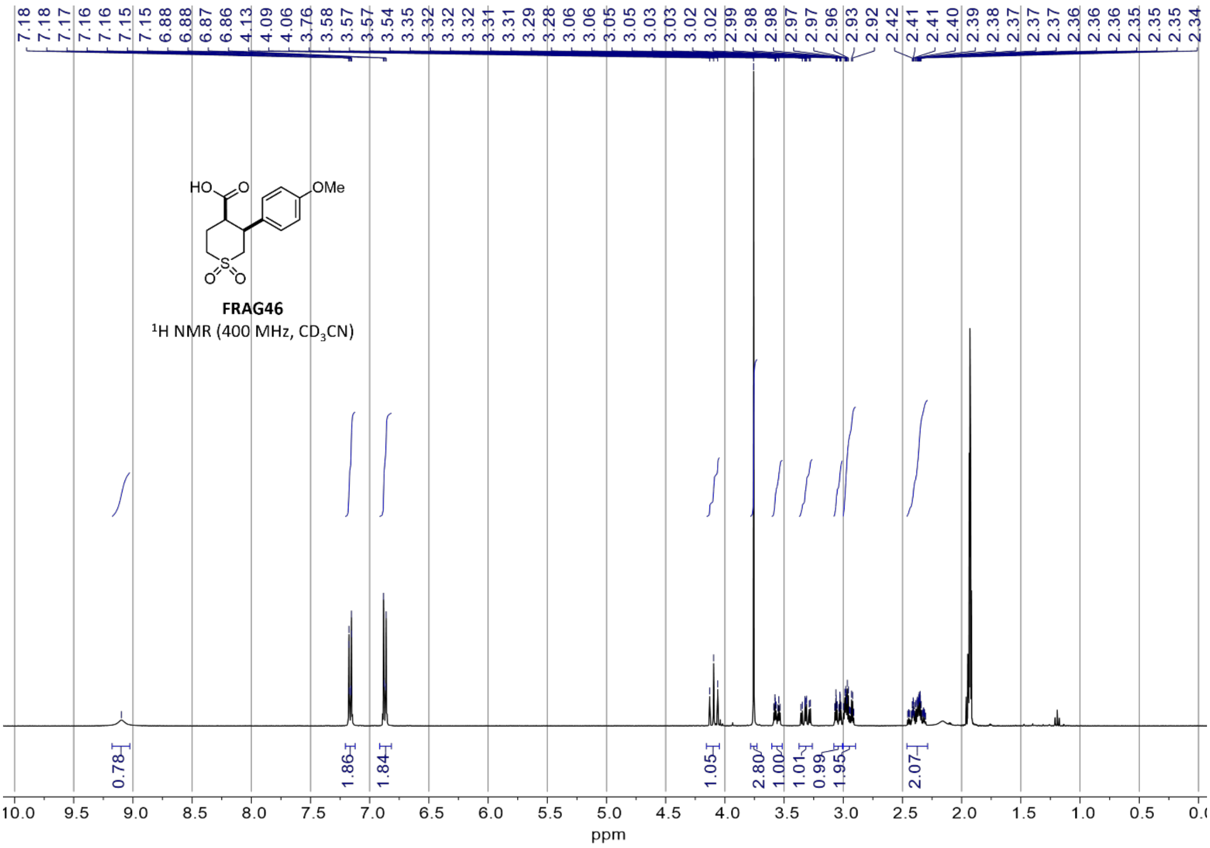

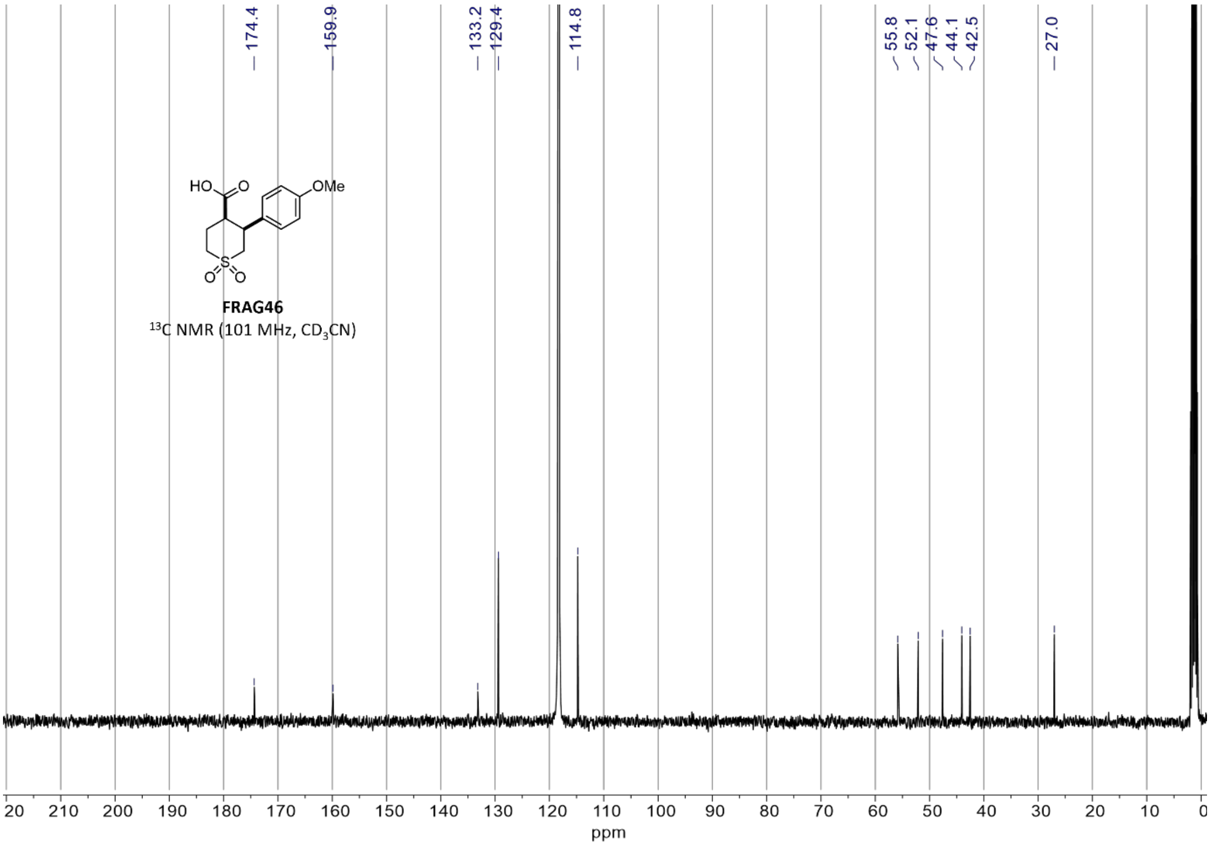

***cis*-(±)-4-(Hydroxymethyl)-3-(4-methoxyphenyl)tetrahydro-2*H*-thiopyran 1,1-dioxide (FRAG47)**

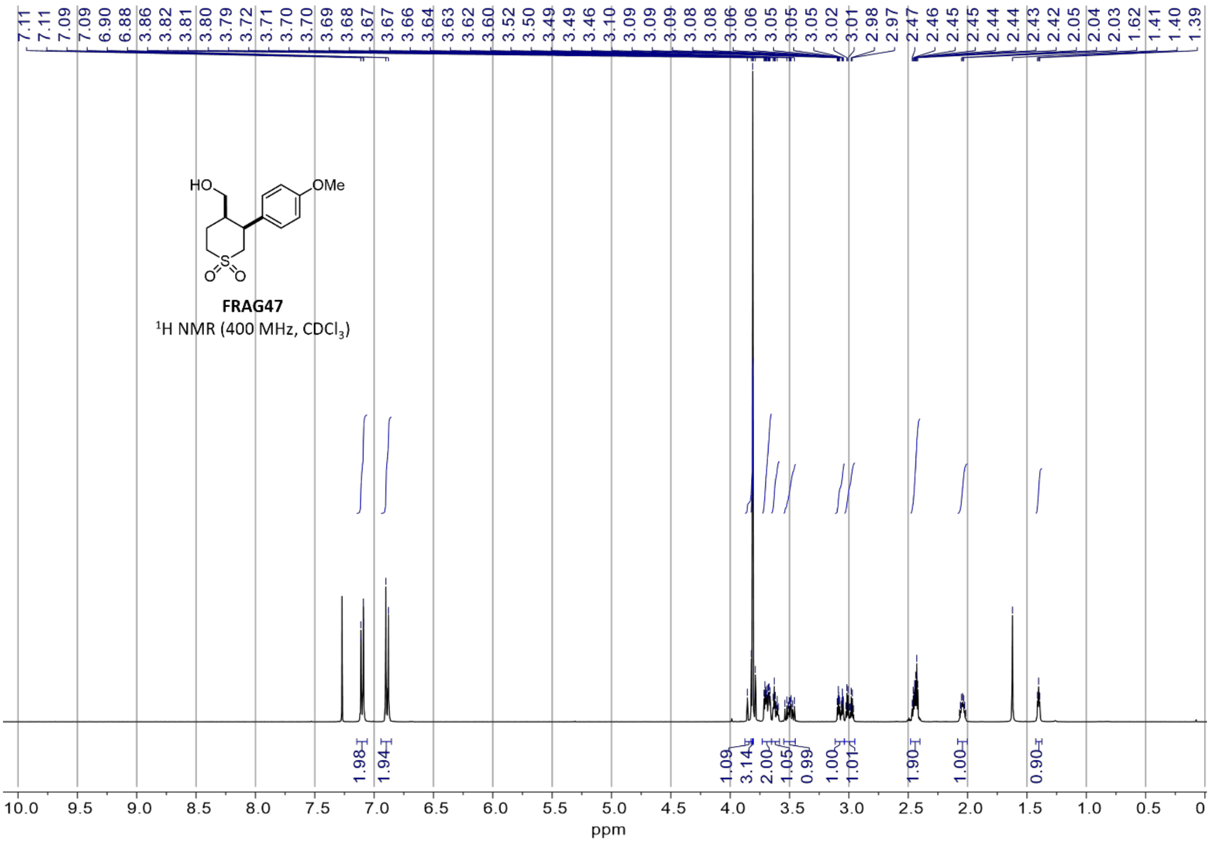

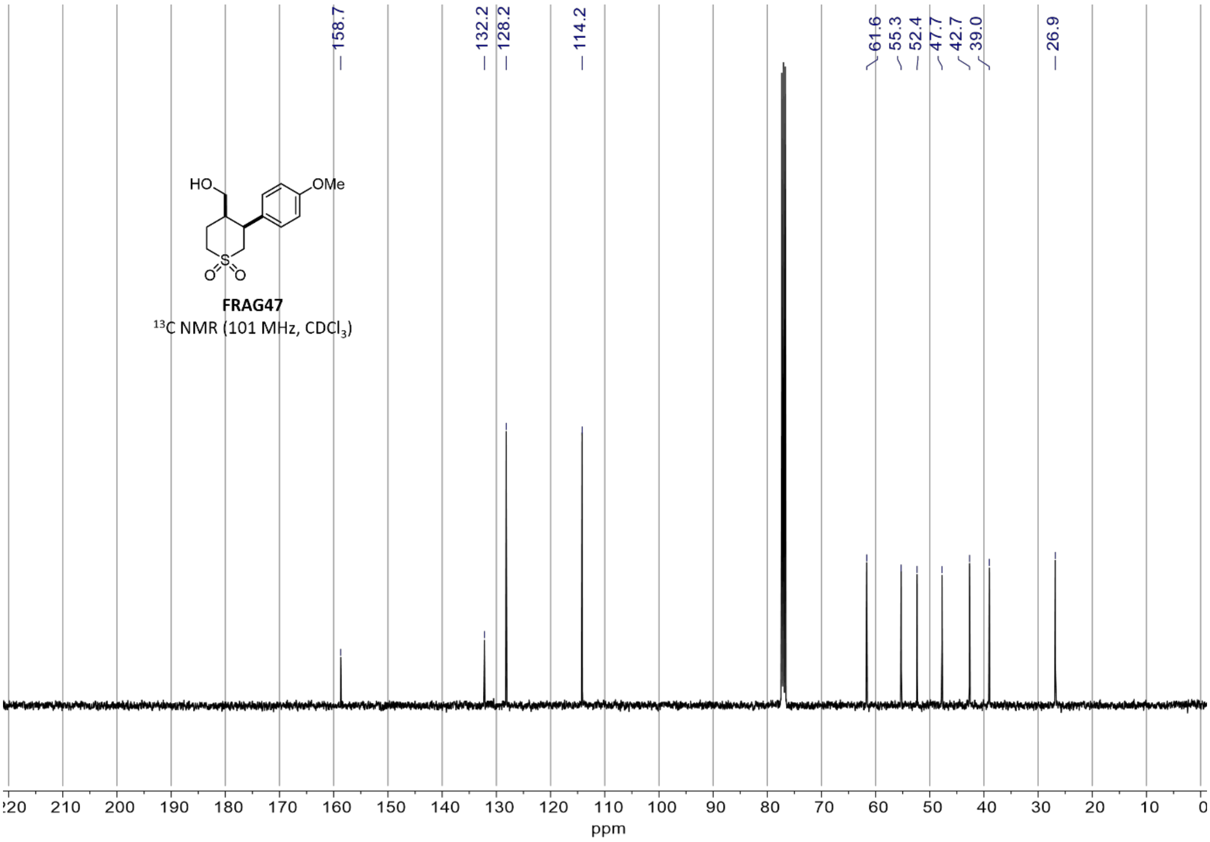

cis-(±)-4,4-Difluoro-2-(4-methoxyphenyl)cyclohexane-1-carboxylic acid (FRAG48)

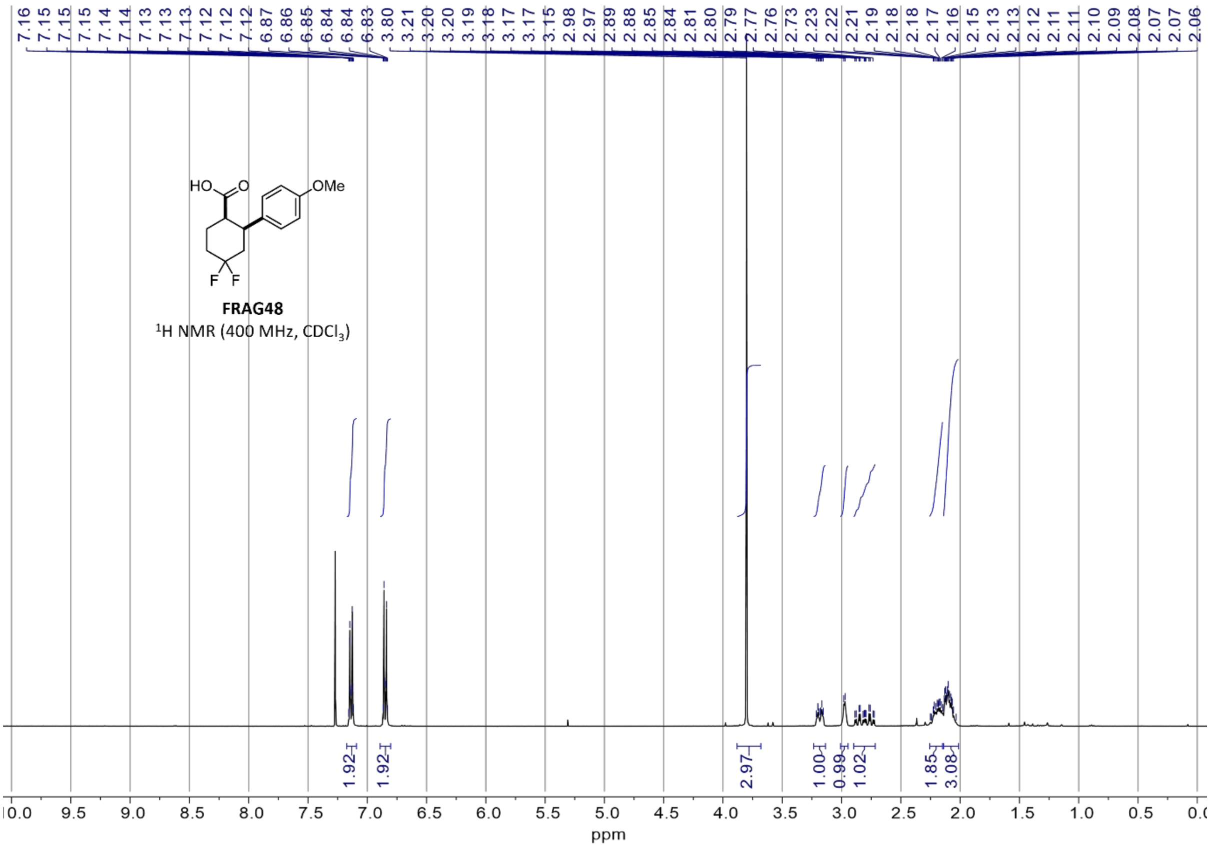

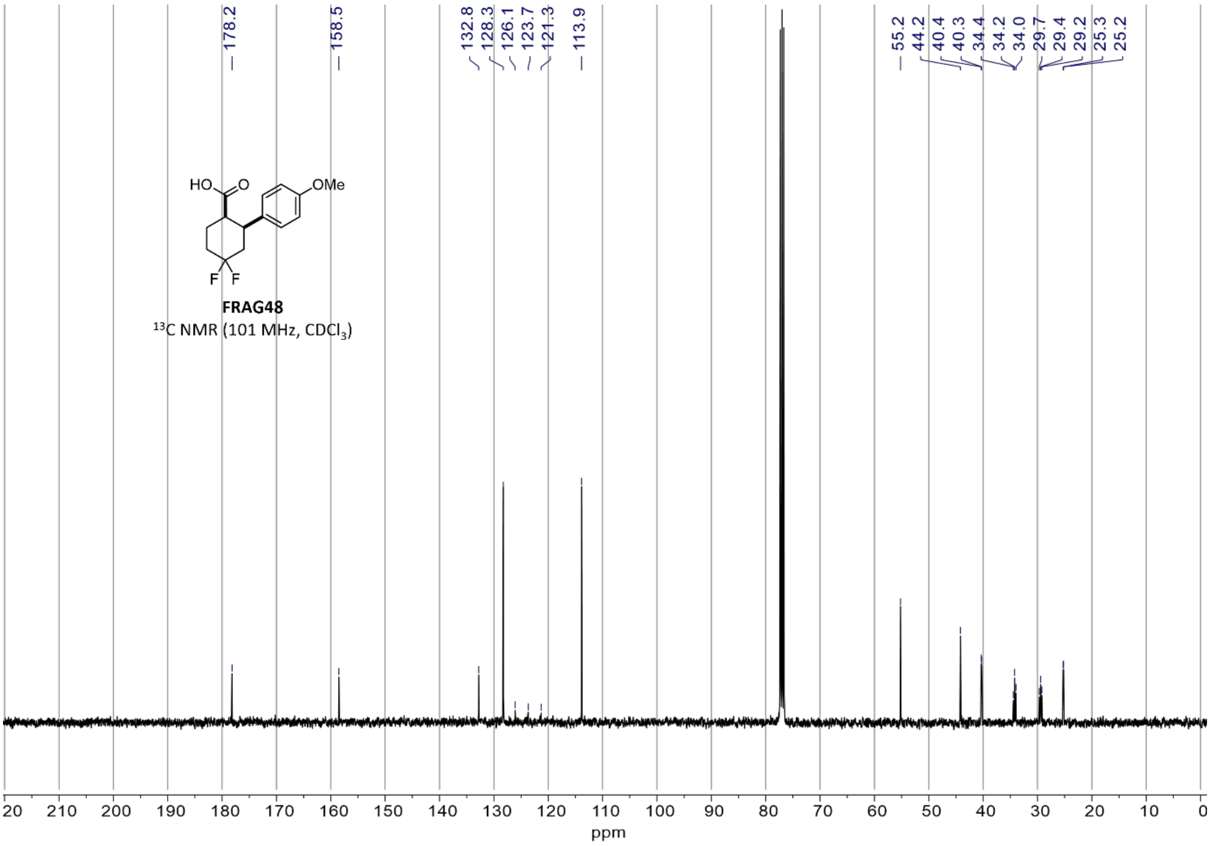

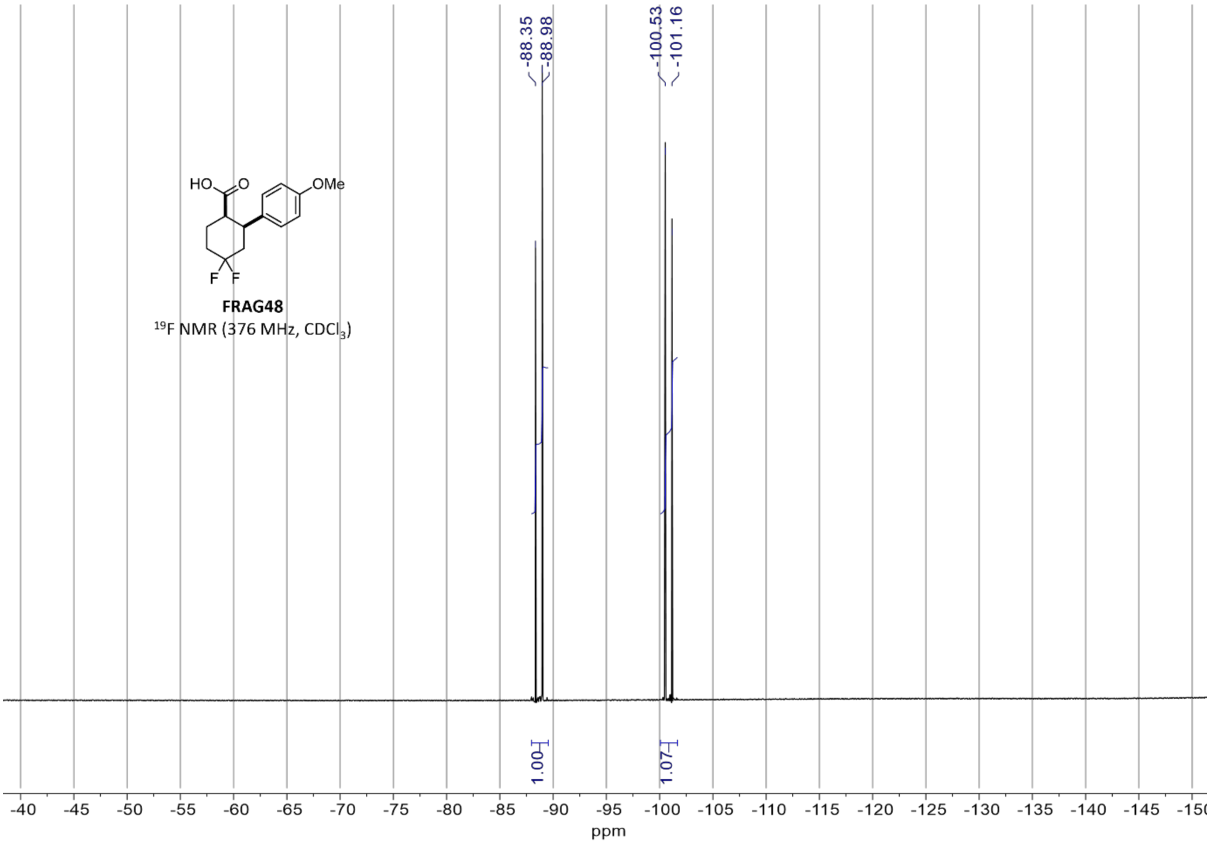

HO
O
OMe
F
F
FRAG48
^{19}F NMR (376 MHz, CDCl$_3$)
-88.35
-88.98
-100.53
-101.16
1.00
1.07
-40 -45 -50 -55 -60 -65 -70 -75 -80 -85 -90 -95 -100 -105 -110 -115 -120 -125 -130 -135 -140 -145 -150
ppm

trans-(±)-4,4-Difluoro-2-(4-methoxyphenyl)cyclohexane-1-carboxylic acid (FRAG49)

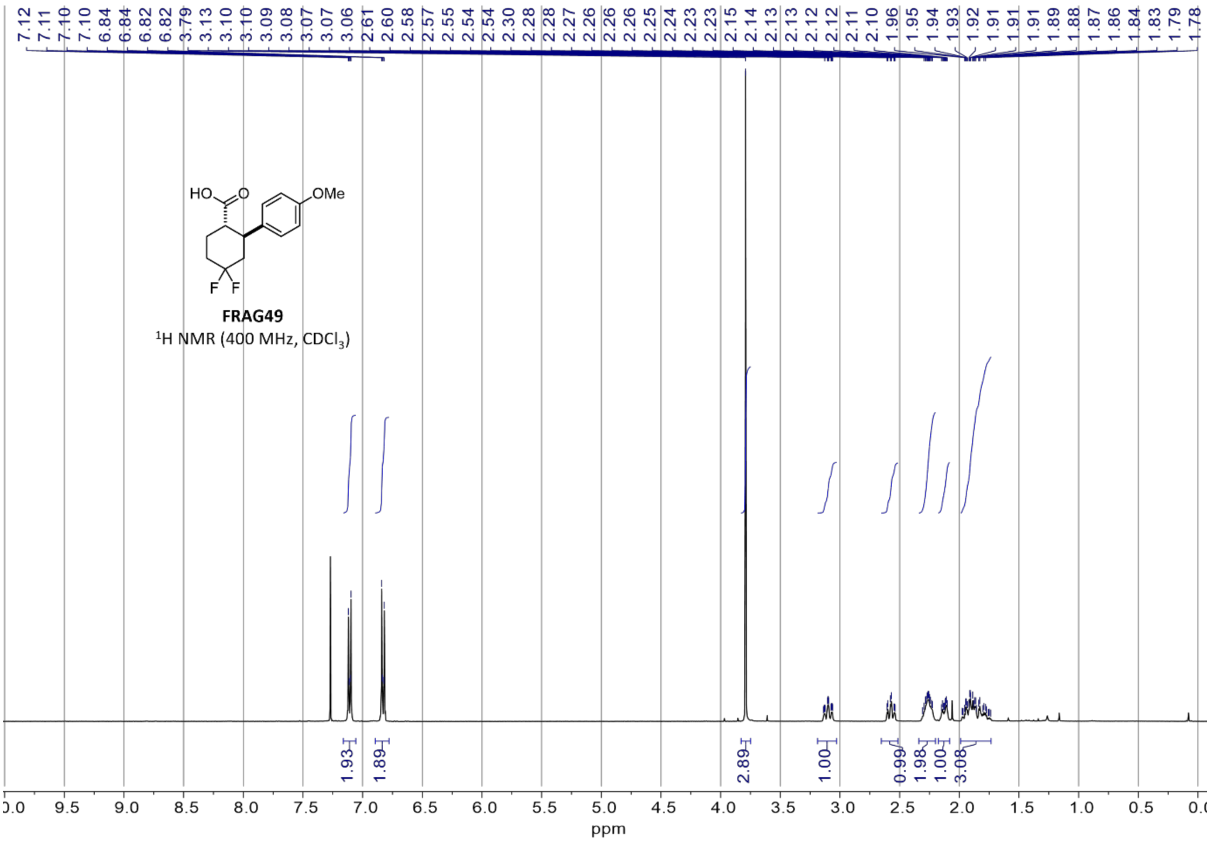

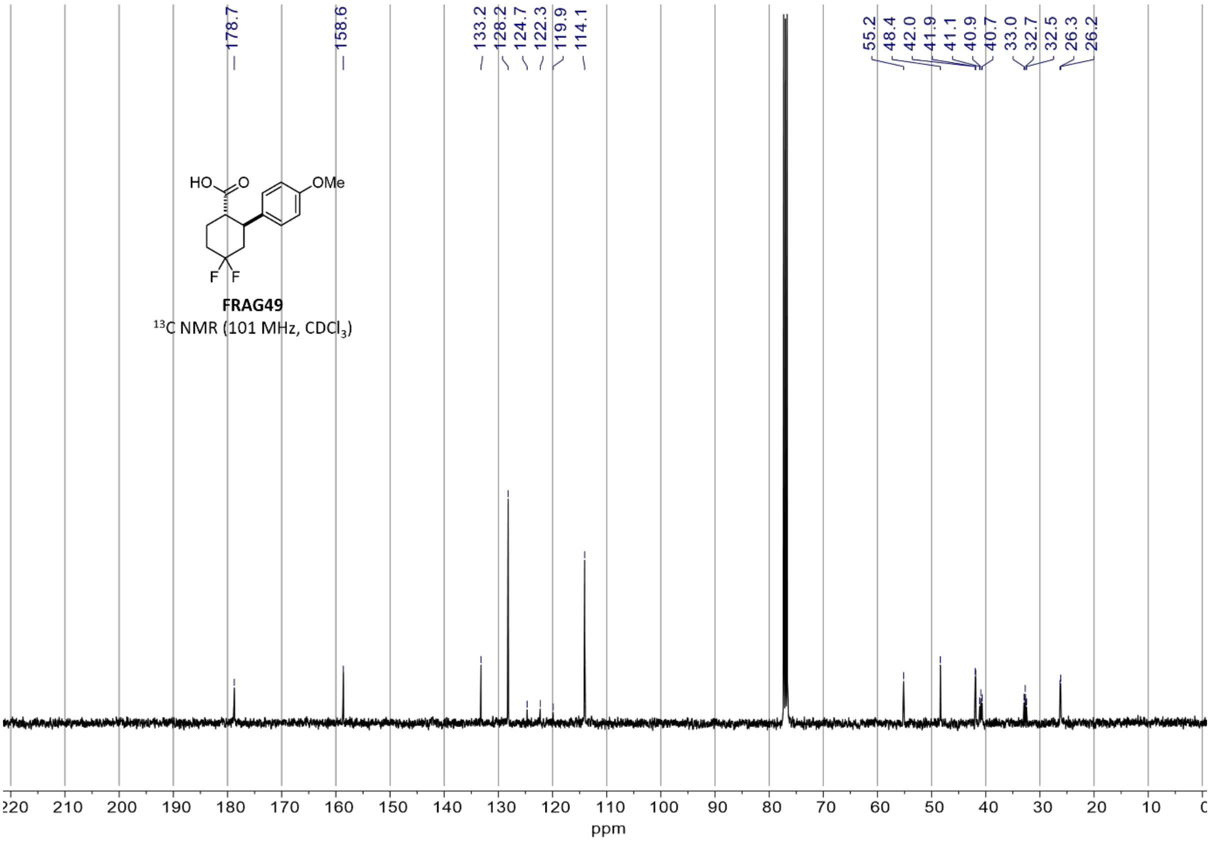

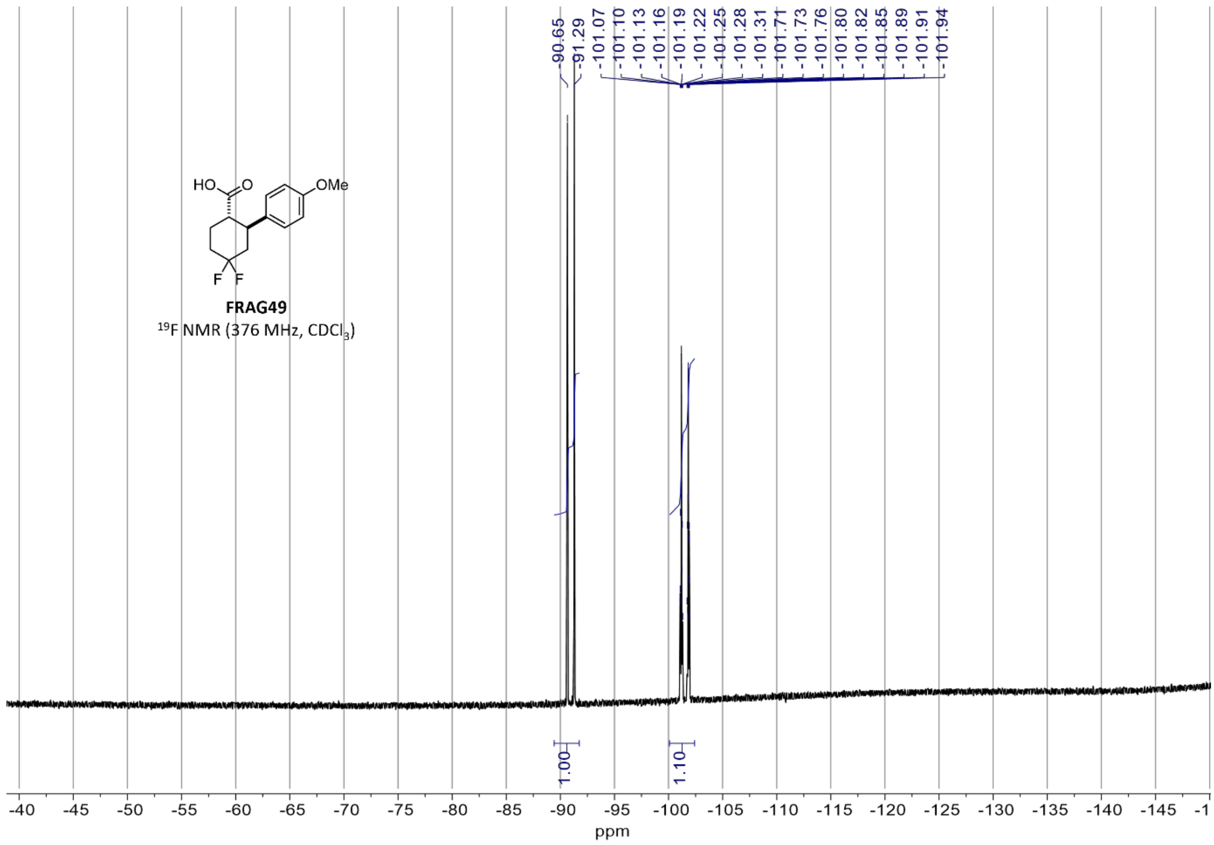

HO
O
OMe
F F
FRAG49
^{19}F NMR (376 MHz, CDCl$_3$)
-90.65
-91.29
-101.07
-101.10
-101.13
-101.16
-101.19
-101.22
-101.25
-101.28
-101.31
-101.71
-101.73
-101.76
-101.80
-101.82
-101.85
-101.89
-101.91
-101.94
1.00
1.10
-40 -45 -50 -55 -60 -65 -70 -75 -80 -85 -90 -95 -100 -105 -110 -115 -120 -125 -130 -135 -140 -145 -1
ppm

***cis*-(±)-4,4-Difluoro-2-(4-methoxyphenyl)cyclohexyl)methanol (FRAG50)**

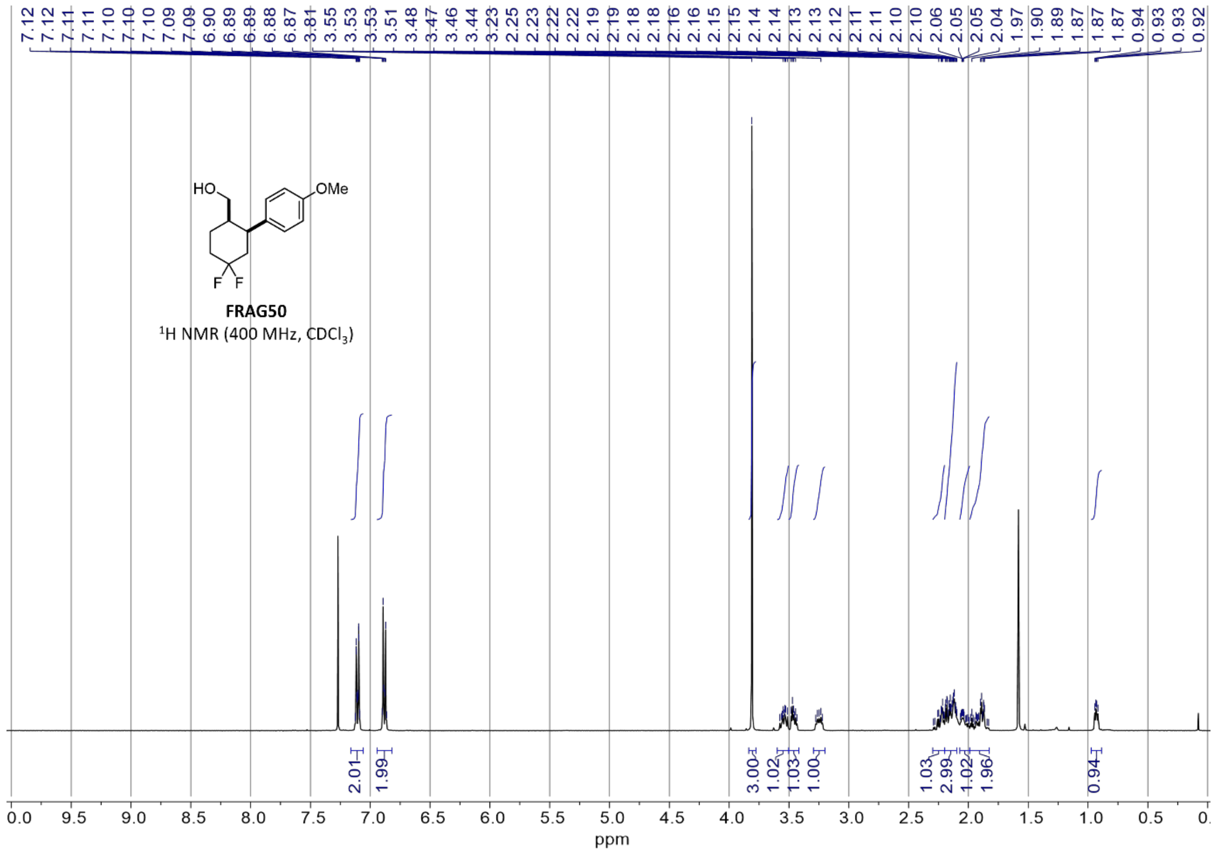

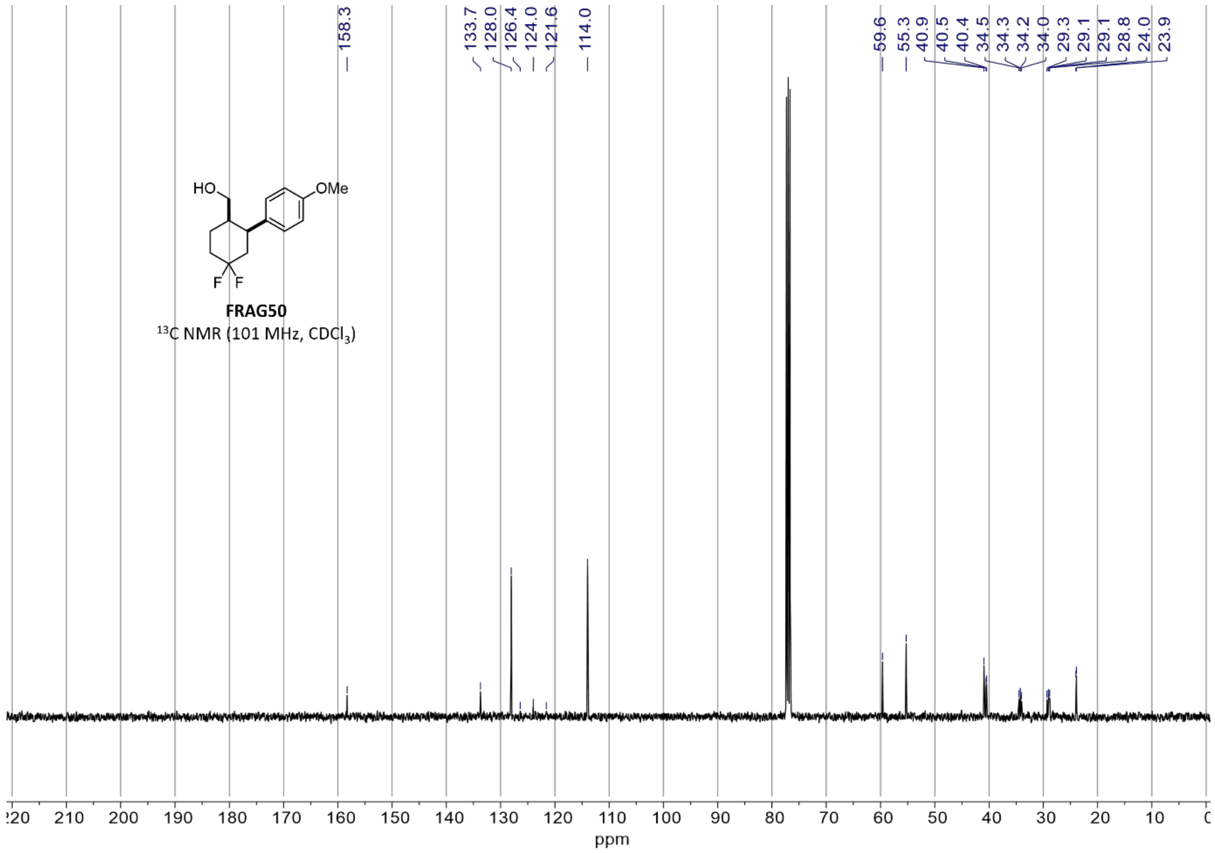

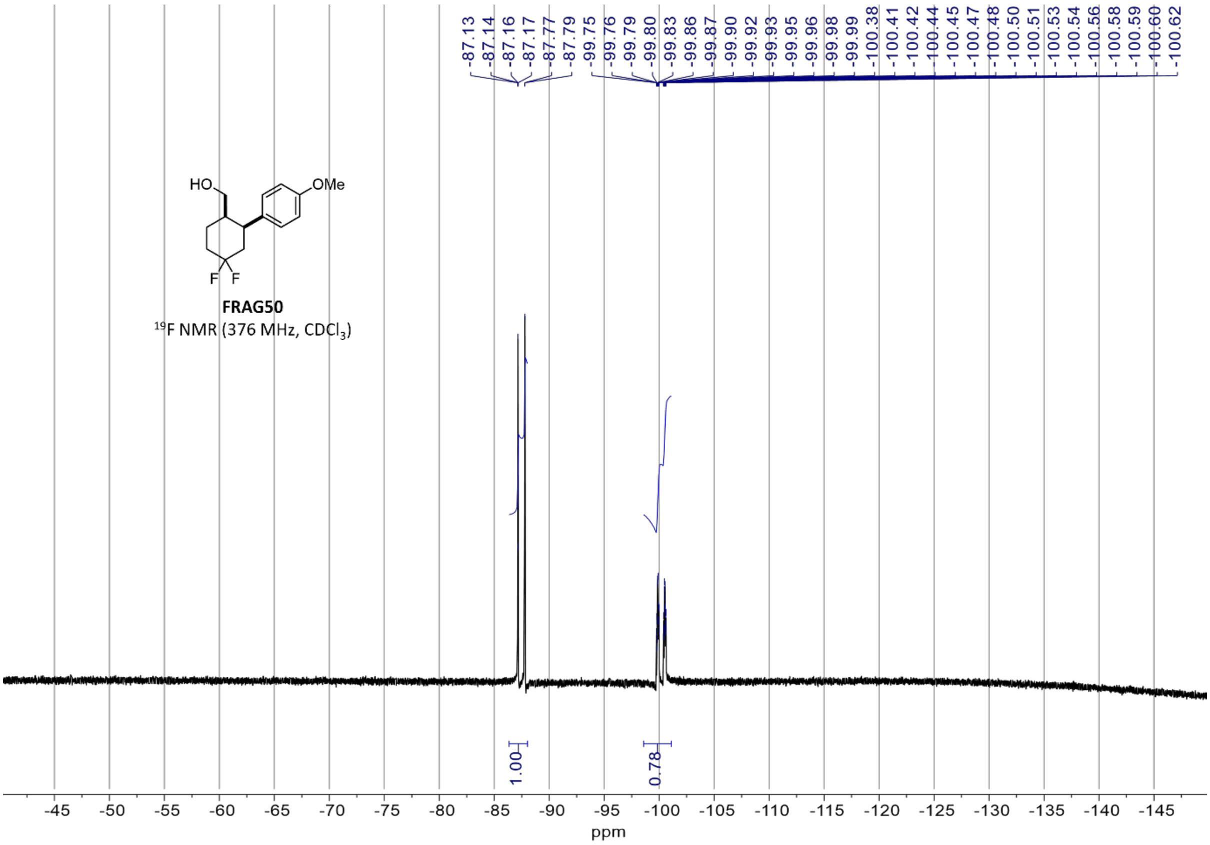

FRAG50

^{19}F NMR (376 MHz, $CDCl_3$)

***trans*-(±)-4,4-Difluoro-2-(4-methoxyphenyl)cyclohexyl)methanol (FRAG51)**

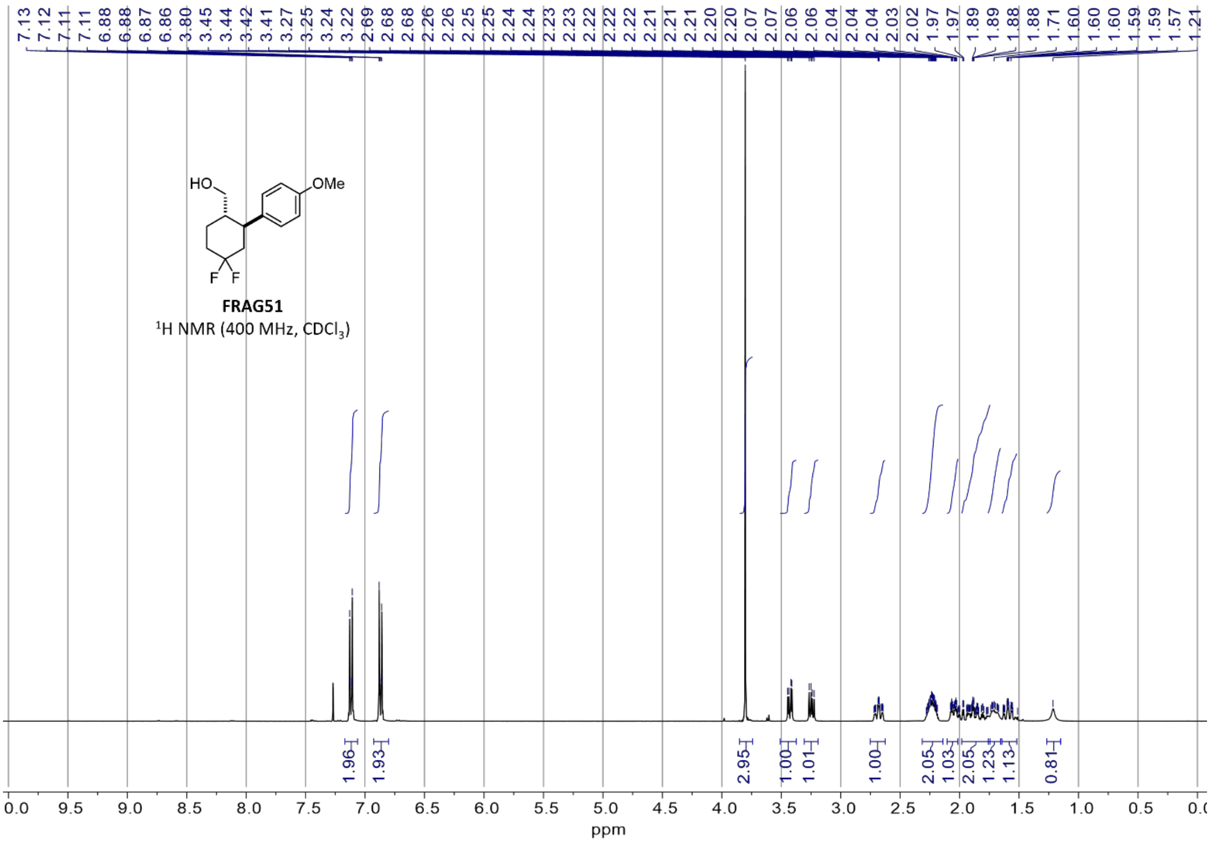

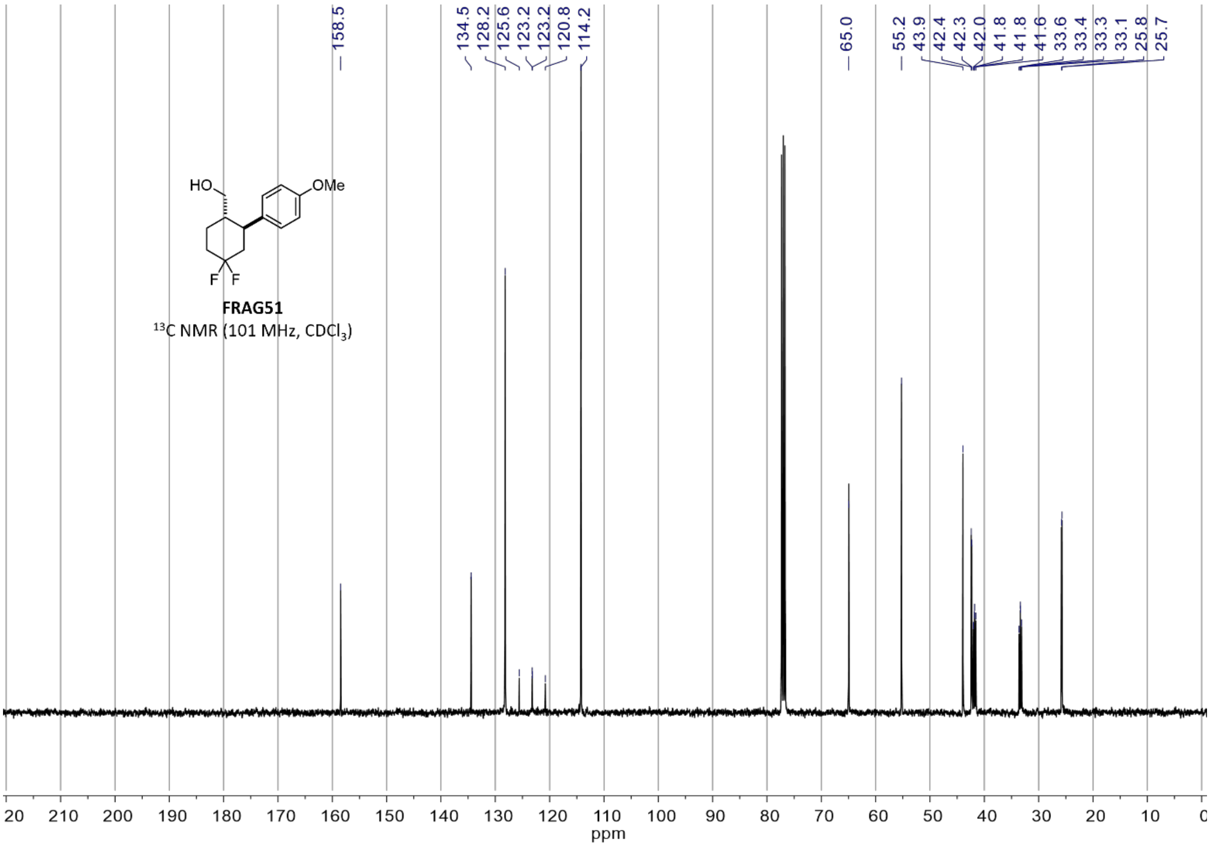

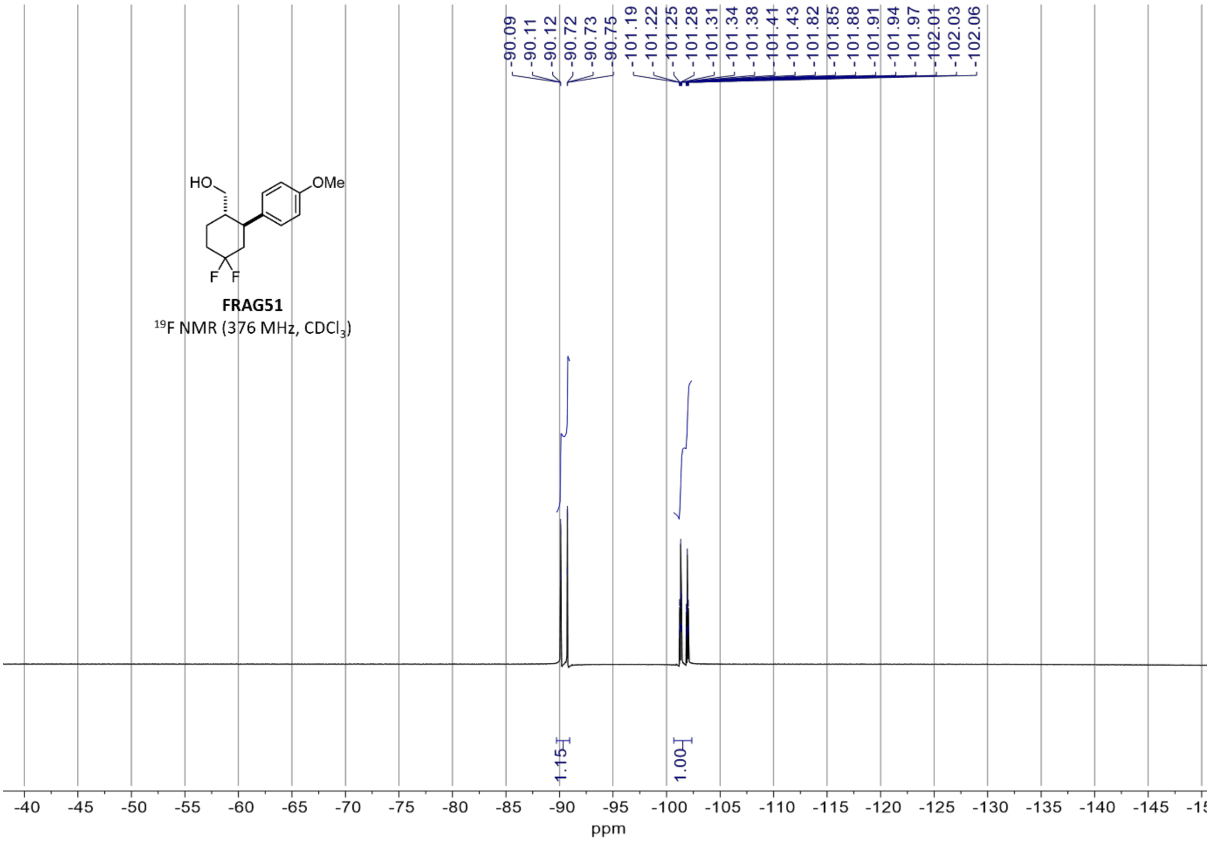
-90.09
-90.11
-90.12
-90.72
-90.73
-90.75
-101.19
-101.22
-101.25
-101.28
-101.31
-101.34
-101.38
-101.41
-101.43
-101.82
-101.85
-101.88
-101.91
-101.94
-101.97
-102.01
-102.03
-102.06
HO
OMe
F
F
FRAG51
19F NMR (376 MHz, CDCl3)
1.15
1.00
-40 -45 -50 -55 -60 -65 -70 -75 -80 -85 -90 -95 -100 -105 -110 -115 -120 -125 -130 -135 -140 -145
ppm
